# Supplementary material for: Unimolecular net heterolysis of symmetric and homopolar σ-bonds
Source: Nature. 2024 Aug 14;632(8025):550–6. doi: 10.1038/s41586-024-07622-7 (PMC11324518; doi:10.1038/s41586-024-07622-7)

nature portfolio

https://doi.org/10.1038/s41586-024-07622-7

Supplementary information

Unimolecular net heterolysis of symmetric and homopolar σ-bonds

In the format provided by the authors and unedited

Supplementary Data

Net heterolysis of symmetric and homopolar σ-bonds by stimulated doublet-doublet electron transfer

Anna F. Tiefel[1,8], Daniel J. Grenda[2,8], Carina Allacher[2], Elias Harrer[1], Carolin H. Nagel[1], Roger J. Kutta[2], David Hernández-Castillo[3,4], Poorva R. Narasimhamurthy[1], Kirsten Zeitler[5], Leticia González[3,6], Julia Rehbein[1*], Patrick Nuernberger[2,7*], Alexander Breder[1*]

[1]Institut für Organische Chemie, Fakultät für Chemie und Pharmazie, Universität Regensburg, Regensburg, Germany. [2]Institut für Physikalische und Theoretische Chemie, Fakultät für Chemie und Pharmazie, Universität Regensburg, Regensburg, Germany. [3]Institute of Theoretical Chemistry, Faculty of Chemistry, University of Vienna, Vienna, Austria. [4]Doctoral School in Chemistry (DoSChem), University of Vienna, Vienna, Austria. [5]Fakultät für Chemie und Mineralogie, Universität Leipzig, Leipzig, Germany. [6]Vienna Research Platform on Accelerating Photoreaction Discovery, University of Vienna, Vienna, Austria. [7]Regensburg Center for Ultrafast Nanoscopy (RUN), University of Regensburg, Regensburg, Germany. [8]These authors contributed equally: Anna F. Tiefel, Daniel J. Grenda.

Correspondence to: julia.rehbein@ur.de; patrick.nuernberger@ur.de; alexander.breder@ur.de

Table of Contents

1 General Remarks 5

2 Experimental setup of the photoreactions 6

3 Screening of the reaction conditions 8

3.1 SDET-induced ampholysis of PhSe–SePh 8

3.2 Atypical S_N1 aminations 11

4 Control experiments 16

4.1 C–Se bond interchange 16

4.2 Se–Se bond interchange 17

4.3 Role of the atmosphere 21

4.4 Application of the generated carbocations in other electrophilic reactions 22

5 Determination of the required number of photons and the quantum efficiency 24

5.1 Photon dependency 24

5.2 Quantum efficiency 28

6 Stationary absorption spectra in the UV/Vis 30

6.1 Spectra of 1a, 2a, 3a, and 4aa 30

6.2 Absorption spectra in the UV/Vis spectral range of TFA and HFIP 31

6.3 Absorption spectrum of a mixture of 2a and 3a 31

6.4 Generation of $PhSe^{-}$ 32

6.5 Generation of $PhSe^{+}$ 33

6.6 Generation of 3a-C^{+} 33

7 Transient absorption spectroscopy 34

7.1 Sub-ps pump/supercontinuum-probe spectroscopy 34

7.2 Transient absorption spectroscopy in the ns to ms time range 35

7.3 Generation of the cyclohexenyl radical 36

7.4 Transient absorption of diphenyl diselenide in HFIP 37

7.5 Transient absorption of 2a in MeCN 38

7.6 Influence of the acid additive on $(PhSe)_2$ 39

8 Computational calculations of the absorption spectra of the phenyl selenyl fragments 41

8.1 Computational Details: 41

8.2 Calculation of absorption spectra in the UV-Vis spectral range 41

8.3 Theoretical study of the excited states of the phenyl selenyl radical 44

9 Computational investigations of the stimulated doublet-doublet electron transfer 46

9.1 Computational Details: 46

9.2 Calculation of redox potentials: 47
10 Computational investigations of the scrambling mechanism... 49
10.1 Computational Details:.. 49
10.2 Bond dissociation and interaction energies.. 49
10.3 Chemical stability of the phenyl selenyl radical in solution 51
10.4 Radical addition elimination mechanism 52
11 Assignment of the regioisomers.. 53
11.1 1*H*-Benzotriazol (3a).. 54
11.2 5-Methyl-1*H*-benzo[*d*][1,2,3]triazole (3b).. 54
11.3 5-Chloro-1*H*-benzo[*d*][1,2,3]triazole (3c).. 56
11.4 1*H*-[1,2,3]triazolo[4,5-*b*]pyridine (3d).. 57
11.5 1*H*-Tetrazole (3e) 60
11.6 5-Methyl-1*H*-tetrazole (3f).. 61
11.7 5-Phenyl-1*H*-pyrazole (3i) 62
11.8 Valsartan (3u)... 63
12 Selectivity of the atypical S_N1-amination reactions 64
12.1 1*H*-Benzotriazol 64
12.2 Aniline... 64
12.3 4-Methylbenzenesulfonamide 65
13 Synthesis and analytical data of starting materials.. 66
14 Synthesis and analytical data of lactonisation products 73
15 Synthesis and analytical data of substitution products... 76
16 References 92
17 ^{1}H-/^{13}C-/^{77}Se-/^{19}F-/IR-spectra 96

1 General Remarks

Oxygen-free reactions were carried out under an inert gas (nitrogen, argon) atmosphere using pre-dried glassware unless otherwise indicated. Dry solvents were obtained from a solvent purification system. Chemicals were obtained from commercial sources and were used without further purification. Common solvents, such as ethyl acetate, petroleum ether, diethyl ether, and dichloromethane were purified by distillation before use. If no specific compound is stated, concentrations (in M = $\frac{mol}{L}$) of reactions are referring to the limiting starting material.

Thin layer chromatography was performed with TLC precoated aluminum sheets (ALUGRAM® Xtra SIL G/UV254 from MACHEREY-NAGEL GmbH & Co. KG, thickness 0.2 mm). Visualization was accomplished by UV light (λ = 254 nm) and Potassium Permanganate, *p*-Anisaldehyde, Cerium Ammonium Molybdate and Vanillin stain. Column chromatography was performed with silica gel (Acros, grain size 0.035–0.070 mm, 60 Å) using forced flow or a puriFlash system by Advion (Model Type: PF 5.050, Serial Number: PF-5050-2238).

For Infrared spectroscopy, all compounds were measured neat using an Agilent Technologies Cary 630 FTIR spectrometer equipped with a Diamond Single Reflection ATR-System.

High resolution mass spectra (HRMS) of the purified products were recorded either on a Jeol Accu TOF GCX, Agilent Q-TOF 6540 UHD or a ThermoQuest Finnigan TSQ 7000 by the Central Analytical Laboratory (University of Regensburg).

Melting points were measured on a KRÜSS M5000 capillary melting point apparatus, values are uncorrected.

^{1}H NMR and ^{13}C NMR spectra were recorded on Bruker Avance 300 (300 MHz (^{1}H), 75 MHz (^{13}C)), a Bruker Avance 400 (400 MHz (^{1}H), 101 MHz (^{13}C)) or a Bruker Avance III HD 400 (400 MHz (^{1}H), 101 MHz (^{13}C)) NMR spectrometer. 2D experiments were recorded on Bruker Avance 400 (400 MHz, 101 MHz) and Bruker Avance III HD 400 (400 MHz, 101 MHz) spectrometer. ^{77}Se NMR spectra were recorded on a Bruker Avance III HD 400 (76 MHz) spectrometer. ^{19}F NMR spectra were recorded on a Bruker Avance III HD 400 (377 MHz) spectrometer. The ^{1}H NMR spectra were recorded in $CDCl_3$ (δ = 7.26 ppm) and therefore the chemical shifts are reported as δ, parts per million (ppm), relative to the signal of $CDCl_3$ (δ = 7.26 ppm). The chemical shifts for ^{13}C NMR are reported as δ, parts per million (ppm), relative to the signal of $CDCl_3$ (δ = 77.0 ppm). Spectra were evaluated in 1st order and the coupling constants are described in Hertz (Hz). Splitting patterns for the spin multiplicity are described in abbreviations: s = singlet, d = doublet, t = triplet, q = quartet, p = quintet/pentet, hept = heptet/septet, m = multiplet).

All stationary UV/VIS-spectra were measured using a UV-1800 spectrometer from Shimadzu or a Cary60 spectrometer from Agilent in quartz cuvettes from Starna or Hellma with an optical path length of 10 mm.

2 Experimental setup of the photoreactions

All photoreactions were carried out in a temperature-controlled reaction setup custom made by the department for precision mechanics of the University of Regensburg (Figure S1). The reactor was either cooled through the tap water cooling system of the University to 19 °C or heated through a JULABO 220F circulator with a water/glycol (1:1) mixture. A cryostat using the same system allowed cooling below room temperature. The cooling/heating-block was built to carry six 40 mL photoreaction vials that were illuminated by the respective light-emitting diodes (LEDs) from below. To ensure reproduceable reaction conditions, all outside light sources were excluded by a cardboard mantle that was put around the reaction vial. The vials were sealed by a septum that was connected through a cannula to a balloon filled with compressed air or perforated with cannulas to guarantee a realistic air atmosphere. Different atmospheres (e.g. nitrogen, argon, pure oxygen) were carried out with a balloon inflated with the respective gas also connected to the vial through a cannula.

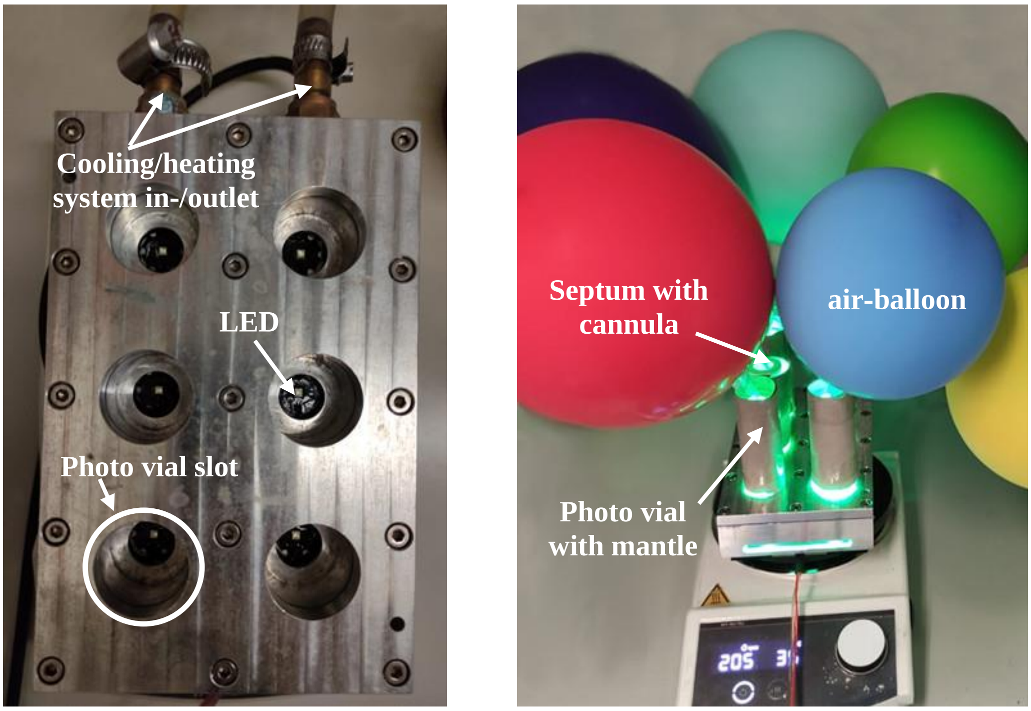

Figure S1: Purpose-built photo reactor layout (left) and reaction set-up (right).

The photoreactions were performed using different light sources, custom made by the electrical workshop of the University of Regensburg. Emission spectra of the LEDs (Figure S2) used in the reaction optimisation and scope expansion were recorded using a fiber-coupled spectrometer of the type OceanOptics USB2000+ to determine the true emission/irradiation window. Further technical data about the LEDs can be found in Table S1.

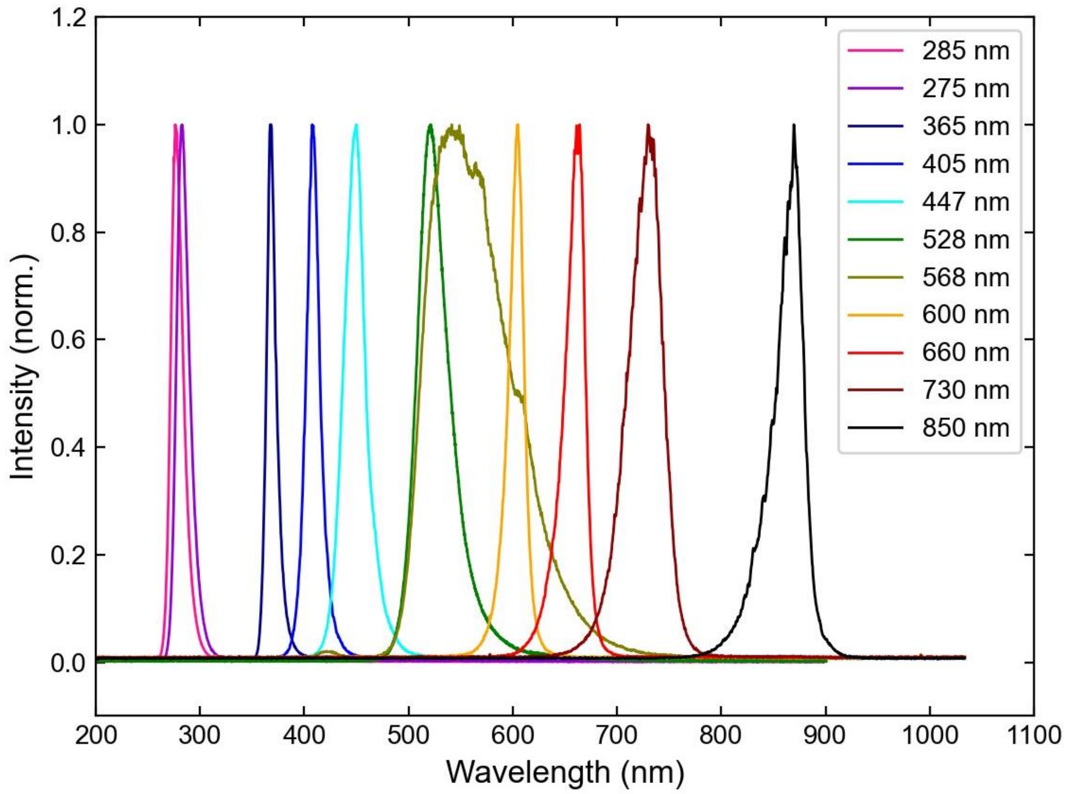

Figure S2: Emission spectra of LEDs used in the experiments.

Table S1: LEDs used in the experiments.

	**Amperage**	**Voltage**	**Specification**	**Emission Window**
**275 nm**	0.1 A	5.9 V		255–315 nm
**285 nm**	0.35 A	5.5 V		260–325 nm
**365 nm**	0.7 A	24 V	LT-2575	350–420 nm
**405 nm**	0.7 A	22 V	NVSU233B UV	375–460 nm
**447 nm**	0.7 A	17.7 V	LT-1013	400–520 nm
**528 nm**	0.7 A	21 V	LT-1966	475–650 nm
**568 nm**	0.7 A	2.75 V	LT-3978	470–730 nm
**600 nm**	0.7 A	2.5 V		555–645 nm
**660 nm**	0.7 A	2.6 V	LT-1974	580–710 nm
**730 nm**	0.7 A	2.3 V	LT-2520	620–810 nm
**850 nm**	0.7 A	2.9 V	LT-2003	760–930 nm

3 Screening of the reaction conditions

3.1 SDET-induced ampholysis of PhSe–SePh

A photoreaction vial was charged with 5-phenylpent-3-enoic acid (**6a**), diphenyl diselenide (**1a**), additives and the appropriate solvent. The reaction was stirred for 24 h at 19 °C with an atmosphere of ambient air and irradiated. The NMR standard 1,3-dinitrobenzene was added, and the solvent was removed under reduced pressure. The yields and conversions were determined via ^{1}H NMR spectroscopy.

Table S2: Optimisation of the selenium catalysed lactonisation via ambipolar Se-species

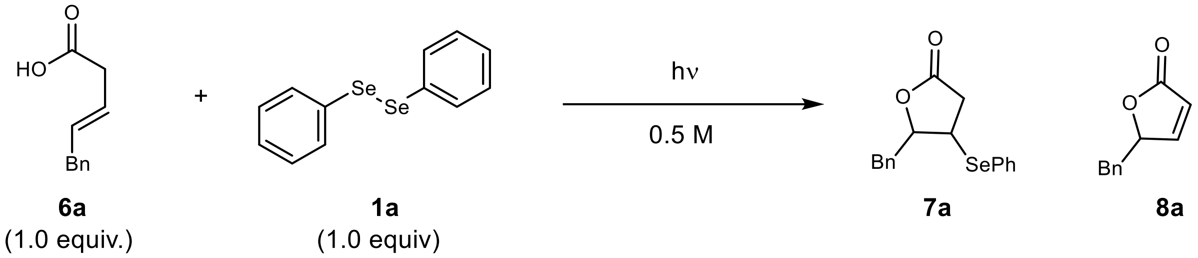

Entry	wavelength	solvent	additive	Conversion (%)[a]	Yield **7a** (%)[a]	Yield **8a** (%)[a]
1	528 nm	HFIP	TFA	61	27	0
2	447 nm	HFIP	TFA	77	72	0
3	447 nm	HFIP	MSA	100	100	<1
4	447 nm	HFIP	–	100	61	21
5	–	HFIP	TFA	8	3	0
6	–	HFIP	MSA	7	4	0
7	447 nm	^{i}PrOH	MSA	59	47	0

[a]Yields determined via ^{1}H NMR spectroscopy and internal standard 1,3-dinitrobenzene.

Catalytic application:

A photo reaction vial was charged with 5-phenylpent-3-enoic acid (**6a**), diphenyl diselenide (**1a**) as catalyst, additives, and the appropriate solvent. The reaction was stirred for a certain amount of time at the noted temperature under air and irradiated with a certain wavelength. The NMR standard 1,3-dinitrobenzene was added, and the solvent was removed under reduced pressure. The yields and conversions were determined via ^{1}H NMR spectroscopy as an average of two trials with the internal standard. (Table S3–Table S9)

Table S3: Wavelength optimisation

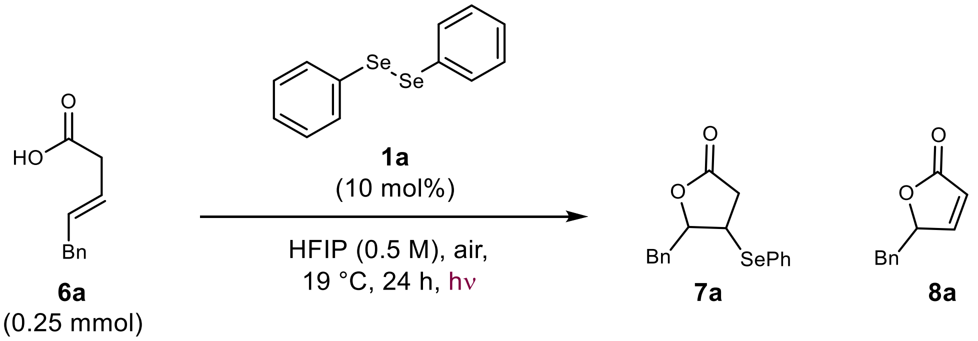

Entry	wavelength	Conversion (%)[a]	Yield **7** (%)[a]	Yield **8** (%)[a]
**1**	**447 nm**	**90**	**20**	**32**
2	528 nm	48	21	7
3	405 nm	99	11	64
4	365 nm	100	5	60

[a]Yields determined via ^{1}H NMR spectroscopy and internal standard 1,3-dinitrobenzene as an average of two trials.

Table S4: Solvent optimisation

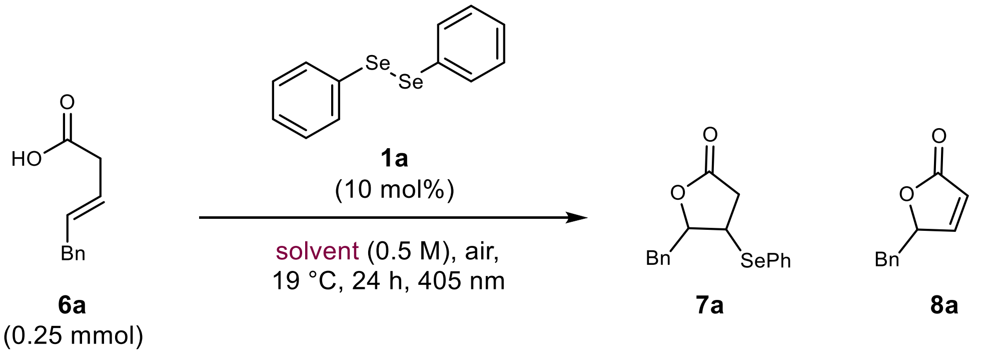

Entry	solvent	Conversion (%)[a]	Yield **7** (%)[a]	Yield **8** (%)[a]
**1**	**HFIP**	**99**	**11**	**64**
2	Toluene	49	16	5
3	*Iso*-propanol	34	5	5
4	THF	31	2	0
5	DMF	39	0	0
6	Cyclohexane	51	17	6
7	Chloroform	48	14	5
8	MeCN	50	19	6
9	2,2,2,-Trifluoro ethanol	60	19	17
10	$MeNO_2$	48	19	7

[a]Yields determined via ^{1}H NMR spectroscopy and internal standard 1,3-dinitrobenzene as an average of two trials.

Table S5: Molarity optimisation

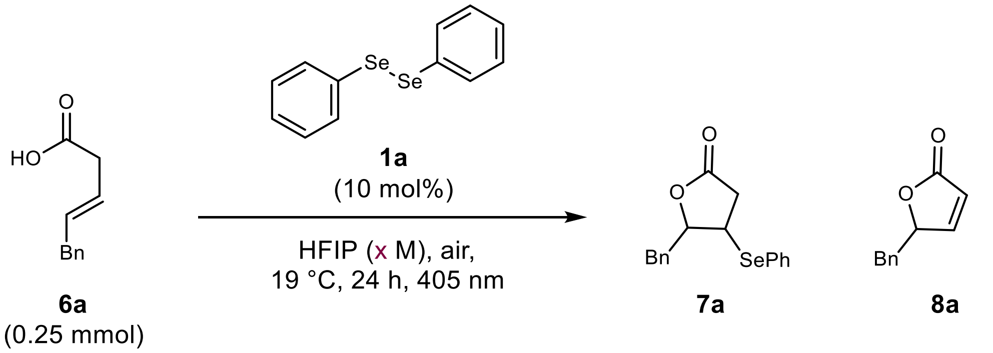

Entry	Molarity	Conversion (%)[a]	Yield **7** (%)[a]	Yield **8** (%)[a]
**1**	**0.50 M**	**99**	**11**	**64**
2	0.25 M	81	21	45
3	0.125 M	77	20	77

[a]Yields determined via ^{1}H NMR spectroscopy and internal standard 1,3-dinitrobenzene as an average of two trials.

Table S6: Catalyst loading optimisation

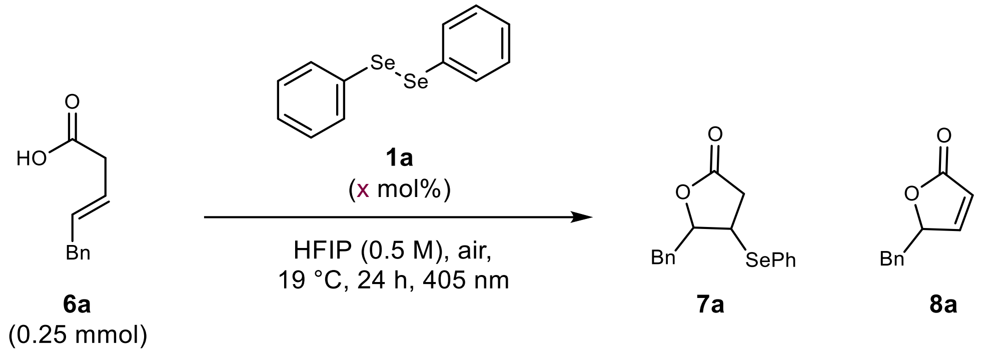

Entry	Catalyst loading	Conversion (%)[a]	Yield **7** (%)[a]	Yield **8** (%)[a]
**1**	**10 mol%**	**90**	**11**	**64**
2	20 mol%	100	15	69
**3**	**5.0 mol%**	**65**	**11**	**34**
4	2.5 mol%	57	5	25

[a]Yields determined via ^{1}H NMR spectroscopy and internal standard 1,3-dinitrobenzene as an average of two trials.

Table S7: Control experiments

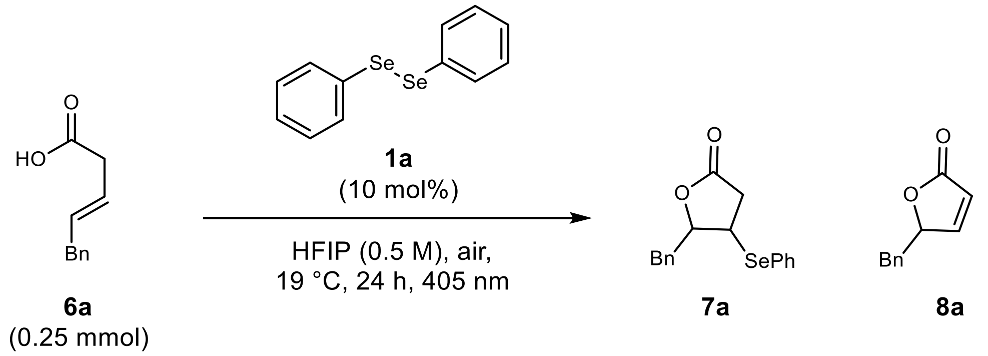

Entry	Starting material	Diselenide Catalyst	Light	Conversion (%)[a]	Yield **7** (%)[a]	Yield **8** (%)[a]
1	**Yes**	**Yes**	**405 nm**	**90**	**11**	**64**
2	No	Yes	405 nm	0	0	0
3	Yes	Yes	No	17	0	0
4 (Ar)	Yes	Yes	405 nm	78	18	18
5	Yes	No	405 nm	22	0	0

[a]Yields determined via ^{1}H NMR spectroscopy and internal standard 1,3-dinitrobenzene as an average of two trials.

Table S8: Temperature optimisation

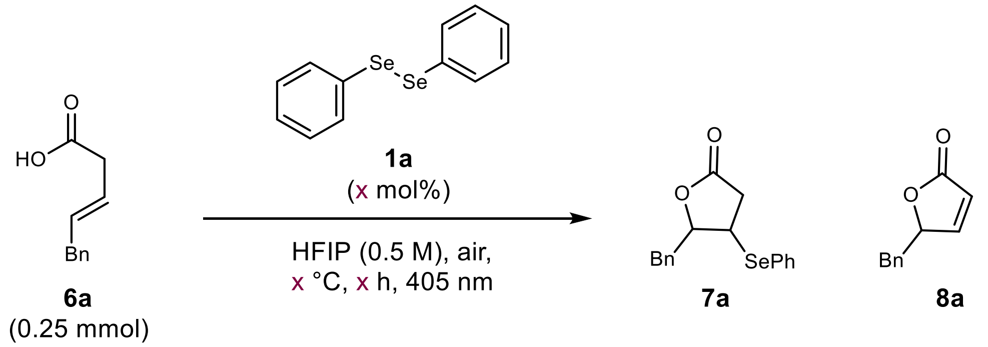

Entry	Catalyst loading	Temperature	Time	Conversion (%)[a]	Yield **7** (%)[a]	Yield **8** (%)[a]
1	10 mol%	19 °C	24 h	90	11	64
2	10 mol%	35 °C	16 h	100	11	70
3	10 mol%	50 °C	8 h	63	20	24
4	5.0 mol%	19 °C	24 h	65	11	34
**5**	**5.0 mol%**	**35 °C**	**22 h**	**100**	**4**	**76**
6	5.0 mol%	50 °C	16 h	100	7	69

[a]Yields determined via ^{1}H NMR spectroscopy and internal standard 1,3-dinitrobenzene as an average of two trials.

Table S9: Upscaling optimisation

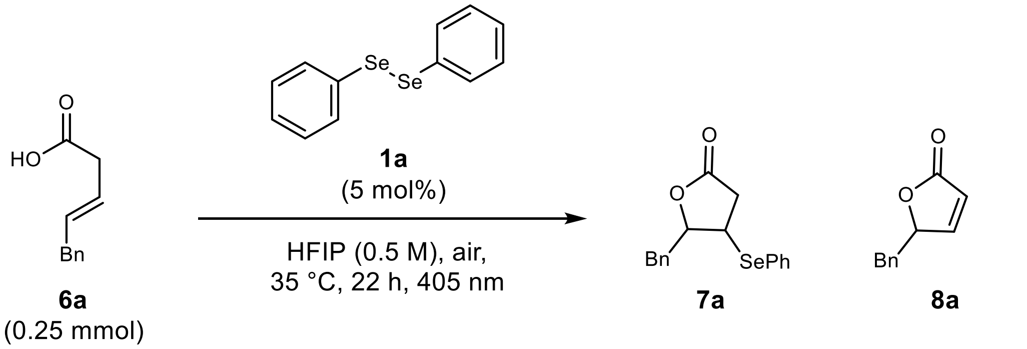

Entry	Amount of substance	Conversion (%)[a]	Yield **7** (%)[a]	Yield **8** (%)[a]
1	0.25 mmol	100	4	76
**2**	**0.50 mmol**	**96**	**11**	**66**
3	1.00 mmol	73	10	38

[a]Yields determined via ^{1}H NMR spectroscopy and internal standard 1,3-dinitrobenzene as an average of two trials.

3.2 Atypical SN1 aminations

A photo reaction vial was charged with cyclohex-2-en-1-yl(phenyl)selane (**2a**), 1*H*-benzotriazole (**3a**), additives and the appropriate solvent. The reaction was stirred for a certain amount of time at the noted temperature with a specific atmosphere and irradiated with green light. The NMR standard 1,3-dinitrobenzene was added, and the solvent was removed under reduced pressure. The yields and conversions were determined via ^{1}H NMR spectroscopy as an average of two trials with the internal standard (Table S10–Table S14).

Table S10: Stoichiometry and temperature optimisation

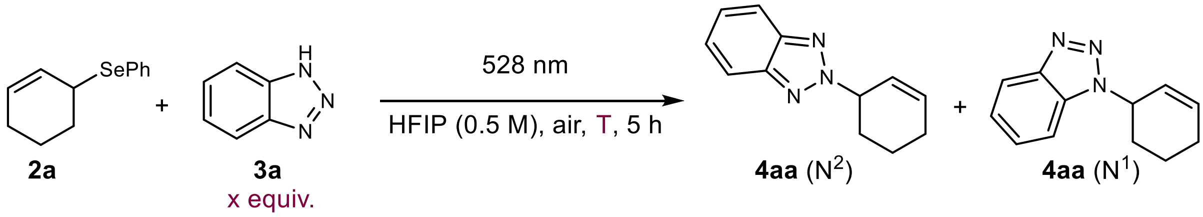

Entry	Equiv. **3a**	Temperature	Conversion (%)[a]	Total Yield (%)[a]
**1**	**0.2**	**19 °C**	**100**	**73**
2	0.33	5 °C	100	50
3	0.33	19 °C	100	66
4	0.33	35 °C	100	67
5	0.33	55 °C	100	67
6	0.5	19 °C	100	42
7	1.0	19 °C	87	34
8	2.0	19 °C	90	45
9	3.0	5 °C	68	40
10	3.0	19 °C	100	53
11	3.0	35 °C	100	50

12	3.0	55 °C	100	48
13	4.0	19 °C	97	52
14	4.0	55 °C	100	50
15	5.0	19 °C	93	51
16	5.0	55 °C	100	50

[a]Yields determined via ^{1}H NMR spectroscopy and internal standard 1,3-dinitrobenzene as an average of two trials.

Table S11: Solvent optimisation

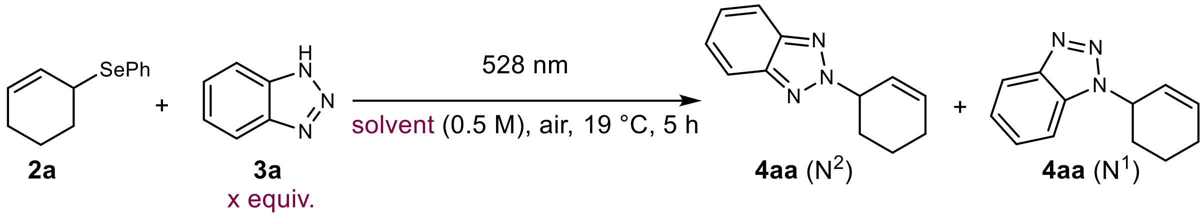

Entry	Equiv. **3a**	Solvent	Conversion (%)[a]	Total Yield (%)[a]
1	0.33	HFIP	100	66
2	3.0	HFIP	100	53
**3**	**3.0**	**HFIP**	**100[b]**	**83[b]**
4	0.33	2,2,2-Trifluoro ethanol	100	58
5	0.33	1,1,1-Trifluoro acetone	64	28
6	0.33	α,α,α-Trifluoro toluene	35	10
7	0.33	Trichloro ethanol	100	21
8	0.33	Chloro acetone	100	8
9	0.33	Ethanol	3	0
10	0.33	*Iso*-propanol	47[b]	10[b]
11	0.33	Acetone	9	2
12	0.33	Toluene	33	4
13	0.33	Chloroform	33	5
14	3.0	Chloroform	100[b]	23[b]
15	0.33	THF	55	0
16	3.0	THF	100[b]	14[b]
17	0.33	Ethyl acetate	28	0
18	0.33	MeCN	30	15
19	3.0	MeCN	100[b]	25[b]
20	0.33	$MeNO_2$	53	32
21	3.0	$MeNO_2$	100[b]	35[b]
22	0.33	DMF	0	0
23	0.33	Water	7	7
24	3.0	Water	46[b]	18[b]

[a]Yields determined via ^{1}H NMR spectroscopy and internal standard 1,3-dinitrobenzene as an average of two trials. [b]Addition of 50 mol% TFA.

Table S12: Additives optimisation

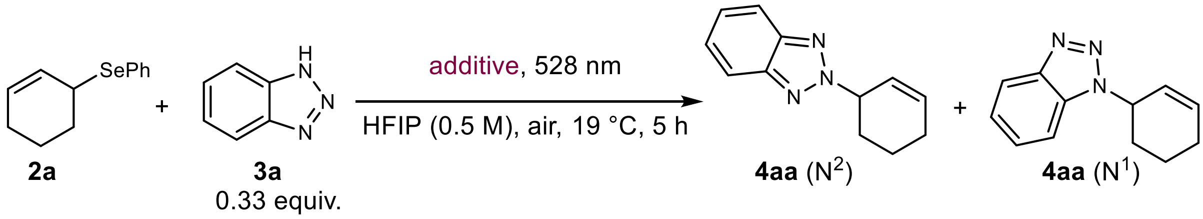

Entry	Additive	Total Yield (%)[a]
1	–	66
2	K_2CO_3 (1.0 equiv.)	35
3	K_2CO_3 (4.0 equiv.)	26
**4**	**Li_2CO_3 (1.0 equiv.)**	**76**
5	Li_2CO_3 (4.0 equiv.)	67
6	Cs_2CO_3 (1.0 equiv.)	36
7	$NaHCO_3$ (4.0 equiv.)	63

8	Na_2HPO_4 (4.0 equiv.)	69
9	NaH_2PO_4 (4.0 equiv.)	74
10	K_3PO_4 (4.0 equiv.)	13
11	Na_3PO_4 (1.0 equiv.)	54
12	Cesium pivalate (1.0 equiv.)	60
13	Sodium pivalate (1.0 equiv.)	52
14	Sodium acetate (1.0 equiv.)	51
15	Disodium oxalate (1.0 equiv.)	65
16	2,6-Di-*tert*-butylpyridine (4.0 equiv.)	20
17	NaF (4.0 equiv.)	64
18	KF (4.0 equiv.)	51
19	CsF (4.0 equiv.)	52
20	AgF (4.0 equiv.)	52
21	CaF_2 (1.0 equiv.)	63
22	CaF_2 (4.0 equiv.)	61
23	2-Nitrobenzaldehyde (1 mol%)	71
24	2-Nitrobenzaldehyde (2.5 mol%)	72
25	2-Nitrobenzaldehyde (5 mol%)	64
26	2-Nitrobenzaldehyde (10 mol%)	65
27	2-Nitrobenzaldehyde (25 mol%)	68
28	2-Nitrobenzaldehyde (50 mol%)	58
29	2-Nitrobenzaldehyde (100 mol%)	63
30	2-Nitrobenzaldehyde (200 mol%)	60
31	CaF_2 (4.0 equiv.) + 2-Nitrobenzaldehyde (5 mol%)	66
32	Li_2CO_3 (1.0 equiv.) + 2-Nitrobenzaldehyde (5 mol%)	67
33	Na_2HPO_4 (4.0 equiv.) + 2-Nitrobenzaldehyde (5 mol%)	71
34	NaH_2PO_4 (4.0 equiv.) + 2-Nitrobenzaldehyde (5 mol%)	67

[a]Yields determined via ^1H NMR spectroscopy and internal standard 1,3-dinitrobenzene as an average of two trials.

Table S13: Acid optimisation

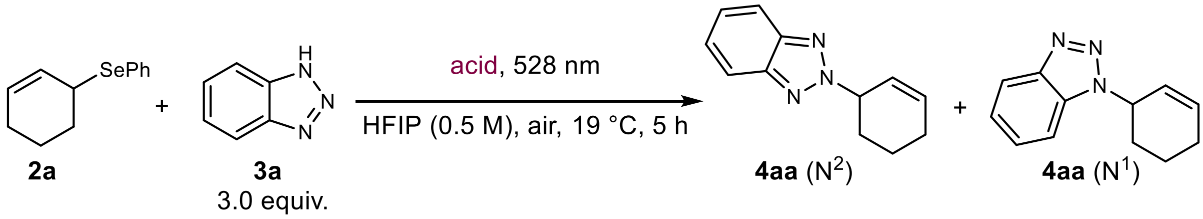

Entry	Acid	Mol% Acid	Total Yield (%)[a]
1	–	–	66
2	MSA	10	54
3	MSA	25	82
4	MSA	25	82[b]
5	MSA	50	79
**6**	**MSA**	**50**	**87[b]**
7	MSA	75	86
8	MSA	75	83[b]
9	MSA	100	71
10	TFA	10	74
11	TFA	50	83
12	TFA	100	84
13	TFA	200	84
14	*p*-Toluene sulfonic acid	50	60
15	Benzene sulfonic acid	50	76
16	Dichloroacetic acid	50	75
17	Diphenyl phosphoric acid	50	78
18	Phosphoric acid	50	58
19	Chloroacetic acid	50	59
20	Formic acid	50	54
21	Acetic acid	50	61

[a]Yields determined via ^{1}H NMR spectroscopy and internal standard 1,3-dinitrobenzene as an average of two trials. [b]Neutralisation with saturated $NaHCO_3$ solution before workup.

Table S14: Wavelength Screening

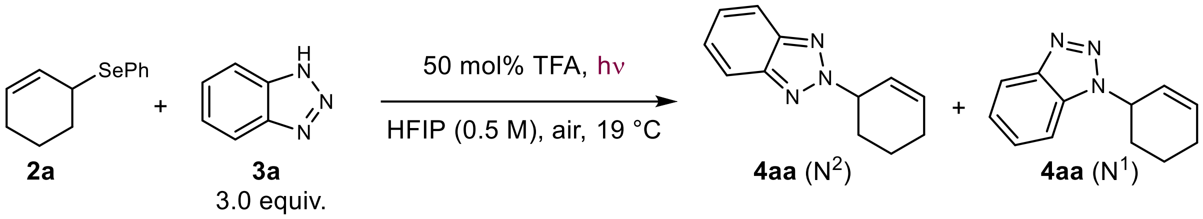

Entry	Light	Wavelength	Longpass Filter	Reaction Vessel	Time	Conversion (%)[a]	Total Yield (%)[a]
1	IR-A	850 nm	700 nm	Photo vial	9 d	8	8
2	IR-A	850 nm	380 nm	Photo vial	24 h	5	5
3	Far Red	730 nm	580 nm	Photo vial	9 d	20	18
4	Far Red	730 nm	700 nm	Photo vial	24 h	4	4
5	Red	660 nm	580 nm	Photo vial	24 h	100	84
6	Red	660 nm	630 nm	Photo vial	24 h	8	6
7	Red	660 nm	645 nm	Photo vial	24 h	9	8
8	Red	660 nm	580 nm	Photo vial	3 h	80	63
9	Red	660 nm	–	Photo vial	24 h	100	85
10	Orange	600 nm	–	Photo vial	3 h	44	35
11	Yellow	568 nm	–	Photo vial	3 h	100[b]	81[b]
12	Green	528 nm	–	Photo vial	3 h	100[b]	83[b]
13	Blue	477 nm	–	Photo vial	3 h	100[b]	81[b]
**14**	**UV**	**365 nm**	**–**	**Photo vial**	**3 h**	**100[b]**	**86[b]**
15	Green	528 nm	–	Cuvette	3 h	85	76
16	UV	285 nm	–	Cuvette	3 h	20	18
17	UV	275 nm	–	Cuvette	3 h	13	13
18	UV	275 nm	–	Cuvette	28 h	85	72
19	–	–	–	Photo vial	3 h	5	5
20	–	–	–	Photo vial	6 d	6	6
21	Room light	–	–	Photo vial	3 h	3	3

[a]Yields determined via ^{1}H NMR spectroscopy and internal standard 1,3-dinitrobenzene. [b]Values determined as an average of two trials.

Since the selenyl radical has been shown to have a broad absorption band ranging from UV light to red light, a variety of LEDs were tested to determine their compatibility in the desired model reaction. We observed that in a range between 300 nm and 650 nm, the desired amination can be performed with yields around 80% and full conversion after 3 h (Table S14, Entry 11–14). To test wavelengths below 300 nm the setup had to be changed from the size-fitted, round photo vials to a square-cut quartz cuvette due to the intrinsic UV absorption of silica glass. Here, the reaction with green light (528 nm, Table S14, Entry 15) did not result in a full conversion of starting material **2a** and in a slightly lower yield of 76%. After 3 h, the reactions with both 285 nm and 275 nm resulted in significantly lower yields of under 20% (Table S14, Entry 16 and 17). Because those LEDs were used with a different power and could therefore not be directly compared to the other conditions, the reaction with 275 nm was repeated with a reaction time of 28 h (Table S14, Entry 18), after which a comparable yield and conversion to the reaction with green light (Table S14, Entry 15) could be achieved. Along the same lines LEDs with higher wavelength were screened in the photo setup. The reaction with light at wavelengths between 600 nm and 700 nm (Table S14, Entry 8 and 10) did not result in a full conversion after 3 h, albeit with a good yield/conversion ratio. Therefore, the reaction with 660 nm was run for 24 h, after which full conversion and a yield of 85% could be observed

(Table S14, Entry 9). Since we had already demonstrated that the reaction works with central wavelengths between 275 nm and 568 nm, and because some LEDs have an intrinsic UV-light emission, we started including longpass filters in our setup, which only allow light of wavelengths above a certain cutoff to pass into the reaction mixture (Figure S3).

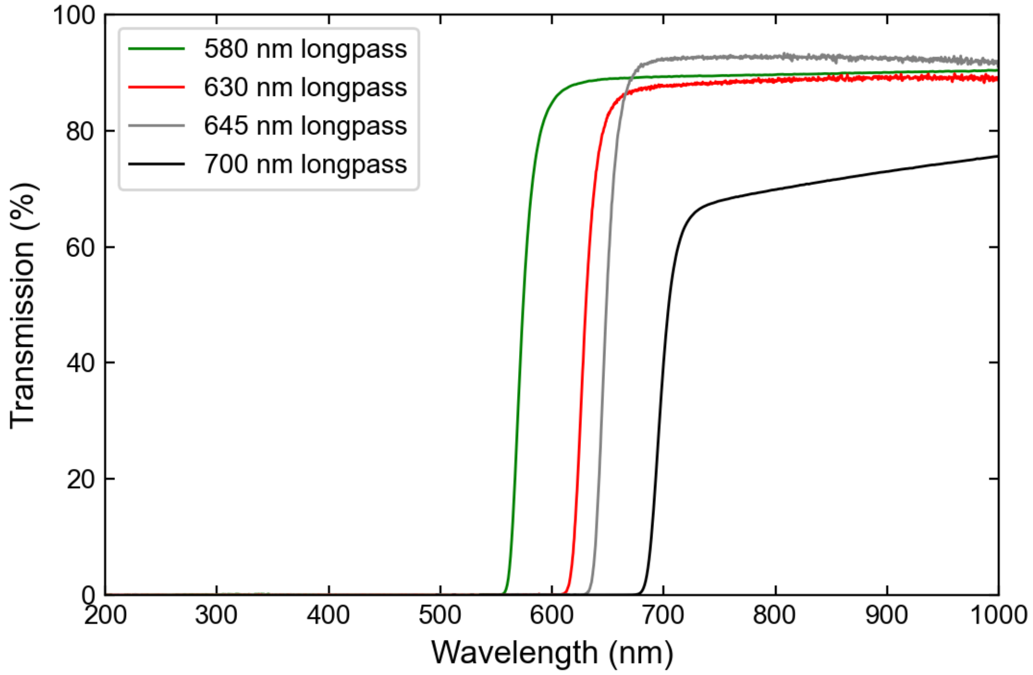

Figure S3: Transmission spectra of filters used in the experiments.

Using a filter with a high transmission only above 580 nm and the LED that emits 660 nm, we were able to demonstrate that the reaction can be successfully performed with light irradiation between 580 nm and 710 nm, resulting in a yield of 84% at full conversion after 24 h (Table S14, Entry 5). If the same LED was used with a filter that only allows light above 630 nm or even 645 nm to pass into the reaction, both conversion of starting material **2a** and the formation of products **4aa** (N^2) and **4aa** (N^1) are significantly inhibited (Table S14, Entry 6 and 7). Thus, it can be concluded that the reaction works with light between 580 nm and 710 nm, but not with light between 635 nm and 710 nm which connotes that the limit of the desired excitation lies between 580 nm and 635 nm. This further substantiates the hypothesis that it is indeed the selenyl radical that is excited in the photoreaction, as the absorption band of the radical ends around 630 nm. To round off the control experiments, the reaction was performed with LEDs with wavelengths 730 nm and 850 nm and filters that exclude UV-light. In both cases, 24 h reaction time (Table S14, Entry 2 and 4) resulted in no significant product formation compared to the reactions without any light (Table S14, Entry 19 and 20). Stirring the reaction for 9 d at this wavelength only slightly increased the yield of the reaction to up to 18% (Table S14, Entry 1 and 3), also indicating the excitation limit of the photoactive species lies below those wavelengths. To exclude the possibility that natural light from the surroundings during preparation or workup of the reactions had an influence on the performance, a control experiment was performed without LED and without coating of the photo vial, thus adjourning the reaction mixture only to room light for 3 h (Table S14, Entry 21). Product formation could be observed in traces, comparable to the reaction without any light (Table S14, Entry 19), showing that direct irradiation with a LED is necessary for a successful reaction.

4 Control experiments

4.1 C–Se bond interchange

During our investigations, we were looking for a way to determine, whether C–Se σ-bonds that are heteronuclear but can be considered as homopolar due to a nearly identical electronegativity participate in the desired homolysis reaction under thermal conditions. For this purpose, Cyclohex-2-en-1-yl(phenyl)selane (**2a**) was deuterated selectively only at the α position (**2a-1*d*,** see page 65). We anticipated that a homolysis, and therefore formation of a radical pair, would result in a second product (**2a-3*d***) due to a radical addition elimination mechanism (Table S15, for theoretical calculations see Chapter 10.4 Radical addition elimination mechanism).

Table S15: Scrambling of Allyl Selenides

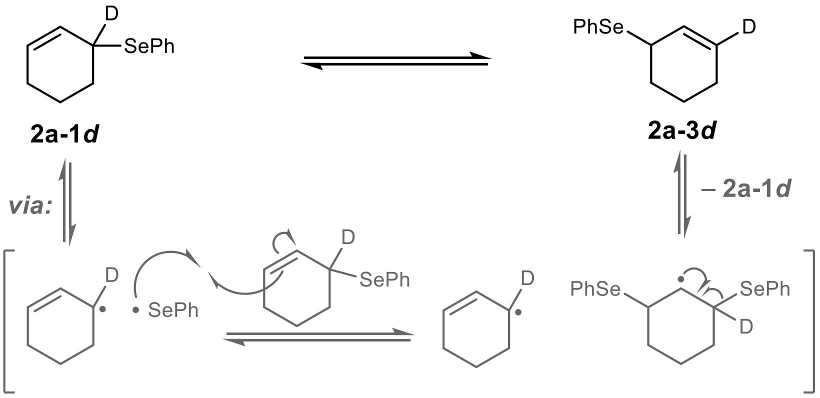

Entry	T	t	Solvent	TFA (50 mol%)	Deuteration of **2a-1*d*** [%]	Ratio **2a-1*d*** (%)	Ratio **2a-3*d*** (%)	C–Se σ-bond interchange ratio η (%)
1	19 °C	3 h	–	–	70%[a]	100[a]	0[a]	–
2	19 °C	3 h	HFIP	no	70%[a]	93[a]	7[a]	14[a]
3	19 °C	3 h	HFIP	no	70%[b]	95[b]	5[b]	10[b]
4	19 °C	3 h	HFIP	yes	70%[a]	50[a]	50[a]	100[a]
5	19 °C	3 h	HFIP	yes	70%[b]	53[b]	47[b]	94[b]
6	19 °C	3 h	^{i}PrOH	no	70%[a]	100[a]	0[a]	0[a]
7	19 °C	3 h	^{i}PrOH	no	70%[b]	100[b]	0[b]	0[b]
8	19 °C	3 h	^{i}PrOH	yes	70%[a]	100[a]	0[a]	0[a]
9	19 °C	3 h	^{i}PrOH	yes	70%[b]	100[b]	0[b]	0[b]
10	55 °C	3 h	HFIP	no	70%[a]	53[a]	47[a]	94[a]
11	19 °C	< 5 min	HFIP	yes	80%[b]	52[b]	48[b]	96[b]
12	19 °C	< 5 min[c]	HFIP	yes	80%[b]	49[b]	39[b]	78[b]

[a]Determined via ^{1}H NMR spectroscopy through comparison of olefinic and allylic proton signals. [b]Determined via ^{2}H NMR spectroscopy through comparison of olefinic and allylic proton signals. [c]Addition of **2a-1*d*** and TFA to 5.0 equiv. TEMPO dissolved in HFIP, new signal in ^{2}H Spectrum (TEMPO-allyl adduct as confirmed via HRMS); Entry 1-12: $c_{\mathbf{2a\text{-}1}d}$ = 0.5 M.

Thus, we defined a value η that should represent the C–Se σ-bond interchange ratio. η is given as the ratio of (**2a-3*d***) observed in the reaction relative to the highest possible ratio of (**2a-3*d***), 0.5, that would be reached in the optimal case if the interchange was complete and a statistic distribution of the two isomers was accomplished. Therefore, η varies between 0% (no C–Se σ-bond interchange) and 100% (full C–Se σ-bond interchange).

$$\eta = \frac{\mathbf{2a\text{-}3}\boldsymbol{d}(observed)}{\mathbf{2a\text{-}3}\boldsymbol{d}\ (optimal)} \qquad \textbf{\textit{(1)}}$$

Most experimental values were evaluated both in ^{1}H and ^{2}H NMR spectroscopy experiments (e.g. Table S15, Entry 2 and 3) to ensure better reliability of the given ratios. As anticipated, we could observe a σ-bond interchange of the C–Se bond of **2a-1*d*** in the presence of the solvent HFIP (Table S15, Entry 2 and 3). Addition of acid (50 mol% TFA) significantly increased the interchange ratio to a nearly complete interchange (Table S15, Entry 4 and 5). On the other hand, ^{i}PrOH as the solvent neither with nor without acid additive resulted in any observable scrambling at all (Table S15, Entry 6–9). This is in accordance with our theory that the hydrogen-bond network in HFIP (that can be strengthened through acid addition) has a stabilising effect not only on ionic, but also neutral open-shell selenium species, and that only this effect makes the interchange feasible at 19 °C. Along the same lines, HFIP without acid results in a similar interchange ratio (Table S15, Entry 10) as the experiment with acid additive if the reaction is heated to 55 °C. Even if HFIP and TFA are only added to **2a-1*d*** and then immediately removed again, consequently allowing for a reaction time of less than 5 min (Table S15, Entry 11), the interchange is already finished. 5.0 equiv. of radical scavenger TEMPO was dissolved in HFIP and **2a-1*d*** and TFA were added to the mixture at which point the solvent was immediately removed, ensuring a reaction time of less than 5 min (Table S15, Entry 12). Indeed, an inhibition of the interchange ratio as well as a new allylic signal in the ^{2}H spectrum was observed and the allyl-TEMPO adduct was confirmed via HRMS ((ESI-MS) [$C_{15}H_{26}DNO$] ([M+H]$^{+}$), obs.: 239.2236, calcd.: 239.2228), further substantiating the hypothesis that the observed scrambling does proceed through radical intermediates and via homolytic C–Se bond cleavage.

4.2 Se–Se bond interchange

Similarly to the C–Se σ-bond interchange, we were also looking for a way to investigate and quantify the Se–Se σ-bond interchange of diselenides (Scheme S1).

Table S16: Scrambling of Diselenides

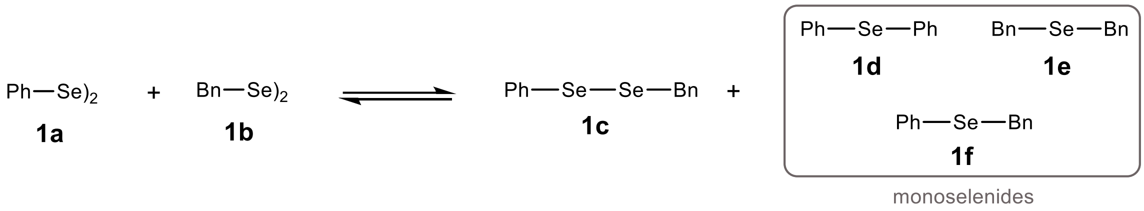

Entry	T	Time (Rxn/NMR)	Solvent (Rxn/ NMR)	TFA (50 mol%)	Se–Se σ-bond interchange ratio θ (%)[a]	Monoselenide formation (%)[a]
1	19 °C	3 h/2 h	HFIP/$CDCl_3$	no	80	8
2	19 °C	3 h/2.25 h	HFIP/$CDCl_3$	yes	78	9
3	19 °C	3 h/2.25 h	^{i}PrOH/$CDCl_3$	no	81	2
4	19 °C	3 h/2.25 h	^{i}PrOH/$CDCl_3$	yes	91	2
5	55 °C	3 h/2 h	HFIP/$CDCl_3$	no	38	31
6	rt	–/15 min	–/$CDCl_3$	no	85	2
7[b]	rt	–/30 min	–/$CDCl_3$	no	54	0
8[c]	rt	–/45 min	–/$CDCl_3$	no	58	4

[a]Calculated from ratios determined via ^{1}H NMR spectroscopy through comparison of benzylic proton signals. [b]Addition of diselenides to 5.0 equiv. TEMPO dissolved in deuterated HFIP. [c]Addition of diselenides to 2.0 equiv. Galvinoxyl free radical dissolved in deuterated HFIP, Entry 1-5: $c_{1a} = c_{1b}$ = 0.25 M (in reaction) $c_{1a} = c_{1b}$ = 0.25 M–0.5 M (in NMR tube), Entry 6-8: $c_{1a} = c_{1b}$ = 0.04 M.

For this purpose, we introduced a value for the Se–Se σ-bond interchange ratio, θ. The statistic and optimal ratio **1a**, **1b** and **1c** would be 1:1:2 when the interchange is complete. Since Bn–Se)$_2$

(**1b**) can not only participate in a Se–Se σ-bond interchange, but also a C–Se σ-bond interchange, the formation of Bn–Se–X monoselenides (**1e** and **1f**) was observed (as judged by benzylic signals in ^{1}H NMR spectrum, for reference spectra see Narayanaperumal *et al.*[1]). Since Ph–Se)$_2$ (**1a**) only weakly participates in the C–Se σ-bond interchange on a notably longer timescale of several days due to the instability of the thereby formed phenyl radical, diphenyl monoselenide **1d** is not observed in any scrambling test reaction (hour time scale).

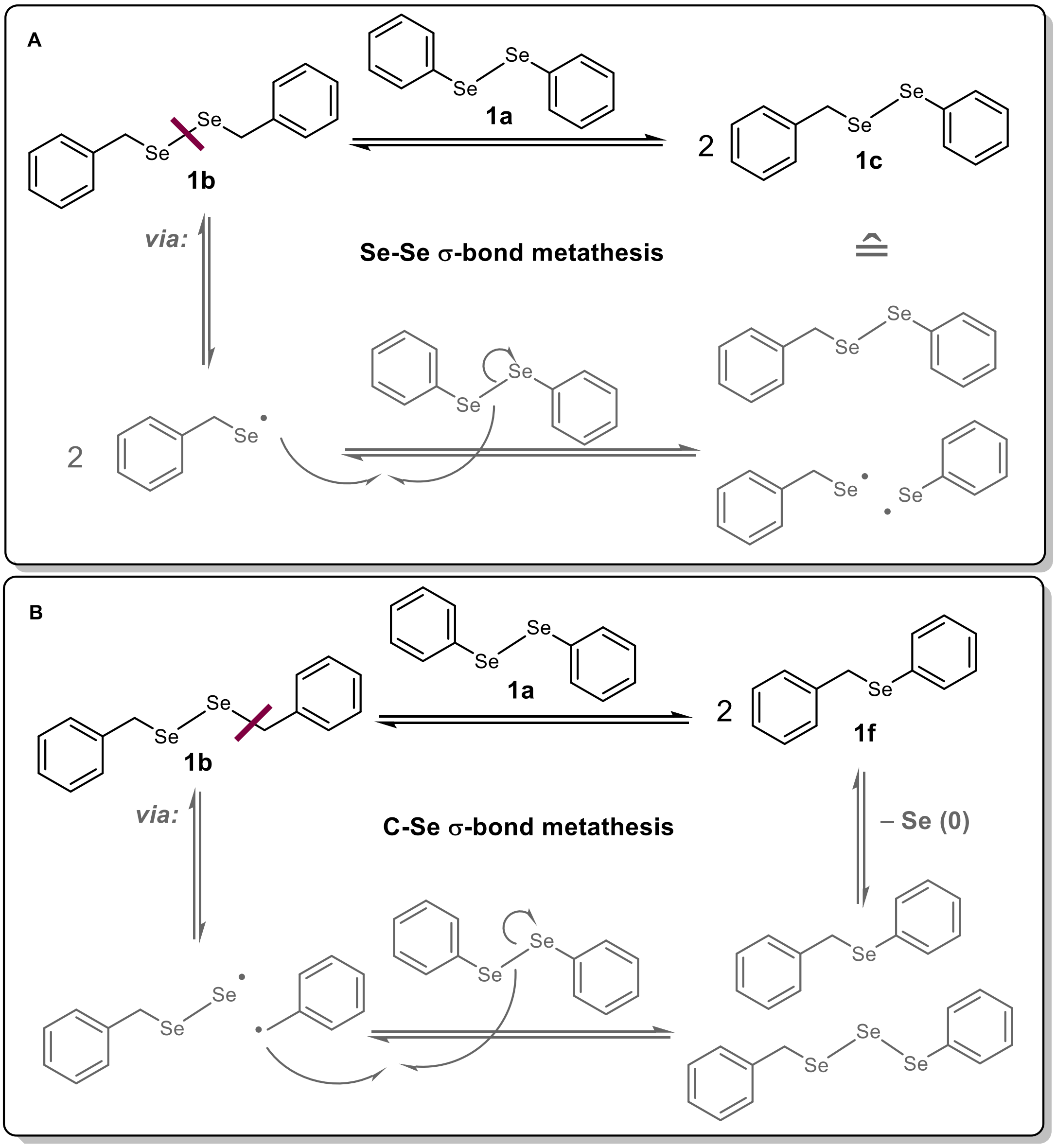

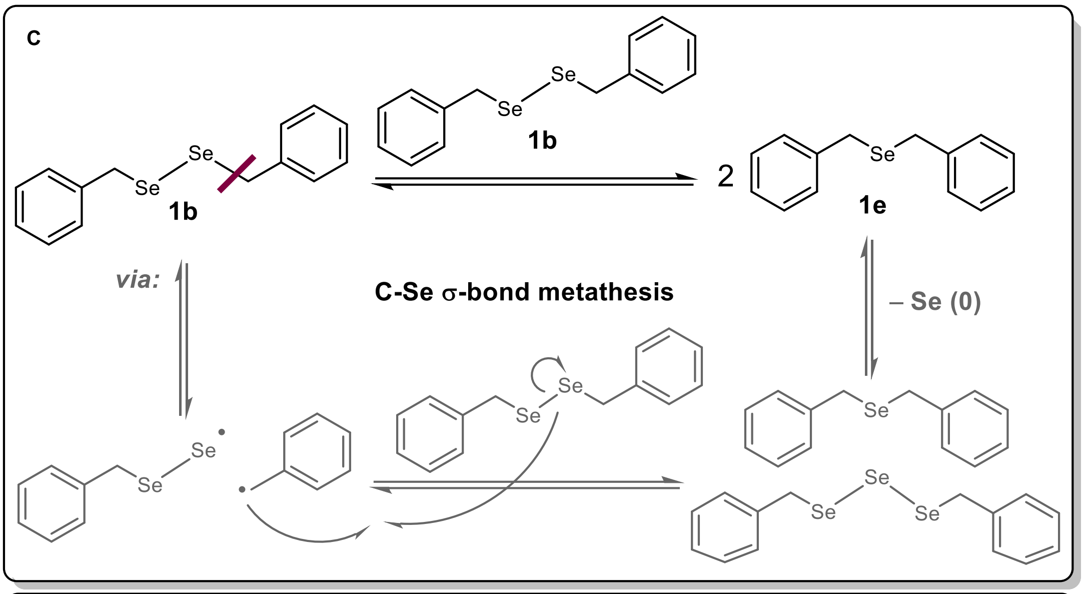

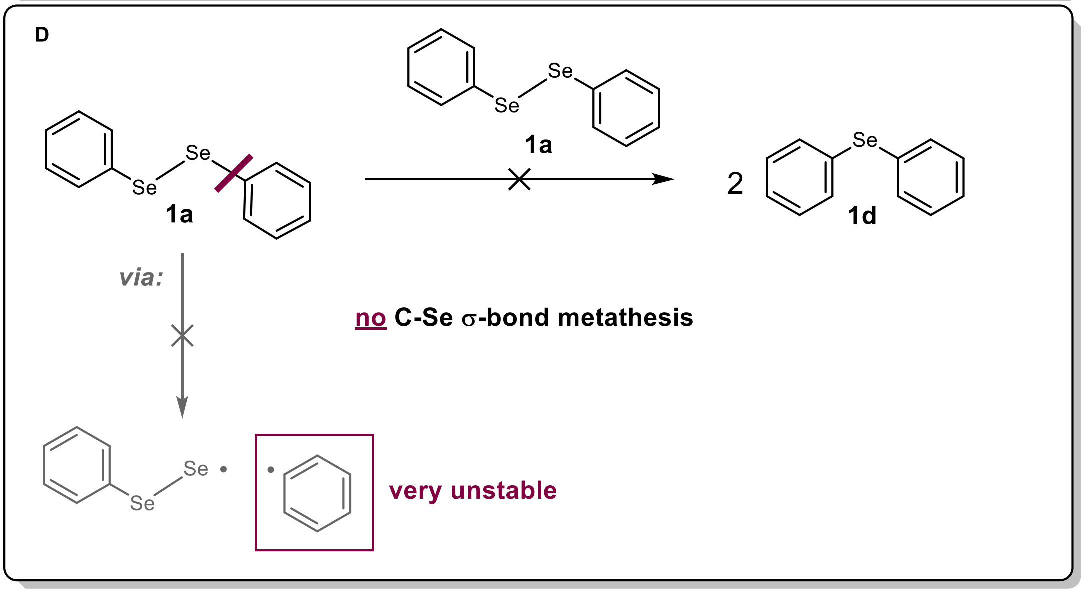

Scheme S1: Depiction of the mechanisms for **A.** the Se–Se σ-bond interchange that results in the formation of the mixed diselenide **1c**; **B.** & **C.** the C–Se σ-bond interchange of **1b** that results in the monoselenide formation of **1e** and **1f**; and **D.** the highly unfavoured C–Se σ-bond cleavage of **1a** that demonstrates why the formation of monoselenide **1d** was not observed in the reaction mixtures.

This additional C–Se σ-bond interchange must be taken into consideration for the Se–Se σ-bond interchange ratio, as this process consumes molecules of **1a** and **1b** that can therefore no longer participate in the scrambling and will no longer be detected in the product mixture. Therefore, the amount of monoselenide formation needs to be abstracted from the theoretically optimal ratio of **1c**. Since the formation of one molecule **1e** formally consumes one molecule of **1b** while the formation of **1f** formally consumes 0.5 molecules of **1b**, this stoichiometry needs to also be taken into consideration. As the ratio in the product mixture can only be measured through the benzylic signals in the ^{1}H NMR spectra, the ratio of **1a**/**1b** at the start of the experiment can reasonably be assumed as 1:1, and since only **1e** can be formed from **1b** without consuming **1a**, the estimation n(**1a**) = n(**1b**) + n(**1e**) was used for the calculation. Thus, the

Se–Se σ-bond interchange ratio θ was calculated as follows (n(end) = true amount at end of reaction, n(int) = amount determined through ratio of integrals in the ^{1}H NMR spectra):

$$\theta = \frac{\mathbf{1c}\ (observed)}{\mathbf{1c}\ (optimal)} = \frac{\frac{n_{1c}(end)}{\frac{n_{1a}(end) + n_{1b}(end)}{2}}}{2 - \frac{n_{1e}(end)}{2} - n_{1f}(end)} = \frac{\frac{n_{1c}(int)}{\frac{n_{1b}(int) + n_{1e}(int) + n_{1b}(int)}{2}}}{2 - \frac{n_{1e}(int)}{2} - n_{1f}(int)} \qquad \textbf{\textit{(2)}}$$

Here, both HFIP and ^{i}PrOH, irrespective of an acid additive, resulted in an interchange ratio of about 80% or higher (Table S16, Entry 1–4). Since previous investigations by the Xu working group[2] suggested that either heat or light was necessary for the Se–Se σ-bond interchange, we therefore conducted a control experiment where we put the two diselenides directly into the NMR tube, added $CDCl_3$ in the dark and immediately measured the ^{1}H NMR spectra (Table S16, Entry 6). Still, an interchange ratio of 85% was observed. This suggests that the Se–Se σ-bond interchange proceeds both readily and rapidly in various solvents at room temperature without light. Analogous to the C–Se σ-bond interchange experiments, addition of radical scavengers such as TEMPO (Table S16, Entry 7) or Galvinoxyl free radical (Table S16, Entry 8) to the solvent HFIP before addition of the diselenides significantly inhibited the interchange reaction. Interestingly, monoselenide formation that can be attributed to a competing C–Se σ-bond interchange reaction was mainly observed in the reactions with HFIP (Table S16, Entry 1, 2 and 5), perfectly in accordance with the results from the allylic selenides (Table S15).

Still, we were wondering why Xu and coworker only observed Se–Se σ-bond interchange while irradiating or heating the sample, whereas we could not stop the interchange except for the addition of radical traps. Therefore, we conducted further test experiments on the system that the Xu working group determined to be one of the least reactive, dibenzyl diselenide (**1b**) and 11,11'-Diselanediylbis(undecan-1-ol), $(HOC11Se)_2$ (**1g**) (Scheme S2).

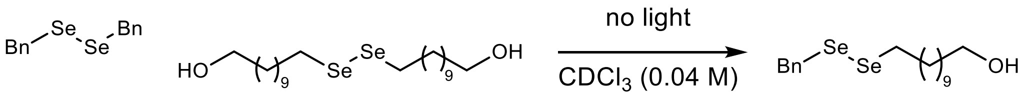

1b **1g** **1h**

Scheme S2: Se–Se σ-bond interchange of dibenzyldiselenide (**1b**) and $(HOC11Se)_2$ (**1g**) to **1h**.

Nevertheless, if deuterated chloroform was added in the dark directly to the NMR tube with **1b** and **1g**, and the ^{1}H NMR spectrum was immediately measured, a similar interchange ratio to the previous experiments (see Table S16, Entry 6; θ = 85%) of θ = 84% was observed. In deuterated HFIP, the value is lower at 48%, which can be attributed to the high dibenzyl monoselenide formation of 23% due to the C–Se σ-bond interchange that is promoted in HFIP. We attribute this discrepancy to previously published results to the fact that the scrambling reaction is very sensitive towards changes in concentration, especially at the start of the reaction, a fact that was already observed by the Xu working group. They showed that raising the concentration from 5 mM to 20 mM resulted in an increased conversion from about 10% to about 25% after only 10 min. Since our reactions were either carried out in a photo vial with a concentration of 500 mM, or in an NMR tube with estimated concentrations >35 mM, we reckon that the effect observed by Xu and coworkers would be amplified, thus resulting in high

interchange ratios in the dark without external heat. It can therefore be assumed that the Se–Se σ-bond interchange scrambling readily takes place in various solvents under our standard reaction conditions and concentrations.

4.3 Role of the atmosphere

During our investigations, we began to ponder the role of the ambient air atmosphere. Control experiments (Table S17) showed that an atmosphere of pure oxygen (Entry 2) could slightly increase the total yield achieved in the model reaction in the same timeframe, while a pure nitrogen atmosphere decelerated both the rate of conversion of starting material and the rate of product formation (Entry 3 and 4).

Table S17: Role of the atmosphere

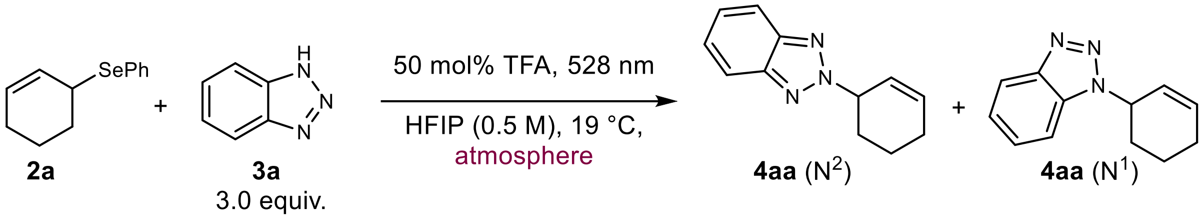

Entry	Atmosphere	Light?	Additives	Time	Conversion (%)[a]	Total Yield (%)[a]
1	air	yes	–	3 h	100	83
2	O_2	yes	–	3 h	100	91
3	N_2	yes	–	1 h	19	15
4	N_2	yes	–	3 h	16	16
5	N_2	yes	–	8 h	35	24
6	N_2	yes	–	24 h	72	40
7	N_2	yes	–	48 h	100	86
8	N_2	no	H_2O_2 (22 mol%)	3 h	51	46

Reactions with N_2 atmosphere were degassed via Freeze-Pump-Thaw method with three cycles and performed with a cannula and N_2 balloon. The O_2 reaction was performed with a cannula and balloon with pure O_2. Ambient air reactions were performed with two cannulas to ensure air exchange with the surrounding atmosphere. [a]Yields determined via ^{1}H NMR spectroscopy and internal standard 1,3-dinitrobenzene.

Letting the reaction run for longer durations of time with the same atmosphere (Entry 5 and 6) constantly increased the yield, even allowing a very good yield of 86% at full conversion (Entry 7), a comparable result to the reaction with ambient air. Therefore, it can be concluded that oxygen is not necessary for the reaction itself but can significantly increase the speed of the reaction. Considering that we hypothesised that the role of oxygen in the reaction is a single electron oxidation of phenyl selenol to from diphenyl diselenide, this may formally result in the formation of $O_2^{\bullet-}$. This species can accept three more electrons to move through the forms of H_2O_2 and $^{\bullet}OH$ to the stable species H_2O. Taking into account that the main incentive of this work consists of a stimulated single electron oxidation of carbon radicals through an excited selenium radical, the hypothesis that any intermediate species in the total four electron reduction of O_2 to two H_2O molecules may imitate this process and initiate a SET does not appear to be unfounded. To further substantiate this hypothesis, we added 0.25 equiv. of H_2O_2 in a degassed photo vial in the dark. Indeed, without any irradiation, about 50% conversion of starting material and yield of the desired product can be observed, which is in accordance with the fact that H_2O_2 can receive two electrons to be reduced to water, thereby resulting in two

reacted molecules of substrate per molecule of peroxide. This is in accordance with our calculated quantum yields for the amination reactions both with oxygen and oxygen-free (argon) conditions. We calculated a quantum efficiency higher than 1 for the reaction with air atmosphere and lower than 1 for the reactions with argon atmosphere (see chapter 5.2 Quantum efficiency). Indeed, the reaction with air is concisely four times faster than the one with argon atmosphere as evidenced by the fact that running the reaction 12 h under argon atmosphere compared to the 3 h under air atmosphere resulted in a yield of 18% and 19% respectively (see Scheme S4). It can thus be concluded that the desired titular SDET follows our predictions and calculations when the reaction is performed under oxygen-free conditions, and oxygen has an accelerating effect on the reaction through facilitating three more SET per successfully performed SDET.

4.4 Application of the generated carbocations in other electrophilic reactions

Since we propose that our SDET mechanism results in the formation of the carbo cation that – at this point of the mechanism – should be completely indistinguishable from any structurally identical cation formed from a heteropolar, activated bond, we concluded that our photo reaction protocol should be extendable to other common electrophilic reactions. To test this theory, we applied two electron rich aromatic systems, anisole (**3x**) and 1,3,5-trimethoxybenzene (**3w**), to our photo reaction with an allylic and a tertiary selenide (**2a** and **2d**). Indeed, we were able to observe the Friedel-Crafts products in all four cases with NMR-yields between 18% and 61%, thus further underlining the broad applicability and versatility of our novel activation method.

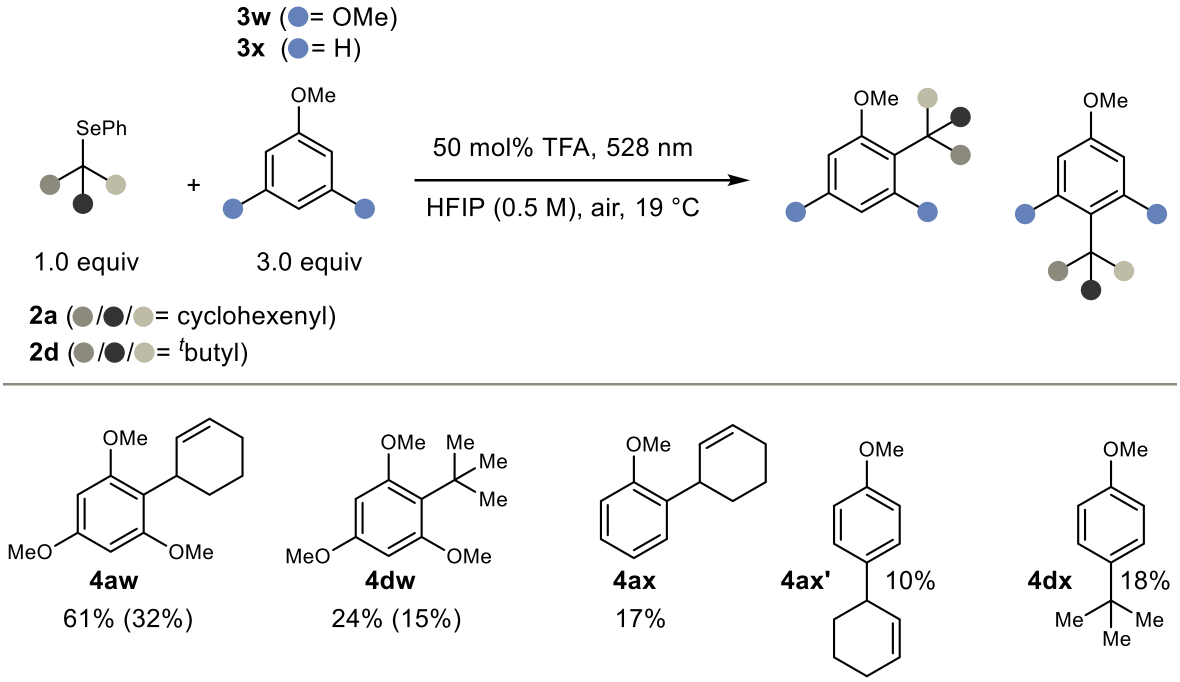

Scheme S3: Overview of the Friedel-Crafts alkylation of anisole (**3x**) and 1,3,5-trimethoxybenzene (**3w**) with selenides **2a** and **2d** in the SDET photo reaction protocol. NMR-yield (isolated yield), NMR-yield determined via ^{1}HNMR and internal standard 1,3-dinitrobenzene.

Due to purification issues, the unpolar products **4ax**, **4ax’** and **4dx** could not be isolated from the complex reaction mixtures, a fact that was already observed by other groups in some of these examples.[3] The NMR yield was determined according to characteristic proton signals in the ^{1}H NMR spectrum in agreement with spectra found in literature,[3,4] and the presence of all products was confirmed via HRMS.

5 Determination of the required number of photons and the quantum efficiency

5.1 Photon dependency

To further solidify and quantify the required numbers of photons for our proposed SDET mechanism, we began investigations regarding the dependence on the photon flux and the quantum efficiency. By limiting the photon flux in a controllable but non-invasive manner through the inclusion of neutral density filters and making an appropriate set of measurements, the dependency of the conversion and yield to the photon flux can be determined. For this purpose, we designed a set-up that would ensure that only the light emitted from the LED was consistently directed through the sample in a reproducible manner. This was achieved through encasing both the underside of the LED as well as the complete flask in black material that 1) prevents light beyond the one contributed by the LED from coming into contact with the sample, and 2) absorbs excessive light that is not absorbed by the sample and is reflected, thus altering the photon flux. Due to the size of the filters, the photo vial reactor described in section 2 was exchanged for a reactor fitted for two 100 mL round-bottom flasks and a corresponding LED. To have a reference point and check the reproducibility of the reactions, the experiments were compared to those conducted in the photo vials.

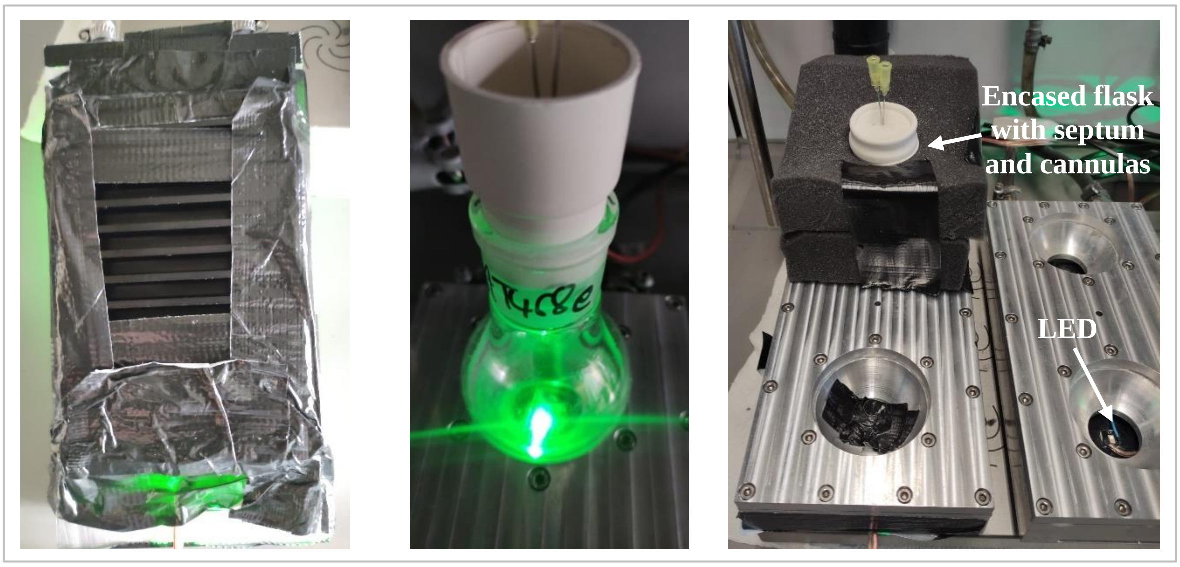

Figure S4: Purpose-built photo reactor with encasing of bottom side/LED (left), designed for irradiation of 100 mL flasks (middle) and black encasing of the flasks (right).

Since we postulate that in the case of the C–Se bonds, the initially proceeding formation of the radical pair is a thermal process and hence occurs without any light stimulus (see chapter 4.1 C–Se bond interchange, page 15), we hypothesize that only the SDET process requires one single photon. Thus, the dependence on the photon flux should be linear. We performed a set of experiments with neutral density filters of different optical density (OD) ranging from 0.6 to 2.0 in the standard amination reaction with benzotriazole, the substrate we already used in the optimisation experiments (see chapter 3.2 Atypical S_N1 aminations). Since we observed a strong effect of oxygen on the reactions, the relevant experiments were repeated under the exclusion of oxygen. For this purpose, HFIP was saturated with argon gas for several hours and

the starting materials **2a** and **3a** were evacuated and put under argon atmosphere twice before the argon-saturated HFIP and TFA were added. Additionally, the experiments were conducted with argon balloons. Since argon is heavier than air atmosphere, we anticipated that we could thus eliminate the influence of oxygen. Previous reactions had already shown that the duration of time that was needed until similar yields and conversions were achieved differ severely in the reactions with or without oxygen (see Table S17). Thus, we concluded that 30 h was an appropriate duration for our experiments with argon atmosphere.

Table S18: Photon flux dependency of the conversion and yields of the atypical amination reactions with argon atmosphere.

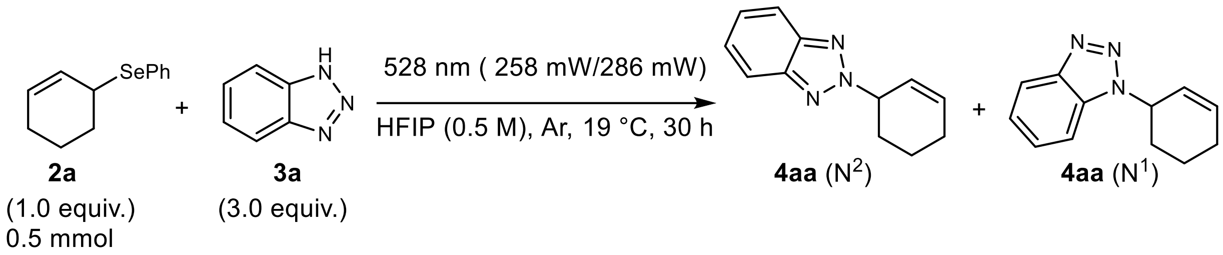

Entry	OD Filter	Light Flux[b]	Conversion (%)[a]	Yield **4ab** (N^2) (%)[a]	Yield **4ab** (N^1) (%)[a]	Total Yield (%)[a]
1	0.7	20%	100	54	33	87
2	0.8 (0.5+0.3)	16%	74	36	27	63
3	0.9 (0.5+0.4)	13%	60	32	22	54
4	1.0	10%	38	18	15	33
5	No light	–	5	1	1	2
6	Room light	?	73	38	27	65

[a]Yields determined via ^1H NMR spectroscopy and internal standard 1,3-dinitrobenzene. [b]Experiments were performed with a LED custom made by the electrical workshop of the University of Regensburg and a maximum output between 258 mW and 286 mW.

As expected, we observed only traces of products **4aa** (N^2 and N^1) in the reaction without light (Table S18, Entry 5), thus confirming our test experiments from the photovial set-up (see Table S14, Entries 19–21) and further underlying the pivotal role of light. A full conversion and total yield of 87% in the experiment with an OD 0.7 filter (Table S18, Entry 1) showed that the best conditions from the photovial could be accurately reproduced (see Table S14, Entry 14), and that 20% of the photon flux emitted from the LED is sufficient for a complete reaction progression. This is in accordance with our observation that at any given time the PhSe radical is only present in such small concentrations it cannot by observed in standard stationary UV/Vis spectroscopy and could only be detected in a transient absorption spectroscopy set-up (see chapter 7.4 Transient absorption of diphenyl diselenide in HFIP). Filters between OD 0.8 and 1.0 resulted in a steadily decreasing conversion and yield (Entries 2–4). Plotting the obtained conversions and yields against the applied photon flux respectively shows an unambiguous linear dependency for all four values with adequate coefficients of determination ($R^2 \geq 0.95$), thus further solidifying the proposed SDET mechanism and a corresponding one-photon process in the case of the C–Se bonds.

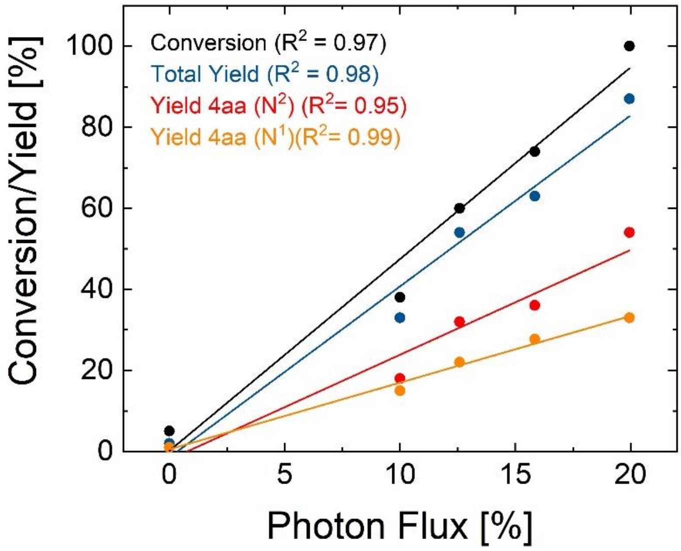

Figure S5: Graphic representation of the linear dependency yields and conversion of the amination against the photon flux with the corresponding coefficients of determination.

We observed that even minuscule amounts of light with filters between 0.8 and 1.0, and thus a LED power below 50 mW was producing results distinctively higher than those without light. This is in a range where strong room light may be sufficient for triggering some reactivity, especially over the duration of 30 h. Indeed, repeating the reaction without any encasing (Table S18, Entry 6) resulted in yields and conversions comparable to those with an OD 0.8 filter. The power of room light can be estimated to be in the same order of magnitude of power as after the OD filter ≥ 0.8, and we have already demonstrated that our desired reaction pathway can be accessed through excitation wavelengths starting at UV light and going up to 630 nm. It can therefore be concluded that the reaction is very sensitive towards light, and that room light can trigger some reactivity on longer reaction time scales, while not having any significant influence on shorter time scales (e.g. 3 h, Table S14, Entry 21), thus not compromising the reaction outcome during the set-up or work-up of the reactions.

Since we anticipated that the Se–Se bond ampholysis works in the same way as the C–Se bond lysis, we theorise that in the lactonisation also only one single photon is required for the SDET process. Thus, the dependence on the photon flux should be linear. We performed a set of experiments with neutral density filters of different optical density (OD) ranging from 0.3 to 1.0 in the lactonisation via ambipolar Se-species with MSA as additive and with 6a, the substrate we already used in the optimisation experiments (see chapter 3.1 SDET-induced ampholysis of PhSe–SePh). A control experiment with no light irradiation was performed to have a comparison to the amination. The experiments were conducted with two cannulas to ensure air exchange with the surrounding atmosphere. We concluded that 6 h was an appropriate duration for our experiments under ambient air atmosphere due to conversion of approximately 80% without any filter, so that a linear or a quadratic photon flux dependency should be clearly distinguishable.

Table S19: Photon flux dependency of the conversion and yields of the SDET-induced ampholysis of PhSe–SePh (**1a**)

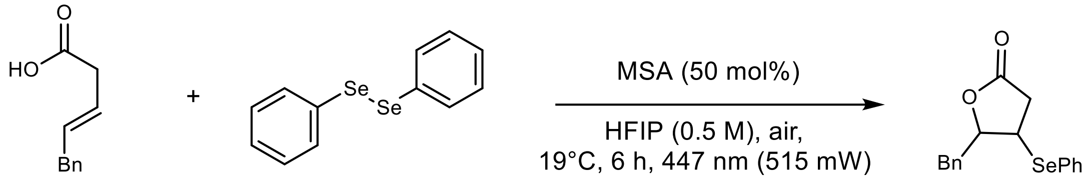

6a
(1.0 equiv.)
0.5 mmol

1a
(1.0 equiv.)

7a

Entry	OD-Filter	Light Flux[b]	Conversion (%)[a]	Yield **7** (%)[a]
1[c]	–	100%	100	93
2	–	100%	71	59
3	0.3	50%	45	36
4	0.7	20%	28	23
5	1.0	10%	19	13
6	No light	–	4	3

[a]Yields determined via ^{1}H NMR spectroscopy and internal standard 1,3-dinitrobenzene [b]Experiments were performed with a LED custom made by the electrical workshop of the University of Regensburg and a maximum output with 515 mW. [c]reaction run for 24 h without black encasing.

As expected, we observed only traces of product with no light (Table S19, Entry 6) thus confirming our test experiments from the photovial set-up (see Table S2, Entry 5) and further underlying the pivotal role of light. A full conversion and total yield of 93% in the experiment without any filter (Table S19, Entry 1) showed that the results from the photovial could be accurately reproduced (see Table S2, Entry 3). Filters between OD 0.0 (without any filter), 0.3, 0.7 and 1.0 resulted in a steadily decreasing conversion and yield (Table S19, Entries 3–5). Plotting the obtained conversions and yields against the applied photon flux respectively shows an unambiguous linear dependency with adequate coefficients of determination ($R^2 \geq 0.97$), thus further solidifying the proposed SDET mechanism and a corresponding one-photon process also in the case of the Se–Se bonds.

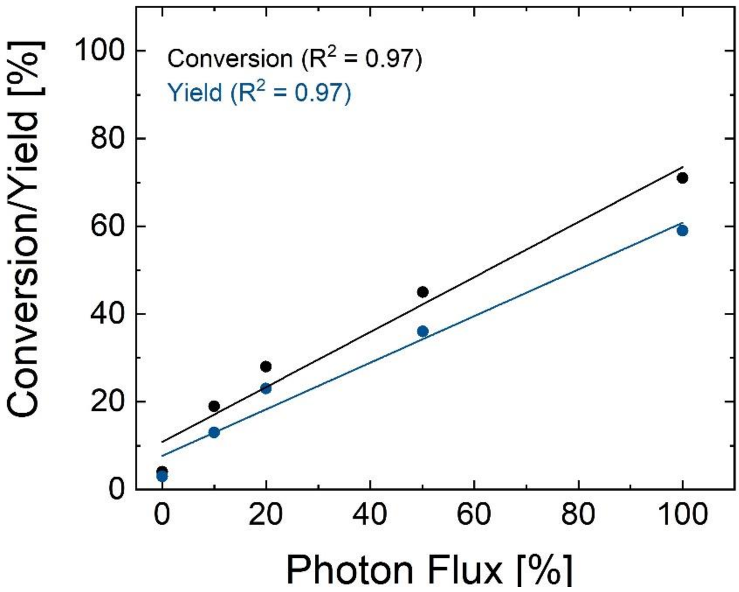

Figure S6: Graphic representation of the linear dependency of yield and conversion of the lactonisation against the photon flux with the corresponding coefficients of determination.

5.2 Quantum efficiency

Due to the very low concentration of the phenylselenyl radical, below the detection limit of stationary UV/Vis spectroscopy, the quantum yield of these reactions cannot be determined with high accuracy. Hence, we perform several approximations, in order to get a rough estimate and no conclusion is drawn beyond the order of magnitude. For calculation of the quantum yield, the number of converted molecules and the number of absorbed photons over the reaction time are required. While the former is obtainable from the amount of starting material, the total yield and the stoichiometry of the reaction, the latter requires knowledge of the number of photons emitted by the LED and the absorption at the utilised wavelength at all times. Therefore, two approximations are made:

On the one hand, the concentration of the phenylselenyl radical is assumed to be constant during the entire reaction time. In the case of the lactonisation, diphenyl diselenide acts as a catalyst, meaning its concentration, and therefore the concentration of the radical, should roughly remain constant. As diphenyl diselenide is also a byproduct of the atypical amination reaction, the concentration of the phenylselenyl radical should not decrease there either.

On the other hand, we need to estimate the absorbance of the radical at the excitation wavelength. For the atypical aminations, this is achieved by examining the spectrum of a representative reaction mixture using *tert*-butyl phenyl selane as a starting material and comparing it with the spectrum of the phenylselenyl radical (Figure S17). The nucleophile is left out to avoid strong scattering.

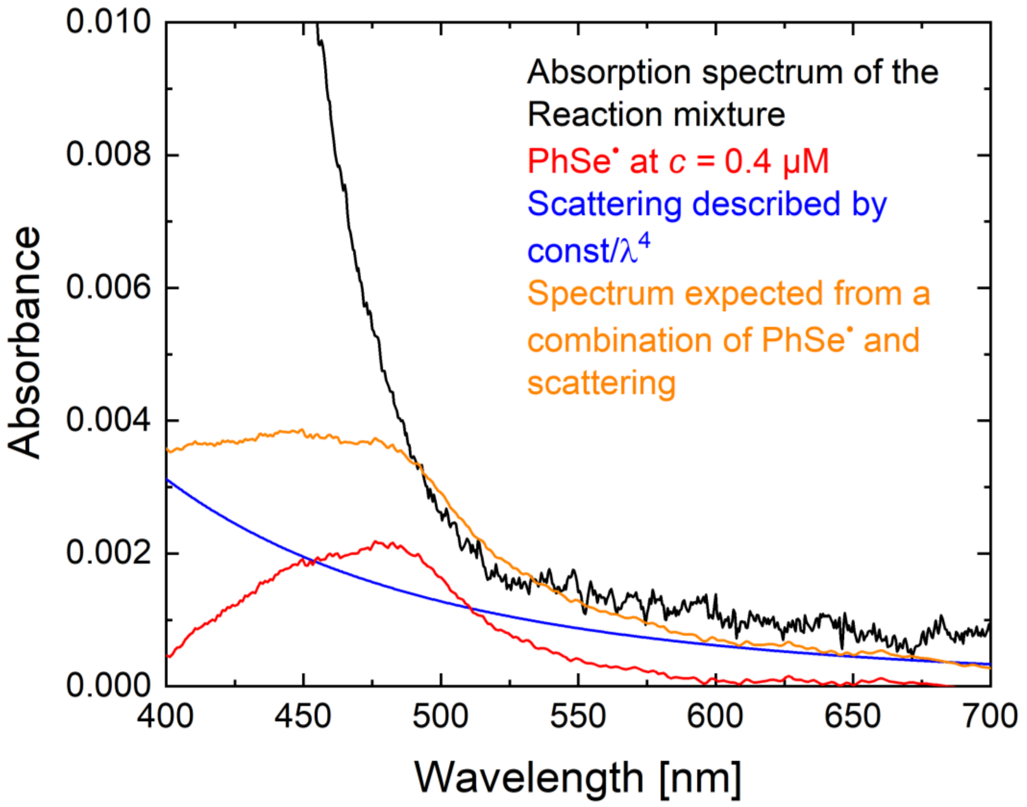

Figure S7: The absorbance of the reaction mixture (black curve) in the spectral range from 500 to 700 nm is assumed to only comprise the spectrum of the phenylselenyl radical (red curve) and a scattering contribution (blue curve). The radical concentration is chosen in such a way that their sum (orange curve) describes the black curve reasonably well within the noise

level of the spectrometer. Below 500 nm diphenyl diselenide, which is generated from the free phenylselenyl radicals, absorbs.

One can see that the spectral features of the radical would not be detected if its absorbance at 490 nm was lower than 0.002. This corresponds to a concentration of about 0.4 µM, according to the extinction coefficient obtained by Ito.[5] In this case, the absorption of the radical at 528 nm would be about 0.001, which means 0.2 % of photons are absorbed by this species. With a yield of 19% of 0.5 mmol after a reaction time of 3 h, using a LED with an emission power of 258 mW and a central wavelength of 528 nm, the quantum yield should be about 4 under air. Hence, the reaction seems to be rather efficient, and a rough estimate of its quantum yield would be 4. While this value is higher than one, it was already established earlier that one photon can transform up to 4 molecules via hydrogen peroxide generation under air (see 4.3 Role of the atmosphere), which is in line with the calculated value. If the same reaction is performed under oxygen-free conditions, one would expect the quantum yield to drop because no subsequent reactions involving hydrogen peroxide can occur. Indeed, with an argon atmosphere, one obtains a yield of 18 % after 12 h, which corresponds to a quantum yield of 0.9 given the same parameters for the absorbance and the LED.

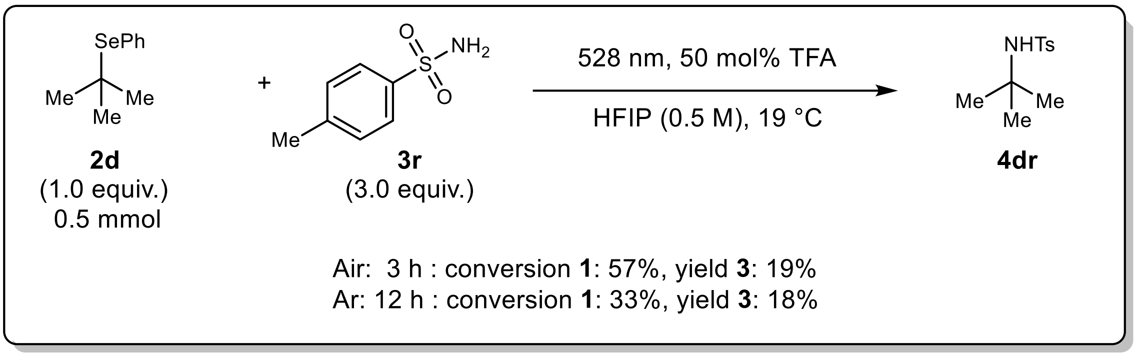

Scheme S4: Results of the atypical SDET amination of *tert*-butyl phenyl selane **2a** with **3r** under air and argon atmosphere, respectively. Yields and conversion determined via ^{1}H NMR spectroscopy and internal standard 1,3-dinitrobenzene.

In the case of the lactonisation, the absorbance cannot be estimated in the same way due to strong scattering of the reaction mixture. However, if the same value is assumed as for the atypical aminations, one gets a quantum yield of about 0.7 for Entry 1 in Table S2.

6 Stationary absorption spectra in the UV/Vis

6.1 Spectra of 1a, 2a, 3a, and 4aa

All these molecules are stable compounds under standard conditions, their absorption spectra in HFIP are shown in Figure S8.

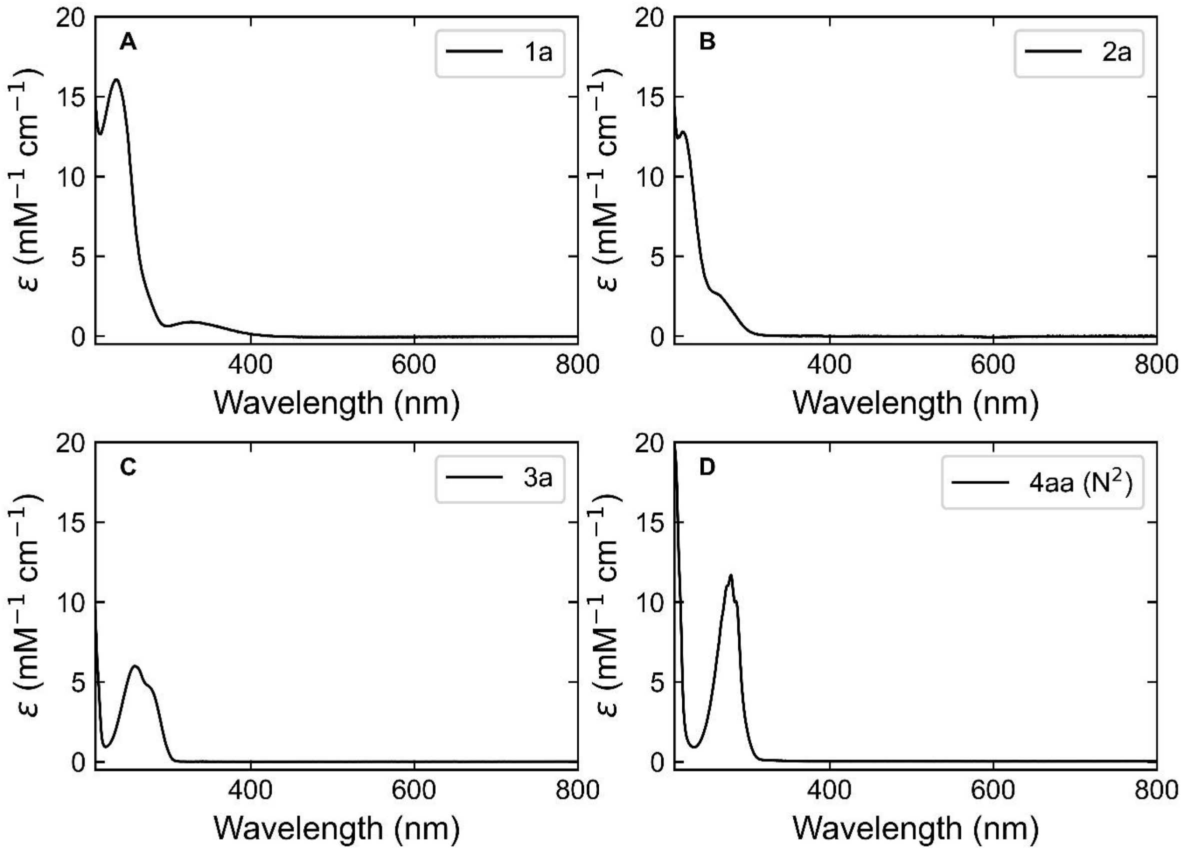

Figure S8: Absorption spectra in the UV-Vis spectral range of diphenyl diselenide (**1a**) (0.085 mM) (A), allylselane **2a** (0.09 mM) (B), nucleophile **3a** (0.08 mM) (C), and product **4aa** (N^2) (0.07 mM) (D) in HFIP.

6.2 Absorption spectra in the UV/Vis spectral range of TFA and HFIP

The absorption spectra of TFA (c = 3.23 mM) in HFIP and HFIP itself were referenced against HFIP and air, respectively (Figure S9).

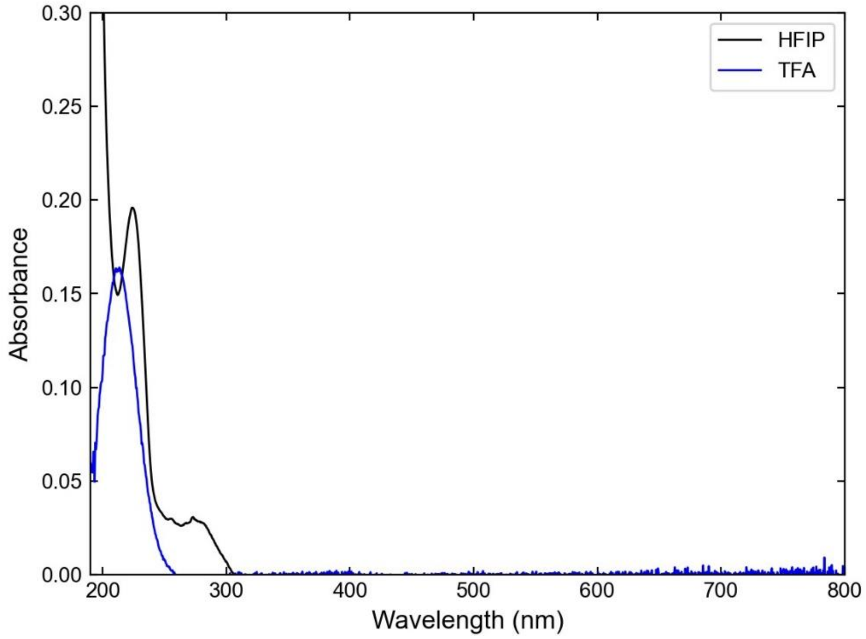

Figure S9: Absorption spectra in the UV-Vis of pure HFIP (black) and TFA (3.23 mM, blue) in HFIP. Since the concentration of pure HFIP is 9.49 M, the extinction coefficient of the peak at 225 nm is only about 0.02 M^{-1} cm^{-1}.

6.3 Absorption spectrum of a mixture of 2a and 3a

The absorption spectrum, especially in the spectral range above 500 nm, of a mixture of **2a** and **3a** in HFIP (Figure S10, c_{2a} = 11.1 mM, c_{3a} = 22.2 mM, imitating the reaction conditions as close as possible due to the spectroscopic limitations) does not show additional electronic transitions arising from a potential complex between these species. Thus, such a hypothetical complex as a key component in the reaction mechanism can be ruled out.

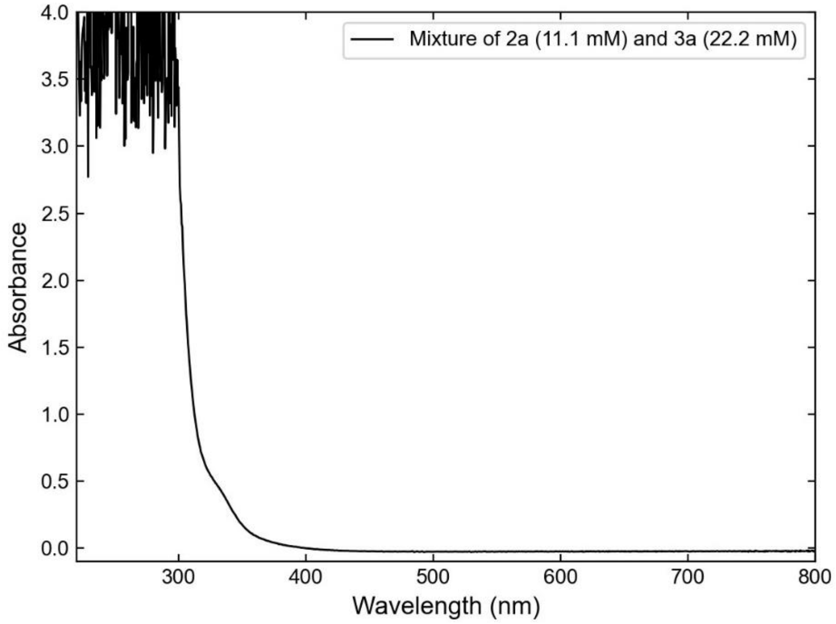

Figure S10: Absorption spectrum in the UV-Vis of a mixture of **2a** (11.1 mM) and **3a** (22.2 mM).

6.4 Generation of $PhSe^-$

Following literature procedure[6] the phenylselenyl anion was generated via reduction of $(PhSe)_2$ (c = 23.6 µM) with a saturated $NaBH_4$ solution in acetonitrile (5 mg of $NaBH_4$ and 10 mL acetonitrile were mixed and sonicated for several minutes (Ultrasonic Cleaner USC-T, VWR)). Remaining $NaBH_4$ particles were filtered off with a syringe filter (PTFE membrane, 0.2 µm, Braun).

The recorded absorption spectrum of $PhSe^-$ (Figure S11) is in good agreement to the data previously published by Ahrika *et al.*[7]

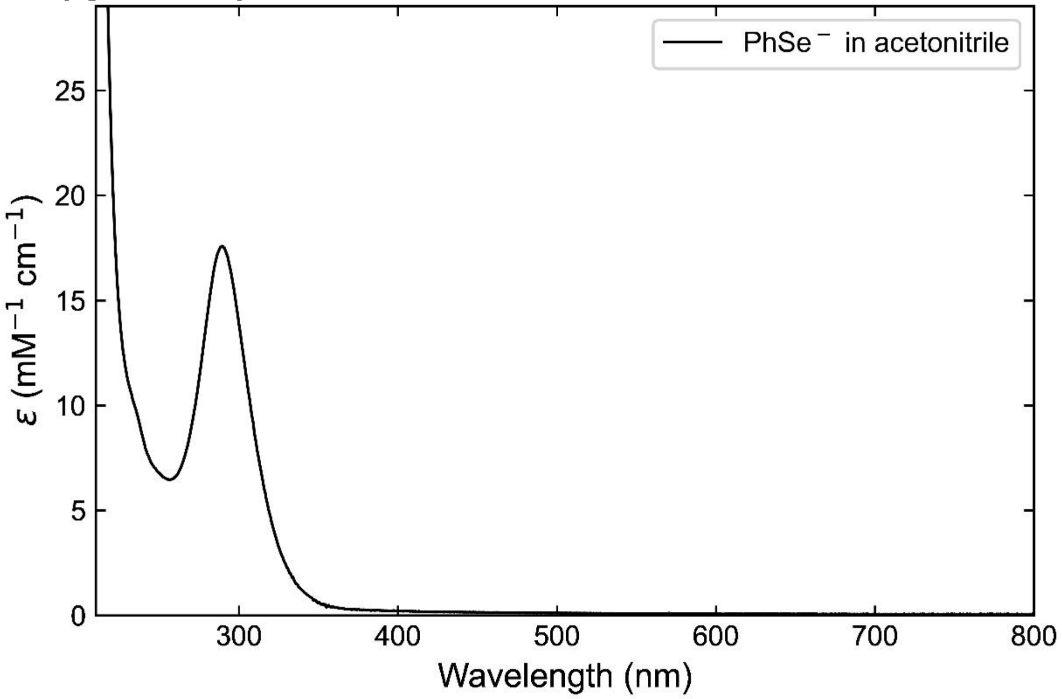

Figure S11: Absorption spectrum in the UV-Vis of $PhSe^-$ generated from $(PhSe)_2$ in saturated $NaBH_4$ solution in acetonitrile.

6.5 Generation of $PhSe^+$

$PhSe^+$ was generated from *N*-(phenylseleno)phthalimide with an excess of methylsulfonic acid in HFIP. In this reaction phthalimide is formed as a by-product. To access the pure cation absorption spectrum, the pure absorption spectra of methylsulfonic acid and phthalimide were subtracted from the spectrum of the reaction mixture (Figure S12).

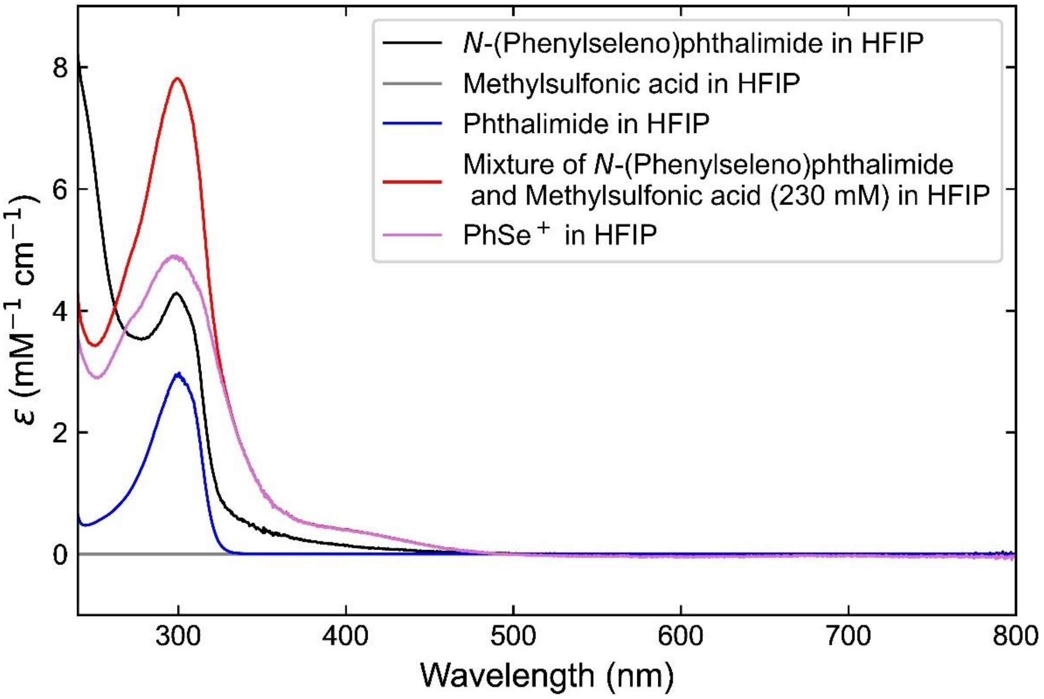

Figure S12: Absorption spectra in the UV-Vis range of *N*-(Phenylseleno)phthalimide (73 µM, black curve), methylsulfonic acid (0.23 M, grey curve) and phthalimide (1.36 mM, blue curve) in HFIP. The red curve shows a mixture of *N*-(Phenylseleno)phthalimide (73 µM) and methylsulfonic acid (0.23 M). The spectrum of the pure $PhSe^+$ (pink curve) was calculated subtracting the pure absorption spectra of methylsulfonic acid and phthalimide from the spectrum of the mixture.

6.6 Generation of 3a-C^+

3a-C$^+$ was generated by solving cyclohexenyl chloride in MeCN (c = 48 mM) and saturating the solution with $AgPF_6$ by sonicating (Ultrasonic Cleaner USC-T, VWR). The remaining $AgPF_6$ as well as the precipitating AgCl were removed from the solution via centrifugation (Centrifuge 5415, Eppendorf). Afterwards, the supernatant was diluted by a factor of 20 of which the absorption spectrum in the UV/Vis was recorded showing a new absorption band at 260 nm due to formation of the cyclohexenium cation (Figure S13).

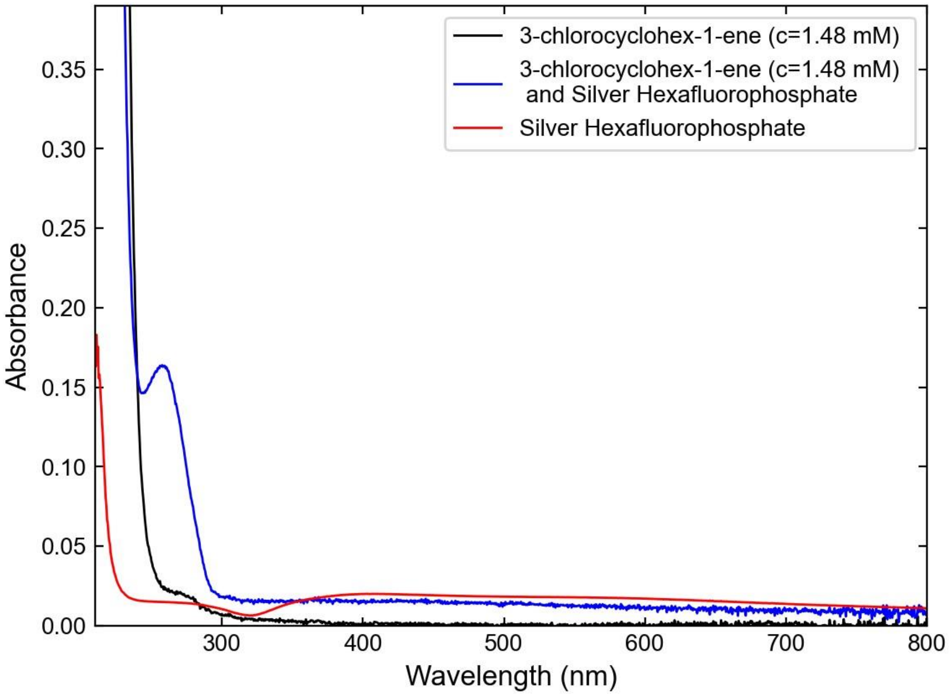

Figure S13: Absorption spectra in the UV-Vis of 3-chlorocyclohex-ene (1.48 mM, black curve) and a mixture of 3-chlorocyclohex-ene and Silver Hexafluorophosphate (blue curve) in MeCN. The red curve shows the rather broad spectrum of a saturated solution of Silver Hexafluorophosphate, scaled by the dilution factor of 20. The strong offset of the blue curve may be partially explained with scattering due to residual solid AgCl.

7 Transient absorption spectroscopy

7.1 Sub-ps pump/supercontinuum-probe spectroscopy

The transient absorption (TA) in the UV/Vis on the fs to ns time range was recorded with an in-house built setup as described previously.[8] The $(PhSe)_2$ sample in HFIP located in a quartz cell with 1 mm pathlength was excited into the $S_1 \leftarrow S_0$ absorption band, *i.e.*, λ_{exc} = 342 nm, with a pulse energy of *ca.* 300 nJ. The sample was co-linearly probed with a white-light supercontinuum (WLSC) that was generated by focussing the fundamental 800 nm pulse of *ca.* 1 mJ into a 5 mm thick CaF_2 disc. The spot sizes for the pump and probe pulses at the sample position were *ca.* 80 µm and *ca.* 40 µm, respectively. The sample was stirred by a magnetic stirring bar rotating in the plane of the quartz cell driven by a rotating magnet. The time axis was chosen to be linear from −1 ps up to 2.0 ps in 20 fs steps and logarithmic afterwards, until the end of the delay stage of 6.5 ns. 200 transient absorption spectra were recorded at each delay position of a single scan. Prior to averaging each single spectrum was corrected for the dark current on the camera and as well as for the fluctuations in the probe spectrum by referencing via a second camera. Averaging of 12 independent scans resulted in the final spectra. For recording the pure population dynamics of all excited states, the polarisation between pump and probe pulses was set to magic angle (54.73°) via a λ/2 plate in the pump beam path. The averaged pre-t_0 laser scatter signal was subtracted from the data and the *ca.* 2 ps chirp of the WLSC is corrected for prior to data analysis using the coherent artefact as an indicator for time zero at each wavelength. No smoothing or filtering procedures were applied to the data.

7.2 Transient absorption spectroscopy in the ns to ms time range

The setup for transient absorption measurements on ns-to ms-timescales was already described in detail.[9] In short, the third harmonic of a Nd:YAG laser (Surelite II, Continuum, 10 mJ, *ca.* 10 ns) at 355 nm was used for excitation (pump pulse) and a pulsed 150 W Xe-flash lamp (Applied Photophysics) served as the probe pulse in an orthogonal pump probe configuration. For detection a spectrograph (200is, Bruker) and a streak camera (C7700, Hamamatsu Photonics) were used, thus allowing the recording of spectrally and temporally resolved TA data simultaneously. By mechanically blocking the pump or the probe with shutters, four different datasets were recorded in one measurement cycle: First $I_{\mathrm{Data},i}$ with both pump and probe light, second $I_{\mathrm{Dark},i}$ with both pump and probe light blocked for dark current correction of $I_{\mathrm{Data},i}$, third $I_{\mathrm{Ref},i}$ with only probe light, and fourth $I'_{\mathrm{Dark},i}$ with both pump and probe light blocked for dark current correction of $I_{\mathrm{Ref},i}$. N such sequences were recorded (typically N = 100), and the corresponding data sets were averaged prior to calculateing the transient absorbance via equation 3.

$$\Delta A = -\log\left(\frac{\sum_i^N (I_{\mathrm{Data},i} - I_{\mathrm{Dark},i})}{\sum_i^N (I_{\mathrm{Ref},i} - I'_{\mathrm{Dark},i})}\right) \qquad \textbf{\textit{(3)}}$$

The samples were prepared in 10 mm × 10 mm quartz cuvettes (Starna or Hellma) and stirred during the measurements. The probe light passed the sample along the window through which the excitation pulse entered the sample in order to probe the sample volume with the highest transient concentration. To check the integrity of the sample due to the illumination stress its steady-state absorption spectrum was recorded before and after each TA recording (UV-1800, Shimadzu or Cary60, Agilent).

$$||\boldsymbol{\Delta A} - \mathbf{FB}||^2 = \mathrm{Min} \qquad \textbf{\textit{(4)}}$$

The recorded time-resolved absorption data are given in matrix $\boldsymbol{\Delta A}$ and the analytical functions for describing the temporal changes in the data are given in matrix $\mathbf{F}$, which in the exponential ansatz are exponential decays (convoluted with the instrument response, typically a Gaussian function). Then matrix $\mathbf{B}$ contains the to be determined spectra. For further reduction of χ^2 a nonlinear least squares algorithm is used to optimise the nonlinear parameters such as the rate constants in matrix $\mathbf{F}$. In case of the exponential ansatz, so-called decay associated difference spectra (DADS in matrix B) and their associated optimised rate constants are obtained by such global fits, which represent the unique result. Accordingly, no model for the kinetics involved in the transient processes is required. The number of exponentials used may be determined by the SVD-based rank analysis.[10] A model that relates the actual species kinetics to the elementary function may be applied afterwards resulting in species associated (difference) spectra (SA(D)S), depending on whether the ground state contribution is added or not. The appropriateness of the model can be decided on the shape of the SA(D)S in terms of identity with well-known spectra or following physical laws. As this step does not change χ^2, this procedure has the advantage that all interpretation is performed with the same quality of fit. In case the transient absorption data were contaminated by strong excitation light scattering these data points were not considered in χ^2 during the global fit by a patching method.[9,11,12]

7.3 Generation of the cyclohexenyl radical

The hydrogen-abstraction capabilities of the excited triplet state of benzophenone were utilised to generate the cyclohexenyl radical.[13] After solving benzophenone in cyclohexene (c = 1.3 mM), transient absorption spectroscopy could be used to trace potential spectral features of the cyclohexenyl radical (Figure S14). Aside from the signal associated with benzophenone,[14] only a very small signal could be observed with a single absorption band peaking at ca. 310 nm, which may be in accordance with the edge of the literature spectrum of the cyclohexenyl radical.[15] The two DADS from a global two exponential fit show spectral features of the benzophenone ketyl radical and potentially those of the cyclohexenyl radicals $C_6H_9^•$ and $C_6H_{11}^•$ or some adduct of molecular oxygen either to the benzophenone ketyl radical or the cyclohexenyl radical.[15]

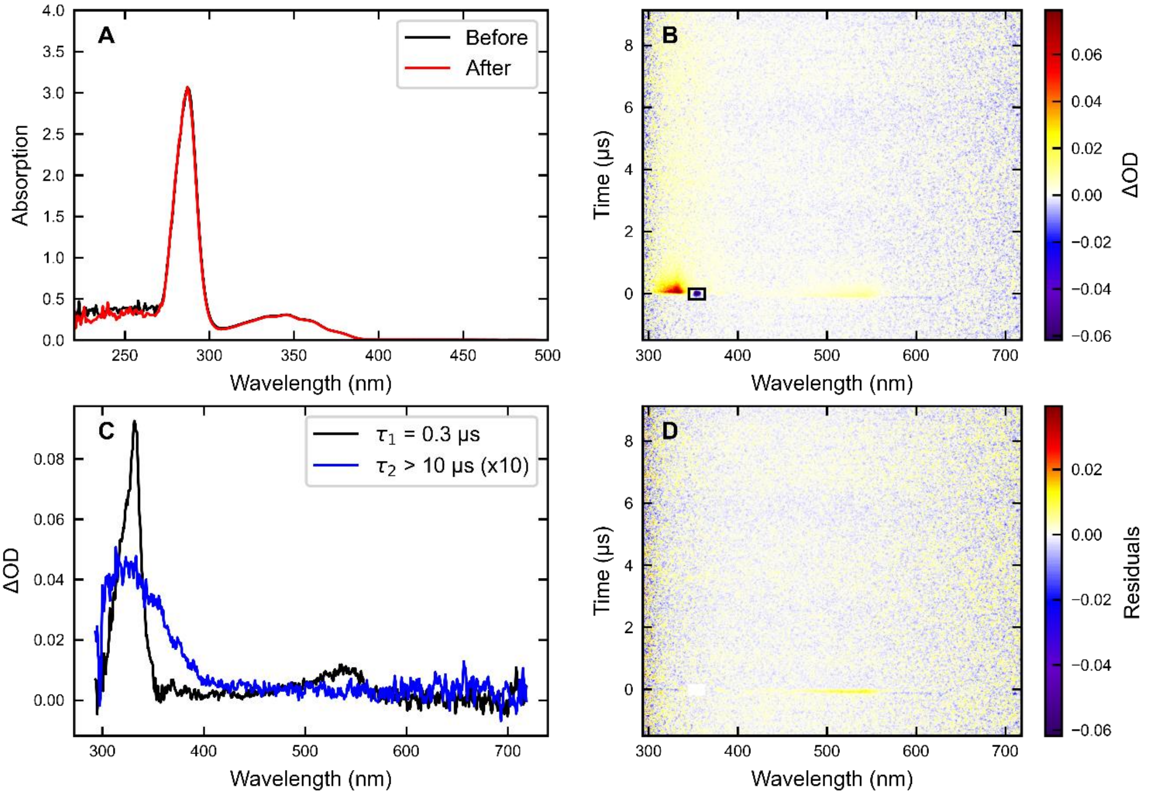

Figure S14: **A**) No changes in the stationary absorption spectrum can be observed after the laser measurement. **B**) Transient absorption data of benzophenone in cyclohexene (λ = 355 nm, E_{Laser} = 9 mJ, 100 laser pulses), displayed in a false colour representation, show a short-lived signal at about 330 and 540 nm and a very weak longer-lived signal below 400 nm. **C**) DADS obtained from a global lifetime analysis. The DADS with $\tau \approx$ 300 ns has spectral features of the ketyl radical from benzophenone, while the DADS with a lifetime longer than the recorded time window (blue DADS scaled by a factor of 10 for better visibility) shows spectral features of potentially the cyclohexenyl radicals $C_6H_9^•$ and $C_6H_{11}^•$ or some adduct of molecular oxygen either to the benzophenone ketyl radical or the cyclohexenyl radical.[15] For the fit the strong negative signal caused by scattering light of the laser was excluded by a patch (gray square in **B**). **D**) Residuals from the biexponential fit.

7.4 Transient absorption of diphenyl diselenide in HFIP

To model and understand the transient absorption data of diphenyl diselenide ($(PhSe)_2$), two systems were considered. In the first system, the radical species is quenched using (2,2,6,6-Tetramethylpiperidin-1-yl)oxyl (TEMPO) allowing the identification of the non-radical species individually (Figure S15, B). The second system is $(PhSe)_2$ alone (Figure S15, A).

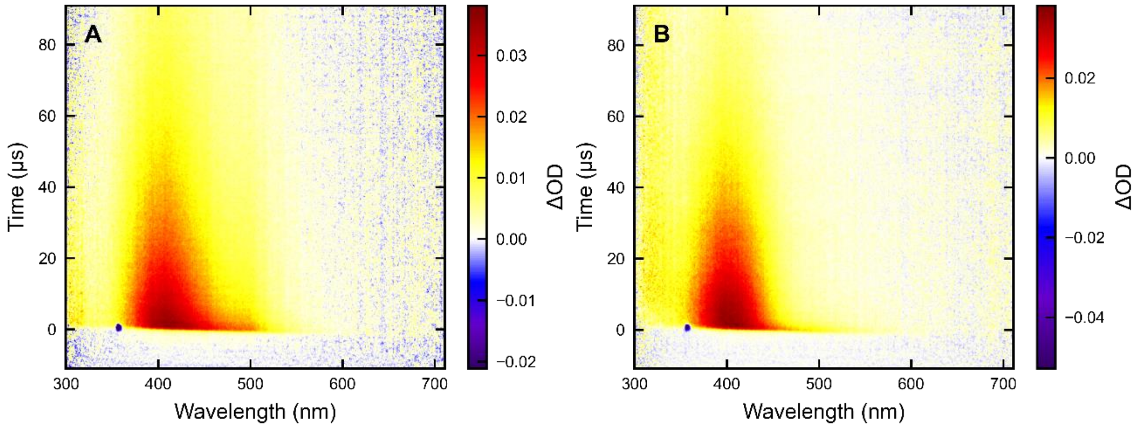

Figure S15: Transient absorption data (λ_{exc} = 355 nm, E_{exc} = 10 mJ, 200 excitation pulses) of **A**) $(PhSe)_2$ (c = 367 µM) in HFIP and **B**) $(PhSe)_2$ (c = 367 µM) and TEMPO (c = 7.2 mM) in HFIP.

The addition of TEMPO leads to a reduction of the decay time of the signal at 500 nm, indicating that the signal is probably caused by a radical species and the comparison to previous studies[16] corroborates that the signal is caused by $PhSe^{\bullet}$. On the other hand, the signal at λ_{max} = 400 nm is not drastically changed by the addition of TEMPO, suggesting that the signal is caused by a closed-shell molecule. The position of the signal does not fit to $PhSe^{-}$ (Figure S11) or PhSeH but is in reasonable agreement with a selenium species that exhibits a positively charged selenium moiety such as PhSeCl or PhSeBr.

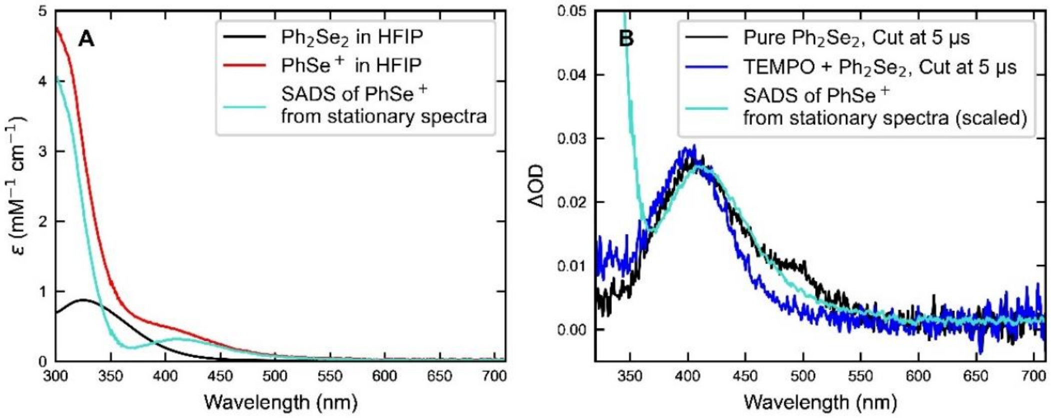

Figure S16: **A**) The absorption spectra of $(PhSe)_2$ (black) and $PhSe^+$ (red), together with the species associated difference spectra (SADS) expected for the formation of $PhSe^+$ from $(PhSe)_2$ (turquoise) as determined by the difference between the steady-state absorption spectra. **B**) The expected SADS for $PhSe^+$ (turquoise) is compared to cuts of the transient absorption data of $(PhSe)_2$ (black) and $(PhSe)_2$ and TEMPO (blue) at 5 µs.

The juxtaposition of the expected SADS of the cation, calculated from the steady-state absorption spectra (Figure S16, A, turquoise), with the transient data (Figure S16, B) shows a certain similarity, but also some deviations, which might originate from the energetically feasible formation of $(PhSe)_3^+$ in the presence of excess Ph_2Se_2.[17]

To extract the spectra of PhSe• from the data, the data obtained with TEMPO ($Data_{Tempo}$) is simply subtracted from the data obtained with pure $(PhSe)_2$ ($Data_{pure}$), as illustrated in Figure S17.

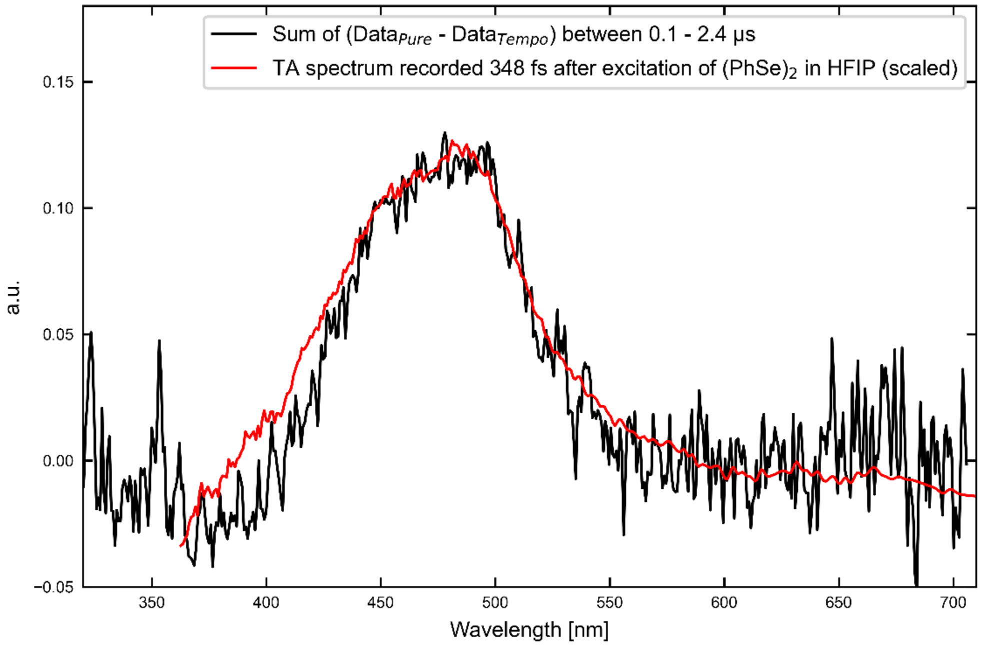

Figure S17: The black graph shows the difference between the µs-time resolved TA data of pure $(PhSe)_2$ and the data of $(PhSe)_2$ with TEMPO. To reduce the noise, the data were summed up between 0.1 µs up to 2.4 µs after excitation. In comparison, the red curve shows the scaled TA spectrum at t_{delay} = 348 fs of the ultrafast TA data on $(PhSe)_2$ in HFIP (red). At this point in time, a potential electron transfer between the radical fragments may not have occurred, yet.

7.5 Transient absorption of 2a in MeCN

The transient absorption of the monoselane **2a** following excitation at 266 nm was recorded in MeCN (Figure S18). **2a** is expected to photodissociate forming the phenylselenyl radical, which in a thermally relaxed state may eventually recombine to the photoproduct diphenyl diselenide only on diffusive encounter and, thus, prohibiting charge separation resulting in phenylselenium cation formation. Indeed, the spectral signatures assigned to the phenylselenium cation (see Figure 4) are absent, and only those assigned to the phenylselenyl radical are observed forming diphenyl diselenide.

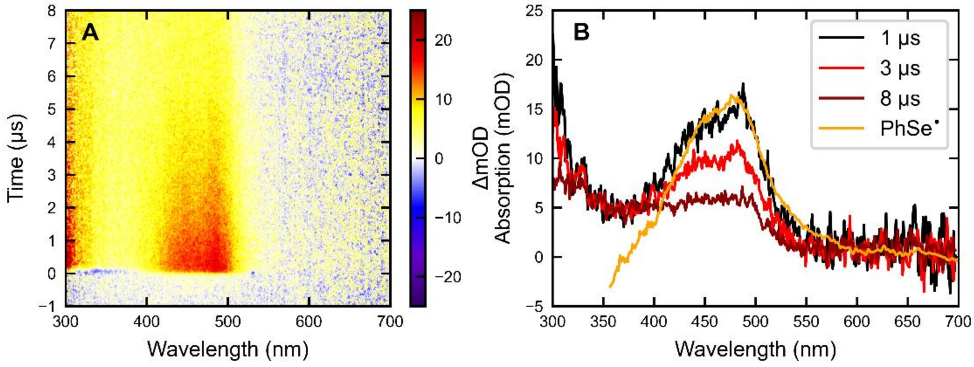

Figure S18: **A)** Transient absorption data ($\boldsymbol{\lambda_{exc} = 266\ nm}$, $\boldsymbol{E_{exc} = 10\ mJ}$, 200 excitation pulses) of compound **2a** in MeCN. **B)** Spectra taken 1, 3, and 8 µs after excitation compared to a scaled spectrum of the phenylselenyl radical (see Figure S17). To note, to avoid coexcitation

of the accumulating photoproduct diphenyl diselenide from diffusive encounters of the photogenerated phenylselenyl radicals, the sample was replaced between the excitation pulses via a flow-system (flow cell: 2 mm pathlength for excitation and 10 mm for probing; peristaltic pump (ecoline, ISMATEC)), which is incompatible with HFIP, so that MeCN was used.

7.6 Influence of the acid additive on $(PhSe)_2$

The addition of 107 mM methylsulfonic acid (MSA) to $(PhSe)_2$ causes a dark reaction over several hours, which was observed *via* UV-VIS spectroscopy (Figure S19). The product spectrum can be identified as $PhSe^+$. However, it is unlikely that it is merely the acid additive driving the synthetic reaction under illumination, as for the spectroscopic investigation of Figure S19, MSA was used in 738-fold excess and the formation of the $PhSe^+$ signal in the dark is significantly slower than the studied photoreaction.

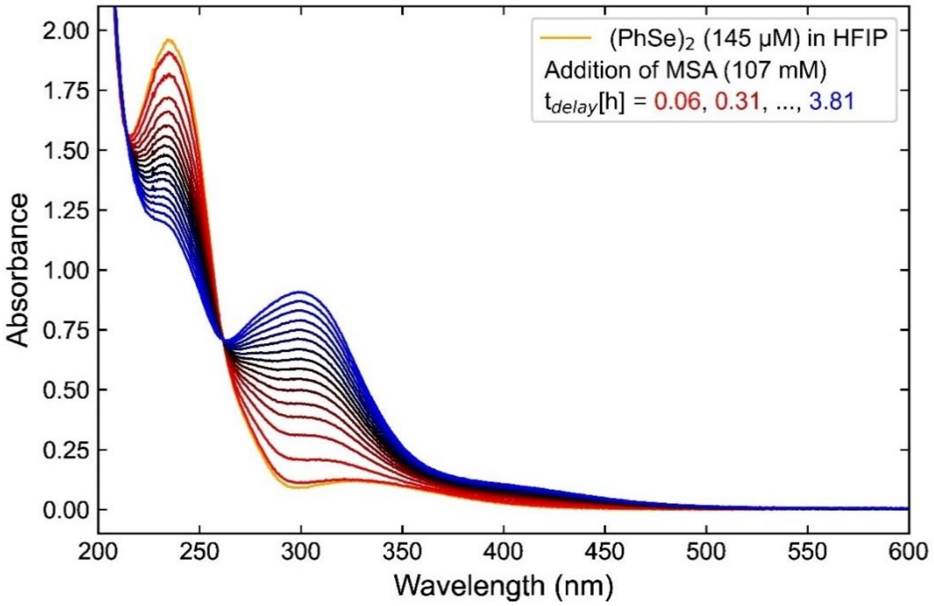

Figure S19: Electronic absorption spectra of $(PhSe)_2$ (c = 145 µM) in HFIP in the presence of an excess of 107 mM methylsulfonic acid (MSA). The spectra show the conversion of $(PhSe)_2$ to $PhSe^+$ within several hours.

For the atypical S_N1-reactions, 50 mol% trifluoroacetic acid (TFA) was used (see manuscript, Fig. 5). While to the best of our knowledge no pK_a values for MSA and TFA in HFIP are available, gas-phase studies of both acids corroborate that TFA is less acidic.[18] Thus, in a concentration of 0.3 M TFA in HFIP, which is slightly higher than the concentration used in the synthesis, the conversion of $(PhSe)_2$ to $PhSe^+$ cannot be observed within 15 hours (Figure S20).

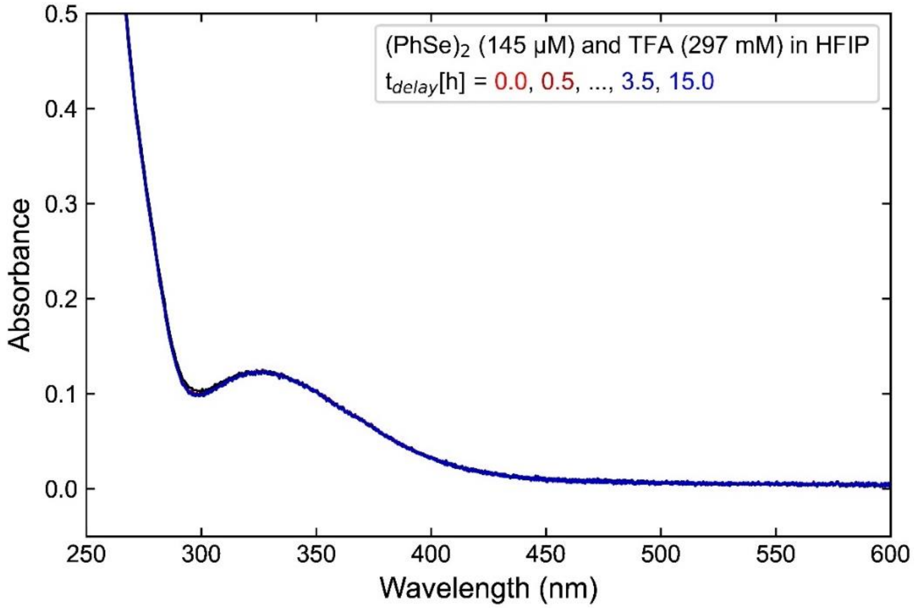

Figure S20: Electronic absorption spectra of $(PhSe)_2$ (c = 145 µM) in HFIP in the presence of an excess of 297 mM trifluoroacetic acid (TFA). The spectra do not show any degradation of $(PhSe)_2$.

Only by going to even higher concentrations of 2.6 M TFA, which is 10 times higher than the concentration used in the catalysis, a very slow reaction can be observed (Figure S24). Therefore, it is highly unlikely that the interactions between TFA and $(PhSe)_2$ drive the reaction, as the formation of the cation in the dark is significantly slower than the reaction under light. On the other hand, these interactions between $(PhSe)_2$ and acids can explain the slight contribution of a dark reaction that was observed (see Table S19, Entry 6).

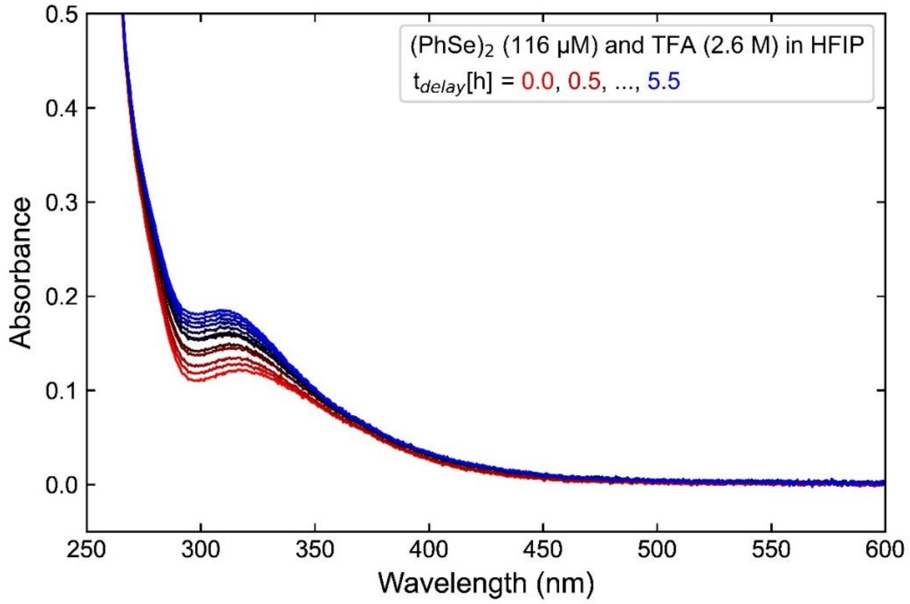

Figure S21: Electronic absorption spectra of $(PhSe)_2$ (c = 116 µM) in a mixture of HFIP and 2.6 M trifluoroacetic acid (TFA). The spectra show a slow conversion of $(PhSe)_2$ probably towards $PhSe^+$.

8 Computational calculations of the absorption spectra of the phenyl selenyl fragments

8.1 Computational Details:

All density functional theory (DFT)[19,20] and Møller-Plesset perturbation theory of second order (MP2)[21] calculations were carried out using the ORCA 5.0.4[22] quantum chemistry software package. Complete active space self-consistent field (CASSCF)[23] calculations were performed using OpenMOLCAS 21.10.[24] Geometries used for CASSCF calculations were optimised using MP2 with a cc-pVDZ basis,[25,26] geometries used for time-dependent DFT (TDDFT)[27] calculations were optimised using B3LYP[28–31]/(u)B3LYP functional including dispersion via Grimme's D4 correction[32,33] and a def2-TZVP basis[34]. TDDFT calculations were performed applying Tamm-Dancoff approximation (TDA).[35] CASSCF and XMS-CASPT2[36] calculations were performed using the cc-pVDZ basis set. For the TDDFT calculations we included solvation implicitly via the conductor-like polarisable continuum model (CPCM).[37] The following PCM parameters for HFIP were used: $\varepsilon = 15.7$; $n = 1.275$.[38,39] For the multireference calculation of the anion, it was averaged over 7 singlet and 7 triplet states, for the cation over 10 singlet and 10 triplet states. The active space for the anion was 12 electrons in 10 orbitals (Figure S25), for the cation 10 electrons in 10 orbitals (Figure S26). All DFT and MP2 calculations were performed using the resolution of identity approximation.[40] DFT calculations utilised chain of sphere exchange.[41] Charge transfer numbers were obtained using TheoDORE 3.0.[42] All minimum geometries and conical intersections in Figure S28 were optimised as implemented in ORCA 5.0.4 using non-equilibrium solvation and TDA-TDDFT. Orbitals and spin-density plots were rendered using VMD.[43]

8.2 Calculation of absorption spectra in the UV-Vis spectral range

At first, we tried to simulate all absorption spectra with a simple and fast method. TDA-TDDFT calculations yielded excellent agreement to the experiment for the phenyl selenyl anion and good agreement to the corresponding radical (Figure S22, Figure S24). Since the solvent HFIP is a strong H-bond donor and hence may coordinate to the selenium atom, the spectrum for the radical was calculated using both, pure implicit solvation and an explicit HFIP trimer together with implicit solvation. Small improvements upon the excitation energies of the phenyl selenyl radical in relation to the experiment could be observed while treating some solvent molecules explicitly (Figure S24). However, since the changes are only of minor nature, subsequently calculated excited state properties were conducted from pure implicit solvation to reduce computational time. Regarding the phenyl selenyl anion, the experimental spectrum was obtained from an acetonitrile solution. Hence, implicit solvation was included for MeCN. To exclude that explicit HFIP coordination results in a red-shift of the absorption spectrum of the phenyl selenyl anion, we also simulated the spectrum in implicit HFIP with an explicit HFIP trimer coordinating to the selenium (Figure S22).

The spectral simulation of $PhSe^+$, on the other hand, was found to be complicated due to the electronic configuration of this heavy cation (Figure S23). More specifically, $PhSe^+$ shows multireference (MR) character, as becomes apparent by two notable markers: a) very small energy gaps between the frontier molecular orbitals and b) a significant fractional occupation density number (FOD: cation >1.6).[44] In combination with a significant spin-orbit-coupling between the lowest singlet and triplet states, the electronic wavefunction of $PhSe^+$ is very complex. To replicate the absorption spectrum of $PhSe^+$ with sufficient accuracy, its computational treatment required the use of multireference methods including SOC (i.e., SOC-XMSCASPT2).[36] This approach has successfully simulated the spectrum, demonstrating good agreement with experimental results, particularly considering the unique electronic structure of the selenium cation.[45] It is noteworthy that the multireference character in the electronic ground state amounts to a nearly equal ratio of triplet and singlet states. Consequently, single reference methods such as TDDFT, or methods that only capture the spin-orbit-coupling (SOC-DFT) but not the multireference character inevitably fail to accurately reproduce the experimental spectrum of $PhSe^+$.

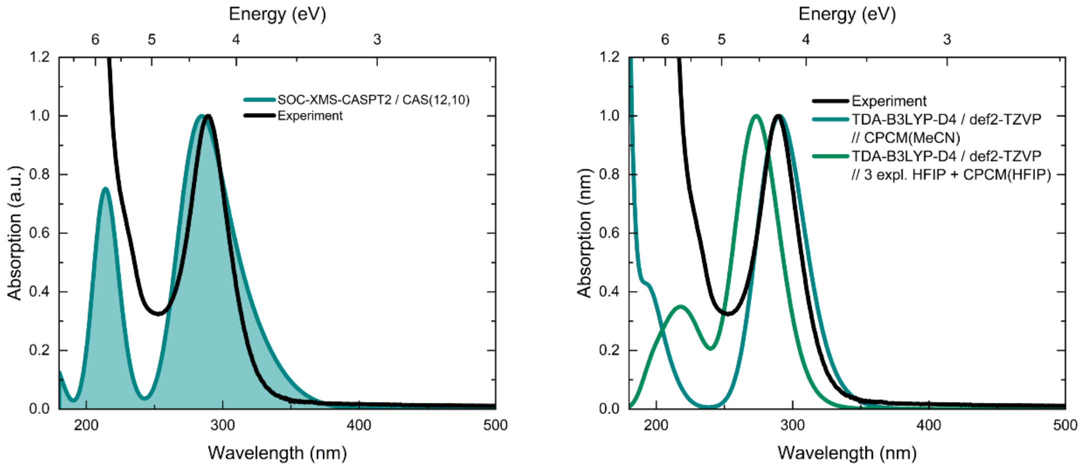

Figure S22: Calculated absorption spectra for the phenyl selenyl anion. FWHM: 3500 cm^{-1}

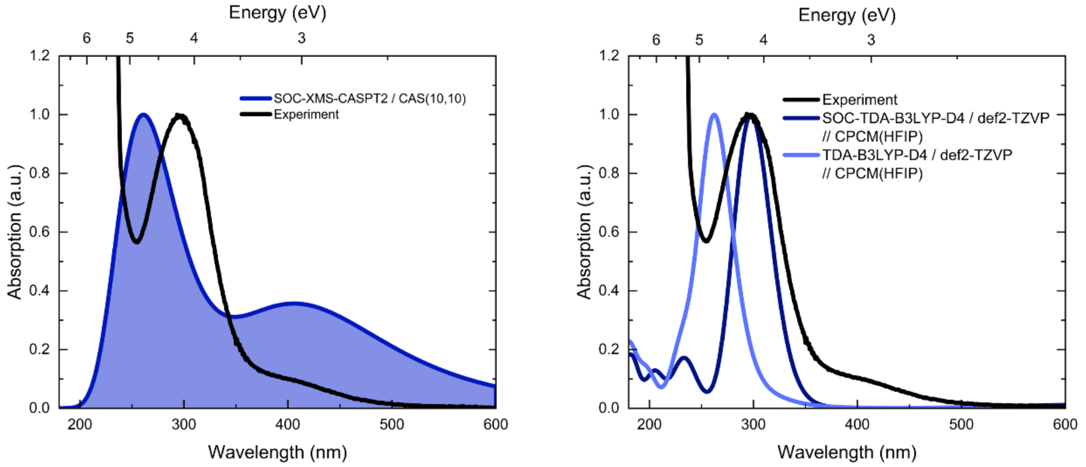

Figure S23: Calculated absorption spectra for the phenyl selenyl cation. FWHM: 3500 cm^{-1}

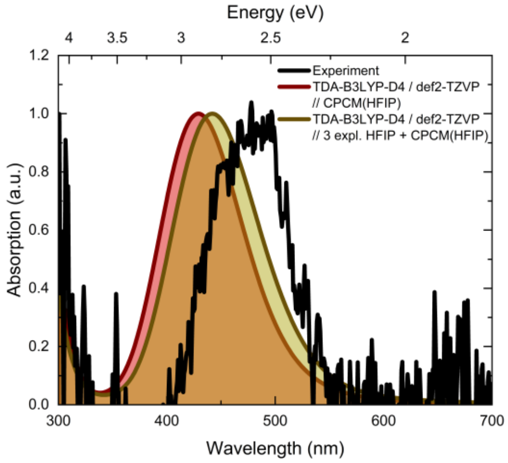

Figure S24: Calculated absorption spectrum for the phenyl selenyl radical. FWHM: 3500 cm^{-1}

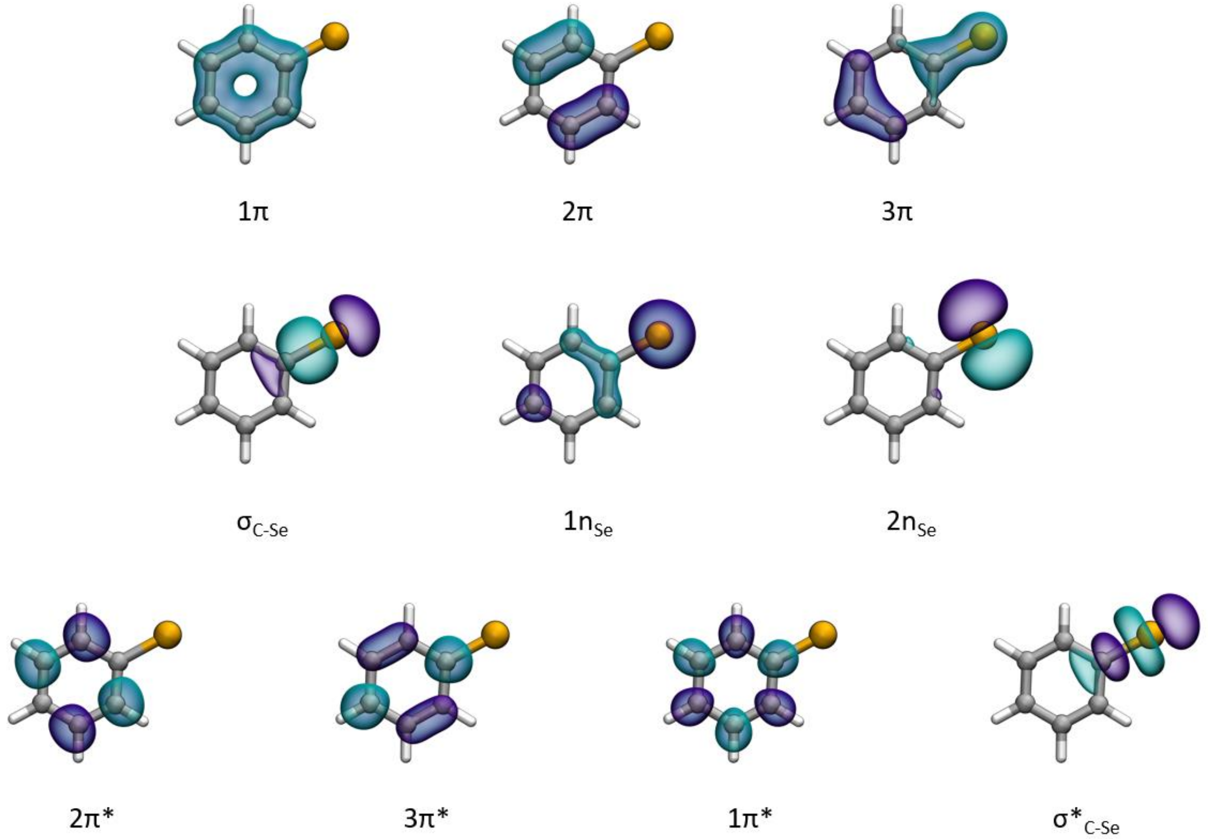

Figure S25: Orbitals included in the active space for the (12,10) CASSCF calculation of the phenyl selenyl anion.

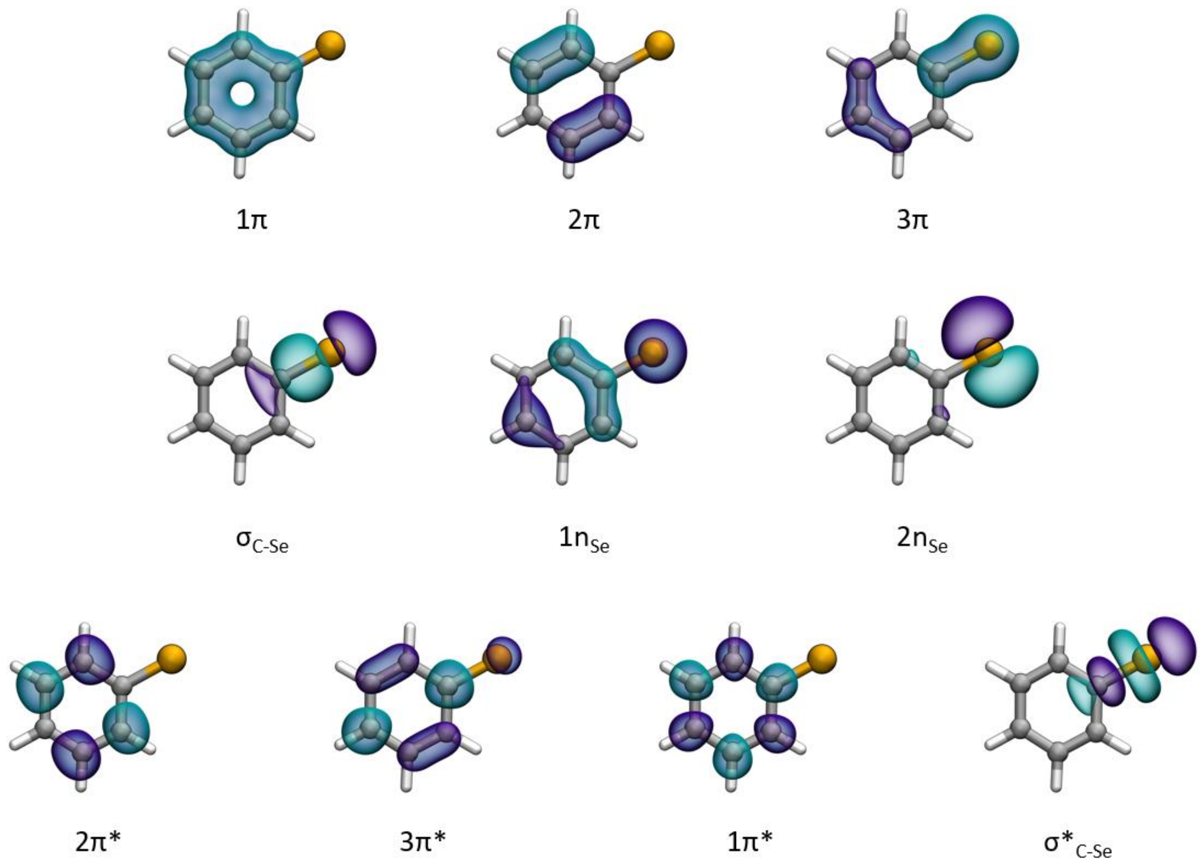

Figure S26: Orbitals included in the active space for the (10,10) CASSCF calculation of the phenyl selenyl cation.

8.3 Theoretical study of the excited states of the phenyl selenyl radical

Following the identification of PhSe• as the light-absorbing species, we conducted a computational study of its electronically excited states (Figure S27) and the corresponding deactivation pathways (Figure S28) to extract the chemically active states for its photochemistry. Irradiation with the experimentally used wavelengths mainly populates the bright D_3 state (Figure S27a) of π-p excitation character (Figure S27b). Furthermore, the D_2 state is of π-p excitation character however with larger charge transfer contributions (Figure S27b,c) which is consistent with the low extinction coefficient. The energetically lowest electronically excited doublet state (D_1) is of a n_{Se}-p charge transfer character resulting in a shift of the spin density to the selenium lone pair which is not in conjugation to the phenyl π-system (Figure S27d). Because of the large energetic difference between D_2 and D_1, we explored the excited state deactivation pathways to identify the chemically active excited state (Figure S28). Excitation to the D_3 state allows for fast relaxation to the D_2 state since the corresponding conical intersection and D_3 minimum geometry are barely split in energy. The computationally estimated excited state potential energy surfaces (Figure S27) suggest a substantial activation barrier for the non-radiative deactivation of a vibrationally cooled D_2 state to the D_1 state. Hence, a long-lived D_2 excited state is to be expected from the perspective of transition state theory, and assuming the non-radiative decay goes through the minimum energy conical intersection D_2/D_1 CI. We argue that at least part of the chemical reactivity of the excited selenyl radical origins from this long-living non-Kasha state. Nonetheless, the actual lifetime could be

lower than that estimated from transition state theory if non-radiative decay takes place through vibrational coupling between the D_2 and D_1 excited states. However, since accurate modeling of such alternative mechanism would require excited state dynamics, which is out of the scope of this paper, we argue that at least a part of the excited selenyl radicals may react from the D_2 state in an anti-Kasha manner.

a. Relevant excited states of PhSe•

State	E (eV)	f_{osc}	Symmetry	Character
D_1	0.44	0.00	A_1	n_{Se} -> p
D_2	2.30	0.00	B_1	π -> p
D_3	2.89	0.14	A_2	π -> p

b. Main contributing molecular orbitals

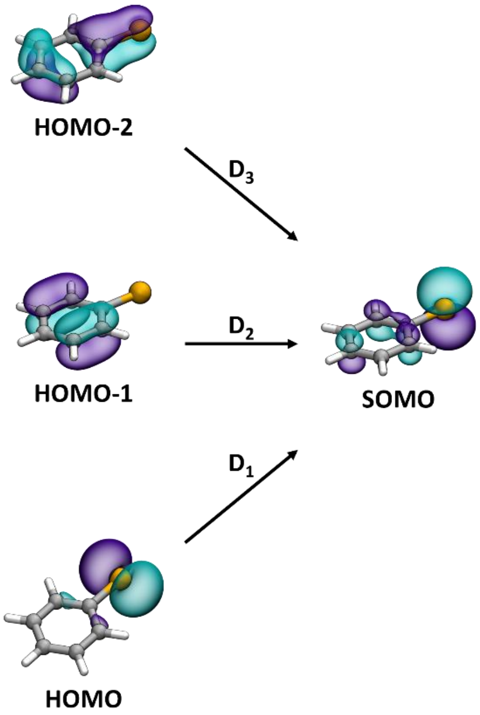

c. Charge transfer matrices

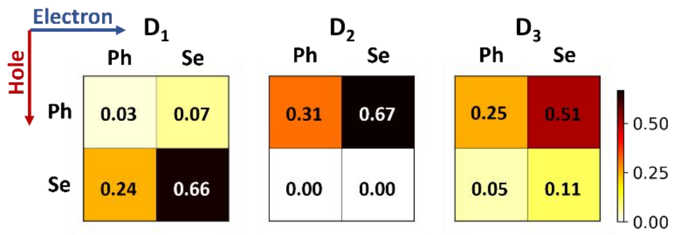

d. Spin densities of the ground and excited states

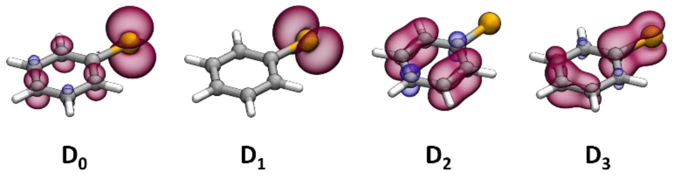

Figure S27: Electronic structure of the relevant electronically excited state of PhSe•. a. Key parameters of the excited states. b. Isosurface plots (isosurface value: 0.05) of the main contributing molecular orbitals. c. Colour coded charge transfer matrices of the corresponding excitations. Electrons hole positions are represented in rows, excited electron positions in columns. Each cell includes the charge transfer number. D. Isosurface plots (isosurface value: 0.005) of the spin density in each discussed electronic state. The bright state, which is excited in the experiment, corresponds to the D_3 showing πp character. Consequent relaxation results in a charge transfer state of πp character (D_2). The energetically lowest electronically excited state corresponds to a n_{Se}-p charge transfer state, shifting the spin density to the selenium lone pair orthogonal to the phenyl-π-system.

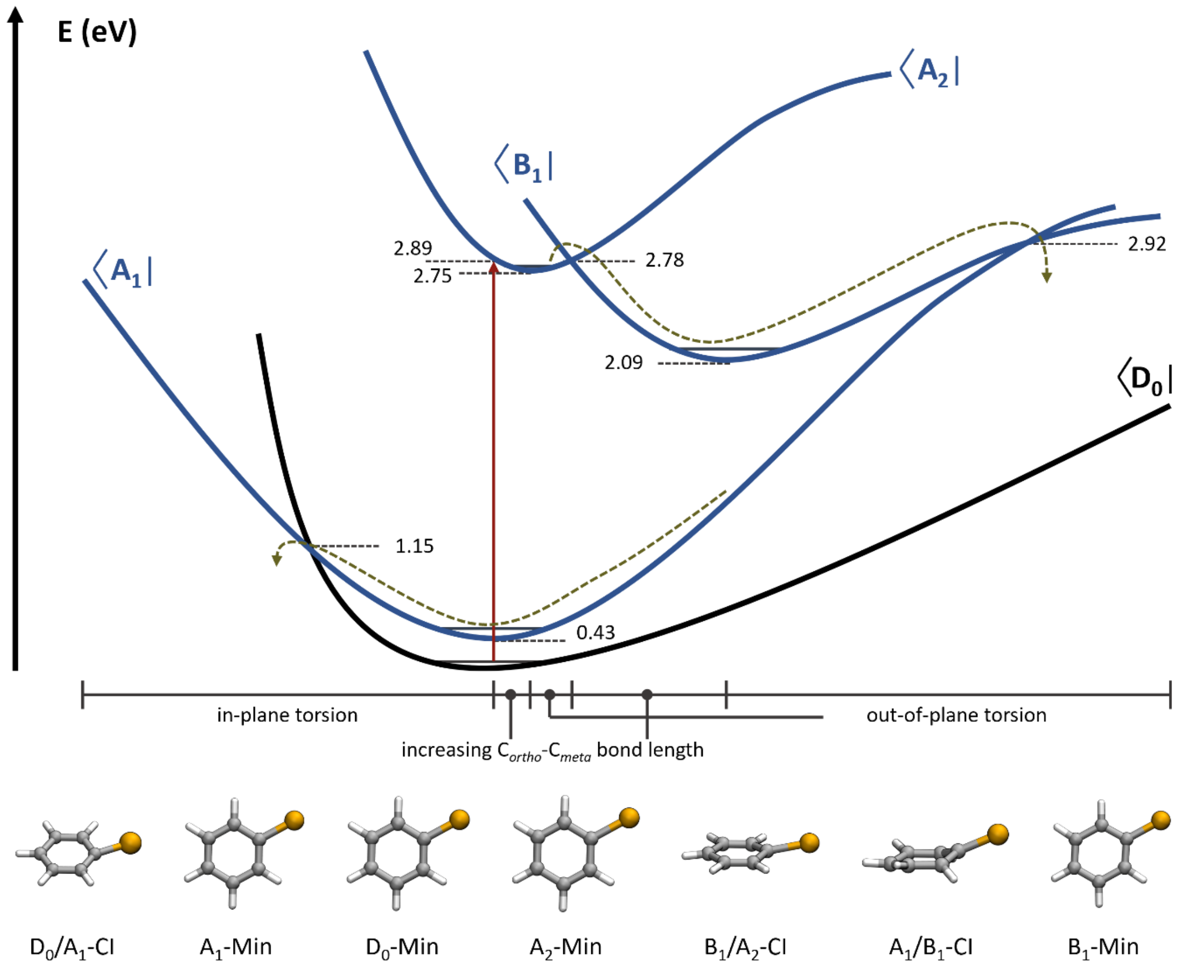

Figure S28: Deactivation pathways after excitation to the bright D_3 state. Symmetry terminology is used for the excited states. Min: adiabatic minimum geometry; CI: conical intersection.

9 Computational investigations of the stimulated doublet-doublet electron transfer

9.1 Computational Details:

All calculations were carried out using the ORCA 5.0.4[22] quantum chemistry software package. The geometries were optimised using B3LYP[28–31]/(u)B3LYP functional including dispersion via Grimme's D4 correction[32,33] with a triple zeta Ahlrich type basis set (def2-TZVP)[46]. Solvation was included during the optimisation via the conductor-like polarisable continuum model (CPCM)[37] using acetonitrile as solvent. Frequencies were calculated to confirm stationary points (zero imaginary frequencies for minimum energy structures, single imaginary frequency for saddle points of first order). For the optimisation of excited states time-dependent DFT (TDDFT) using Tamm-Dancoff approximation[35] was applied and equilibrium solvation was used. B3LYP was chosen as the exchange-correlation functional approximation as it yielded the best results in describing the phenyl selenyl radical spectrum.

9.2 Calculation of redox potentials:

To calculate the standard absolute reduction potentials, Gibbs free energy values G for all involved species are needed. Ground state G values were obtained from geometry optimisation followed by a frequency calculation to obtain thermal correctios to the electronic energy. To estimate the reduction potential of the selenyl radical in its excited states, we performed a TDDFT geometry optimisation in the D_1 and D_2 state. In order to estimate the thermodynamical contributions to the excited state geometry including the zero-point energy a numerical frequency calculation was performed.

The index in the Gibbs free energies G refers to the reduced (red) and oxidised (ox) form of a given molecule. All the following potentials refer to standard absolute reduction potentials E^0, while the index indicates if in the reaction the species is oxidised (ox) or reduced (red). The (excited state) redox potentials and corresponding potential differences were calculated according to the following equations under the assumption of infinite separation:

Standard absolute reduction potential:

$$E^0 = \frac{-(G_{\mathrm{red}} - G_{\mathrm{ox}})}{nF} \quad \textbf{(5)}$$

Redox potential difference:

$$\Delta E(\mathrm{red}, \mathrm{ox}) = E^0_{\mathrm{red}} - E^0_{\mathrm{ox}} \quad \textbf{(6)}$$

As an upper bound to the absolute coulombic interaction, the potential was calculated[47] at the equilibrium distance of the Se–C bond connecting the allyl- and the phenyl selenyl fragment in the allylselane (2.020 Å) with the dielectric constant of HFIP (ε = 15.7) and found to be −5.57 kcal/mol. As the computations show, doublet-doublet electron transfer between the allyl radical and the phenyl selenyl radical is only feasible in the case of an excited phenyl selenyl radical (Figure S8). Electron transfer from the D_1 state is only weakly exergonic (−3.81 kcal/mol) and even slightly endergonic when taking the upper bound to the absolute coulombic interaction into account. Electron transfer from the D_2 state is strongly exergonic (−41.30 kcal/mol) even under consideration of the coulombic interaction. However, under the assumption of implicit solvation, the differences in Gibbs free energy for electron transfer from the lowest electronically excited state can be considered an upper bound to an explicitly solvated system since in the D_1 state the selenium lone pairs are not fully occupied in contrast to the anion ground state. Hence, HFIP coordination may stabilise the anion ground state stronger than the D_1 state of the selenium radical.

a. non-stimulated doublet-doublet electron transfer

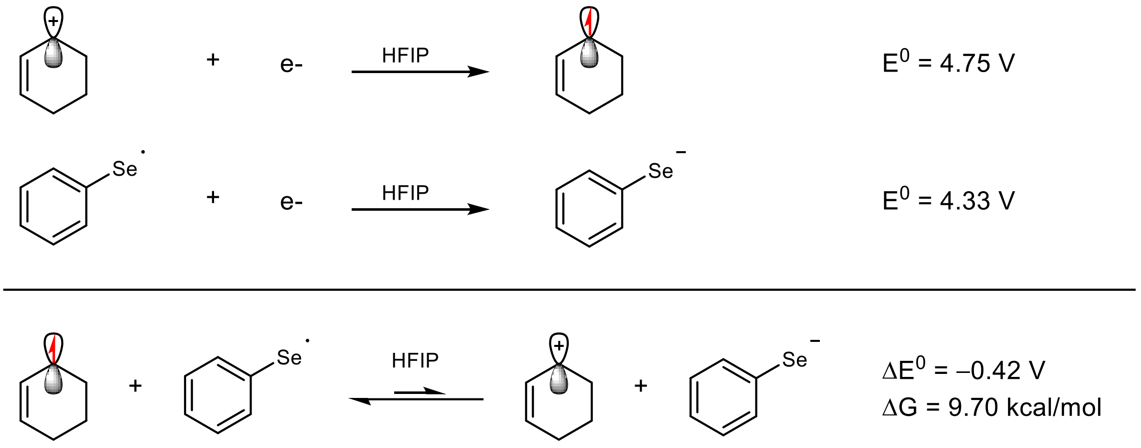

b. stimulated doublet-doublet electron transfer from the D_1 and D_2 state:

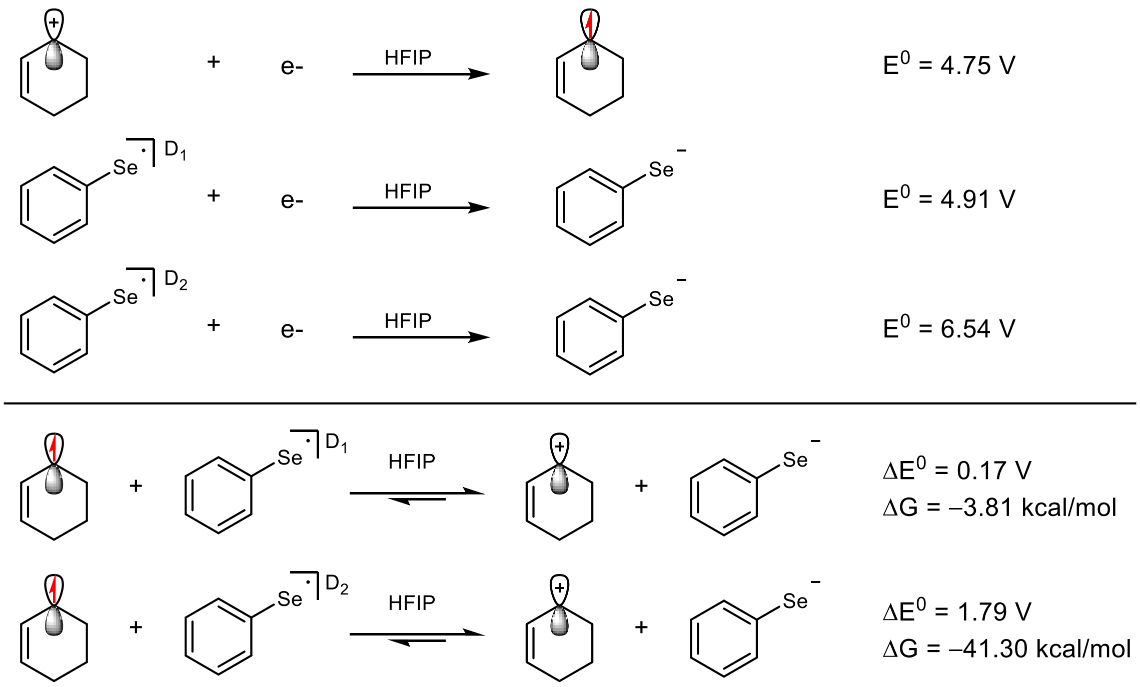

Figure S29: Computational analysis of the thermodynamics for the doublet-doublet electron transfer. **a)** without excitation of the phenyl selenyl radical, **b)** with excitation of the phenyl selenyl radical.

10 Computational investigations of the scrambling mechanism

10.1 Computational Details:

All calculations were carried out using the ORCA 5.0.4[22] quantum chemistry software package. The geometries were optimised using B3LYP[28–31]/(u)B3LYP functional including dispersion via Grimme's D4 correction[32,33] with a triple zeta Ahlrich type basis set (def2-TZVP)[34] to minimise basis set superposition error. Solvation was included during the optimisation via the conductor-like polarisable continuum model (CPCM).[37] Frequencies were calculated for the stationary points to confirm if the structure corresponds to a minimum geometry (zero imaginary frequencies) or a transition state (one imaginary frequency). Imaginary frequencies below 20 cm^{-1} were ignored as they are only an artefact of too loose integration grid size.[48] To further increase accuracy regarding thermodynamic data, high level single point energies were calculated using the range separated hybrid functional ωB97M-V[49] (to reduce self-interaction error) with a large quadruple-zeta basis set (def2-QZVPP).[34] For the single point energies, solvation was included using charge-density solvation model (SMD).[34] SMD parameters of isopropanol are available in the used software package, while the solvation model for HFIP was considered as: $\varepsilon = 15.7$; $(n^2) = 1.626$; $\alpha = 0.57$; $\beta = 0.25$; $\gamma = 20.13$; $\varphi = 0$; $\psi = 0.6$.[38,39]

10.2 Bond dissociation and interaction energies

Bond dissociation energies (BDE) and bond dissociation free energies (BDFE) were calculated according to the following equations:

BDE:

$$\Delta H = H(\text{PhSe radical}) + H(\text{Allyl radical}) - H(\text{Allylselane}) \quad \textbf{\textit{(7)}}$$

BDFE:

$$\Delta G = G(\text{PhSe radical}) + G(\text{Allyl radical}) - G(\text{Allylselane}) \quad \textbf{\textit{(8)}}$$

Interaction Energy:

$$\Delta H = H(\text{Radical with solvent trimer}) + H(\text{Radical}) - H(\text{Solvent trimer}) \quad \textbf{\textit{(9)}}$$

Interaction Free Energy:

$$\Delta G = G(\text{Radical with solvent trimer}) + G(\text{Radical}) - G(\text{Solvent trimer}) \quad \textbf{\textit{(10)}}$$

Non-covalent interactions between the trimers and the radicals are in both cases much stronger for HFIP trimers suggesting significantly stronger solvation of the radicals in HFIP (Table S21). The BDE values, however, do not change significantly by adding explicit solvation. BDFE were lowered by a small amount if using an explicit HFIP trimer in comparison to an explicit ^{i}PrOH trimer (Table S20). Comparable or slightly higher BDE values of Se–Se and Se–C have been computed by others (41.1 kcal/mol and 58.3 kcal/mol respectively)[50] and were used to explain the dynamic-covalent character of Se–Se and Se–C bonds and pathed their application in self-healing polymers.[51] A more recent study transferred the mechanistic concept of [2+1] path identified for disulfide exchange reactions onto diselenide exchange reactions (see also chapter 10.4 Radical addition elimination mechanism). This indicates that the mechanistic path to the Ar–Se-radicals may involve dimers or aggregates in general that feature suitable kinetics for a fast thermal exchange despite of BDEs being on average in the region of 40–50 kcal/mol.[52]

Table S20: BDE and BDFE of the allylselanes with and without explicit solvation.

Entry	Name	Structure	BDE (kcal/mol)	BDFE (kcal/mol)
1	Allylselane (2a) Implicit solvation (HFIP)	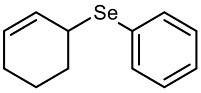	45.3	31.3
2	Allylselane (2a) Explicit solvation (HFIP-trimer)	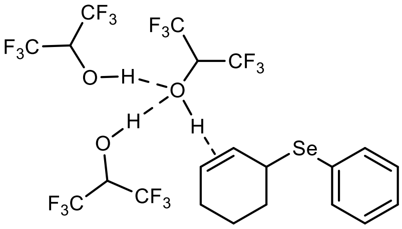	45.6	28.5
3	Allylselane (2a) Explicit solvation (*ⁱ*PrOH-trimer)	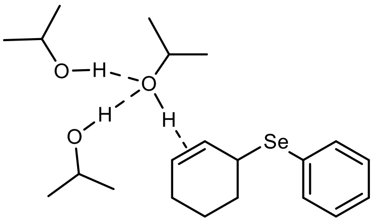	45.2	29.9

Table S21: Non-covalent interaction energies between the free radicals and solvent trimers.

Entry	Name	Structure	Interaction Enthalpy (kcal/mol)	Interaction Free Energy (kcal/mol)
1	Phenyl selenyl radical Explicit solvation (HFIP-trimer)	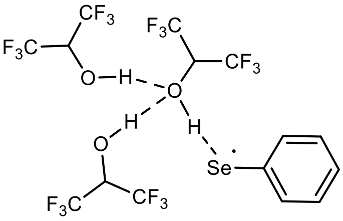	−7.1	9.2
2	Allyl radical Explicit solvation (HFIP-trimer)	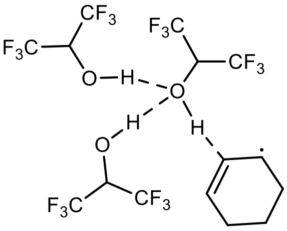	−6.9	9.7
3	Phenyl selenyl radical Explicit solvation (^{i}PrOH -trimer)	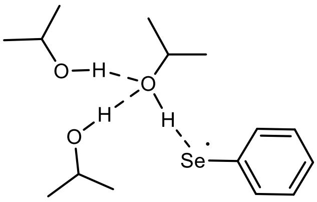	−3.5	10.0
4	Allyl radical Explicit solvation (^{i}PrOH -trimer)	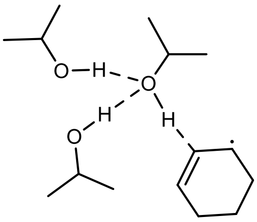	−3.3	11.0

Considering the large differences between BDE and BDFE, entropical contributions have to play a major role in the dissociation of the allyl selenide (Table S22). Since the Se–C bond connecting the allyl and the phenyl selenyl fragment is not freely rotatable due to noncovalent-interactions (Figure S30), the rotational contributions to the entropy of the allyl selane are barely higher than the rotational entropy values of the resulting fragments. Together with a present electronic entropy in the radical fragments, BDFEs are particularly lower than corresponding BDEs in this example.

Table S22: Contributions to the entropy *S* correction regarding the BDFE calculations. The indices correspond to the following contributions: el: electronic, vib: vibrational, rot: rotational, trans: translational.

**Name**	**S_{el} (kcal/mol)**	**S_{vib} (kcal/mol)**	**S_{rot} (kcal/mol)**	**S_{trans} (kcal/mol)**	**Sum (kcal/mol)**
Allyl selane	0.00	10.35	9.53	12.61	32.45
Allyl radical	0.41	2.53	7.77	11.66	22.37
Selenyl radical	0.41	2.92	8.53	12.24	24.10

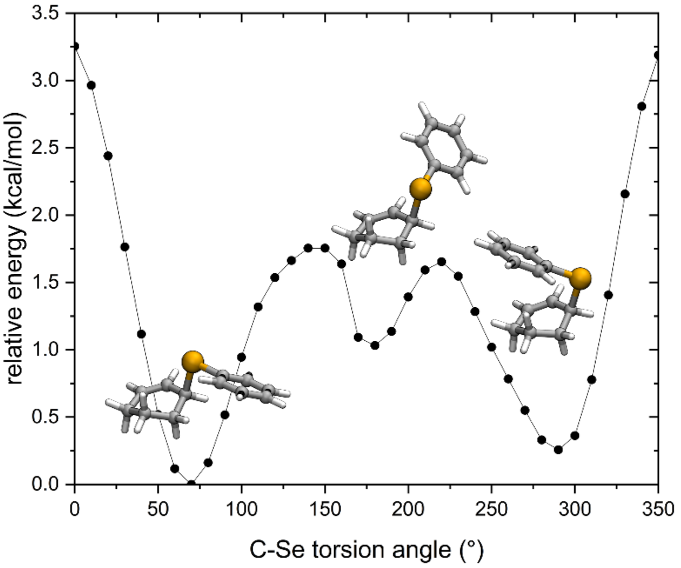

Figure S30: Relaxed surface scan along the torsion around the C-Se bond connecting the allyl and the phenyl selenyl fragment. The Gibbs free energy relative to the global minimum is plotted on the y-axis. Rendered structures for the local minima are shown as insets.

10.3 Chemical stability of the phenyl selenyl radical in solution

To derive a physical justification for the increased chemical stability and hence, less efficient radical recombination after homolysis, of the phenyl selenyl radical in a HFIP solution, orbital energies of implicitly and explicitly solvated radicals shall be compared (Figure S31). One finds the interesting trend, that all the orbital energies of the relevant SOMOs in alpha state are reduced from implicitly solvated, to explicitly solvated by an *iso*-propanol trimer to explicitly solvated by a HFIP trimer. This results in a n energetic lowering of the reactive SOMO leading to a reduced chemical reactivity in solution and hence, bigger lifetime values. The origin of this

trend may be assigned to the H-bond donation strength of HFIP coordinating to the selenium lone pair.

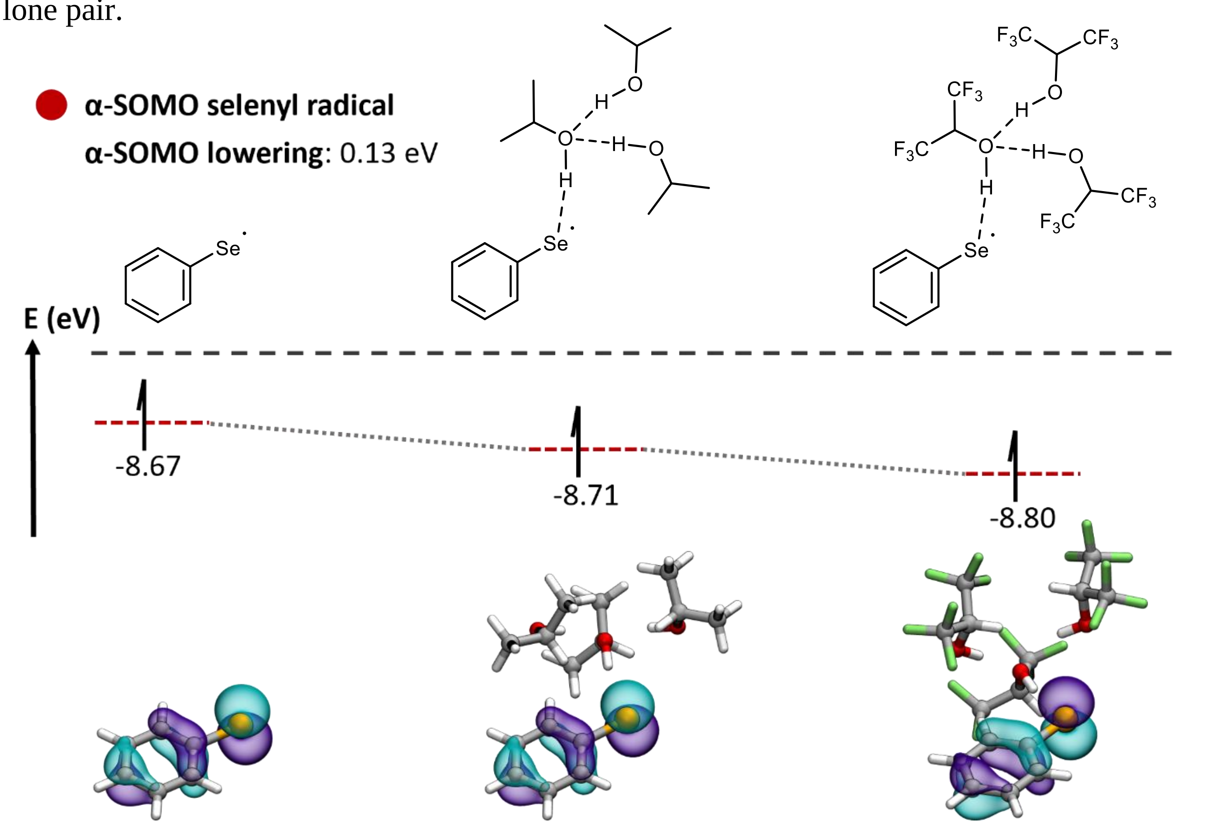

Figure S31: Calculated orbital energies in eV. Red colouring indicates the alpha-spin singly occupied molecular orbitals of the selenyl radical. Isosurface plots (isosurface value: 0.05) of the corresponding molecular orbitals are shown at the bottom.

10.4 Radical addition elimination mechanism

While considering that no spectral contributions of free phenyl selenyl radicals could be observed during the scrambling experiments, we suggest a radical addition elimination chain-mechanism[53] to occur (Figure S32). Fast addition of a phenyl selenyl radical to the unsaturated carbon-carbon bond in the allylselenide **2a** leads to a transient carbon-centred radical intermediate. The corresponding activation barrier of 18.3 kcal/mol is low enough for fast addition at room temperature. Subsequent elimination yields again a free phenyl selenyl radical and the allylselane **2a**. This mechanism explains both a selenyl radical concentration below the sensitivity threshold of the respective spectroscopy and a fast scrambling as soon as a low amount of free radicals is present accounting for the high BDEs.

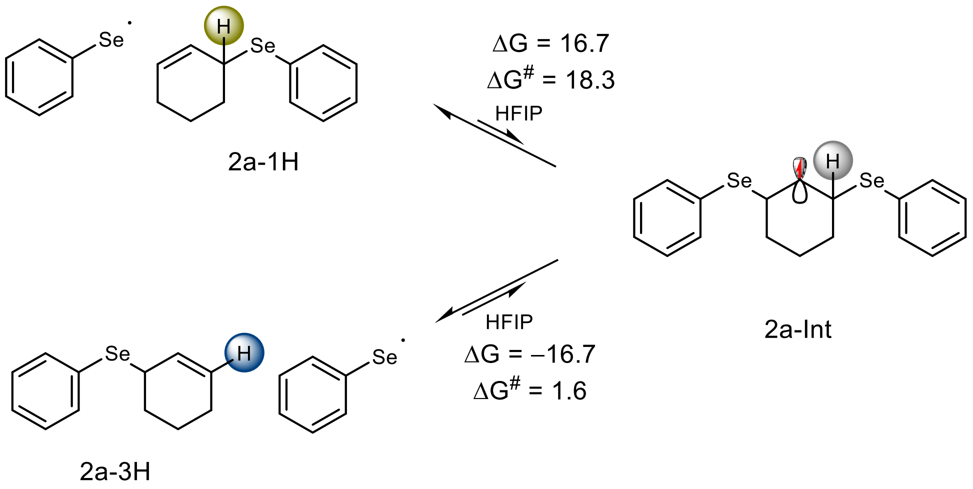

Figure S32: Computationally studied mechanism leading to the observed scrambling. All energy values are given in units of kcal/mol.

11 Assignment of the regioisomers

In the reactions with more than one nucleophilic nitrogen atom in the reactant, the formation of regioisomers in the titular reaction was observed.

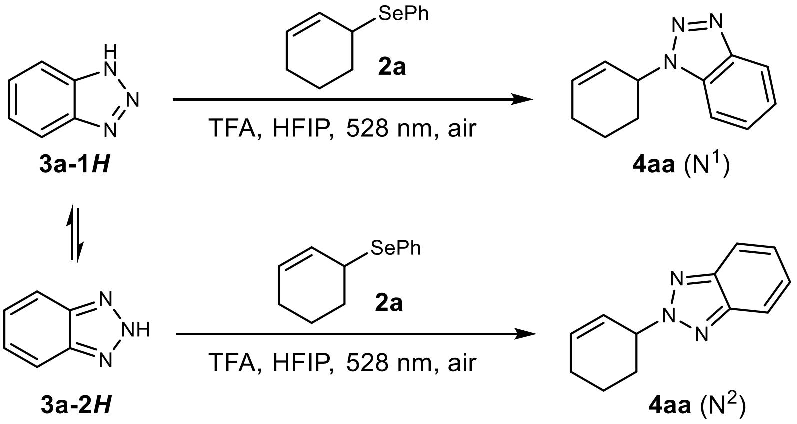

Scheme S5: Photoreaction of the two tautomers 1*H*- and 2*H*-benzotriazol-**3a-1*H*** and **3a-2*H*** with selenide **2a** to give the two substitution products **4aa** (N^1) and **4aa** (N^2).

To assign the correct structure to the isolated compounds and make a sensible statement about the regioselectivity of the reaction, NOESY spectra of the concerning products were recorded and analysed. Due to the nuclear Overhauser effect, cross-relaxation between ^{1}H nuclei can be observed and correlated to a physical proximity of the atoms in a molecule.

11.1 1*H*-Benzotriazol (3a)

The interaction between the allylic or olefinic protons and the aromatic protons of the benzotriazole moiety should result in a NOESY signal in the case of structure **4aa** (N^1) due to a close proximity, while the signals should not be visible in the spectrum of structure **4aa** (N^2) due to a larger distance between the same protons. A comparison of aromatic, olefinic and allylic signals in the NOESY spectra of the isolated compounds is shown below:

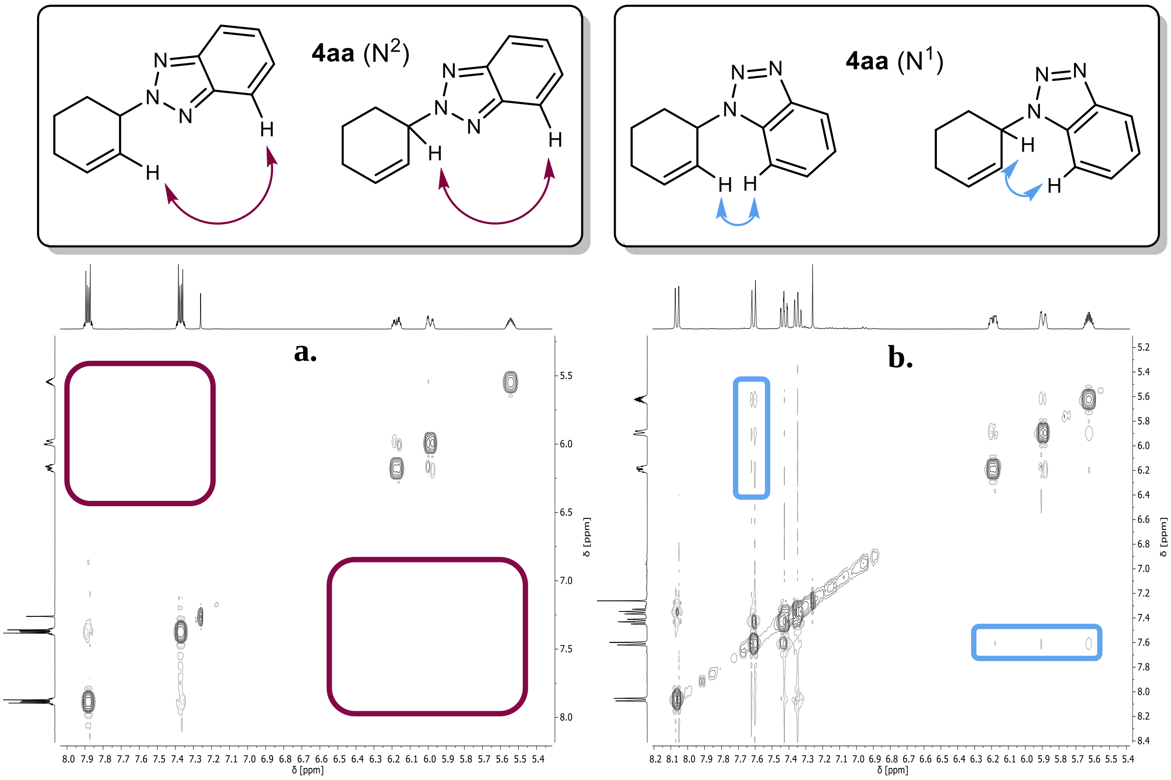

Figure S33: Comparison of the NOESY-spectra of **a.** the first isolated compound with a R_f-value of 0.5 showing no cross peaks between the allylic signal (5.54 ppm) or the olefinic signals (5.99 ppm and 6.18 ppm) and the aromatic signals (7.37 ppm and 7.88 ppm) and **b.** the second isolated compound with a R_f-value of 0.19 showing cross peaks between both the allylic signal (5.62 ppm) and one aromatic signal (7.61 ppm) and the olefinic signals (5.89 ppm and 6.19 ppm) and the same aromatic signal (7.61 ppm).

11.2 5-Methyl-1*H*-benzo[*d*][1,2,3]triazole (3b)

The interaction between the allylic or olefinic protons and the aromatic protons of the benzotriazole moiety should result in a NOESY signal in the case of structures **4ab** ($N^{1/3}$) due to a close proximity, while the signals should not be visible in the spectrum of structure **4ab** (N^2) due to a larger distance between the same protons. The first fraction only included one isomer (as judged by the methyl signal), while the second fraction included two isomers. Since no NOE cross-relaxation was observed in fraction one, it can be assigned to structure **4ab** (N^2) while the two isomers **4ab** ($N^{1/3}$) are assigned to fraction two. A comparison of aromatic, olefinic and allylic signals in the NOESY spectra of the isolated compounds is shown below:

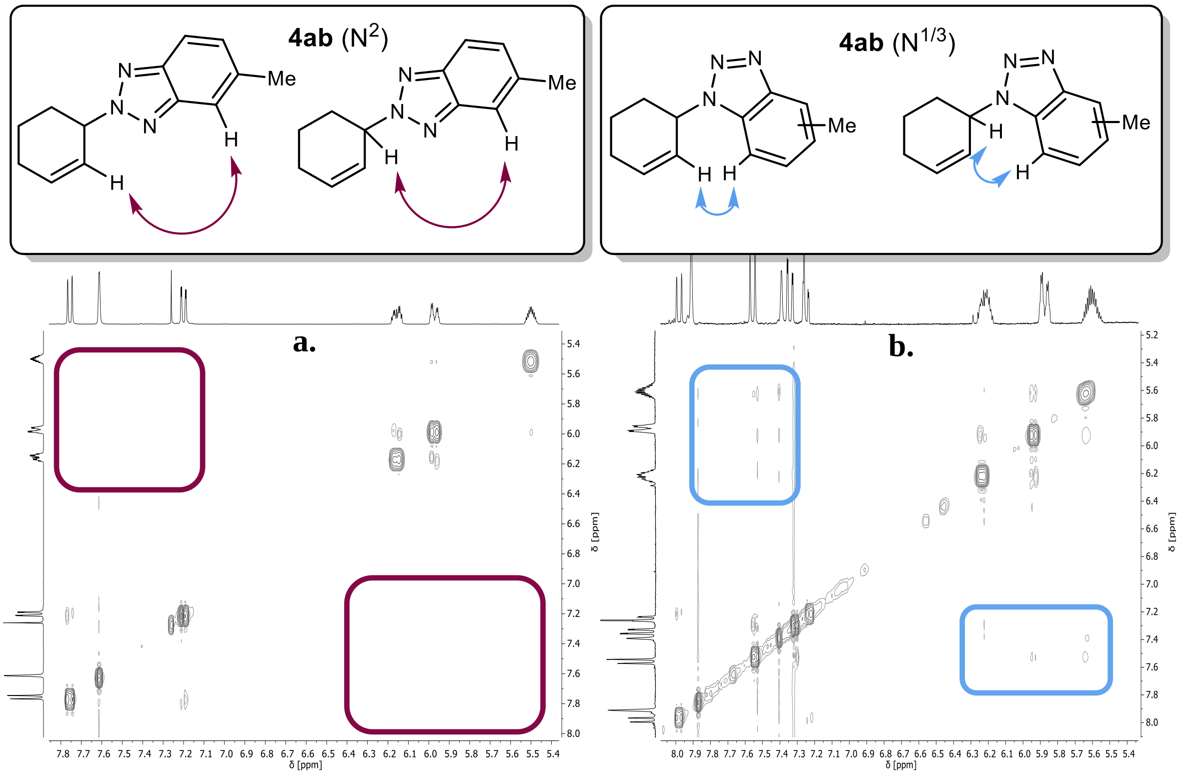

Figure S34: Comparison of the NOESY spectra of **a.** the first isolated compound with a R_f -value of 0.5 showing no cross peaks between the allylic signal (5.55 ppm) or the olefinic signals (5.97 ppm and 6.15 ppm) and the aromatic signals (7.20 ppm, 7.61 ppm and 7.75 ppm) and **b.** the second isolated compound with a R_f-value of 0.25 showing cross peaks between both the allylic signal (5.60 ppm) and the olefinic signals (5.86 ppm and 6.19 ppm) and aromatic signals (7.26–7.85 ppm).

To differentiate between the remaining isomers in fraction two, the ^{1}H NMR spectrum was considered. When calculating the predicted signals via Ensemble NMR Prediction[54], it becomes apparent that two proton signals should be significantly shifted in the two isomers, differing even in the arrangement relative to each other.

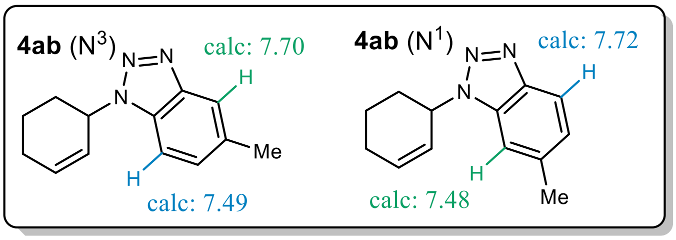

Figure S35: ^{1}H NMR spectroscopy ppm values of unique aromatic signals (green and blue) of structures **4ab** (N^3) and **4ab** (N^1) calculated via Ensemble NMR Prediction.

An even stronger effect than with the calculated values can be observed in the experimental spectra. Here, values of the signals differ by 0.50 ppm and 0.42 ppm respectively. Structures **4ab** (N^3) and **4ab** (N^1) were assigned to the remaining isomers accordingly.

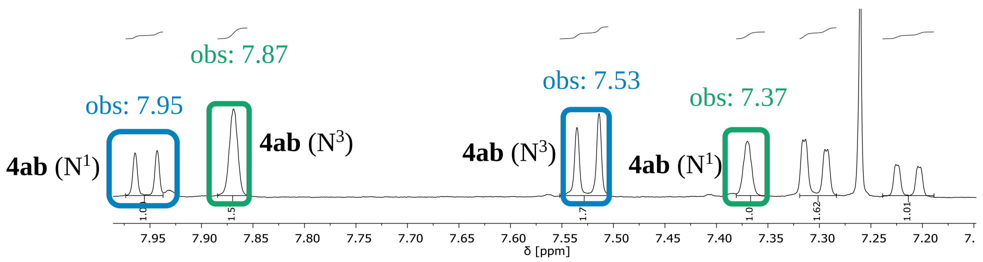

Figure S36: ^{1}H NMR spectroscopy analysis of two unique aromatic protons (blue and green) of the isomeric mixture of **4ab** (N^{1}) and **4ab** (N^{3}). The major isomer (integral of 1.6) and the minor isomer (integral of 1.0) were assigned to the structures **4ab** (N$^{1/3}$) due to a comparison with the values calculated via Ensemble NMR Prediction.

11.3 5-Chloro-1*H*-benzo[*d*][1,2,3]triazole (3c)

The interaction between the allylic or olefinic protons and the aromatic protons of the benzotriazole moiety should result in a NOESY signal in the case of structures **4ac** (N$^{1/3}$) due to a close proximity, while the signals should not be visible in the spectrum of structure **4ac** (N^{2}) due to a larger distance between the same protons. Since no NOE cross-relaxation was observed in fraction one, it can be assigned to structure **4ac** (N^{2}) while the two isomers **4ac** (N$^{1/3}$) are assigned to fraction two. A comparison of aromatic, olefinic and allylic signals in the NOESY spectra of the isolated compounds is shown below:

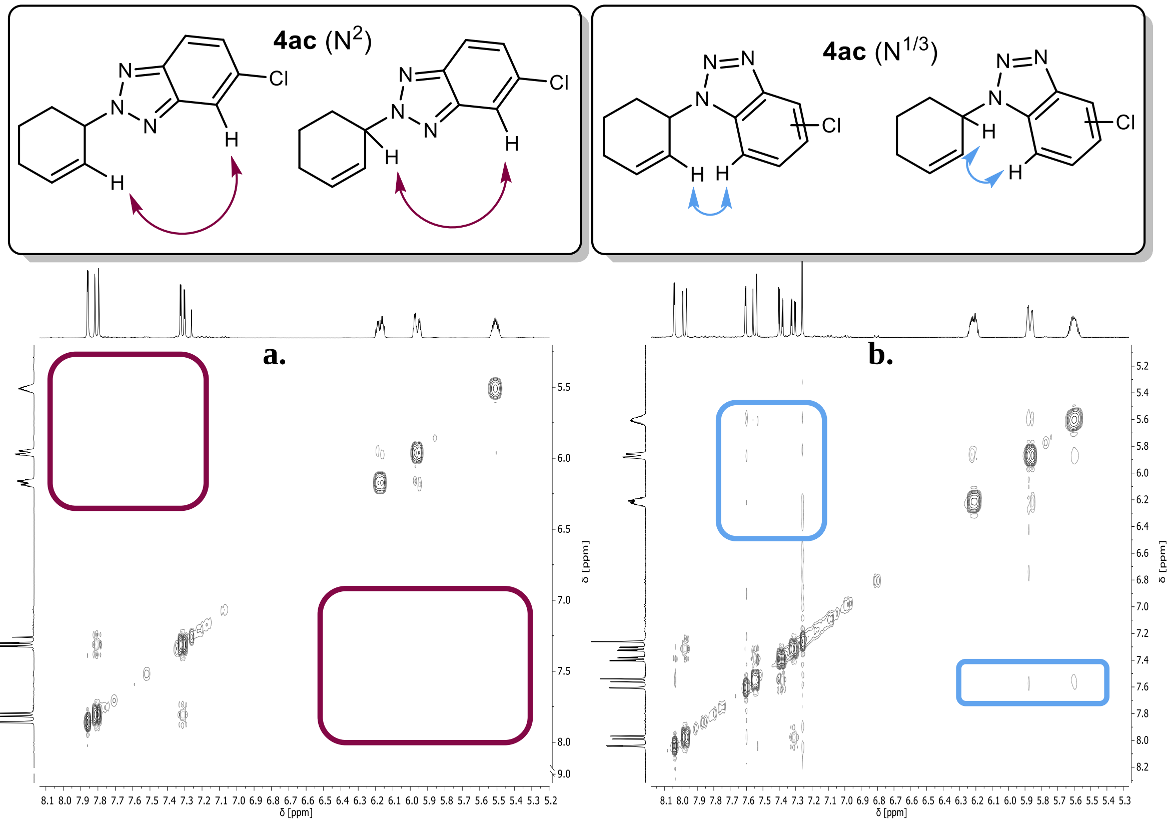

Figure S37: Comparison of the NOESY spectra of **a.** the first isolated compound with a R_f -value of 0.38 showing no cross peaks between the allylic signal (5.52 ppm) or the olefinic signals (5.95 ppm and 6.11 ppm) and the aromatic signals (7.30 ppm, 7.80 ppm and 7.87 ppm) and **b.** the second isolated compound with a R_f-value of 0.13 showing cross peaks between both the allylic signal (5.60 ppm) and the olefinic signals (5.86 ppm and 6.22 ppm) and aromatic signals (7.20–7.60 ppm).

To differentiate between the remaining isomers in fraction two, the ^{1}H NMR spectrum was considered. When calculating the predicted signals via Ensemble NMR Prediction, it becomes apparent that two proton signals should be significantly shifted in the two isomers, differing even in the arrangement relative to each other.

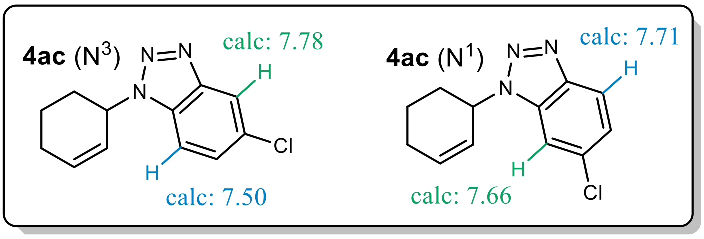

Figure S38: ^{1}H NMR spectroscopy ppm values of unique aromatic signals (green and blue) of structures of **4ac** (N^{1}) and **4ac** (N^{3}). calculated via Ensemble NMR Prediction.

A similar effect to the calculated values can be observed in the experimental spectra. Here, values of the signals differ by 0.43 ppm. Structures **4ac** (N^{3}) and **4ac** (N^{1}) were assigned to the remaining structures accordingly.

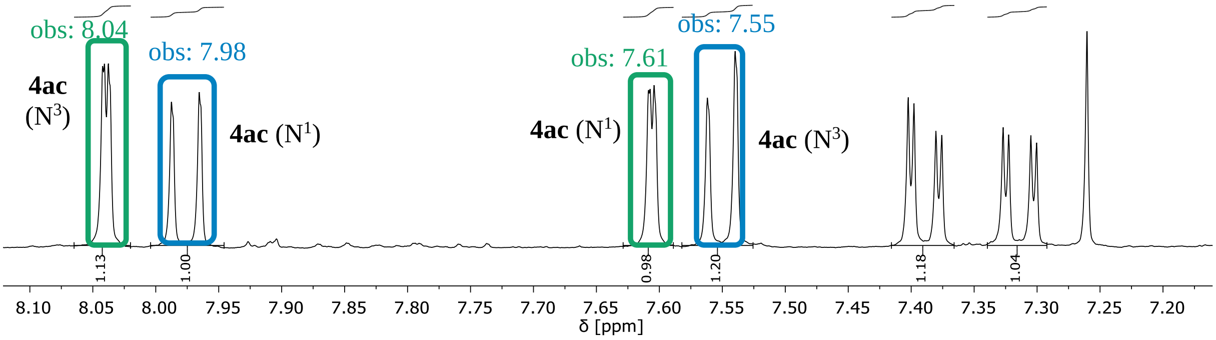

Figure S39: ^{1}H NMR spectroscopy analysis of two unique aromatic protons (blue and green) of the isomeric mixture of **4ac** (N^{1}) and **4ac** (N^{3}). The major isomer (integral of 1.2) and the minor isomer (integral of 1.0) were assigned to the structures **4ac** (N^{3}) and **4ac** (N^{1}). due to a comparison with the values calculated via Ensemble NMR Prediction.

11.4 1*H*-[1,2,3]triazolo[4,5-*b*]pyridine (3d)

The interaction between the allylic or olefinic protons and the aromatic protons of the benzotriazole moiety should result in a NOESY signal in the case of structure **4ad** (N^{3}) due to a close proximity, while the signals should not be visible in the spectrum of structure **4ad** (N^{1}) and **4ad** (N^{2}) due to a larger distance between the same protons. Since no NOE cross-relaxation was observed in fraction one and two, they can be assigned to structures structure **4ad** (N^{1}) and **4ad** (N^{2}) while isomer **4ad** (N^{3}) is assigned to fraction three due to a visible cross peak. A comparison of aromatic, olefinic and allylic signals in the NOESY spectra of the isolated compounds is shown below:

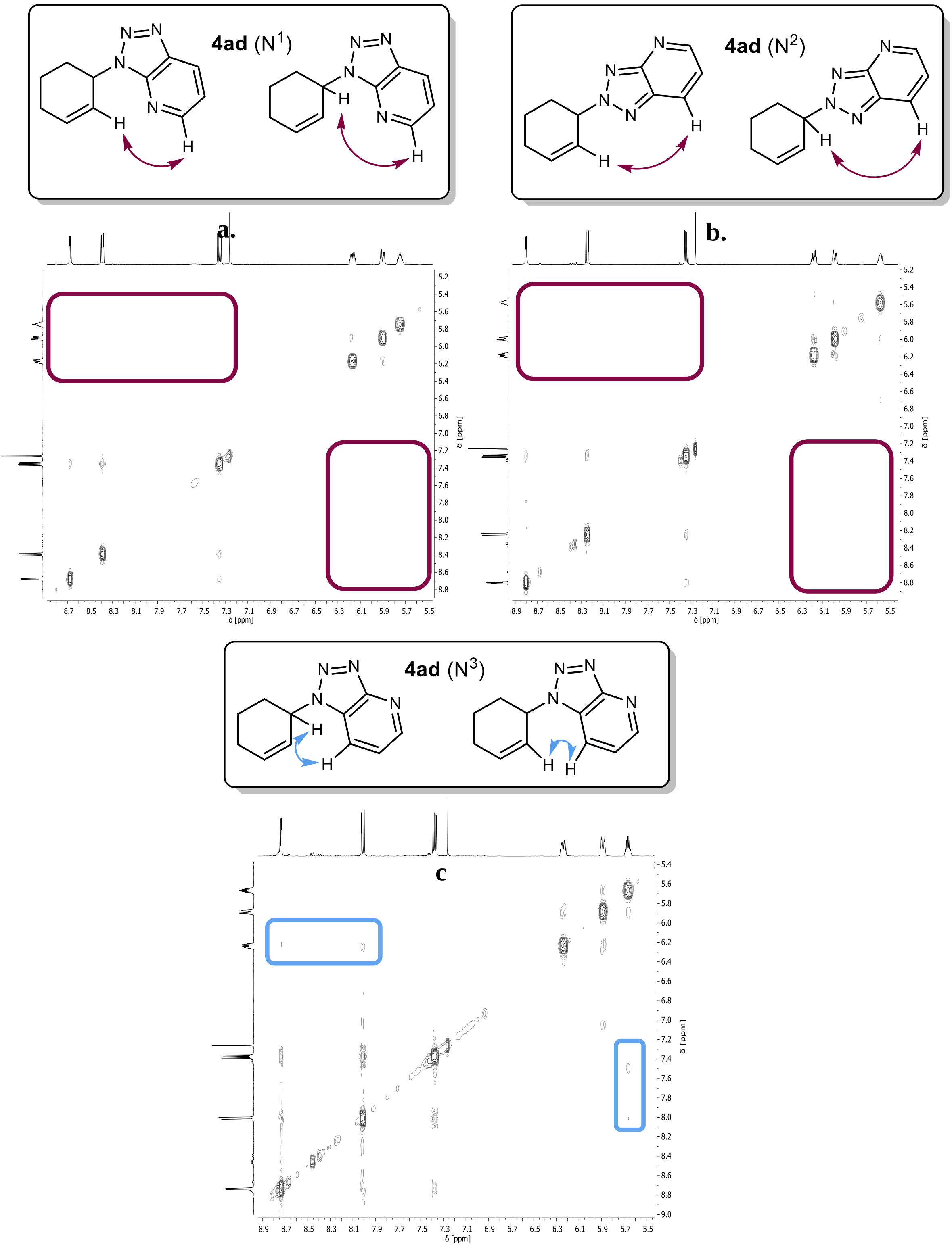

Figure S40: Comparison of the NOESY spectra of **a.** the first isolated compound with a R_f -value of 0.43 showing no cross peaks between the allylic signal (5.75 ppm) or the olefinic signals (5.90 ppm and 6.17 ppm) and the aromatic signals (7.35 ppm, 8.35 ppm and 8.68 ppm), **b.** the second isolated compound with a R_f -value of 0.38 showing no cross peaks between the allylic signal (5.57 ppm) or the olefinic signals (5.99 ppm and 6.17 ppm) and the aromatic signals (7.33 ppm, 8.25 ppm and 8.80 ppm) and **c.** the third isolated compound with a R_f-value of 0.08 showing cross peaks between both the allylic signal (5.66 ppm) and the olefinic signals (5.88 ppm and 6.24 ppm) and aromatic signals (7.48–8.73 ppm).

To differentiate between the remaining isomers in fraction one and two, the ^{13}C NMR was considered. When calculating the predicted signals via Ensemble NMR Prediction, it becomes apparent that one signal should be significantly shifted the two isomers, differing in up to 15 ppm values.

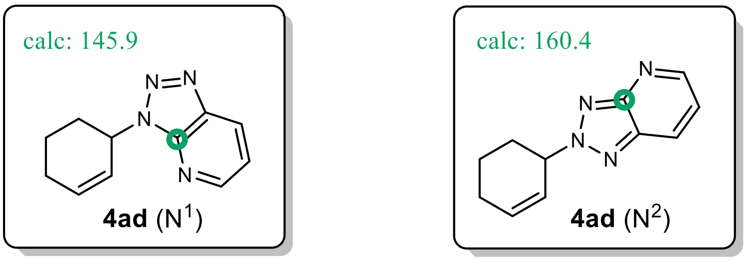

Figure S41: ^{13}C NMR ppm values of unique aromatic signals of structures **4ad** (N^1) and **4ad** (N^2) calculated via Ensemble NMR Prediction.

Although not as strong as with the calculated values, a similar effect can be observed in the experimental spectra. Here, values of 155.8 ppm and 145.6 ppm were obtained. Thus, structures **4ad** (N^1) and **4ad** (N^2) were assigned to the respective structures accordingly.

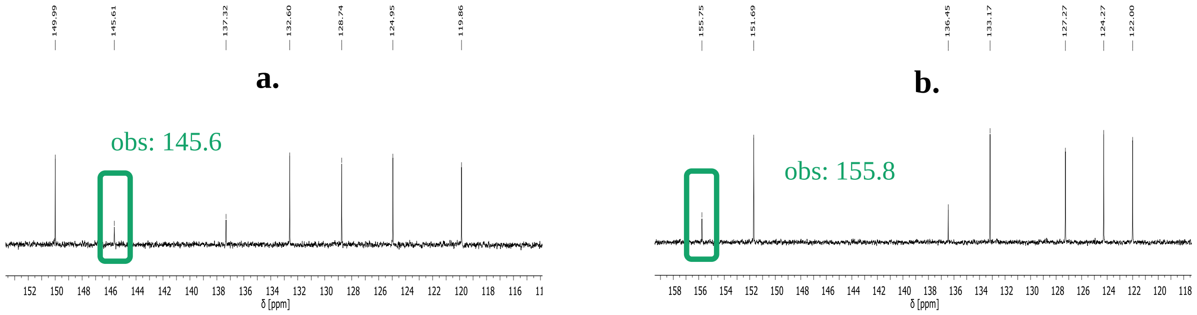

Figure S42: ^{13}C NMR analysis of two unique aromatic carbon atoms of the two remaining fractions containing structures **4ad** (N^1) and **4ad** (N^2). The first (**a.**) and second (**b.**) fraction were assigned to the structures **4ad** (N^1) and **4ad** (N^3) due to a comparison with the values calculated via Ensemble NMR Prediction.

11.5 1*H*-Tetrazole (3e)

The interaction between the allylic or olefinic protons and the aliphatic protons of the methyl moiety should result in a NOESY signal in the case of structure **4ae** (N^1) due to a close proximity, while the signals should not be visible in the spectrum of structure **4ae** (N^2) due to a larger distance between the same protons. A comparison of olefinic and allylic signals as well as the methyl group in the NOESY spectra of the isolated compounds is shown below:

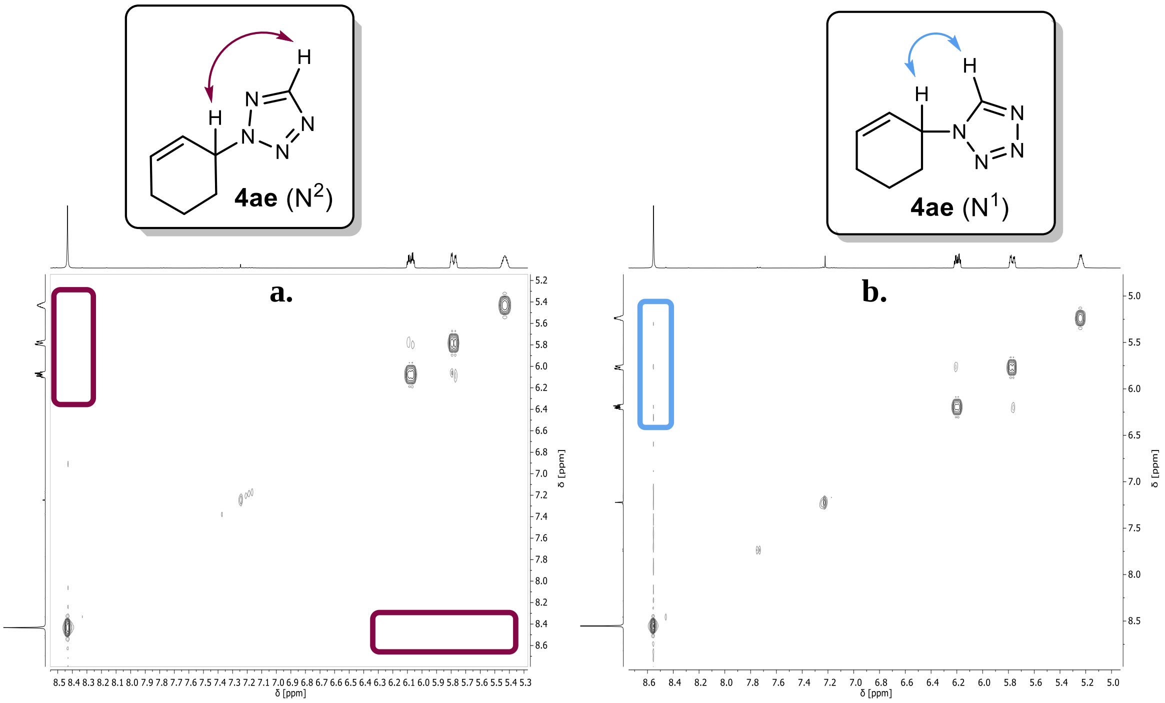

Figure S43: C Comparison of the NOESY spectra of **a.** the first isolated compound with a R_f-value of 0.36 showing no cross peaks between the allylic signal (5.43 ppm) or the olefinic signals (5.78 ppm and 6.08 ppm) and the proton in the heterocycle (8.42 ppm) and **b.** the second isolated compound with a R_f-value of 0.17 showing cross peaks between both the allylic signal (5.23 ppm) and the olefinic signals (5.77 ppm and 6.19 ppm) and the proton in the heterocycle (8.55 ppm).

Therefore, the isomeric mixture of **4ae** (N^2)/**4ae** (N^1) was determined to be 3.6:1.0 in the isolated compounds.

11.6 5-Methyl-1*H*-tetrazole (3f)

The interaction between the allylic or olefinic protons and the aliphatic protons of the methyl moiety should result in a NOESY signal in the case of structure **4af** (N^1) due to a close proximity, while the signals should not be visible in the spectrum of structure **4af** (N^2) due to a larger distance between the same protons. A comparison of olefinic and allylic signals as well as the methyl group in the NOESY spectra of the isolated compounds is shown below:

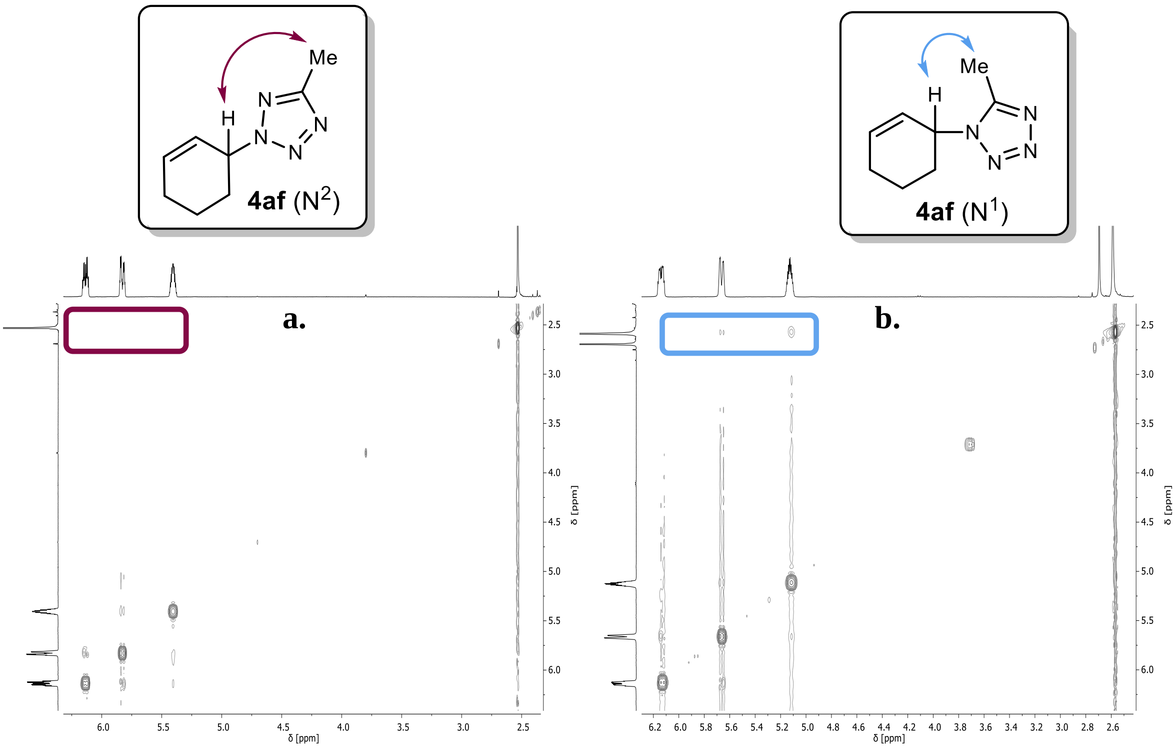

Figure S44: Comparison of the NOESY spectra of **a.** the first isolated compound with a R_f-value of 0.4 showing no cross peaks between the allylic signal (5.40 ppm) or the olefinic signals (5.82 ppm and 6.13 ppm) and the methyl group (2.52 ppm) and **b.** the second isolated compound with a R_f-value of 0.1 showing cross peaks between both the allylic signal (5.10 ppm) and one olefinic signal (5.66 ppm) and the methyl group (2.58 ppm).

Therefore, the isomeric mixture of **4af** (N^2)/**4af** (N^1) was determined to be 10.0:1.0 in the isolated compounds.

11.7 5-Phenyl-1*H*-pyrazole (3i)

The interaction between the allylic proton and the aromatic protons of the phenyl moiety should result in a NOESY signal in the case of structure **4ai** (N^2) due to a close proximity, while the signals should not be visible in the spectrum of structure **4ai** (N^1) due to a larger distance between the same protons. Respectively, the interaction between the olefinic protons and the aromatic proton of the heterocycle should result in a NOESY signal in the case of structure **4ai** (N^1) due to a close proximity, while the signals should not be visible in the spectrum of structure **4ai** (N^2) due to a larger distance between the same protons A comparison of aromatic, olefinic and allylic signals in the NOESY spectra of the isolated compounds is shown below:

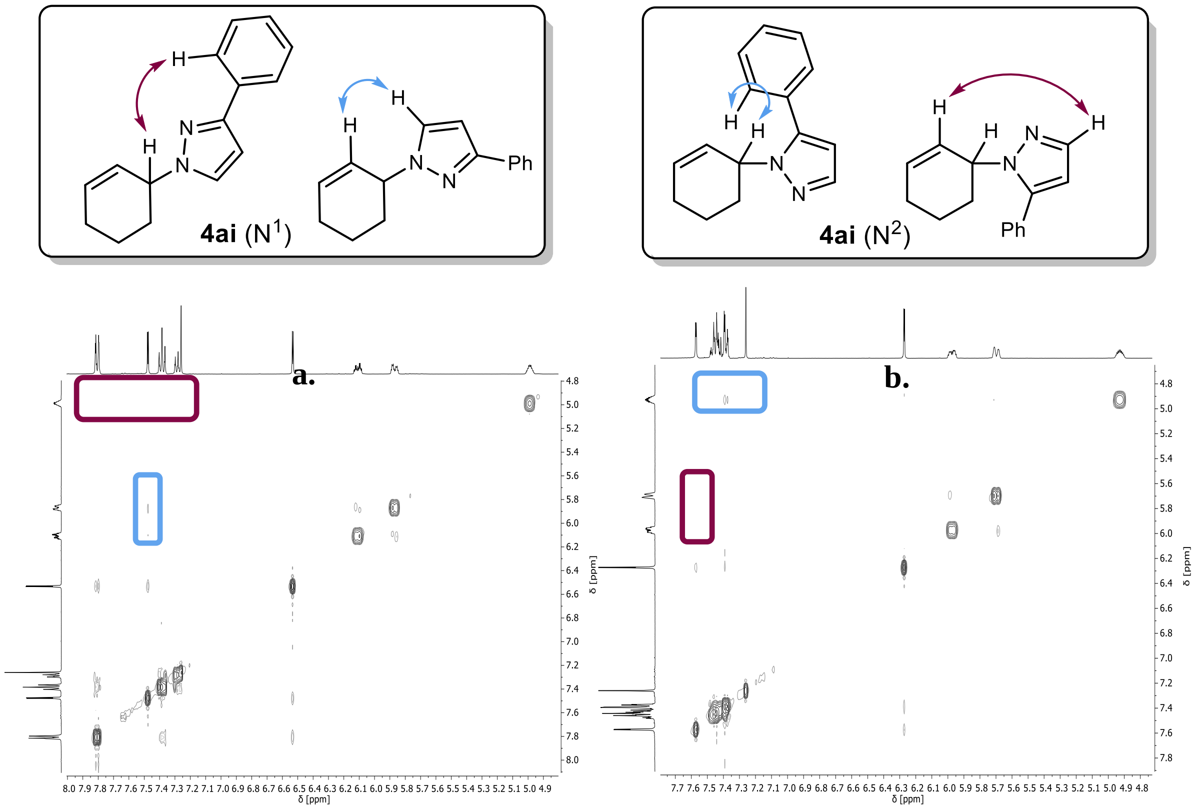

Figure S45: Comparison of the NOESY spectra of **a.** the first isolated compound with a R_f-value of 0.34 showing no cross peaks between the allylic signal (5.00 ppm) and the aromatic signals, but showing a cross peak between the olefinic signals (5.86 ppm and 6.10 ppm) and one aromatic signal (7.47 ppm) and **b.** the second isolated compound with a R_f-value of 0.2 showing a cross peaks between the allylic signal (4.92 ppm) and one aromatic signal (7.38 ppm), but showing no cross peak between the olefinic signals (5.68 and 5.96 ppm) and the aromatic signals.

Therefore, the isomeric mixture of **4ai** (N^2)/**4ai** (N^1) was determined to be 3.5:1.0 in the isolated compounds.

11.8 Valsartan (3u)

In the case of the nucleophile Valsartan, the isomers could not be separated on the column, instead an isomeric mixture was isolated. Since the signals of the two isomers are overlaying in case of the signals that could be considered in the NOESY spectrum, namely the allylic and olefinic protons, a different spectrum had to be used. In the ^{13}C spectrum, ppm values can be predicted through calculation with Ensemble NMR Prediction. When regarding those values, it becomes apparent that the signals of the carbon that is directly attached to the nucleophile should be noticeably shifted in the ^{13}C spectrum.

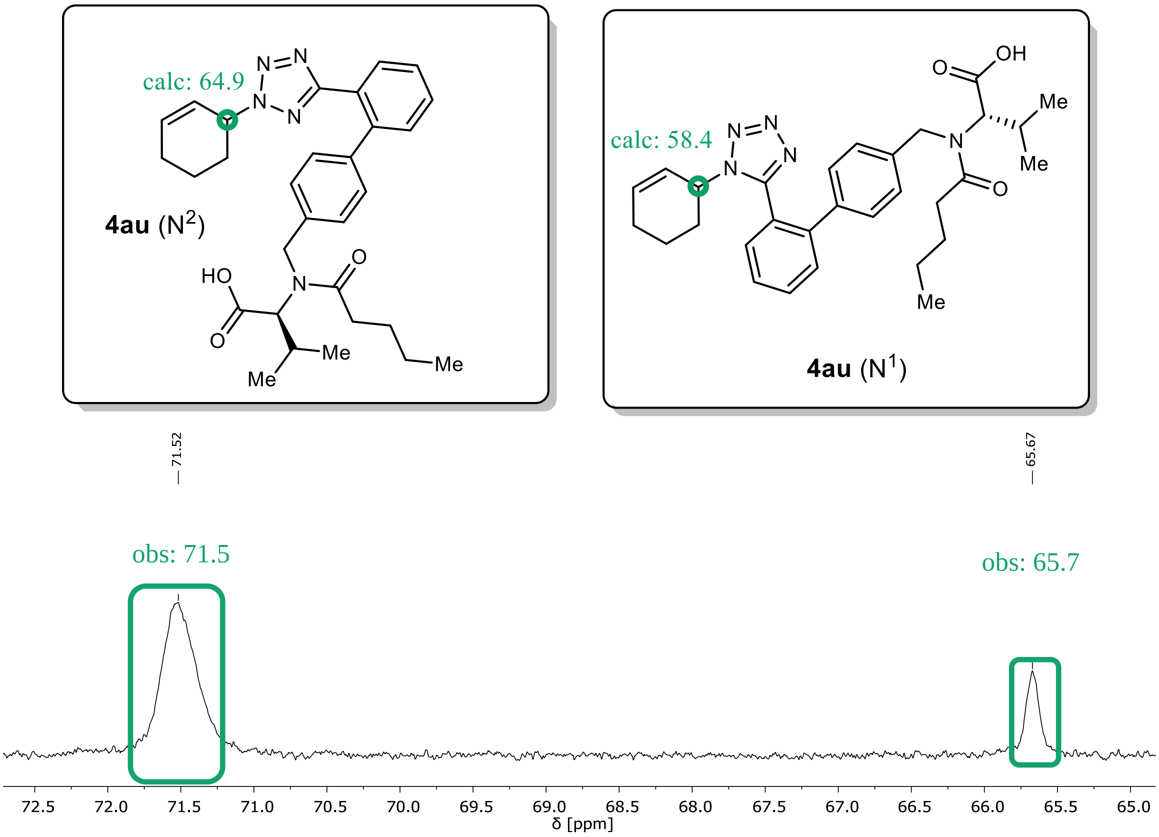

Figure S46: ^{13}C NMR analysis of the two unique allylic carbon atoms of the two structures **4au** (N^2) and **4au** (N^1). The major and minor isomer were assigned to the structures **4au** (N^2) and **4au** (N^1) due to a comparison with the values calculated via Ensemble NMR Prediction.

Indeed, a notable shift in ppm value could be observed, thus allowing the assignment of structure **4au** (N^2) as the major isomer and structure **4ai** (N^1) as the minor isomer.

12 Selectivity of the atypical S_N1-amination reactions

Since we were able to asign all isolated compounds to the respective structures via analysis of the NMR spectra (see chapter 11 Assignment of the regioisomers), we now wanted to compare our novel atypical S_N1-amination with classic S_N1 reaction profiles of halide analogues **9** and **9'**.

12.1 1*H*-Benzotriazol

Table S23: Regioselectivity comparison of desired reaction and control experiment in the reaction of 1*H*-bezotriazol.

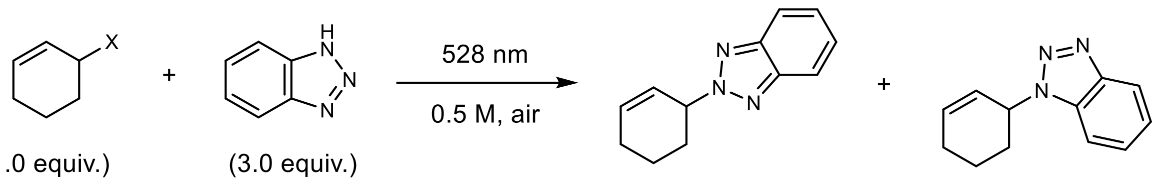

(1.0 equiv.)
X = SePh: **2a**
X = Br: **9**

(3.0 equiv.)
3a

4aa (N^2)

4aa (N^1)

Entry	X = ?	Solvent	Additive	Light	Time	Yield N^2 (%)[a]	Yield N^1 (%)[a]	Ratio
1	Se	HFIP	TFA (50 mol%)	yes	5 h	59	25	1:0.42
2	Br	HFIP	TFA (50 mol%)	no	5 h	75	25	1:0.33
3	Br	HFIP	K_2CO_3 (1.0 equiv.)	no	5 h	64	21	1:0.33
4	Br	MeCN	K_2CO_3 (1.0 equiv.)	no	5 h	38	55	1:1.45

[a]Yields determined via ^{1}H NMR spectroscopy and internal standard 1,3-dinitrobenzene. All reactions showed full conversion.

We observed that **2a** and **9** displayed a similar reactivity concerning both yield and N^2/N^1 regioselectivities (rs) when the reaction was performed in HFIP irrespective of the additive that was added to the reactions of **9** (Entry 1–3). On the other hand, running the reaction with substrate **9** in MeCN with a base additive resulted in a orthorgonal selectivity and a preferred formation of the N^1 isomere (Entry 4), further highlighting the major role of HFIP and hydrogen-bond networks in our SDET reactions.

12.2 Aniline

Table S24: Regioselectivity comparison of desired reaction and control experiment in the reaction of aniline.

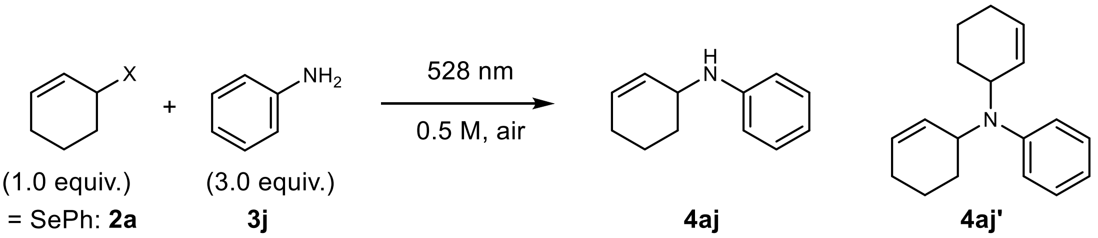

(1.0 equiv.)
X = SePh: **2a**
X = Br: **9**

(3.0 equiv.)
3j

4aj

4aj'

Entry	X = ?	Solvent	Additive	Light	Time	Conversion (%)[a]	Yield **4aj** (%)[a]	Yield **4aj'** (%)[a]
1	Se	HFIP	TFA (50 mol%)	yes	5 h	100	82	0
2	Br	HFIP	TFA (50 mol%)	no	3 h	100	12	4
3	Br	HFIP	K_2CO_3 (1.0 equiv.)	no	3 h	100	52	6
4	Br	MeCN	K_2CO_3 (1.0 equiv.)	no	5 h	100	90	5

[a]Yields determined via ^{1}H NMR spectroscopy and internal standard 1,3-dinitrobenzene, **4aj'** confirmed via HRMS ((EI-MS) [$C_{18}H_{23}N$] ($[M]^{+\bullet}$), obs.: 253.1820, calcd.: 253.1825)

Next, we compared the reactivity profile of other *N*-nucleophiles in oure SDET protocol with established S_N1 reactions on allyl bromide **9**. While out method resulted in a nearly quantitative formation of the desired product **4aj** (Entry 1), starting material **9** resulted in a severly reduced yield of only 12% under the same conditions (Entry 2). Addition of base instead of acid resulted in a acceptable yield of 52% (Entry 3). However, only switching the solvent from HFIP to MeCN resulted in a comparable yield of 90% of **9** to our new SDET method (Entry 4). In the light-mediated reaction of selenide **2a**, we observed a selective monofunctionalisation of aniline (**3j**) to obtain only **4aj** in good yields. In all three reactions with bromide **9**, the formation of a second product signal that we attributed to the difunctionalisation product (confirmed via HRMS) could be observed, thus further highlighting the selectivity of our novel protocol.

12.3 4-Methylbenzenesulfonamide

Table S25: Regioselectivity comparison of desired reaction and control experiment in the reaction of 4-methyl-benzenesulfonamide.

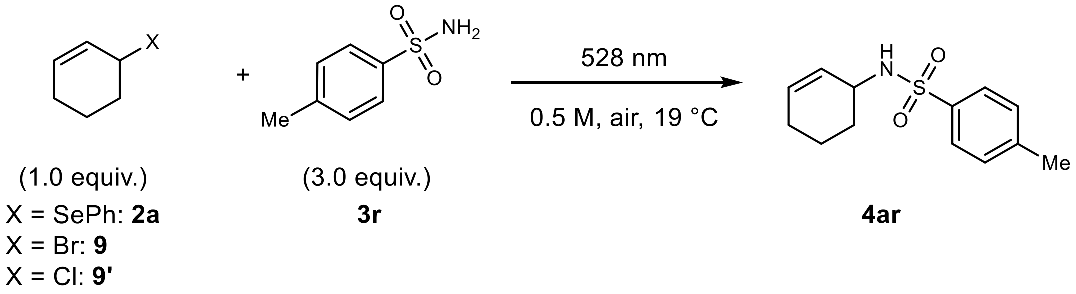

Entry	X = ?	Solvent	Additive	Light	Time	Conversion (%)[a]	Yield (%)[a]
1	Se	HFIP	TFA (50 mol%)	yes	3 h	100	96
2	Br	HFIP	TFA (50 mol%)	no	3 h	100	24
3	Br	HFIP	K_2CO_3 (1.0 equiv.)	no	3 h	100	68
4	Br	MeCN	K_2CO_3 (1.0 equiv.)	no	5 h	74	19
5	Se	HFIP	TFA (50 mol%)	yes	0.5 h	100	96
6	Br	HFIP	TFA (50 mol%)	yes	0.5 h	83	25
7	Cl	HFIP	TFA (50 mol%)	yes	0.5 h	100	52

[a]Yields determined via ^{1}H NMR spectroscopy and internal standard 1,3-dinitrobenzene.

Since our reactions with the nucleophile 4-methylbenzene-sulfonamide (**3r**) resulted in the selective formation of the primary substituted allyation product **4ar** –and thus a tosyl-protected primary amine– in nearly quantitative yields (Entry 1), we were wondering if this was an immanent feature of our reaction protocol or if established reaction conditions would achieve a similar result. Indeed, applying our reaction conditions to starting material **9** resulted in a severly inhibited product formation of only 24% (Entry 2). As was the case with nucleophile **3j**, exchanging the acid additive for the base K_2CO_3 increased the yield to a value of 68% (Entry 3). In contrast to chapter 12.2 Aniline, changing the solvent to MeCN as it is often applied in established S_N1 reactions resulted in the lowest yield of 19% (Entry 4), thereby demonstrateing a possible asset of our novel reaction protocol. This was also demonstrated trough directly comparing cyclohex-2-en-1-ylhalides (Hal = Cl, Br) using the same reaction conditions that furnished product **4ar** in quantitative yields for selenide **2a** after only 30 minutes (Entry 5). Indeed, we observed that the reaction with cyclohex-2-en-1-ylbromide (**9**) did not result in a full conversion after the same time with a significantly decreased yield of of 25% (Entry 6). While the corresponding chloride (**9'**) did result in fullk conversion, a decreased yield of 52% was observed under the same reaction conditions (Entry 7).

13 Synthesis and analytical data of starting materials

General Procedure A: Synthesis of allylic and benzylic selenides

Following literature procedure:[55] The diselenide (0.500 equiv.) was dissolved in EtOH (dried over mol sieves, 1.0 M) under nitrogen atmosphere at 0 °C. $NaBH_4$ (1.30 equiv.) was added portion wise at this temperature and stirred until a clear solution was formed. The bromide (1.0 equiv.) was added, and the solution was stirred overnight at rt. The mixture was extracted with EA, and washed with 10% NaOH, saturated aqueous $NaHCO_3$ solution, and brine. The combined organic phases were dried over Na_2SO_4 and concentrated to give the crude mixture that was purified by column chromatography to give the desired product.

General Procedure B: Synthesis of secondary allylic selenides

Following literature procedure:[56] The allylic selenide (1.00 equiv.) was dissolved in dry THF (0.1 M) under nitrogen atmosphere and cooled to −78 °C. Lithium diisopropylamide (LDA, 1.2 equiv.) was added and the mixture was stirred for 1 h. The bromide (1.0 equiv.) was added, and the solution was stirred overnight at rt. The reaction was quenched with NH_4Cl, extracted with *n*-pentane two times, and washed with brine. The combined organic phases were dried over Na_2SO_4 and concentrated to give the crude mixture that was purified by column chromatography to give the desired product.

General Procedure C: Synthesis of alkenoic acids

Following literature procedure[17,57]: To a solution of malonic acid (2.20 equiv.) in DMSO (0.17 M), piperidine (0.0200 equiv.) and acetic acid (0.0200 equiv.) were added dropwise. The solution was heated to 65 °C, aldehyde (1.00 equiv.) was added dropwise, and the mixture is stirred at 75 °C for an appropriate amount of time. The reaction was quenched with water and Et_2O. The layers were separated, and the aqueous phase was extracted with Et_2O three times. The combined organic layers were washed with water five and with brine one time. The combined organic phases were dried over Na_2SO_4 and concentrated under reduced pressure to give the crude mixture that was purified by column chromatography to get the desired product.

3-(4-Chlorophenyl)propanal,[58] (*E*)-5-(4-chlorophenyl)pent-3-enoic acid(**6d**),[57] and (*E*)-5-(4-(methoxycarbonyl)phenyl)pent-3-enoic acid (**6e**)[57] were synthesised according to literature.

Cyclohex-2-en-1-yl(phenyl)selane (2a)

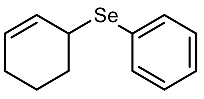

Following General Procedure A: Diphenyl diselenide (**1a**) (3.41 g, 10.9 mmol, 0.500 equiv.), dry EtOH (20 mL, 1.0 M), $NaBH_4$ (1.08 g, 28.6 mmol, 1.31 equiv.), 3-Bromocyclohex-1-ene (2.6 mL, 22 mmol, 1.0 equiv.), 24 h; eluting with pure *n*-pentane to *n*-pentane/EA 9:1; the desired product **2a** was isolated as a yellow oil (5.12 g, 21.6 mmol, 99%).

R*f* = 0.74 (*n*-pentane); **^{1}H NMR** (300 MHz, $CDCl_3$): δ [ppm] = 7.64–7.46 (m, 2H), 7.33–7.13 (m, 3H), 5.93–5.80 (m, 1H), 5.81–5.69 (m, 1H), 4.03–3.92 (m, 1H), 2.11–1.81 (m, 5H), 1.70–1.55 (m, 1H); **^{13}C NMR** (101 MHz, $CDCl_3$): δ [ppm] = 134.1, 130.7, 129.8, 129.0, 127.8, 127.3, 41.2, 29.4, 24.9, 19.7; **^{77}Se NMR** (76 MHz, $CDCl_3$): δ [ppm] = 394.9; **HRMS** (EI-MS) [$C_{12}H_{14}Se$]: ($[M]^{+\bullet}$), obs.: 238.0252, calcd.: 238.0255; **IR** (ATR, neat): $\tilde{\nu}$ [cm^{-1}] = 3056, 3056, 3027, 2930, 2863, 1580, 1476, 1439, 1252, 1178, 1070, 1021, 999, 865, 738, 693.

(Cyclohex-2-en-1-yl-1-*d*)(phenyl)selane (2a-1*d*)

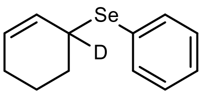

A dried Schlenk flask was loaded with THF (80 mL, 0.11 M) and cooled to –78°C in an acetone bath. LDA (5.7 mL, 1.8 M in THF, 10 mmol, 1.2 equiv.) and KO*t*Bu (1.18 g, 10.5 mmol, 1.22 equiv.) were added and the solution was stirred for 1 h at −78°C. At this temperature, **2a** (2.00 g, 8.43 mmol, 1.00 equiv.) was added. The red solution was quenched with D_2O (5.1 mL, 0.25 mol, 30 equiv.) after 1 h at −78 °C, allowed to warm to rt and starred at this temperature for 18 h. After drying over $MgSO_4$ the yellow solution was concentrated and purified by column chromatography (*n*-pentane) to yield 1.82 g (7.66 mmol, 91%) of (cyclohex-2-en-1-yl-1-*d*)(phenyl)selane (70%-D, 30% of **2a** left in the mixture) as a yellow oil.

R*f* = 0.74 (*n*-pentane); **^{1}H NMR** (300 MHz, $CDCl_3$): δ [ppm] = 7.62–7.52 (m, 2H), 7.32–7.22 (m, 3H), 5.92–5.83 (m, 1H), 5.81–5.66 (m, 1H), 4.02–3.89 (m, 0.3H), 2.12–2.04 (m, 2H), 2.06–1.83 (m, 3H), 1.70–1.56 (m, 1H); **^{13}C NMR** (101 MHz, $CDCl_3$): δ [ppm] = 134.1, 129.8, 129.7 (**2a**), 129.0, 127.7 (**2a**), 127.7, 127.2, 41.2, 29.4 (**2a**), 29.3, 24.9, 19.7 (**2a**), 19.6; **^{77}Se NMR** (76 MHz, $CHCl_3$): δ [ppm] = 403.4; **^{2}H NMR** (61 MHz, $CHCl_3$): δ [ppm] = 3.98; **HRMS** (EI-MS) [$C_{12}H_{13}SeD$]: ($[M]^{+\bullet}$), obs.: 239.0324, calcd.: 239.0318; **IR** (ATR, neat): $\tilde{\nu}$ [cm^{-1}] = 3056, 3023, 2926, 2859, 1685, 1577, 1476, 1439, 1252, 1178, 1066, 902, 734, 690.

11,11'-Diselanediylbis(undecan-1-ol) (1g)

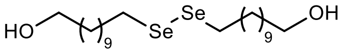

Following literature procedure:[2] Sodium borohydride (0.480 g, 12.7 mmol, 1.00 equiv.) was dissolved in H_2O (2 mL, 6 M), the solution was added to elementary selenium (1.00 g, 12.7 mmol, 1.00 equiv.) suspended in H_2O (2 mL, 6 M) at 0 °C. After the initial vigorous reaction had subsided the mixture was stirred for 15 min and then refluxed briefly to complete the dissolution of the selenium. The brownish red aqueous solution of NaSeSeNa was then added to a THF solution (4 mL, 6 M) of 11-bromoundecanol (5.73 g, 22.8 mmol, 1.80 equiv.) and reacted at 50 °C overnight. The crude mixture was purified by column chromatography with eluent DCM/EA 4:1 to give the desired product as a yellow solid (1.80 g, 3.60 mmol, 57%).

R*f* = 0.24 (DCM/EA 4:1); **mp**: 79.1 °C; **^{1}H NMR** (300 MHz, $CDCl_3$): *δ* [ppm] = 3.64 (t, *J* = 6.6 Hz, 2H), 2.96–2.80 (m, 2H), 1.72 (p, *J* = 7.3 Hz, 2H), 1.57 (p, *J* = 6.9 Hz, 2H), 1.45–1.17 (m, 16H); **^{13}C NMR** (101 MHz, $CDCl_3$): *δ* [ppm] = 63.1, 32.8, 31.0, 30.3, 29.7, 29.5, 29.5, 29.4, 29.1, 25.7; **^{77}Se NMR** (76 MHz, $CDCl_3$): *δ* [ppm] = 306.3; **HRMS** (ESI-MS) [$C_{22}H_{46}O_2Se_2$]: ([M+H]$^+$), obs.: 503.1904, calcd.: 503.1901; **IR** (ATR, neat): $\tilde{\nu}$ [cm^{-1}] = 3433, 3370, 2922, 2851, 1741, 1469, 1364, 1215, 1059.

(*E*)-Cinnamyl(phenyl)selane/ (*E*)-phenyl(3-phenylprop-2-en-1-yl)selane (2g)

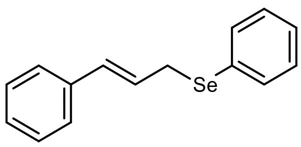

Following General Procedure A: Diphenyl diselenide (**1a**) (8.74 g, 28.0 mmol, 0.500 equiv.) dry EtOH (60 mL, 1.0 M), $NaBH_4$ (2.99 g, 79.0 mmol, 1.41 equiv.), (*E*)-cinnamylbromide/ (*E*)-(3-bromo-prop-1-en-1-yl)benzene (11.1 g, 56.2 mmol, 1.00 equiv.); 24 h; eluting with pure *n*-pentane to *n*-pentane/EA 9:1; isolated yield: 12.2 g, 44.5 mmol, 80%, colourless solid.

R*f* = 0.5 (PE/EA 19:1); **mp**: 64.1 °C; **^{1}H NMR** (300 MHz, $CDCl_3$): *δ* [ppm] = 7.55–7.47 (m, 2H), 7.33–7.14 (m, 8H), 6.42–6.25 (m, 1H), 6.24 (d, *J* = 15.7 Hz, 1H), 3.69 (d, *J* = 7.0 Hz, 2H); **^{13}C NMR** (101 MHz, $CDCl_3$): *δ* [ppm] = 136.9, 134.0, 132.1, 129.9, 129.0, 128.5, 127.5, 127.4, 126.3, 125.9, 30.7; **^{77}Se NMR** (76 MHz, $CDCl_3$): *δ* [ppm] = 341.1; **HRMS** (EI-MS) [$C_{15}H_{14}Se$]: ([M]$^{+\bullet}$), obs.: 274.0246, calcd.: 274.0261; **IR** (ATR, neat): $\tilde{\nu}$ [cm^{-1}] = 3466; 3056, 3027, 2930, 1946, 1874, 1800, 1744, 1677, 1576, 1476, 1439, 1178, 1122, 1074, 1021, 962, 734, 690.

(*E*)-phenyl(1-phenylhept-1-en-3-yl)selane (2b)

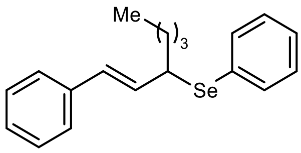

Following General Procedure B: (*E*)-Cinnamyl(phenyl)selane (**2g**) (4.01 g, 14.7 mmol, 1.00 equiv.), dry THF (0.15 L, 0.090 M), LDA (7.5 mL, 1.8 M, 18 mmol, 1.2 equiv.), 1-bromobutane (7.5 mL, 15 mmol, 1.0 equiv.), 24 h; eluting with pure *n*-pentane and *n*-pentane/Tol 10:1 twice; isolated yield: 2.04 g, 6.21 mmol, 42%, yellowish oil.

R*f* = 0.63 (*n*-pentane/Tol 10:1); **^{1}H NMR** (400 MHz, $CDCl_3$): *δ* [ppm] = 7.53 (dq, *J* = 6.6, 1.2 Hz, 2H), 7.34–7.13 (m, 8H), 6.16 (ddd, *J* = 15.7, 9.6, 0.8 Hz, 1H), 5.97 (d, *J* = 15.7 Hz, 1H), 3.91–3.78 (m, 1H), 1.92–1.69 (m, 2H), 1.48–1.17 (m, 4H), 0.89 (t, *J* = 7.2 Hz, 3H); **^{13}C NMR** (101 MHz, $CDCl_3$): *δ* [ppm] = 137.0, 136.2, 131.2, 129.9, 129.1, 128.7, 128.4, 127.8, 127.2, 126.2, 48.2, 34.7, 30.5, 22.4, 14.0; **^{77}Se NMR** (76 MHz, $CDCl_3$): *δ* [ppm] = 429.5; **HRMS** (EI-MS) [$C_{19}H_{22}Se$]: ([M]$^{+\bullet}$), obs.: 330.0875, calcd.: 330.0881; **IR** (ATR, neat): $\tilde{\nu}$ [cm^{-1}] = 3466, 3060, 3027, 2956, 2930, 2859, 1684, 1577, 1476, 1439, 1327, 1178, 1126, 1066, 1021, 969, 738, 690.

(*E*)-phenyl(1-phenyltridec-1-en-3-yl)selane (2c)

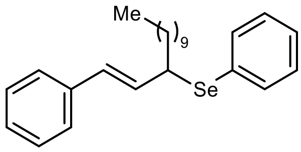

Following General Procedure B: (*E*)-Cinnamyl(phenyl)selane (**2g**) (2.19 g, 8.00 mmol, 1.00 equiv.), dry THF (80 mL, 0.1 M), lithium diisopropylamide (4.6 mL, 1.9 M, 8.8 mmol, 1.1 equiv.), 1-bromodecane (1.7 mL, 8.0 mmol, 1.0 equiv.), 17.5 h; eluting with pure *n*-pentane; isolated yield: 910 mg, 2.21 mmol, 28%, yellowish oil.

R*f* = 0.5 (*n*-pentane); **^{1}H NMR** (300 MHz, $CDCl_3$): δ [ppm] = 7.56–7.47 (m, 2H), 7.34–7.12 (m, 8H), 6.15 (dd, *J* = 15.7, 9.5 Hz, 1H), 5.95 (d, *J* = 15.7 Hz, 1H), 3.85 (td, *J* = 9.0, 6.0 Hz, 1H), 1.91–1.65 (m, 2H), 1.51–1.34 (m, 2H), 1.36–1.18 (m, 16H), 0.93–0.82 (m, 3H); **^{13}C NMR** (101 MHz, $CDCl_3$): δ [ppm] = 137.0, 136.2, 131.2, 129.9, 129.1, 128.7, 128.4, 127.8, 127.2, 126.2, 48.3, 34.9, 31.9, 29.6, 29.5, 29.3, 29.3, 22.7, 14.1; **HRMS** (EI-MS) [$C_{25}H_{34}Se$]: ([M]$^{+\bullet}$), obs.: 414.1831, calcd.: 414.1820; **IR** (ATR, neat): $\tilde{\nu}$ [cm^{-1}] = 3056, 3027, 2922, 2851, 1942, 1797, 1737, 1580, 1461, 1070, 1021, 958, 738, 690.

***tert*-Butyl(phenyl)selane (2d)**

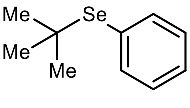

Following literature procedure:[59] 2-Bromo-2-methylpropane (2.2 mL, 20 mmol 1.0 equiv.), zinc (0.664 g, 10.2 mmol, 0.510 equiv.) and diphenyl diselenide (**1a**) (2.89 g, 9.26 mmol, 0.463 equiv.) were dissolved in DCM (100 mL, 0.2 M) and stirred at rt for 7 h. The reaction was quenched with HCl solution (1 M), extracted with DCM, and washed with HCl solution one and brine two times. The organic phases were dried over Na_2SO_4 and concentrated to give the crude mixture that was purified by column chromatography using *n*-pentane/THF 19:1 to give the desired product as a yellow oil (1.73 g, 8.12 mmol, 41%).

R*f* = 0.5 (*n*-hexane); **^{1}H NMR** (300 MHz, $CDCl_3$): δ [ppm] = 7.68–7.61 (m, 2H), 7.40–7.21 (m, 3H), 1.41 (s, 9H); **^{13}C NMR** (75 MHz, $CDCl_3$): δ [ppm] = 138.3, 128.7, 128.5, 128.5, 43.0, 32.3; **^{77}Se NMR** (76 MHz, $CDCl_3$): δ [ppm] = 522.8; **HRMS** (EI-MS) [$C_{10}H_{14}Se$]: ([M]$^{+\bullet}$), obs.: 214.0255, calcd.: 214.0255; **IR** (ATR, neat): $\tilde{\nu}$ [cm^{-1}] = 3056, 2956, 2889, 2859, 1577, 1476, 1435, 1301, 1152, 1066, 1021, 738, 693.

Adamantan-1-yl(phenyl)selane (2e)

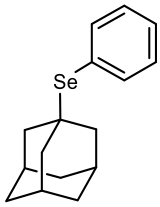

Following literature procedure:[60] 1-Bromoadamantane (431 mg, 2.00 mmol, 1.00 equiv.), was dissolved in dry DCM (20 mL, 0.10 M) under nitrogen atmosphere. Indium (142 mg, 1.24 mmol, 0.620 equiv.), and diphenyl diselenide (**1a**) (313 mg, 1.00 mmol, 0.500 equiv.), was added and the mixture was refluxed for 2.5 h. The reaction was quenched with HCl solution (1 M), extracted with Et_2O, and washed with HCl solution one and brine two times. The organic phases were dried over $MgSO_4$ and concentrated to give the crude mixture that was purified by column chromatography with eluent *n*-pentane to give the desired product as a yellow solid (551 mg, 1.89 mmol, 94%).

R_f = 0.3 (*n*-pentane), **mp**: 43.1 °C, **^{1}H NMR** (400 MHz, $CDCl_3$): δ [ppm] = 7.64–7.59 (m, 2H), 7.39–7.34 (m, 1H), 7.32–7.27 (m, 2H), 2.01–1.94 (m, 9H), 1.75–1.59 (m, 6H); **^{13}C NMR** (101 MHz, $CDCl_3$): δ [ppm] = 138.4, 128.5, 128.3, 126.4, 47.0, 44.7, 36.2, 30.7; **^{77}Se NMR** (76 MHz, $CDCl_3$): δ [ppm] = 528.8; **HRMS** (EI-MS) [$C_{16}H_{20}Se$]: ([M]$^{+\bullet}$), obs.: 292.0717, calcd.: 292.0725; **IR** (ATR, neat): $\tilde{\nu}$ [cm^{-1}] = 3056, 2904, 2848, 1476, 1454, 1342, 1297, 738, 693.

(*E*)-5-phenylpent-3-enoic acid (6a)

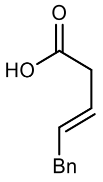

Following General Procedure C: Malonic acid (34.3 g, 330 mmol, 2.20 equiv.), DMSO 0.25 L, 0.60 M), piperidine (0.30 mL, 0.26 g, 3.0 mmol, 0.020 equiv.), acetic acid (0.18 mL, 0.19 mg, 3.1 mmol, 0.021 equiv.), 3-phenyl propanal (20 mL, 20 g, 0.15 mol, 1.0 equiv.), 5 h, eluting with PE/DCM 1:1 to PE/Et_2O 1:1; isolated yield: 16.1 g, 201 mmol, 61%, colourless oil.

The analytical data are in agreement with literature.[17]

R_f = 0.20 (PE/Et_2O 3:1); **^{1}H NMR** (300 MHz, $CDCl_3$): δ [ppm] = 7.33–7.27 (m, 2H), 7.24–7.10 (m, 3H), 5.77 (dtt, *J* = 14.7, 6.6, 1.3 Hz, 1H), 5.63 (dtt, *J* = 15.2, 6.9, 1.4 Hz, 1H), 3.40 (d, *J* = 6.6 Hz, 2H), 3.13 (dq, *J* = 6.8, 1.1 Hz, 2H); **^{13}C NMR** (101 MHz, $CDCl_3$): δ [ppm] = 177.7, 139.9, 133.9, 128.5, 128.5, 126.2, 122.3, 38.9, 37.6; **HRMS:** (EI-MS) [$C_{11}H_{12}O_2$] ([M]$^{+\bullet}$), obs.: 176.0829, calcd.: 176.0832; **IR** (ATR, neat): $\tilde{\nu}$ [cm^{-1}] = 701, 746, 932, 969, 1223, 1290, 1420, 1454, 1495, 1707, 2669, 2904, 3027;

(*E*)-5-(4-methoxyphenyl)pent-3-enoic acid (6b)

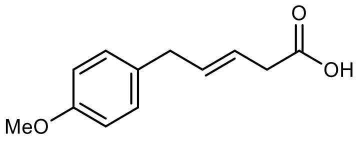

Following General Procedure C: Malonic acid (688 mg, 6.61 mmol, 2.20 equiv.), DMSO (18 mL, 0.17 M), piperidine (5.9 µL, 60 µmol, 0.020 equiv.), acetic acid (3.4 µL, 59 µmol, 0.020 equiv.), 3-(4-methoxyphenyl) propanal (493 mg, 3.00 mmol, 1.00 equiv.), 21.5 h, eluting with PE/Et_2O 1:1; isolated yield: 178 mg, 865 µmol, 29%, yellow oil.

The analytical data are in agreement with literature.[17]

R_f = 0.27 (PE/Et_2O 1:1); **^{1}H NMR** (300 MHz, $CDCl_3$): δ [ppm] = 7.12–7.08 (m, 2H), 6.87–6.82 (m, 2H), 5.80–5.68 (m, 1H), 5.65–5.53 (m, 1H), 3.79 (s, 3H), 3.33 (d, *J* = 6.5 Hz, 2H), 3.12 (d, *J* = 6.7 Hz, 2H); **^{13}C NMR** (75 MHz, $CDCl_3$): δ [ppm] = 178.1, 158.1, 134.3, 132.1, 129.6, 122.1, 114.0, 55.4, 38.1, 37.7; **HRMS:** (EI-MS) [$C_{12}H_{14}O_3$] ([M]$^{+\bullet}$), obs.: 206.0935, calcd.: 206.0938; **IR** (ATR, neat): $\tilde{\nu}$ [cm^{-1}] = 3034, 3001, 2937, 2837, 1707, 1610, 1584, 1513, 1461, 1420, 1301, 1245, 1178, 1111, 1036, 969, 816.

(*E*)-5-(4-(trifluoromethyl)phenyl)pent-3-enoic acid (6c)

Following General Procedure C: Malonic acid (581 mg, 5.58 mmol, 2.21 equiv.), DMSO (15 mL, 0.17 M), piperidine (4.9 µL, 50 µmol, 0.020 equiv.), acetic acid (3.0 µL, 52 µmol,

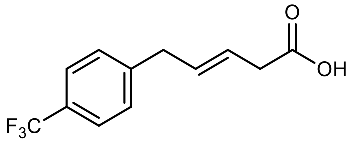

0.021 equiv.), 3-(4-(trifluoromethyl)phenyl) propanal (509 mg, 2.52 mmol, 1.00 equiv.), 20.5 h, eluting with PE/Et_2O 1:1; isolated yield: 289 mg, 1.19 mmol, 47%, white solid.

R*f* = 0.43 (PE/Et_2O 1:1); **mp**: 75.6 °C; **^{1}H NMR** (300 MHz, $CDCl_3$): δ [ppm] = 7.55 (d, *J* = 8.0 Hz, 2H), 7.30 (d, *J* = 8.0 Hz, 2H), 5.79–5.70 (m, 2H), 5.70–5.60 (m, 2H), 3.45 (d, *J* = 6.4 Hz, 2H), 3.14 (d, *J* = 6.7 Hz, 2H); **^{13}C NMR** (75 MHz, $CDCl_3$): δ [ppm] = 178.1, 144.1, 132.9, 129.0, 128.7 (q, *J* = 32.5 Hz, C–F), 125.5 (q, *J* = 3.8 Hz, C–F), 124.4 (q, *J* = 271.6 Hz, C–F), 123.4, 38.8, 37.7; **^{19}F NMR** (376 MHz, $CDCl_3$): δ [ppm] = −62.9; **HRMS:** (EI-MS) [$C_{12}H_{11}O_3F_3$] ($[M]^{+\bullet}$), obs.: 244.0706, calcd.: 244.0706; **IR** (ATR, neat): $\tilde{\nu}$ [cm^{-1}] = 2926, 2647, 2080, 1931, 1797, 1707, 1618, 1416, 1319, 1230, 1159, 1107, 1066, 1018, 969, 917, 842, 820, 745.

(*E*)-8-Chlorooct-3-enoic acid (6f)

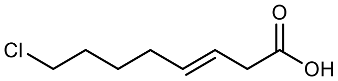

Following literature procedure for 6-chlorohexanal:[61] oxalyl dichloride (2.6 mL, 3.8 g, 30 mmol, 1.5 equiv.) in dry DCM (40 mL), N_2 atmosphere, DMSO (2.8 mL, 3.1 g, 40 mmol, 2.0 equiv.) in dry DCM (5 mL) , at -78 °C for 20 min, 6-chlorohexan-1-ol (2.73 g, 20.0 mmol. 1.00 equiv.) in dry DCM (5 mL), at -78 °C for 30 min, dry NEt_3 (8.4 mL, 6.1 g, 60 mmol, 3.0 equiv.), at -78 °C for 20 min and at r.t. overnight, eluting with *n*-pentane/EA 40:1 to 20:1.

According to literature procedure/[57]; Following literature procedure for (*E*)-8-Chlorooct-3-enoic acid:[61] Malonic acid (6.24 g, 60.0 mmol, 3.00 equiv.), DMSO (30 mL, 0.67 M), piperidine (99 µL, 85 mg, 1.0 mmol, 0.050 equiv.), acetic acid (57 µL, 60 mg, 1.0 mmol, 0.050 equiv.), 6-chlorohexanal (20.0 mmol, 1.00 equiv., from step before), 24 h, eluting with PE/Et_2O 1:1; isolated yield: 1.38 mg, 7.80 mmol, 39%, yellowish solid.

R*f* = 0.43 (PE/Et_2O 1:1); **mp**: 31.1 °C; **^{1}H NMR** (300 MHz, $CDCl_3$): δ [ppm] = 5.64–5.47 (m, 2H), 3.53 (t, *J* = 3.5 Hz, 2H), 3.11–3.05 (m, 2H), 2.13–2.04 (m, 2H), 1.83–1.72 (m, 2H), 1.59–1.47 (m, 2H); **^{13}C NMR** (75 MHz, $CDCl_3$): δ [ppm] = 178.4, 134.7, 121.6, 45.1, 37.8, 32.1, 31.8, 26.4; **HRMS:** (ESI-MS) [$C_8H_{13}O_2Cl$] ($[M+NH_4]^+$), obs.: 195.0975, calcd.: 195.0974; **IR** (ATR, neat): $\tilde{\nu}$ [cm^{-1}] = 2937, 2863, 1707, 1416, 1290, 1223, 969, 723.

***Iso*-Propyl isonicotinate (10)**

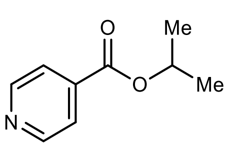

Following literature procedure:[62] 1,1'-Carbonyldiimidazole (CDI) (4.01 g, 24.7 mmol, 1.23 equiv.) was added portion wise to a mixture of isonicotinic acid (2.47 g, 20.1 mmol, 1.00 equiv.) in DCM (10 mL, 2.0 M). The mixture was stirred until evolution of CO_2 ceased (as judged by bubble counter). *Iso*-propanol (7.8 mL, 0.10 mol, 5.0 equiv.) was added to the homogeneous mixture and the resulting solution was stirred for 24 h at rt. The solvent was removed under reduced pressure and the resulting crude product was purified by column chromatography with eluent PE/EA 1:1 to give the desired product as a yellow oil (3.01 g, 18.2 mmol, 91%).

$\mathbf{R_f}$ = 0.35 (PE/EA 1:1); **^{1}H NMR** (400 MHz, $CDCl_3$): δ [ppm] = 9.08–8.66 (m, 2H), 8.12–7.68 (m, 2H), 5.26 (hept, J = 6.3 Hz, 1H), 1.37 (d, J = 6.3 Hz, 6H); **^{13}C NMR** (101 MHz, $CDCl_3$): δ [ppm] = 164.6, 150.5, 138.1, 122.8, 69.5, 21.8; **HRMS** (EI-MS) [$C_9H_{11}NO_2$]: ([M]$^{+\bullet}$), obs.: 165.0785, calcd.: 165.0784; **IR** (ATR, neat): $\tilde{\nu}$ [cm^{-1}] = 3034, 2982, 2937, 1722, 1562, 1469, 1409, 1375, 1323, 1279, 1182, 1103, 992, 917, 854, 824, 760, 708, 675.

1-(1,3-Dioxoisoindolin-2-yl) 4-methyl bicyclo[2.2.2]octane-1,4-dicarboxylate (11)

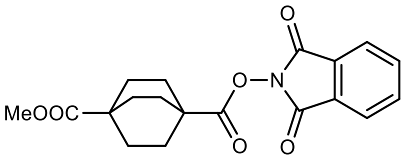

Following literature procedure:[63] 4-(Methoxycarbonyl) bicyclo[2.2.2]octane-1-carboxylic acid (334 mg, 1.57 mmol, 1.00 equiv.), 2-hydroxyisoindoline-1,3-dione (261 mg, 1.60 mmol, 1.02 equiv.) and *N*,*N*-dimethylpyridin-4-amine (21 mg, 0.17 mmol, 0.11 equiv.) were dissolved in DCM (7 mL, 0.2 M) and stirred at rt for 30 min. Diisopropylmethanediimine (0.25 mL, 1.6 mmol, 1.0 equiv.) was added dropwise and the mixture was stirred at rt for 24 h. The reaction was filtered through a fritted funnel and washed with DCM. The mixture was concentrated and purified by column chromatography with eluent PE/EA 9:1 to 2:1 to give the desired product as a colourless solid (484 mg, 1.36 mmol, 86%).

The analytical data are in agreement with literature.[63]

$\mathbf{R_f}$ = 0.39 (PE/EA 4:1); **mp**: 165.3 °C; **^{1}H NMR** (400 MHz, $CDCl_3$): δ [ppm] = 7.81 (dd, J = 5.5, 3.1 Hz, 2H), 7.71 (dd, J = 5.5, 3.1 Hz, 2H), 3.61 (s, 3H), 2.09–1.95 (m, 6H), 1.88–1.72 (m, 6H); **^{13}C NMR** (101 MHz, $CDCl_3$): δ [ppm] = 177.4, 173.3, 162.0, 134.7, 129.0, 123.9, 51.9, 38.5, 38.3, 27.6, 27.5; **HRMS** (ESI-MS) [$C_{19}H_{19}NO_6$]: ([M+H]$^+$), obs.: 358.1291, calcd.: 358.1285, ([M+Na]$^+$), obs.: 380.1105, calcd.: 380.1110, ([M+NH_4]$^+$), obs.: 375.1554, calcd.: 37.1551; **IR** (ATR, neat): $\tilde{\nu}$ [cm^{-1}] = 2960, 2878, 1808, 1782, 1722, 1610, 1461, 1435, 1357, 1252, 1182, 1141, 1073, 1025, 977, 939, 880, 853, 809, 761, 697.

Methyl 4-(phenylselanyl)bicyclo[2.2.2]octane-1-carboxylate (2f)

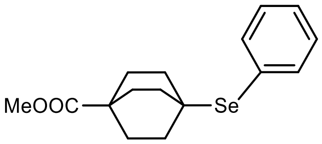

Following literature procedure:[64] Reaction was carried out in heat-dried glassware under nitrogen atmosphere. 1-(1,3-Dioxoisoindolin-2-yl) 4-methyl bicyclo[2.2.2]octane-1,4-dicarboxylate (**11**) (1.21 g, 3.40 mmol, 1.00 equiv.), diphenyl diselenide (**1a**) (2.12 g, 6.79 mmol, 1.97 equiv.) 4,4,4',4',5,5,5',5'-octamethyl-2,2'-bi(1,3,2-dioxaborolane) (1.61 g, 6.27 mmol, 1.84 equiv.) and *iso*-propyl isonicotinate (**10**) (0.196 g, 1.19 mmol, 0.350 equiv.) were dissolved in dry trifluoro toluene (20 mL, 0.17 M) and stirred at 100 °C for one day. The solvent was removed under reduced pressure and the resulting crude product was purified by column chromatography with eluent PE/EA 9:1 to give the desired product as a yellow solid (0.931 g, 2.88 mmol, 85%).

The analytical data are in agreement with literature.[64]

$\mathbf{R_f}$ = 0.75 (PE/EA 9:1); **mp**: 71.4 °C; **^{1}H NMR** (400 MHz, $CDCl_3$): δ [ppm] = 7.62–7.55 (m, 2H), 7.41–7.34 (m, 1H), 7.33–7.27 (m, 2H), 3.61 (s, 3H), 1.96–1.76 (m, 12H); **^{13}C NMR** (101 MHz, $CDCl_3$): δ [ppm] = 177.7, 138.3, 128.7, 128.5, 126.3, 51.7, 43.6, 37.9, 32.7, 30.0;

^{77}Se NMR (76 MHz, $CDCl_3$): δ [ppm] = 480.5; **HRMS** (EI-MS) [$C_{16}H_{20}O_2Se$]: ([M]$^{+\bullet}$), obs.: 324.0618, calcd.: 324.0630; **IR** (ATR, neat): $\tilde{\nu}$ [cm^{-1}] = 3056, 2952, 2919, 2870, 1730, 1580, 1457, 1331, 1245, 1074, 1014, 910, 738, 693.

14 Synthesis and analytical data of lactonisation products

General procedure D: Hybrid Lactonisation

A photo reaction vial was charged with alkenoic acid **6a-f** (1.00 equiv.), diphenyl diselenide **1a** (0.050 equiv.) and HFIP (1.0 mL, 0.50 M). The reaction was stirred for a certain amount of time at 35 °C with an atmosphere of ambient air and irradiated with ultraviolet light (λ = 405 nm). The NMR standard 1,3-dinitrobenzene (0.500 equiv.) was added, and the mixture was concentrated under reduced pressure. The NMR yield and conversion was determined via ^{1}H NMR before the purification and isolation. Two outcomes were differentiated:
1) Crude mixtures with 100% conversion were purified by column chromatography to give the desired product.
2) Crude mixtures without 100% conversion were transferred with DCM for extraction. The crude mixture was washed with saturated $NaHCO_3$ solution three times and the combined aqueous phases were extracted with DCM one time. The combined organic phases were dried over Na_2SO_4, filtered, and concentrated under reduced pressure. The acid-free crude mixture was then purified by column chromatography to give the desired product.

5-Benzyl-4-(phenylselanyl)dihydrofuran-2(3*H*)-one (7a)

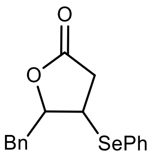

A photo reaction vial was charged with 5-phenylpent-3-enoic acid (**6a**) (88.9 mg, 0.504 mmol, 1.00 equiv.), diphenyl diselenide (**1a**) (158 mg, 0.506 mmol, 1.00 equiv.), TFA (20 µL, 0.31 mmol, 0.50 equiv.) and HFIP (1 mL, 0.5 M). The reaction was stirred for 24 h at 19 °C with an atmosphere of ambient air and irradiated with blue light (447 nm). The NMR standard 1,3-dinitrobenzene was added, and the solvent was removed under reduced pressure. NMR yield: 100%, isolated yield: 134 mg, 0.405 mmol, 80%, yellow solid.

R_f = 0.13 (PE/EA 9:1); **mp**: 66.7 °C; **^{1}H NMR** (300 MHz, $CDCl_3$): δ [ppm] = 7.56–7.51 (m, 2H), 7.43–7.23 (m, 6H), 7.17–7.11 (m, 2H), 4.64 (td, *J* = 6.6, 4.4 Hz, 1H), 3.53 (td, *J* = 8.5, 7.0 Hz, 1H), 3.10 (dd, *J* = 14.5, 4.4 Hz, 1H), 2.92 (dd, *J* = 14.5, 6.4 Hz, 1H), 2.75 (dd, *J* = 18.0, 8.5 Hz, 1H), 2.50 (dd, *J* = 18.1, 8.6 Hz, 1H); **^{13}C NMR** (101 MHz, $CDCl_3$): δ [ppm] = 174.5, 136.2, 135.4, 129.6, 129.6, 129.1, 128.7, 127.1, 125.9, 85.8, 39.1, 37.7, 36.1; **^{77}Se NMR** (76 MHz, $CDCl_3$): δ [ppm] = 372.0; **HRMS** (ESI-MS) [$C_{17}H_{16}O_2Se$]: ([M+H]$^{+}$), obs.: 333.0391, calcd.: 333.0388, ([M+NH_4]$^{+}$), obs.: 350.0656, calcd.: 350.0654; **IR** (ATR, neat): $\tilde{\nu}$ [cm^{-1}] = 3060, 3027, 2919, 1774, 1580, 1476, 1439, 1349, 1279, 1200, 1159, 1077, 917, 738, 693.

5-Benzylfuran-2(5*H*)-one (8a)

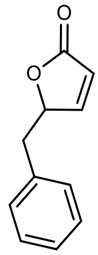

Following General Procedure D: (*E*)-5-Phenylpent-3-enoic acid (**6a**) (86.2 mg, 489 µmol, 1.00 equiv.), diphenyl diselenide (**1a**) (7.7 mg, 25 µmol, 0.051 equiv.), HFIP (1.0 mL, 0.49 M), 24 h, 35 °C, λ = 405 nm; 1,3-dinitrobenzene (37.1 mg, 221 µmol, 0.446 equiv.); NMR yield: 73%, conversion: 100%; eluting with PE/Et_2O 2:1 to 1:1; isolated yield: 57.0 mg, 327 µmol, 67%, colourless, viscose liquid.

$\mathbf{R}_f$ = 0.16 (PE/Et_2O 2:1); **^{1}H NMR** (300 MHz, $CDCl_3$): δ [ppm] = 7.40 (dd, *J* = 5.7, 1.5 Hz, 1H), 7.36–7.24 (m, 3H), 7.23–7.19 (m, 2H), 6.07 (dd, *J* = 5.7, 2.0 Hz, 1H), 5.23 (ddt, *J* = 7.0, 6.4, 1.7 Hz, 1H), 3.15 (dd, *J* = 13.8, 6.4 Hz, 1H), 2.96 (dd, *J* = 13.8, 7.0 Hz, 1H); **^{13}C NMR** (75 MHz, $CDCl_3$): δ [ppm] = 172.9, 155.7, 134.9, 129.5, 128.8, 127.4, 122.2, 83.5, 39.7; **HRMS** (EI-MS) [$C_{11}H_{10}O_2$]: ([M]$^{+\bullet}$), obs.: 174.0675, calcd.: 174.0675; **IR** (ATR, neat): $\tilde{\nu}$ [cm^{-1}] = 3086, 3030, 2926, 1744, 1603, 1495, 1454, 1338, 1256, 1159, 1100, 1021, 980, 924, 749, 701.

5-(4-Methoxybenzyl)furan-2(5*H*)-one (8b)

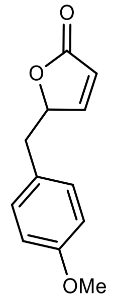

Following General Procedure D: (*E*)-5-(4-Methoxyphenyl)pent-3-enoic acid (**6b**) (105 mg, 507 µmol, 1.00 equiv.), diphenyl diselenide (**1a**) (8.0 mg, 26 µmol, 0.051 equiv.), HFIP (1.0 mL, 0.51 M), 24 h, 35 °C, λ = 405 nm; 1,3-dinitrobenzene (36.9 mg, 219 µmol, 0.432 equiv.); NMR yield: 51%, conversion: 83%; eluting with PE/Et_2O 2:1 to 1:1; isolated yield: 45.3 mg, 222 µmol, 44%, colourless liquid.

$\mathbf{R}_f$ = 0.05 (PE/Et_2O 2:1); **^{1}H NMR** (300 MHz, $CDCl_3$): δ [ppm] = 7.39 (dd, *J* = 5.7, 1.5 Hz, 1H), 7.15–7.08 (m, 2H), 6.88–6.81 (m, 2H), 6.06 (dd, *J* = 5.7, 2.0 Hz, 1H), 5.18 (tt, *J* = 6.3, 1.7 Hz, 1H), 3.78 (s, 3H), 3.08 (dd, *J* = 14.0, 6.2 Hz, 1H), 2.91 (dd, *J* = 14.0, 7.0 Hz, 1H); **^{13}C NMR** (101 MHz, $CDCl_3$): δ [ppm] = 172.9, 158.9, 155.8, 130.5, 126.8, 122.1, 114.2, 83.7, 55.4, 38.6; **HRMS** (EI-MS) [$C_{12}H_{12}O_3$]: ([M]$^{+\bullet}$), obs.: 204.0784, calcd.: 204.0781; **IR** (ATR, neat): $\tilde{\nu}$ [cm^{-1}] = 3086, 3001, 2933, 2837, 1744, 1610, 1513, 1461, 1301, 1245, 1163, 1100, 1029, 980, 917, 813, 749, 701, 671.

5-(4-(Trifluoromethyl)benzyl)furna-2(5*H*)-one (8c)

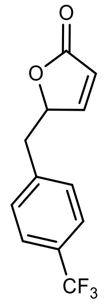

Following General Procedure D: (*E*)-5-(4-(Trifluoromethyl)phenyl)pent-3-enoic acid (**6c**) (122 mg, 498 µmol, 1.00 equiv.), diphenyl diselenide (**1a**) (7.8 mg, 25 µmol, 0.050 equiv.), HFIP (1.0 mL, 0.50 M), 24 h, 35 °C, λ = 405 nm; 1,3-dinitrobenzene (36.4 mg, 217 µmol, 0.436 equiv.); NMR yield: 76%, conversion: 100%; eluting with PE/Et_2O 2:1 to 1:1; isolated yield: 85.6 mg, 353 µmol, 71%, colourless liquid.

$\mathbf{R}_f$ = 0.02 (PE/Et_2O 2:1); **^{1}H NMR** (300 MHz, $CDCl_3$): δ [ppm] = 7.58 (d, *J* = 8.0 Hz, 2H), 7.41 (dd, *J* = 5.7, 1.5 Hz, 1H), 7.34 (d, *J* = 8.0 Hz, 2H), 6.10 (dd, *J* = 5.7, 2.0 Hz, 1H), 5.26 (tt, *J* = 6.4, 1.8 Hz, 1H), 3.12 (d, *J* = 6.4 Hz, 2H); **^{13}C NMR** (101 MHz, $CDCl_3$): δ [ppm] =172.5, 155.1, 139.1, 129.9 , 129.8 (q, *J* = 32.3 Hz, C–F), 125.8 (q, *J* = 3.8 Hz, C–F), 123.9 (q, *J* = 272.2 Hz, C–F), 82.8, 39.4; **^{19}F NMR** (376 MHz, $CDCl_3$): δ [ppm] = 63.1; **HRMS** (EI-MS) [$C_{12}H_9O_2F_3$]: ([M]$^{+\bullet}$), obs.: 242.0548, calcd.: 242.0549; **IR** (ATR, neat):

ṽ [cm^{-1}] = 3094, 2930, 1756, 1621, 1420, 1323, 1260, 1159, 1111, 1066, 1021, 984, 921, 846, 813, 738, 705, 671.

5-(4-Chlorobenzyl)furan-2(5*H*)-one (8d)

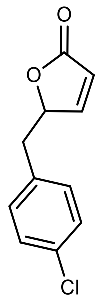

Following General Procedure D: (*E*)-5-(4-chlorophenyl)pent-3-enoic acid (**6d**) (105 mg, 501 µmol, 1.00 equiv.), diphenyl diselenide (**1a**) (8.0 mg, 25 µmol, 0.050 equiv.), HFIP (1.0 mL, 0.50 M), 48 h, 35 °C, λ = 405 nm; 1,3-dinitrobenzene (34.0 mg, 202 µmol, 0.403 equiv.); NMR yield: 63%, conversion: 90% eluting with PE/Et_2O 2:1 to 1:1; isolated yield: 51.3 mg, 246 µmol, 49%, colourless liquid.

R*f* = 0.08 (PE/Et_2O 2:1); **^{1}H NMR** (300 MHz, $CDCl_3$): *δ* [ppm] = 7.39 (dd, *J* = 5.7, 1.5 Hz, 1H), 7.31–7.24 (m, 2H), 7.17–7.10 (m, 2H), 6.07 (dd, *J* = 5.7, 1.9 Hz, 1H), 5.21 (tt, *J* = 6.4, 1.8 Hz, 1H), 3.10–2.95 (m, 2H); **^{13}C NMR** (75 MHz, $CDCl_3$): *δ* [ppm] = 172.6, 155.3, 133.3, 133.3, 130.9, 128.9, 122.4, 83.0, 38.9; **HRMS** (EI-MS) [$C_{11}H_9O_2Cl$]: ([M]$^{+\bullet}$), obs.: 208.0281, calcd.: 208.0286; **IR** (ATR, neat): ṽ [cm^{-1}] = 3090, 2926, 1744, 1603, 1491, 1435, 1334, 1256, 1159, 1096, 1018, 917, 805, 708.

Methyl 4-((5-oco-2,5-dihydrofuran-2-yl)methyl)benzoate (8e)

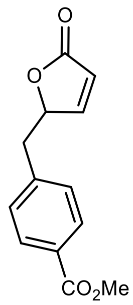

Following General Procedure D: (*E*)-5-(4-methoxycarbonyl)phenyl)pent-3-enoic aicd (**6e**) (117 mg, 501 µmol, 1.00 equiv.), diphenyl diselenide (**1a**) (7.8 mg, 25 µmol, 0.050 equiv.), HFIP (1.0 mL, 0.50 M), 48 h, 35 °C, λ = 405 nm; 1,3-dinitrobenzene (33.1 mg, 197 µmol, 0.393 equiv.); NMR yield: 71%, conversion: 100%; eluting with PE/Et_2O 1:2 to pure Et_2O; isolated yield: 70.7 mg, 304 µmol, 61%, white solid.

R*f* = 0.12 (PE/Et_2O 1:2); **mp**: 72.0 °C; **^{1}H NMR** (300 MHz, $CDCl_3$): *δ* [ppm] = 8.01–7.95 (m, 2H), 7.40 (dd, *J* = 5.7, 1.5 Hz, 1H), 7.32–7.27 (m, 2H), 6.08 (dd, *J* = 5.7, 2.0 Hz, 1H), 5.25 (tt, *J* = 6.4, 1.8 Hz, 1H) 3.90 (s, 3H), 3.19– 3.03 (m, 2H); **^{13}C NMR** (75 MHz, $CDCl_3$): *δ* [ppm] = 172.6, 166.9, 155.2, 140.2, 130.1, 129.6, 129.4, 122.5, 82.9, 52.3, 39.6; **HRMS** (ESI-MS) [$C_{13}H_{12}O_4$]: ([M+H]$^+$), obs.: 233.0805, calcd.: 233.0808, ([M+NH_4]$^+$), obs.: 250.1074, calcd.: 250.1074; **IR** (ATR, neat): ṽ [cm^{-1}] = 3094, 3004, 2952, 1752, 1715, 1614, 1577, 1513, 1435, 1279, 1163, 1103, 1021, 965, 921, 865, 820, 760, 708.

5-(4-Chlorobutyl)furan-2(5*H*)-one (8f)

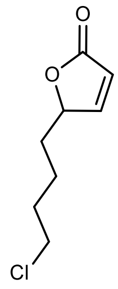

Following General Procedure D: (*E*)-8-Chlorooct-3-enoic acid (**6f**) (86.7 mg, 491 µmol, 1.00 equiv.), diphenyl diselenide (**1a**) (7.7 mg, 25 µmol, 0.051 equiv.), HFIP (1.0 mL, 0.49 M), 48 h, 35 °C, λ = 405 nm; 1,3-dinitrobenzene (34.1 mg, 203 µmol, 0.413 equiv.); NMR yield: 80%, conversion: 94%; eluting with PE/Et_2O 2:1 to 1:1; isolated yield: 59.0 mg, 338 µmol, 69%, colourless liquid.

R*f* = 0.15 (PE/Et_2O 1:1); **^{1}H NMR** (400 MHz, $CDCl_3$): *δ* [ppm] = 7.45 (dd, *J* = 5.7, 1.5 Hz, 1H), 6.13 (dd, *J* = 5.7, 2.0 Hz, 1H), 5.09-5.01 (m, 1H), 3.54 (t, *J* = 6.5 Hz, 2H), 1.90-1.78 (m, 3H), 1.75-1.55 (m, 3H); **^{13}C NMR** (75 MHz, $CDCl_3$): *δ* [ppm] = 173.1, 156.1, 121.9, 83.1, 44.6, 32.5, 32.2, 22.5; **HRMS** (EI-MS) [$C_8H_{11}O_2Cl$]: ([M]$^{+\bullet}$),

obs.: 174.0440, calcd.: 174.0442; **IR** (ATR, neat): $\tilde{\nu}$ [cm^{-1}] = 3094, 2945, 2870, 1744, 1603, 1457, 1331, 1163, 1107, 1014, 902, 861, 813, 742, 708.

15 Synthesis and analytical data of substitution products

General Procedure E: Light-stimulated amination reaction

A photo reaction vial was charged with the selenide (1.00 equiv.), the nucleophile (3.00 equiv.), TFA (0.5 equiv.) and HFIP (0.50 M). The reaction was stirred for a certain amount of time at 19 °C with an air-balloon while irradiated with green light (λ = 528 nm). The NMR standard 1,3-dinitrobenzene was added, and the mixture was concentrated. The crude mixture was purified by column chromatography to give the desired product.

2-(Cyclohex-2-en-1-yl)-2*H*-benzo[*d*][1,2,3]triazole (4aa N^2), 1-(Cyclohex-2-en-1-yl)-1*H*-benzo[*d*][1,2,3]triazole (4aa N^1)

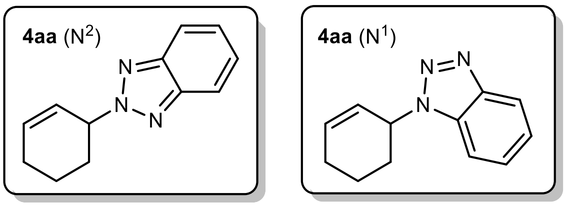

2 : 1

Following General Procedure E: Cyclohex-2-en-1-yl(phenyl)selane (**2a**) (239 mg, 1.01 mmol, 1.00 equiv.), 1*H*-benzotriazole (**3a**) (360 mg, 3.02 mmol, 2.99 equiv.), TFA (0.04 mL, 0.5 mmol, 0.5 equiv.), 5 h; eluting with PE /Tol 4:1 to pure Tol to Tol/EA 9:1; NMR yield: 59% (**N^2**) and 25% (**N^1**) (2.4:1.0); isolated yield **A**: 107 mg, 0.538 mmol, 53%, yellow oil; **B**: 60.4 mg, 0.303 mmol, 30%, yellow oil; ratio isomers in isolated compounds: N^2/N^1 = 1.8:1.0

R*f* = **N^2:** 0.5 (PE/EA 9:1), **N^1:** 0.19 (PE/EA 9:1); **^{1}H NMR** (400 MHz, $CDCl_3$): δ [ppm] = **N^2:** 7.92–7.83 (m, 2H), 7.40–7.33 (m, 2H), 6.18 (dtd, *J* = 9.8, 3.7, 2.1 Hz, 1H), 6.03–5.92 (m, 1H), 5.54 (ddp, *J* = 8.0, 5.3, 2.6 Hz, 1H), 2.38–2.10 (m, 4H), 2.04–1.88 (m, 1H), 1.85–1.70 (m, 1H); **B:** 8.06 (dt, *J* = 8.3, 1.0 Hz, 1H), 7.61 (dt, *J* = 8.4, 1.0 Hz, 1H), 7.43 (ddd, *J* = 8.3, 6.9, 1.1 Hz, 1H), 7.35 (ddd, *J* = 8.1, 6.9, 1.1 Hz, 1H), 6.23–6.15 (m, 1H), 5.93–5.82 (m, 1H), 5.62 (ddp, *J* = 8.5, 5.6, 2.7 Hz, 1H), 2.39–2.09 (m, 4H), 1.96 (ddt, *J* = 12.4, 9.7, 4.2 Hz, 1H), 1.90–1.77 (m, 1H); **^{13}C NMR** (101 MHz, $CDCl_3$): δ [ppm] = **N^2:** 144.2, 132.6, 126.1, 124.6, 118.1, 62.3, 30.3, 24.6, 19.8; **N^1:** 46.7, 132.9, 132.3, 126.9, 125.2, 123.8, 120.3, 110.7, 56.4, 29.8, 24.8, 20.7; **HRMS** (EI-MS) [$C_{12}H_{13}N_3$]: ([M]$^{+\bullet}$), **N^2:** obs.: 199.1101, calcd.: 199.1104, **N^1:** obs.: 199.1099, calcd.: 199.1104; **IR** (ATR, neat): $\tilde{\nu}$ [cm^{-1}] = **N^2:** 3034, 2930, 2866, 1566, 1446, 1316, 1271, 1208, 977, 887, 854, 813, 746; **N^1:** 3034, 2937, 2870, 2840, 2363, 1491, 1454, 1271, 1159, 1096, 794, 746.

2-(Cyclohex-2-en-1-yl)-5-methyl-2*H*-benzo[*d*][1,2,3]triazole (4ab N²), 1-(Cyclohex-2-en-1-yl)-5-methyl-1*H*-benzo[*d*][1,2,3]triazole (4ab N³), 1-(Cyclohex-2-en-1-yl) -6-methyl-1*H*-benzo[*d*] [1,2,3]triazole (4ab N¹)

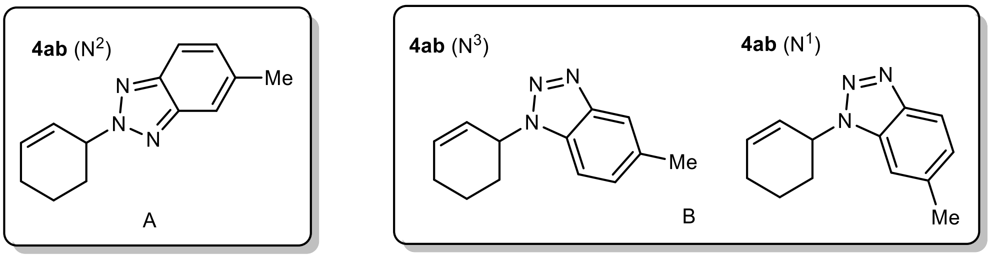

2.3 : 1.0

Following General Procedure E: Cyclohex-2-en-1-yl(phenyl)selane (**2a**) (243 mg, 1.02 mmol, 1.00 equiv.), 5-methyl-1*H*-benzo[*d*][1,2,3]triazole (**3b**) (456 mg, 3.42 mmol, 3.35 equiv.), TFA (0.04 mL, 0.5 mmol, 0.5 equiv.), 5 h, eluting **A** & **B** 1st column with PE/Tol 4:1 to pure Tol to Tol/EA 9:1 and **B** 2nd column with PE/EA 9:1; NMR yield: 55% (**A**) and 24% (**B**) (2.3:1.0, isomeric ratio ($N^2/N^3/N^1$): 5.8:1.5:1.0); isolated yield: **A**: 88.5 mg, 0.415 mmol, 41%, yellow oil; **B**: 48.4 mg, 0.227 mmol, 22%; yellow oil; ratio isomers in isolated compounds: $N^2/N^3/N^1$ = 5.0:1.6:1.0.

R_f = **A:** 0.5 (pure Tol), **B:** 0.25 (pure Tol); **^{1}H NMR** (400 MHz, $CDCl_3$): δ [ppm] = **A:** 7.76 (dd, *J* = 8.8, 0.8 Hz, 1H), 7.61 (q, *J* = 1.1 Hz, 1H), 7.20 (dd, *J* = 8.8, 1.5 Hz, 1H), 6.16 (dtd, *J* = 9.8, 3.7, 2.1 Hz, 1H), 6.01–5.93 (m, 1H), 5.50 (ddp, *J* = 8.0, 5.3, 2.6 Hz, 1H), 2.48 (d, *J* = 1.0 Hz, 3H), 2.37–2.08 (m, 4H), 2.01–1.88 (m, 1H), 1.84–1.69 (m, 1H); **B:** 7.95 (d, *J* = 8.6 Hz, 1H, **N¹**), 7.87 (dt, *J* = 1.7, 1.0 Hz, 2H, **N³**), 7.52 (d, *J* = 8.6 Hz, 2H, **N³**), 7.37 (q, *J* = 1.1 Hz, 1H, **N¹**), 7.30 (dd, *J* = 8.5, 1.4 Hz, 2H, **N³**), 7.21 (dd, *J* = 8.6, 1.4 Hz, 1H, **N¹**), 6.20 (dtd, *J* = 10.2, 4.7, 2.7 Hz, 3H, **N³+N¹**), 5.94–5.80 (m, 3H, **N³+N¹**), 5.66–5.52 (m, 3H, **N³+N¹**), 2.53 (s, 3H, **N¹**), 2.52 (s, 6H, **N³**), 2.37–2.17 (m, 8H, **N³+N¹**), 2.21–2.06 (m, 3H, **N³+N¹**), 2.03–1.88 (m, 3H, **N³+N¹**), 1.89–1.76 (m, 3H, **N³+N¹**); **^{13}C NMR** (101 MHz, $CDCl_3$): δ [ppm] = **A:** 144.7, 142.8, 136.2, 132.5, 129.0, 124.7, 117.5, 116.3, 62.1, 30.3, 24.6, 22.1, 19.8; **B:** 147.1 (**N³**), 145.2 (**N¹**), 137.3 (**N³+ N¹**), 133.7 (**N³**), 132.7 (**N³**), 132.6 (**N¹**), 130.7 (**N¹**), 129.0 (**N³**), 126.0 (**N¹**), 125.2 (**N¹**), 125.1 (**N³**), 119.5 (**N¹**), 119.0 (**N³**), 110.0 (**N³**), 109.6 (**N¹**), 56.2 (**N³**), 56.1, 29.7 (**N³**), 29.6 (**N¹**), 24.6 (**N³+ N¹**), 22.1 (**N¹**), 21.5 (**N³**), 20.6 (**N¹**), 20.6 (**N³**); **HRMS** (EI-MS) [$C_{13}H_{15}N_3$]: ([M]$^{+\bullet}$), **A:** obs.: 213.1257, calcd.: 213.1261, **B:** obs.: 213.1268, calcd.: 213.1261; **IR** (ATR, neat): $\tilde{\nu}$ [cm^{-1}] = **A:** 3034, 2922, 2866, 2837, 1744, 1722, 1655, 1629, 1562, 1446, 1398, 1327, 1267, 1208, 1156, 980, 887, 857, 798, 727; **B:** 3030, 2937, 2863, 1655, 1621, 1588, 1498, 1450, 1398, 1316, 1275, 1230, 1163, 1115, 954, 891, 850, 798, 749.

5-Chloro-2-(cyclohex-2-en-1-yl)-2*H*-benzo[*d*][1,2,3]triazole (4ac N^2), 5-Chloro-1-(cyclohex-2-en-1-yl)-1*H*-benzo[*d*][1,2,3]triazole (4ac N^3), 6-Chloro-1-(cyclohex-2-en-1-yl)-1*H*-benzo[*d*][1,2,3]triazole (4ac N^1)

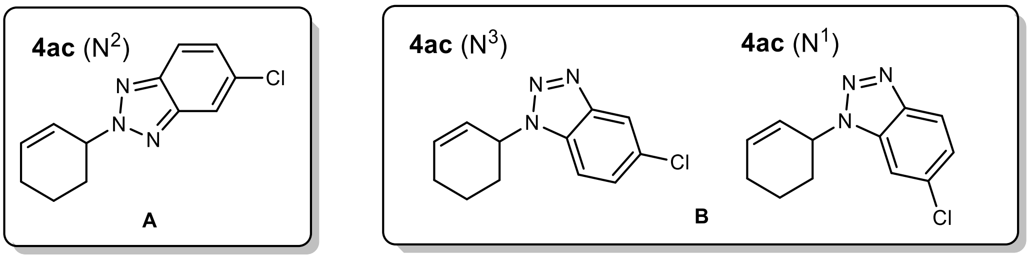

1.2 : 1.0

Following General Procedure E: Cyclohex-2-en-1-yl(phenyl)selane (**2a**) (242 mg, 1.02 mmol, 1.00 equiv.), 5-chloro-1*H*-benzo[*d*][1,2,3]triazole (**3c**) (469 mg, 3.05 mmol, 2.99 equiv.), TFA (0.04 mL, 0.5 mmol, 0.5 equiv.), 3 h; eluting **A & B** 1st column with PE/Tol 4:1 to pure Tol to Tol/EA 19:1 and **B** 2nd column with PE/EA 9:1; NMR yield: 41% (**A**) and 35% (**B**) (1.2:1.0, isomeric ratio ($N^2/N^3/N^1$): 2.4:1.0:1.0); isolated yield: **A**: 31.7 mg, 0.136 mmol, 13% in clean fraction; 76.8 mg, 0.329 mmol, 32% in mixed fraction with NMR standard 1,3-dinitrobenzene; yield total: 109 mg, 0.470 mmol, 45%, yellow oil; **B**: 69.0 mg, 0.295 mmol, 29%, yellow oil; ratio isomers in isolated compounds: $N^2/N^3/N^1$= 3.43:1.25:1.0.

R_f = **A:** 0.38 (pure Tol), **B:** 0.13 (pure Tol); **^{1}H NMR** (400 MHz, $CDCl_3$): δ [ppm] = **A:** 7.86 (dd, J = 1.9, 0.8 Hz, 1H), 7.81 (dd, J = 9.0, 0.7 Hz, 1H), 7.31 (dd, J = 9.0, 1.9 Hz, 1H), 6.17 (dtd, J = 9.8, 3.7, 2.1 Hz, 1H), 5.96 (dq, J = 10.0, 2.5 Hz, 1H), 5.51 (ddp, J = 7.9, 5.3, 2.6 Hz, 1H), 2.38–2.08 (m, 4H), 2.01–1.86 (m, 1H), 1.83–1.70 (m, 1H); **B:** 8.04 (dd, J = 1.9, 0.7 Hz, 1H, **N^3**), 7.98 (dd, J = 8.8, 0.6 Hz, 1H, **N^1**), 7.61 (dd, J = 1.8, 0.6 Hz, 1H, **N^1**), 7.55 (dd, J = 8.8, 0.7 Hz, 1H, **N^3**), 7.39 (dd, J = 8.8, 1.8 Hz, 1H, **N^3**), 7.31 (dd, J = 8.8, 1.8 Hz, 1H, **N^1**), 6.21 (ddq, J = 10.0, 4.7, 2.4 Hz, 2H, **N^3+N^1**), 5.87 (dd, J = 10.1, 2.5 Hz, 2H, **N^3+N^1**), 5.60 (tdq, J = 11.0, 5.4, 2.6 Hz, 2H, **N^3+N^1**), 2.40–2.16 (m, 6H, **N^3+N^1**), 2.17–2.02 (m, 2H, **N^3+N^1**), 1.95 (dtt, J = 13.1, 9.3, 5.2 Hz, 2H, **N^3+N^1**), 1.89–1.76 (m, 2H, **N^3+N^1**); **^{13}C NMR** (101 MHz, $CDCl_3$): δ [ppm] = **A:** 144.6, 142.8, 133.0, 132.1, 127.8, 124.4, 119.4, 117.3, 62.6, 30.4, 24.7, 19.8; **B:** 147.2 (**N^1**), 145.1 (**N^3**), 133.4 (**N^1**), 133.3 (**N^3**), 133.2 (**N^1**), 130.9 (**N^3**), 129.6 (**N^3**), 127.8 (**N^3**), 126.7 (**N^1**), 125.0 (**N^1**), 124.6 (**N^3**), 124.6 (**N^1**), 121.0 (**N^3**), 119.4 (**N^1**), 111.5 (**N^3**), 110.3 (**N^1**), 56.5 (**N^1**), 56.5 (**N^3**), 29.8 (**N^1**), 29.7 (**N^3**), 24.6 (**N^3**), 24.6 (**N^1**), 20.5 (**N^1**), 20.4 (**N^3**); **HRMS** (EI-MS) [$C_{12}H_{12}N_3Cl$]: ([M]$^{+\bullet}$), **A:** obs.: 233.0710, calcd.: 233.0714, **B:** obs.: 233.0708, calcd.: 233.0714; **IR** (ATR, neat): $\tilde{\nu}$ [cm^{-1}] = **A:** 3071, 2941, 2866, 2840, 2359, 1785, 1730, 1618, 1558, 1476, 1443, 1323, 1267, 1234, 1141, 1051, 939, 887, 857, 805, 772, 731; **B:** 3034, 2937, 2866, 2836, 2363, 1722, 1655, 1610, 1472, 1275, 1215, 1144, 110, 1047, 880, 842, 809, 727.

2-(Cyclohex-2-en-1-yl)-2*H*-[1,2,3]triazolo[4,5-*b*]pyridine (4ad N^2), 3-(Cyclohex-2-en-1-yl)-3*H*-[1,2,3]triazolo[4,5-*b*]pyridine (4ac N^1), 1-(Cyclohex-2-en-1-yl)-1*H*-[1,2,3]triazolo [4,5-*b*]pyri-dine (4ad N^3)

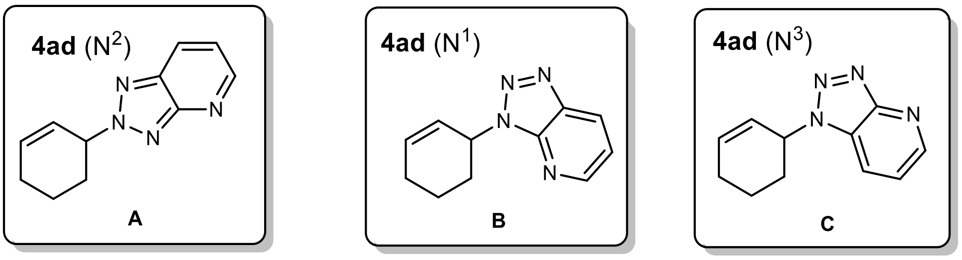

2.3 : 3.3 : 1.0

Following General Procedure E: Cyclohex-2-en-1-yl(phenyl)selane (**2a**) (241 mg, 1.01 mmol, 1.00 equiv.), 1*H*-[1,2,3]triazolo[4,5-*b*]pyridine (**3d**) (359 mg, 2.99 mmol, 2.96 equiv.), TFA (0.04 mL, 0.5 mmol, 0.5 equiv.), 3 h; eluting **A & B & C** 1st column with PE/Tol 4:1 to pure Tol to Tol/Et_2O 3:1 and **C** 2nd column with PE/Tol 4:1 to pure Tol to Tol/Et_2O 2:1 and **C** with PE/EA 4:1 to 1:2; NMR-yield: 28% (**A**), 40% (**B**) and 12% (**C**) (2.3:3.3:1.0); isolated yield **A**: 47.7 mg, 0.238 mmol, 24% in clean fraction; 10.0 mg, 49.9 µmol, 5% in mixed fraction with NMR-standard; yield total: 57.7 mg, 0.288 mmol, 29%, **B**: 65.6 mg, 0.328 mmol, 32% in clean fraction; 12.9 mg, 64.4 µmol, 6% in mixed fraction with NMR-standard; yield total: 77.3 mg, 0.386 mmol, 38%, **C:** 19.9 mg, 0.994 mmol, 10%; brown oil; ratio isomers in isolated compounds: N^2/N^1/N^3= 2.9:3.8:1.0.

R*f* = **A:** 0.38 (Tol/Et_2O 2:1), **B:** 0.43 (Tol/Et_2O 2:1), **C:** 0.08 (Tol/Et_2O 2:1); **^{1}H NMR** (400 MHz, $CDCl_3$): δ [ppm] = **A:** 8.80 (dd, *J* = 4.2, 1.6 Hz, 1H), 8.24 (dd, *J* = 8.5, 1.6 Hz, 1H), 7.34 (dd, *J* = 8.5, 4.2 Hz, 1H), 6.18 (dtd, *J* = 9.7, 3.8, 2.0 Hz, 1H), 5.99 (dq, *J* = 10.0, 2.5 Hz, 1H), 5.57 (ddq, *J* = 7.8, 5.2, 2.6 Hz, 1H), 2.42–2.21 (m, 3H), 2.22–2.10 (m, 1H), 2.04–1.90 (m, 1H), 1.86–1.71 (m, 1H); **B:** 8.68 (dd, *J* = 4.5, 1.5 Hz, 1H), 8.39 (dd, *J* = 8.3, 1.5 Hz, 1H), 7.35 (dd, *J* = 8.3, 4.5 Hz, 1H), 6.17 (dtd, *J* = 9.9, 3.7, 2.3 Hz, 1H), 5.90 (dq, *J* = 10.1, 2.5 Hz, 1H), 5.74 (tq, *J* = 7.8, 2.7 Hz, 1H), 2.40–2.24 (m, 3H), 2.24–2.13 (m, 1H), 2.08–1.93 (m, 1H), 1.90–1.76 (m, 1H); **C:** 8.74 (dd, *J* = 4.4, 1.5 Hz, 1H), 8.01 (dd, *J* = 8.4, 1.5 Hz, 1H), 7.38 (dd, *J* = 8.4, 4.4 Hz, 1H), 6.24 (dtd, *J* = 9.9, 3.8, 2.5 Hz, 1H), 5.95–5.84 (m, 1H), 5.67 (ddp, *J* = 8.4, 5.5, 2.7 Hz, 1H), 2.38–2.15 (m, 3H), 2.17–1.99 (m, 1H), 2.01–1.73 (m, 2H); **^{13}C NMR** (101 MHz, $CDCl_3$): δ [ppm] = **A:** 155.8, 151.7, 136.5, 133.2, 127.3, 124.3, 122.0, 63.2, 30.2, 24.7, 19.8; **B:** 150.0, 145.6, 137.3, 132.6, 128.7, 125.0, 119.9, 54.1, 29.7, 24.7, 20.3; **C:** 158.1, 148.2, 133.9, 124.7, 124.4, 121.7, 119.9, 57.3, 30.1, 24.7, 20.4; **HRMS** [$C_{11}H_{12}N_4$]: (EI-MS) ([M]$^{+\bullet}$), **A:** obs.: 200.1058, calcd.: 200.1057, **B:** obs.: 200.1062, calcd.: 200.1057, (ESI-MS) ([M+H]$^+$), **C:** obs.: 201.1136, calcd.: 201.1135; **IR** (ATR, neat): $\tilde{\nu}$ [cm^{-1}] = **A:** 3452, 3034, 2941, 2870, 2837, 2363, 1603, 1562, 1450, 1398, 1323, 1271, 887, 809, 731; **B:** 3034, 2937, 2866, 2837, 1588, 1428, 1226, 1148, 1096, 936, 805, 775, 727; **C:** 3448, 3064, 3034, 2937, 2870, 2837, 2363, 2337, 1718, 1655, 1599, 1413, 1312, 1275, 1141, 1081, 984, 876, 787.

2-(Cyclohex-2-en-1-yl)-2*H*-tetrazole (4ae N²), 1-(Cyclohex-2-en-1-yl)-1*H*-tetrazole (4ae N¹)

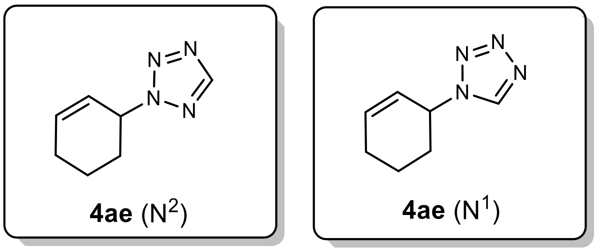

3.6 : 1.0

Following General Procedure E: Cyclohex-2-en-1-yl(phenyl)selane (**2a**) (243 mg, 1.02 mmol, 1.00 equiv.), 1*H*-tetrazole (**3e**) 213 mg, 3.04 mmol, 2.98 equiv.), TFA (0.04 mL, 0.5 mmol, 0.5 equiv.), 3 h; NMR yield: 68% (**N²**) & 19% (**N¹**) (3.6:1.0); eluting with Tol/EA 19:1 to 4:1, isolated yield: **N²**: 90.3 mg, 0.601 mmol, 59%, yellow oil; **N¹**: 12 mg, 0.076 mmol, 23%, yellow oil; ratio isomers in isolated compounds: N²/N¹ = 2.57:1.0.

R*f* = **N²:** 0.36 (Tol/EA 9:1), **N¹:** 0.17 (Tol/EA 9:1); **¹H NMR** (300 MHz, $CDCl_3$): δ [ppm] = **N²:** 8.44 (s, 1H), 6.08 (dtd, *J* = 9.7, 3.7, 2.0 Hz, 1H), 5.78 (dq, *J* = 10.1, 2.5 Hz, 1H), 5.43 (ddq, *J* = 10.2, 5.2, 2.6 Hz, 1H), 2.27–1.98 (m, 4H), 1.92–1.73 (m, 1H), 1.75–1.59 (m, 1H); **N¹:** 8.56 (s, 1H), 6.20 (dtd, *J* = 9.6, 3.8, 1.8 Hz, 1H), 5.77 (ddt, *J* = 10.0, 4.1, 2.2 Hz, 1H), 5.24 (tdq, *J* = 5.7, 4.0, 2.0 Hz, 1H), 2.25–1.90 (m, 4H), 1.78–1.63 (m, 1H), 1.60–1.44 (m, 1H); **¹³C NMR** (75 MHz, $CDCl_3$): δ [ppm] = **N²:** 152.6, 133.5, 123.1, 59.4, 29.3, 24.4, 19.2; **N¹:** 141.6, 135.7, 122.1, 54.5, 29.9, 24.5, 18.3; **HRMS** (ESI-MS) [$C_7H_{10}N_4$]: **N²:** ($[M+H]^+$) obs.: 151.0978, calcd.: 152.0978, ($[M+Na]^+$) obs.: 173.0796, calcd.: 173.0798, **N¹:** ($[M+H]^+$) obs.: 151.0980, calcd.: 151.0978, ($[M+Na]^+$) obs.: 173.0796, calcd.: 173.0798; **IR** (ATR, neat): $\tilde{\nu}$ [cm^{-1}] = **N²:** 3138, 3034, 2941, 2870, 2840, 1655, 1450, 1394, 1323, 1282, 1204, 1133, 1025, 932, 880, 839, 779, 731, 697; **N¹:** 3131, 3034, 2937, 2870, 1718, 1655, 1469, 1308, 1174, 1103, 969, 880, 775, 731, 678.

2-(Cyclohex-2-en-1-yl)-5-methyl-2*H*-tetrazole (4af N²), 1-(Cyclohex-2-en-1-yl)-5-methyl-1*H*-tetr-azole (4af N¹)

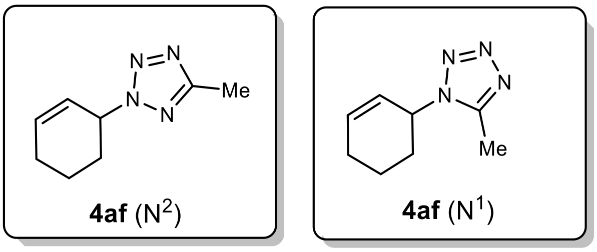

10.0 : 1.0

Following General Procedure E: Cyclohex-2-en-1-yl(phenyl)selane (**2a**) (234 mg, 0.988 mmol, 1.00 equiv.), 5-methyl-1*H*-tetrazole (**3f**) 251 mg, 2.96 mmol, 3.00 equiv.), TFA (0.04 mL, 0.5 mmol, 0.5 equiv.), 5 h; NMR yield: 85% (**N²**) & 10% (**N¹**) (8.5:1.0); eluting **N²** 1st column with PE/EA 9:1 and **N¹** 1st column with PE/EA 9:1 to pure EA, 2nd and 3rd column with pure DCM to pure EA, isolated yield: **N²**:125 mg, 0.758 mmol, 77%, yellow oil **N¹**: 12 mg, 0.076 mmol, 8%, colourless crystals; ratio isomers in isolated compounds: N²/N¹ = 9.6:1.0.

R*f* = **N²:** 0.4 (PE/EA 9:1), **N¹:** 0.1 (DCM); **¹H NMR** (300 MHz, $CDCl_3$): δ [ppm] = **N²:** 6.14 (dtd, *J* = 9.9, 3.8, 2.1 Hz, 1H), 5.83 (dq, *J* = 9.8, 2.5 Hz, 1H), 5.41 (ddp, *J* = 8.1, 5.5, 2.4 Hz, 1H), 2.53 (s, 3H), 2.33–2.02 (m, 4H), 2.00–1.80 (m, 1H), 1.83–1.63 (m, 1H); **¹H NMR** (400 MHz, $CDCl_3$): δ [ppm] = **N¹:** 6.20–6.03 (m, 1H), 5.71–5.53 (m, 1H), 5.11 (ddp, *J* = 8.3, 5.5, 2.6 Hz, 1H), 2.57 (s, 3H), 2.31–2.07 (m, 3H), 2.04–1.90 (m, 2H), 1.82–1.67 (m, 1H); **¹³C NMR** (75 MHz, $CDCl_3$): δ [ppm] = **N²:** 162.7, 133.3, 123.4, 59.2, 29.4, 24.5, 19.4, 11.0; **¹³C NMR** (101 MHz, $CDCl_3$): δ [ppm] = **N¹:** 150.8, 133.4, 123.5, 54.7, 29.5, 24.3, 20.0, 9.5;

HRMS (ESI-MS) [$C_8H_{12}N_4$]: **N²:** ([M+H]$^+$) obs.: 165.1134, calcd.: 165.1135, ([M+Na]$^+$) obs.: 187.0949, calcd.: 187.0954, **N¹:** ([M+H]$^+$) obs.: 165.1133, calcd.: 165.1135, ([M+Na]$^+$) obs.: 187.0951, calcd.: 187.0954; **IR** (ATR, neat): $\tilde{\nu}$ [cm^{-1}] = **N²:** 3034, 2941, 2870, 1655, 1506, 1450, 1387, 1342, 1170, 1033, 781, 880, 842, 783; **N¹:** 3034, 2937, 2870, 2870, 2870, 1521, 1431, 1402, 1271, 1118, 1006, 932, 839, 787, 734, 686.

1-(Cyclohex-2-en-1-yl)-1*H*-pyrazole-4-carbonitrile (4ag)

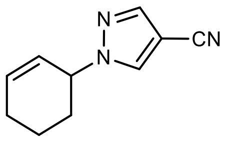

Following General Procedure E: Cyclohex-2-en-1-yl(phenyl)selane (**2a**) (249 mg, 1.05 mmol, 1.00 equiv.), *1H*-pyrazole-4-carbonitrile (**3g**) (286 mg, 3.08 mmol, 2.93 equiv.), TFA (0.04 mL, 0.5 mmol, 0.5 equiv.), 3 h; eluting with pure Tol to pure EA; NMR yield: 92%; isolated yield: 148 mg, 0.853 mmol, 81%, beige solid.

R*f* = 0.2 (PE/EA 9:1); **mp**: 56.0 °C; **¹H NMR** (300 MHz, $CDCl_3$): *δ* [ppm] = 7.86 (s, 1H), 7.81 (s, 1H), 6.19 (dtd, *J* = 9.7, 3.7, 1.8 Hz, 1H), 5.78 (ddt, *J* = 10.1, 4.2, 2.3 Hz, 1H), 4.94 (tdq, *J* = 5.8, 4.0, 2.1 Hz, 1H), 2.28–1.89 (m, 4H), 1.77–1.46 (m, 2H); **¹³C NMR** (75 MHz, $CDCl_3$): *δ* [ppm] = 142.3, 134.8, 133.1, 123.5, 113.7, 91.6, 57.8, 30.0, 24.7, 18.5; **HRMS** (EI-MS) [$C_{10}H_{11}N_3$]: ([M]$^{+\bullet}$), obs. 173.0949:, calcd.: 173.0948; **IR** (ATR, neat): $\tilde{\nu}$ [cm^{-1}] = 3124, 3034, 2937, 2866, 2233, 1655, 1539, 1443, 1383, 1316, 1223, 1129, 1103, 876, 775, 731.

4-Chloro-1-(cyclohex-2-en-1-yl)-1*H*-pyrazole (4ah)

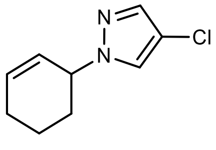

Following General Procedure E: Cyclohex-2-en-1-yl(phenyl)selane (**2a**) (254 mg, 1.07 mmol, 1.00 equiv.), 4-chloro-1*H*-pyrazole (**3h**) (331 mg, 3.23 mmol, 3.02 equiv.), TFA (0.04 mL, 0.5 mmol, 0.5 equiv.), 5 h; eluting 1st column with pure PE to PE/EA 19:1 and 2nd column with PE/EA 3:1; NMR yield: 61%; isolated yield: 95.0 mg, 0.522 mmol, 49%, beige solid.

R*f* = 0.63 (PE/EA 3:1); **mp**: 35.1 °C; **¹H NMR** (300 MHz, $CDCl_3$): *δ* [ppm] = 7.43 (s, 2H), 6.11 (dtd, *J* = 9.7, 3.7, 1.9 Hz, 1H), 5.78 (ddt, *J* = 10.1, 4.0, 2.3 Hz, 1H), 4.85 (dtd, *J* = 7.7, 3.5, 2.1 Hz, 1H), 2.28–1.96 (m, 3H), 2.02–1.84 (m, 1H), 1.73–1.61 (m, 2H); **¹³C NMR** (101 MHz, $CDCl_3$): *δ* [ppm] = 137.5, 133.4, 125.9, 124.7, 109.2, 57.8, 30.3, 24.8, 19.0; **HRMS** (ESI-MS) [$C_9H_{11}ClN_2$]: ([M+H]$^+$), obs.: 183.0683, calcd.: 183.0684; **IR** (ATR, neat): $\tilde{\nu}$ [cm^{-1}] = 3116, 3030, 2933, 2866, 2837, 1752, 1655, 1588, 1521, 1431, 1387, 1308, 1238, 1170, 969, 880, 835, 772.

1-(Cyclohex-2-en-1-yl)-3-phenyl-1*H*-pyrazole (4ai N²), 1-(Cyclohex-2-en-1-yl)-5-phenyl-1*H*-pyr-azole (4ai N¹)

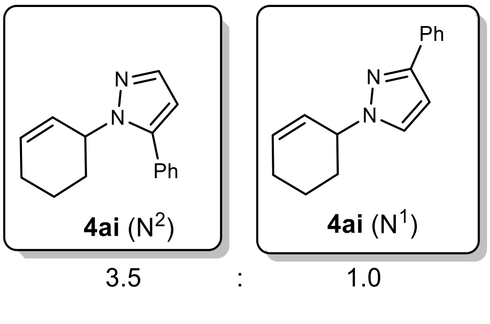

Following Procedure E: Cyclohex-2-en-1-yl(phenyl)selane (**2a**) (243 mg, 1.02 mmol, 1.00 equiv.), 5-phenyl-1*H*-pyrazole (**3i**) (438 mg, 3.04 mmol, 2.98 equiv.), TFA (0.04 mL, 0.5 mmol, 0.5 equiv.), 5 h; eluting **N²** & **N¹** with PE/Et_2O 1:1, **N²** & **N¹** with pure Tol to Tol/EA 9:1, **N¹** with PE/EA 9:1; NMR yield: 39% (**N²**) and 10% (**N¹**) (3.9:1.0); isolated

yield: **N^2**: 69.0 mg, 0.309 mmol, 30%, yellow oil and **N^1**: 20 mg, 0.089 mmol, 9%, yellow oil; ratio isomers in isolated compounds: N^2/ N^1 = 3.3:1.0.

R*f* = **N^2:** 0.2 (Tol), **N^1:** 0.34 (Tol); **^{1}H NMR** (400 MHz, $CDCl_3$): *δ* [ppm] = **N^2:** 7.57 (d, *J* = 1.8 Hz, 1H), 7.49–7.36 (m, 5H), 6.27 (d, *J* = 1.8 Hz, 1H), 5.97 (ddt, *J* = 10.4, 5.3, 3.0 Hz, 1H), 5.74–5.62 (m, 1H), 4.93 (ddp, *J* = 8.2, 5.3, 2.5 Hz, 1H), 2.29–2.16 (m, 1H), 2.17–2.08 (m, 1H), 2.10–1.83 (m, 3H), 1.72–1.51 (m, 1H); **N^1:** 7.84–7.77 (m, 2H), 7.48 (d, *J* = 2.3 Hz, 1H), 7.41–7.33 (m, 2H), 7.32–7.27 (m, 1H), 6.53 (d, *J* = 2.3 Hz, 1H), 6.11 (dtd, *J* = 9.7, 3.7, 2.0 Hz, 1H), 5.87 (ddt, *J* = 10.0, 3.9, 2.3 Hz, 1H), 4.99 (dtt, *J* = 5.7, 4.3, 2.2 Hz, 1H), 2.25–1.96 (m, 4H), 1.69 (p, *J* = 6.1 Hz, 2H); **^{13}C NMR** (101 MHz, $CDCl_3$): *δ* [ppm] = **N^2:** 143.2, 138.8, 131.2, 130.7, 129.2, 128.7, 128.5, 127.4, 106.0, 54.6, 30.5, 24.5, 20.9; **N^1**: 151.2, 133.8, 132.9, 128.9, 128.6, 127.4, 125.6, 125.4, 102.2, 57.2, 30.6, 24.9, 19.9; **HRMS** (EI-MS) [$C_{15}H_{16}N_2$]: ([M]$^{+\bullet}$), **N^2:** obs.: 224.1309, calcd.: 224.1308, **N^1:** obs.: 224.1305, calcd.: 224.1308; **IR** (ATR, neat): $\tilde{\nu}$ [cm^{-1}] = **N^2:** 3030, 2937, 2863, 2837, 1737, 1484, 1454, 1402, 1245, 1144, 1074, 977, 928, 783, 727; **N^1:** 3064, 3030, 2933, 2863, 1737, 1607, 1498, 1461, 1357, 1320, 1238, 1215, 1096, 1044, 880, 683, 753.

N-(Cyclohex-2-en-1-yl)aniline (4aj)

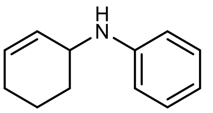

Following General Procedure E: Cyclohex-2-en-1-yl(phenyl)selane (**2a**) (240 mg, 1.00 mmol, 1.0 equiv.), aniline (**3j**) (0.28 mL, 3.0 mmol, 3.0 equiv.), TFA (0.04 mL, 0.5 mmol, 0.5 equiv.), 168 h; eluting with PE/EA gradient on puriFlash system; NMR yield: 82%; isolated yield: 92.0 mg, 0.532 mmol, 53%, yellowish oil.

R*f* = 0.65 (PE/Tol 1:1); **^{1}H NMR** (300 MHz, $CDCl_3$): *δ* [ppm] = 7.22–7.12 (m, 2H), 6.75–6.60 (m, 3H), 5.86 (dtd, *J* = 10.0, 3.5, 1.6 Hz, 1H), 5.80–5.69 (m, 1H), 4.05–3.92 (m, 1H), 2.04 (dddd, *J* = 8.3, 5.5, 3.2, 1.8 Hz, 2H), 1.96–1.81 (m, 1H), 1.79–1.55 (m, 3H); **^{13}C NMR** (75 MHz, $CDCl_3$): *δ* [ppm] = 147.2, 130.2, 129.3, 128.6, 117.2, 113.2, 47.9, 28.9, 25.2, 19.7; **HRMS** (EI-MS) [$C_{12}H_{15}N$]: ([M]$^{+\bullet}$), obs.: 173.1195, calcd.: 173.1199; **IR** (ATR, neat): $\tilde{\nu}$ [cm^{-1}] = 3407, 3053, 3023, 2926, 2863, 1603, 1502, 1431, 1312, 1245, 1182, 1103, 749, 693.

N-(Cyclohex-2-en-1-yl)-2,4,6-trimethylaniline (4ak)

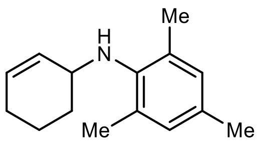

Following General Procedure E: Cyclohex-2-en-1-yl(phenyl)selane (**2a**) (238 mg, 1.00 mmol, 1.00 equiv.), 2,4,6-trimethylaniline (**3k**) (0.43 mL, 3.1 mmol, 3.1 equiv.), TFA (0.04 mL, 0.5 mmol, 0.5 equiv.), 72 h; eluting 1st column with PE/EA 2:1 and 2nd column with PE/EA 3:1; NMR yield: 78%; isolated yield: 170 mg, 0.791 mmol, 79%, dark red oil.

R*f* = 0.59 (PE/EA 9:1); **^{1}H NMR** (400 MHz, $CDCl_3$): *δ* [ppm] = 6.98 (td, *J* = 7.2, 1.7 Hz, 1H), 6.48–6.42 (m, 1H), 6.37 (d, *J* = 6.9 Hz, 2H), 5.77 (dtd, *J* = 10.0, 3.6, 1.7 Hz, 1H), 5.67 (ddt, *J* = 10.0, 3.7, 2.2 Hz, 1H), 3.91 (dh, *J* = 5.1, 2.6 Hz, 1H), 3.66 (s, 1H), 2.20 (s, 3H), 2.06–1.87 (m, 2H), 1.88–1.73 (m, 1H), 1.69–1.48 (m, 3H); **^{13}C NMR** (101 MHz, $CDCl_3$): *δ* [ppm] = 147.0, 139.1, 130.1, 129.2, 128.6, 118.3, 114.17, 110.5, 48.0, 28.9, 25.2, 21.6, 19.7; **HRMS** (EI-MS) [$C_{15}H_{21}N$]: ([M]$^{+\bullet}$), obs.: 215.1667, calcd.: 215.1669; **IR** (ATR, neat): $\tilde{\nu}$ [cm^{-1}] = 3370, 3019, 2922, 2855, 1484, 1446, 1372, 1300, 1230, 1156, 1081, 1033, 854, 723, 697.

N-(Cyclohex-2-en-1-yl)-3-methylaniline (4al)

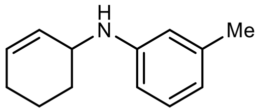

Following General Procedure E: Cyclohex-2-en-1-yl(phenyl)selane (**2a**) (237 mg, 1.00 mmol, 1.00 equiv.), *m*-toluidine (**3l**) (0.33 mL, 3.1 mmol, 3.1 equiv.), TFA (0.04 mL, 0.5 mmol, 0.5 equiv.), 72 h; eluting with PE/Tol 4:1 and second time with PE/EA 9:1; NMR yield: 60%; isolated yield: 57.1 mg, 0.305 mmol, 31%, brown oil.

R*f* = 0.59 (PE/EA 9:1); **^{1}H NMR** (400 MHz, $CDCl_3$): δ [ppm] = 6.98 (td, *J* = 7.2, 1.7 Hz, 1H), 6.48–6.42 (m, 1H), 6.37 (d, *J* = 6.9 Hz, 2H), 5.77 (dtd, *J* = 10.0, 3.6, 1.7 Hz, 1H), 5.67 (ddt, *J* = 10.0, 3.7, 2.2 Hz, 1H), 3.91 (dh, *J* = 5.1, 2.6 Hz, 1H), 3.66 (s, 1H), 2.20 (s, 3H), 2.06–1.87 (m, 2H), 1.88–1.73 (m, 1H), 1.69–1.48 (m, 3H); **^{13}C NMR** (101 MHz, $CDCl_3$): δ [ppm] = 147.0, 139.1, 130.1, 129.2, 128.6, 118.3, 114.17, 110.5, 48.0, 28.9, 25.2, 21.6, 19.7; **HRMS** (EI-MS) [$C_{13}H_{17}N$]: ([M]$^{+\bullet}$), obs.: 187.1350, calcd.: 187.1356; **IR** (ATR, neat): $\tilde{\nu}$ [cm^{-1}] = 3403, 3023, 2922, 2863, 2244, 1603, 1506, 1446, 1260, 1178, 1107, 910, 731, 693.

4-(Cyclohex-2-en-1-ylamino)benzonitrile (4am)

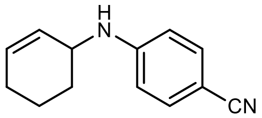

Following General Procedure E: Cyclohex-2-en-1-yl(phenyl)selane (**2a**) (237 mg, 1.00 mmol, 1.0 equiv.), 4-aminobenzonitrile (**3m**) (351 mg, 2.97 mmol, 2.97 equiv.), TFA (0.04 mL, 0.5 mmol, 0.5 equiv.), 72 h; eluting 1st column with PE/EA 9:1 and 2nd column with PE/Tol 1:1; NMR yield: 82%; isolated yield: 128 mg, 0.646 mmol, 65%, beige solid.

R*f* = 0.15 (PE/Tol 1:1); **mp**: 93.1 °C; **^{1}H NMR** (400 MHz, $CDCl_3$): δ [ppm] = 7.49–7.38 (m, 2H), 6.59 (d, *J* = 8.5 Hz, 2H), 5.94 (dtd, *J* = 9.4, 3.7, 1.7 Hz, 1H), 5.76–5.65 (m, 1H), 4.23 (s, 1H), 4.12–3.77 (m, 1H), 2.17–1.99 (m, 2H), 1.99–1.86 (m, 1H), 1.81–1.53 (m, 3H); **^{13}C NMR** (101 MHz, $CDCl_3$): δ [ppm] = 150.3, 133.8, 131.4, 127.0, 120.5, 112.5, 98.4, 47.4, 28.5, 25.0, 19.4; **HRMS** (EI-MS) [$C_{13}H_{14}N_2$]: ([M]$^{+\bullet}$), obs.: 198.1152, calcd.: 198.1152; **IR** (ATR, neat): $\tilde{\nu}$ [cm^{-1}] = 3358, 3027, 2933, 2863, 2210, 1603, 1521, 1338, 1260, 1170, 1096, 824.

Methyl 2-(cyclohex-2-en-1-ylamino)benzoate (4an)

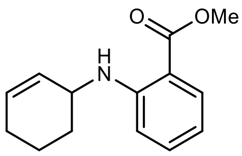

Following General Procedure E: Cyclohex-2-en-1-yl(phenyl)selane (**2a**) (240 mg, 1.00 mmol, 1.00 equiv.), methyl 2-aminobenzoate (**3n**) (0.40 mL, 3.1 mmol, 3.1 equiv.), TFA (0.04 mL, 0.5 mmol, 0.5 equiv.), 70 h; eluting with PE/EA gradient on puriFlash system; NMR yield: 66%; isolated yield: 120 mg, 0.518 mmol, 51%, brown oil.

R*f* = 0.8 (PE/EA 9:1); **^{1}H NMR** (400 MHz, $CDCl_3$): δ [ppm] = 7.93 (dd, *J* = 8.0, 1.7 Hz, 1H), 7.84 (s, 1H), 7.37 (ddd, *J* = 8.7, 7.0, 1.7 Hz, 1H), 6.79 (d, *J* = 8.5 Hz, 1H), 6.60 (ddd, *J* = 8.1, 7.1, 1.1 Hz, 1H), 5.91 (dtd, *J* = 9.9, 3.6, 1.7 Hz, 1H), 5.79 (ddt, *J* = 10.0, 3.9, 2.2 Hz, 1H), 4.11 (s, 1H), 3.87 (s, 3H), 2.24–1.87 (m, 3H), 1.84–1.60 (m, 3H); **^{13}C NMR** (101 MHz, $CDCl_3$): δ [ppm] = 169.0, 150.0, 134.5, 131.9, 130.5, 127.7, 114.5, 111.8, 110.2, 51.4, 47.1, 28.7, 25.1, 19.6; **HRMS** (EI-MS) [$C_{14}H_{17}NO_2$]: ([M]$^{+\bullet}$), obs.: 231.1249, calcd.: 231.1254; **IR** (ATR, neat):

$\tilde{\nu}$ [cm^{-1}] = 3351, 3079, 3023, 2937, 2863, 1684, 1606, 1580, 1513, 1457, 1312, 1234, 1103, 1077, 749.

_N_'-(Cyclohex-2-en-1-yl)benzohydrazide (4ao)

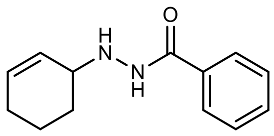

Following General Procedure E: Cyclohex-2-en-1-yl(phenyl)selane (**2a**) (241 mg, 1.02 mmol, 1.00 equiv.), benzohydrazide (**3o**) (415 mg, 3.05 mmol, 2.99 equiv.), TFA (0.04 mL, 0.5 mmol, 0.5 equiv.), 96 h; eluting 1st column with Tol/EA 4:1 and 2nd and 3rd column pure DCM to pure EA; NMR yield: 52%; isolated yield: 130 mg, 0.602 mmol, 59%, yellowish solid.

R*f* = 0.13 (PE/EA 3:1); **mp**: 126.2 °C; **^{1}H NMR** (400 MHz, $CDCl_3$): *δ* [ppm] = 7.87 (s, 1H), 7.63–7.53 (m, 2H), 7.39–7.31 (m, 1H), 7.30–7.17 (m, 2H), 5.71 (dtd, *J* = 9.5, 3.6, 1.7 Hz, 1H), 5.59–5.48 (m, 1H), 4.79 (s, 1H), 3.40 (tp, *J* = 4.9, 2.3 Hz, 1H), 1.94–1.73 (m, 2H), 1.64 (tdt, *J* = 15.3, 12.2, 4.3 Hz, 2H), 1.49–1.32 (m, 2H); **^{13}C NMR** (101 MHz, $CDCl_3$): 167.4, 132.8, 131.8, 131.3, 128.7, 126.9, 126.7, 55.8, 27.4, 25.3, 19.5; **HRMS** (ESI-MS) [$C_{13}H_{16}N_2O$]: ([M+H]$^+$), obs.: 217.1336, calcd.: 217.1335, ([M+Na]$^+$), obs.: 239.1158, calcd.: 239.1155; **IR** (ATR, neat): $\tilde{\nu}$ [cm^{-1}] = 3280, 3228, 3060, 3015, 2941, 2859, 1640, 1580, 1539, 1476, 1320, 1085, 913, 880, 798, 723, 690.

_N_'-(Cyclohex-2-en-1-yl)-4-methoxybenzohydrazide (4ap)

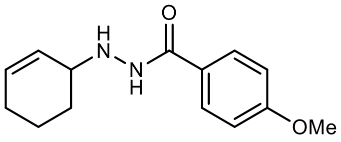

Following General Procedure E: Cyclohex-2-en-1-yl(phenyl)selane (**2a**) (234 mg, 0.99 mmol, 1.00 equiv.), 4-methoxybenzohydrazide (**3p**) (496 mg, 2.99 mmol, 3.02 equiv.), TFA (0.04 mL, 0.5 mmol, 0.5 equiv.), 24 h; eluting 1st column with Tol/EA 4:1 to Tol/EA 1:1 and 2nd and 3rd column pure DCM to pure EA; NMR yield: 50%; isolated yield: 109 mg, 0.444 mmol, 45%, colourless solid.

R*f* = 0.28 (Tol/EA 1:1); **mp**: 102.9 °C; **^{1}H NMR** (400 MHz, $CDCl_3$): *δ* [ppm] = 8.19 (s, 1H), 7.77–7.71 (m, 2H), 6.95–6.80 (m, 2H), 5.88–5.74 (m, 1H), 5.76–5.63 (m, 1H), 4.94 (s, 1H), 3.82 (d, *J* = 1.4 Hz, 3H), 3.54 (qd, *J* = 4.9, 2.1 Hz, 1H), 2.01–1.89 (m, 2H), 1.86–1.70 (m, 2H), 1.62–1.41 (m, 2H); **^{13}C NMR** (101 MHz, $CDCl_3$): 167.0, 162.4, 131.1, 128.8, 126.8, 125.1, 113.8, 55.8, 55.4, 27.4, 25.8, 19.5; **HRMS** (ESI-MS) [$C_{14}H_{18}N_2O_2$]: ([M+H]$^+$), obs.: 247.1435, calcd: 247.1441; **IR** (ATR, neat): $\tilde{\nu}$ [cm^{-1}] = 3273, 3068, 3019, 2933, 2837, 1607, 1510, 1457, 1305, 1252, 1182, 1029, 790, 727.

2-(Cyclohex-2-en-1-ylamino)isoindoline-1,3-dione (4aq)

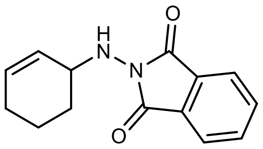

Following General Procedure E: Cyclohex-2-en-1-yl(phenyl)selane (**2a**) (253 mg, 1.07 mmol, 1.00 equiv.), *N*-aminophthalimide (**3q**) (563 mg, 3.47 mmol, 3.24 equiv.), TFA (0.04 mL, 0.5 mmol, 0.5 equiv.), 5 h; eluting with PE/EA 19:1 to PE/EA 5:1; NMR yield: 79%; isolated yield: 160 mg, 0.660 mmol, 62%, beige solid.

R$_f$ = 0.19 (PE/EA 5:1); **mp**: 85.3 °C; **^{1}H NMR** (300 MHz, $CDCl_3$): δ [ppm] = 7.90–7.81 (m, 2H), 7.77–7.68 (m, 2H), 5.92 (dtd, J = 10.2, 3.6, 1.8 Hz, 1H), 5.74 (dt, J = 10.0, 2.5 Hz, 1H), 3.75 (d, J = 6.5 Hz, 1H), 2.17–1.67 (m, 4H), 1.69–1.44 (m, 2H); **^{13}C NMR** (101 MHz, $CDCl_3$): δ [ppm] = 166.9, 134.2, 131.9, 130.3, 125.9, 123.4, 55.4, 27.5, 25.2, 19.1; **HRMS** (ESI-MS) [$C_{14}H_{14}N_2O_2$]: ([M+H]$^+$), obs.: 243.1132, calcd.: 243.1128, ([M+Na]$^+$), obs.: 265.0951, calcd.: 265.0947; **IR** (ATR, neat): $\tilde{\nu}$ [cm^{-1}] = 3291, 3027, 2937, 2866, 1782, 1718, 1379, 1122, 1070, 883, 712.

***N*-(Cyclohex-2-en-1-yl)-4-methylbenzenesulfonamide (4ar)**

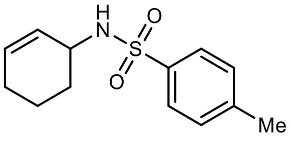

Following General Procedure E: Cyclohex-2-en-1-yl(phenyl)selane (**2a**) (240 mg, 1.01 mmol, 1.00 equiv.), 4-methylbenzenesulfonamide (**3r**) (519 mg, 3.03 mmol, 3.00 equiv.), TFA (0.04 mL, 0.5 mmol, 0.5 equiv.), 3 h; eluting with pure Tol to Tol/EA 9:1; NMR yield: 96%; isolated yield: 226 mg, 0.901 mmol, 89%, yellowish crystals.

R$_f$ = 0.41 (Tol/EA 9:1); **mp**: 104.0 °C; **^{1}H NMR** (400 MHz, $CDCl_3$): δ [ppm] = 7.79–7.73 (m, 2H), 7.34–7.28 (m, 2H), 5.77 (dtd, J = 9.6, 3.7, 1.8 Hz, 1H), 5.34 (ddt, J = 10.0, 4.0, 2.2 Hz, 1H), 4.37 (d, J = 8.7 Hz, 1H), 3.93–3.68 (m, 1H), 2.43 (s, 3H), 2.01–1.85 (m, 2H), 1.82–1.71 (m, 1H), 1.67–1.45 (m, 3H); **^{13}C NMR** (101 MHz, $CDCl_3$): δ [ppm] = 143.3, 138.4, 131.6, 129.7, 127.1, 127.0, 49.0, 30.3, 24.5, 21.6, 19.3; **HRMS** (ESI-MS) [$C_{13}H_{17}NO_2S$]: ([M+H]$^+$), obs.: 252.1056, calcd.: 252,1058, ([M+Na]$^+$), obs.: 274.0875, calcd.: 274.0878, ([M+NH_4]$^+$), obs.: 269.1321, calcd.: 269.1324; **IR** (ATR, neat): $\tilde{\nu}$ [cm^{-1}] = 3280, 3030, 2930, 2863, 2363, 1599, 1428, 1159, 1073, 1010, 887, 816, 667.

***N*-(Cyclohex-2-en-1-yl)-4-methoxybenzenesulfonamide (4as)**

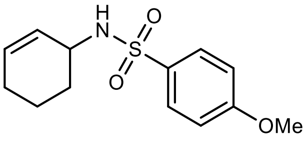

Following General Procedure E: Cyclohex-2-en-1-yl(phenyl)selane (**2a**) (241 mg, 1.01 mmol, 1.00 equiv.), 4-methoxybenzenesulfonamide (**3s**) (832 mg, 4.45 mmol, 4.41 equiv.), TFA (0.04 mL, 0.5 mmol, 0.5 equiv.), 4 h; eluting with PE/EA 4:1; NMR yield: 95%; isolated yield: 244 mg, 1.01 mmol, 90%, colourless crystals.

R$_f$ = 0.28 (PE/EA 4:1); **mp**: 94.1 °C; **^{1}H NMR** (400 MHz, $CDCl_3$): δ [ppm] = 7.96–7.73 (m, 2H), 7.03–6.90 (m, 2H), 5.77 (dtd, J = 9.5, 3.7, 1.8 Hz, 1H), 5.50–5.26 (m, 1H), 4.35 (d, J = 8.6 Hz, 1H), 3.81 (d, J = 7.0 Hz, 1H), 2.07–1.81 (m, 2H), 1.84–1.71 (m, 1H), 1.68–1.47 (m, 3H); **^{13}C NMR** (101 MHz, $CDCl_3$): δ [ppm] = 133.0, 131.6, 129.1, 127.1, 114.2, 55.6, 49.0, 30.3, 24.5, 19.3; **HRMS** (ESI-MS) [$C_{13}H_{17}NO_3S$]: ([M+H]$^+$), obs.: 268.1004, calcd.: 268.1002, ([M+Na]$^+$), obs.: 290.0824, calcd.: 290.0821; **IR** (ATR, neat): $\tilde{\nu}$ [cm^{-1}] = 3280, 3027, 2930, 2844, 1595, 1498, 1443, 1327, 1260, 1156, 1095, 1025, 939, 887, 835, 801, 731, 675.

_N_-(Cyclohex-2-en-1-yl)-_N_,4-dimethylbenzenesulfonamide (4at)

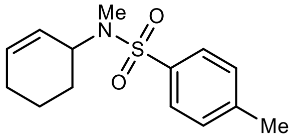

Following General Procedure E: Cyclohex-2-en-1-yl(phenyl)selane (**2a**) (249 mg, 1.05 mmol, 1.00 equiv.), *N*,4-dimethylbenzenesulfonamide (**3t**) (587 mg, 3.20 mmol, 3.04 equiv.), TFA (0.04 mL, 0.5 mmol, 0.5 equiv.), 4 h; eluting with PE/EA 4:1; NMR yield: 93%; isolated yield: 209 mg, 1.00 mmol, 79%, yellow oil.

R_f = 0.55 (PE/EA 4:1); **^{1}H NMR** (300 MHz, $CDCl_3$): δ [ppm] = 7.77–7.64 (m, 2H), 7.33–7.27 (m, 2H), 5.81 (dddd, J = 9.1, 6.5, 4.5, 1.9 Hz, 1H), 5.10 (dqd, J = 10.2, 2.2, 1.2 Hz, 1H), 4.57 (ddt, J = 10.5, 5.3, 2.6 Hz, 1H), 2.70 (s, 3H), 2.43 (s, 3H), 2.01–1.88 (m, 2H), 1.82–1.66 (m, 2H), 1.70–1.54 (m, 1H), 1.55–1.38 (m, 1H); **^{13}C NMR** (101 MHz, $CDCl_3$): δ [ppm] = 143.0, 137.3, 132.4, 129.7,, 127.2, 127.1, 54.3, 29.2, 26.8, 24.4, 21.5, 21.4; **HRMS** (ESI-MS) [$C_{14}H_{19}NO_2S$]: ([M+H]$^+$), obs.: 266.1211, calcd.: 266.1209, ([M+Na]$^+$), obs.: 288.1031, calcd.: 288.1029; **IR** (ATR, neat): $\tilde{\nu}$ [cm^{-1}] = 3027, 2937, 2866, 2356, 1651, 1599, 1454, 1338, 1167, 992, 958, 816, 775, 671.

_N_-((2'-(1-(Cyclohex-2-en-1-yl)-2_H_-tetrazol-5-yl)-[1,1'-biphenyl]-4-yl)methyl)-_N_-pentanoyl-_L_-valine (4au N^2), _N_-((2'-(2-(Cyclohex-2-en-1-yl)-2_H_-tetrazol-5-yl)-[1,1'-biphenyl]-4-yl) methyl)-_N_-pentanoyl-_L_-valine (B) (4au N^1)

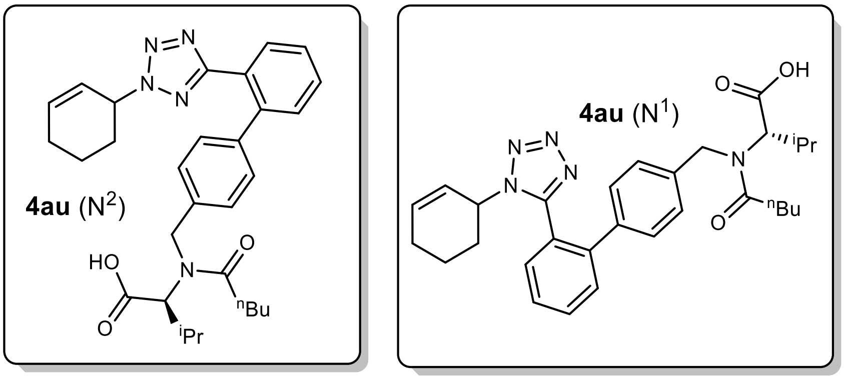

3.7 : 1

Following General Procedure E: Cyclohex-2-en-1-yl(phenyl)selane (**2a**) (242 mg, 1.02 mmol, 1.00 equiv.), *N*-((2'-(2*H*-tetrazol-5-yl)-[1,1'-biphenyl]-4-yl)methyl)-*N*-pentanoyl-*L*-valine (Valsartan, **3u**) (1.32 g, 3.03 mmol, 2.97 equiv.), TFA (0.04 mL, 0.5 mmol, 0.5 equiv.), 68 h; eluting 1st column with PE/EA 9:1 to PE/EA 1:1 and 2nd column with DCM/MeOH 19:1 to DCM/MeOH 9:1; NMR yield: **N^2**: 40 %, **N^1**: 7 % (5.7:1.0); isolated as isomeric mixture 252 mg, 0.490 mmol, 48% corresponds to isolated yield: **N^2**: 199 mg, 0.387 mmol, 38%, **N^1**: 53.0 mg, 0.103 mmol, 10%; colourless solid, ratio isomers in isolated compound: N^2/ N^1 = 3.8:1.0.

R_f = 0.8 (EA/MeOH 9:1); **mp**: 60.4 °C; **^{1}H NMR** (400 MHz, CDCl3): δ [ppm] = 7.82 (dd, J = 7.6, 1.5 Hz, 4H, **N^2**), 7.80–7.75 (m, 1H, **N^1**), 7.58–7.40 (m, 15H, **N^2 + N^1**), 7.19–6.97 (m, 20H, **N^2 + N^1**), 6.18–5.99 (m, 5H, **N^2 + N^1**), 5.89–5.72 (m, 5H, **N^2 + N^1**), 5.43–5.28 (m, 5H, **N^2 + N^1**), 5.17 (d, J = 15.4 Hz, 1H, **N^1**), 4.71 (d, J = 16.8 Hz, 4H, **N^2**), 4.45 (d, J = 16.8 Hz, 4H, **N^2**), 4.09 (d, J = 15.3 Hz, 1H, **N^1**), 3.99 (d, J = 10.9 Hz, 1H, **N^1**), 3.86 (dd, J = 10.8, 3.2 Hz, 4H, **N^2**), 2.72–2.56 (m, 5H, **N^2 + N^1**), 2.52–2.34 (m, 10H, **N^2 + N^1**), 2.27–1.98 (m, 15H, **N^2 + N^1**),

1.88–1.59 (m, 10H, **N^2 + N^1**), 1.36 (tq, *J* = 14.6, 7.3 Hz, 10H, **N^2 & N^1**), 1.07–0.81 (m, 45H, **N^2 + N^1**); **^{13}C NMR** (101 MHz, $CDCl_3$): 177.1 (**N^2**), 177.0 (**N^1**), 174.5 (**N^2**), 172.0 (**N^1**), 171.5 (**N^1**), 171.4 (**N^2**), 164.8 (**N^1**), 164.7 (**N^2**), 141.9 (**N^1**), 141.2 (**N^2**), 140.8 (**N^2**, 139.2 (**N^1**), 136.9 (**N^1**), 134.1 (**N^2**), 133.4 (**N^1**), 133.1 (**N^2**), 130.6 (**N^2**), 130.4 (**N^2**), 129.9 (**N^1**), 129.8 (**N^2**), 128.7 (**N^1**), 127.9 (**N^1**), 127.6 (**N^2**), 127.3 (**N^1**), 126.3 (**N^2**), 126.3 (**N^1**), 126.1 (**N^1**), 123.2 (**N^2**), 123.2 (**N^2**), 123.0 (**N^1**), 71.5 (**N^2**), 65.7 (**N^1**), 59.3 (**N^1**), 59.2 (**N^2**), 54.2 (**N^2**), 45.5 (**N^1**), 34.0 (**N^2**), 33.4 (**N^1**), 29.6 (**N^1**), 29.1 (**N^2**), 29.1 (**N^1**), 27.3 (**N^1**), 27.3 (**N^1**), 27.2 (**N^2**), 27.1 (**N^2**), 24.4 (**N^2**), 22.5 (**N^1**), 22.3 (**N^2**), 20.0 (**N^1**), 19.7 (**N^2**), 19.4 (**N^1**), 19.0 (**N^2**), 19.0 (**N^2**), 18.8 (**N^1**), 13.9 (**N^1**), 13.8 (**N^2**); **HRMS** (ESI-MS) [$C_{30}H_{37}N_5O_3$]: ([M]+H$^+$), obs.: 516.2974, calcd.: 516.2969, ([M]+Na$^+$), obs.: 538.2788, calcd.: 538.2789; **IR** (ATR, neat): $\tilde{\nu}$ [cm^{-1}] = 3034, 2960, 2933, 2874, 2248, 1730, 1651, 1607, 1469, 1200, 910, 760, 731.

_N_-(Cyclohex-2-en-1-yl)-4-(5-(_p_-tolyl)-3-(trifluoromethyl)-1_H_-pyrazol-1-yl)benzene sulfonamide (4av)

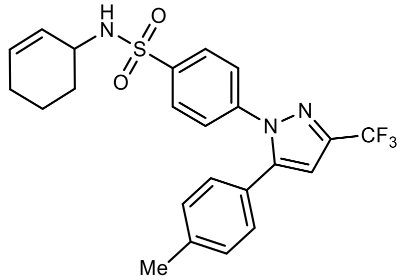

Following General Procedure E: Cyclohex-2-en-1-yl(phenyl)selane (**2a**) (123 mg, 0.517 mmol, 1.00 equiv.), 4-(5-(*p*-tolyl)-3-(trifluoromethyl)-1*H*-pyrazol-1-yl) benzene-sulfonamide (**3v**) (592 mg, 1.55 mmol, 3.0 equiv.), TFA (0.02 mL, 0.3 mmol, 0.6 equiv.), 5 h; eluting 1st column with Tol/EA gradient on puriFlash system and 2nd and 3rd column with pure Tol; NMR yield: 80%; isolated yield: 128 mg, 0.278 mmol, 54%, colourless crystals.

R*f* = 0.43 (Tol); **mp**: 140.1 °C; **^{1}H NMR** (400 MHz, $CDCl_3$): δ [ppm] = 7.98–7.78 (m, 2H), 7.52–7.45 (m, 2H), 7.19–7.13 (m, 2H), 7.11–7.05 (m, 2H), 6.74 (s, 1H), 5.79 (dtd, *J* = 9.6, 3.7, 1.8 Hz, 1H), 5.34 (ddt, *J* = 10.0, 4.1, 2.2 Hz, 1H), 4.55 (d, *J* = 8.7 Hz, 1H), 3.92–3.78 (m, 1H), 2.38 (s, 3H), 1.94 (tdt, *J* = 6.1, 3.9, 2.4 Hz, 2H), 1.81–1.71 (m, 1H), 1.64–1.40 (m, 3H); **^{13}C NMR** (101 MHz, $CDCl_3$): δ [ppm] = 145.6**,** 144.1 (q, *J* = 38.6 Hz), 142.3, 140.9, 139.7, 132.0, 129.7, 128.7, 127.9, 126.6, 125.7, 125.6, 121.0 (q, *J* = 269.1 Hz), 106.3–105.9 (m), 49.2, 30.2, 24.4, 21.3, 19.2; **^{19}F NMR** (377 MHz, $CDCl_3$): δ [ppm] = 62.9; **HRMS** (ESI-MS) [$C_{23}H_{22}F_3N_3O_2S$]: ([M+H]$^+$), obs.: 462.1458, calcd.: 462.1458; **IR** (ATR, neat): $\tilde{\nu}$ [cm^{-1}] = 3284, 3030, 2941, 1599, 1472, 1334, 1238, 1163, 1096, 977, 842, 809.

(_E_)-4-Methyl-_N_-(1-phenylhept-1-en-3-yl)benzenesulfonamide (4br)

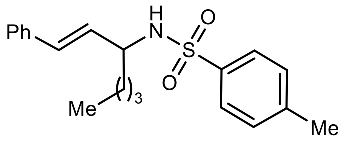

Following General Procedure E: (*E*)-Phenyl(1-phenylhept-1-en-3-yl)selane (**2b**) (328 mg, 1.00 mmol, 1.0 equiv.), 4-methylbenzenesulfonamide (**3r**) (523 mg, 3.06 mmol, 3.06 equiv.), TFA (0.04 mL, 0.5 mmol, 0.5 equiv.), 3 h; eluting with pure Tol to Tol/EA 9:1; NMR yield: 64%; isolated yield: 160 mg, 0.465 mmol, 47%, colourless crystals.

R*f* = 0.1 (Tol); **mp**: 101.2 °C; **^{1}H NMR** (400 MHz, $CDCl_3$): δ [ppm] = 7.77–7.66 (m, 2H), 7.31–7.14 (m, 5H), 7.13–7.06 (m, 2H), 6.21 (d, *J* = 15.9 Hz, 1H), 5.71 (dd, *J* = 15.9, 7.4 Hz, 1H), 4.43 (d, *J* = 7.7 Hz, 1H), 3.92 (p, *J* = 6.9 Hz, 1H), 2.31 (s, 3H), 1.68–1.48 (m, 2H),

1.37–1.17 (m, 4H), 0.87–0.74 (m, 3H).; **^{13}C NMR** (101 MHz, $CDCl_3$): δ [ppm] = 143.2, 138.2, 136.3, 131.4, 129.5, 129.0, 128.4, 127.6, 127.3, 126.3, 56.4, 35.7, 27.6, 22.3, 21.4, 13.9; **HRMS** (ESI-MS) [$C_{20}H_{25}NO_2S$]: ([M+H]$^+$), obs.: 344.1680, calcd.: 344.1679, ([M+Na]$^+$), obs.: 366.1503, calcd.: 366.1498, ([M+NH_4]$^+$), obs.: 361.1948, calcd.: 361.1944; **IR** (ATR, neat): $\tilde{\nu}$ [cm^{-1}] = 3269, 3060, 3027, 2956, 2863, 1599, 1495, 1450, 1323, 1159, 1096, 1040, 969, 813, 749, 667.

(*E*)-4-Methyl-*N*-(1-phenyltridec-1-en-3-yl)benzenesulfonamide (4cr)

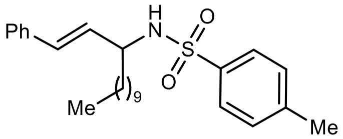

Following General Procedure E: (*E*)-Phenyl(1-phenyltridec-1-en-3-yl)selane (**2c**) (466 mg, 1.13 mmol, 1.00 equiv.), 4-methylbenzenesulfonamide (**3r**) (575 mg, 3.36 mmol, 2.97 equiv.), TFA (0.04 mL, 0.5 mmol, 0.5 equiv.), 5 h; eluting with Tol/PE 1:1 to pure Tol to Tol/EA 9:1; NMR yield: 56%; isolated yield: 258 mg, 0.604 mmol, 54%, yellowish crystals.

R*f* = 0.38 (Tol): **mp**: 87.3 °C; **^{1}H NMR** (300 MHz, $CDCl_3$): δ [ppm] = 7.72 (dd, *J* = 8.5, 2.0 Hz, 2H), 7.32–7.06 (m, 7H), 6.21 (d, *J* = 15.9 Hz, 1H), 5.71 (dd, *J* = 15.8, 7.5 Hz, 1H), 4.48 (d, *J* = 7.8 Hz, 1H), 3.98–3.82 (m, 1H), 2.30 (s, 3H), 1.54 (d, *J* = 10.4 Hz, 2H), 1.23 (d, *J* = 8.9 Hz, 16H), 0.93–0.79 (m, 3H); **^{13}C NMR** (101 MHz, $CDCl_3$): δ [ppm] = 143.2, 138.2, 136.3, 131.4, 129.5, 129.0, 128.4, 127.7, 127.3, 126.3, 56.3, 36.0, 31.9, 29.6, 29.5, 29.4, 29.3, 29.2, 25.4, 22.7, 21.4, 14.1; **HRMS** (ESI-MS) [$C_{26}H_{37}NO_2S$]: ([M+H]$^+$), obs.: 428.2620, calcd.: 428.2618, ([M+Na]$^+$), obs.: 450.2442, calcd.: 450.2437, ([M+NH_4]$^+$), obs.: 445.2885, calcd.: 445.2883; **IR** (ATR, neat): $\tilde{\nu}$ [cm^{-1}] = 3273, 3027, 2926, 2855, 1599, 1495, 1450, 1327, 1159, 1096, 965, 813, 749, 693.

***N*-(*tert*-Butyl)-4-methylbenzenesulfonamide (4dr)**

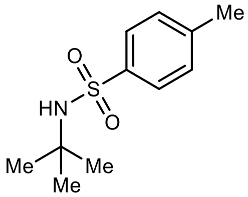

Following General Procedure E: *tert*-Butyl(phenyl)selane (**2d**) (218 mg, 1.02 mmol, 1.00 equiv.), 4-methylbenzenesulfonamide (**3r**) (541 mg, 3.16 mmol, 3.10 equiv.), TFA (0.04 mL, 0.5 mmol, 0.5 equiv.), 6 d; eluting with pure Tol to Tol/EA 9:1; NMR yield: 79%; isolated yield: 164 mg, 0.719 mmol, 70%, colourless solid.

R*f* = 0.3 (Tol/EA 9:1); **mp**: 112.1 °C; **^{1}H NMR** (400 MHz, $CDCl_3$): δ [ppm] = 7.79–7.74 (m, 2H), 7.31–7.23 (m, 2H), 4.49 (d, *J* = 19.7 Hz, 1H), 2.42 (s, 3H), 1.22 (s, 9H).; **^{13}C NMR** (101 MHz, $CDCl_3$): δ [ppm] = 142.9, 140.5, 129.5, 127.0, 54.6, 30.2, 21.5; **HRMS** (ESI-MS) [$C_{11}H_{17}NO_2S$]: ([M+H]$^+$), obs.: 228.1057, calcd.: 228.1053, ([M+Na]$^+$), obs.: 250.0879, calcd.: 250.0872, ([M+NH_4]$^+$), obs.: 245.1321, calcd.: 245.1318; **IR** (ATR, neat): $\tilde{\nu}$ [cm^{-1}] = 3265, 2974, 2878, 1599, 1394, 1305, 1141, 1096, 999, 872, 816.

***N*-(Adamantan-1-yl)-4-methylbenzenesulfonamide (4er)**

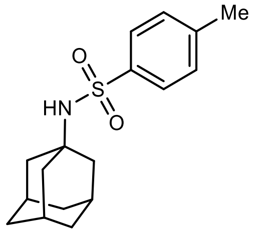

Following General Procedure E: Adamantan-1-yl(phenyl)selane (**2e**) (288 mg, 0.989 mmol, 1.00 equiv.), 4-methylbenzenesulfon amide (**3r**) (506 mg, 2.95 mmol, 2.98 equiv.), TFA (0.04 mL, 0.5 mmol, 0.5 equiv.), 28 h; eluting with pure Tol to Tol/EA 9:1; NMR yield: 95%; isolated yield: 208 mg, 0.680 mmol, 69%, colourless solid.

R*f* = 0.15 (Tol); **mp**: 166.8 °C; **^{1}H NMR** (400 MHz, $CDCl_3$): *δ* [ppm] = 7.84–7.67 (m, 2H), 7.31–7.24 (m, 2H), 4.39 (s, 1H), 2.42 (s, 3H), 2.01 (s, 3H), 1.78 (d, *J* = 2.9 Hz, 6H), 1.62–1.47 (m, 6H). **^{13}C NMR** (101 MHz, $CDCl_3$): *δ* [ppm] = 142.8, 141.1, 129.5, 127.0, 55.1, 43.1, 35.9, 29.5, 21.5; **HRMS** (ESI-MS) [$C_{17}H_{23}NO_2S$]: ($[M+H]^+$), obs.: 306.1528, calcd.: 306.1522, ($[M+Na]^+$), obs.: 328.1350, calcd.: 238.1342, ($[M+NH_4]^+$), obs.: 323.1791, calcd.: 323.1788; **IR** (ATR, neat): $\tilde{\nu}$ [cm^{-1}] = 3288, 2907, 2848, 1431, 1364, 1316, 1152, 1088, 965, 865, 809, 708.

Methyl 4-((4-methylphenyl)sulfonamido)bicyclo[2.2.2]octane-1-carboxylate (4fr)

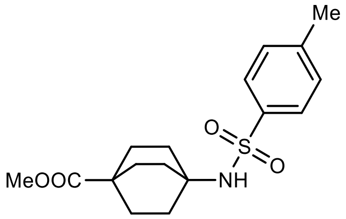

Following General Procedure E: Methyl 4-(phenylselanyl) bicyclo[2.2.2]octane-1-carboxylate (**2f**) (67.0 mg, 0.207 mmol, 1.00 equiv.), 4-methylbenzenesulfon amide (**3r**) (107 mg, 0.627 mmol, 3.03 equiv.), TFA (0.01 mL, 0.1 mmol, 0.5 equiv.), 240 h; NMR yield: 20%, compound was isolated from an additional photo reaction to give the analytical data due to purification issues.

R*f* = 0.54 (PE/EA 1:2); **^{1}H NMR** (400 MHz, $CDCl_3$): *δ* [ppm] = 7.79–7.70 (m, 2H), 7.28 (d, *J* = 8.0 Hz, 2H), 3.60 (s, 3H), 2.42 (s, 3H), 1.86–1.67 (m, 12H); **^{13}C NMR** (101 MHz, $CDCl_3$): *δ* [ppm] = 177.3, 143.0, 140.6, 129.6, 126.9, 54.3, 51.8, 38.0, 31.3, 28.6, 21.5; **HRMS** (ESI-MS) [$C_{17}H_{23}NO_4S$]: ($[M+H]^+$), obs.: 338.1421, calcd.: 338.1421; **IR** (ATR, neat): $\tilde{\nu}$ [cm^{-1}] = 3310, 2873, 2952, 2926, 1599, 1703, 1495, 1439, 1331, 1260, 1156, 1096, 947, 902, 854, 816, 663.

Methyl 4-((1,1,1,3,3,3-hexafluoropropan-2-yl)oxy)bicyclo[2.2.2]octane-1-carboxylate (4fr')

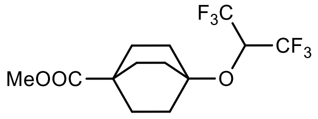

Following General Procedure E: Methyl 4-(phenylselanyl) bicyclo[2.2.2]octane-1-carboxylate (**2f**) (67.0 mg, 0.207 mmol, 1.00 equiv.), 4-methylbenzenesulfon amide (**3r**) (107 mg, 0.627 mmol, 3.03 equiv.), TFA (0.01 mL, 0.1 mmol, 0.5 equiv.), 240 h; NMR yield determined via internal standard trifluoro toluene and ^{19}F NMR: 20%; compound was isolated from an additional photo reaction to give the analytical data due to purification issues.

R*f* = 0.48 (PE/EA 9:1); **^{1}H NMR** (400 MHz, $CDCl_3$): *δ* [ppm] = 4.31 (hept, *J* = 5.9 Hz, 1H), 3.64 (s, 3H), 2.02–1.87 (m, 6H), 1.82–1.72 (m, 6H); **^{13}C NMR** (151 MHz, $CDCl_3$): *δ* [ppm] = 177.0, 124.6–118.3 (m), 80.0, 69.1 (hept, *J* = 32.4 Hz), 51.87, 38.31, 29.74, 29.29; **^{19}F NMR**

(376 MHz, $CDCl_3$): δ [ppm] = 73.8 (d, J = 5.9 Hz); **HRMS** (EI-MS) [$C_{13}H_{16}F_6O_3$]: ([M]$^{+\bullet}$), obs.: 334.0998, calcd.: 334.0998; **IR** (ATR, neat): $\tilde{\nu}$ [cm^{-1}] = 2960, 2930, 2878, 2855, 1722, 1461, 1372, 1349, 1282, 1223, 1103, 936, 891.

2',4',6'-Trimethoxy-1,2,3,4-tetrahydro-1,1'-biphenyl (4aw)

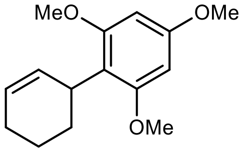

Following General Procedure D: Cyclohex-2-en-1-yl(phenyl)selane (**2a**) (122 mg, 513 mmol, 1.00 equiv.), 1,3,5-trimethoxybenzene (**3w**) (257 mg, 1.53 mmol, 2.98 equiv.), TFA (0.02 mL, 0.3 mmol, 0.6 equiv.), 3 h; eluting 1st column with PE/Tol 1:1, 2nd column with PE/Tol 9:1, and 3rd column with PE/EA 19:1; NMR-yield: 61%; isolated yield: 40.2 mg, 0.162 mmol, 32%, colourless crystals.

R$_f$ = 0.28 (PE/EA 19:1); **mp**: 63.2 °C; **^{1}H NMR** (400 MHz, $CDCl_3$): δ [ppm] = 6.14 (s, 2H), 5.64 (ddt, J = 12.8, 5.3, 2.4 Hz, 1H), 5.57–5.51 (m, 1H), 3.97–3.87 (m, 1H), 3.80 (s, 3H), 3.76 (s, 6H), 2.18–1.94 (m, 2H), 1.97–1.78 (m, 2H), 1.77–1.59 (m, 2H); **^{13}C NMR** (101 MHz, $CDCl_3$): δ [ppm] = 159.3, 159.2, 132.4, 124.0, 115.0, 91.3, 55.9, 55.3, 32.1, 28.0, 24.8, 23.6; **HRMS** (EI-MS) [$C_{15}H_{20}O_3$]: ([M]$^{+\bullet}$), obs.: 248.1411, calcd.: 248.1407; **IR** (ATR, neat): $\tilde{\nu}$ [cm^{-1}] = 2997, 2933, 2837, 2356, 1607, 1495, 1454, 1331,1219, 1152, 1118, 1066, 984.

2-(*tert*-Butyl)-1,3,5-trimethoxybenzene (4dw)

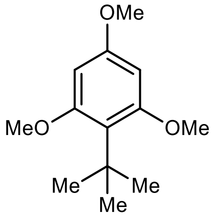

Following General Procedure D; *tert*-Butyl(phenyl)selane (**2d**) (106 mg, 0.497 mmol, 1.00 equiv.), 1,3,5-trimethoxybenzene (**3w**) (265 mg, 1.58 mmol, 3.18 equiv.), TFA (0.02 mL, 0.3 mmol, 0.6 equiv.), 48 h; eluting 1st column with PE/Tol 1:1, and 2nd column with PE/Et_2O 4:1; NMR-yield: 24%; isolated yield: 16.7 mg, 74.5 µmol, 15%, beige crystals.

R$_f$ = 0.67 (PE/Et_2O 4:1); **mp**: 65.3 °C; **^{1}H NMR** (400 MHz, $CDCl_3$): δ [ppm] = 6.14 (s, 2H), 3.79 (s, 3H), 3.76 (s, 6H), 1.44 (s, 9H); **^{13}C NMR** (101 MHz, $CDCl_3$): δ [ppm] = 160.3, 158.5, 118.7, 93.0, 56.0, 55.1, 35.8, 32.0; **HRMS** (EI-MS) [$C_{13}H_{16}F_6O_3$]: ([M]$^{+\bullet}$), obs.: 224.1400, calcd.: 244.1407; **IR** (ATR, neat): $\tilde{\nu}$ [cm^{-1}] = 2997, 2948, 2837, 1607, 1454, 1409, 1357, 1327, 1230, 1193, 1159, 1118, 1059, 954, 813

4'-Methoxy-1,2,3,4-tetrahydro-1,1'-biphenyl (4ax), 2'-methoxy-1,2,3,4-tetrahydro-1,1'-biphenyl (4ax')

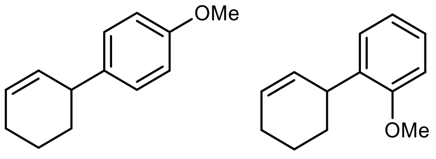

4ax **4ax'**

Following General Procedure D: Cyclohex-2-en-1-yl(phenyl)selane (**2a**) (119 mg, 500 mmol, 1.00 equiv.), anisole (**3x**) (174 mg, 1.61 mmol, 3.22 equiv.), TFA (0.02 mL, 0.3 mmol, 0.6 equiv.), 3 h; as reported by Trillo *et al.* products could not be isolated due to impossible separation from starting materials or side products even after multiple attempts, thus only the NMR yield was determined according to the characteristic allylic proton signals in the ^{1}H NMR[3] and the presence of the products was confirmed via HRMS. NMR-yield: **4ax**: 17%, **4ax'**: 10%

HRMS (EI-MS) [$C_{13}H_{16}O$]: ($[M]^{+\bullet}$), obs.: 188.1200, calcd.: 188.1196.

1-(*tert*-Butyl-4-methoxybenzene (4dx)

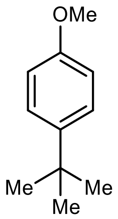

Following General Procedure D; *tert*-Butyl(phenyl)selane (**2d**) (104 mg, 0.487 mmol, 1.00 equiv.), anisole (**3x**) (174 mg, 1.61 mmol, 3.31 equiv.), TFA (0.02 mL, 0.3 mmol, 0.6 equiv.), 48 h; eluting with PE/Tol (19:1), and with PE/DCM (19:1); NMR-yield: 18%; determined according to the characteristic methyl and methoxy signals in the ^{1}H NMR, all signals are according to literature.[4]

R_f = 0.35 (PE/Tol 9:1); **^{1}H NMR** (300 MHz, $CDCl_3$): δ [ppm] = 7.39–7.28 (m, 2H), 6.94–6.78 (m, 2H), 3.80 (s, 3H), 1.32 (s, 9H); **HRMS** (EI-MS) [$C_{11}H_{16}O$]: ($[M]^{+\bullet}$), obs.: 164.1192, calcd.: 164.1196.

16 References

1. Narayanaperumal, S. *et al.* Ionic liquid: an efficient and recyclable medium for synthesis of unsymmetrical diorganyl selenides promoted by InI. *Org. Biomol. Chem.* **7,** 4647–4650; 10.1039/B910699E (2009).
2. Ji, S., Cao, W., Yu, Y. & Xu, H. Dynamic diselenide bonds: exchange reaction induced by visible light without catalysis. *Angew. Chem. Int. Ed.* **53,** 6781–6785; 10.1002/anie.201403442 (2014).
3. Trillo, P., Baeza, A. & Nájera, C. Fluorinated alcohols as promoters for the metal-free direct substitution reaction of allylic alcohols with nitrogenated, silylated, and carbon nucleophiles. *J. Org. Chem.* **77,** 7344–7354; 10.1021/jo301049w (2012).
4. Huang, H. & Lambert, T. H. Electrophotocatalytic SN Ar Reactions of Unactivated Aryl Fluorides at Ambient Temperature and Without Base. *Angew. Chem. Int. Ed.* **59,** 658–662; 10.1002/anie.201909983 (2020).
5. Ito, O. Kinetic study for reactions of phenylseleno radical with vinyl monomers. *J. Am. Chem. Soc.* **105,** 850–853; 10.1021/ja00342a034 (1983).
6. Xu, H. *et al.* Design, synthesis and biological evaluation of novel semicarbazone-selenochroman-4-ones hybrids as potent antifungal agents. *Bioorganic Med. Chem. Lett.* **29,** 126726; 10.1016/j.bmcl.2019.126726 (2019).
7. Ahrika, A., Robert, J., Anouti, M. & Paris, J. Characterization of organic polyselenide ions in N,N-dimethylacetamide. *New J. Chem.* **25,** 741–746; 10.1039/B100877N (2001).
8. Pavlovska, T. *et al.* Tuning Deazaflavins Towards Highly Potent Reducing Photocatalysts Guided by Mechanistic Understanding - Enhancement of the Key Step by the Internal Heavy Atom Effect. *Chem. Eur. J.* **28,** e202200768; 10.1002/chem.202200768 (2022).
9. Kutta, R.-J., Langenbacher, T., Kensy, U. & Dick, B. Setup and performance of a streak camera apparatus for transient absorption measurements in the ns to ms range. *Appl. Phys. B* **111,** 203–216; 10.1007/s00340-012-5320-2 (2013).
10. Lanzl, K., Sanden-Flohe, M. V., Kutta, R.-J. & Dick, B. Photoreaction of mutated LOV photoreceptor domains from Chlamydomonas reinhardtii with aliphatic mercaptans: implications for the mechanism of wild type LOV. *Phys. Chem. Chem. Phys.* **12,** 6594–6604; 10.1039/B922408D (2010).
11. Kutta, R.-J. *Blitzlichtphotolyse - Untersuchung zu LOV-Domänen und photochromen Systemen (Dissertation)* (Regensburg, 2012).
12. König, B. *Chemical photocatalysis* (De Gruyter, Berlin, Boston, 2013).
13. Myers, D. Y., Stroebel, G. G., Ortiz de Montellano, B. R. & Gardner, P. D. Reaction of 3-cyclohexenyl radical with nucleophiles. *J. Am. Chem. Soc.* **95,** 5832–5833; 10.1021/ja00798a102 (1973).
14. Sakamoto, M. *et al.* Transient Absorption Spectra and Lifetimes of Benzophenone Ketyl Radicals in the Excited State. *J. Phys. Chem. A* **108,** 8147–8150; 10.1021/jp047058a (2004).
15. Schuler, R. H. & Patterson, L. K. The absorption spectra of cyclohexyl and cyclohexenyl radicals. *Chem. Phys. Lett.* **27,** 369–375; 10.1016/0009-2614(74)90243-7 (1974).

16. Tojo, S., Fujitsuka, M., Ouchi, A. & Majima, T. Selenium–Selenium Bond Cleavage of Diaryl Diselenide Radical Anions During Pulse Radiolysis. *ChemPlusChem* **80,** 68–73; 10.1002/cplu.201402300 (2015).

17. Ortgies, S. *et al.* Mechanistic and Synthetic Investigations on the Dual Selenium-π-Acid/Photoredox Catalysis in the Context of the Aerobic Dehydrogenative Lactonization of Alkenoic Acids. *ACS Catal.* **7,** 7578–7586; 10.1021/acscatal.7b02729 (2017).

18. Koppel, I. A. *et al.* The Gas-Phase Acidities of Very Strong Neutral Bronsted Acids. *J. Am. Chem. Soc.* **116,** 3047–3057; 10.1021/ja00086a038 (1994).

19. Hohenberg, P. & Kohn, W. Inhomogeneous Electron Gas. *Phys. Rev.* **136,** B864-B871; 10.1103/PhysRev.136.B864 (1964).

20. Sham, L. J. & Kohn, W. One-Particle Properties of an Inhomogeneous Interacting Electron Gas. *Phys. Rev.* **145,** 561–567; 10.1103/PhysRev.145.561 (1966).

21. Møller, C. & Plesset, M. S. Note on an Approximation Treatment for Many-Electron Systems. *Phys. Rev.* **46,** 618–622; 10.1103/PhysRev.46.618 (1934).

22. Neese, F., Wennmohs, F., Becker, U. & Riplinger, C. The ORCA quantum chemistry program package. *J. Chem. Phys.* **152,** 224108; 10.1063/5.0004608 (2020).

23. Liu, B. Ab initio potential energy surface for linear H3. *J. Chem. Phys.* **58,** 1925–1937; 10.1063/1.1679454 (1973).

24. Aquilante, F. *et al.* Modern quantum chemistry with OpenMolcas. *J. Chem. Phys.* **152,** 214117; 10.1063/5.0004835 (2020).

25. Weigend, F., Köhn, A. & Hättig, C. Efficient use of the correlation consistent basis sets in resolution of the identity MP2 calculations. *J. Chem. Phys.* **116,** 3175–3183; 10.1063/1.1445115 (2002).

26. Hättig, C. Optimization of auxiliary basis sets for RI-MP2 and RI-CC2 calculations: Core–valence and quintuple-ζ basis sets for H to Ar and QZVPP basis sets for Li to Kr. *Phys. Chem. Chem. Phys.* **7,** 59–66; 10.1039/B415208E (2005).

27. Burke, K., Werschnik, J. & Gross, E. K. U. Time-dependent density functional theory: past, present, and future. *J. Chem. Phys.* **123,** 62206; 10.1063/1.1904586 (2005).

28. Vosko, S. H., Wilk, L. & Nusair, M. Accurate spin-dependent electron liquid correlation energies for local spin density calculations: a critical analysis. *Can. J. Phys.* **58,** 1200–1211; 10.1139/p80-159 (1980).

29. Stephens, P. J., Devlin, F. J., Chabalowski, C. F. & Frisch, M. J. Ab Initio Calculation of Vibrational Absorption and Circular Dichroism Spectra Using Density Functional Force Fields. *J. Phys. Chem.* **98,** 11623–11627; 10.1021/j100096a001 (1994).

30. Lee, C., Yang, W. & Parr, R. G. Development of the Colle-Salvetti correlation-energy formula into a functional of the electron density. *Phys. Rev. B* **37,** 785–789; 10.1103/PhysRevB.37.785 (1988).

31. Becke, A. D. Density-functional exchange-energy approximation with correct asymptotic behavior. *Phys. Rev. A.* **38,** 3098–3100; 10.1103/PhysRevA.38.3098 (1988).

32. Caldeweyher, E. *et al.* A generally applicable atomic-charge dependent London dispersion correction. *J. Chem. Phys.* **150,** 154122; 10.1063/1.5090222 (2019).

33. Caldeweyher, E., Bannwarth, C. & Grimme, S. Extension of the D3 dispersion coefficient model. *J. Chem. Phys.* **147,** 34112; 10.1063/1.4993215 (2017).

34. Weigend, F. & Ahlrichs, R. Balanced basis sets of split valence, triple zeta valence and quadruple zeta valence quality for H to Rn: Design and assessment of accuracy. *Phys. Chem. Chem. Phys.* **7,** 3297–3305; 10.1039/B508541A (2005).

35. Hirata, S. & Head-Gordon, M. Time-dependent density functional theory within the Tamm–Dancoff approximation. *Chem. Phys. Lett.* **314,** 291–299; 10.1016/S0009-2614(99)01149-5 (1999).

36. Shiozaki, T., Gyorffy, W., Celani, P. & Werner, H.-J. Communication: extended multi-state complete active space second-order perturbation theory: energy and nuclear gradients. *J. Chem. Phys.* **135,** 81106; 10.1063/1.3633329 (2011).

37. Cossi, M., Rega, N., Scalmani, G. & Barone, V. Energies, structures, and electronic properties of molecules in solution with the C-PCM solvation model. *J. Comput. Chem.* **24,** 669–681; 10.1002/jcc.10189 (2003).

38. Mayans, E. *et al.* Effect of Solvent Choice on the Self-Assembly Properties of a Diphenylalanine Amphiphile Stabilized by an Ion Pair. *ChemPhysChem* **18,** 1888–1896; 10.1002/cphc.201700180 (2017).

39. Eberson, L., Hartshorn, M. P. & Persson, O. 1,1,1,3,3,3-Hexafluoropropan-2-ol as a solvent for the generation of highly persistent radical cations. *J. Chem. Soc., Perkin Trans. 2,* 1735; 10.1039/p29950001735 (1995).

40. Kendall, R. A. & Früchtl, H. A. The impact of the resolution of the identity approximate integral method on modern ab initio algorithm development. *Theor Chem Acta* **97,** 158–163; 10.1007/s002140050249 (1997).

41. Neese, F., Wennmohs, F., Hansen, A. & Becker, U. Efficient, approximate and parallel Hartree–Fock and hybrid DFT calculations. A 'chain-of-spheres' algorithm for the Hartree–Fock exchange. *Chem. Phys.* **356,** 98–109; 10.1016/j.chemphys.2008.10.036 (2009).

42. Plasser, F. TheoDORE: A toolbox for a detailed and automated analysis of electronic excited state computations. *J. Chem. Phys.* **152,** 84108; 10.1063/1.5143076 (2020).

43. Humphrey, W., Dalke, A. & Schulten, K. VMD: visual molecular dynamics. *J. Mol. Graphics* **14,** 33-8, 27-8; 10.1016/0263-7855(96)00018-5 (1996).

44. Grimme, S. & Hansen, A. A practicable real-space measure and visualization of static electron-correlation effects. *Angew. Chem. Int. Ed.* **54,** 12308–12313; 10.1002/anie.201501887 (2015).

45. Loos, P.-F., Scemama, A., Boggio-Pasqua, M. & Jacquemin, D. Mountaineering Strategy to Excited States: Highly Accurate Energies and Benchmarks for Exotic Molecules and Radicals. *J. Chem. Theory Comput.* **16,** 3720–3736; 10.1021/acs.jctc.0c00227 (2020).

46. Rappoport, D. & Furche, F. Property-optimized gaussian basis sets for molecular response calculations. *J. Chem. Phys.* **133,** 134105; 10.1063/1.3484283 (2010).

47. Vauthey, E. Photoinduced symmetry-breaking charge separation. *ChemPhysChem* **13,** 2001–2011; 10.1002/cphc.201200106 (2012).

48. Bursch, M., Mewes, J.-M., Hansen, A. & Grimme, S. Best-Practice DFT Protocols for Basic Molecular Computational Chemistry. *Angew. Chem. Int. Ed.* **61,** e202205735; 10.1002/anie.202205735 (2022).

49. Mardirossian, N. & Head-Gordon, M. ωB97M-V: A combinatorially optimized, range-separated hybrid, meta-GGA density functional with VV10 nonlocal correlation. *J. Chem. Phys.* **144,** 214110; 10.1063/1.4952647 (2016).

50. Kildahl, N. K. Bond Energy Data Summarized. *J. Chem. Educ.* **72,** 423; 10.1021/ed072p423 (1995).

51. Ji, S., Xia, J. & Xu, H. Dynamic Chemistry of Selenium: Se-N and Se-Se Dynamic Covalent Bonds in Polymeric Systems. *ACS Macro Letters* **5,** 78–82; 10.1021/acsmacrolett.5b00849 (2016).

52. An, X. *et al.* Aromatic diselenide crosslinkers to enhance the reprocessability and self-healing of polyurethane thermosets. *Polym. Chem.* **8,** 3641–3646; 10.1039/C7PY00448F (2017).

53. Baldwin, J. E. & Kelly, D. R. Applications of consecutive radical addition–elimination reactions in synthesis. *J. Chem. Soc., Chem. Commun.,* 682–684; 10.1039/C39850000682 (1985).

54. Cobas, C. NMR signal processing, prediction, and structure verification with machine learning techniques. *Magn. Reson. Chem.* **58,** 512–519; 10.1002/mrc.4989 (2020).

55. Shea, R. G. *et al.* Allylic selenides in organic synthesis: new methods for the synthesis of allylic amines. *J. Org. Chem.* **51,** 5243–5252; 10.1021/jo00376a037 (1986).

56. Zheng, Z. *et al.* Selenenate Anions (PhSeO −) as Organocatalyst: Synthesis of trans - Stilbenes and a PPV Derivative. *Adv. Synth. Catal.* **362,** 659–666; 10.1002/adsc.201901201 (2020).

57. Lei, T. *et al.* Asymmetric Photoaerobic Lactonization and Aza-Wacker Cyclization of Alkenes Enabled by Ternary Selenium-Sulfur Multicatalysis. *ACS Catal.* **13,** 16240–16248; 10.1021/acscatal.3c04443 (2023).

58. Shang, Y., Jie, X., Jonnada, K., Zafar, S. N. & Su, W. Dehydrogenative desaturation-relay via formation of multicenter-stabilized radical intermediates. *Nat Commun* **8,** 2273; 10.1038/s41467-017-02381-8 (2017).

59. Krief, A., Derock, M. & Lacroix, D. Zinc-Mediated Synthesis of Tertiary Alkyl Selenides from Tertiary Alkyl Halides. *Synlett* **18,** 2832–2834; 10.1055/s-2005-918933 (2005).

60. Munbunjong, W. *et al.* Indium-mediated cleavage of diphenyl diselenide and diphenyl disulfide: efficient one-pot synthesis of unsymmetrical diorganyl selenides, sulfides, and selenoesters. *Tetrahedron* **65,** 2467–2471; 10.1016/j.tet.2009.01.072 (2009).

61. Lei, T., Appleson, T. & Breder, A. Intermolecular Aza-Wacker Coupling of Alkenes with Azoles by Photo-Aerobic Selenium-pi-Acid Multicatalysis. *manuscript under consideration* **Manuscript No.: cs-2024-01327d** (2024).

62. Sevov, C. S. *et al.* Evolutionary Design of Low Molecular Weight Organic Anolyte Materials for Applications in Nonaqueous Redox Flow Batteries. *J. Am. Chem. Soc.* **137,** 14465–14472; 10.1021/jacs.5b09572 (2015).

63. Qin, T. *et al.* A general alkyl-alkyl cross-coupling enabled by redox-active esters and alkylzinc reagents. *Science* **352,** 801–805; 10.1126/science.aaf6123 (2016).

64. Cao, J., Gao, L., Wang, G. & Li, S. Pyridine-Boryl Radical Mediated Decarboxylative Homolytic Substitution of N-hydroxyphthalimide Ester with Ar 2 X 2 (X=S, Se). *Eur. J. Org. Chem.* **2022,** e202201290; 10.1002/ejoc.202201290 (2022).

17 ^{1}H-/^{13}C-/^{77}Se-/^{19}F-/IR-spectra

^{1}H NMR (300 MHz, $CDCl_3$) of **2a**

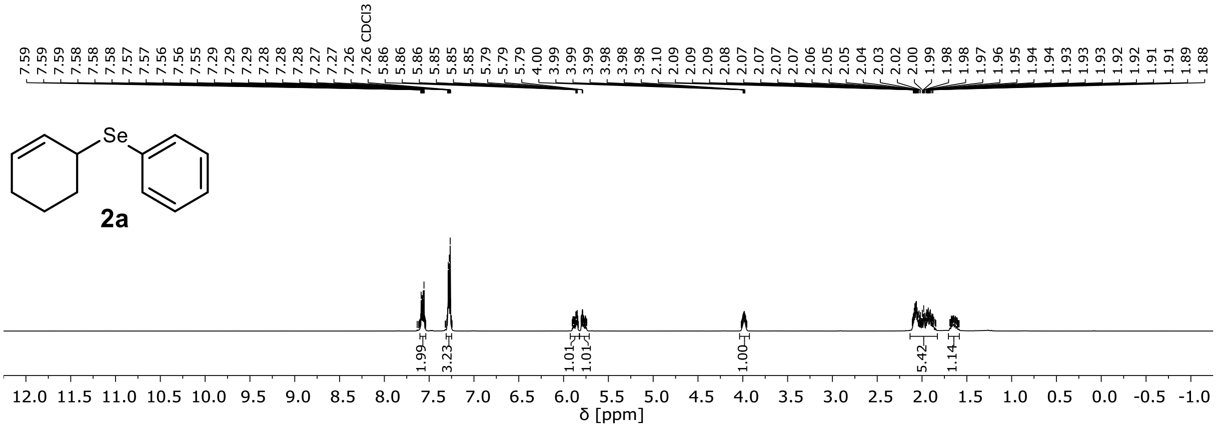

^{13}C NMR (101 MHz, $CDCl_3$) of **2a**

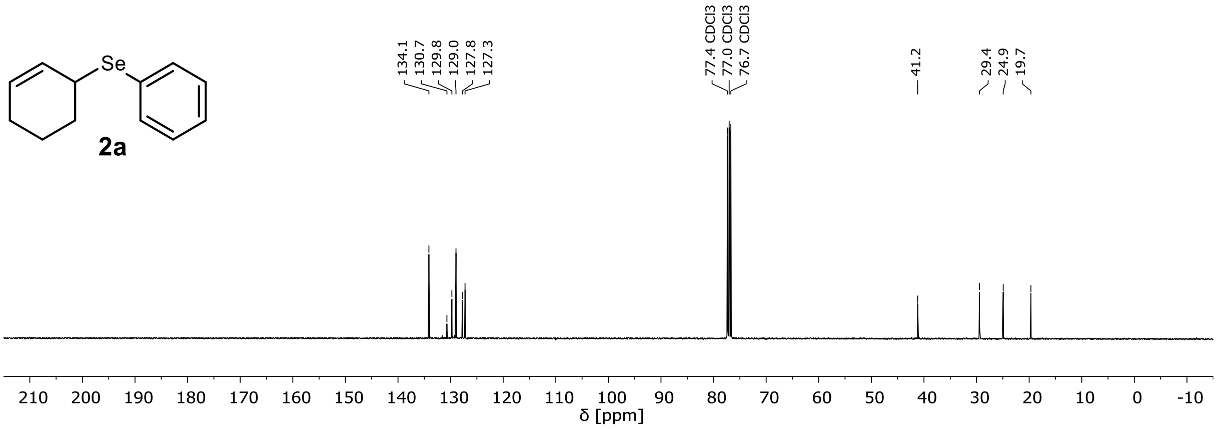

^{77}Se NMR (76 MHz, $CDCl_3$) of **2a**

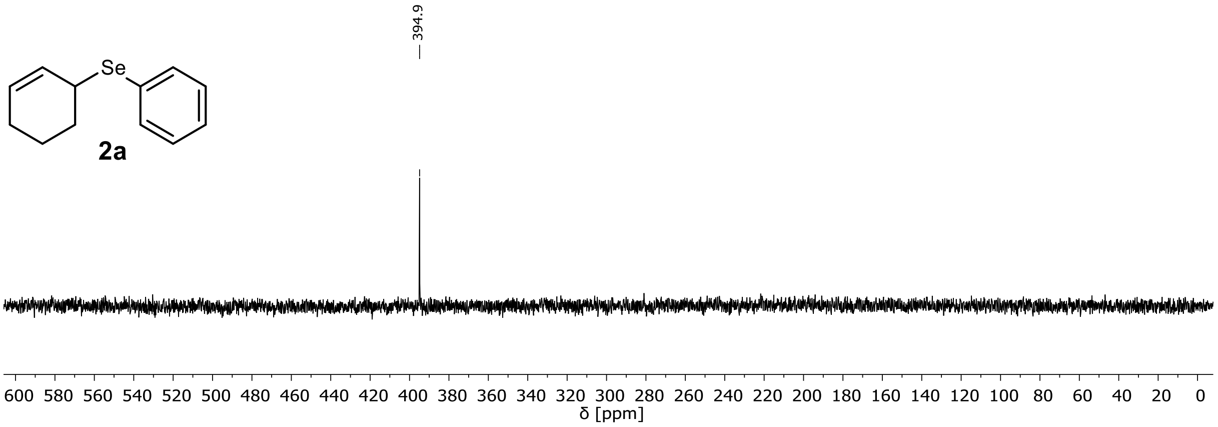

IR (ATR, neat) of **2a**

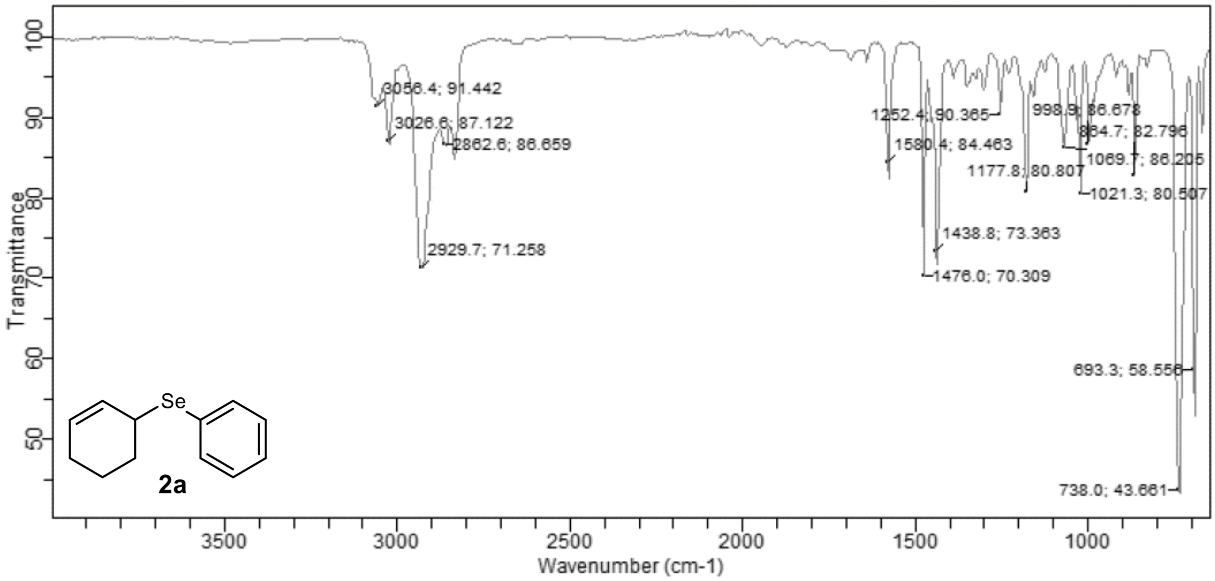

^{1}H NMR (400 MHz, $CDCl_3$) of **2a-1*d***

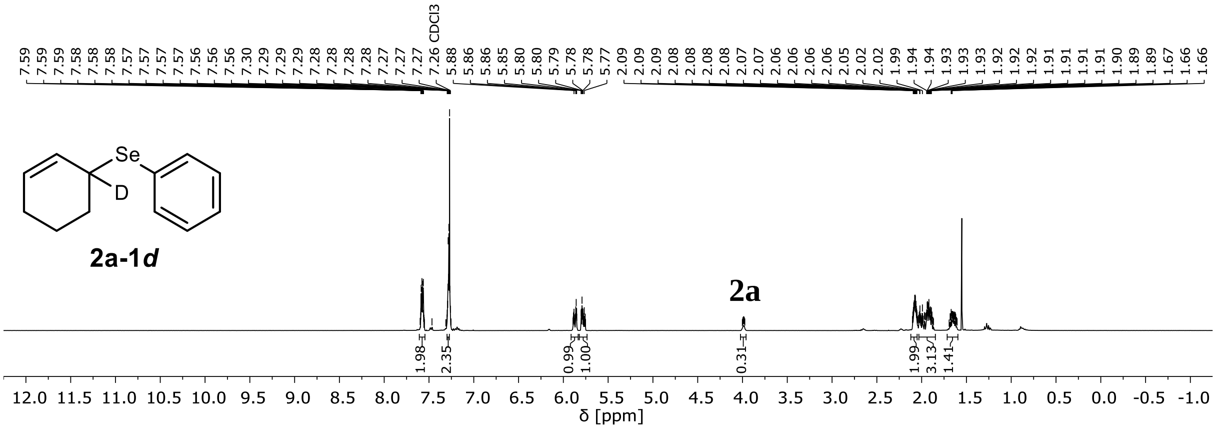

^{13}C NMR (101 MHz, $CDCl_3$) of **2a-1*d***

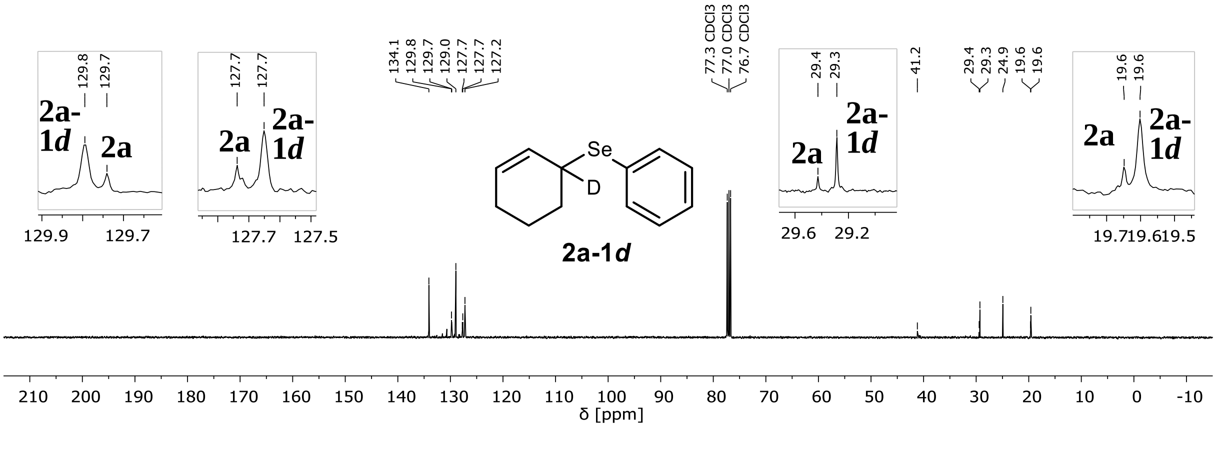

^{77}**Se NMR** (76 MHz, $CDCl_3$) of **2a-1*d***

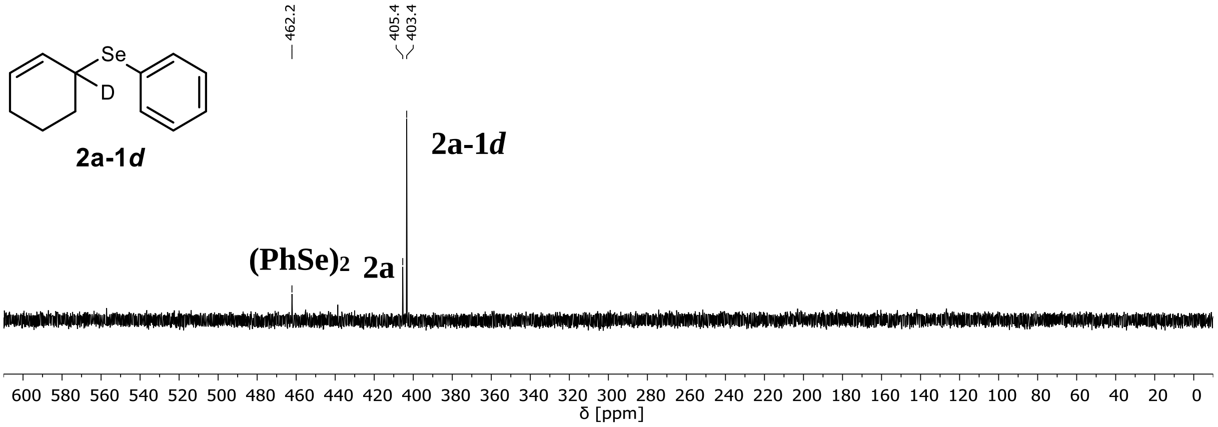

^{2}**H NMR** (61 MHz, $CDCl_3$) of **2a-1*d***

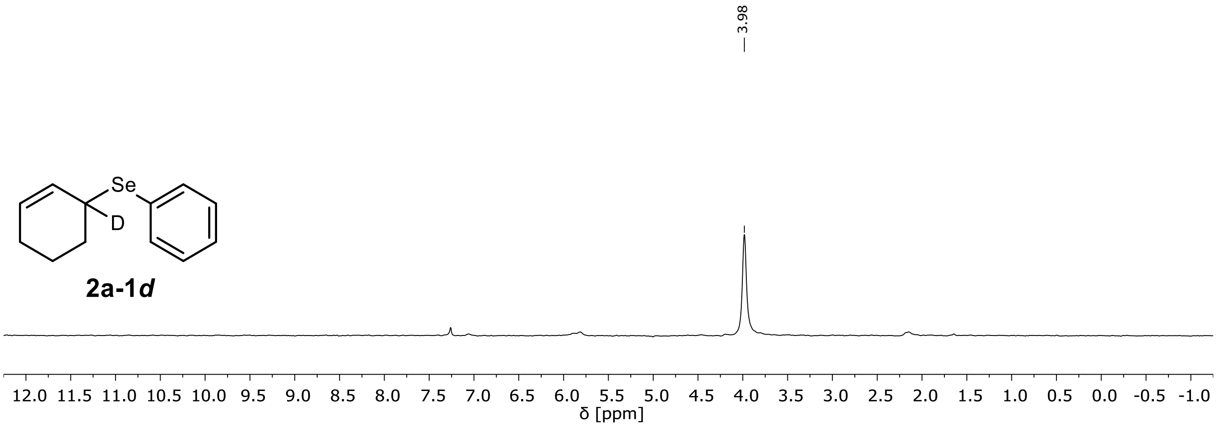

IR (ATR, neat) of **2a-1*d***

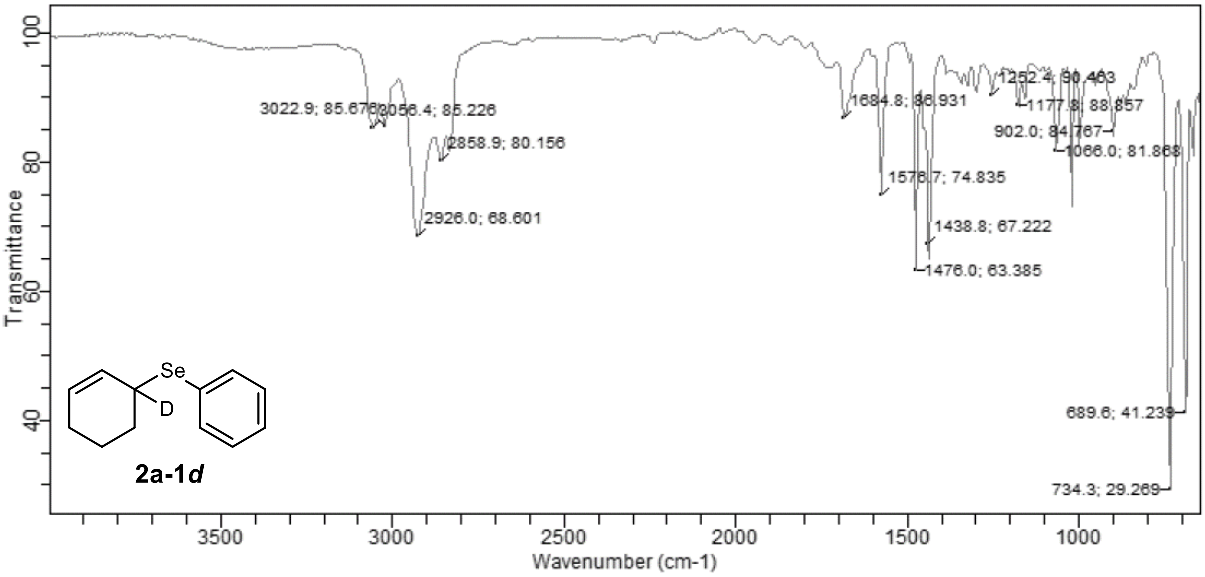

^{1}H NMR (300 MHz, $CDCl_3$) of **1g**

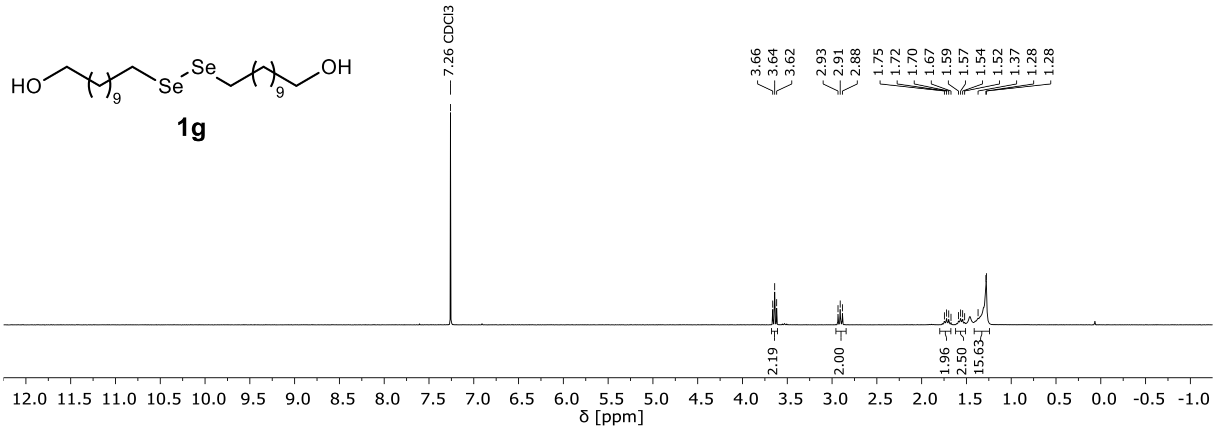

^{13}C NMR (101 MHz, $CDCl_3$) of **1g**

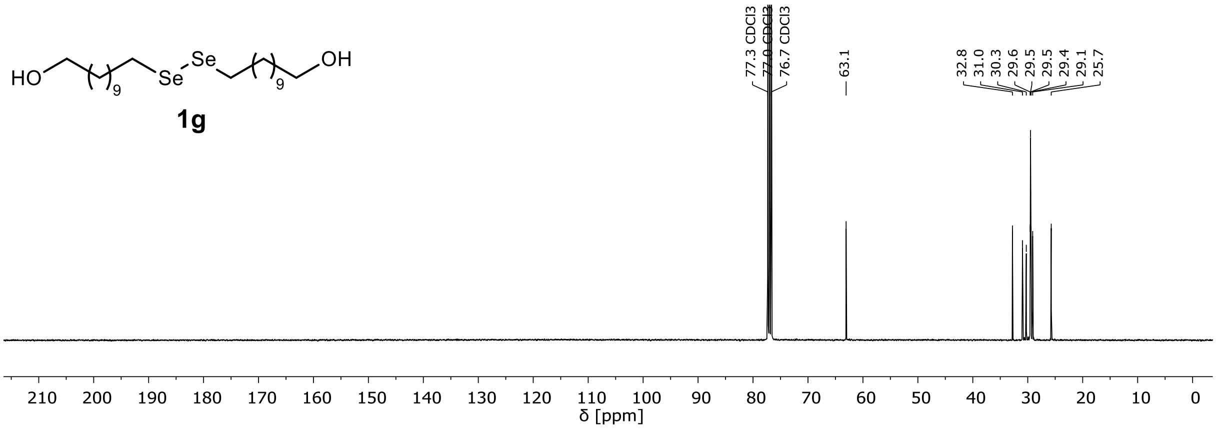

^{77}Se NMR (76 MHz, $CDCl_3$) of **1g**

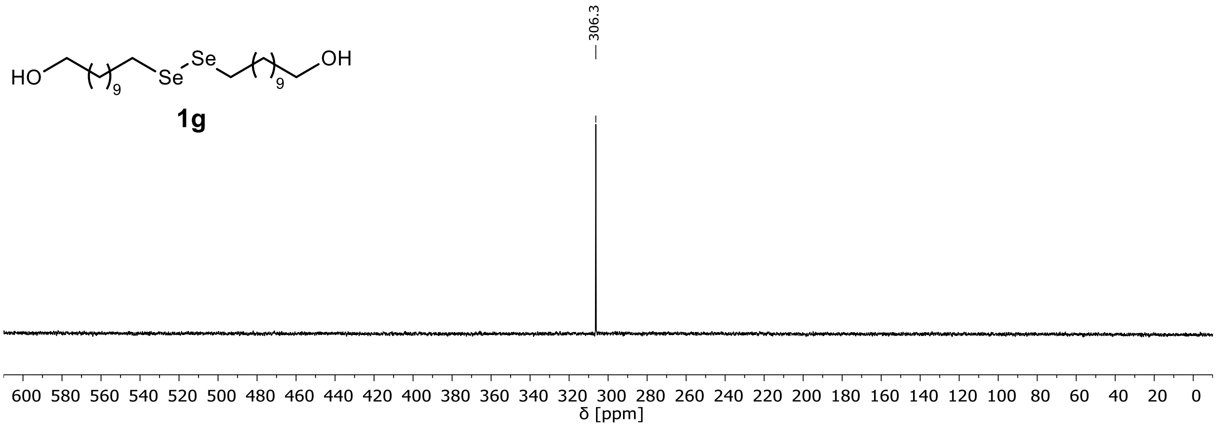

IR (ATR, neat) of **1g**

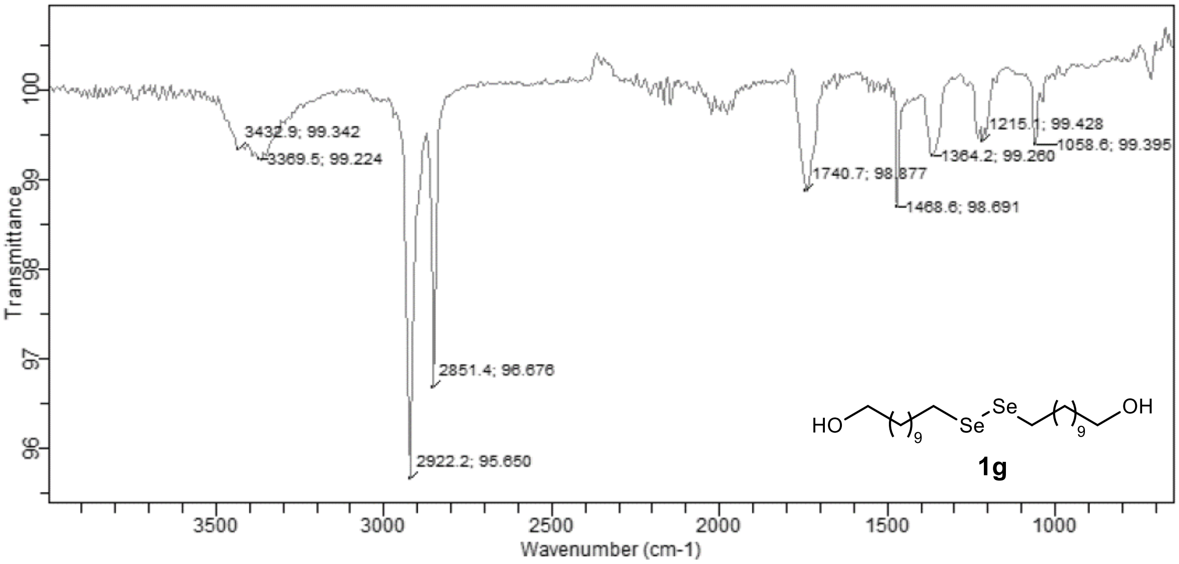

^{1}H NMR (300 MHz, $CDCl_3$) of **2g**

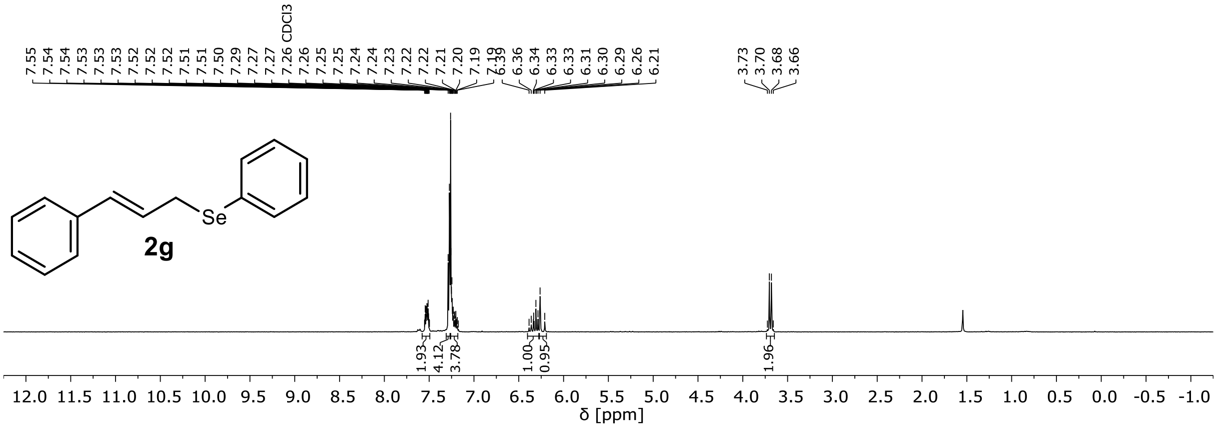

^{13}C NMR (101 MHz, $CDCl_3$) of **2g**

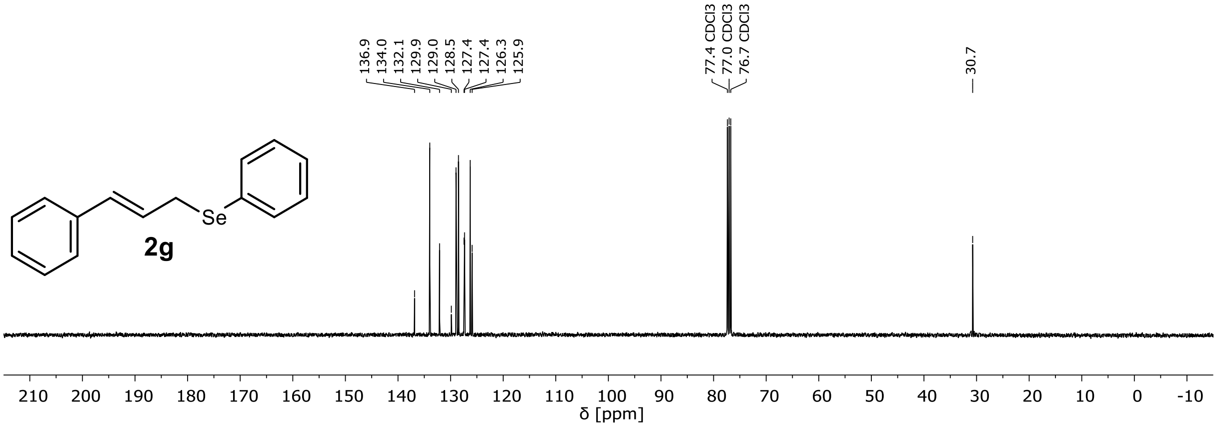

^{77}**Se NMR** (76 MHz, $CDCl_3$) of **2g**

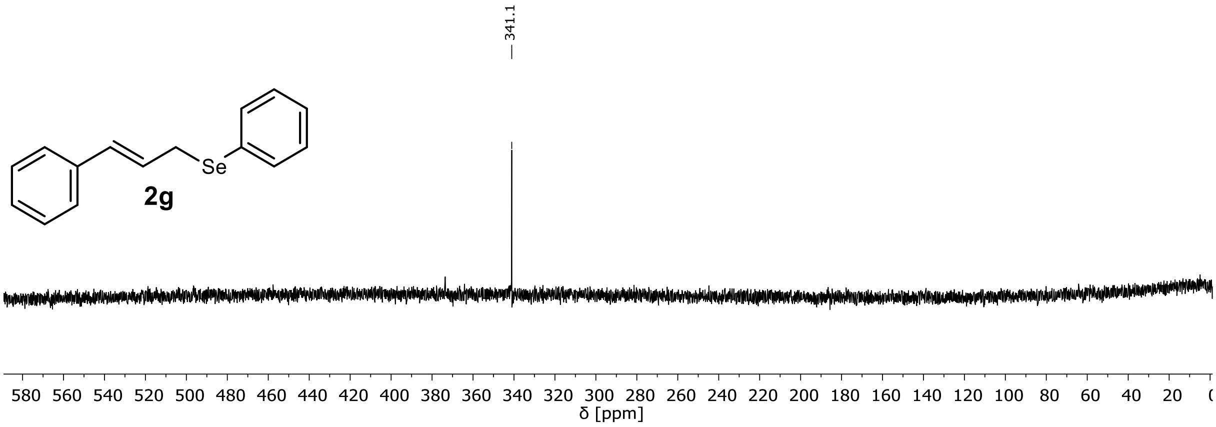

IR (ATR, neat) of **2g**

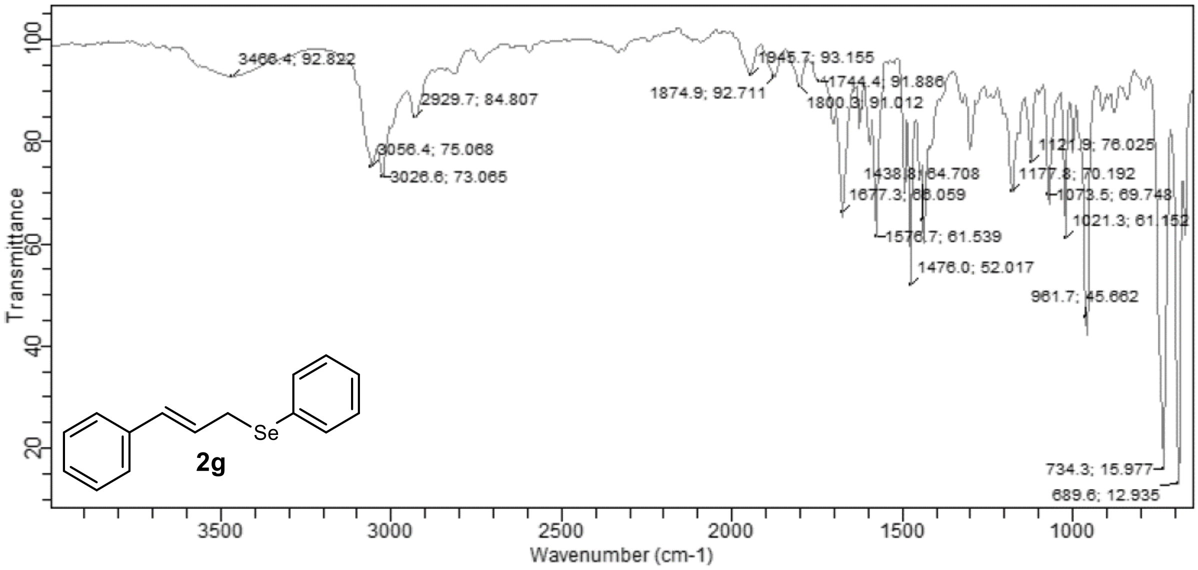

^{1}**H NMR** (400 MHz, $CDCl_3$) of **2b**

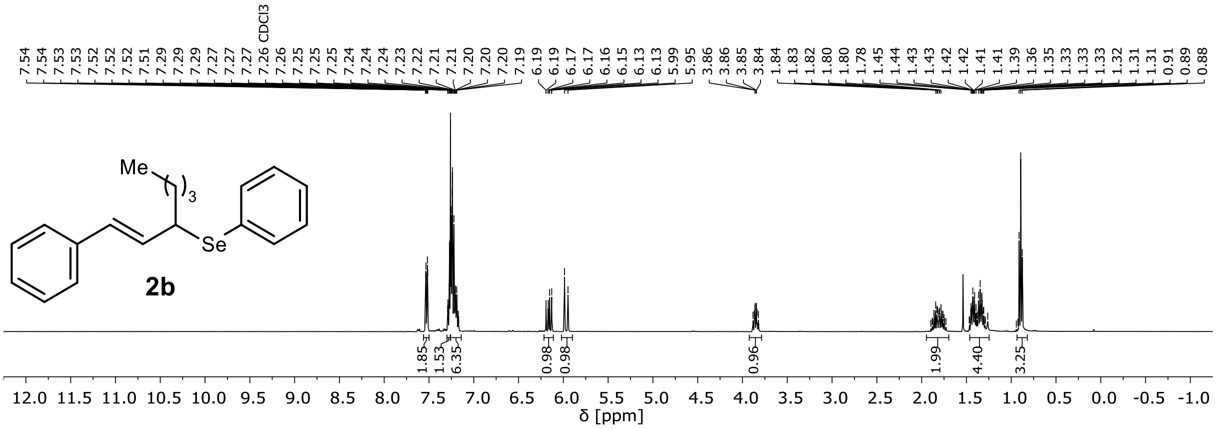

^{13}**C NMR** (101 MHz, $CDCl_3$) of **2b**

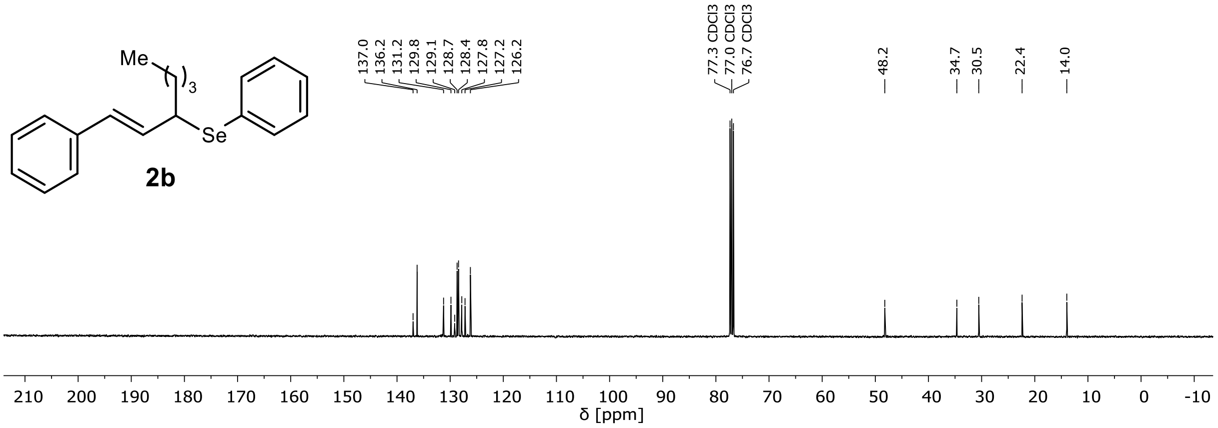

^{77}**Se NMR** (76 MHz, $CDCl_3$) of **2b**

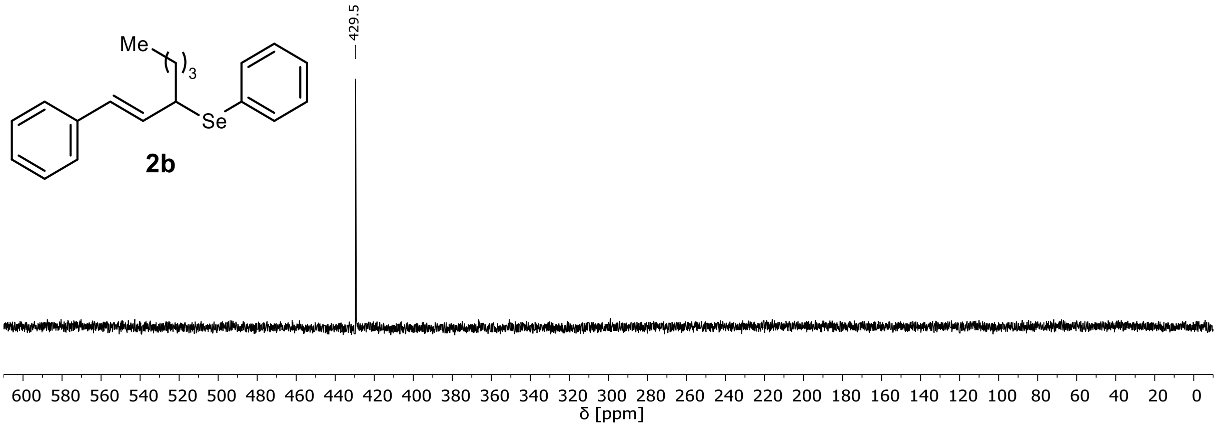

IR (ATR, neat) of **2b**

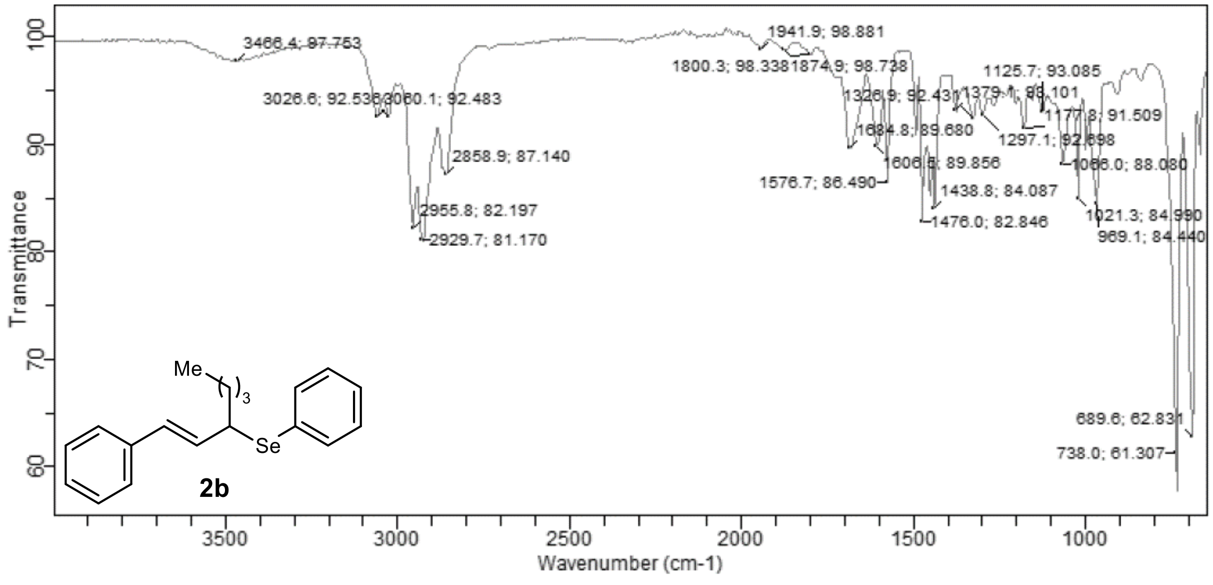

^{1}H NMR (300 MHz, CDCl$_3$) of **2c**

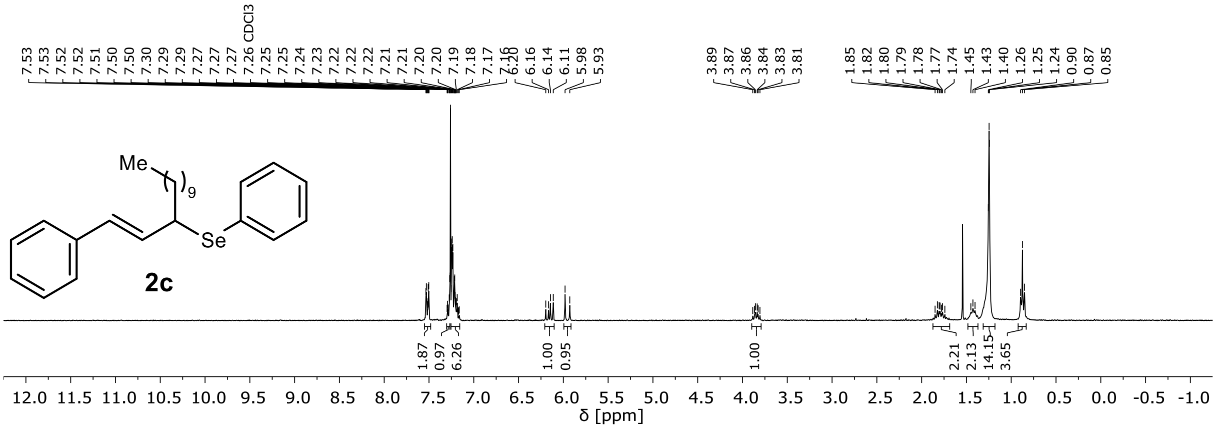

^{13}C NMR (101 MHz, CDCl$_3$) of **2c**

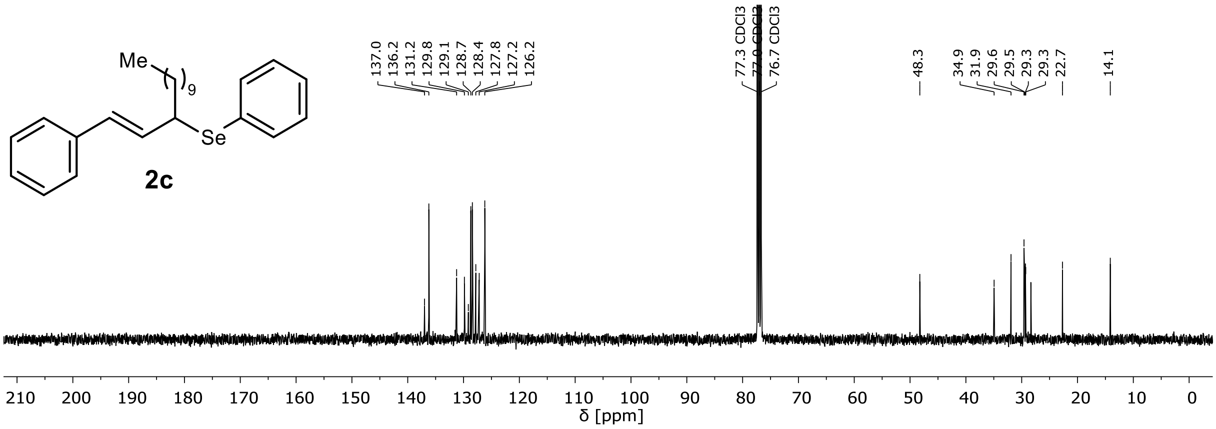

^{77}Se NMR (76 MHz, CDCl$_3$) of **2c**

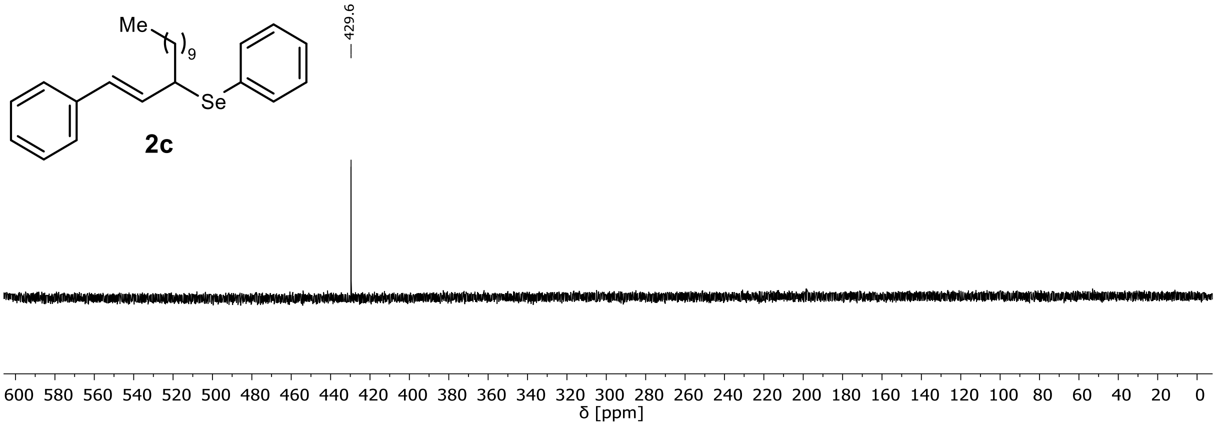

IR (ATR, neat) of **2c**

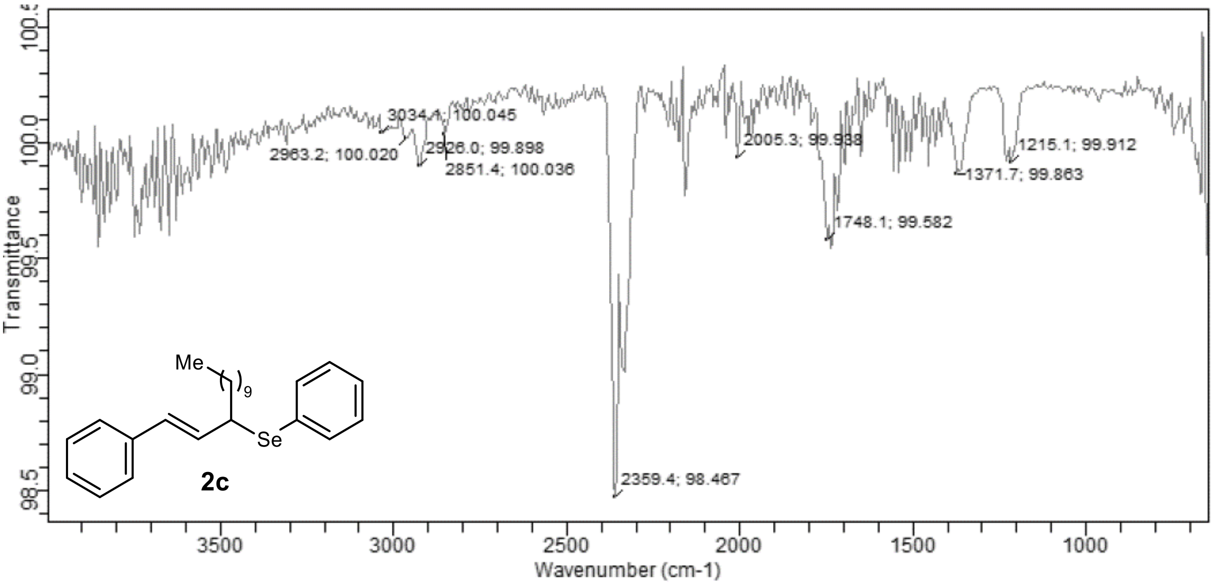

^{1}H NMR (300 MHz, $CDCl_3$) of **2d**

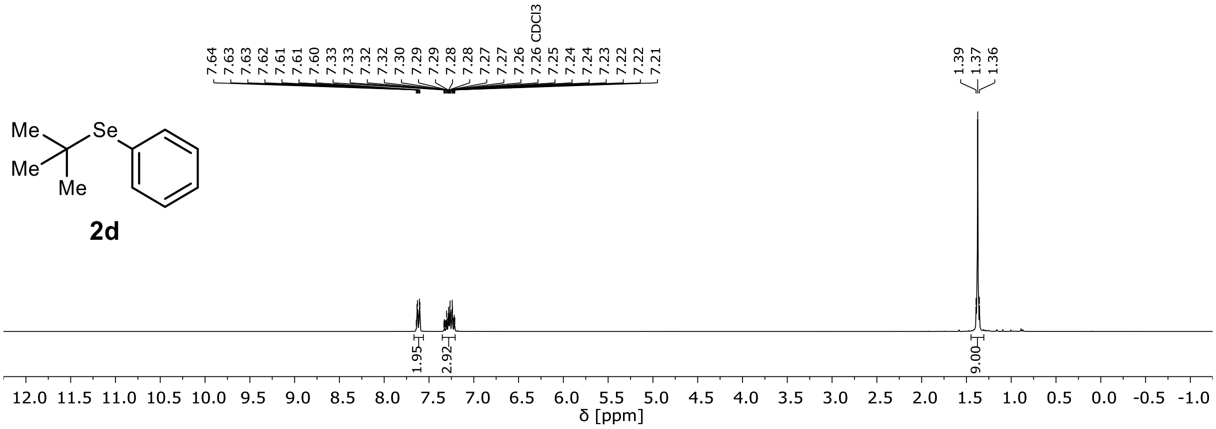

^{13}C NMR (75 MHz, $CDCl_3$) of **2d**

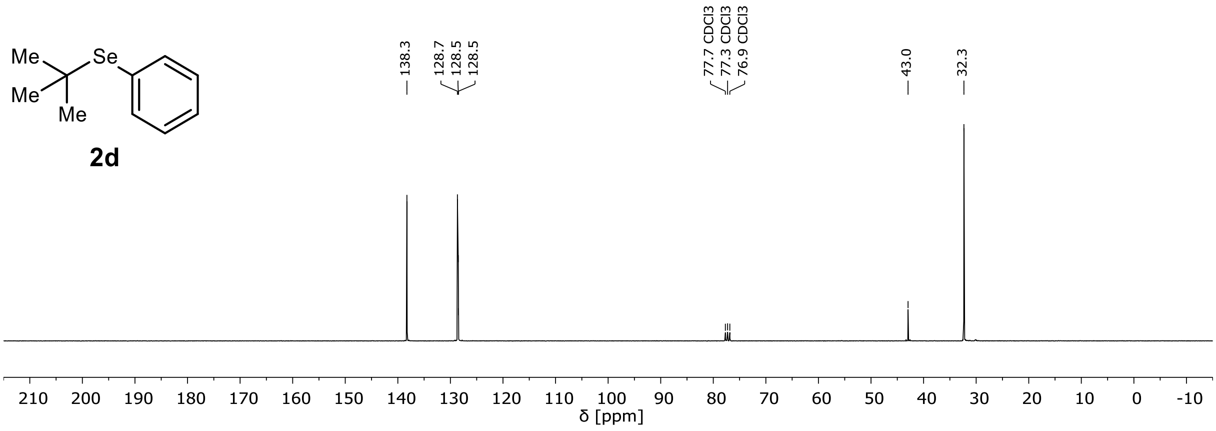

^{77}Se NMR (76 MHz, $CDCl_3$) of **2d**

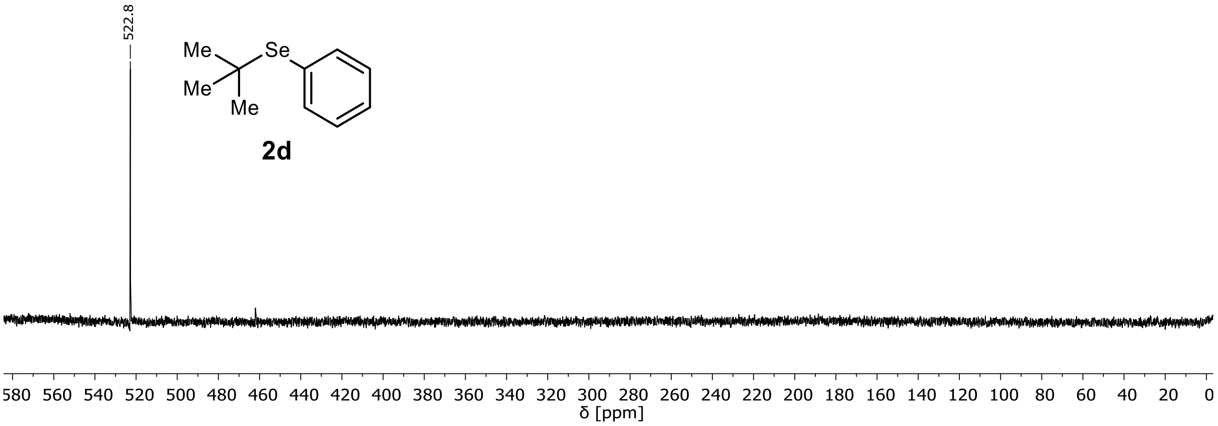

IR (ATR, neat) of **2d**

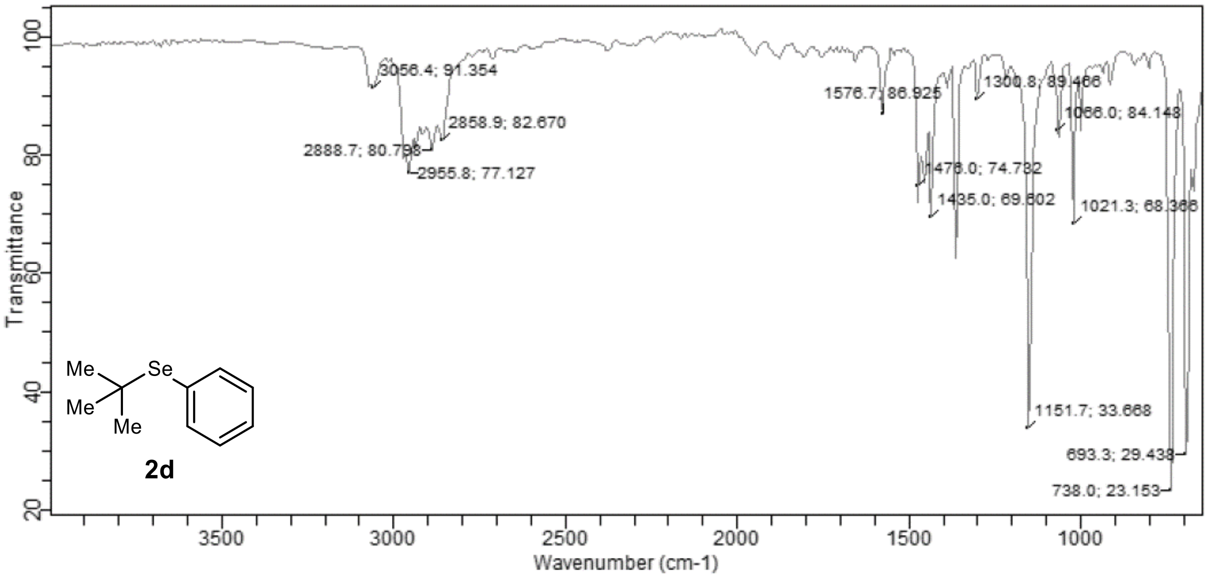

^{1}H NMR (300 MHz, $CDCl_3$) of **2e**

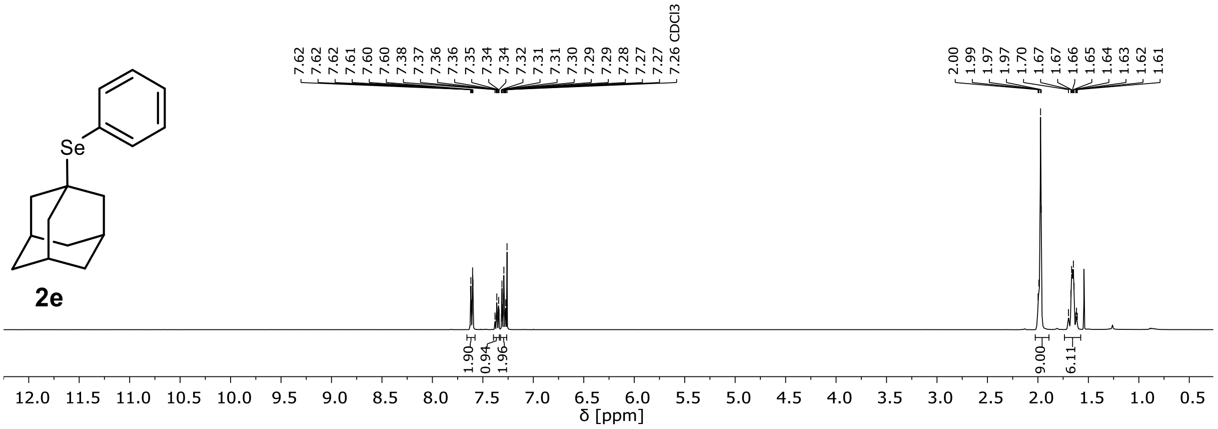

^{13}C NMR (101 MHz, $CDCl_3$) of **2e**

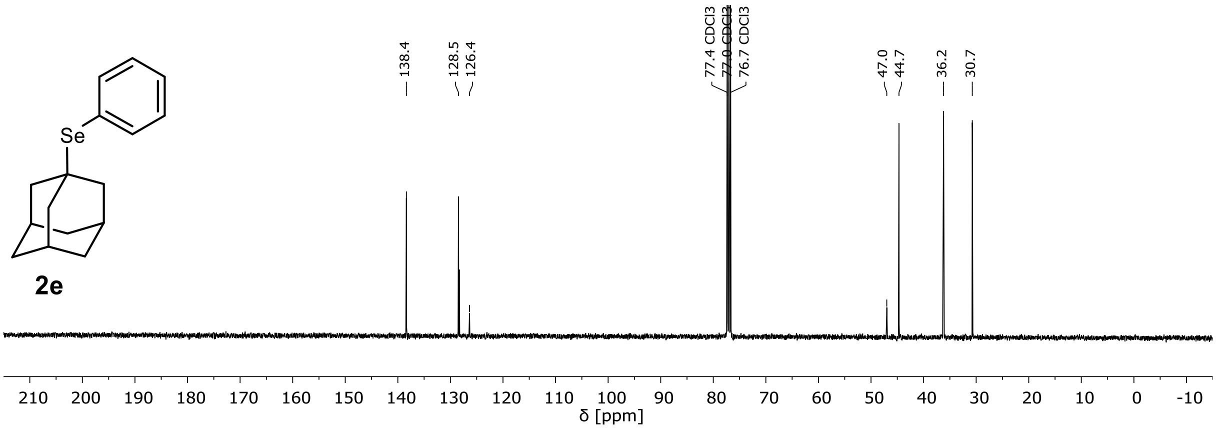

^{77}Se NMR (76 MHz, $CDCl_3$) of **2e**

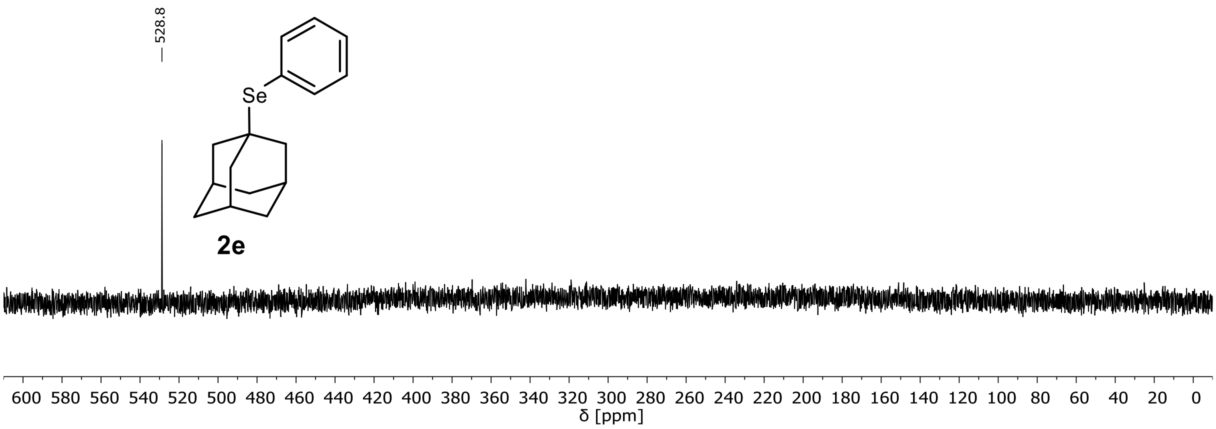

IR (ATR, neat) of **2e**

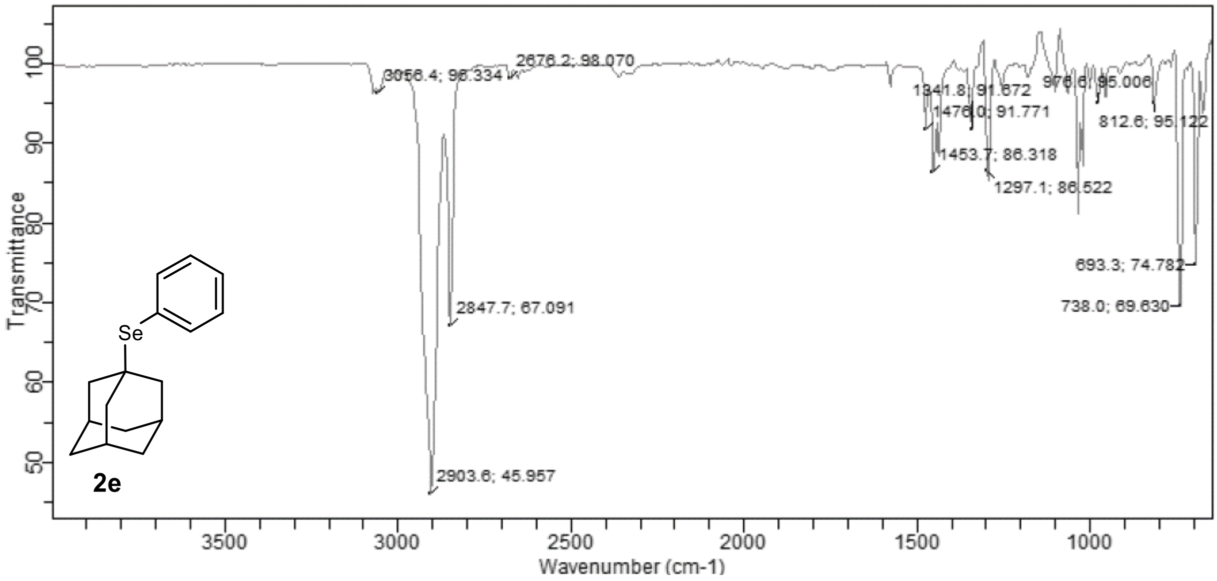

^{1}H NMR (300 MHz, CDCl$_3$) of **6a**

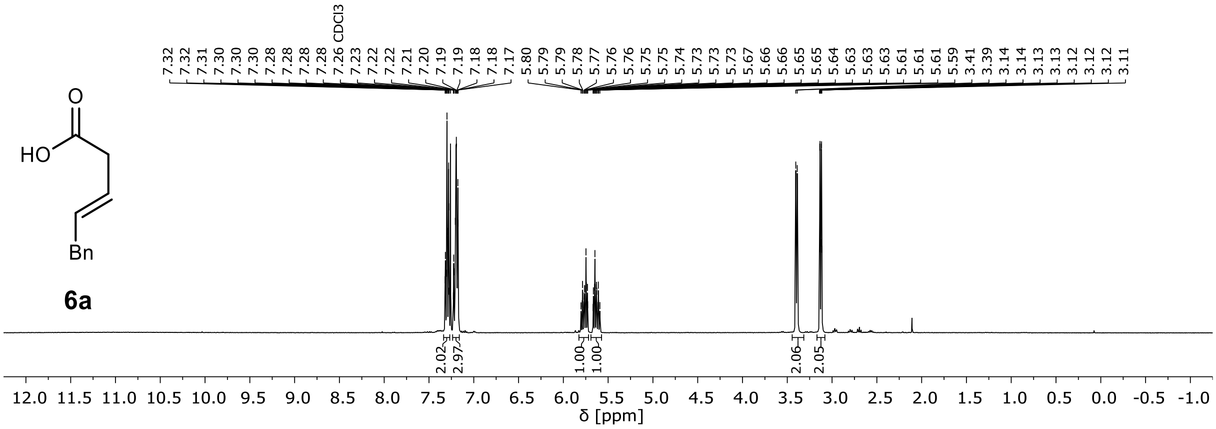

^{13}C NMR (101 MHz, CDCl$_3$) of **6a**

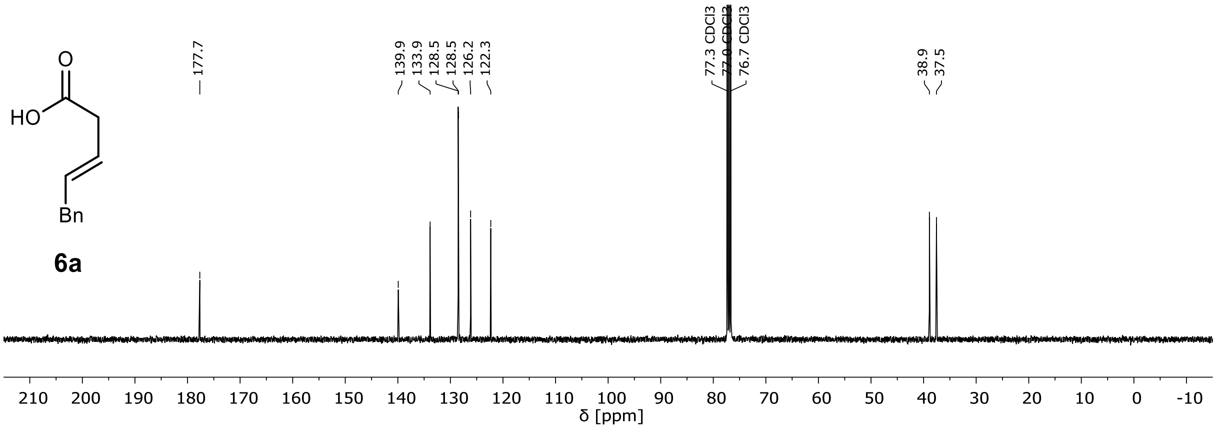

IR (ATR, neat) of **6a**

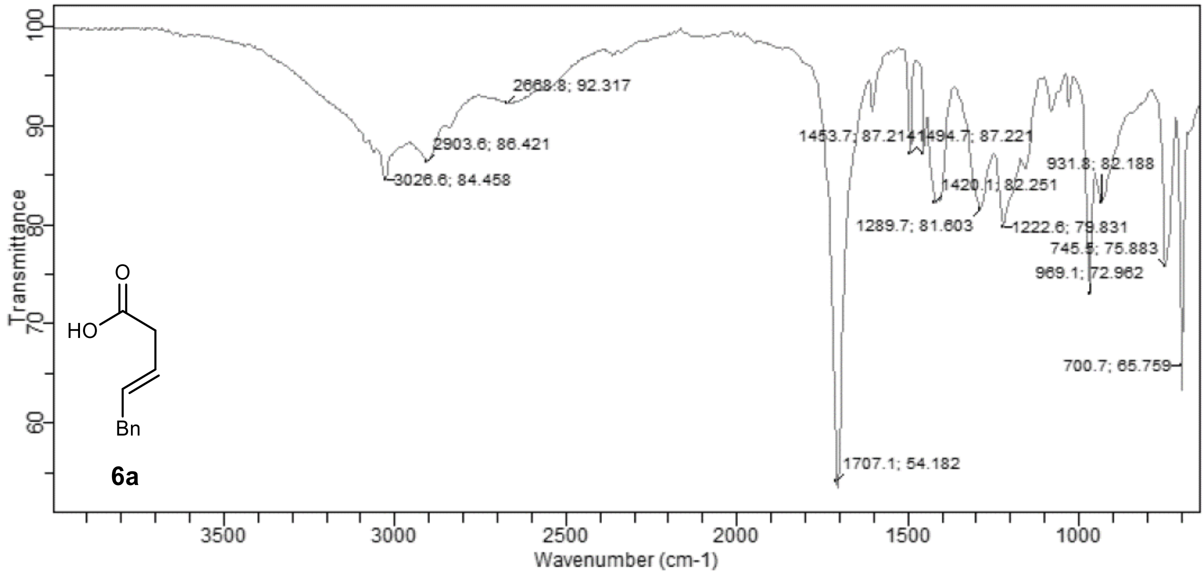

^{1}H NMR (300 MHz, $CDCl_3$) of **6b**

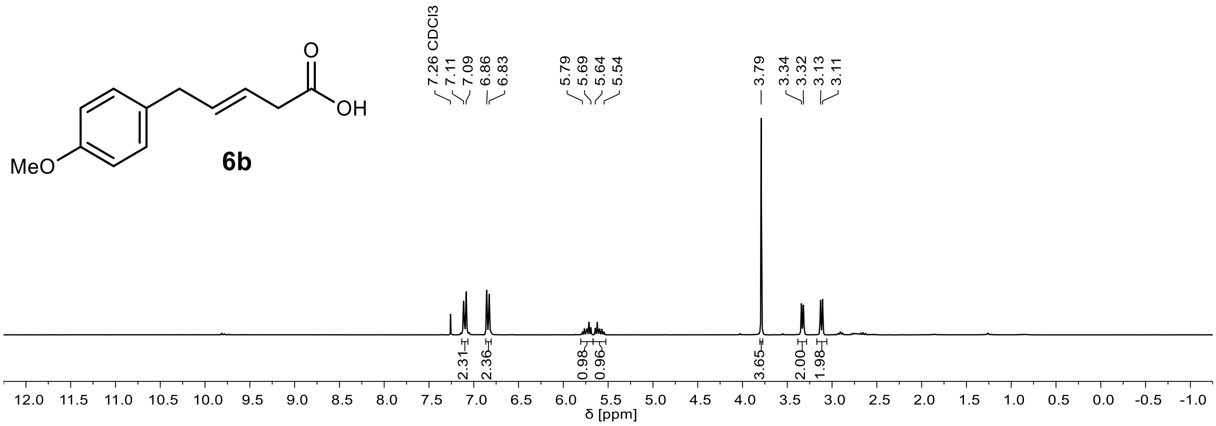

^{13}C NMR (75 MHz, $CDCl_3$) of **6b**

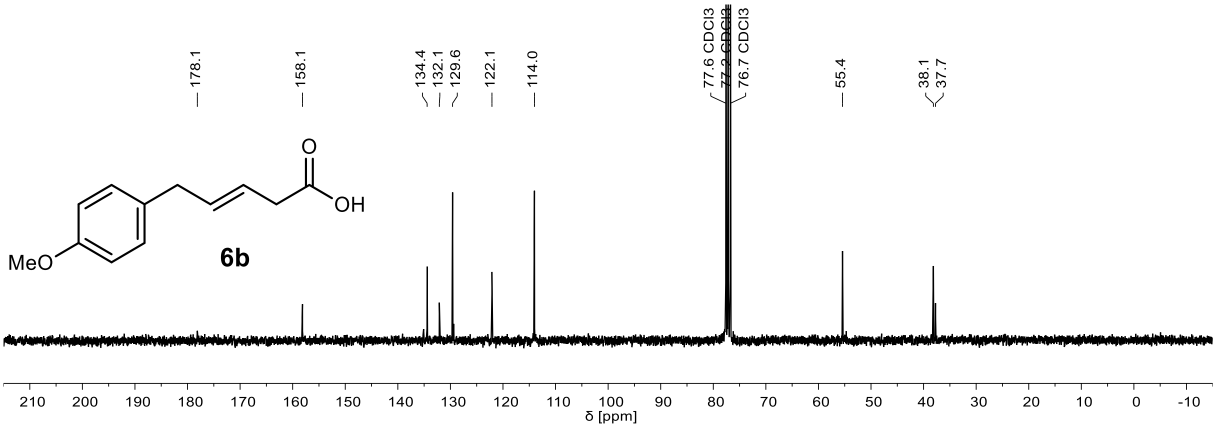

IR (ATR, neat) of **6b**

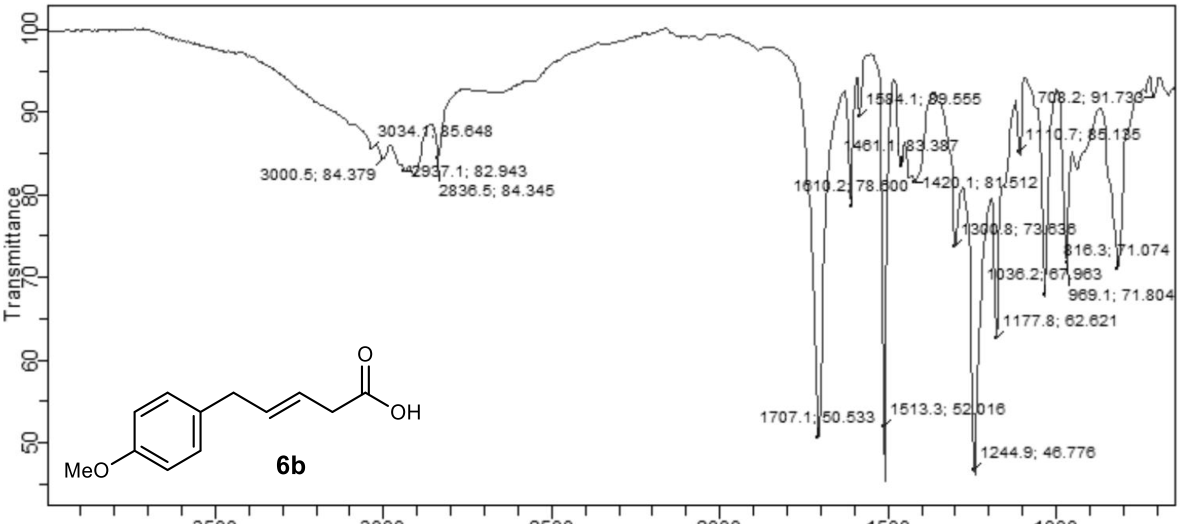

¹H NMR (300 MHz, $CDCl_3$) of **6c**

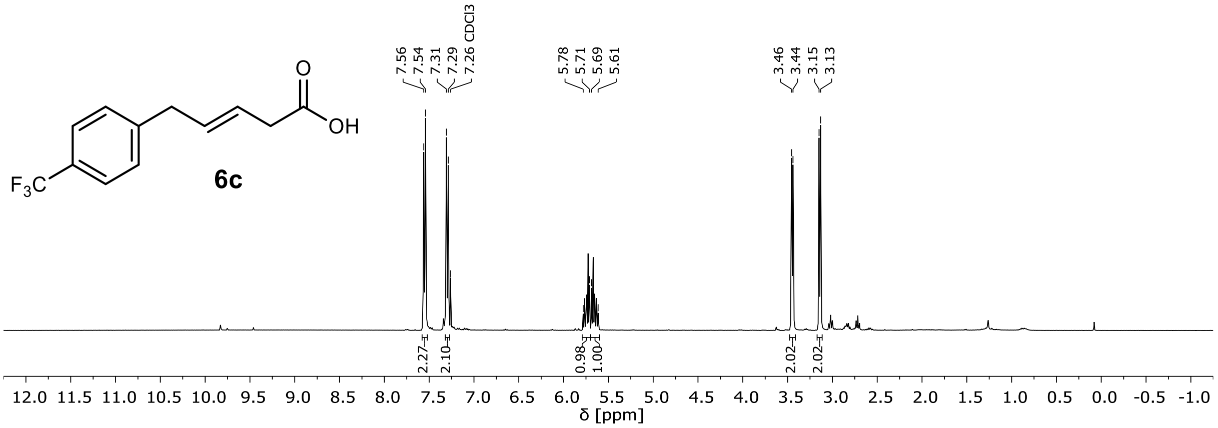

¹³C NMR (75 MHz, $CDCl_3$) of **6c**

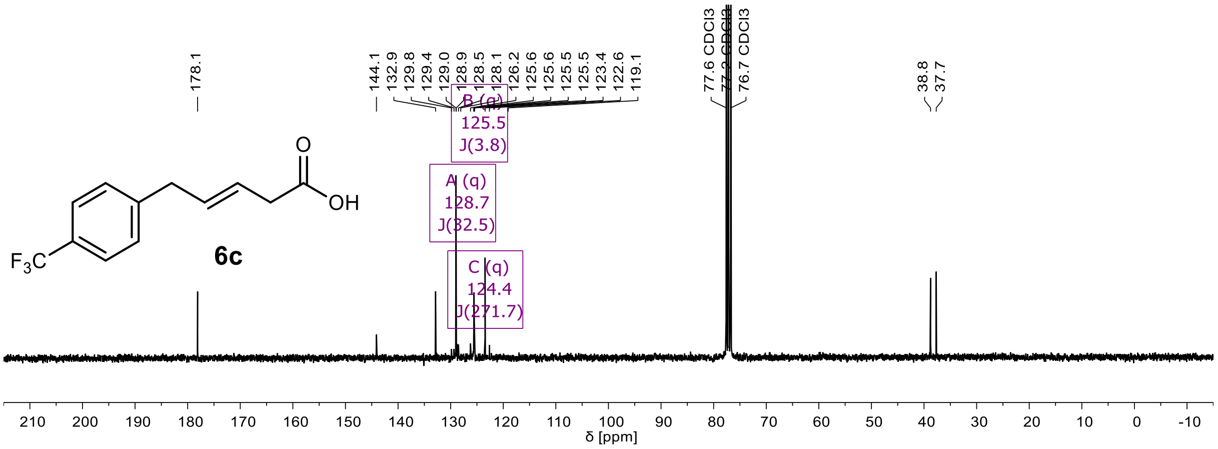

¹⁹F NMR (376 MHz, $CDCl_3$) of **6c**

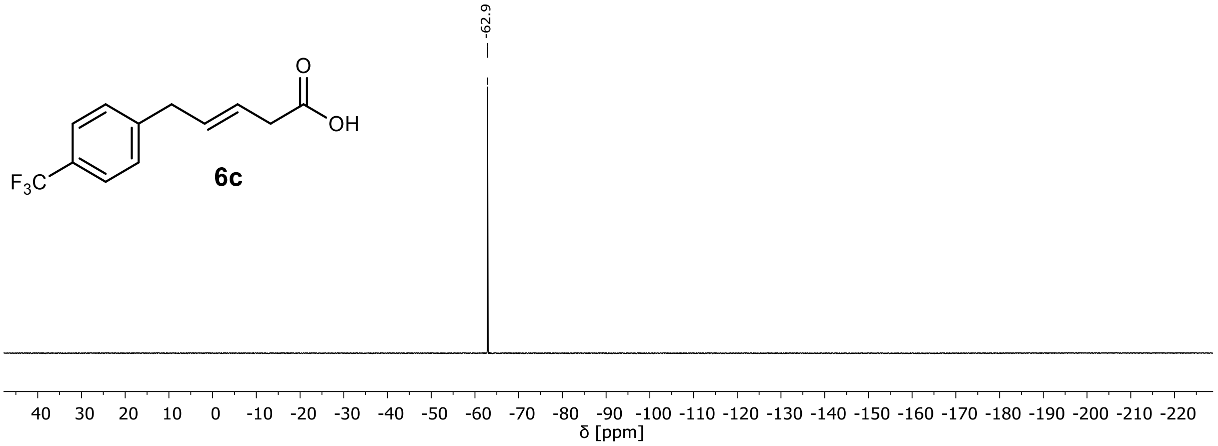

IR (ATR, neat) of **6c**

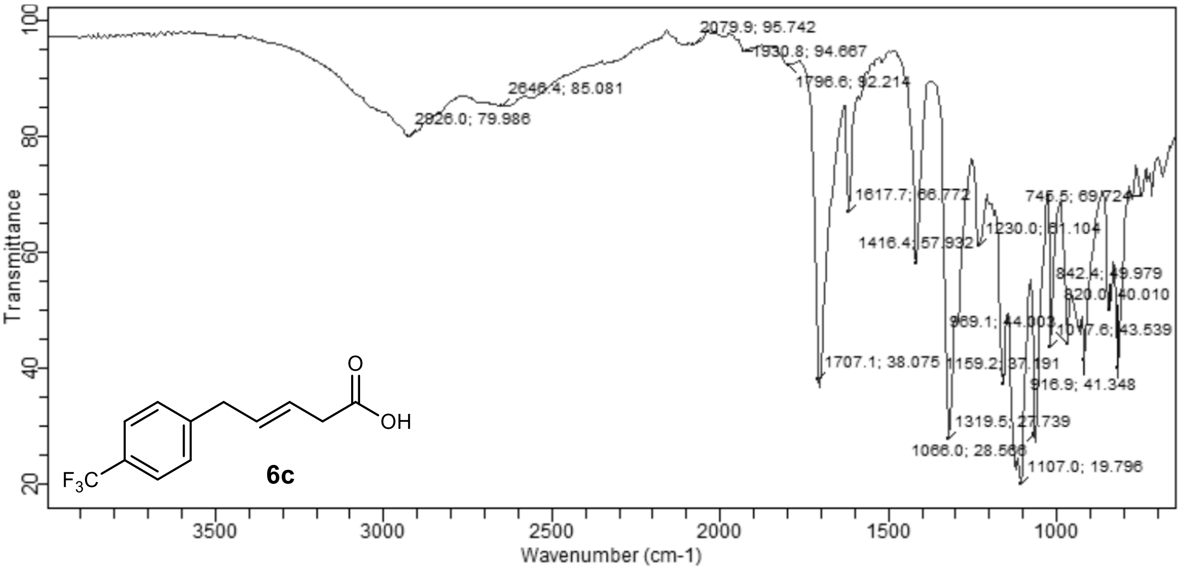

¹H NMR (300 MHz, $CDCl_3$) of **6f**

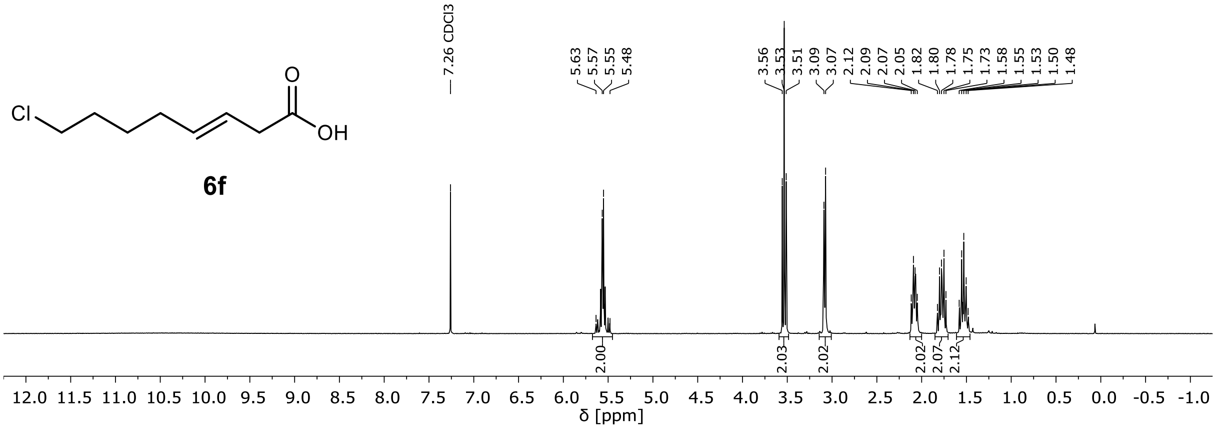

¹³C NMR (75 MHz, $CDCl_3$) of **6f**

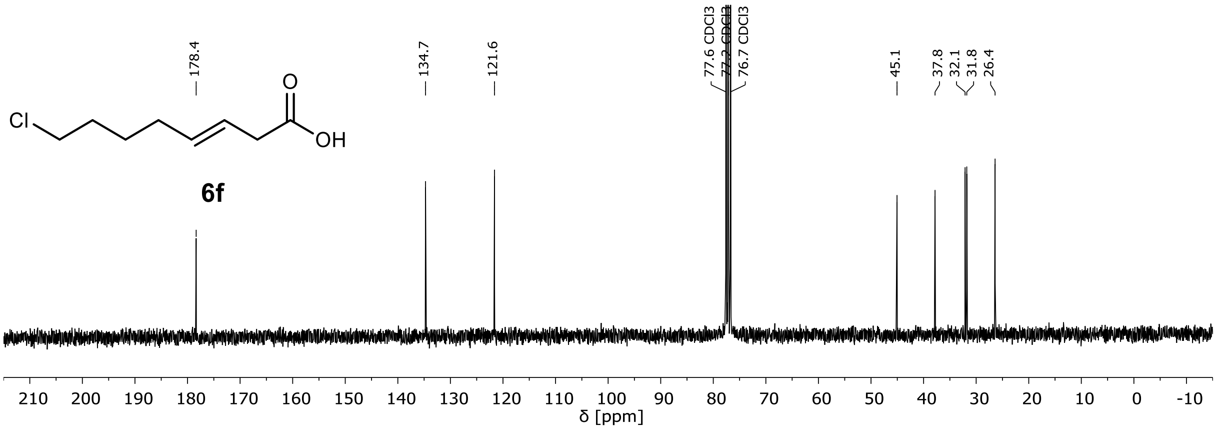

IR (ATR, neat) of **6f**

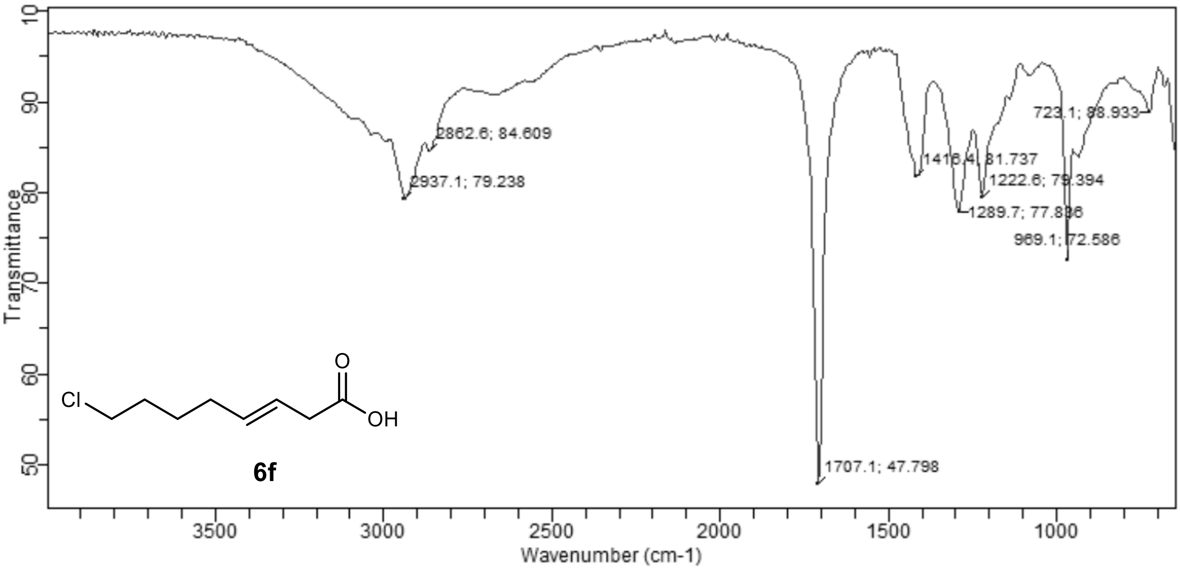

^{1}H NMR (400 MHz, $CDCl_3$) of **10**

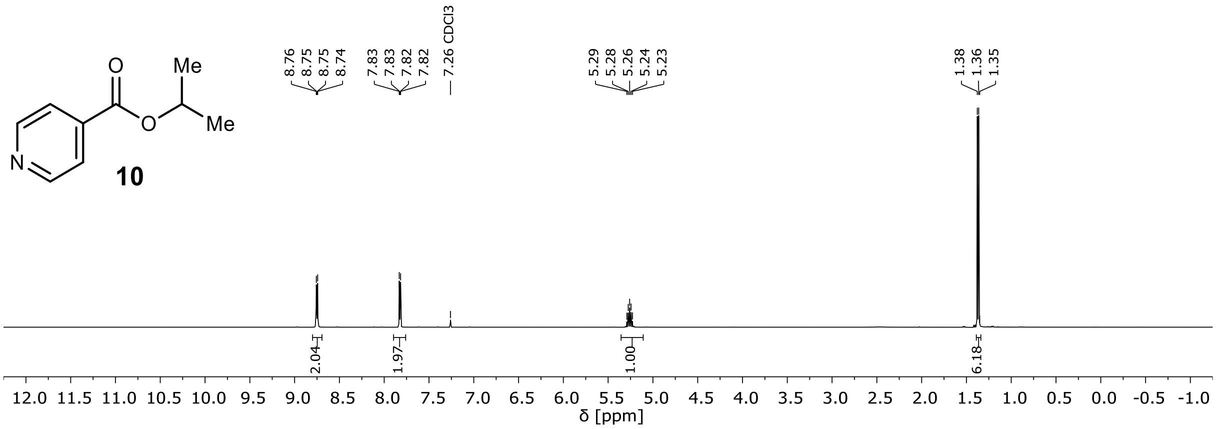

^{13}C NMR (101 MHz, $CDCl_3$) of **10**

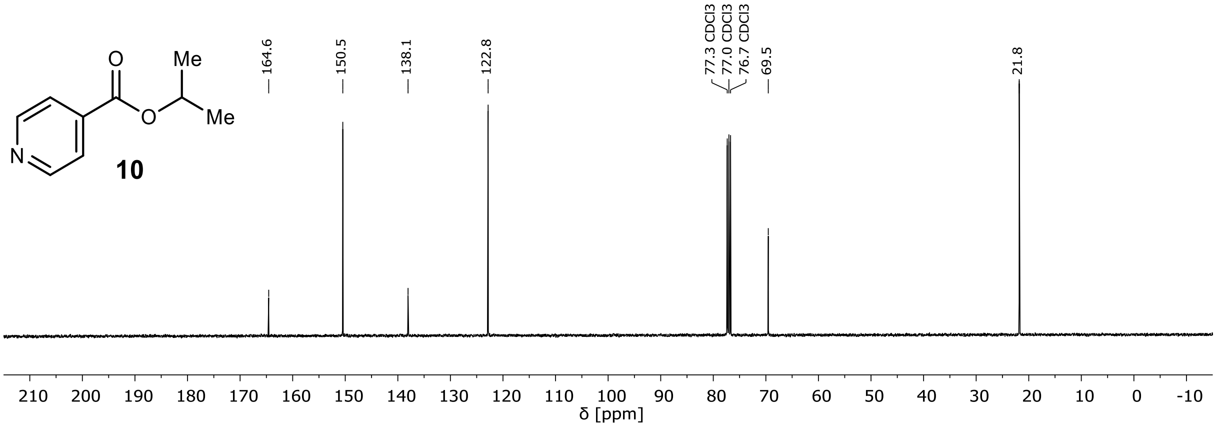

IR (ATR, neat) of **10**

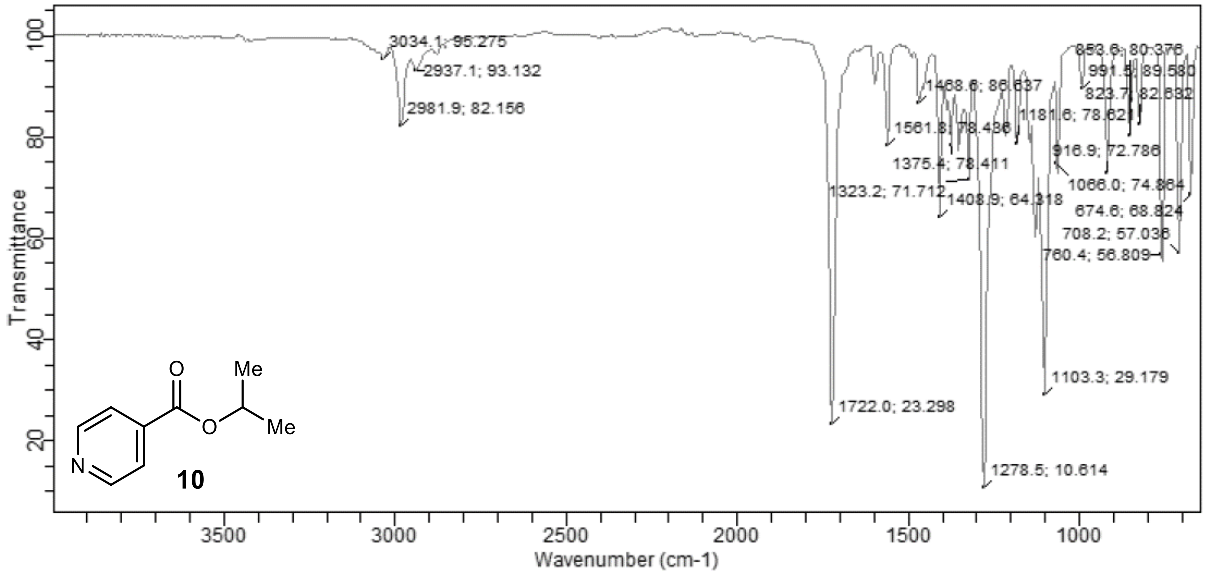

^{1}H NMR (400 MHz, $CDCl_3$) of **11**

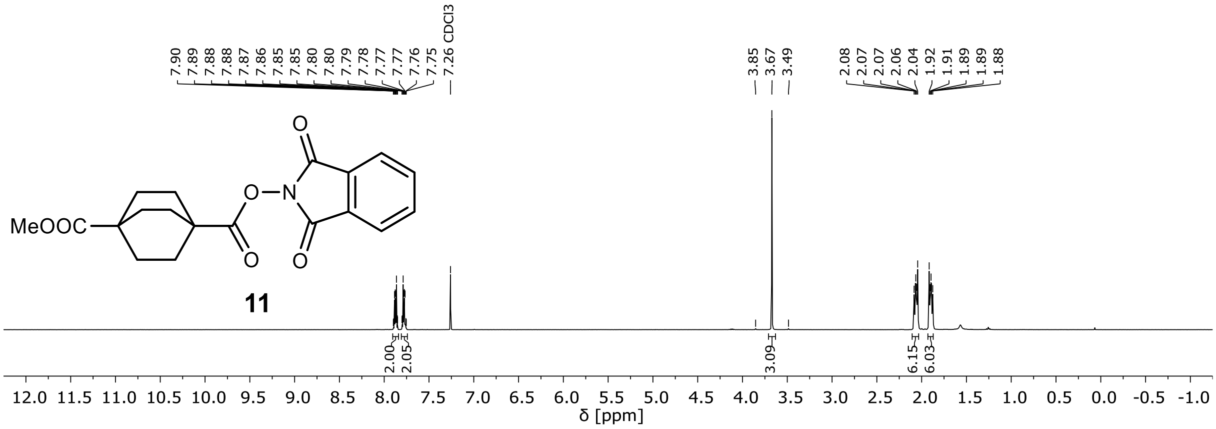

^{13}C NMR (101 MHz, $CDCl_3$) of **11**

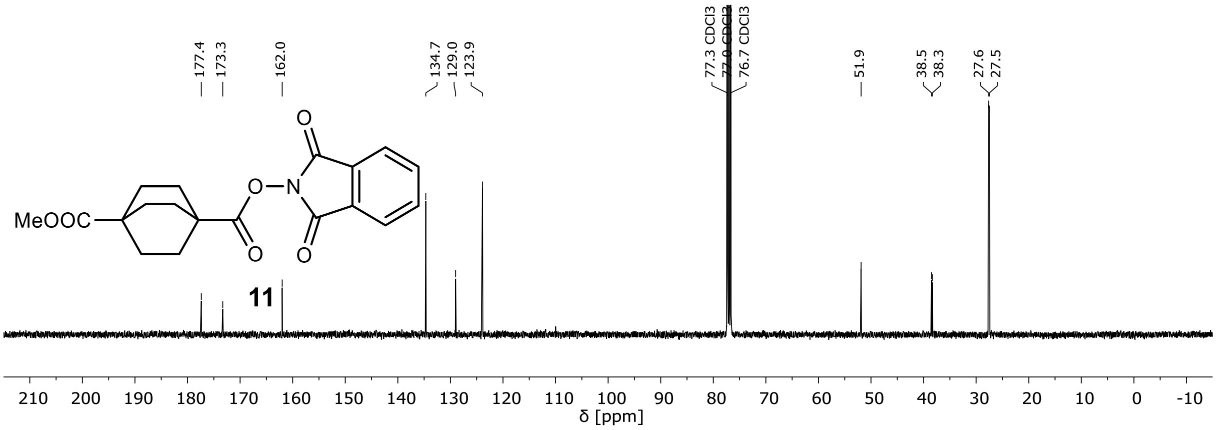

IR (ATR, neat) of **11**

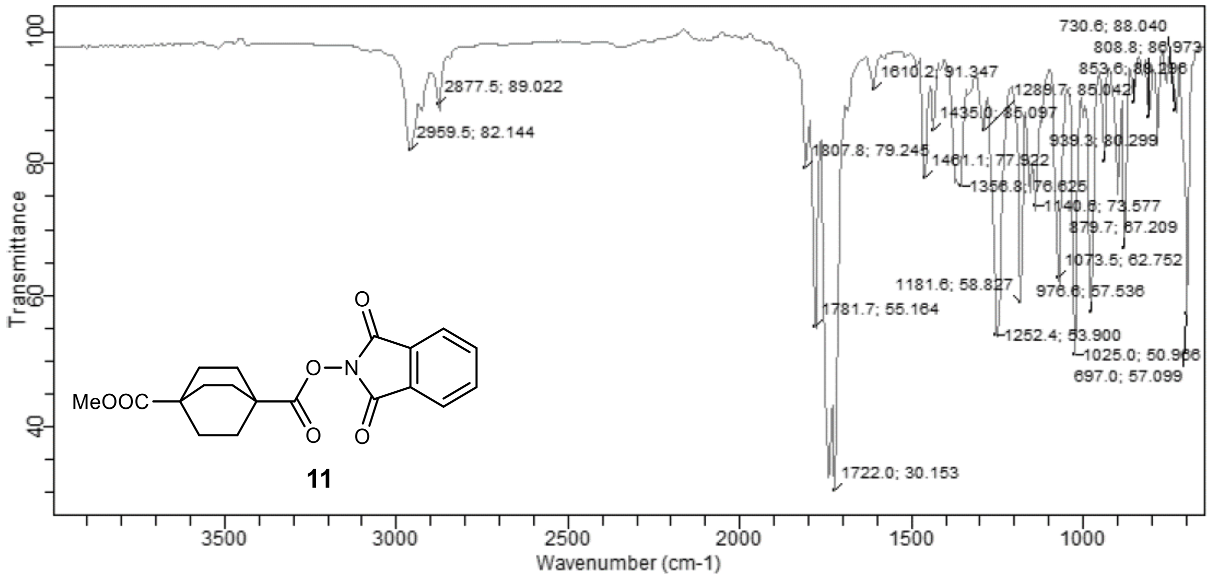

¹H NMR (400 MHz, $CDCl_3$) of **2f**

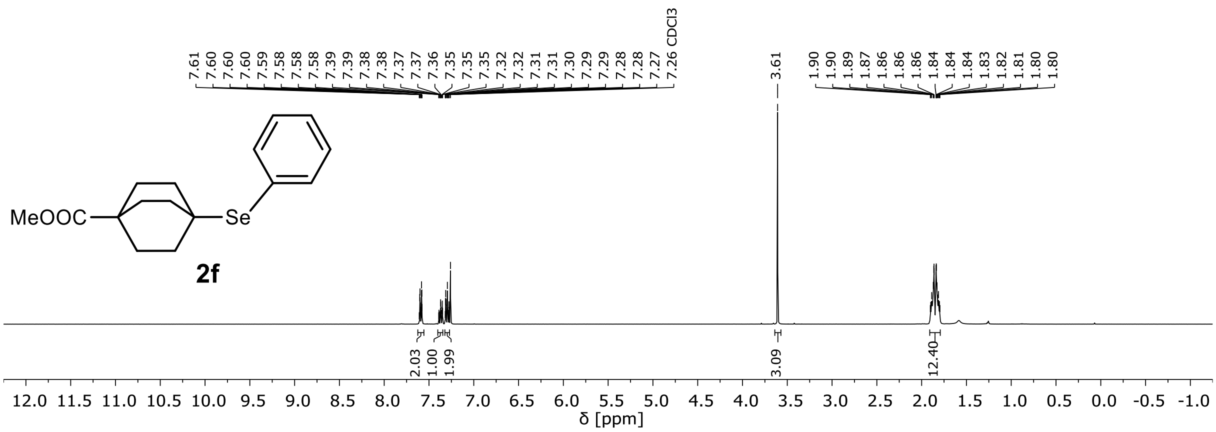

¹³C NMR (101 MHz, $CDCl_3$) of **2f**

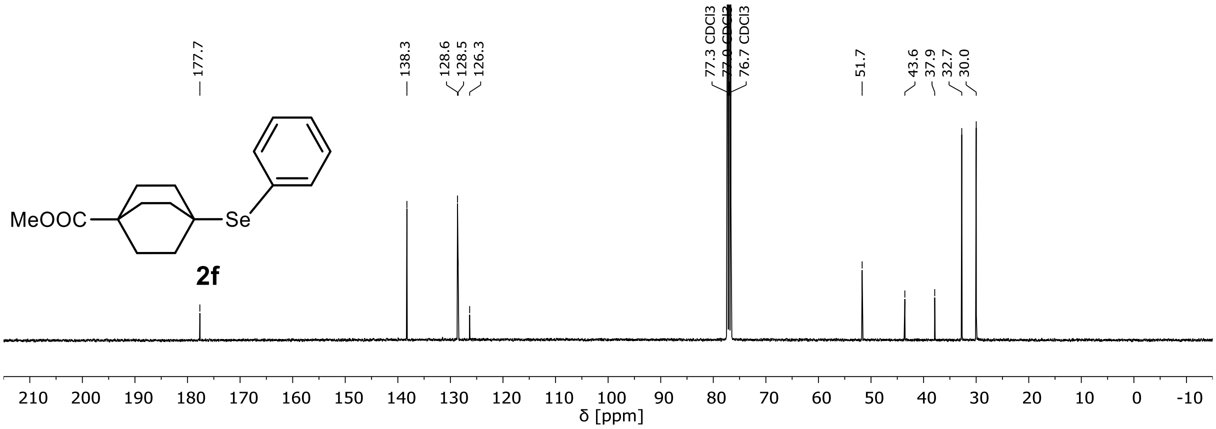

^{77}Se NMR (76 MHz, $CDCl_3$) of **2f**

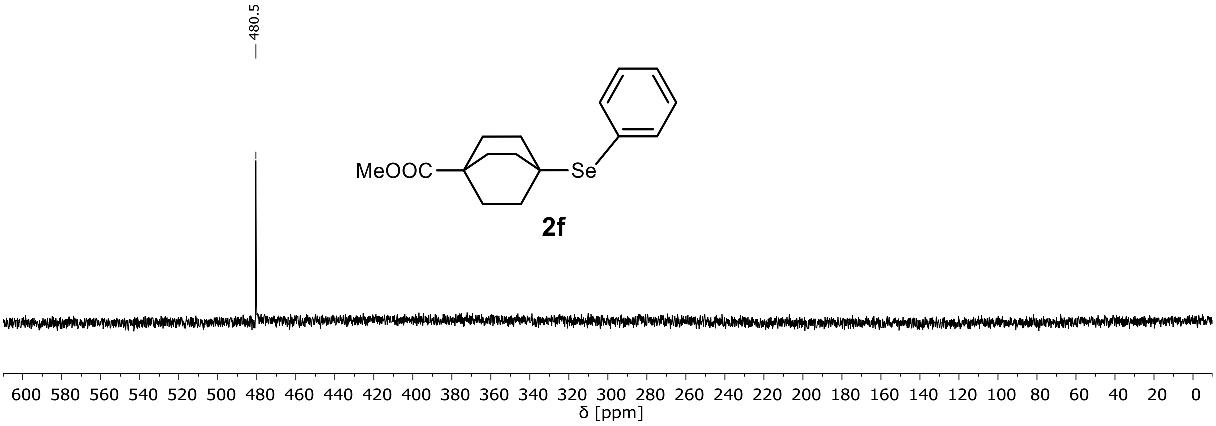

IR (ATR, neat) of **2f**

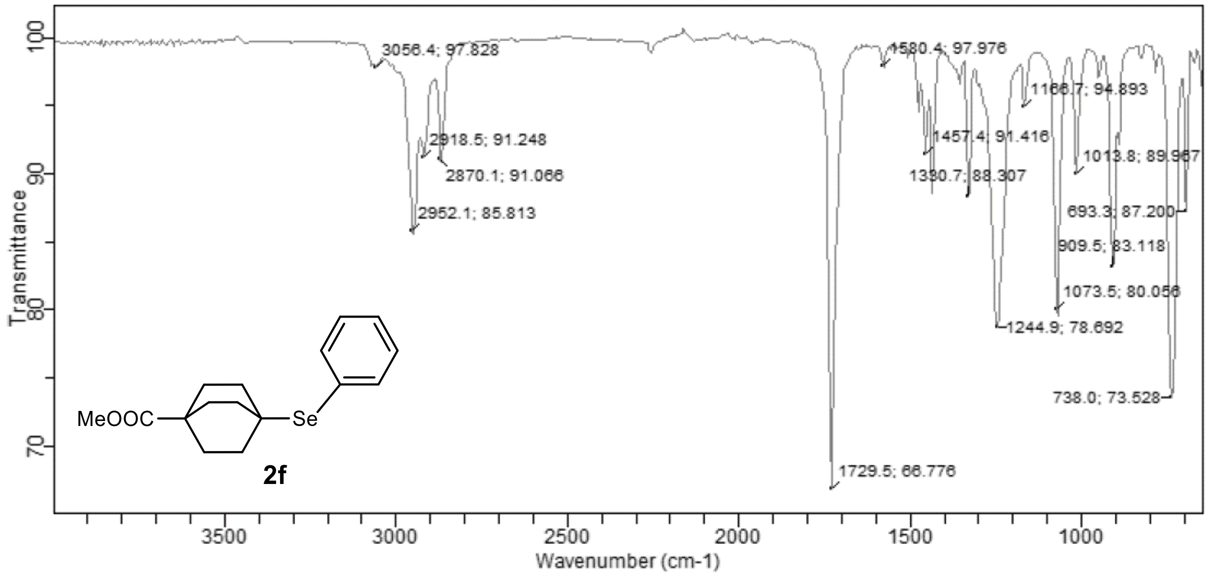

^{1}H NMR (300 MHz, $CDCl_3$) of **7a**

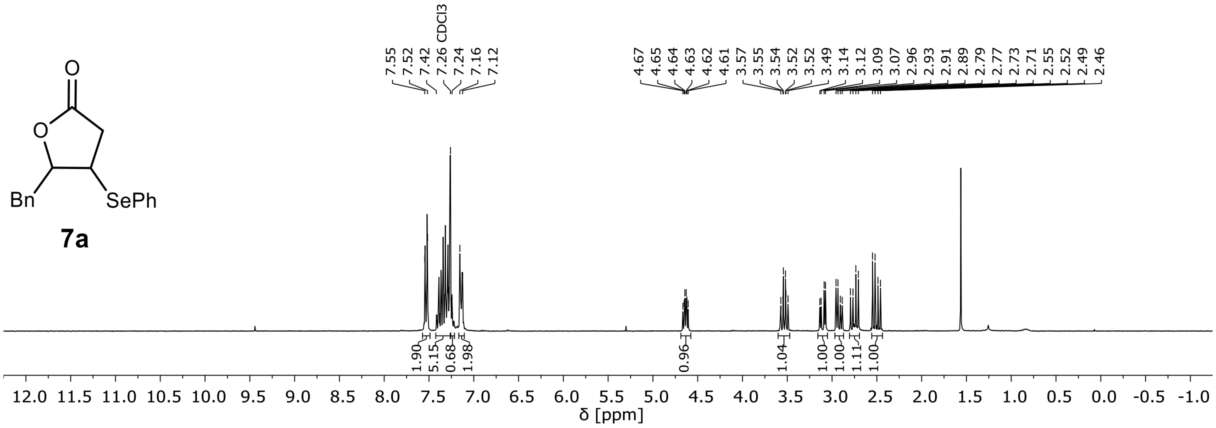

^{13}**C NMR** (101 MHz, $CDCl_3$) of **7a**

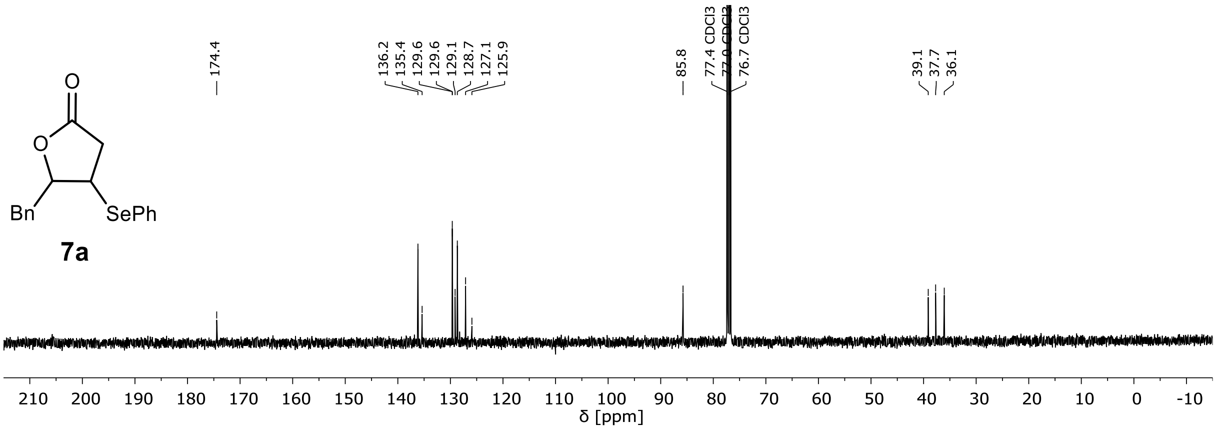

^{77}**Se NMR** (76 MHz, $CDCl_3$) of **7a**

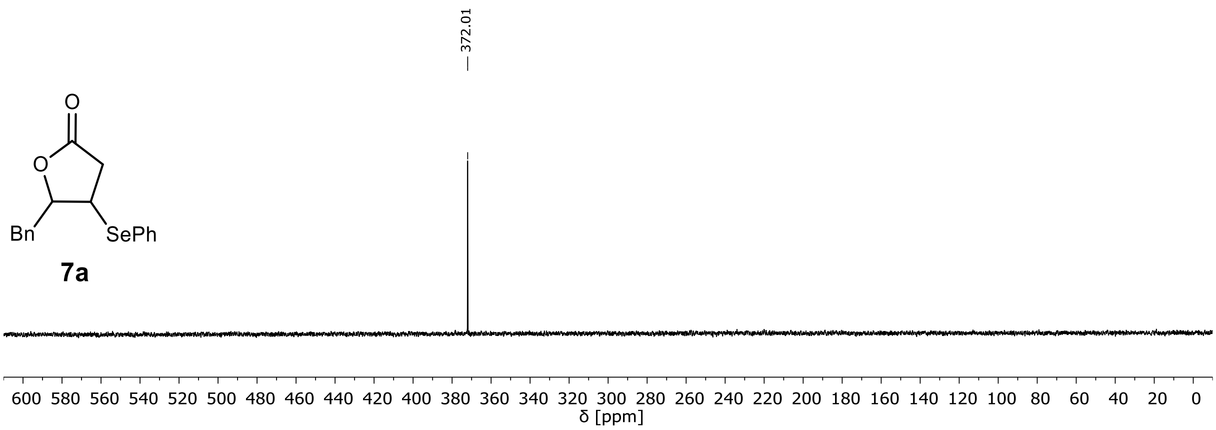

IR (ATR, neat) of **7a**

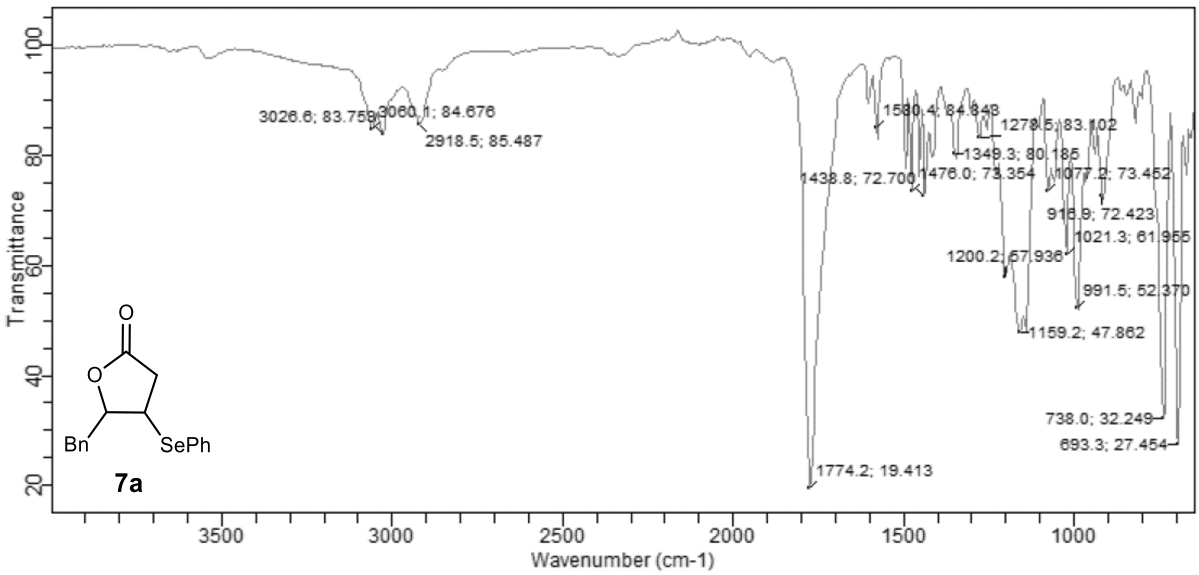

^{1}H NMR (300 MHz, $CDCl_3$) of **8a**

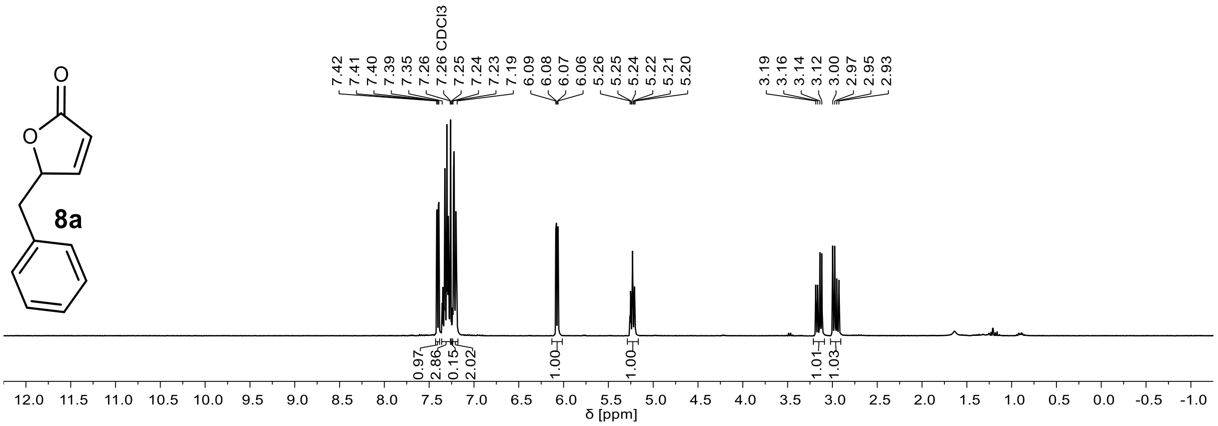

^{13}C NMR (75 MHz, $CDCl_3$) of **8a**

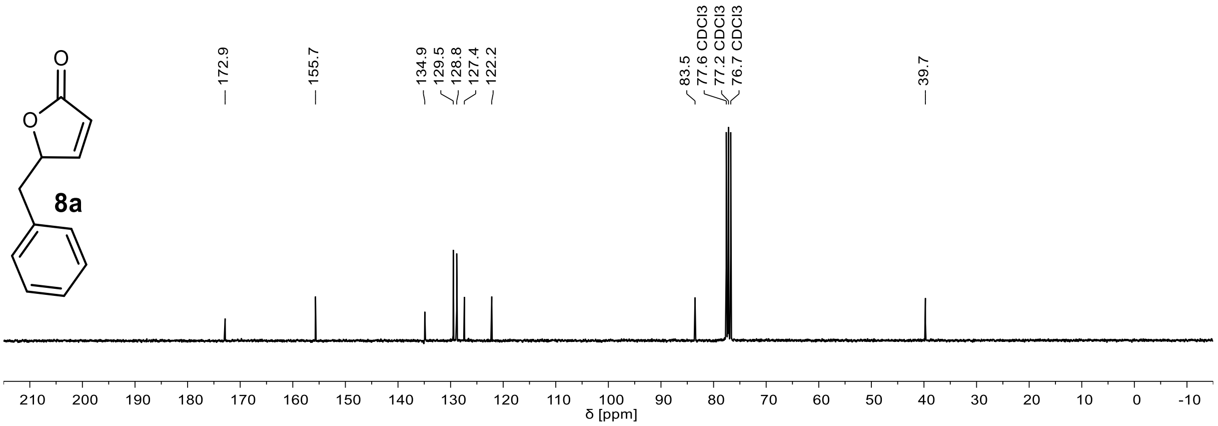

IR (ATR, neat) of **8a**

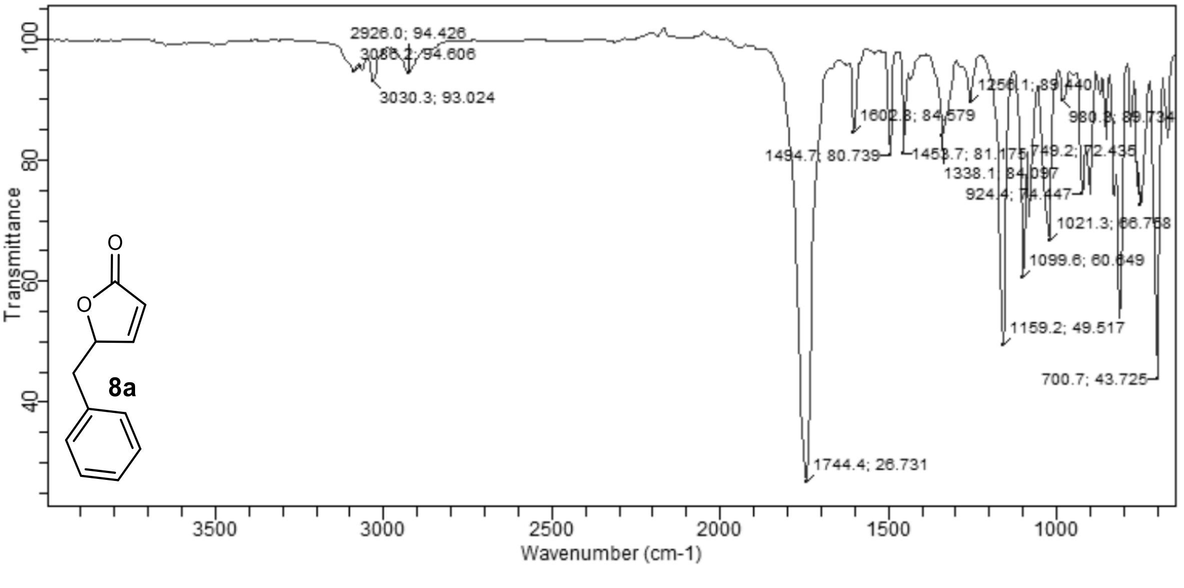

^{1}H NMR (300 MHz, $CDCl_3$) of **8b**

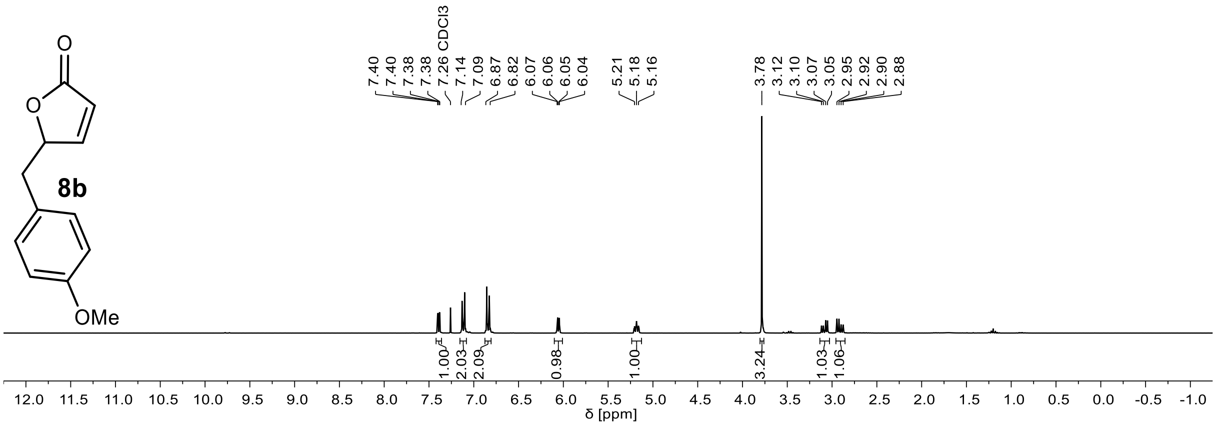

^{13}C NMR (101 MHz, $CDCl_3$) of **8b**

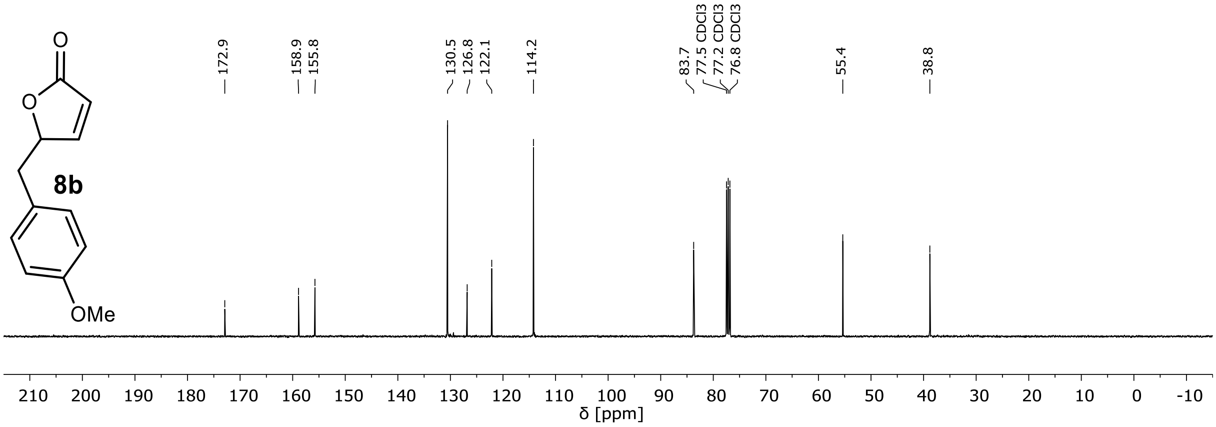

IR (ATR, neat) of **8b**

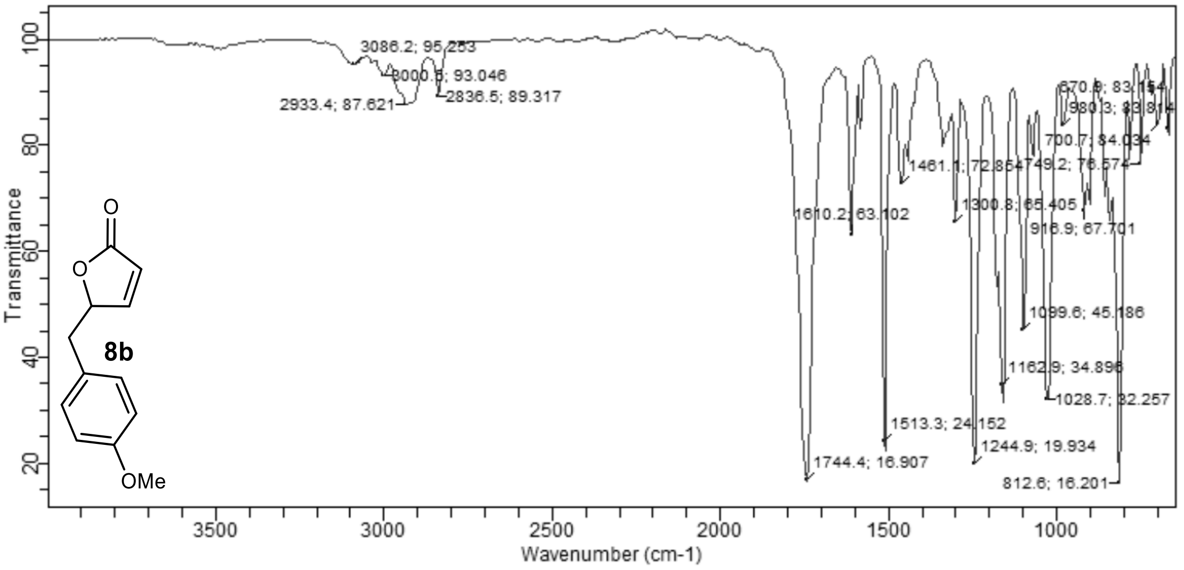

^{1}H NMR (300 MHz, $CDCl_3$) of **8c**

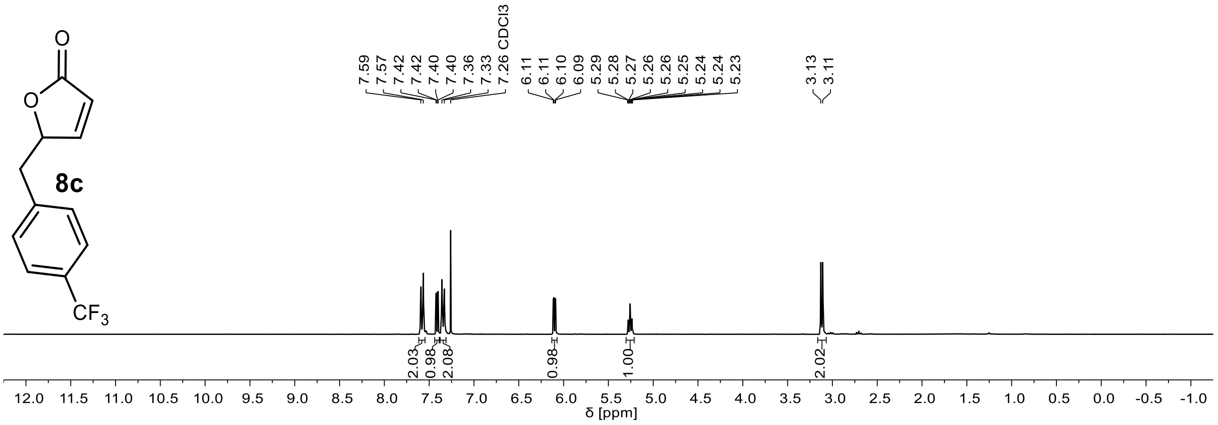

^{13}C NMR (101 MHz, $CDCl_3$) of **8c**

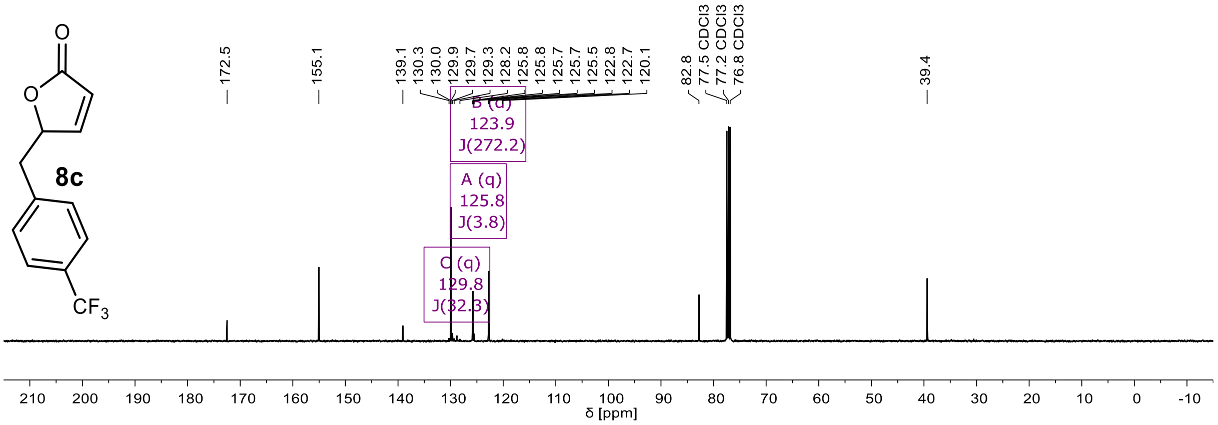

^{19}F NMR (376 MHz, $CDCl_3$) of **8c**

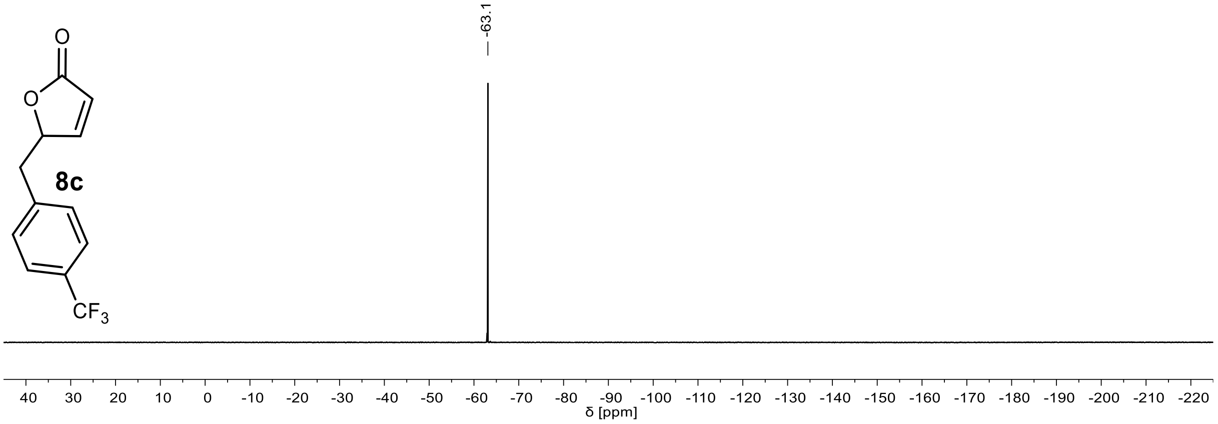

IR (ATR, neat) of **8c**

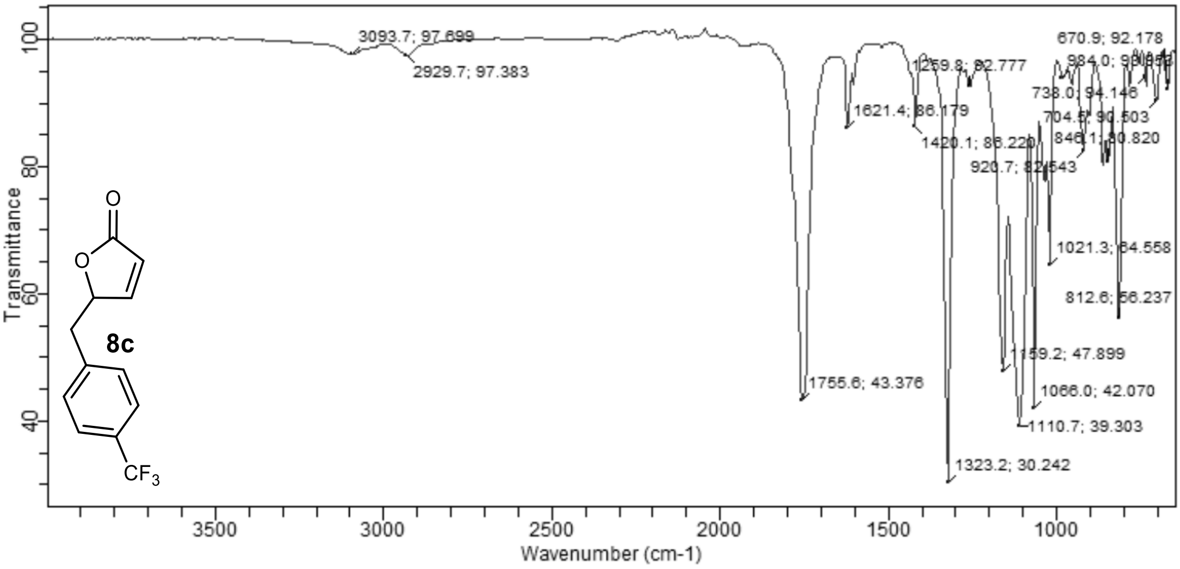

^{1}**H NMR** (300 MHz, $CDCl_3$) of **8d**

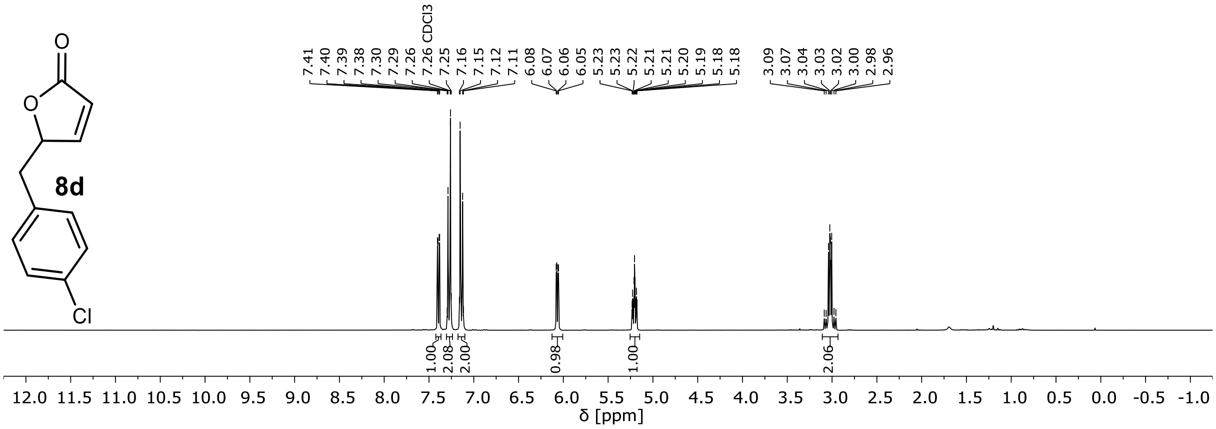

^{13}**C NMR** (75 MHz, $CDCl_3$) of **8d**

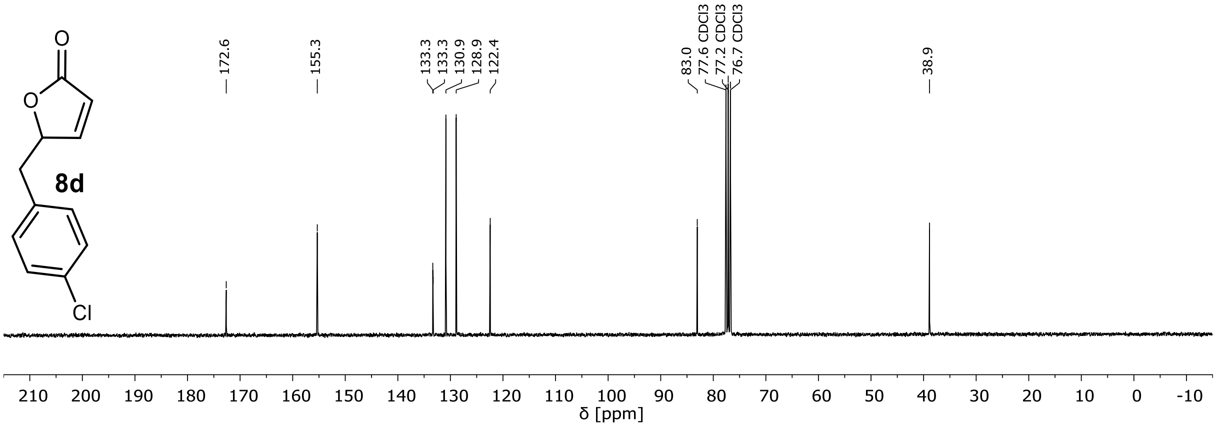

IR (ATR, neat) of **8d**

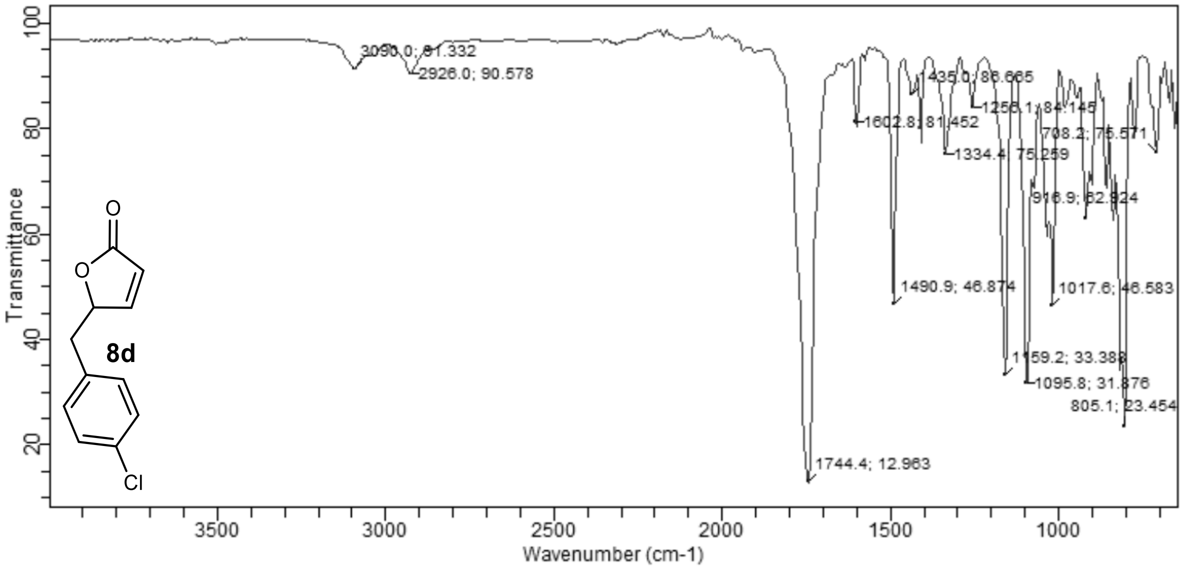

^{1}H NMR (300 MHz, $CDCl_3$) of **8e**

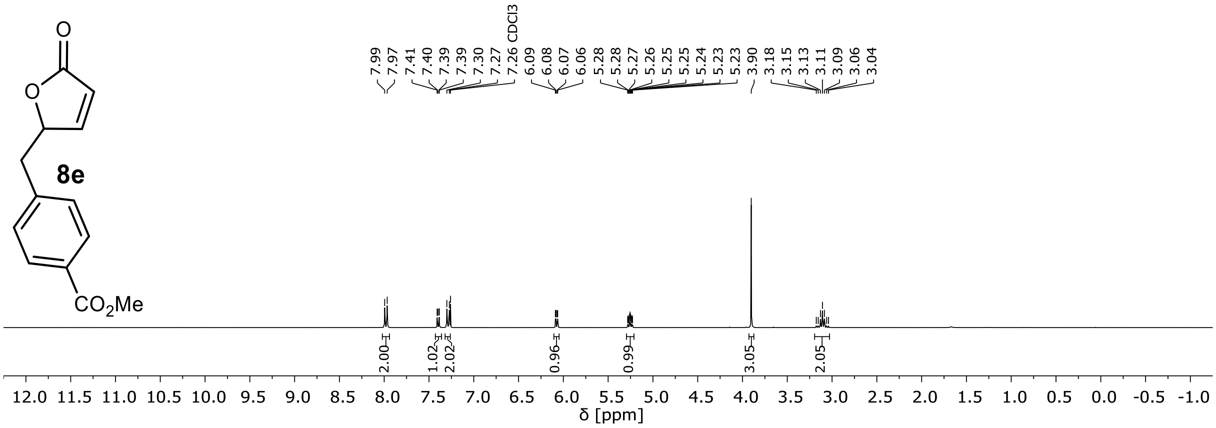

^{13}C NMR (75 MHz, $CDCl_3$) of **8e**

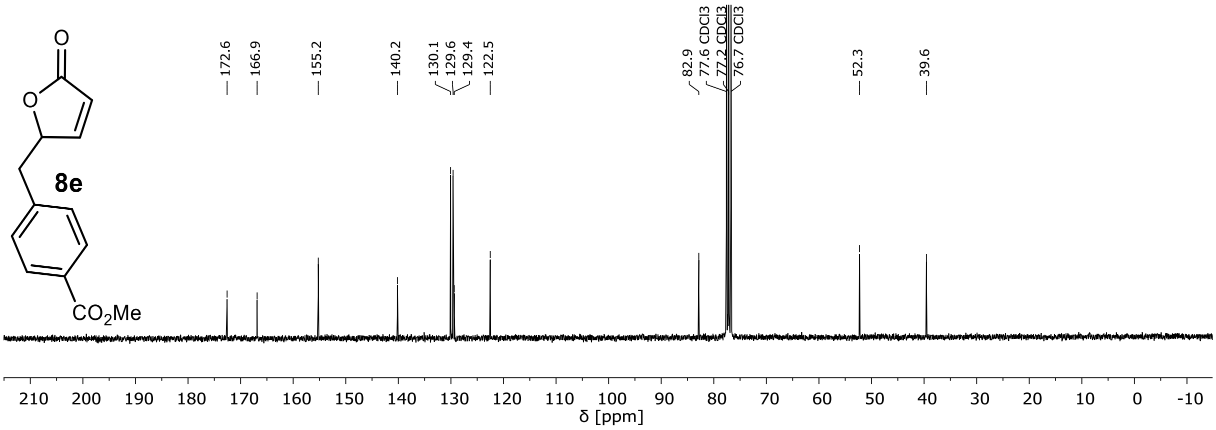

IR (ATR, neat) of **8e**

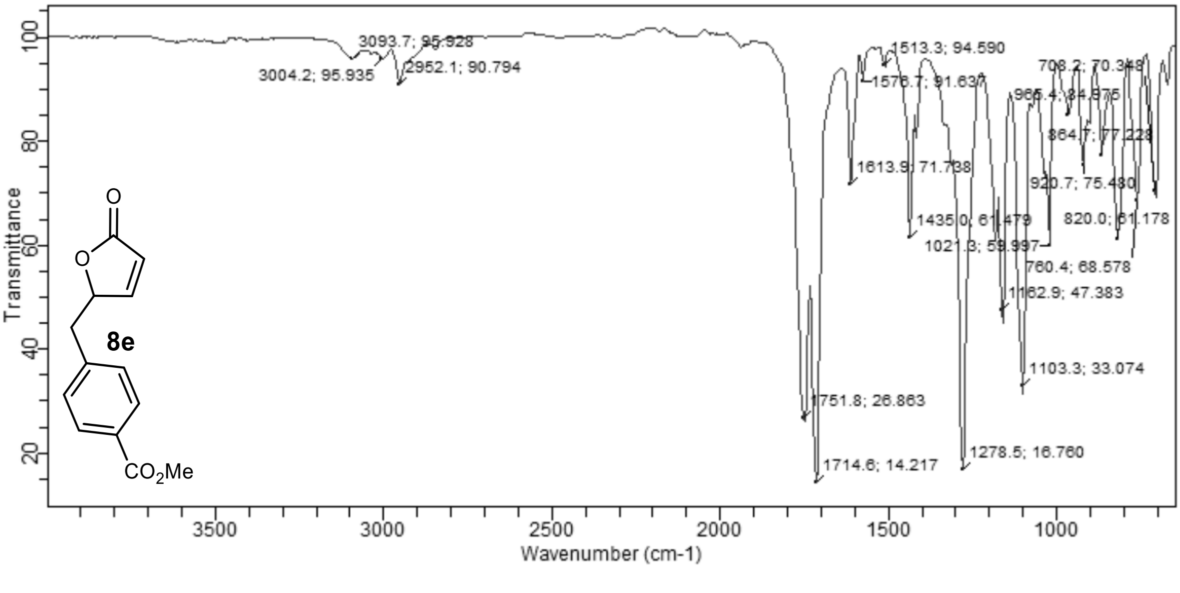

^{1}H NMR (300 MHz, $CDCl_3$) of **8f**

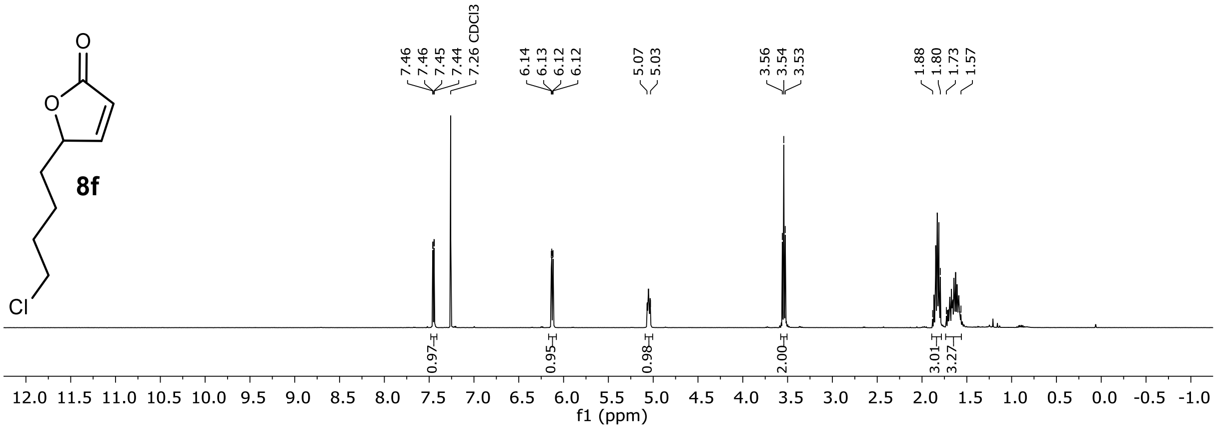

^{13}C NMR (75 MHz, $CDCl_3$) of **8f**

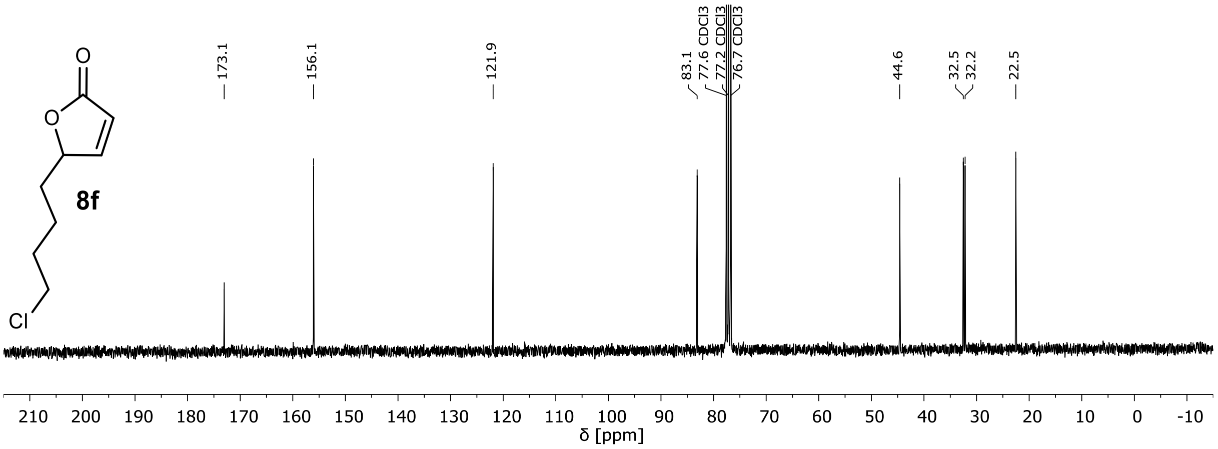

IR (ATR, neat) of **8f**

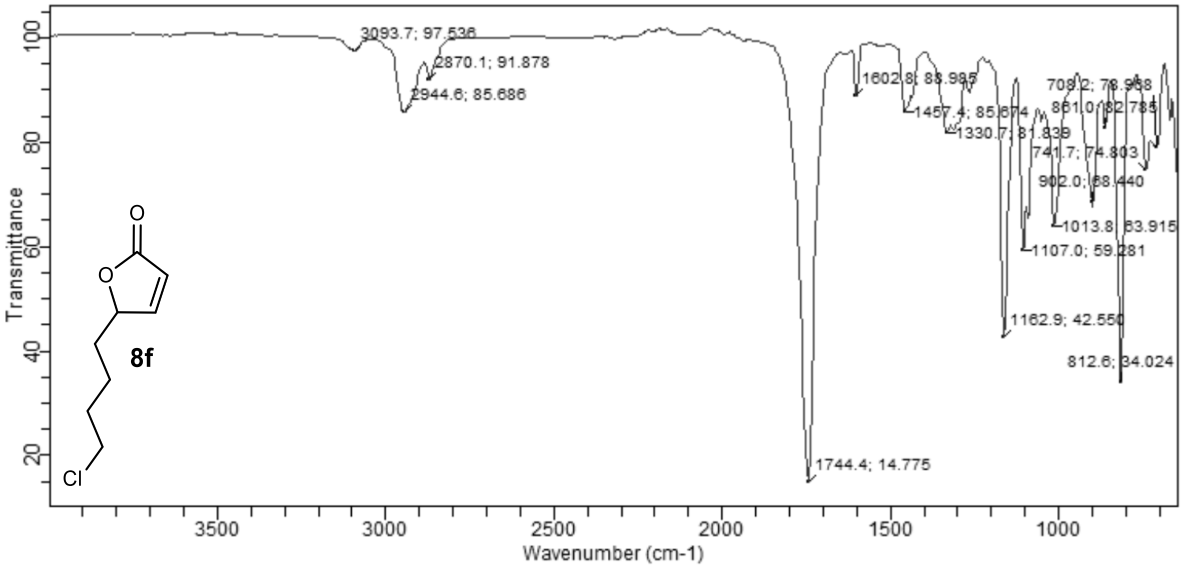

^{1}H NMR (400 MHz, $CDCl_3$) of **4aa** (N^2)

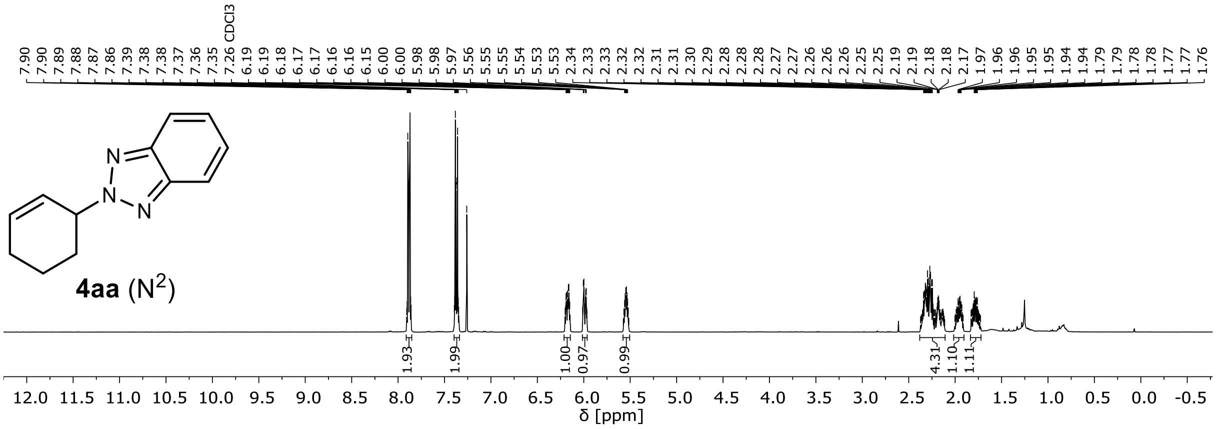

^{13}C NMR (101 MHz, $CDCl_3$) of **4aa** (N^2)

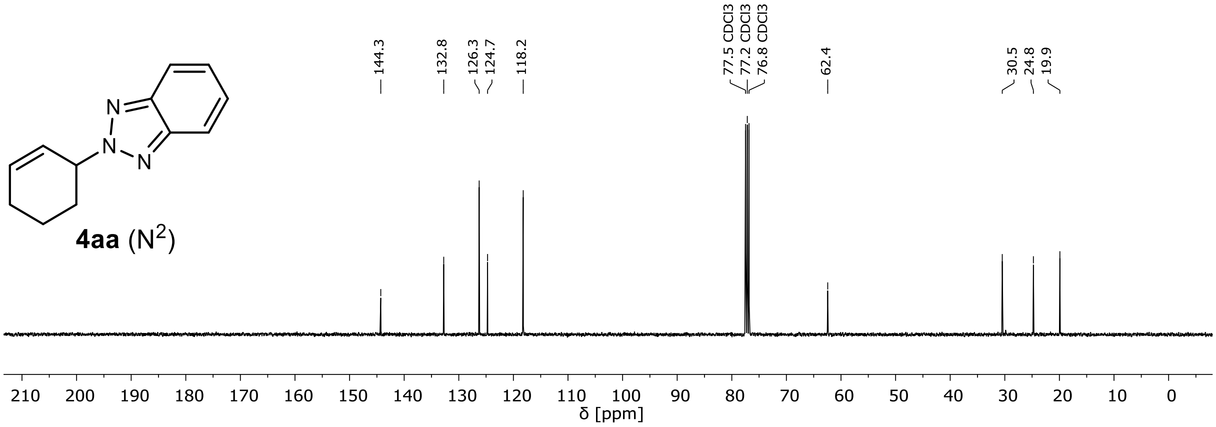

IR (ATR, neat) of **4aa** (N^2)

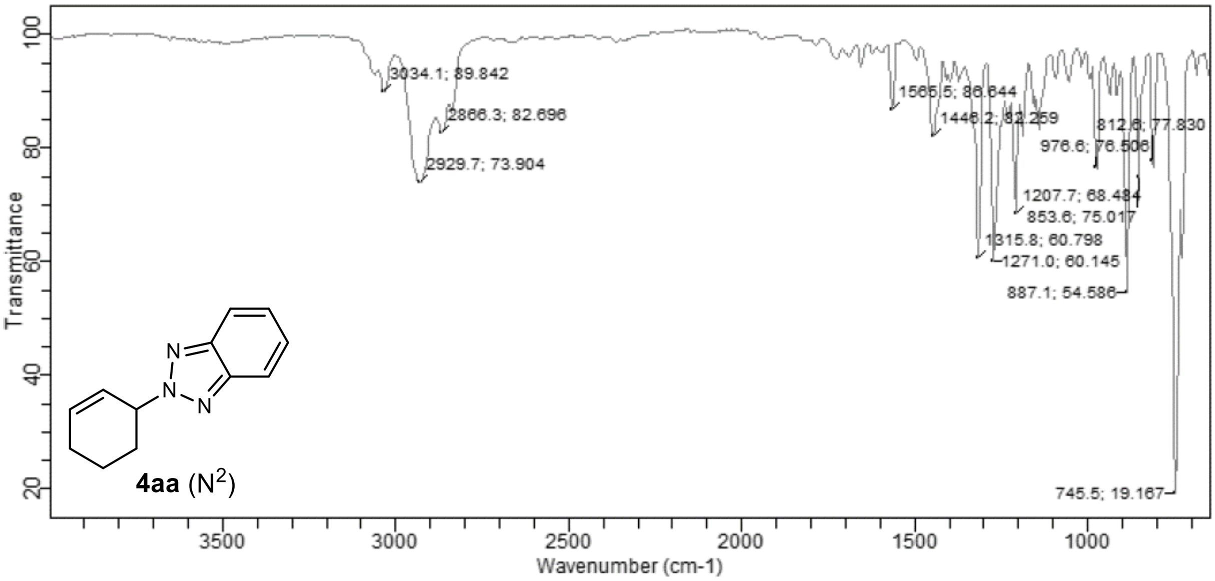

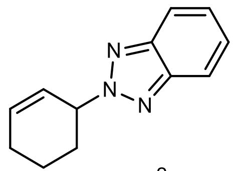

4aa (N^2)

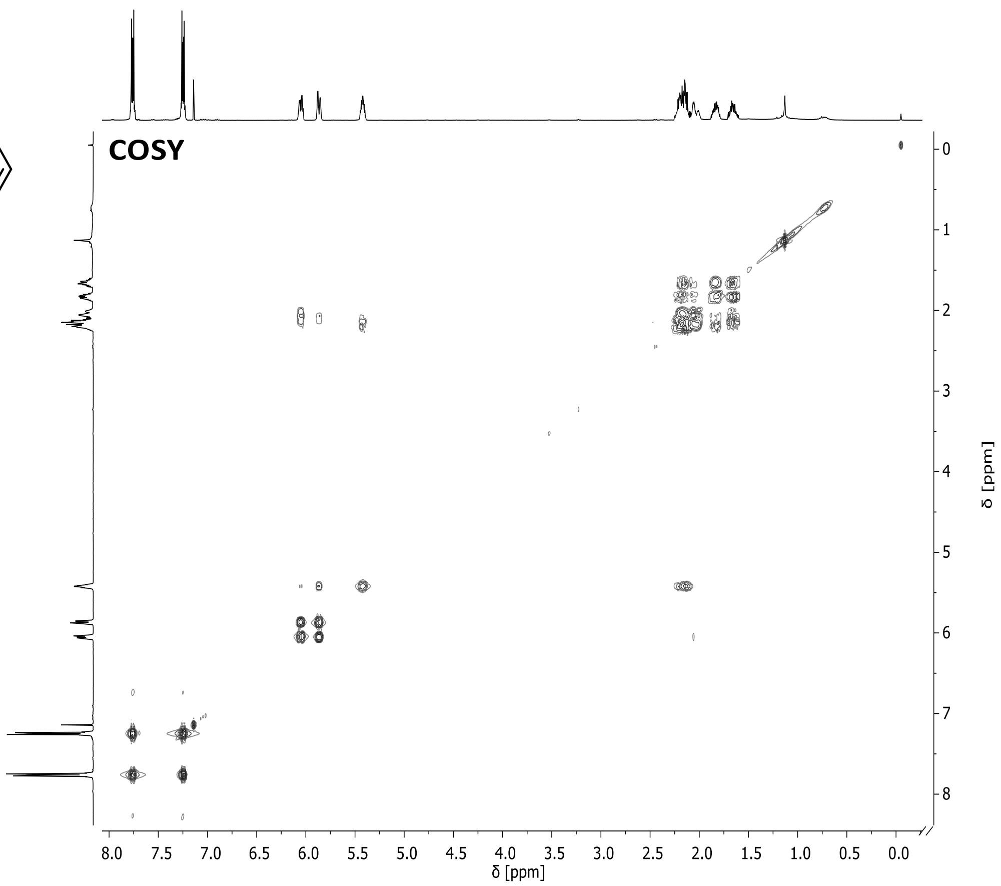

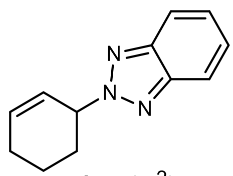

4aa (N^2)

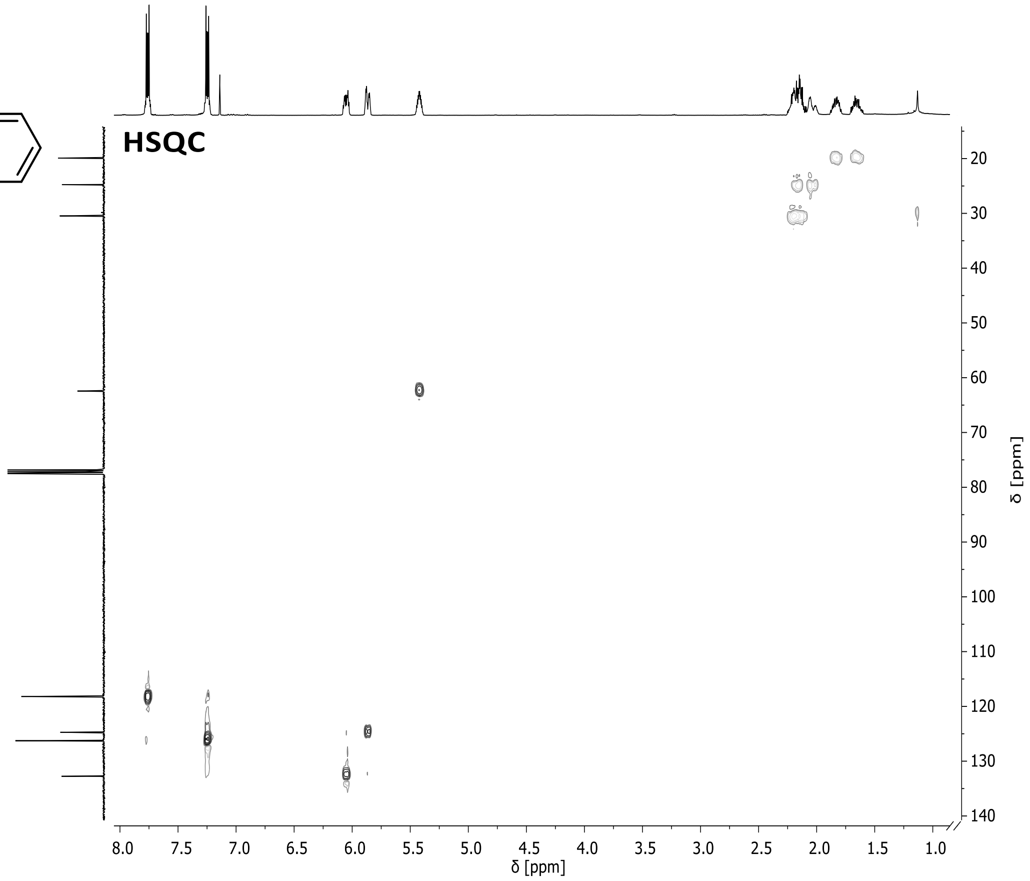

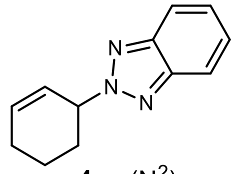

4aa (N²)

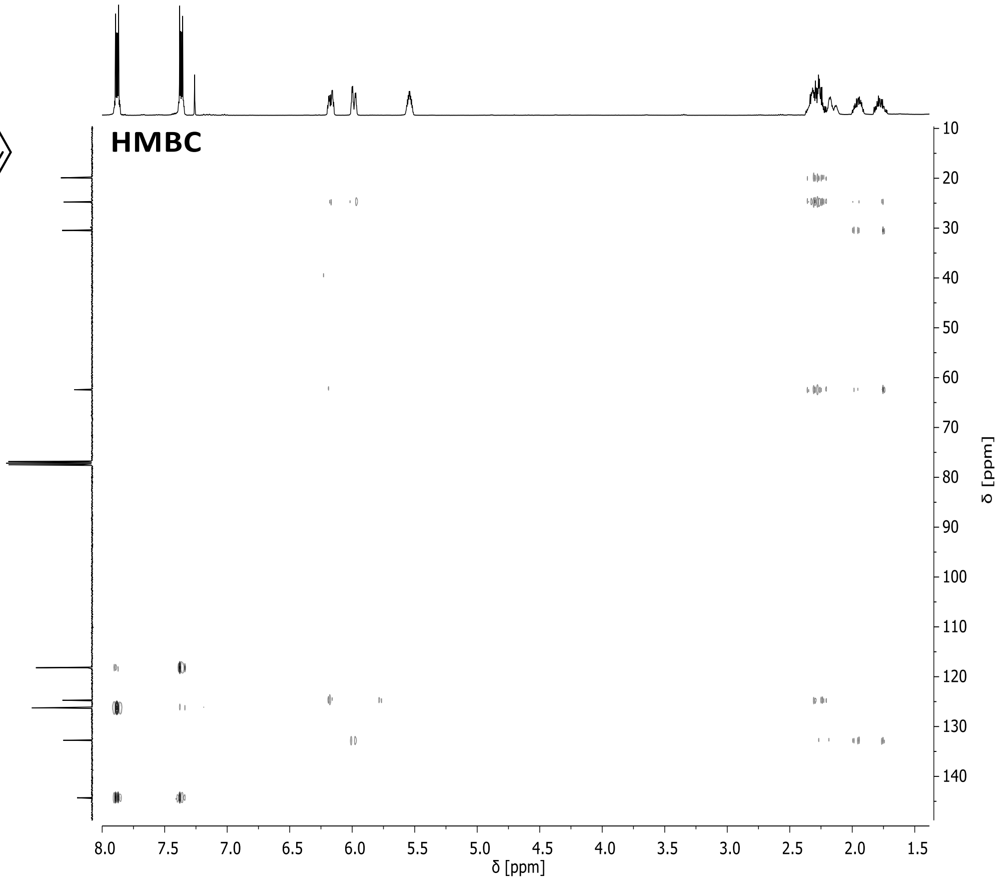

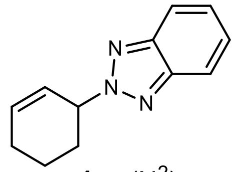

4aa (N²)

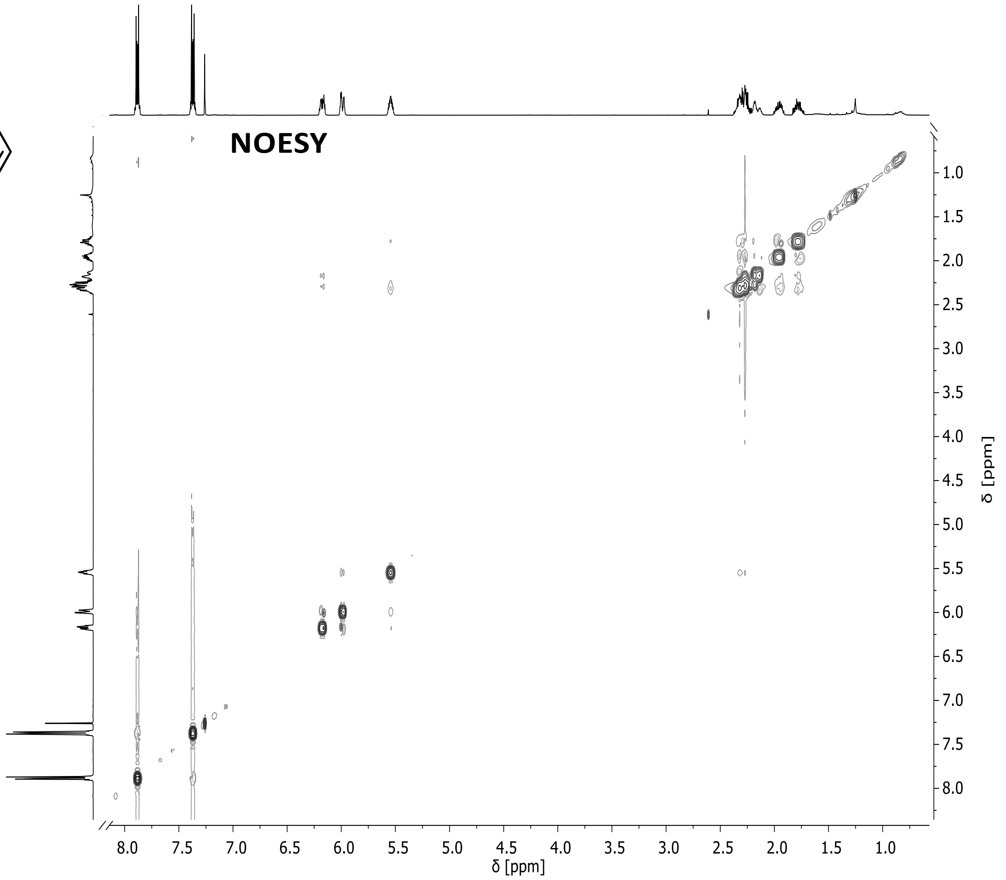

^{1}H NMR (400 MHz, $CDCl_3$) of **4aa** (N^1)

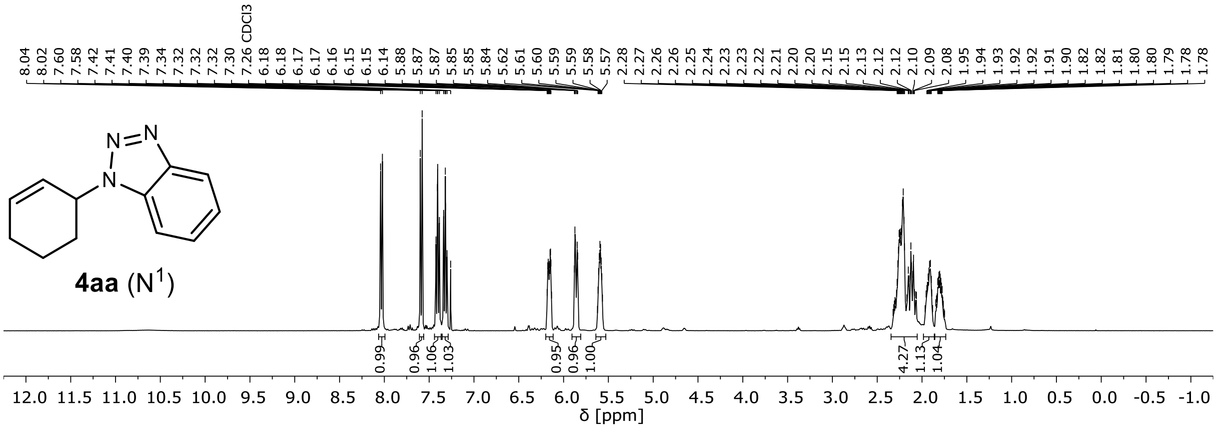

^{13}C NMR (101 MHz, $CDCl_3$) of **4aa** (N^1)

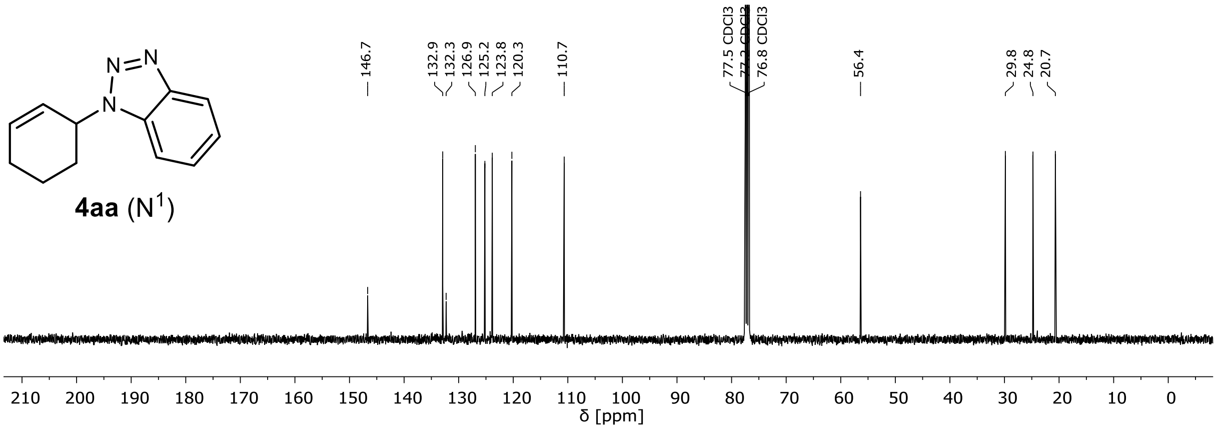

IR (ATR, neat) of **4aa** (N^1)

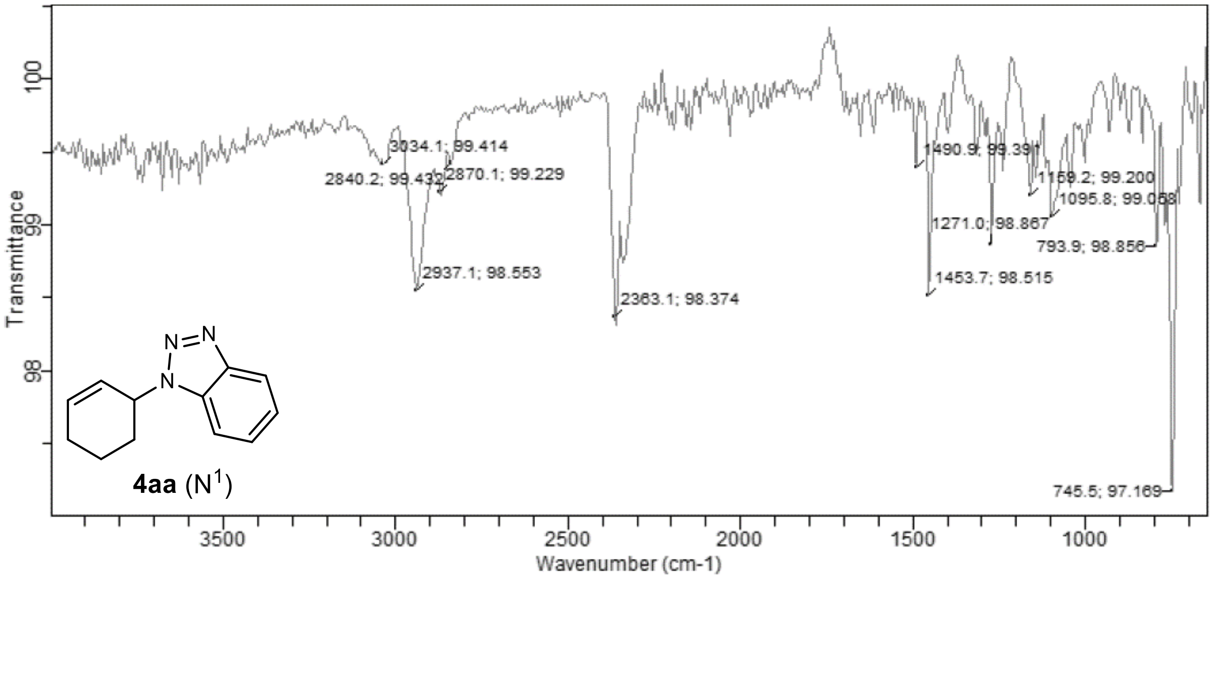

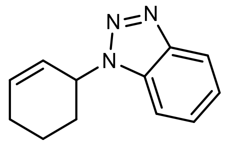

4aa (N1)

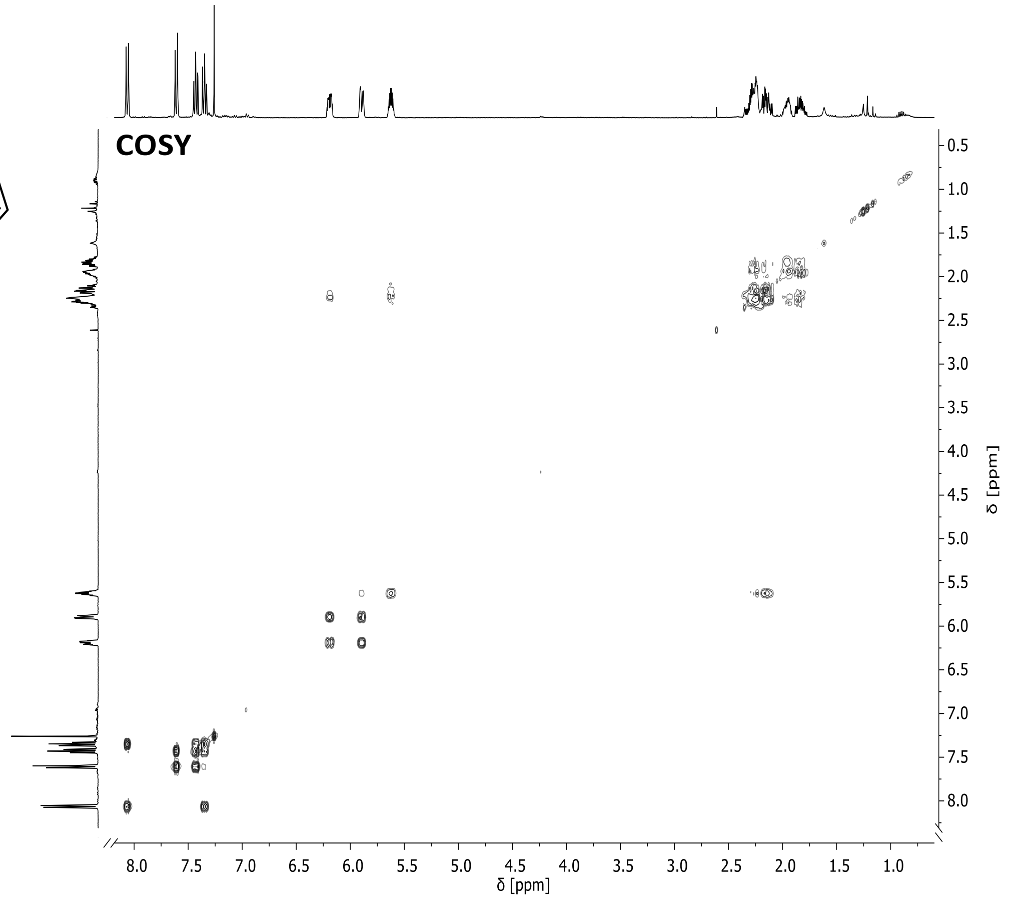

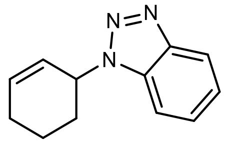

4aa (N1)

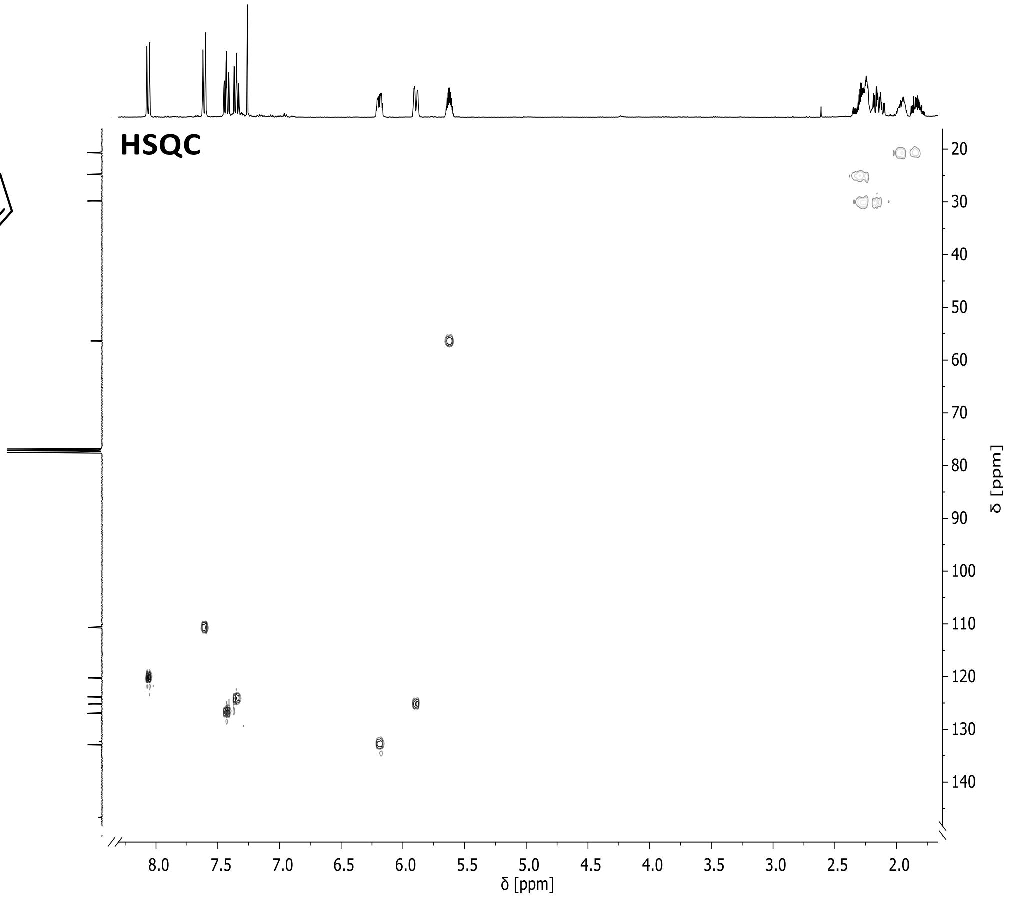

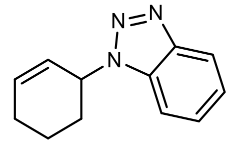

4aa (N^1)

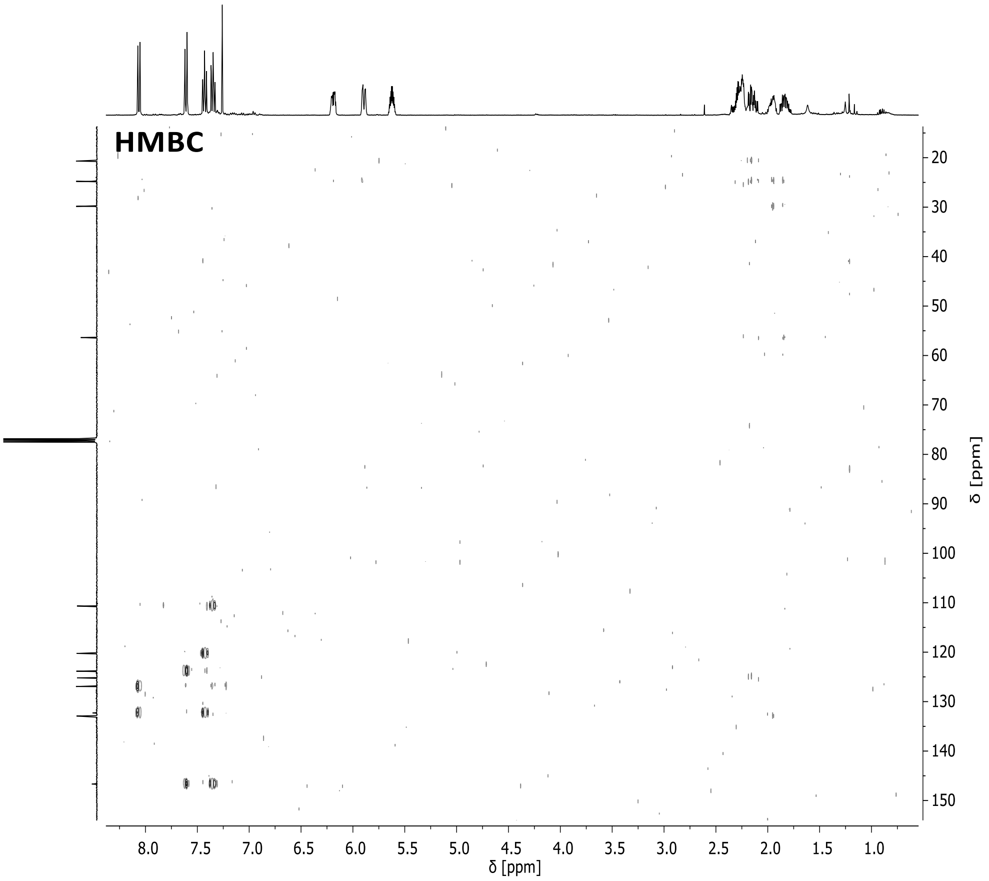

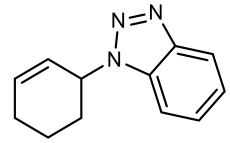

4aa (N^1)

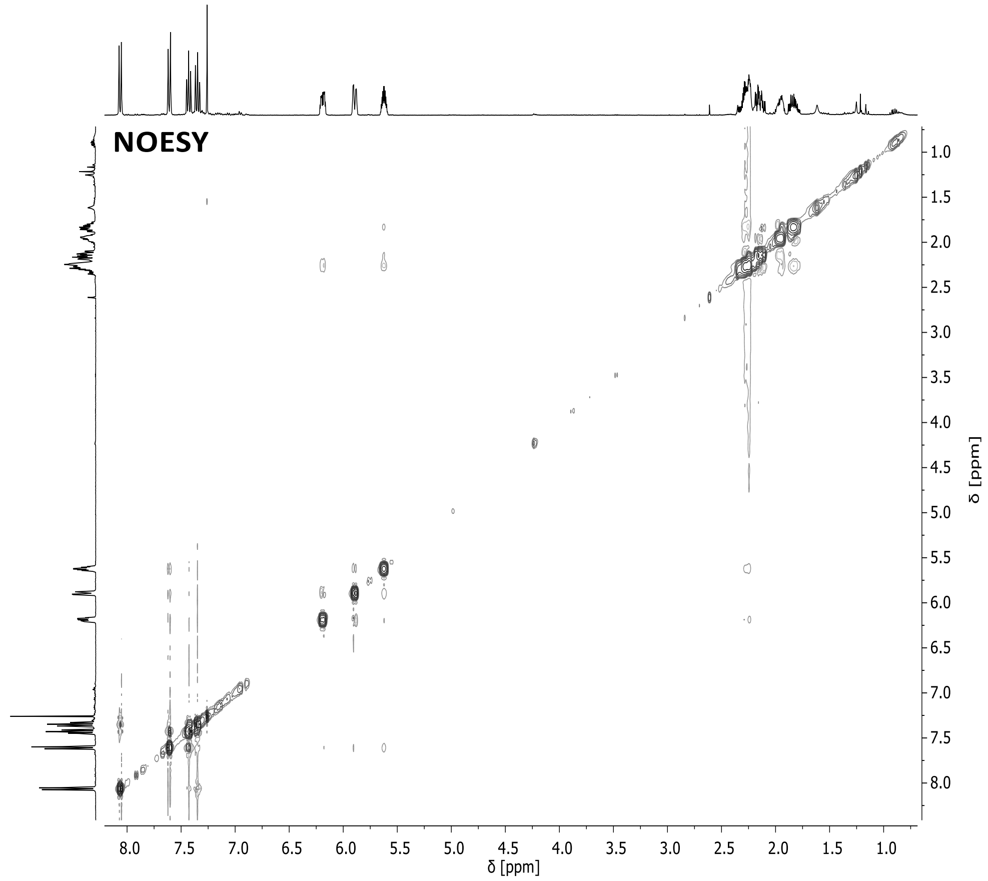

^{1}H NMR (400 MHz, $CDCl_3$) of **4ab** (N^2)

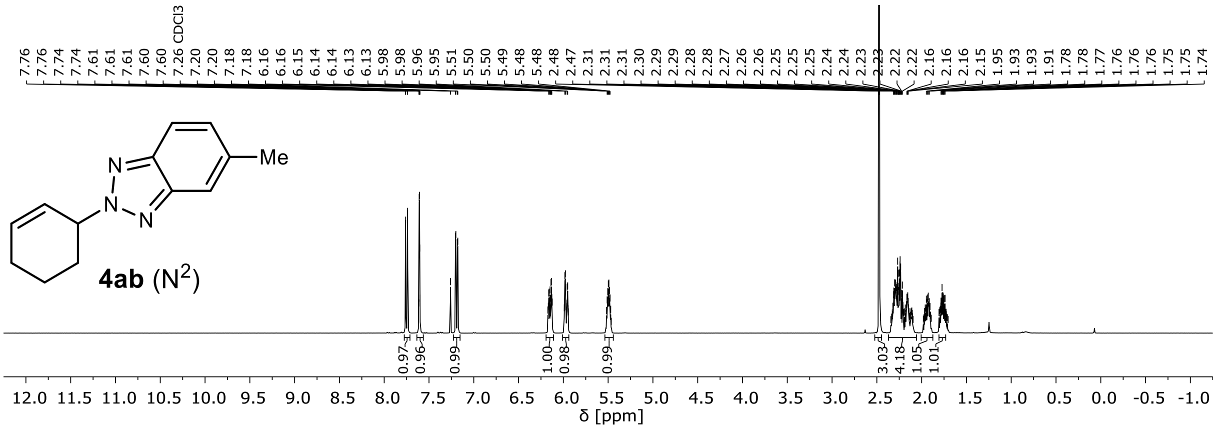

^{13}C NMR (101 MHz, $CDCl_3$) of **4ab** (N^2)

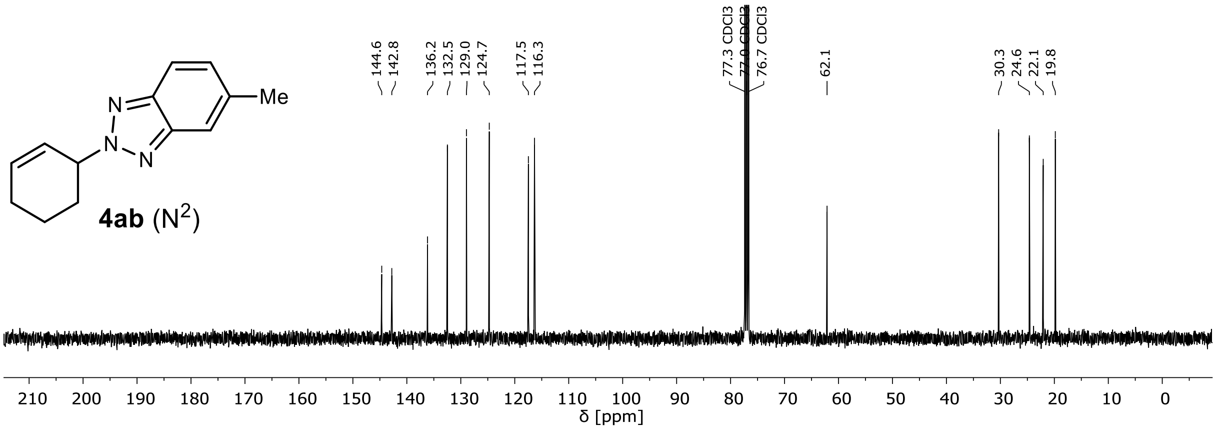

IR (ATR, neat) of **4ab** (N^2)

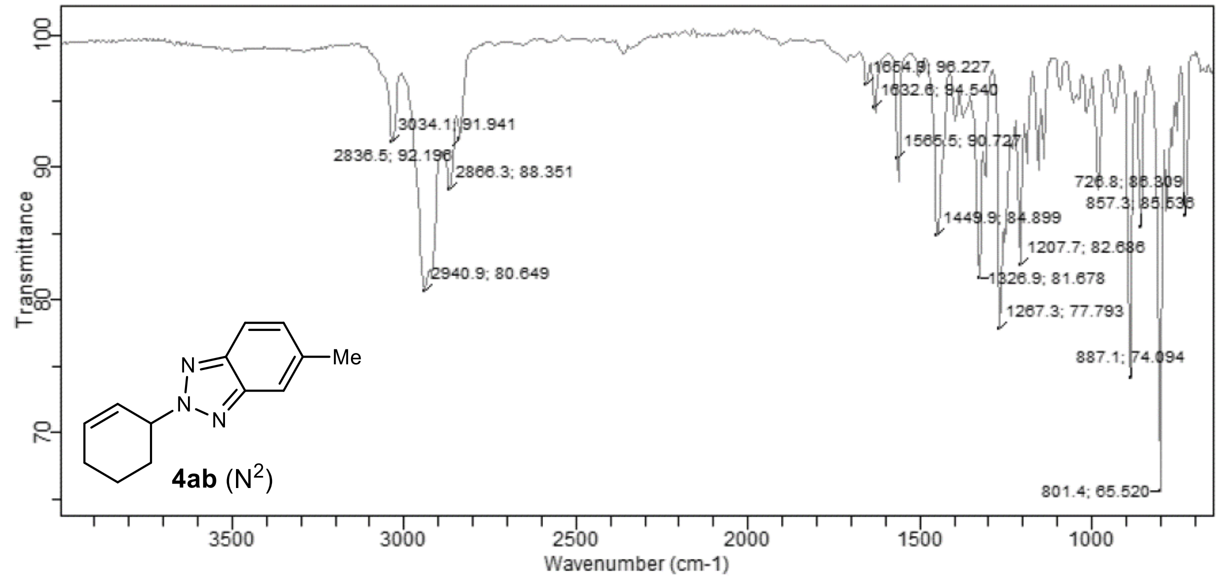

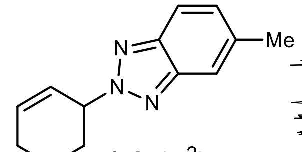

4ab (N^2)

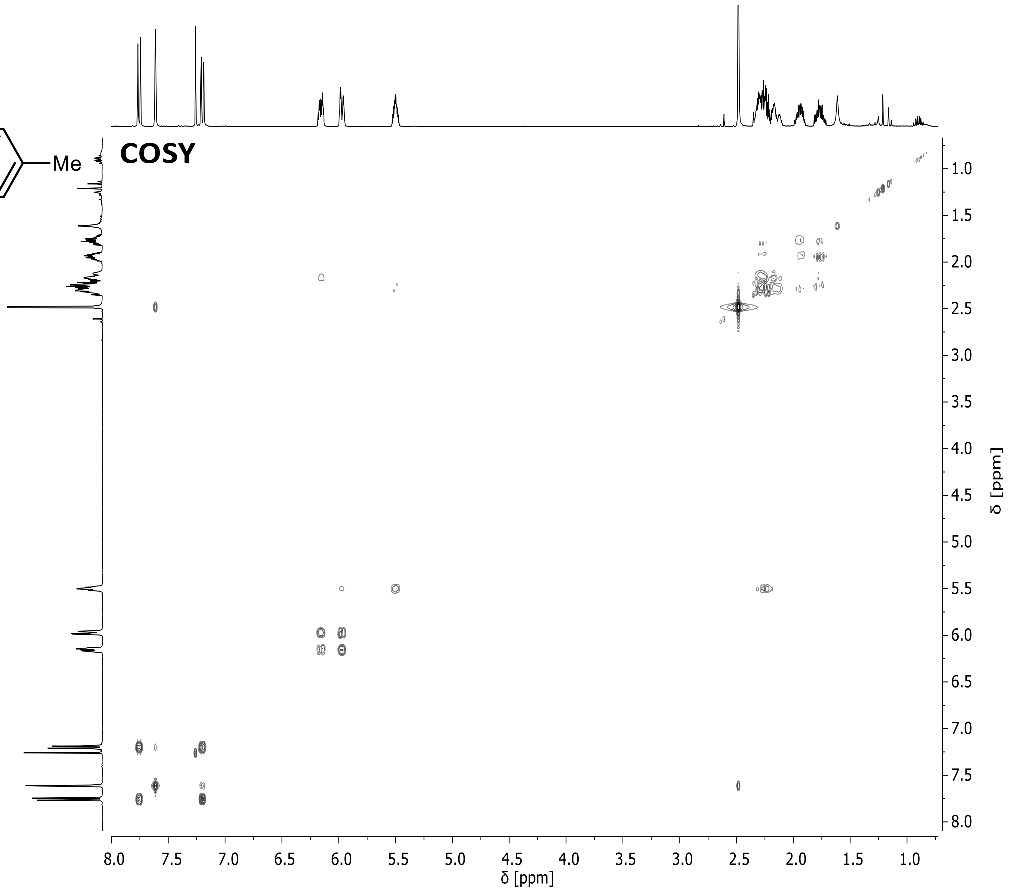

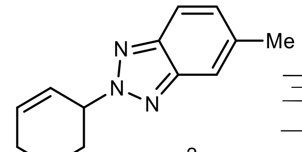

4ab (N^2)

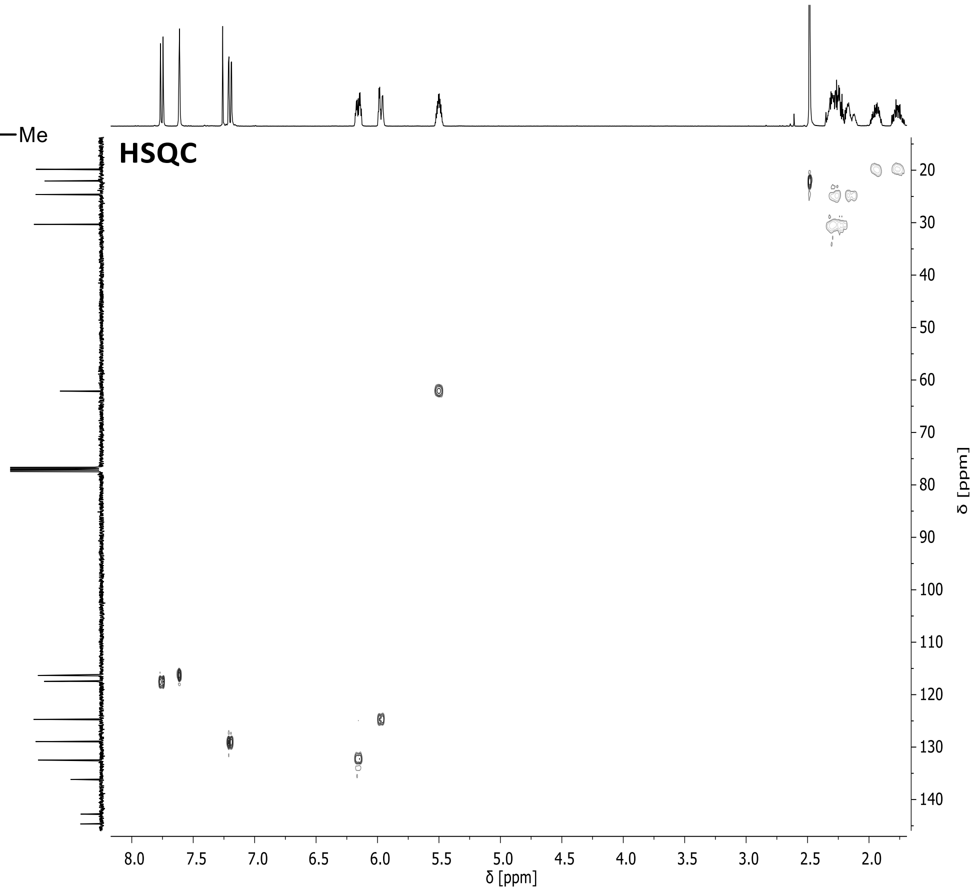

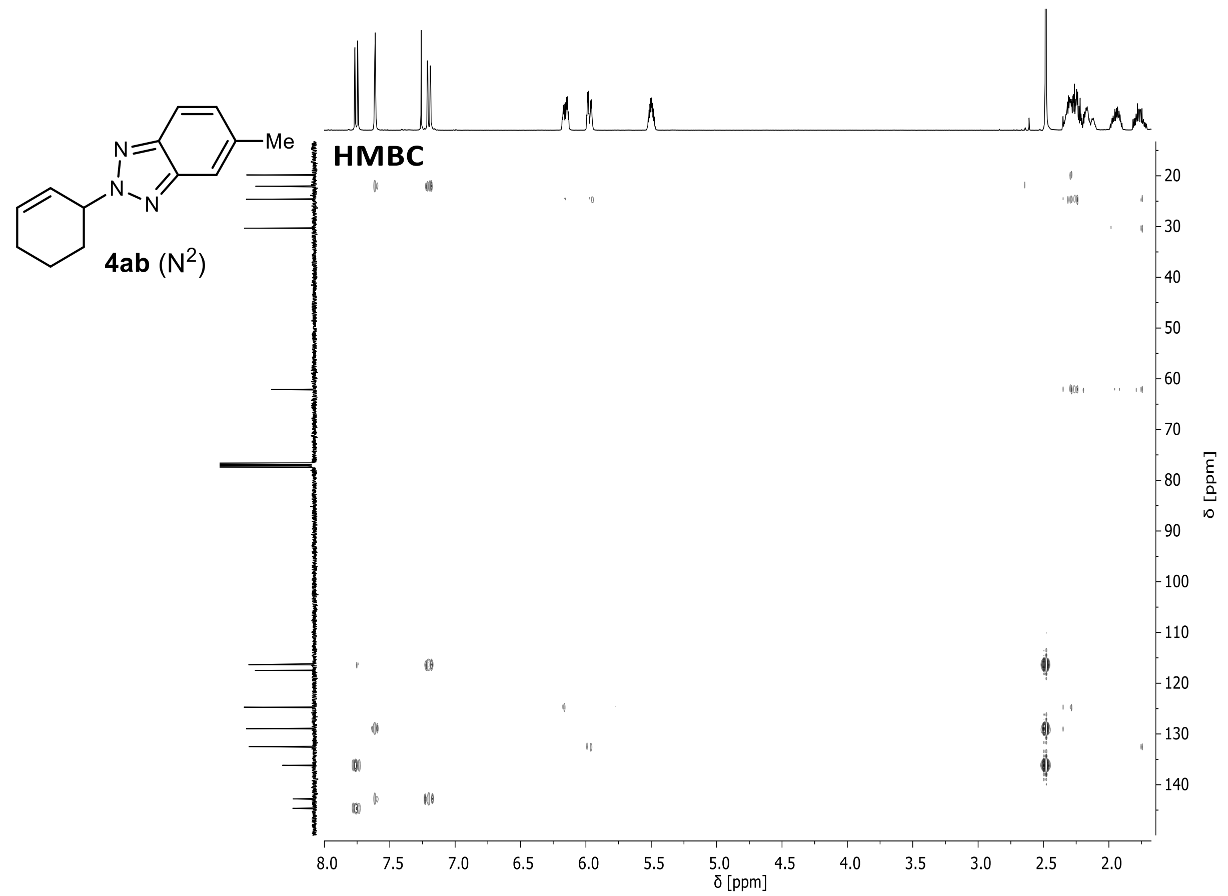
Me
N
N
N
4ab (N2)
HMBC
δ [ppm]
δ [ppm]

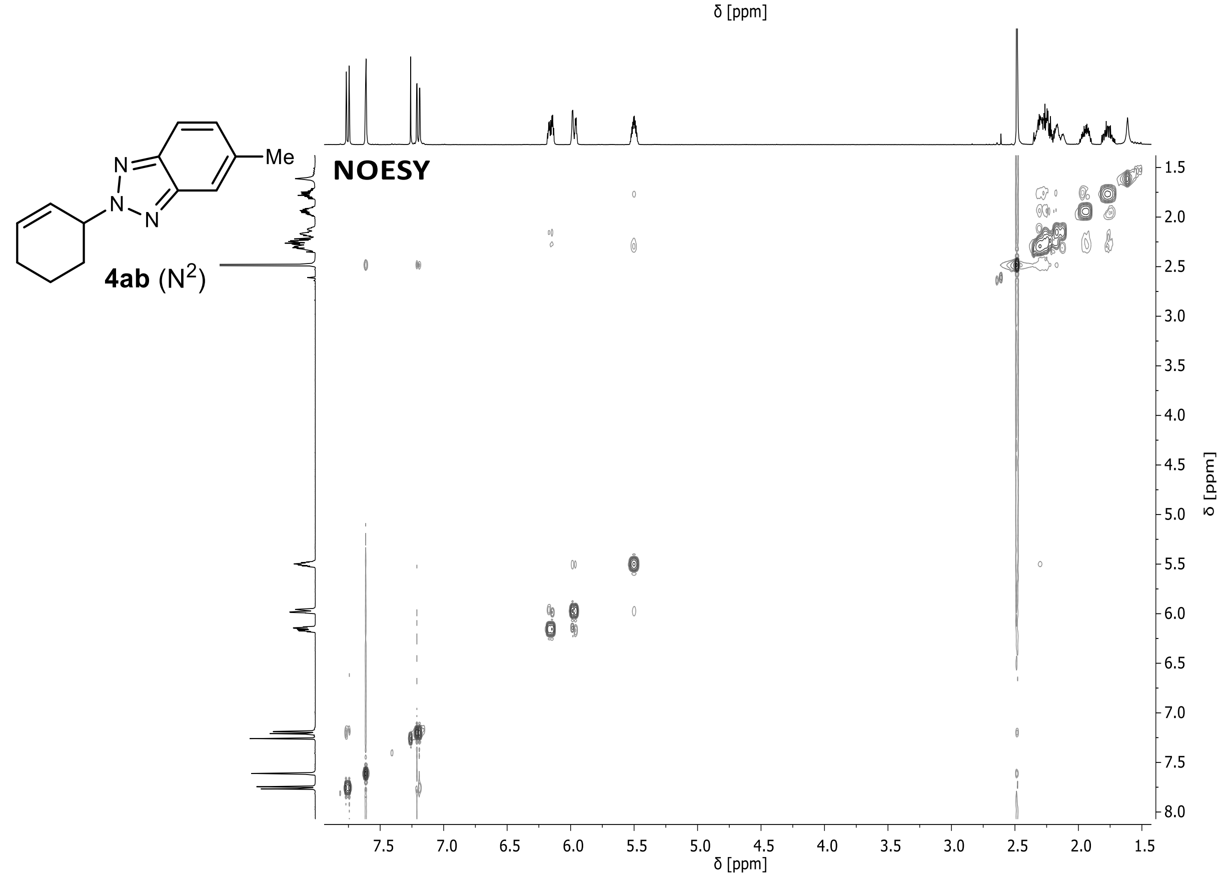
Me
N
N
N
4ab (N2)
NOESY
δ [ppm]
δ [ppm]

^{1}H NMR (400 MHz, $CDCl_3$) of **4ab** ($N^3 + N^1$)

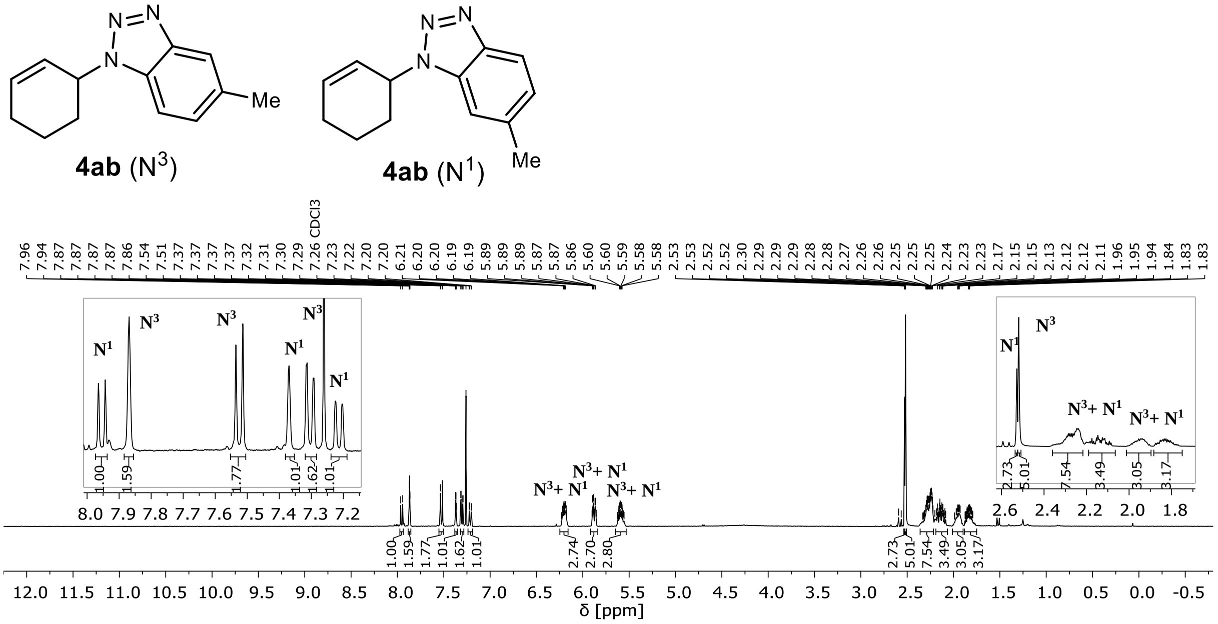

^{13}C NMR (101 MHz, $CDCl_3$) of **4ab** ($N^1 + N^3$)

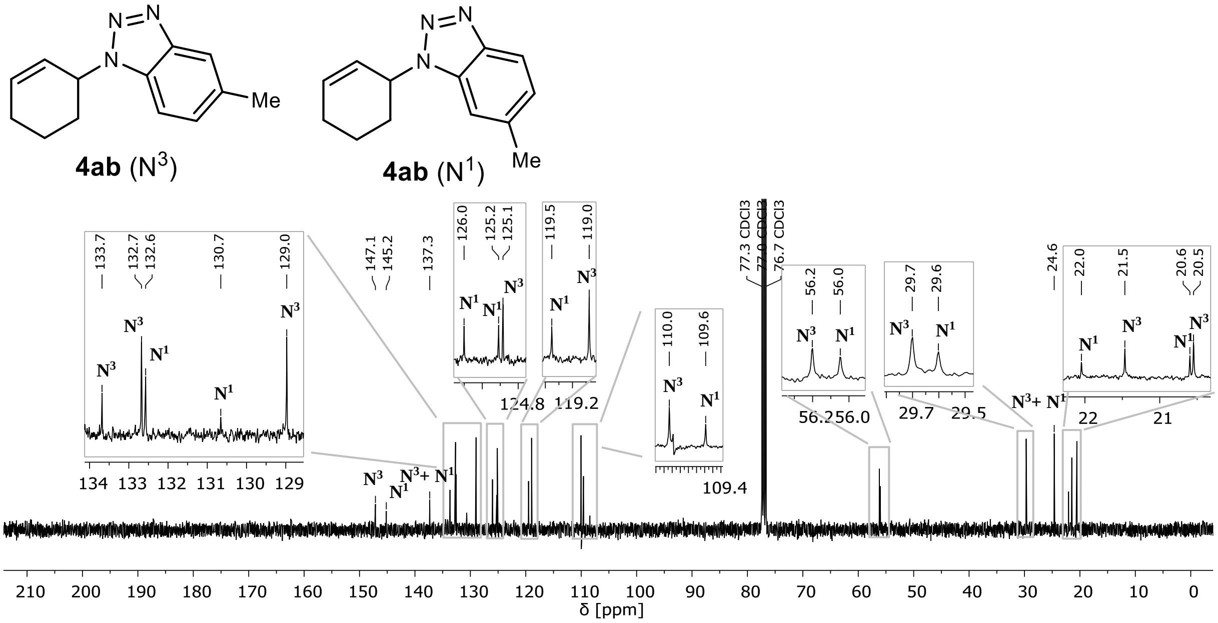

IR (ATR, neat) of **4ab** (N^1 + N^3)

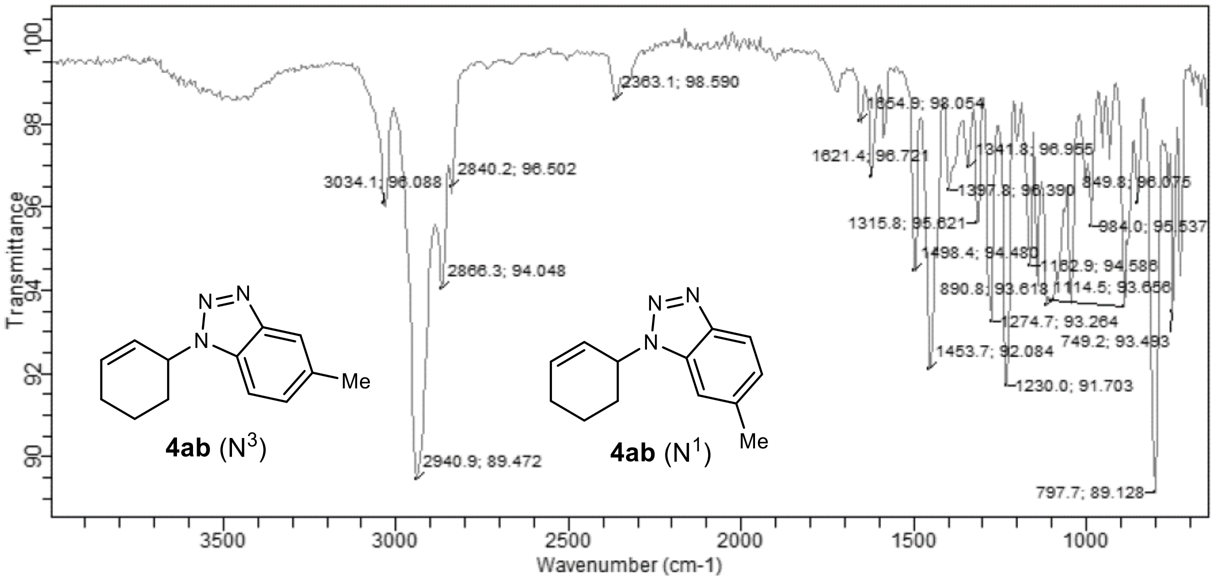

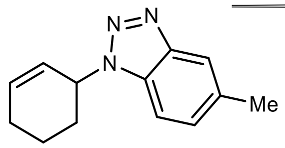

4ab (N^3)

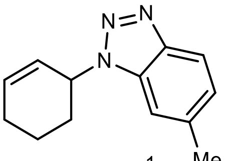

4ab (N^1)

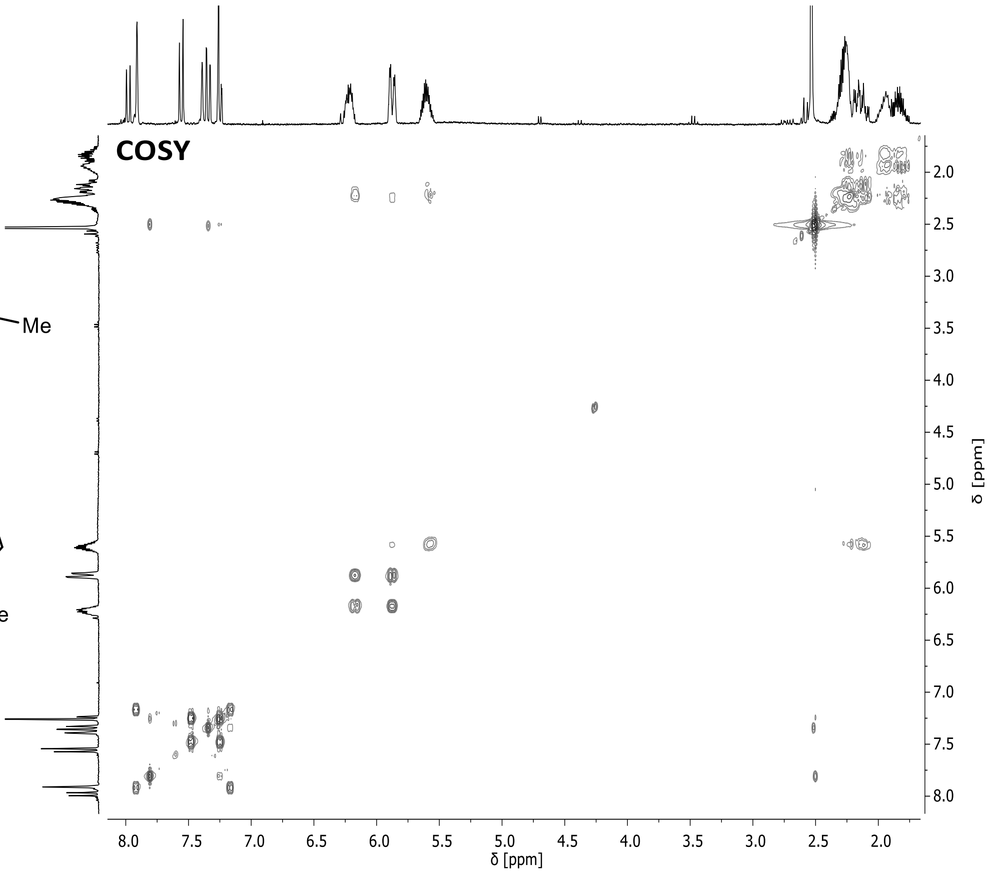

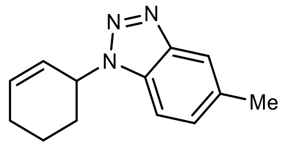

4ab (N^3)

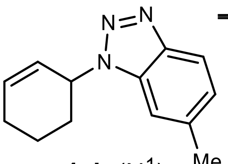

4ab (N^1)

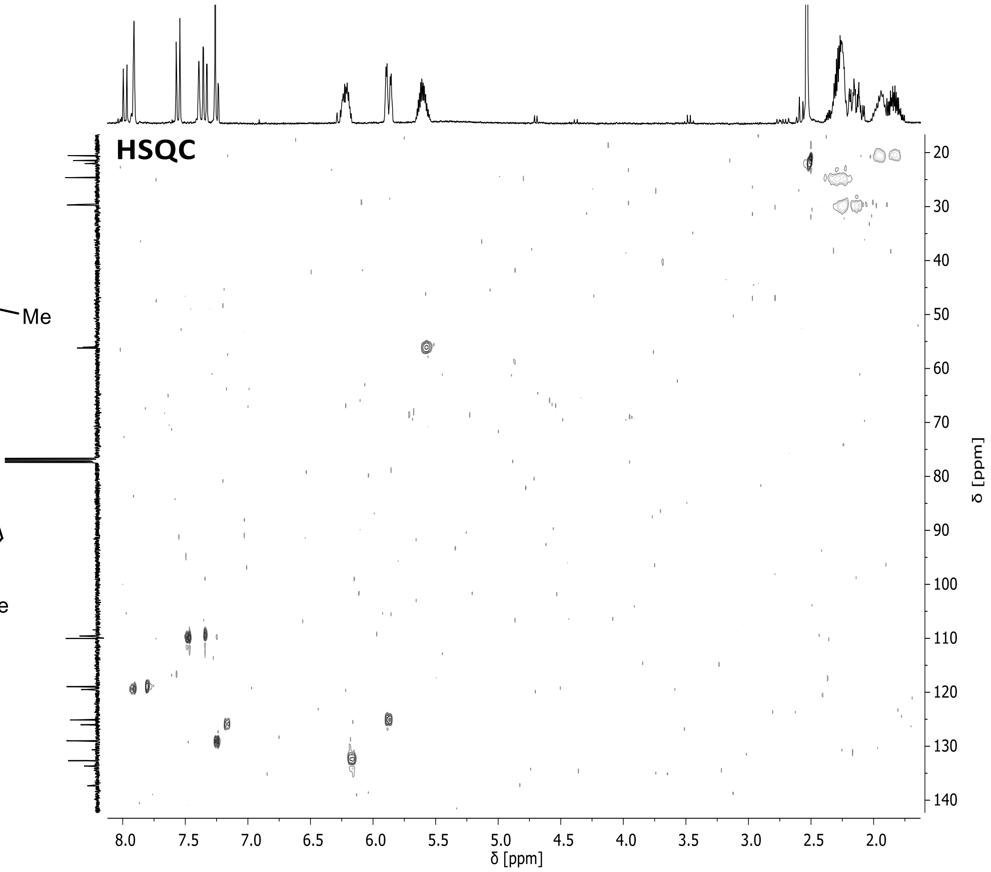

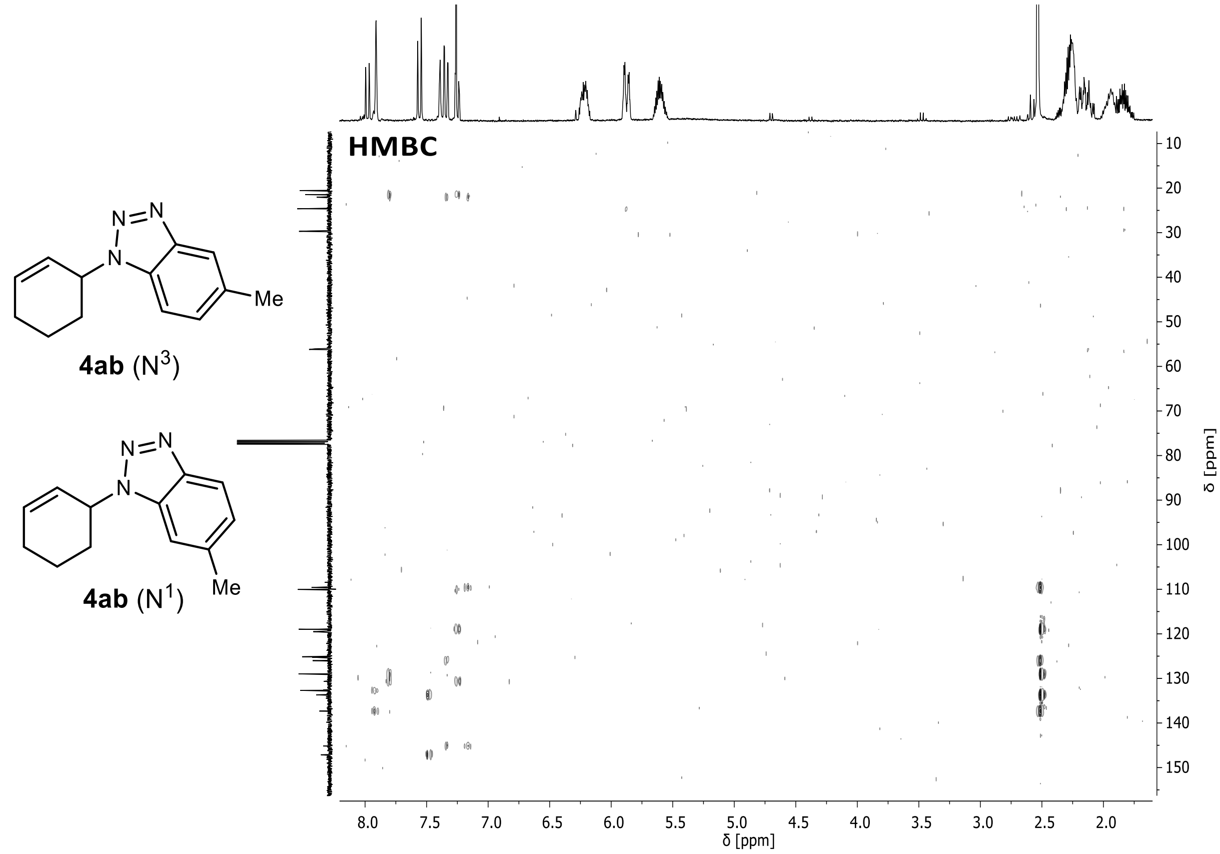
HMBC
4ab (N3)
4ab (N1)
Me
Me
δ [ppm]
δ [ppm]

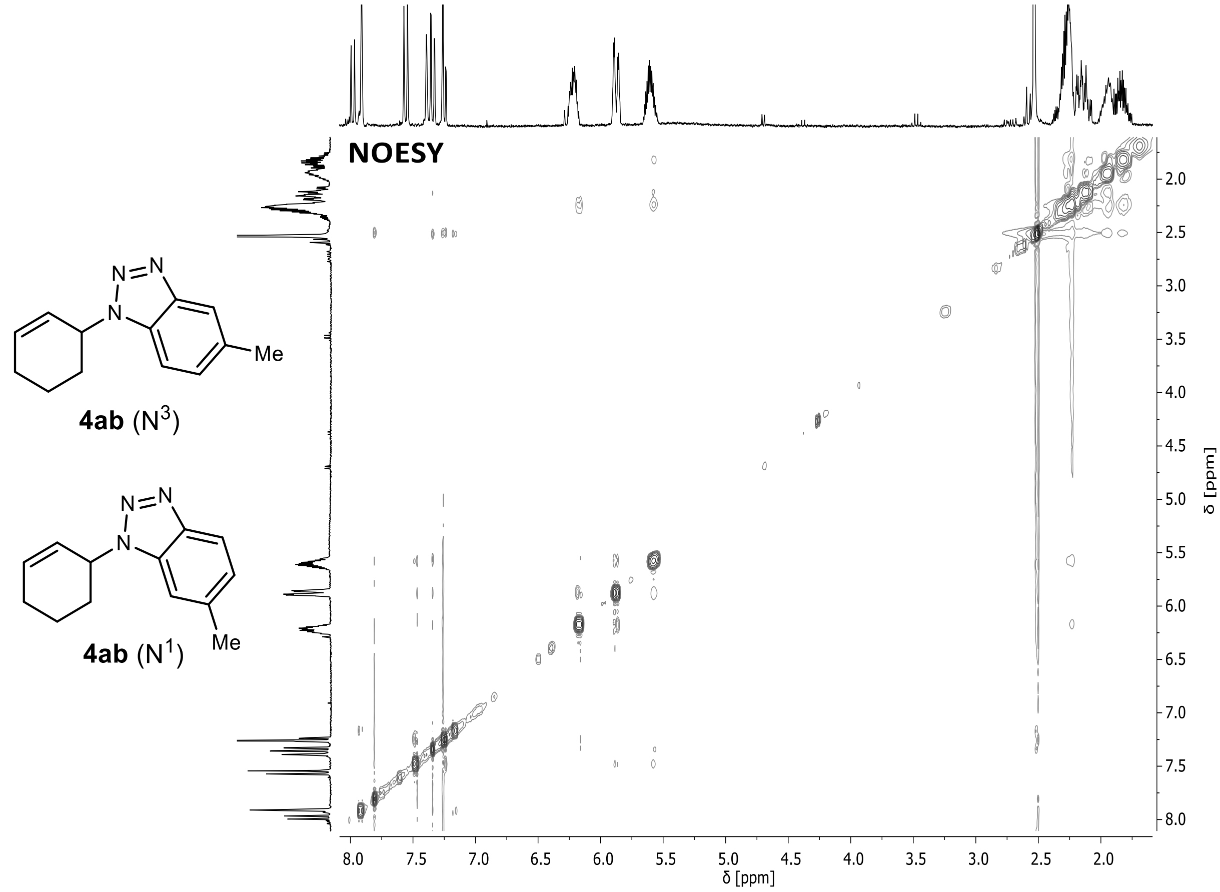
NOESY
4ab (N3)
4ab (N1)
Me
Me
δ [ppm]
δ [ppm]

^{1}H NMR (400 MHz, $CDCl_3$) of **4ac** (N^2)

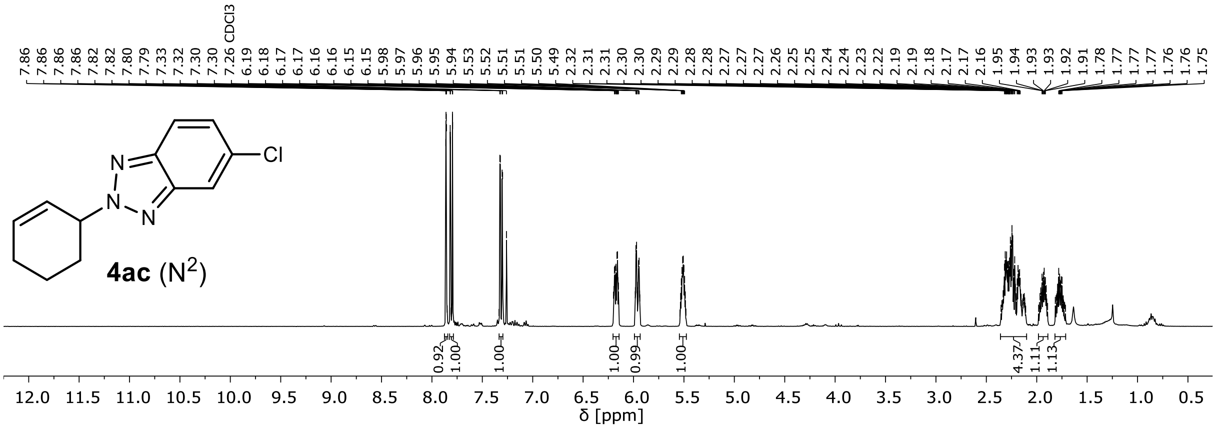

^{13}C NMR (101 MHz, $CDCl_3$) of **4ac** (N^2)

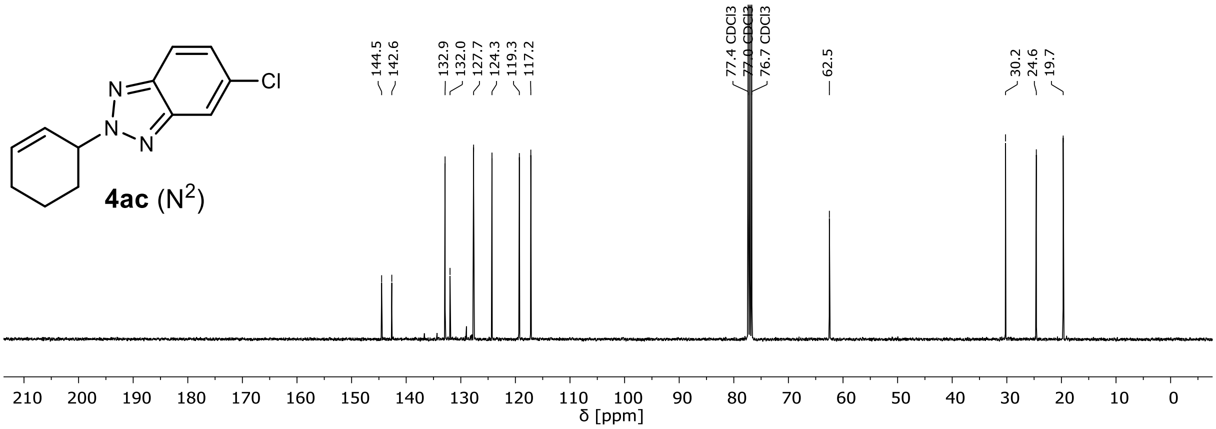

IR (ATR, neat) of **4ac** (N^2)

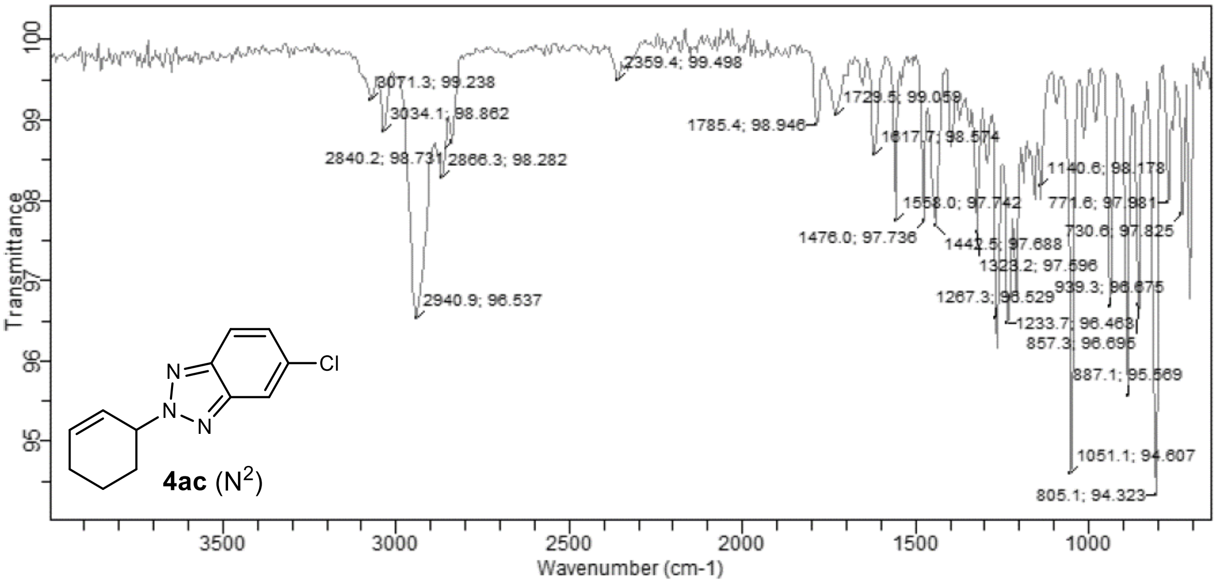

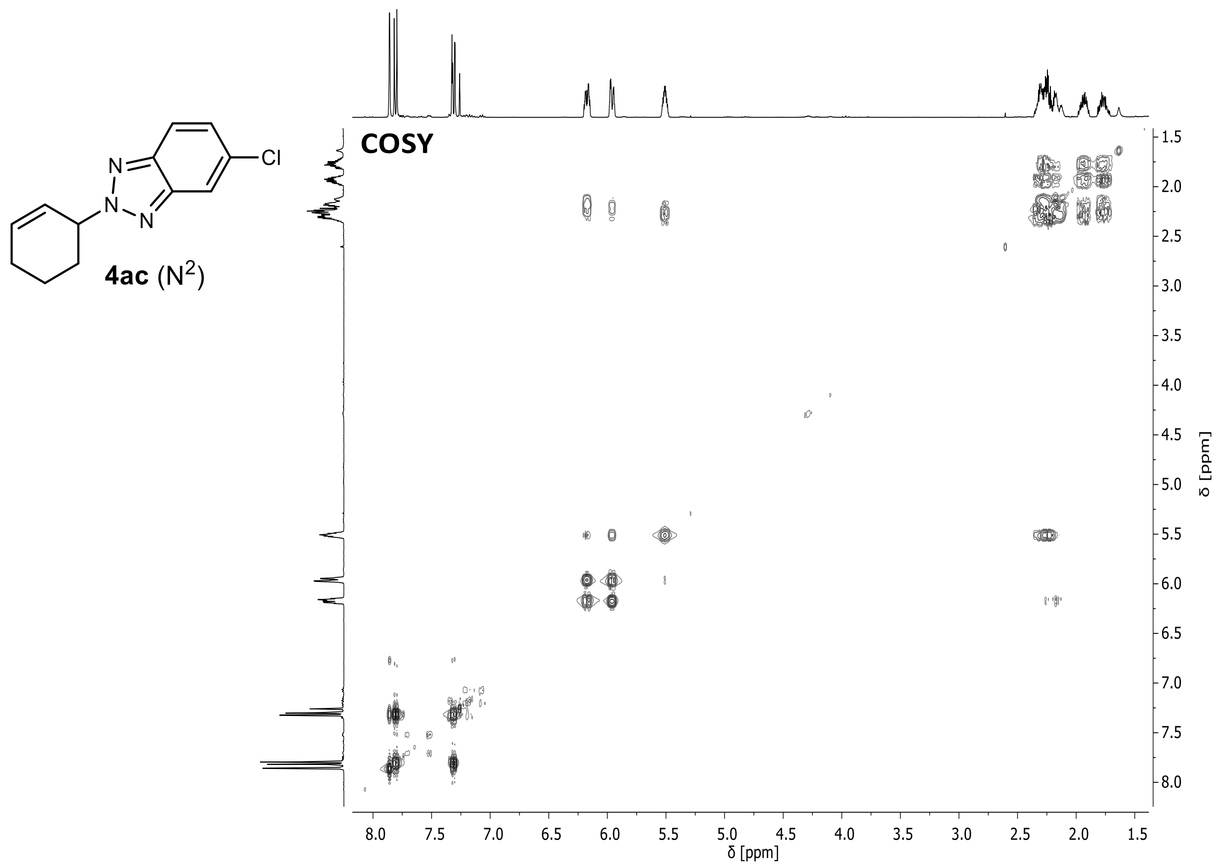
N
N
N
Cl
4ac (N2)
COSY
δ [ppm]
δ [ppm]

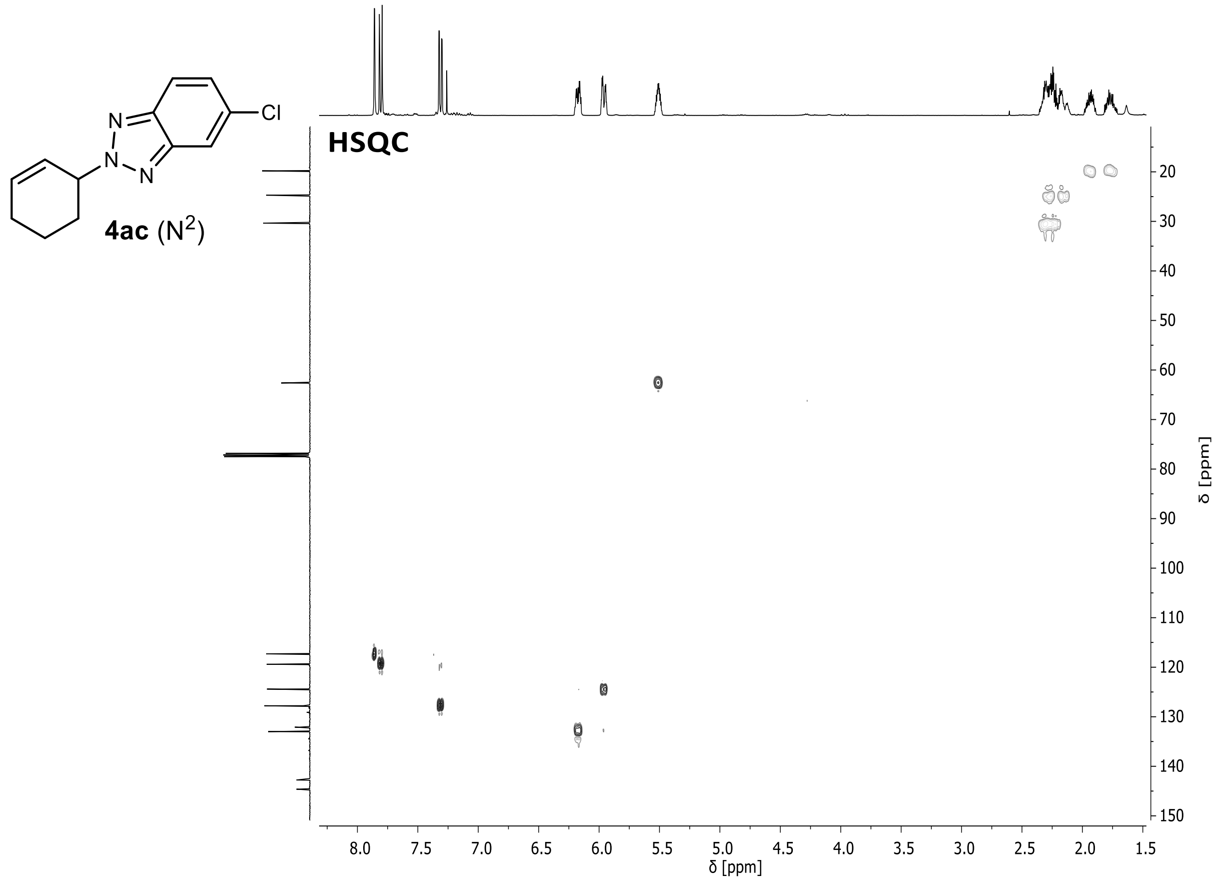
N
N
N
Cl
4ac (N2)
HSQC
δ [ppm]
δ [ppm]

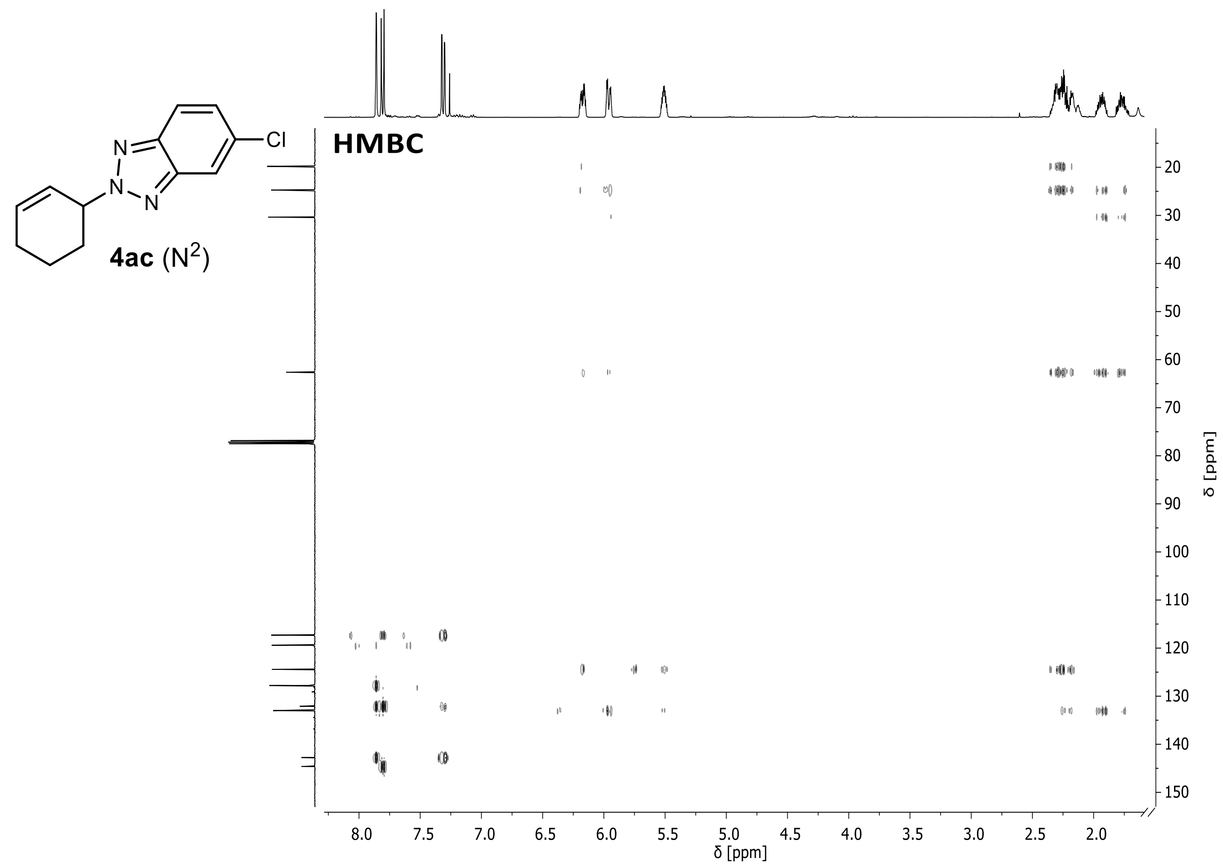

HMBC
4ac (N2)
Cl
N
δ [ppm]

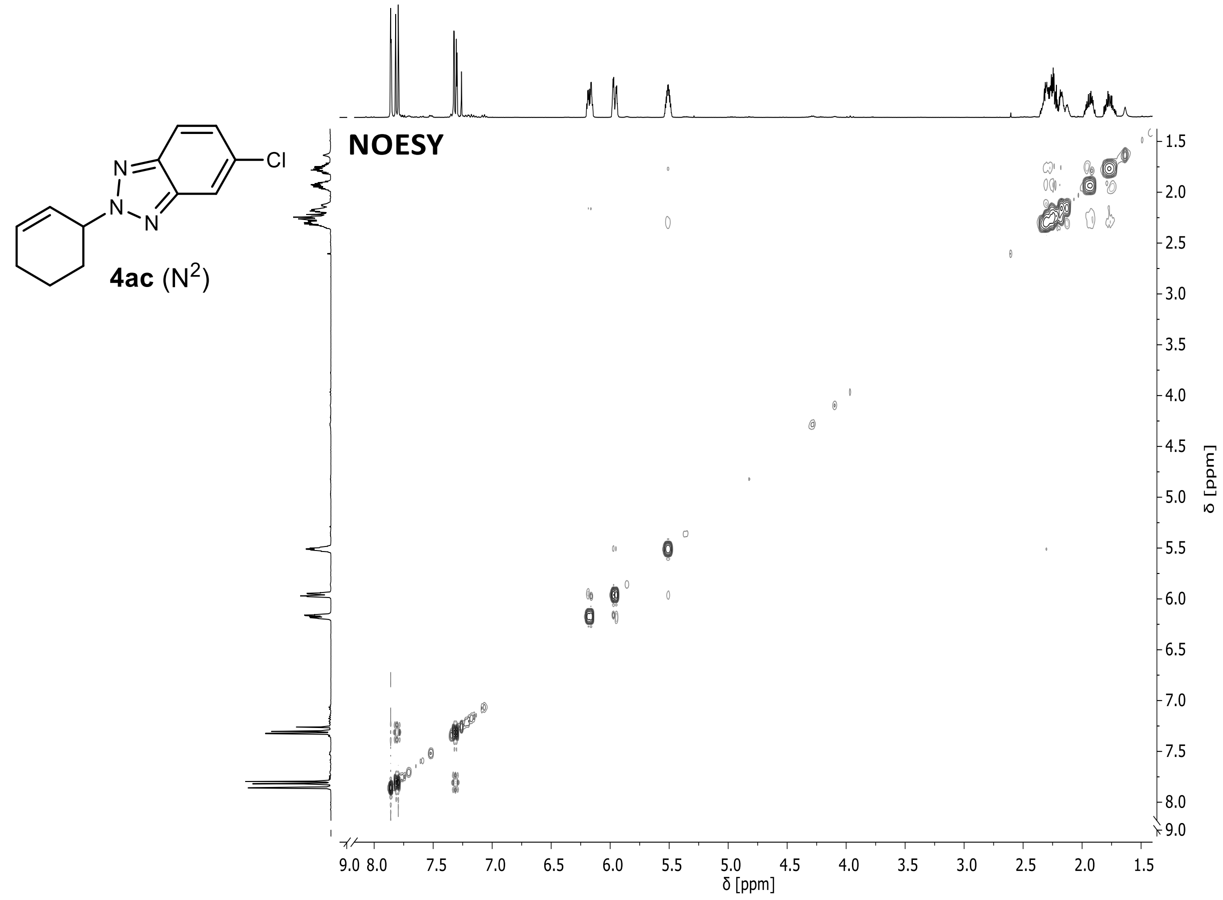

NOESY
4ac (N2)
Cl
N
δ [ppm]

^{1}H NMR (400 MHz, $CDCl_3$) of **4ac** ($N^3 + N^1$)

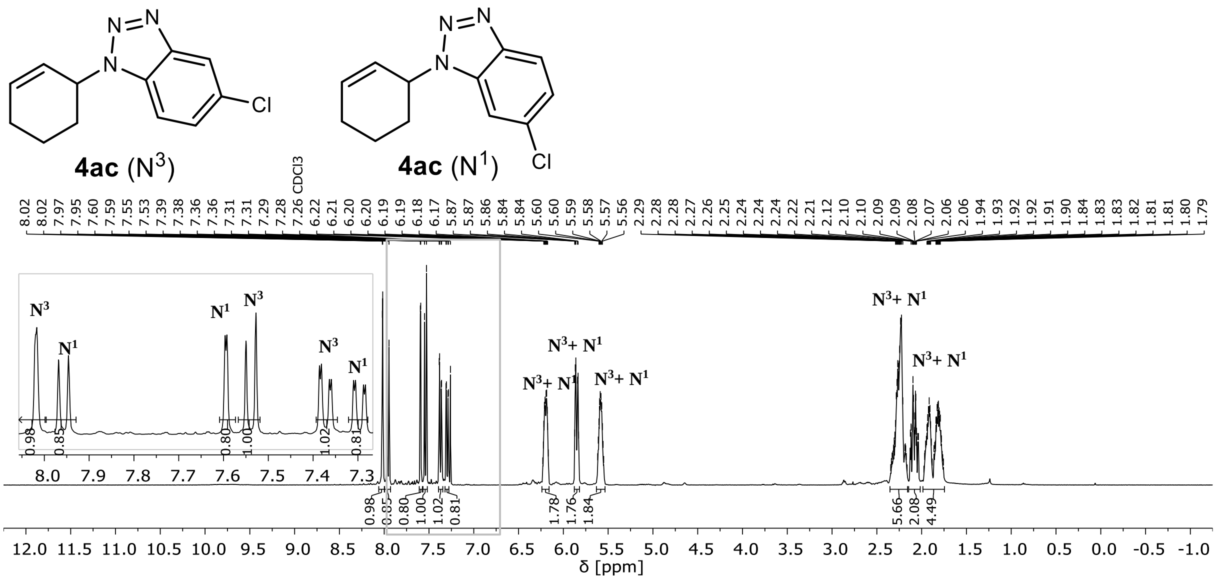

^{13}C NMR (101 MHz, $CDCl_3$) of **4ab** ($N^1 + N^3$)

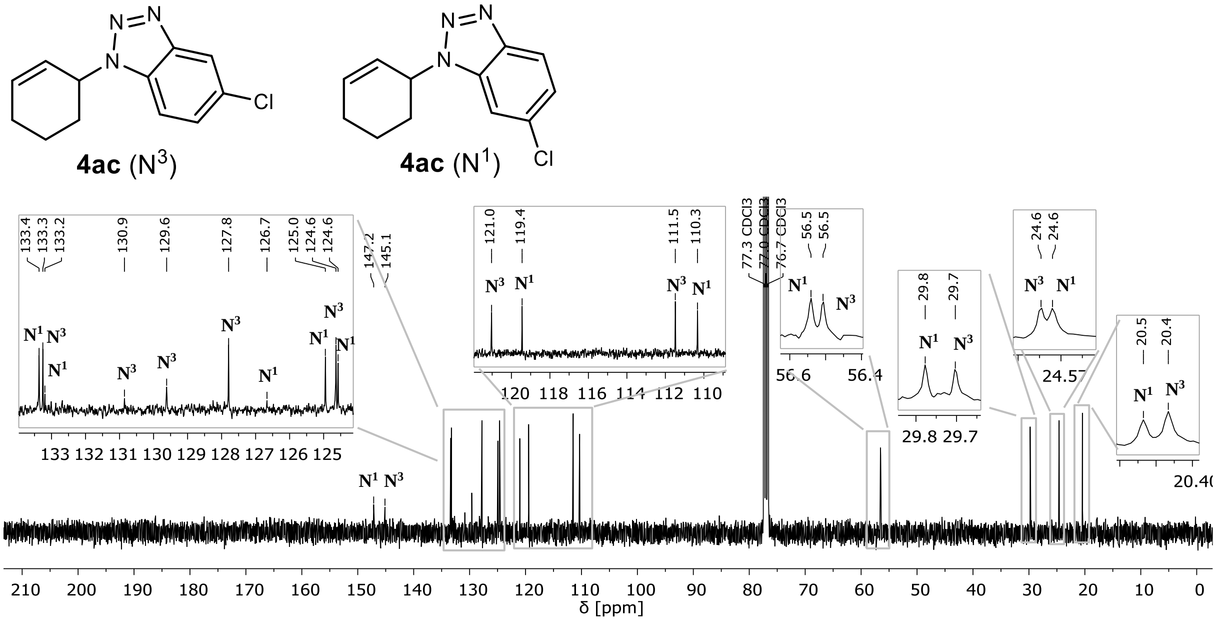

IR (ATR, neat) of **4ab** (N[1] + N[3])

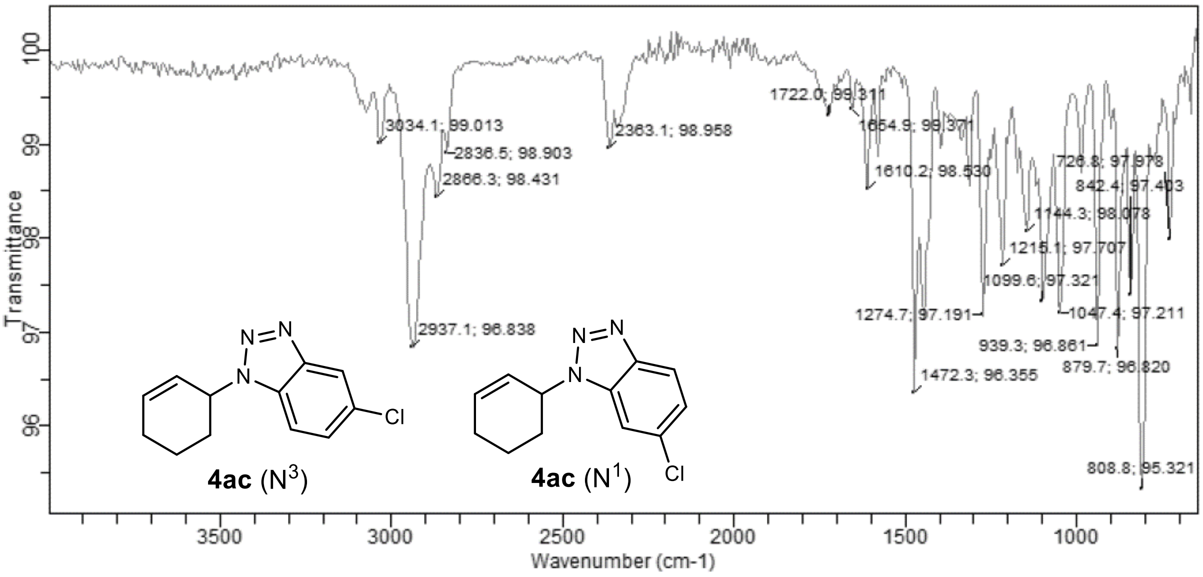

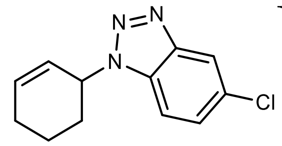

4ac (N^3)

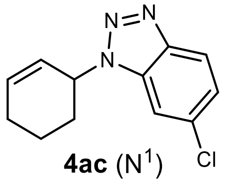

4ac (N^1)

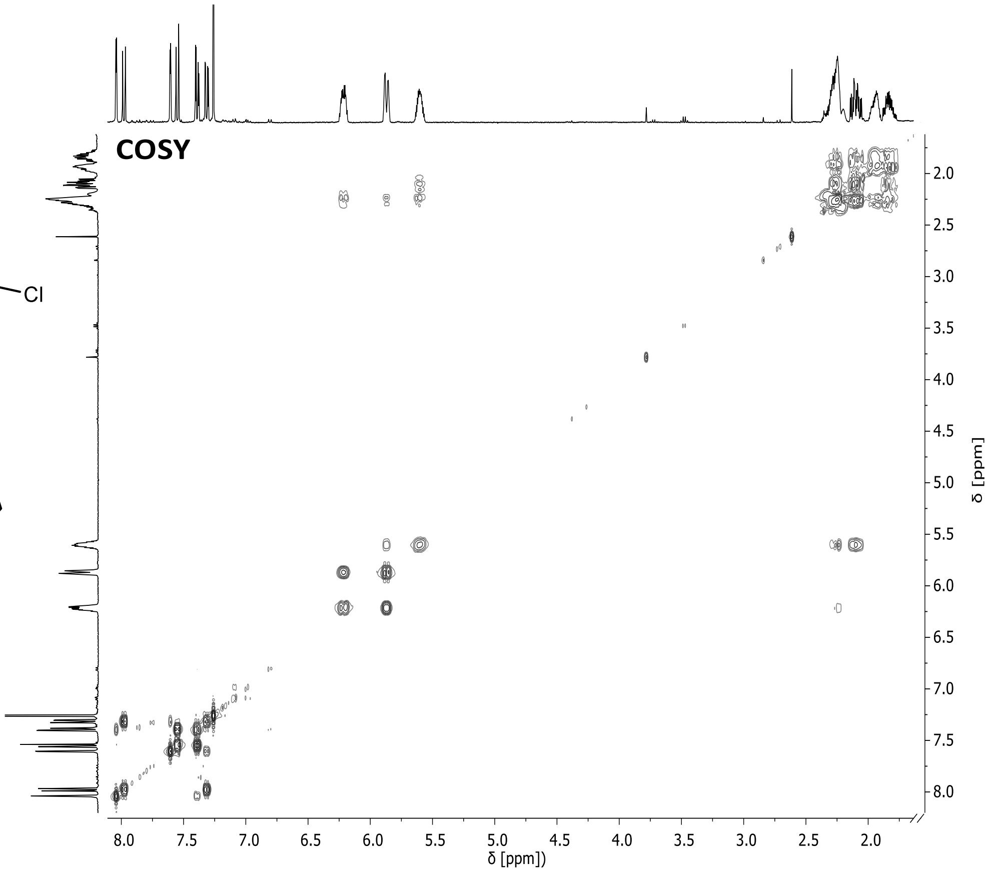

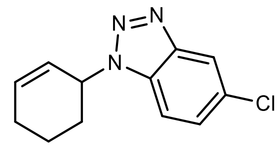

4ac (N^3)

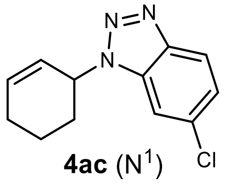

4ac (N^1)

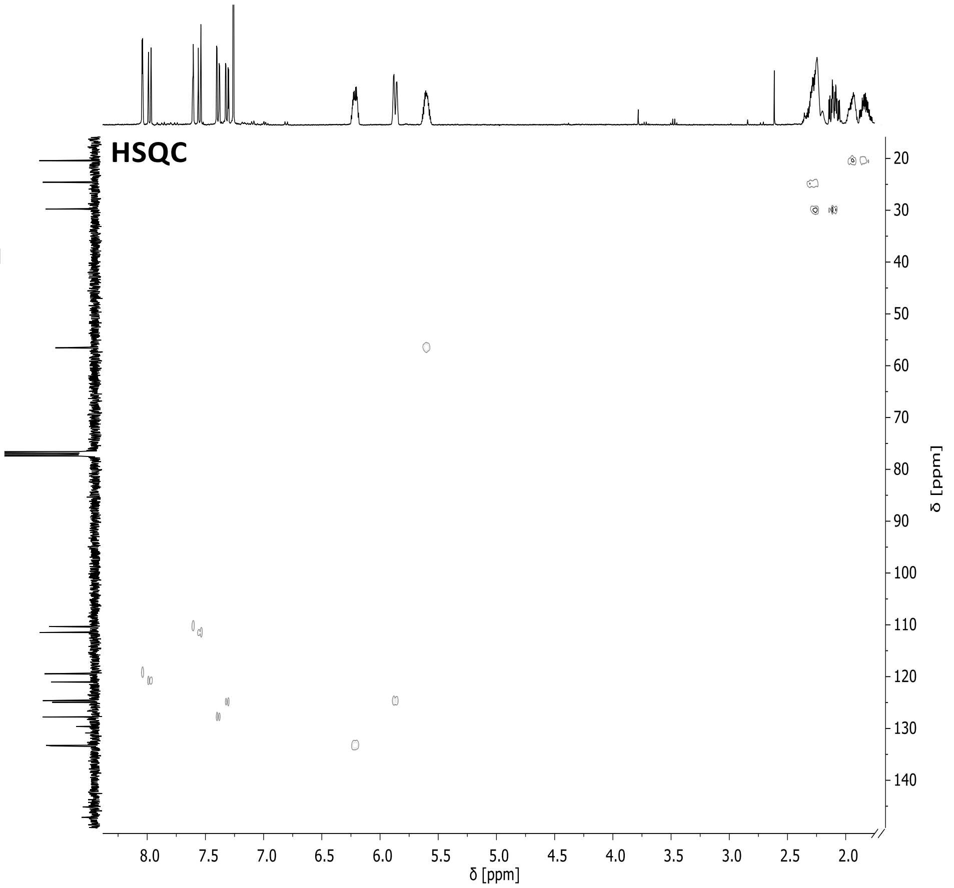

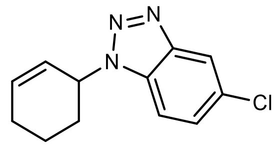

4ac (N³)

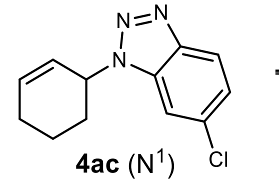

4ac (N¹)

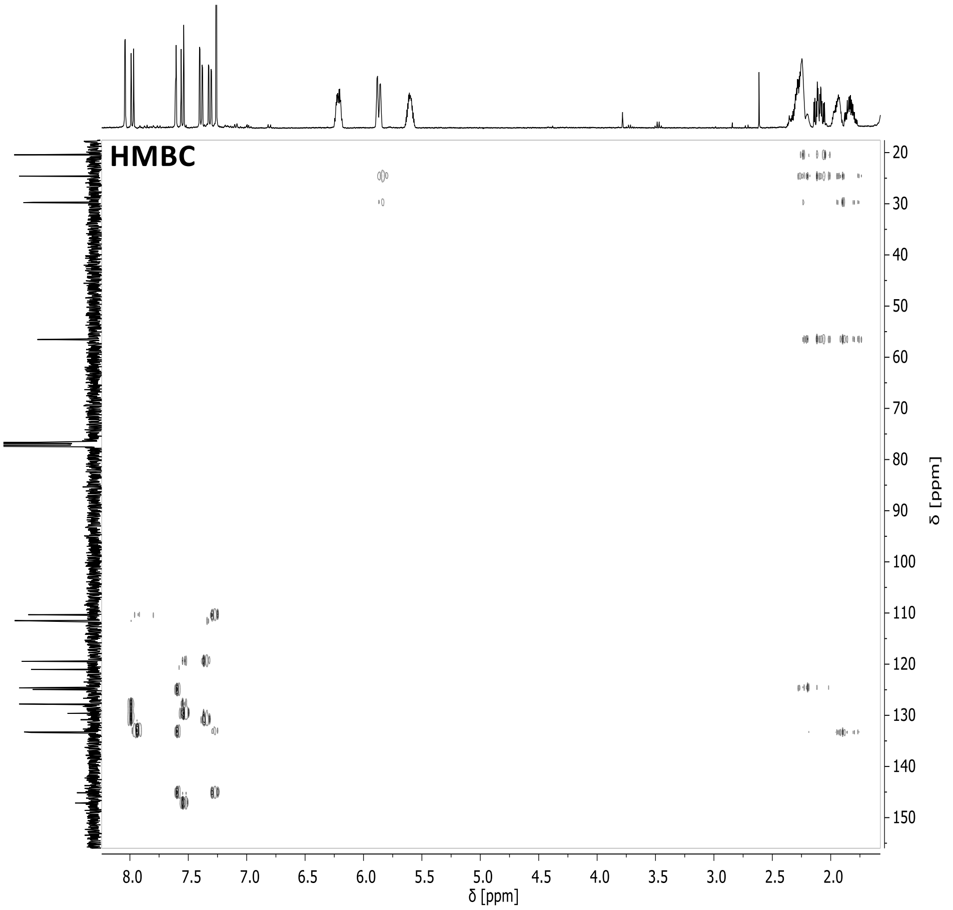

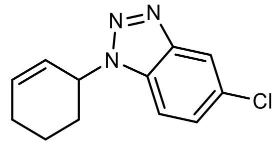

4ac (N³)

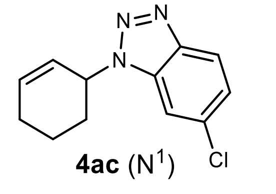

4ac (N¹)

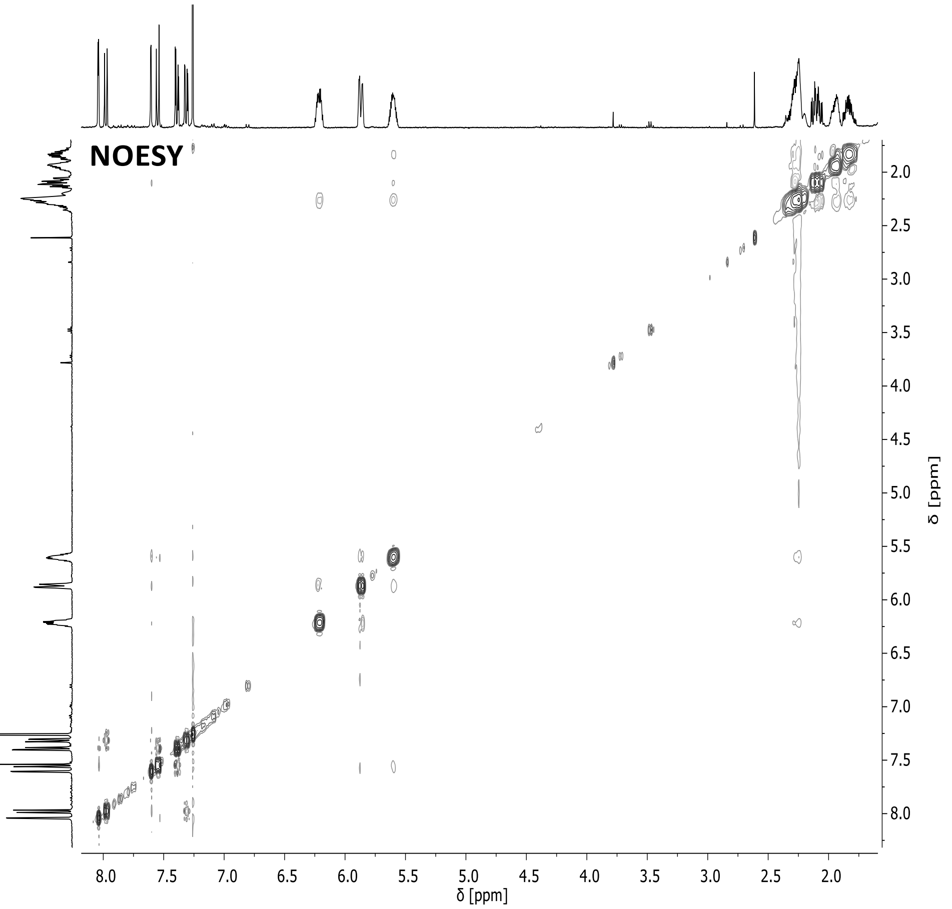

^{1}**H NMR** (400 MHz, $CDCl_3$) of **4ad** (N^2)

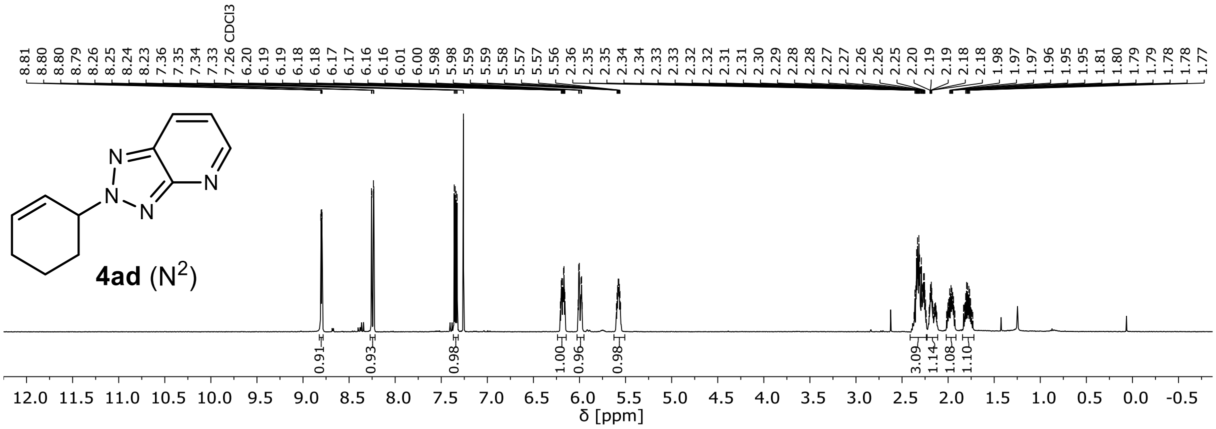

^{13}**C NMR** (101 MHz, $CDCl_3$) of **4ad** (N^2)

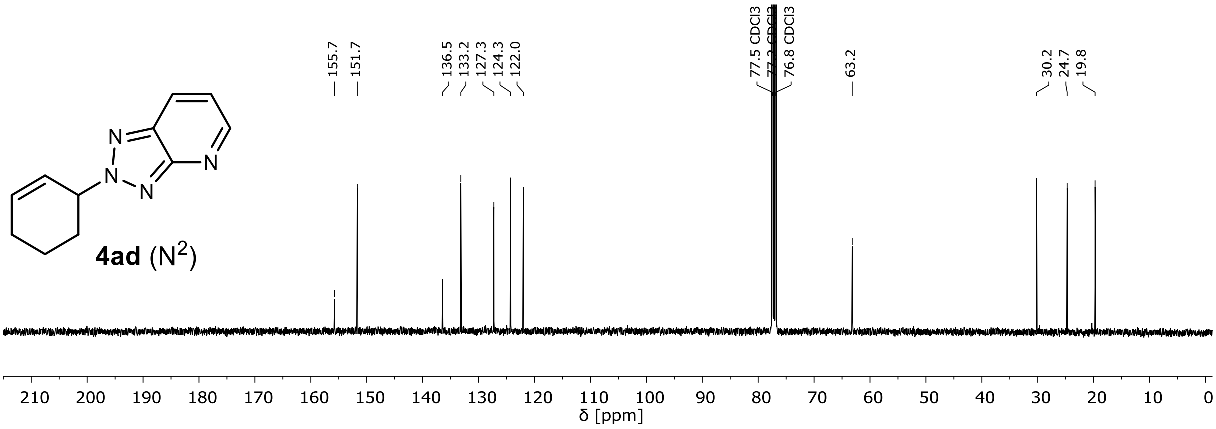

IR (ATR, neat) of **4ad** (N^2)

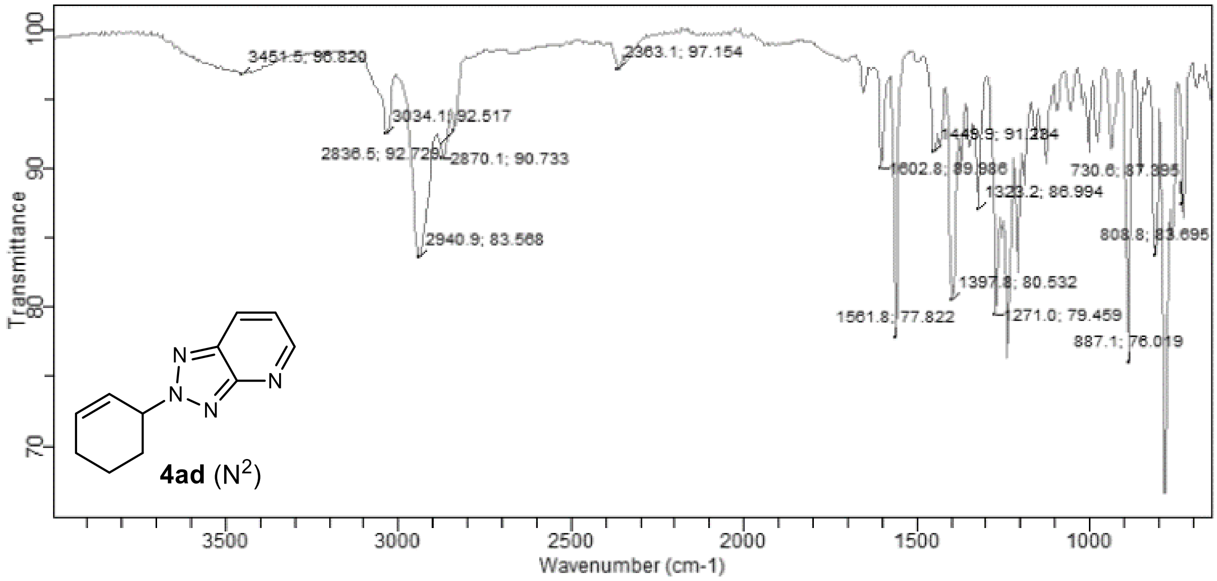

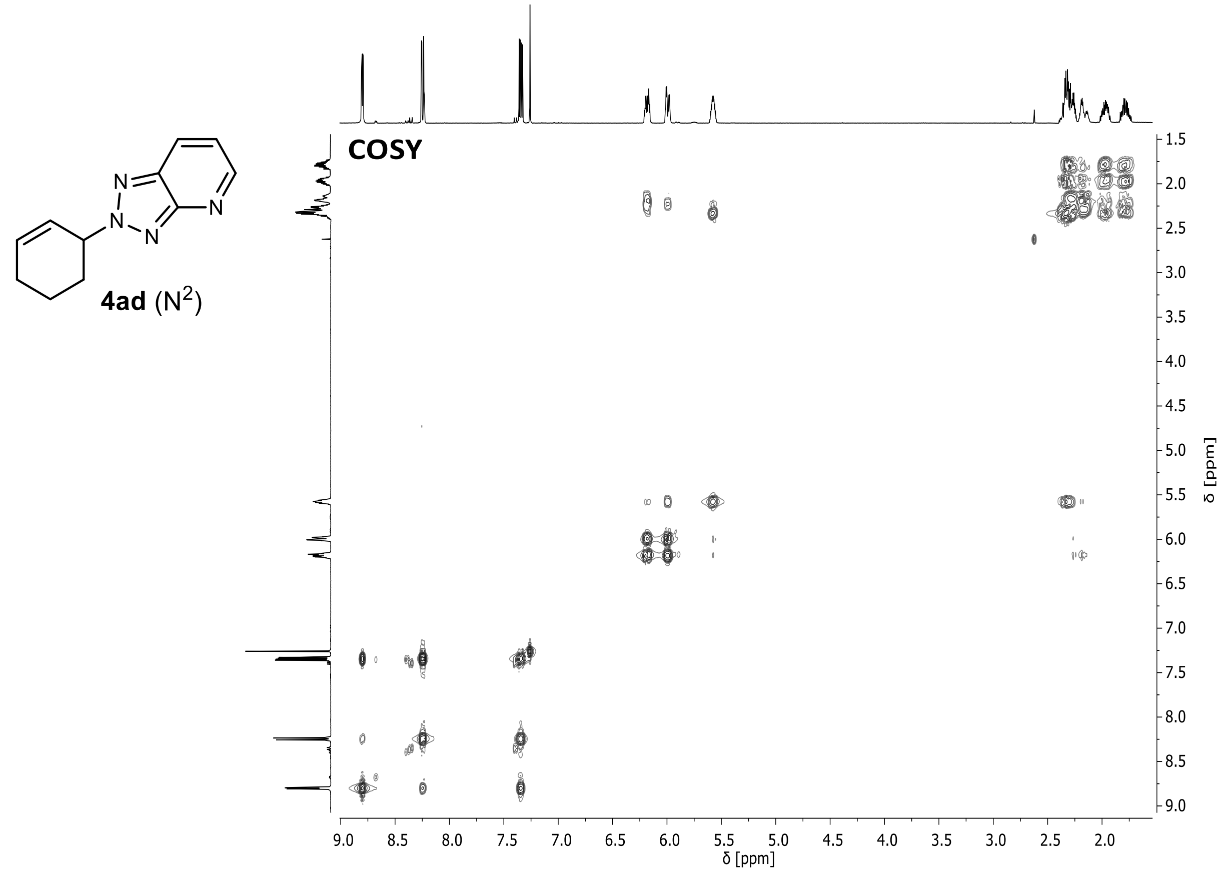
N
N
N
N
4ad (N2)
COSY
δ [ppm]
δ [ppm]

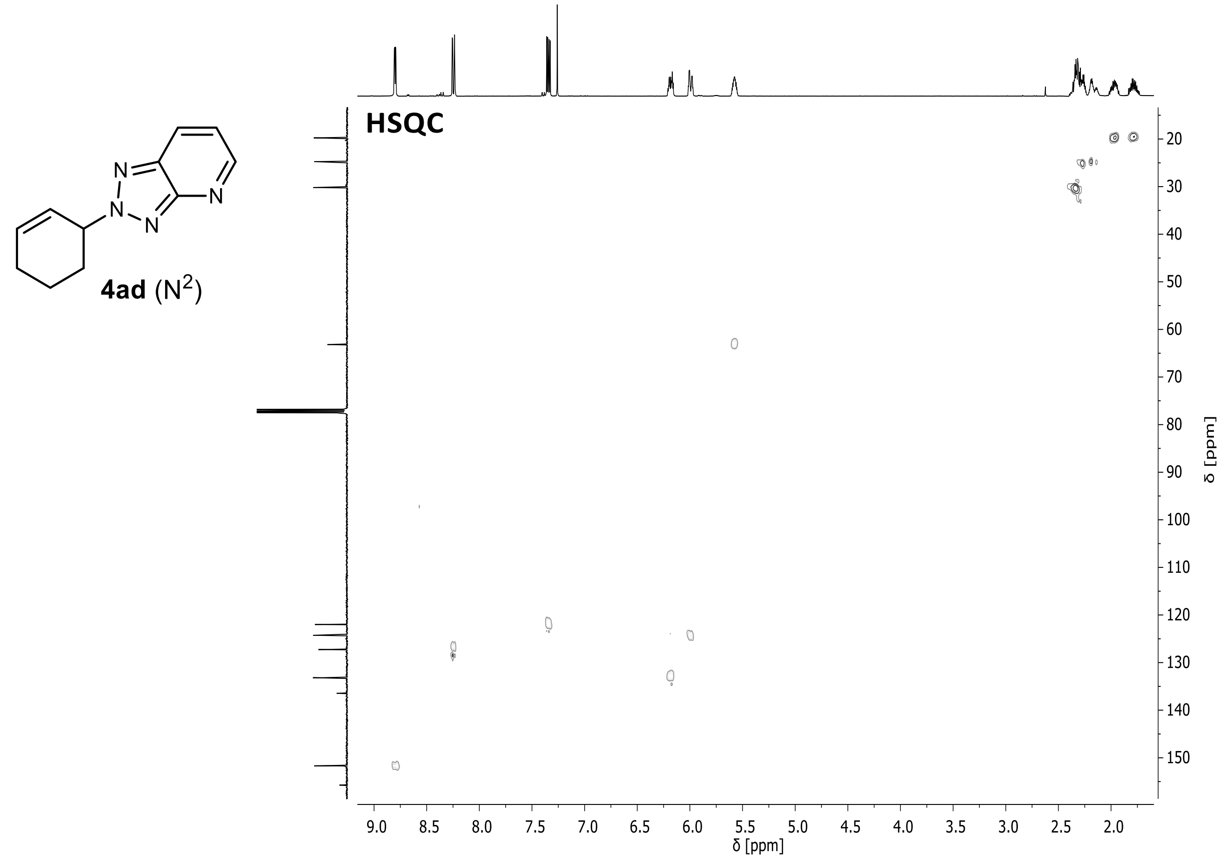
N
N
N
N
4ad (N2)
HSQC
δ [ppm]
δ [ppm]

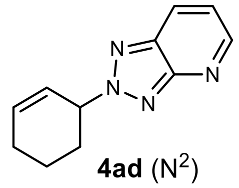

4ad (N^2)

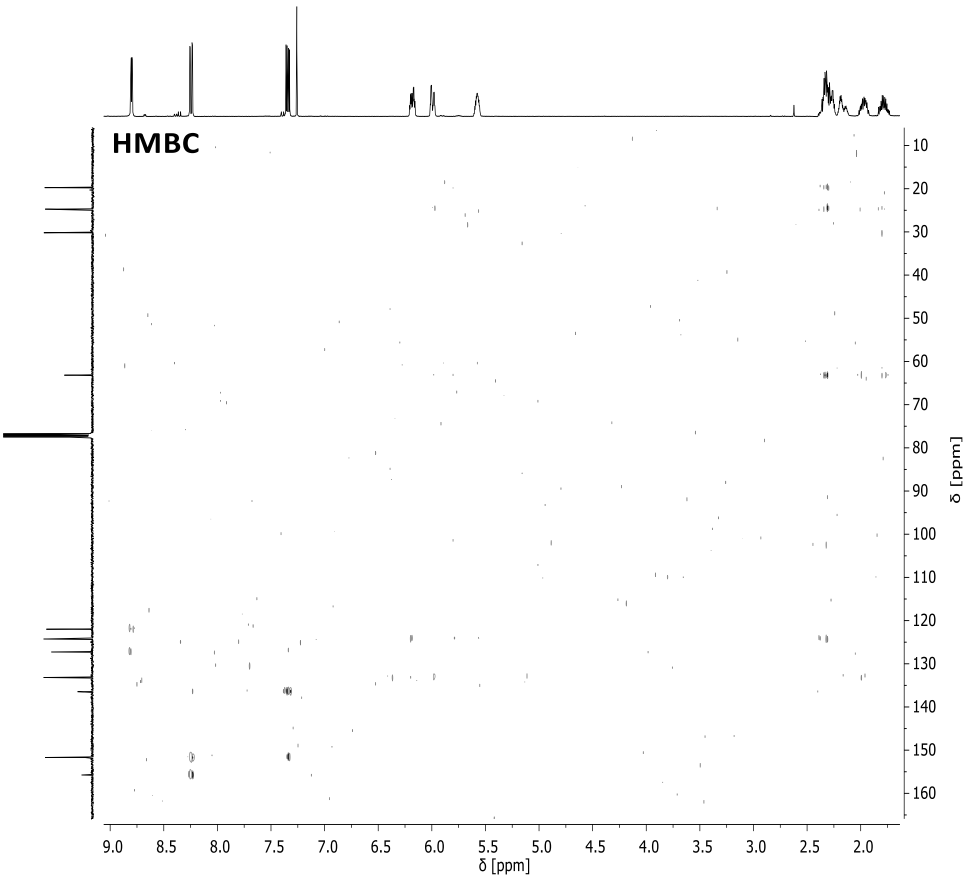

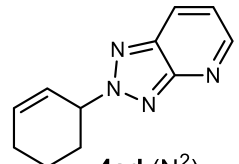

4ad (N^2)

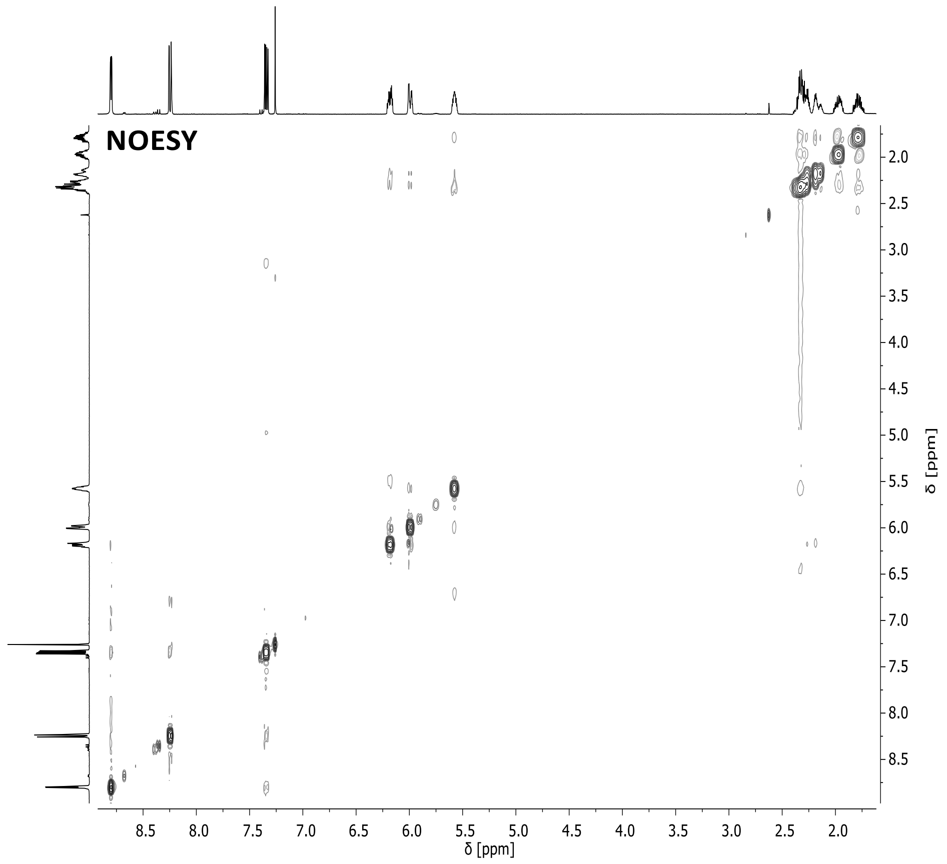

^{1}**H NMR** (400 MHz, $CDCl_3$) of **4ad** (N^1)

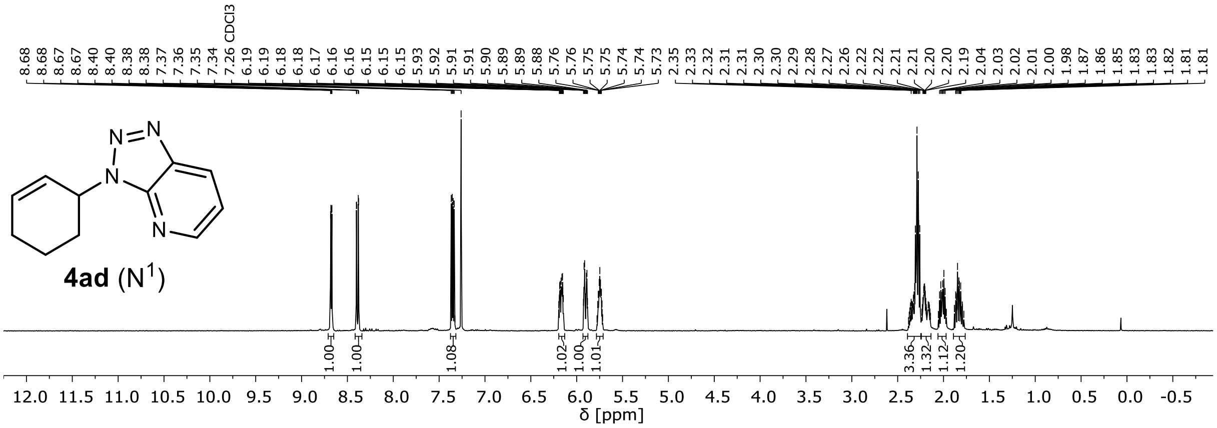

^{13}**C NMR** (101 MHz, $CDCl_3$) of **4ad** (N^1)

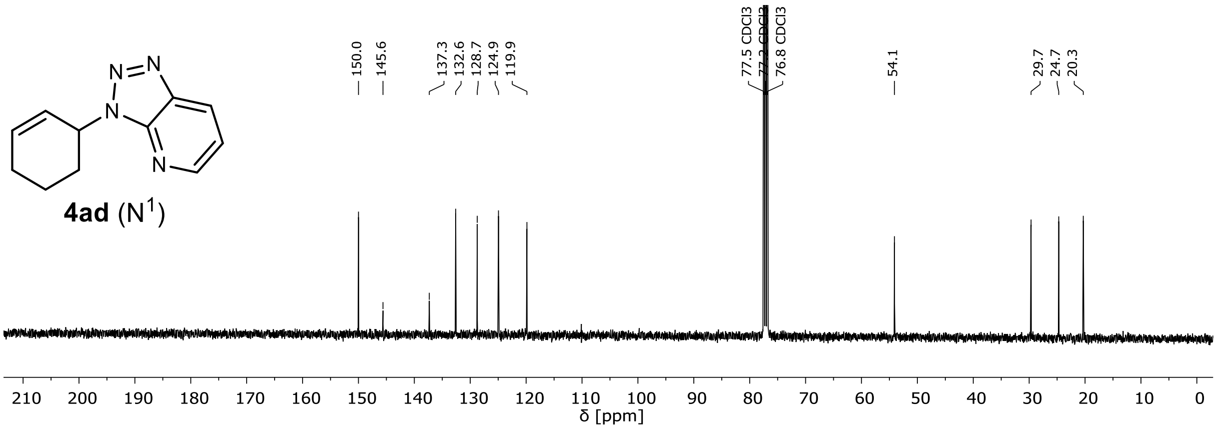

IR (ATR, neat) of **4ad** (N^1)

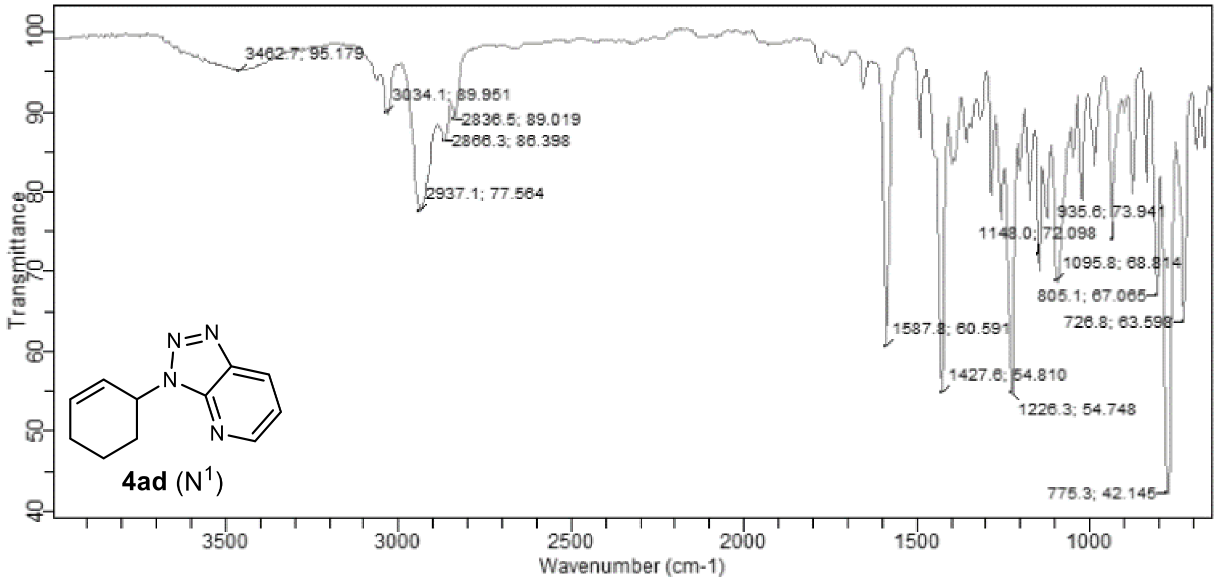

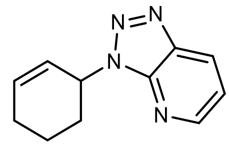

4ad (N[1])

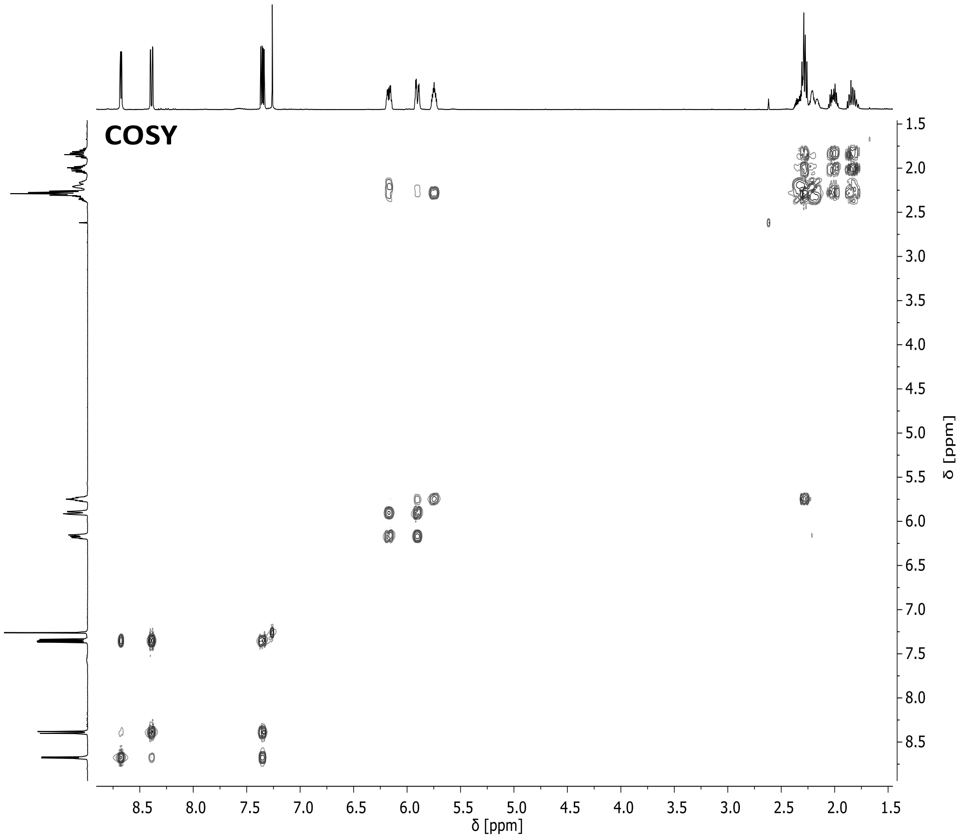

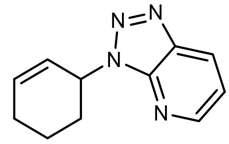

4ad (N[1])

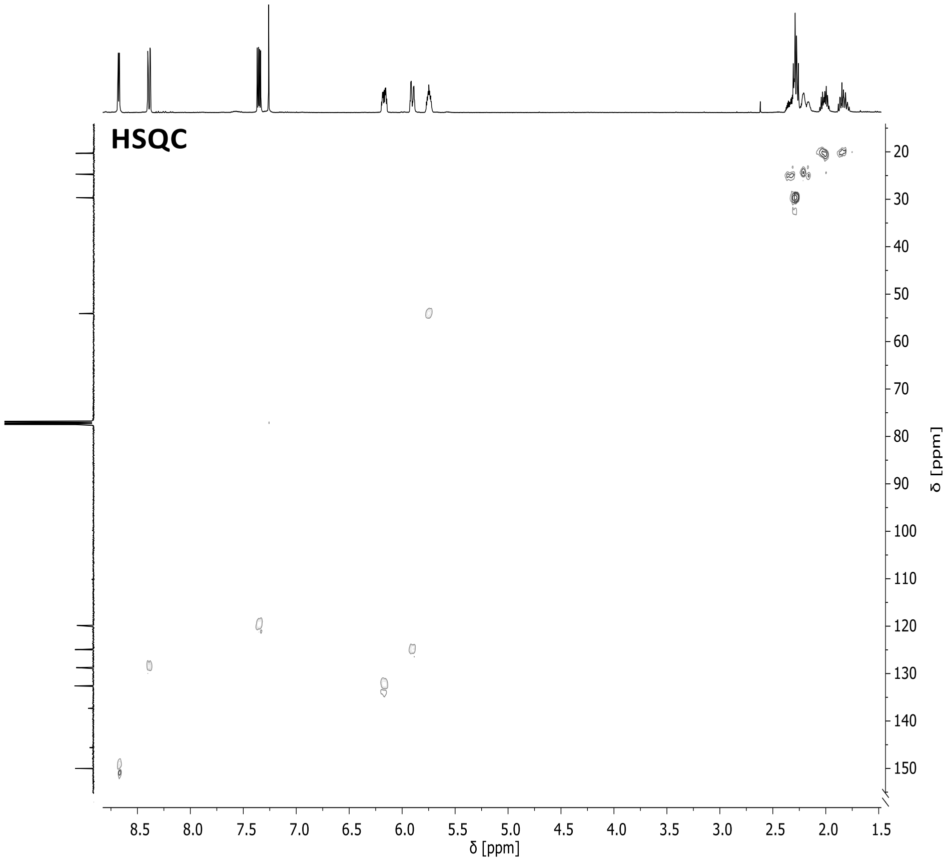

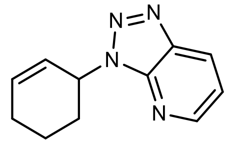

4ad (N[1])

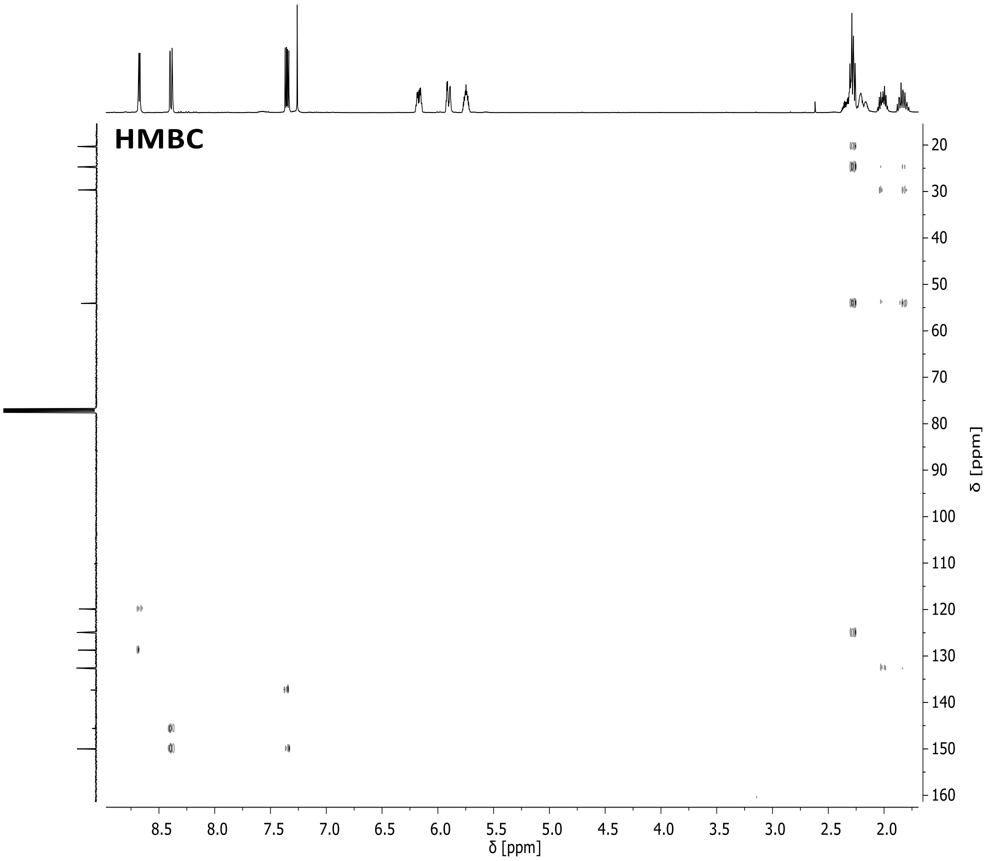

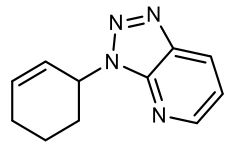

4ad (N[1])

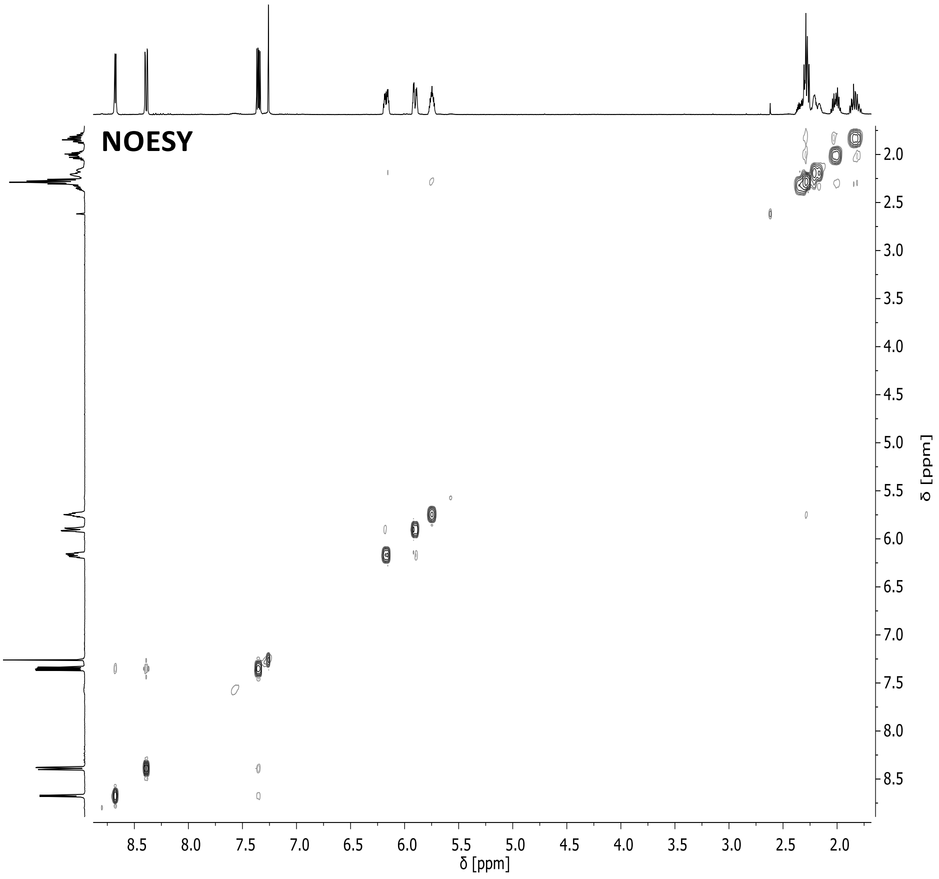

^{1}H NMR (400 MHz, $CDCl_3$) of **4ad** (N^3)

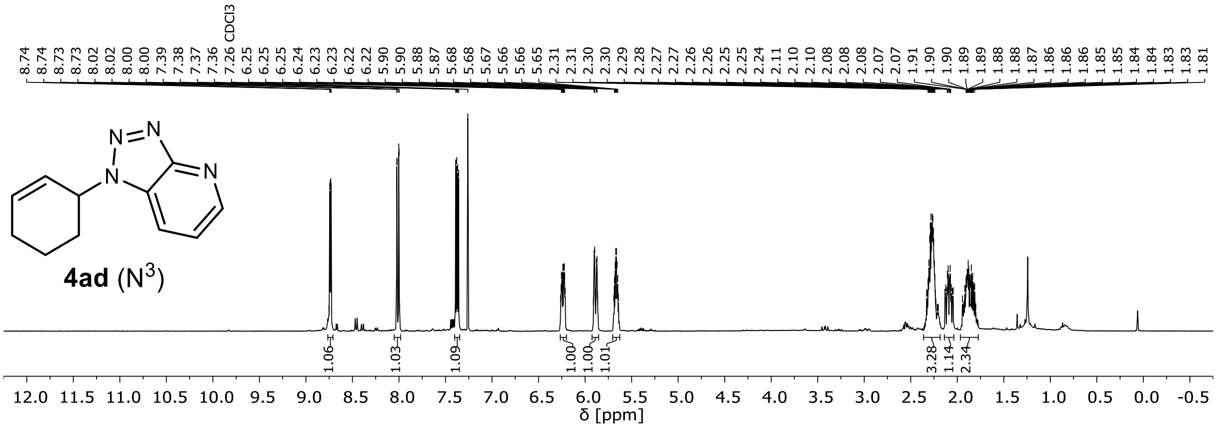

^{13}C NMR (101 MHz, $CDCl_3$) of **4ad** (N^3)

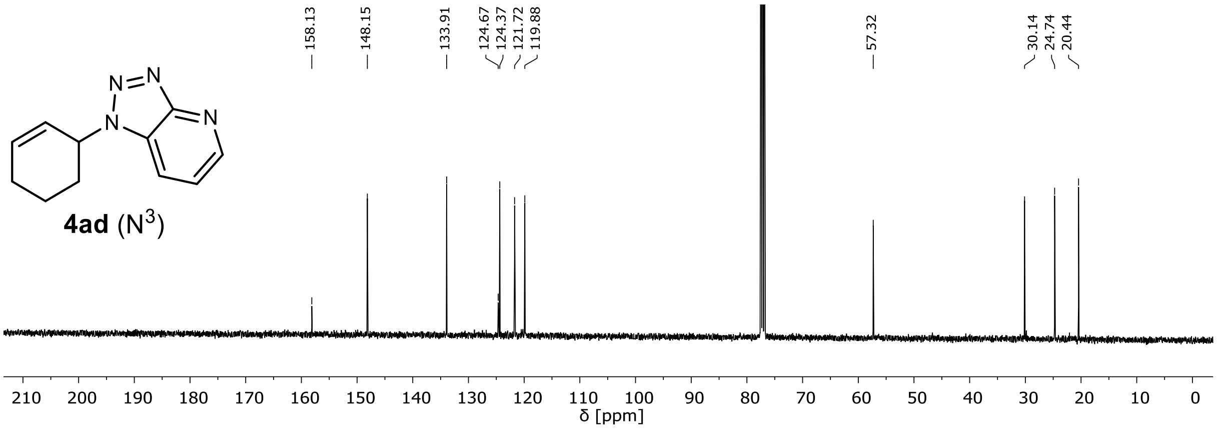

IR (ATR, neat) of **4ad** (N^3)

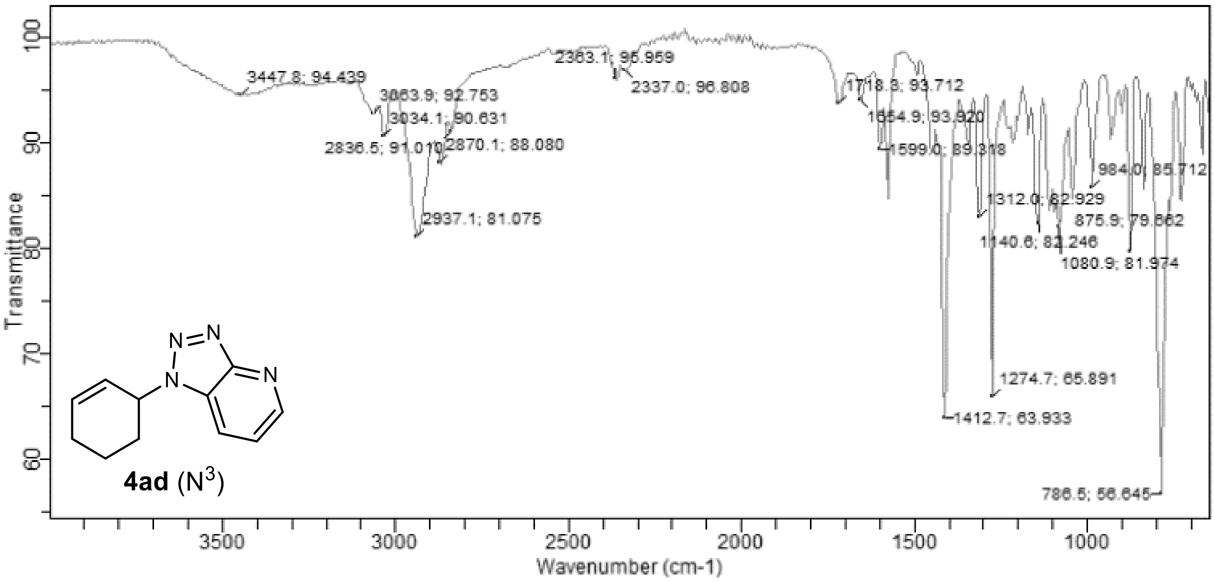

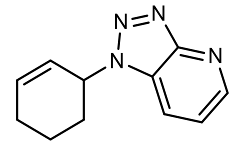

4ad (N^3)

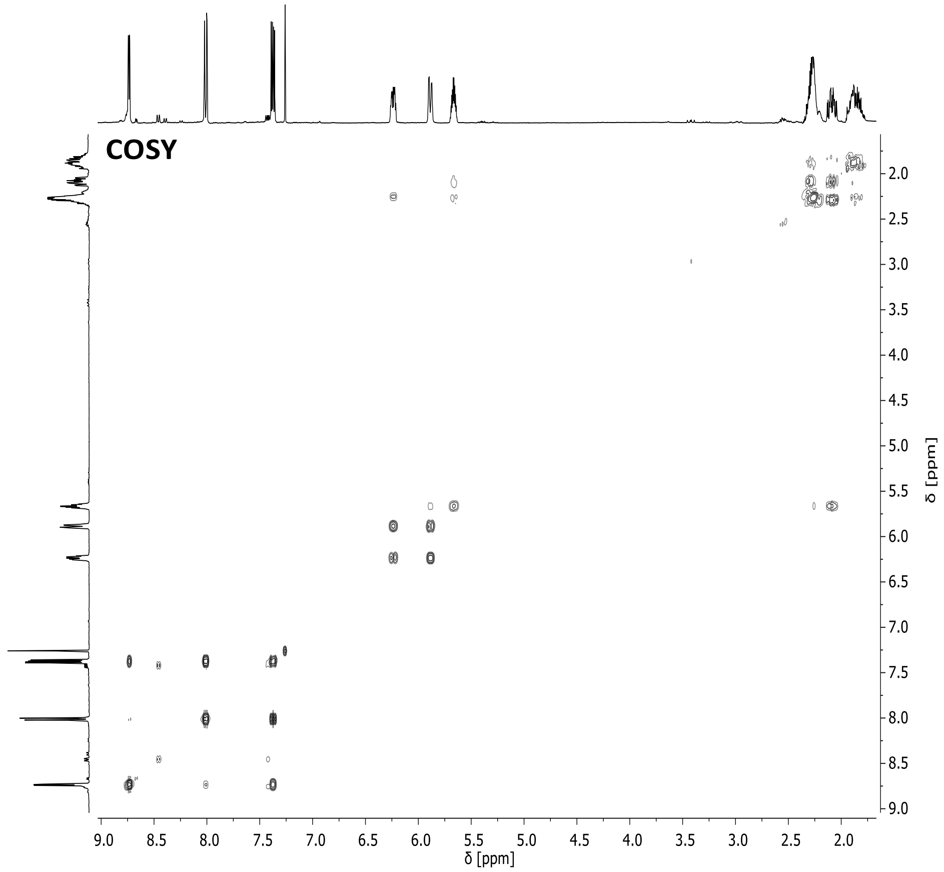

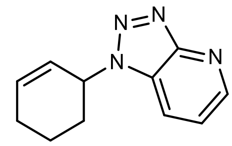

4ad (N^3)

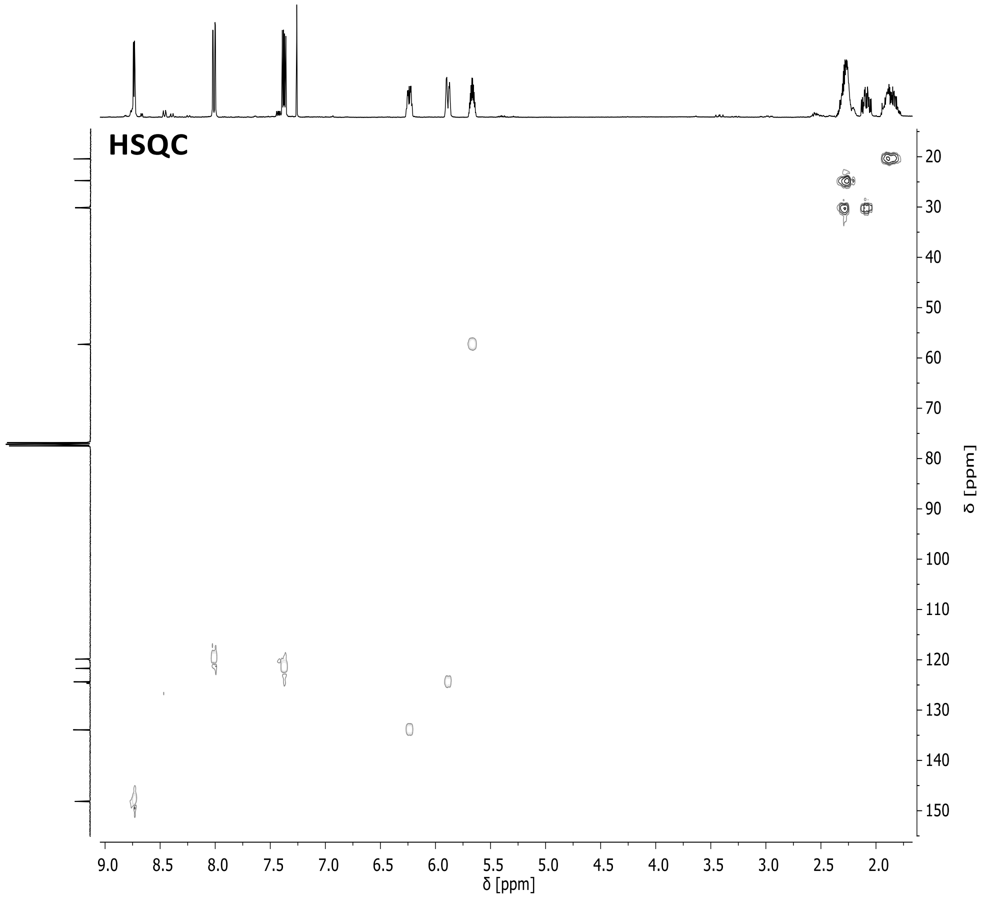

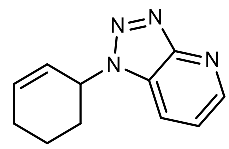

4ad (N^3)

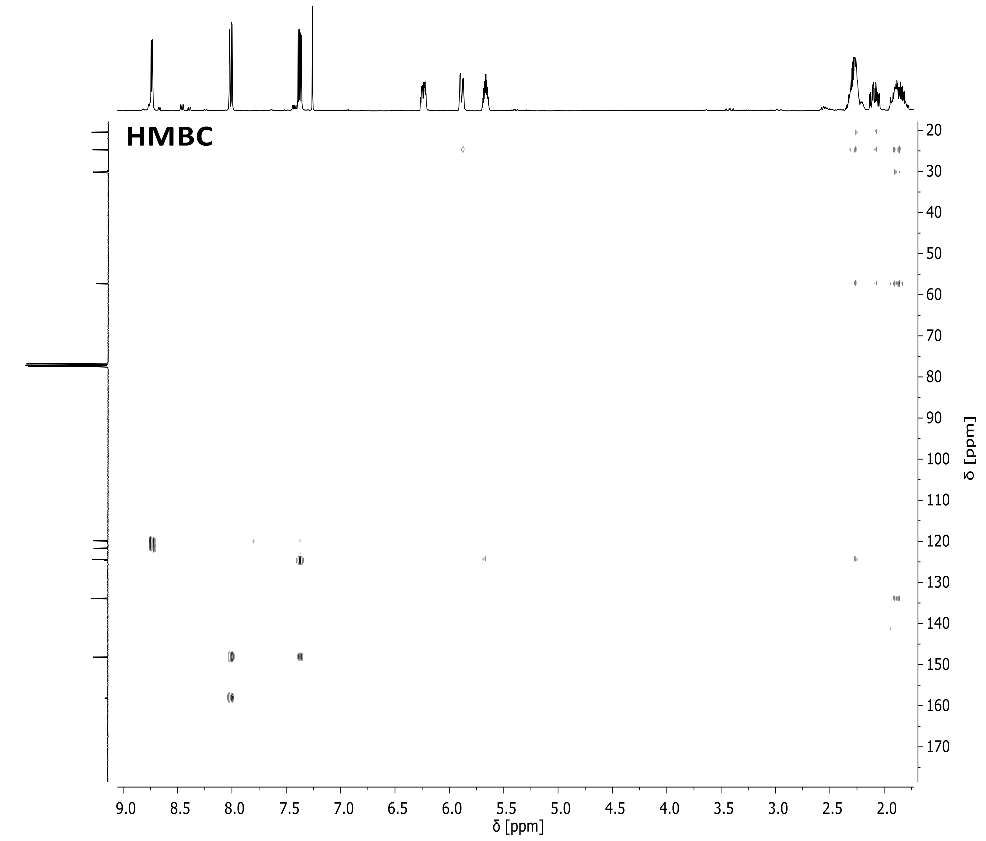

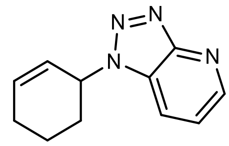

4ad (N^3)

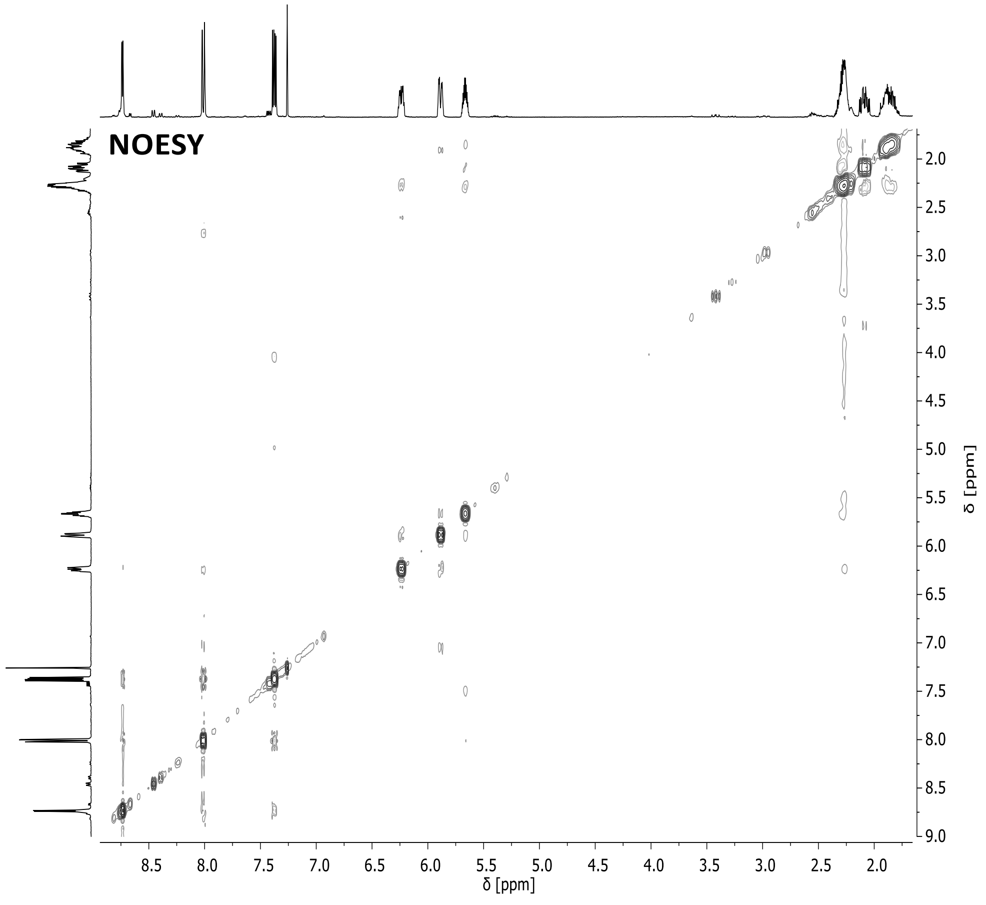

^{1}**H NMR** (300 MHz, $CDCl_3$) of **4ae** (N^2)

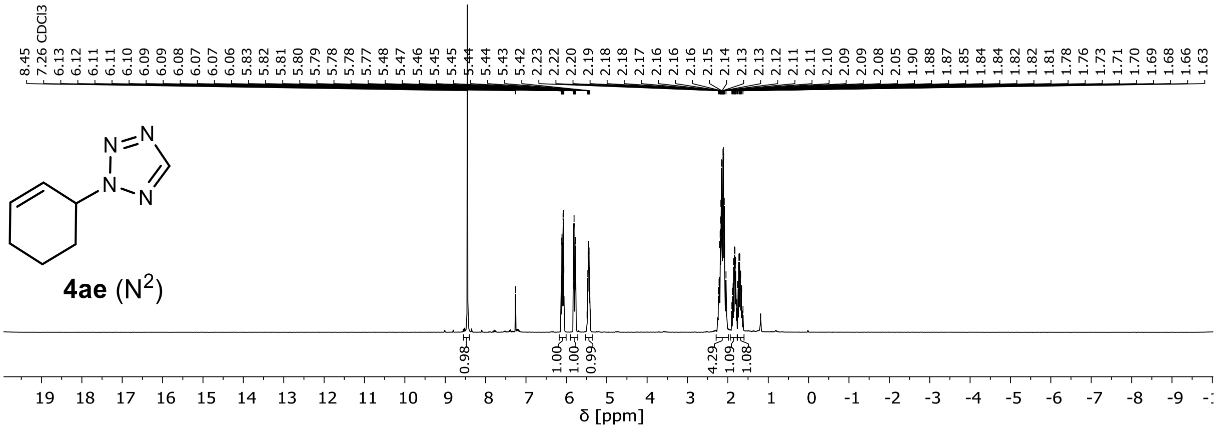

^{13}**C NMR** (75 MHz, $CDCl_3$) of **4ae** (N^2)

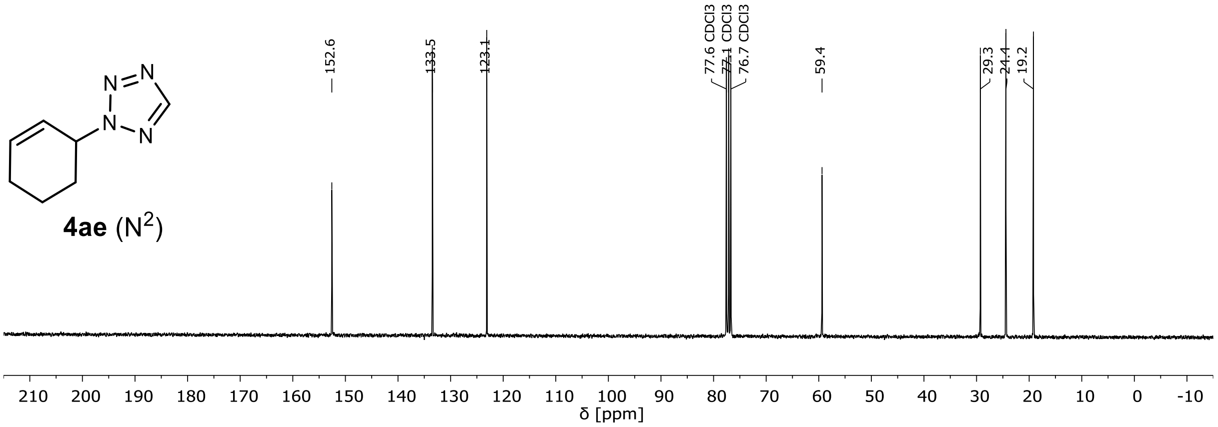

IR (ATR, neat) of **4ae** (N^2)

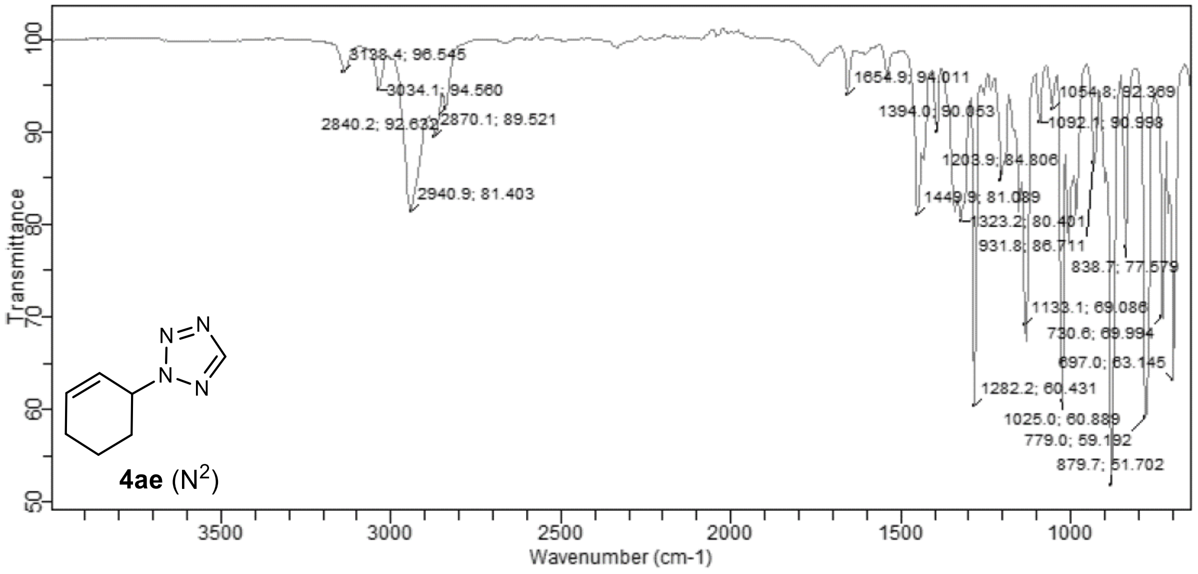

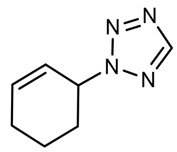

4ae (N^2)

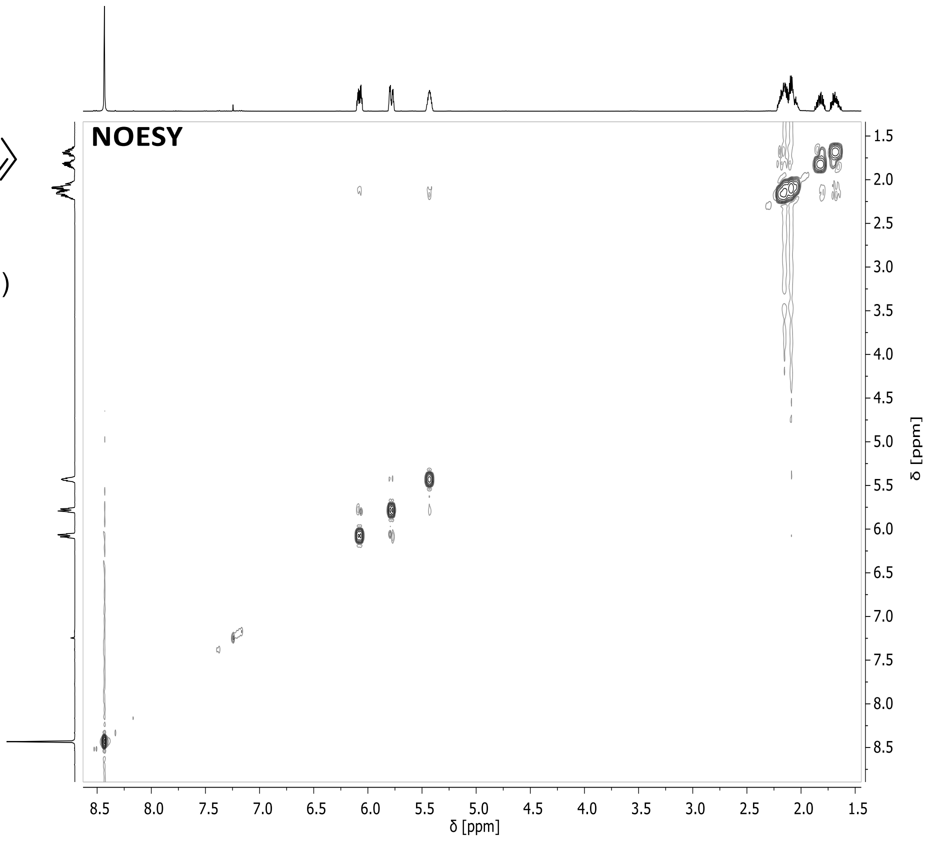

¹H NMR (300 MHz, $CDCl_3$) of **4ae** (N[1])

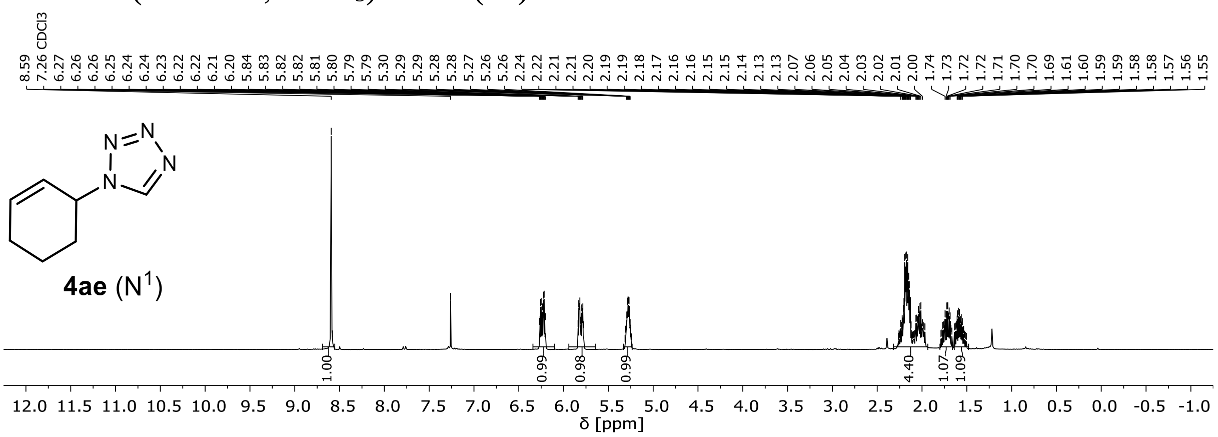

¹³C NMR (75 MHz, $CDCl_3$) of **4ae** (N[1])

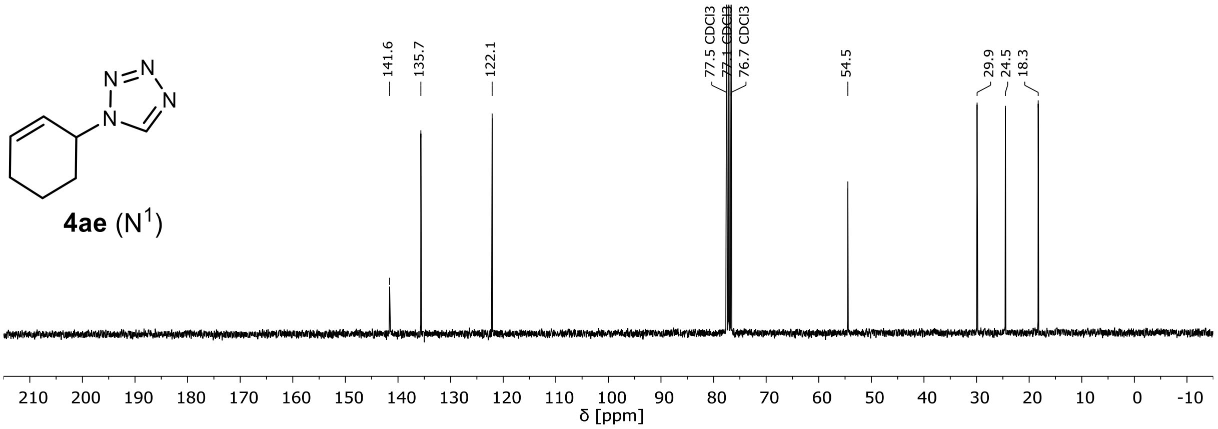

IR (ATR, neat) of **4ae** (N[1])

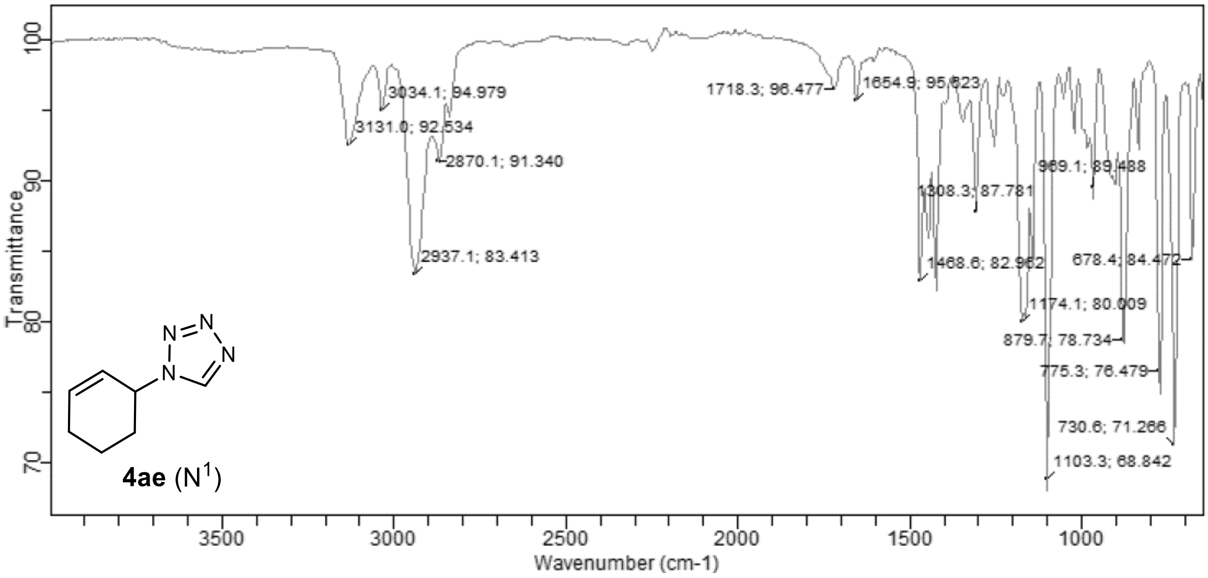

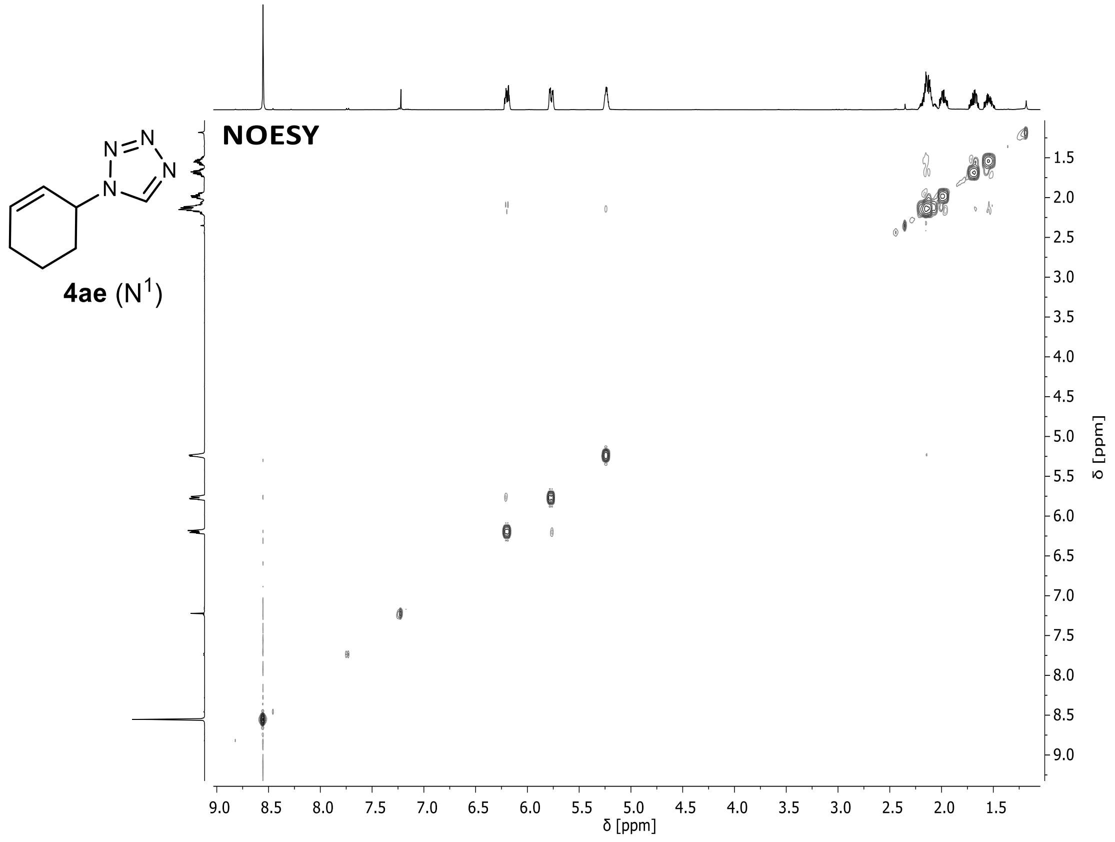
NOESY
4ae (N1)
δ [ppm]
δ [ppm]

^{1}H NMR (300 MHz, $CDCl_3$) of **4af** (N^2)

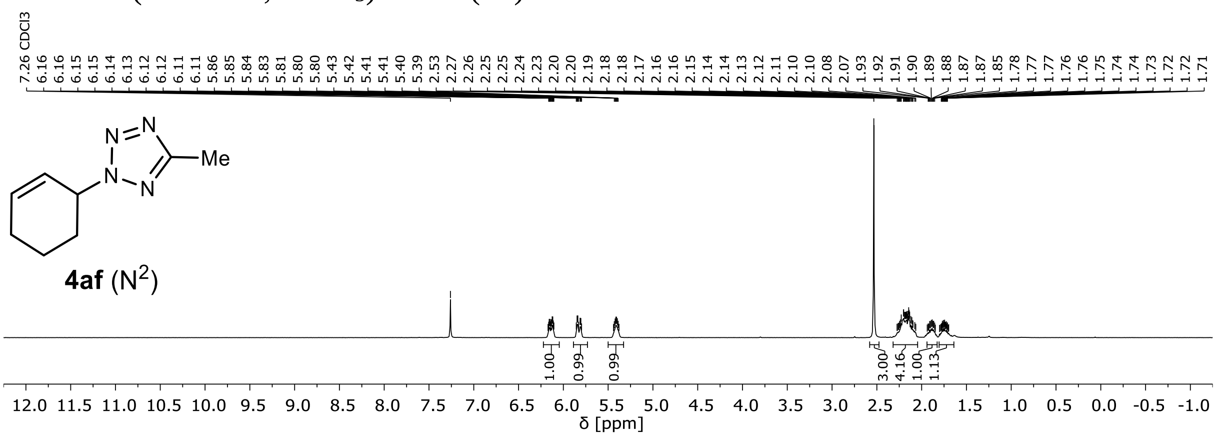

^{13}C NMR (101 MHz, $CDCl_3$) of **4af** (N^2)

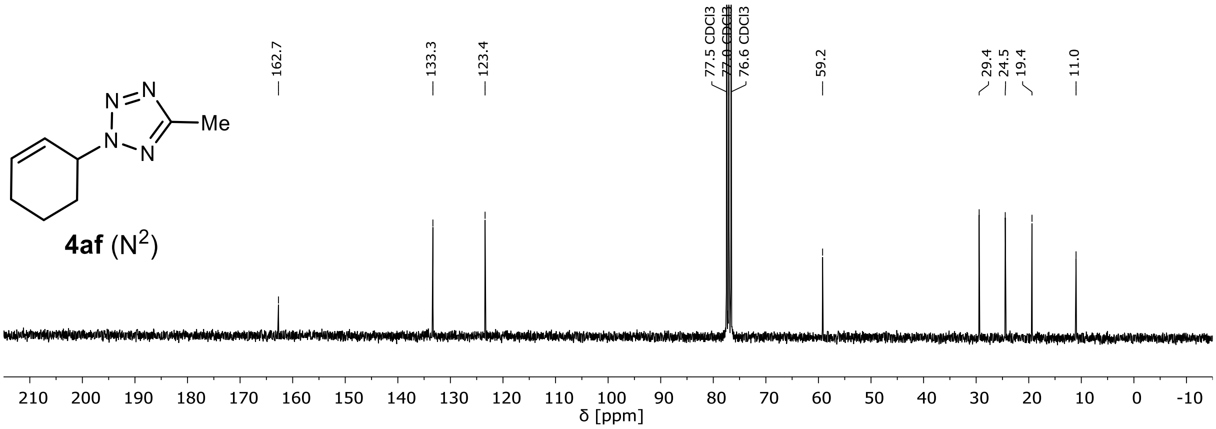

IR (ATR, neat) of **4af** (N^2)

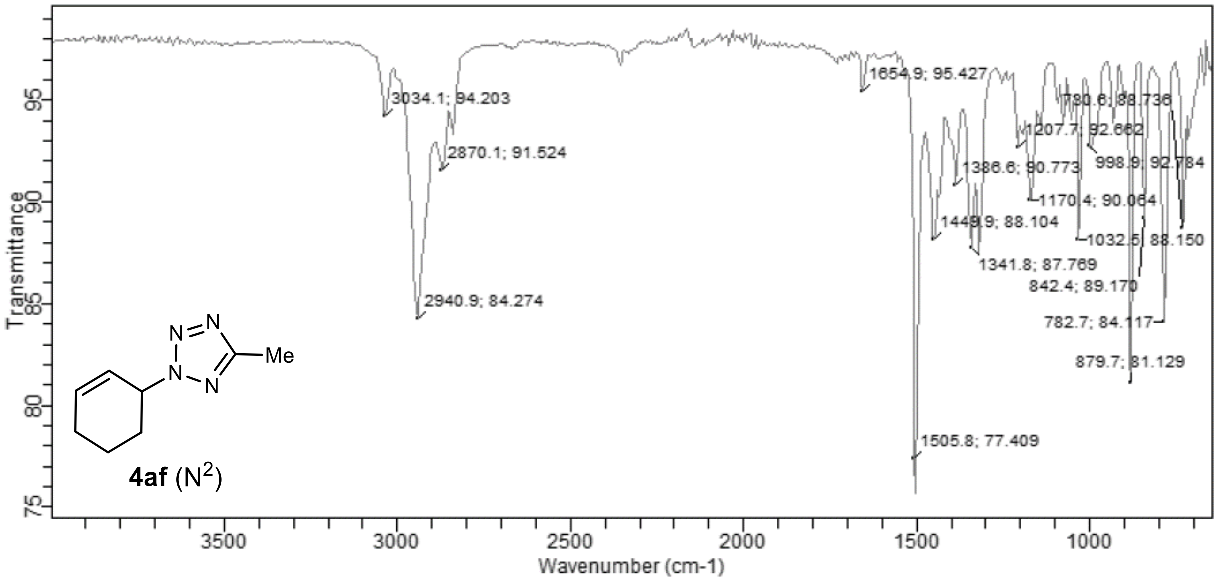

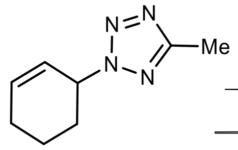

4af (N^2)

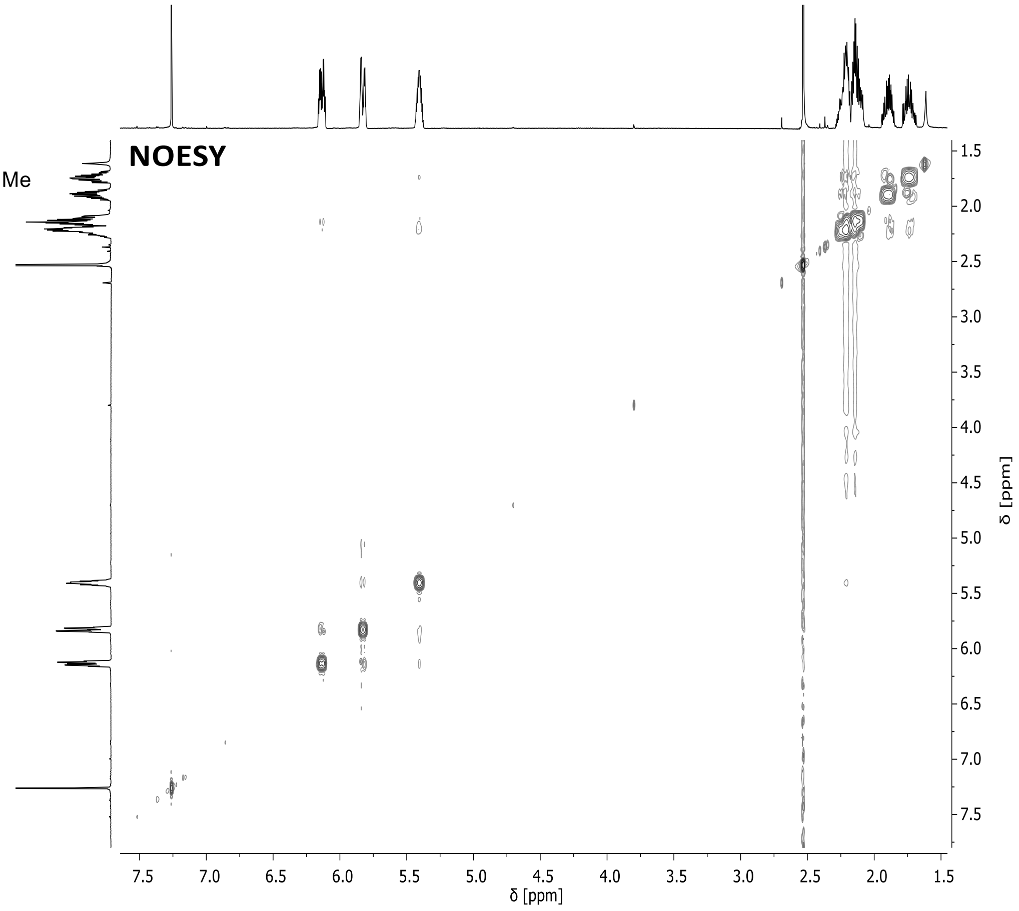

^{1}H NMR (300 MHz, $CDCl_3$) of **4af** (N[1])

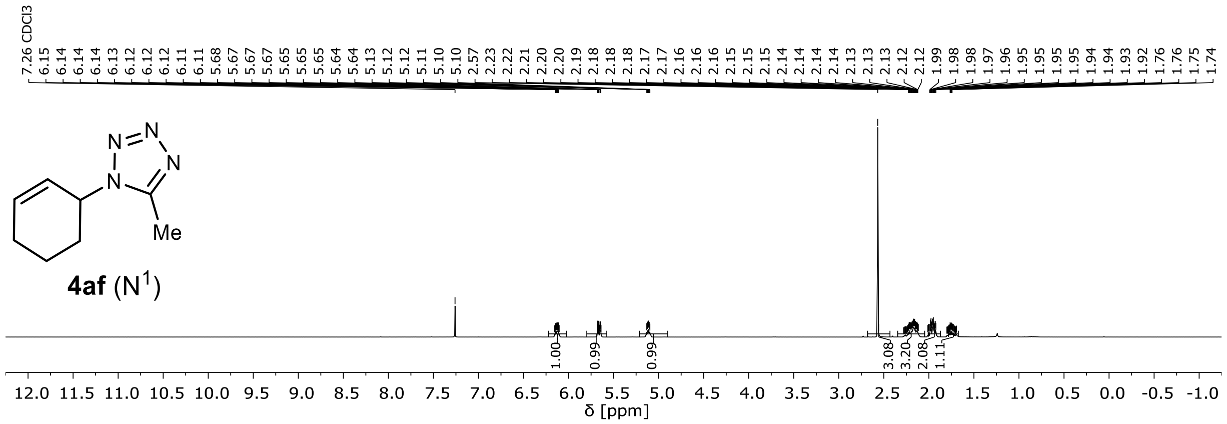

^{13}C NMR (101 MHz, $CDCl_3$) of **4af** (N[1])

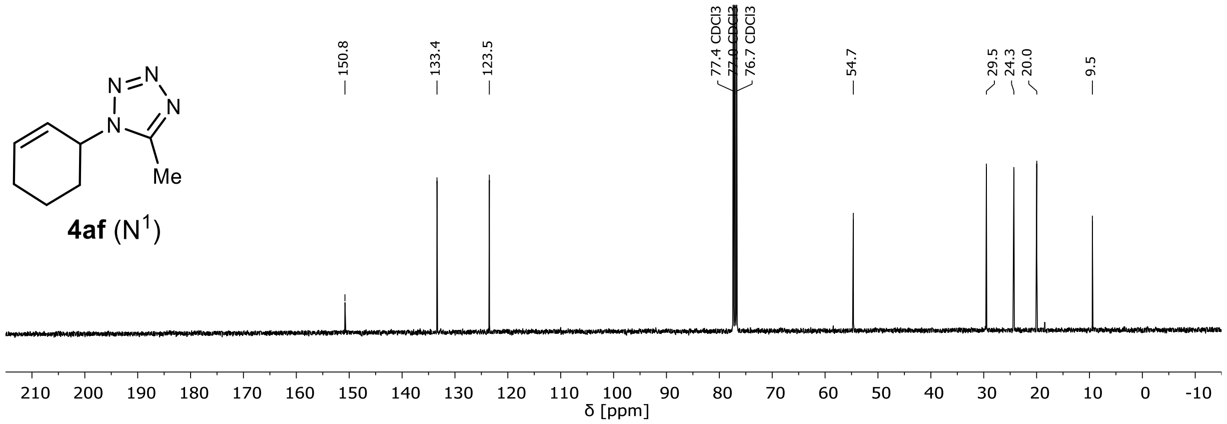

IR (ATR, neat) of **4af** (N[1])

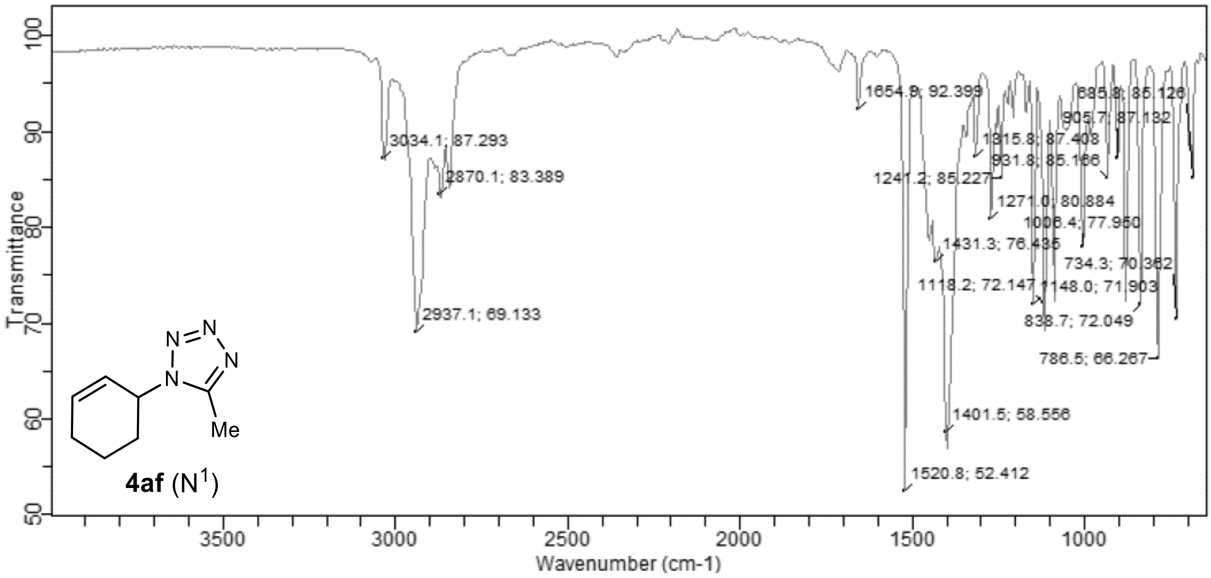

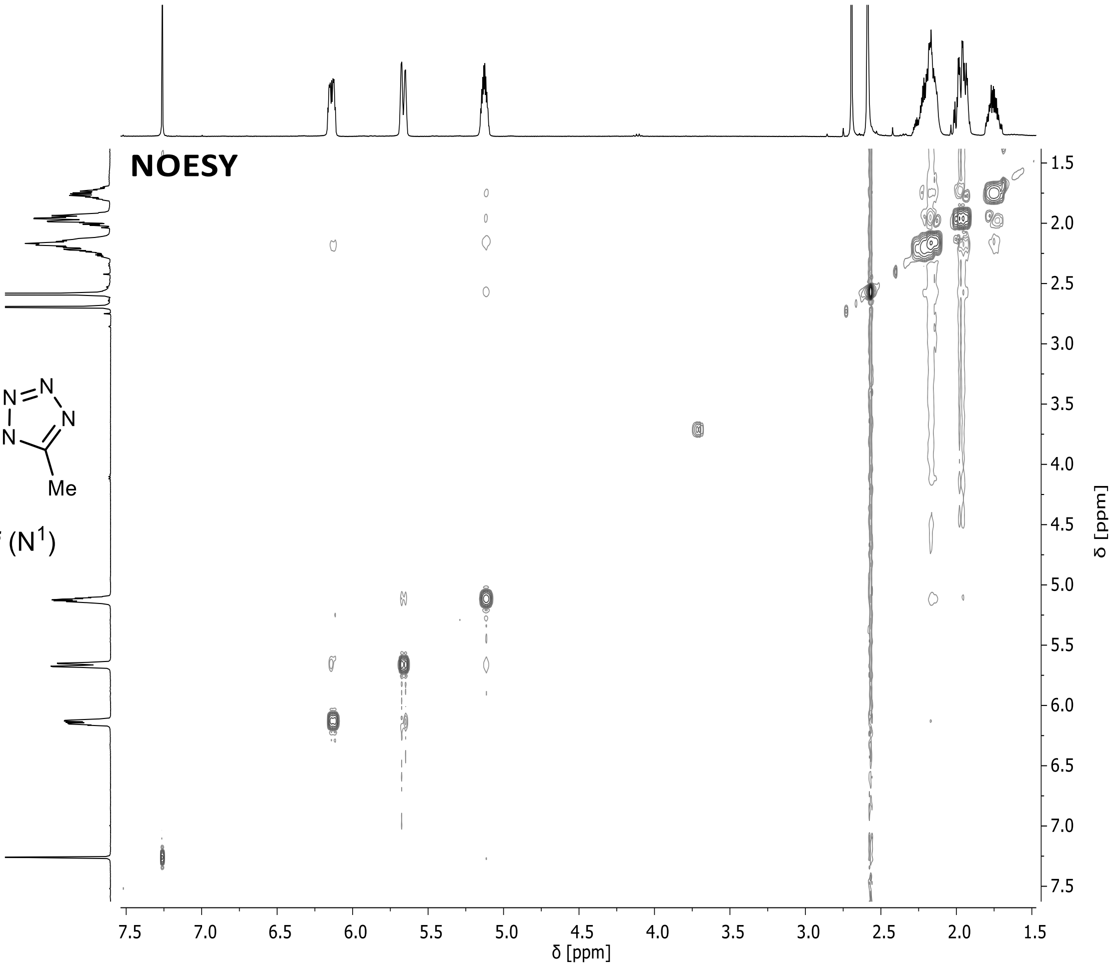

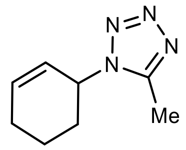

4af (N[1])

^{1}H NMR (300 MHz, $CDCl_3$) of **4ag**

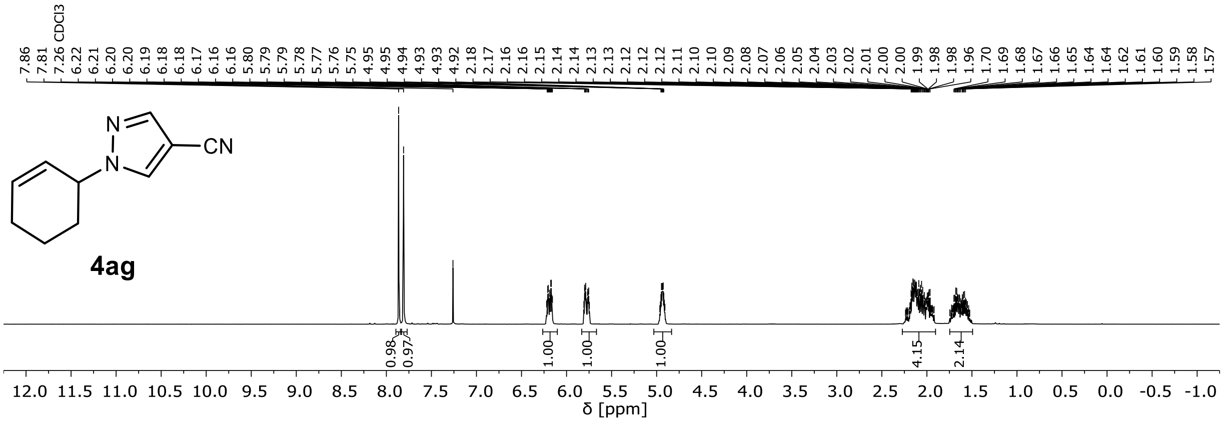

^{13}C NMR (75 MHz, $CDCl_3$) of **4ag**

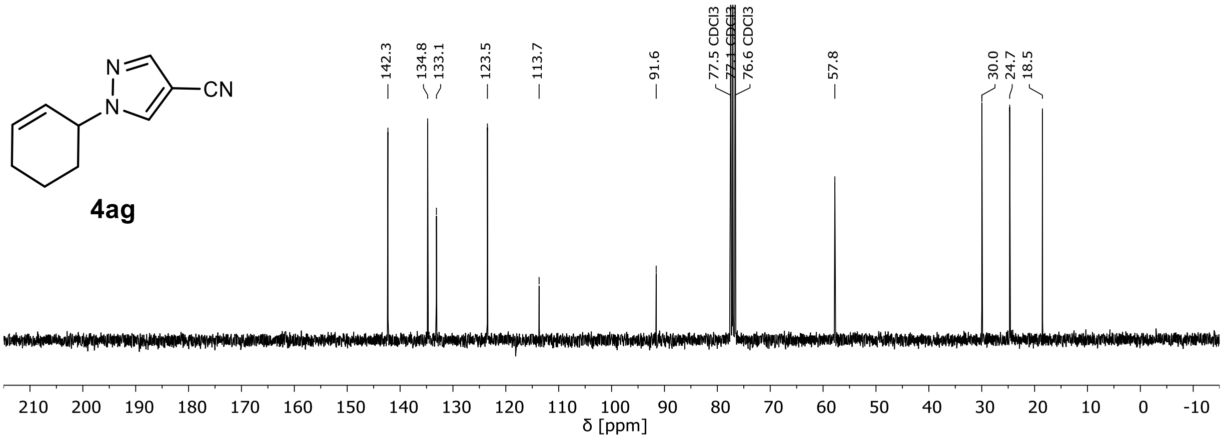

IR (ATR, neat) of **4ag**

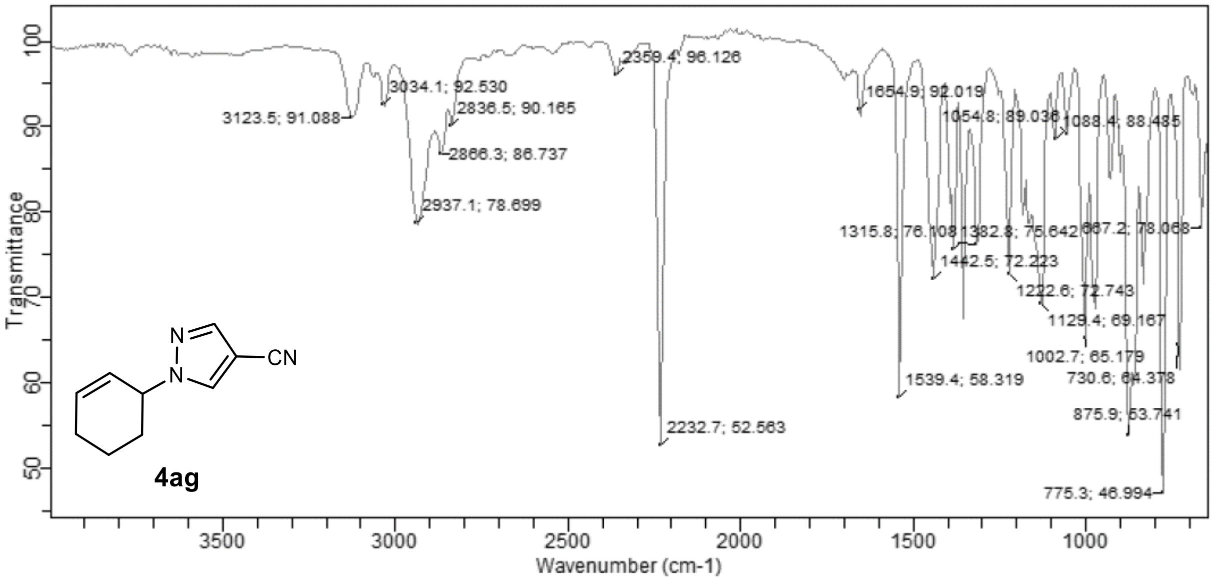

^{1}H NMR (300 MHz, $CDCl_3$) of **4ah**

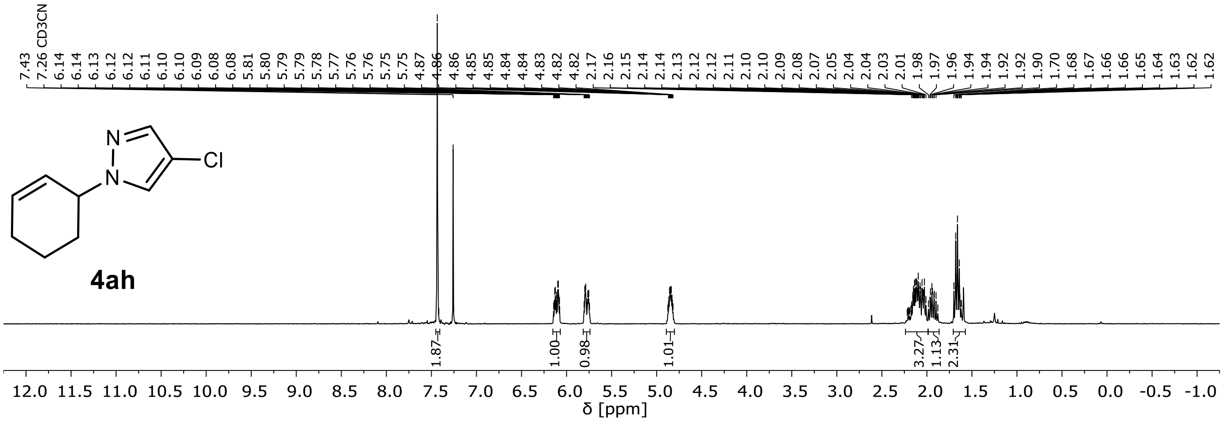

^{13}C NMR (75 MHz, $CDCl_3$) of **4ah**

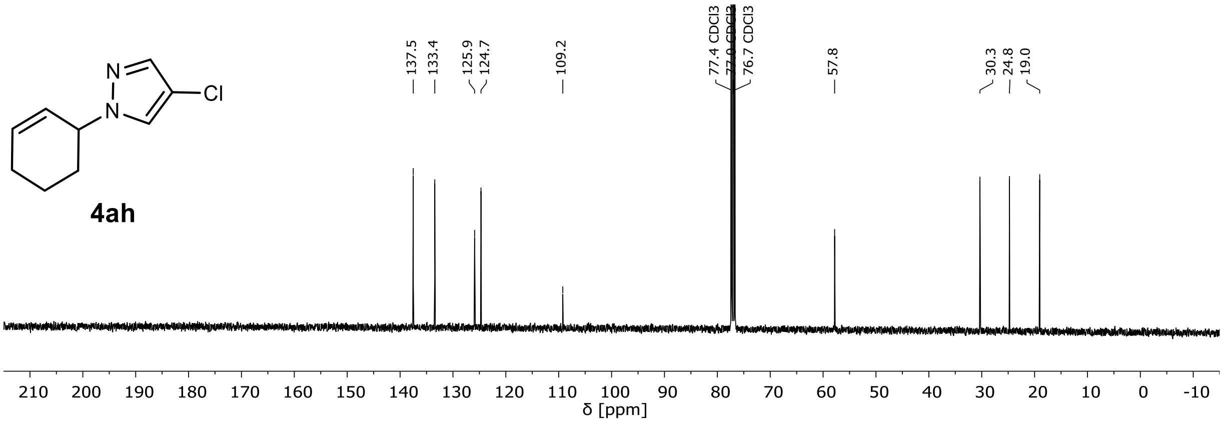

IR (ATR, neat) of **4ah**

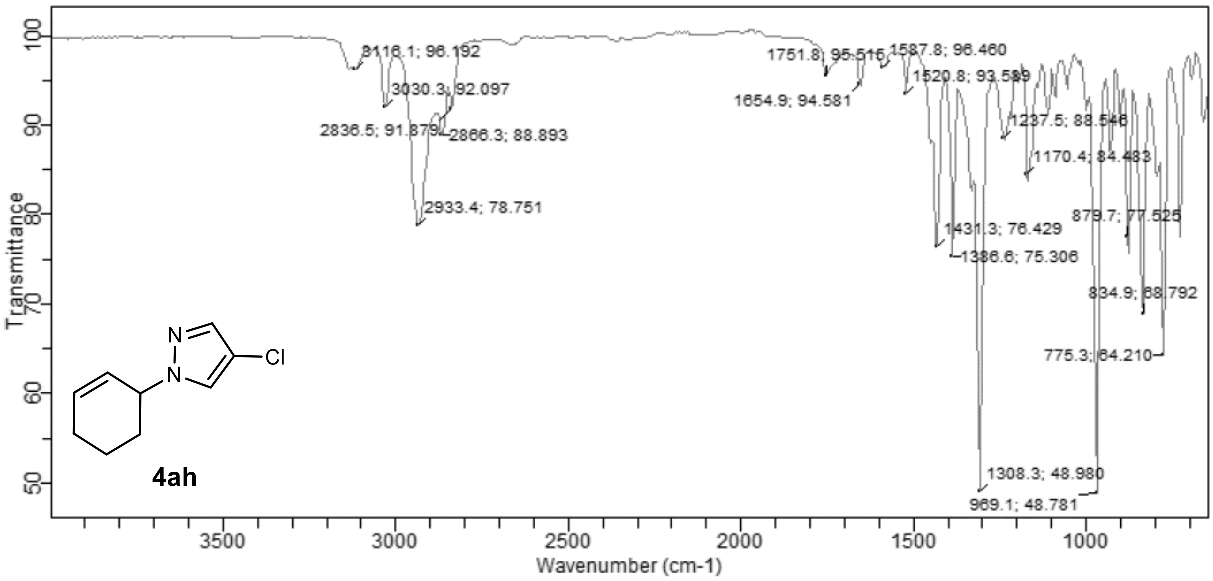

^{1}H NMR (400 MHz, $CDCl_3$) of **4ai** (N^2)

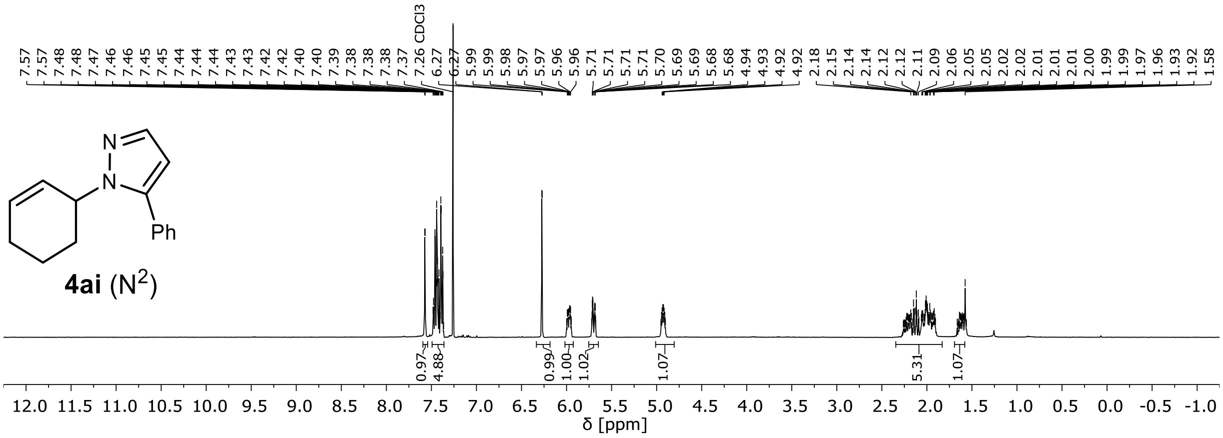

^{13}C NMR (101 MHz, $CDCl_3$) of **4ai** (N^2)

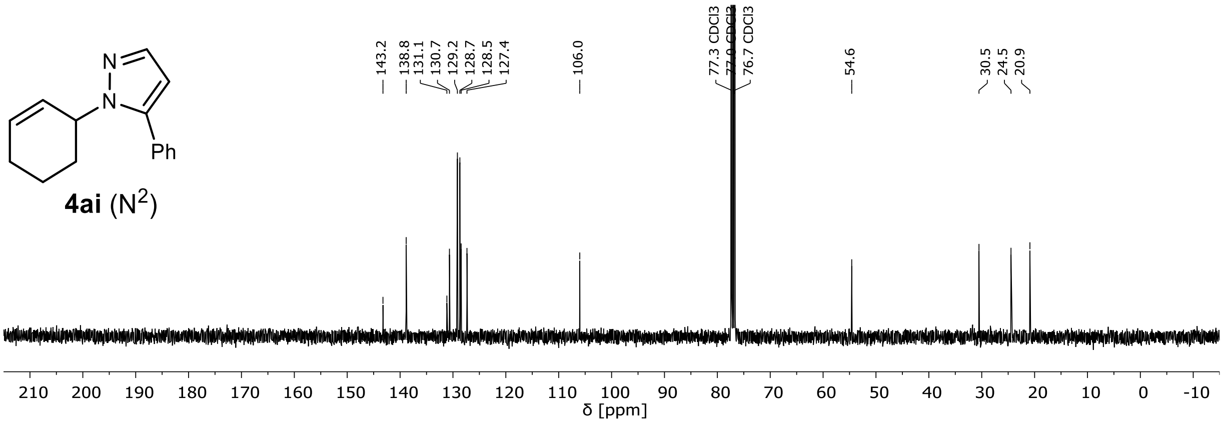

IR (ATR, neat) of **4ai** (N^2)

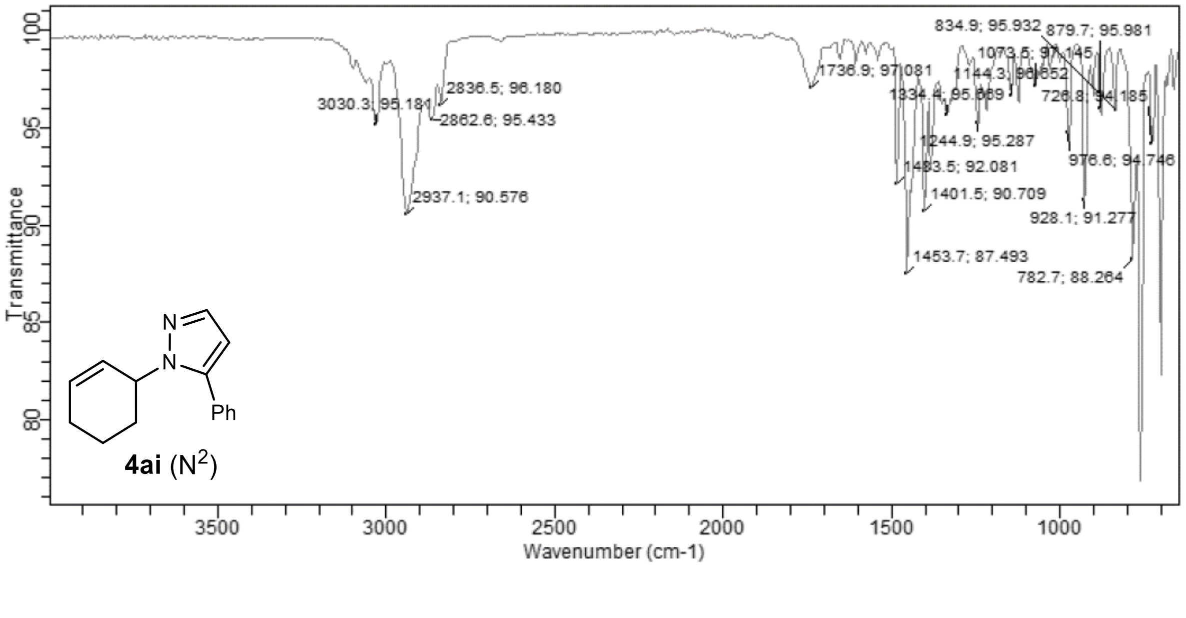

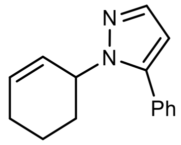

4ai (N^2)

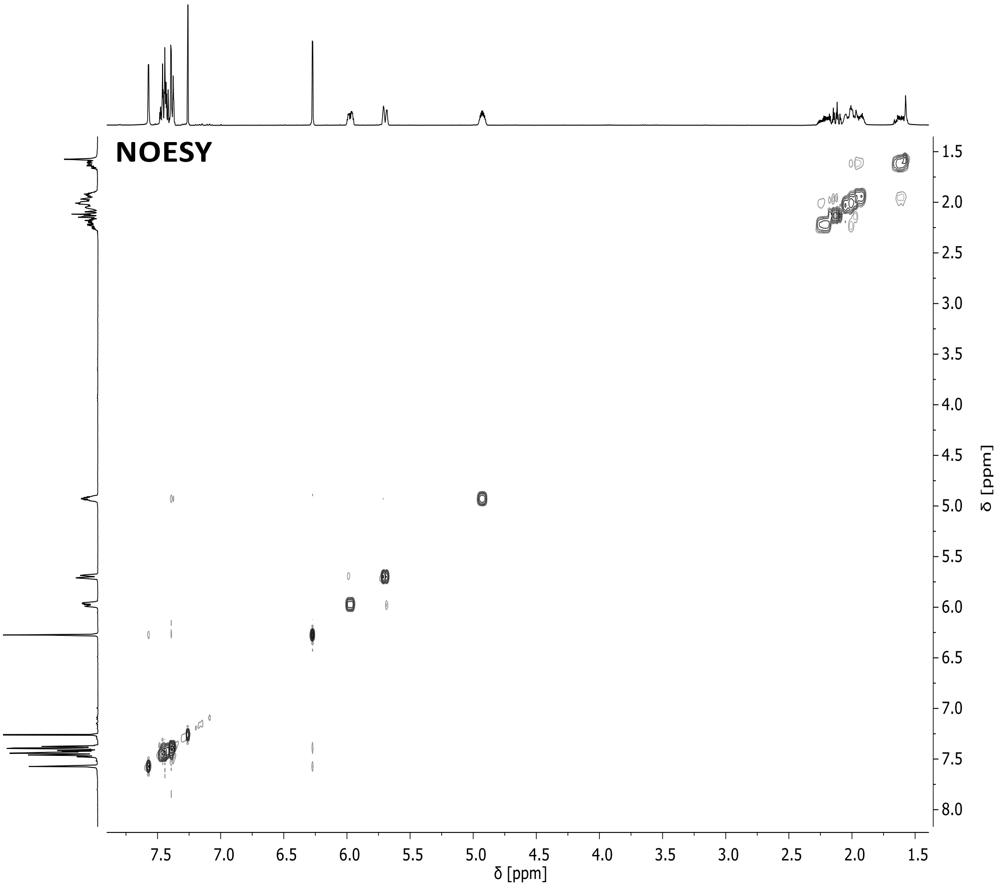

^{1}H NMR (400 MHz, CDCl$_3$) of **4ai** (N[1])

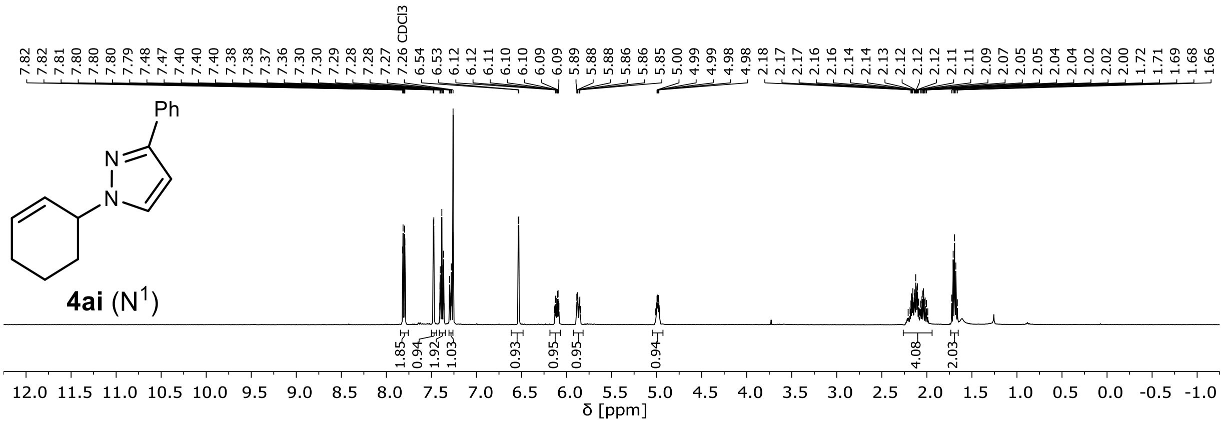

^{13}C NMR (101 MHz, CDCl$_3$) of **4ai** (N[1])

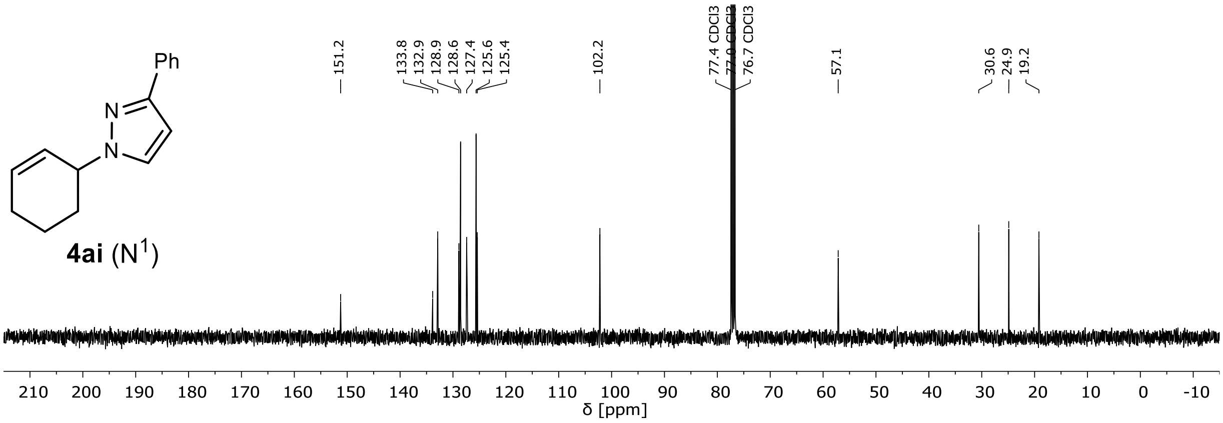

IR (ATR, neat) of **4ai** (N[1])

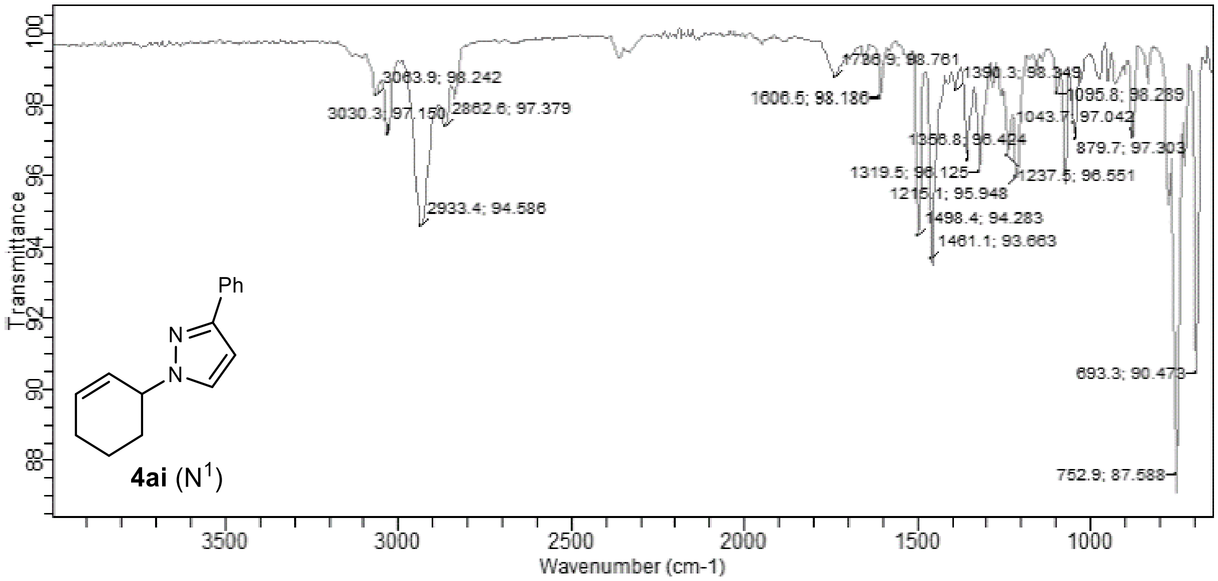

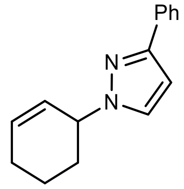

4ai (N[1])

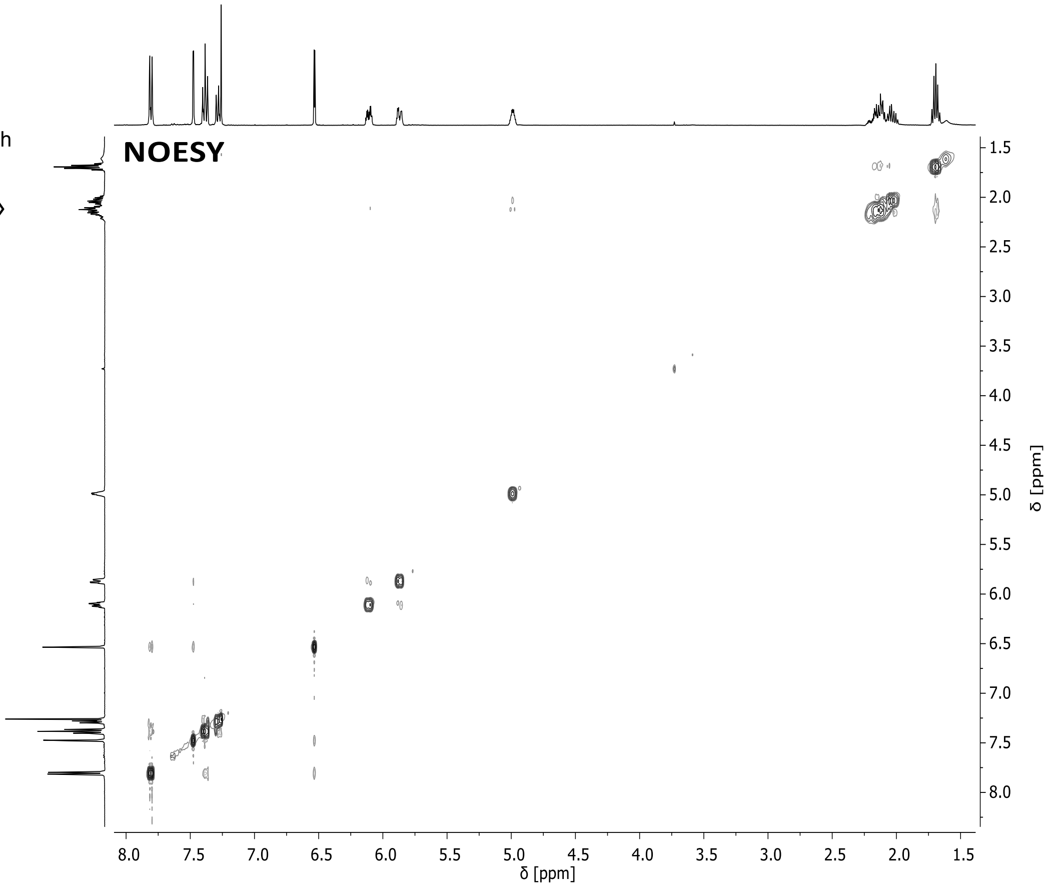

^{1}H NMR (300 MHz, $CDCl_3$) of **4aj**

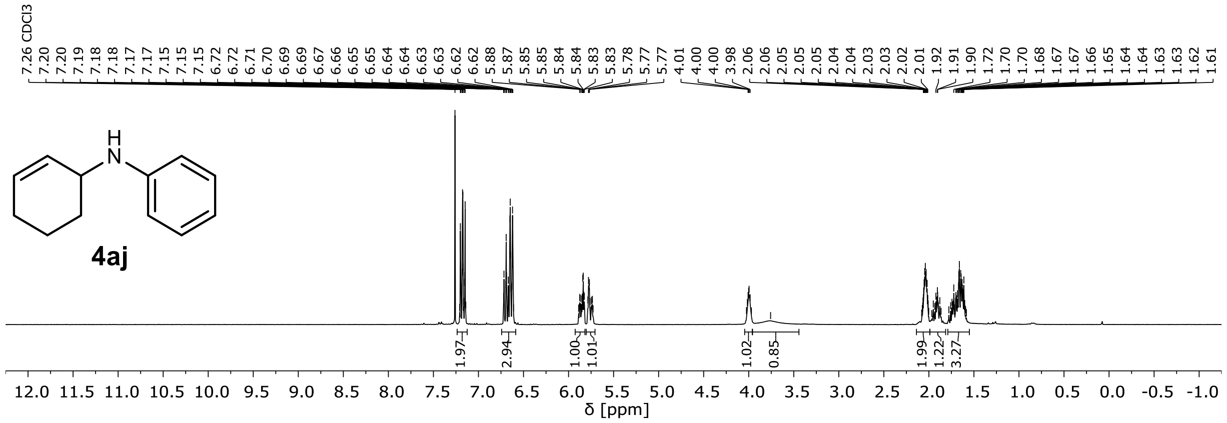

^{13}C NMR (75 MHz, $CDCl_3$) of **4aj**

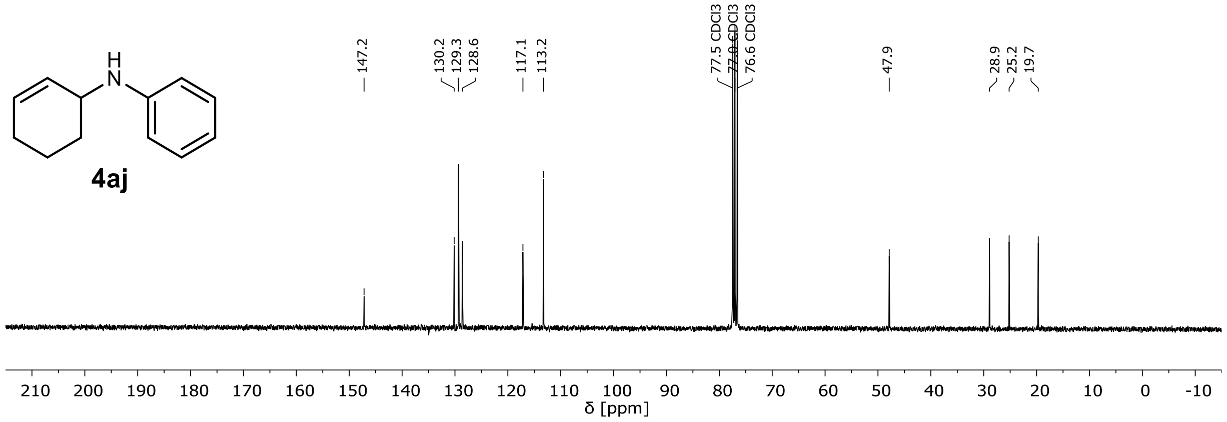

IR (ATR, neat) of **4aj**

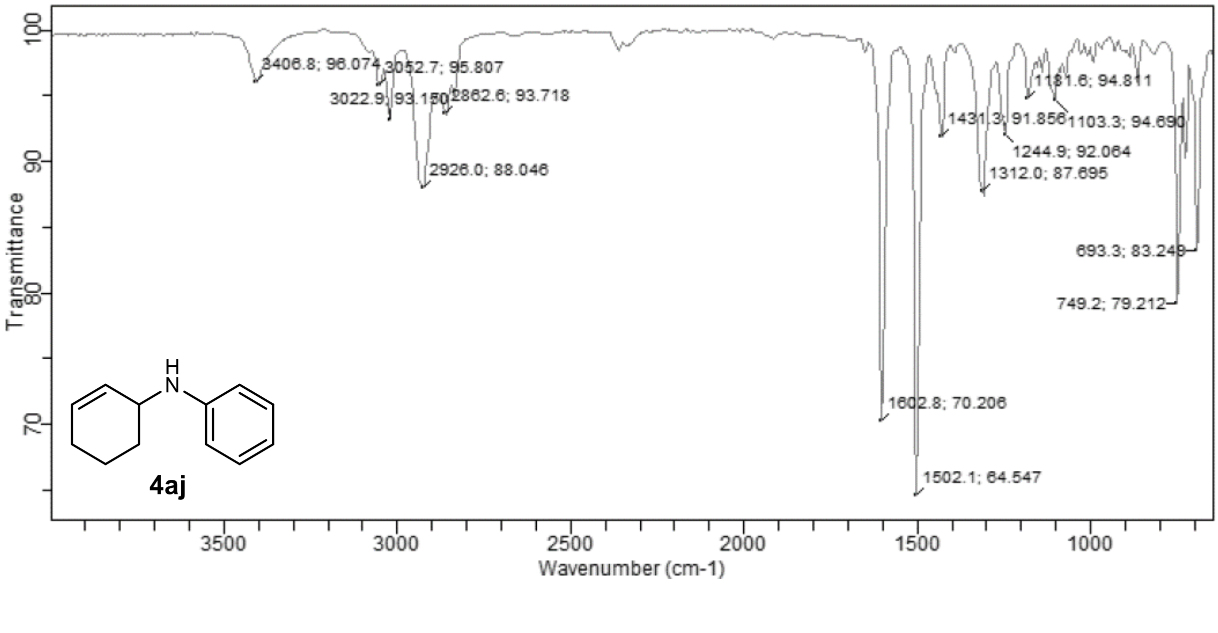

^{1}H NMR (400 MHz, $CDCl_3$) of **4ak**

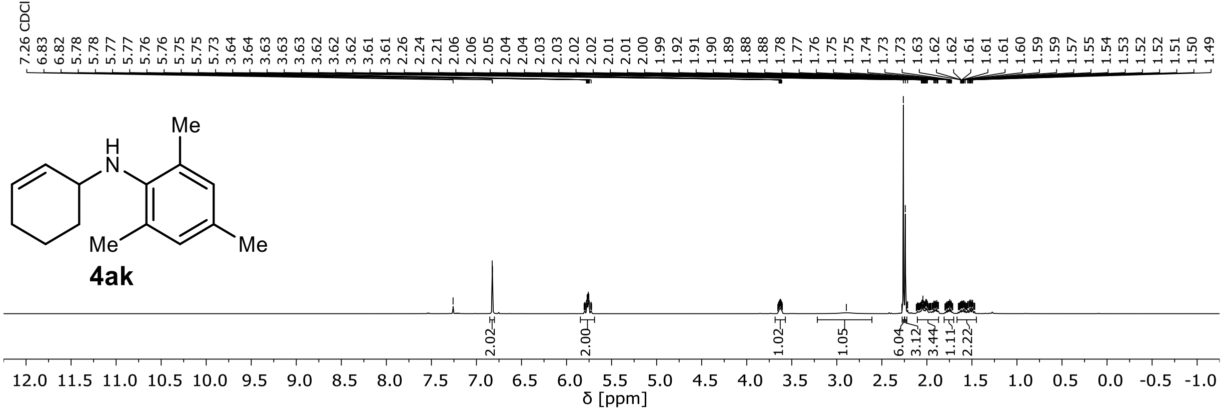

^{13}C NMR (101 MHz, $CDCl_3$) of **4ak**

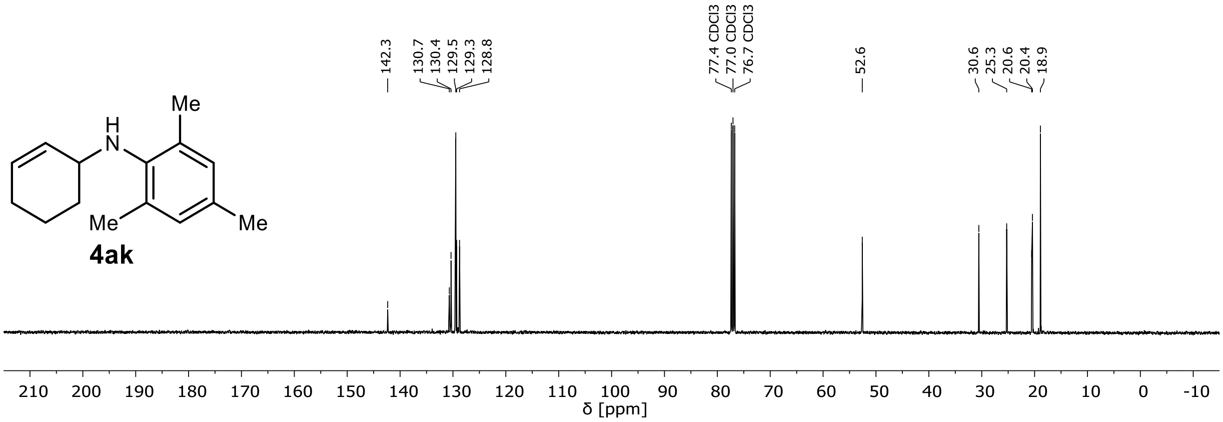

IR (ATR, neat) of **4ak**

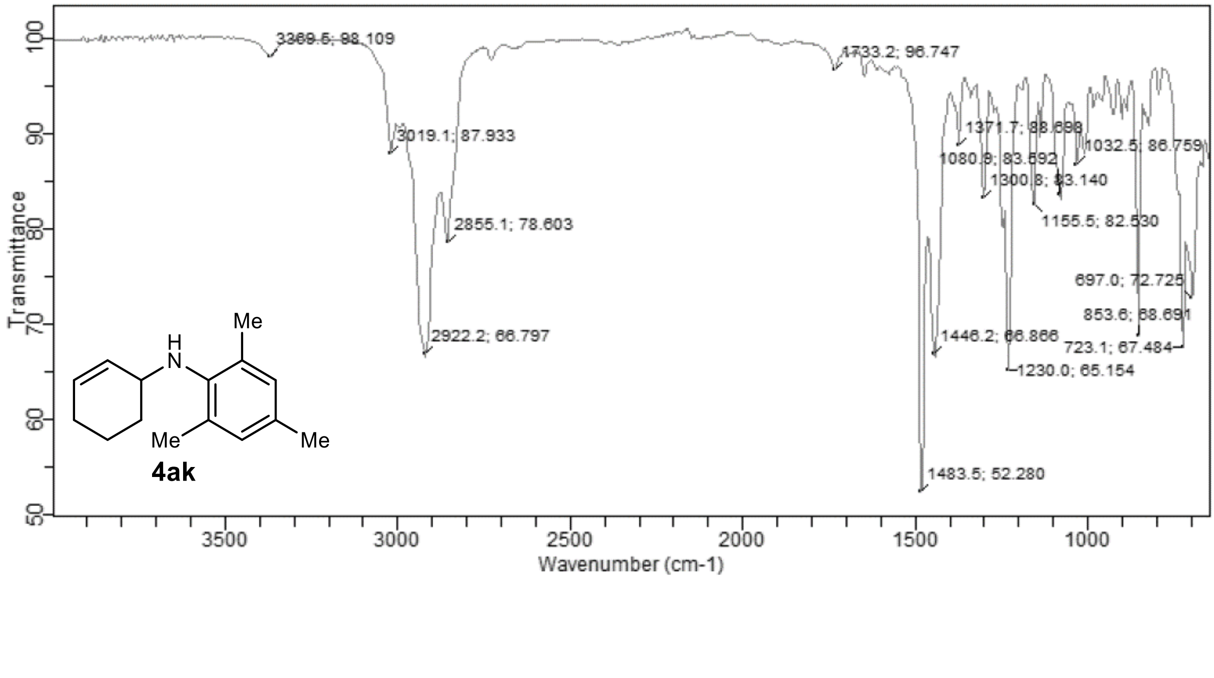

^{1}H NMR (400 MHz, $CDCl_3$) of **4al**

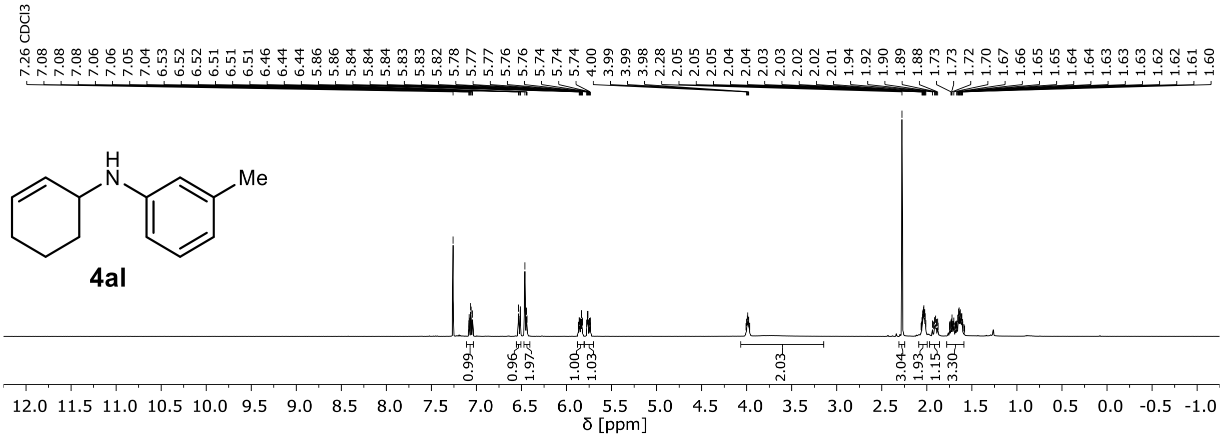

^{13}C NMR (101 MHz, $CDCl_3$) of **4al**

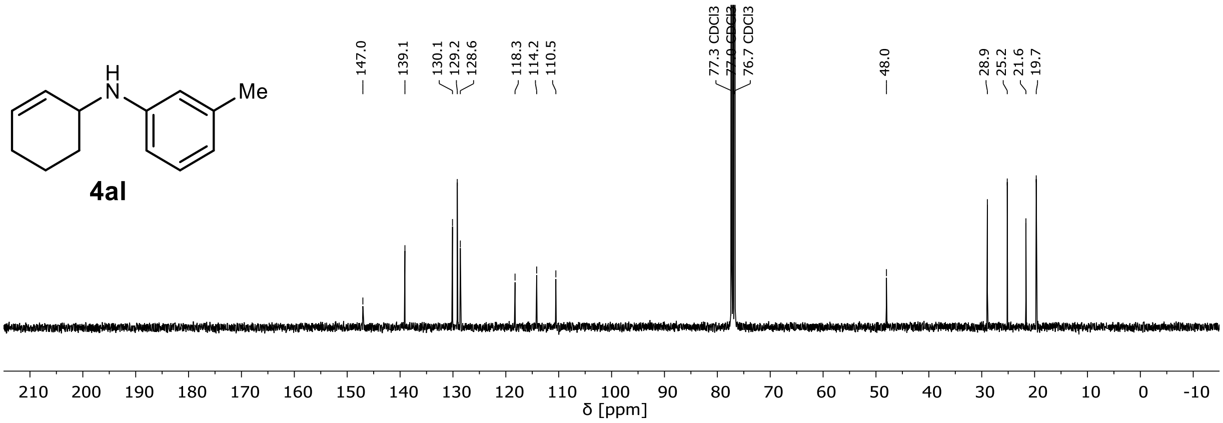

IR (ATR, neat) of **4al**

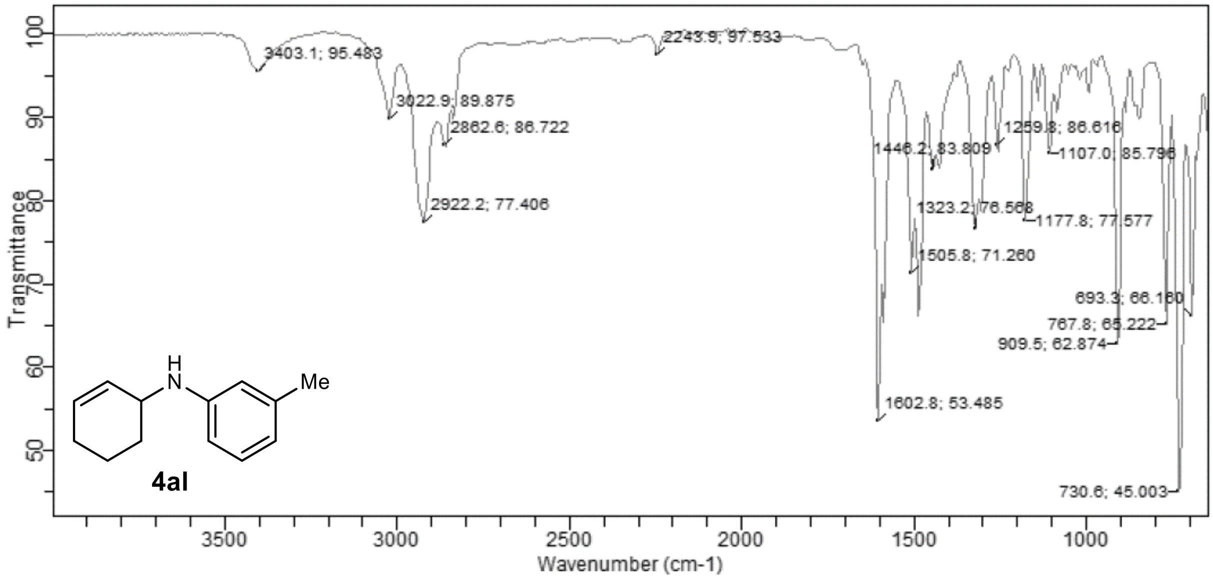

^{1}H NMR (400 MHz, $CDCl_3$) of **4am**

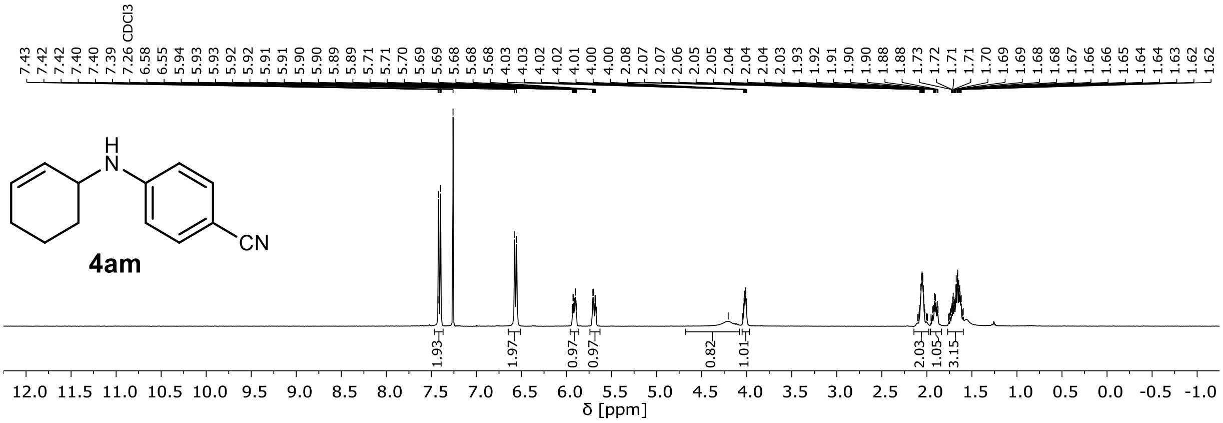

^{13}C NMR (101 MHz, $CDCl_3$) of **4am**

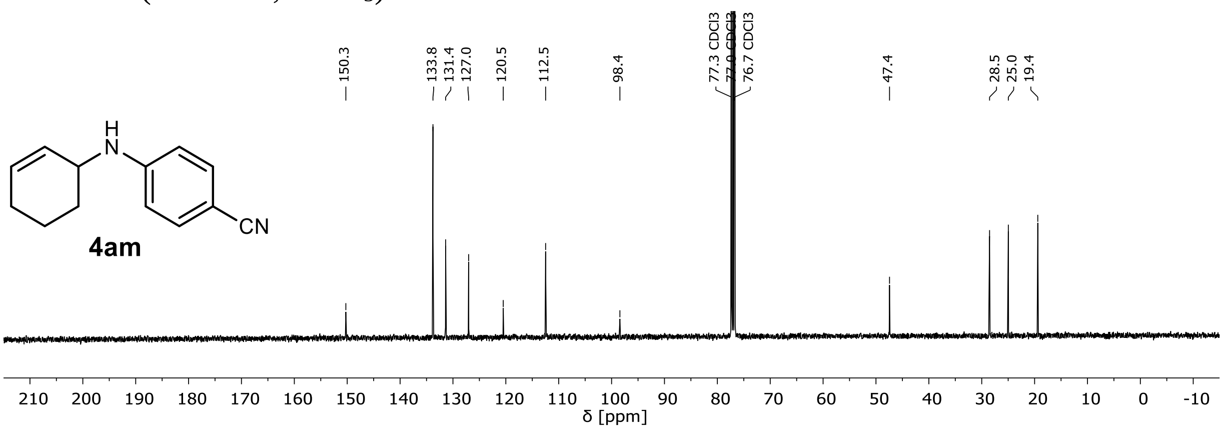

IR (ATR, neat) of **4am**

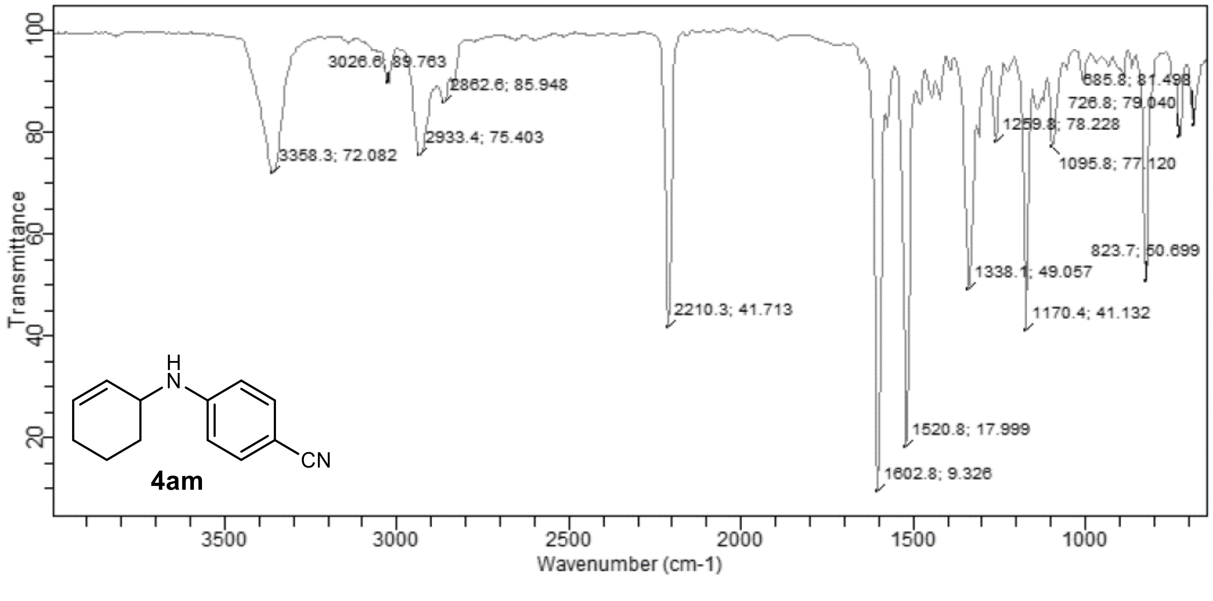

^{1}H NMR (400 MHz, $CDCl_3$) of **4an**

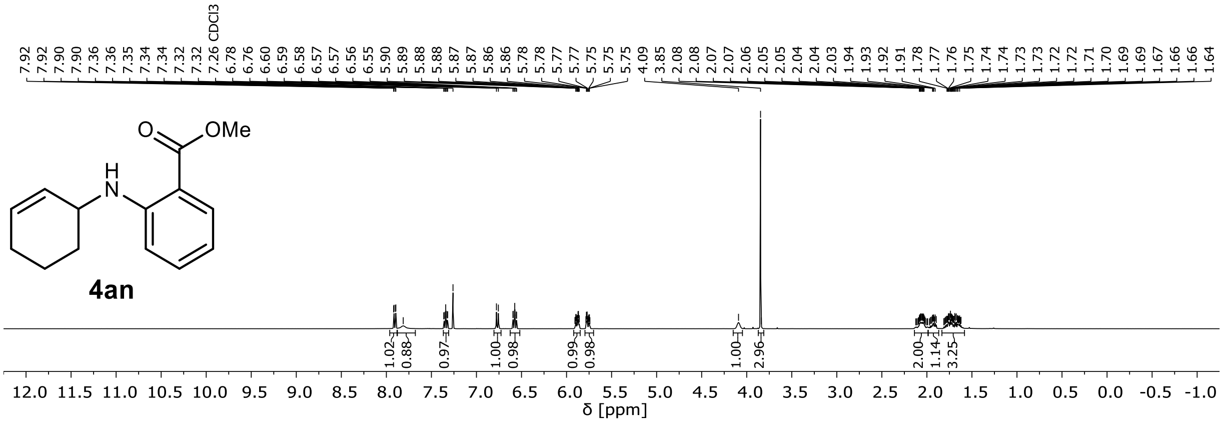

^{13}C NMR (101 MHz, $CDCl_3$) of **4an**

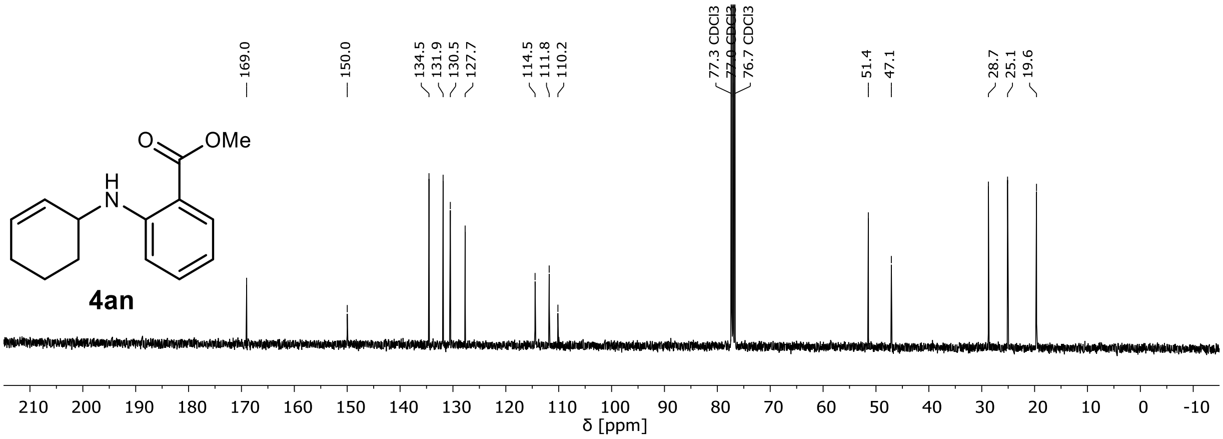

IR (ATR, neat) of **4an**

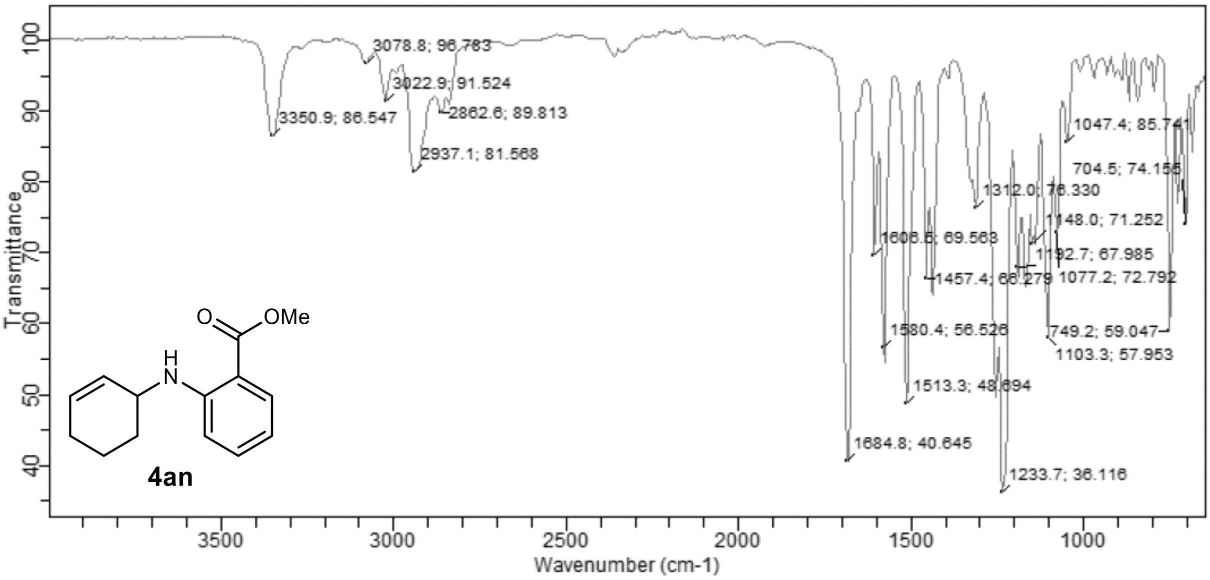

^{1}H NMR (400 MHz, $CDCl_3$) of **4ao**

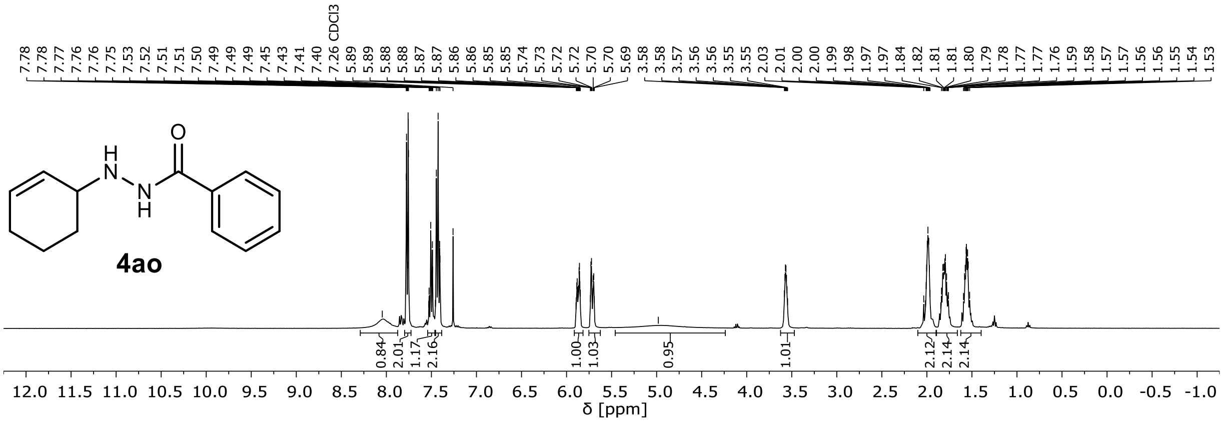

^{13}C NMR (101 MHz, $CDCl_3$) of **4ao**

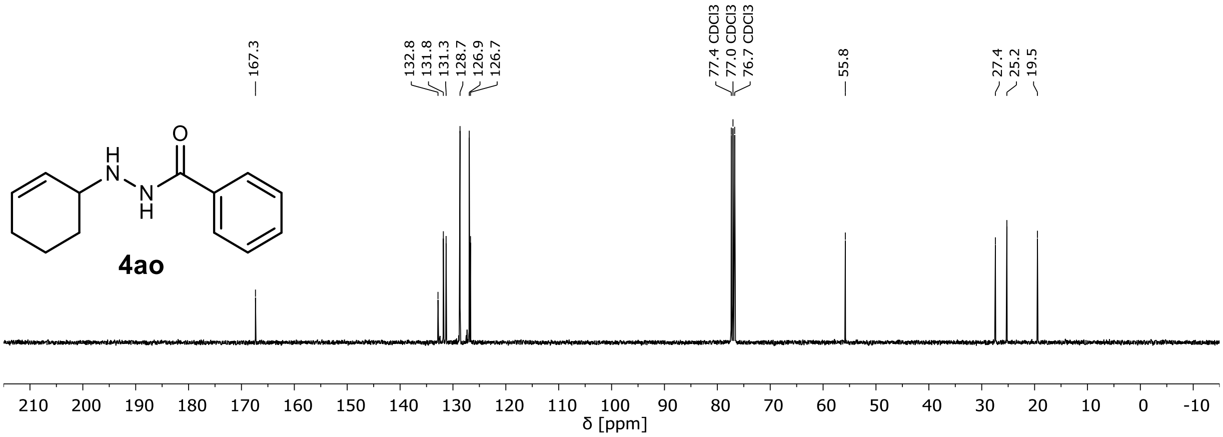

IR (ATR, neat) of **4ao**

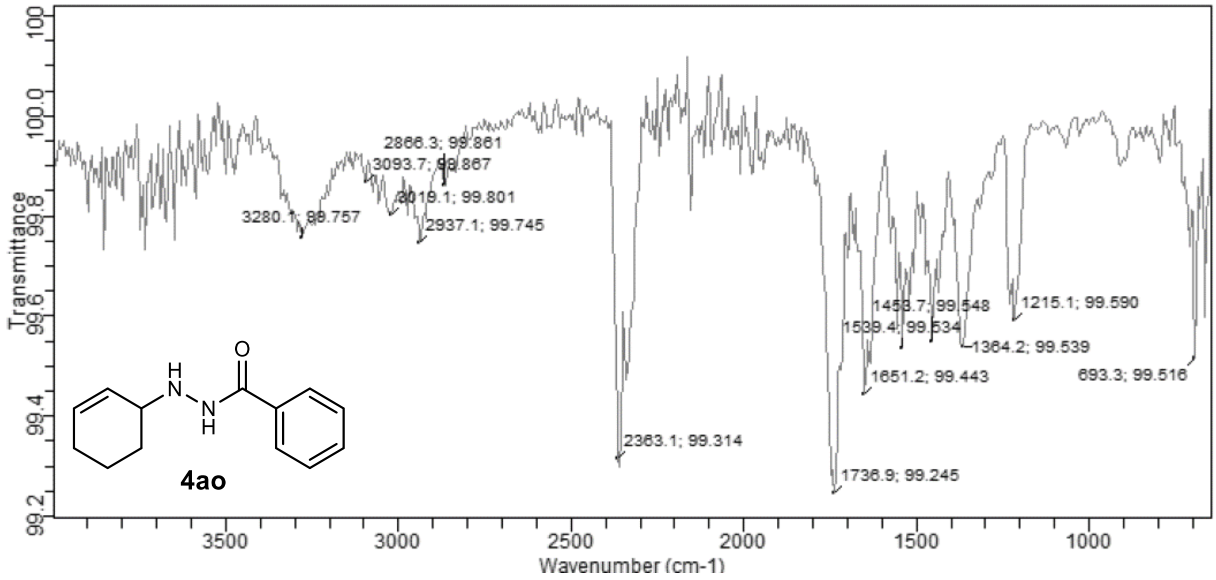

^{1}H NMR (400 MHz, CDCl$_3$) of **4ap**

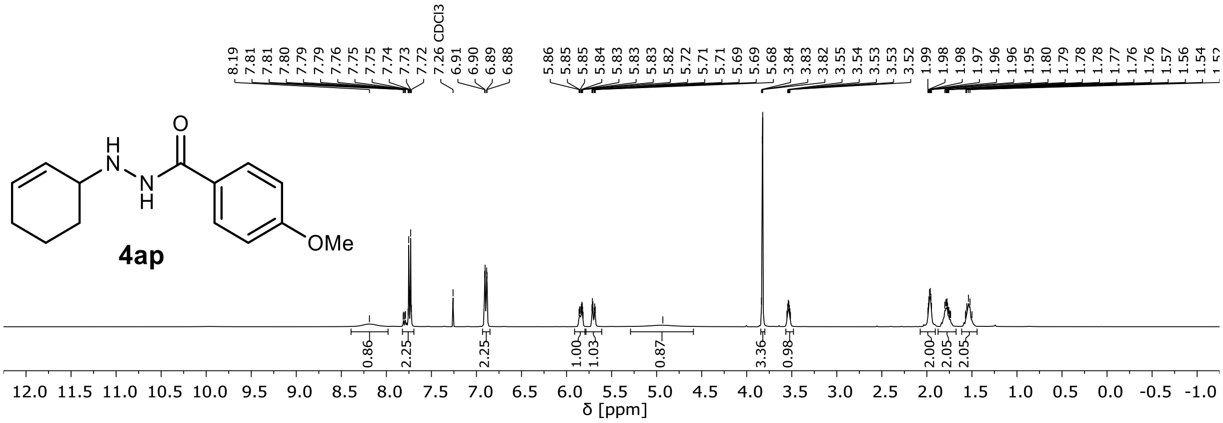

^{13}C NMR (101 MHz, CDCl$_3$) of **4ap**

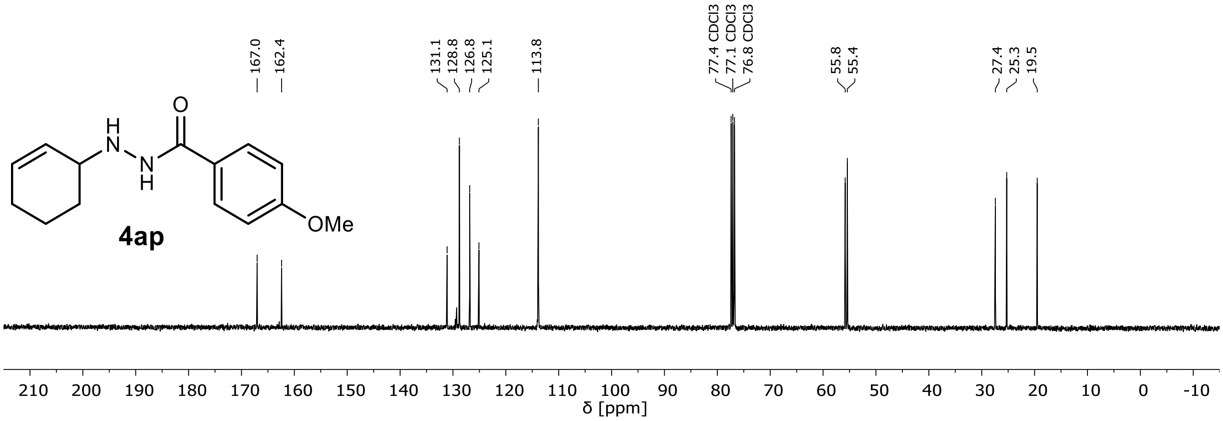

IR (ATR, neat) of **4ap**

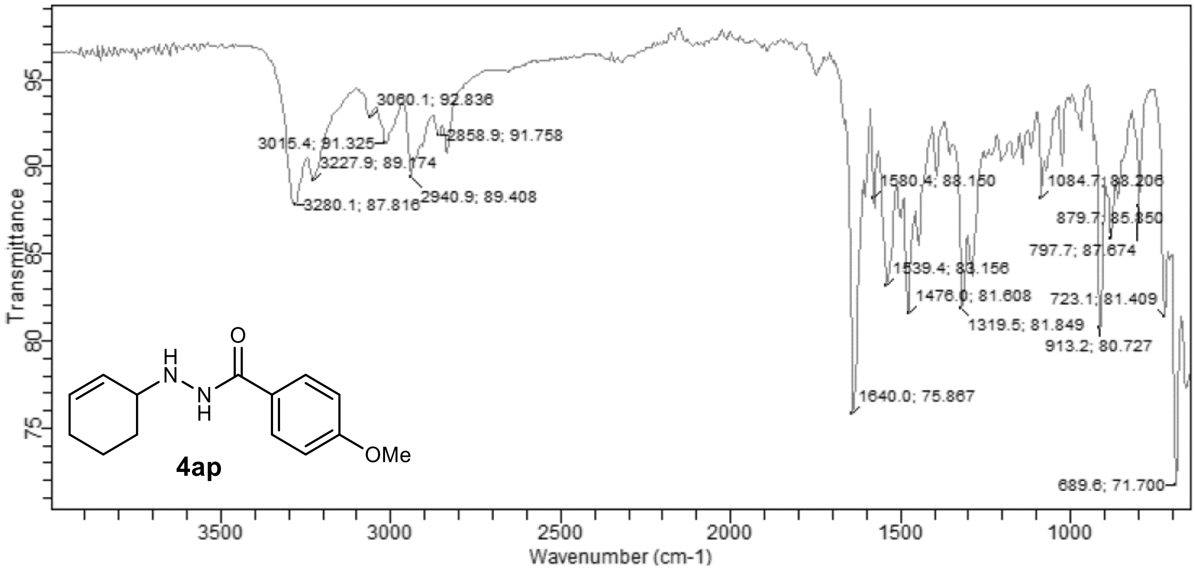

^{1}H NMR (400 MHz, $CDCl_3$) of **4aq**

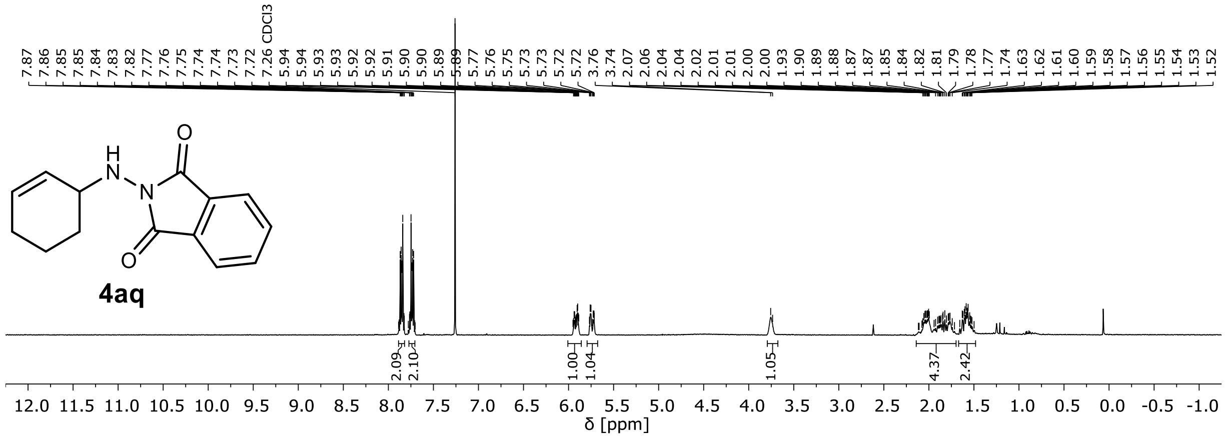

^{13}C NMR (101 MHz, $CDCl_3$) of **4aq**

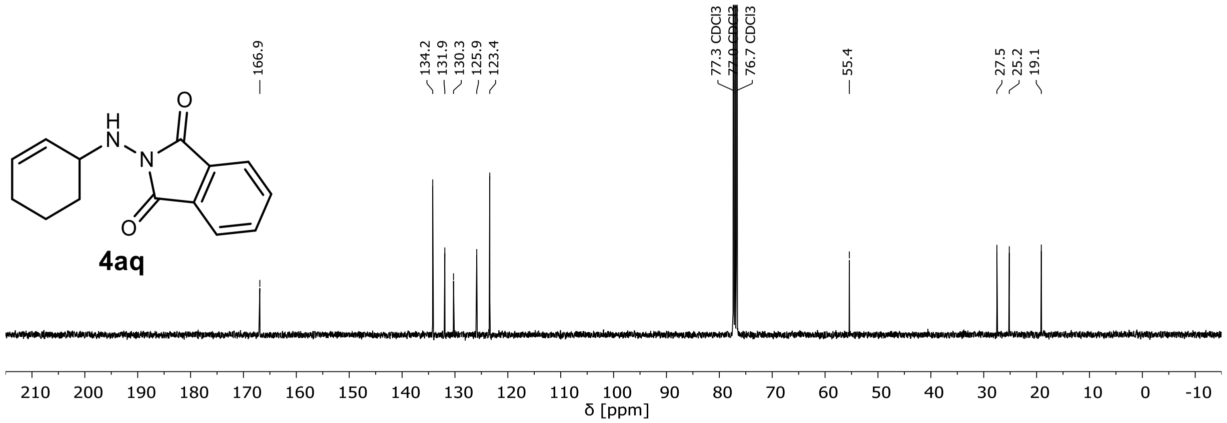

IR (ATR, neat) of **4aq**

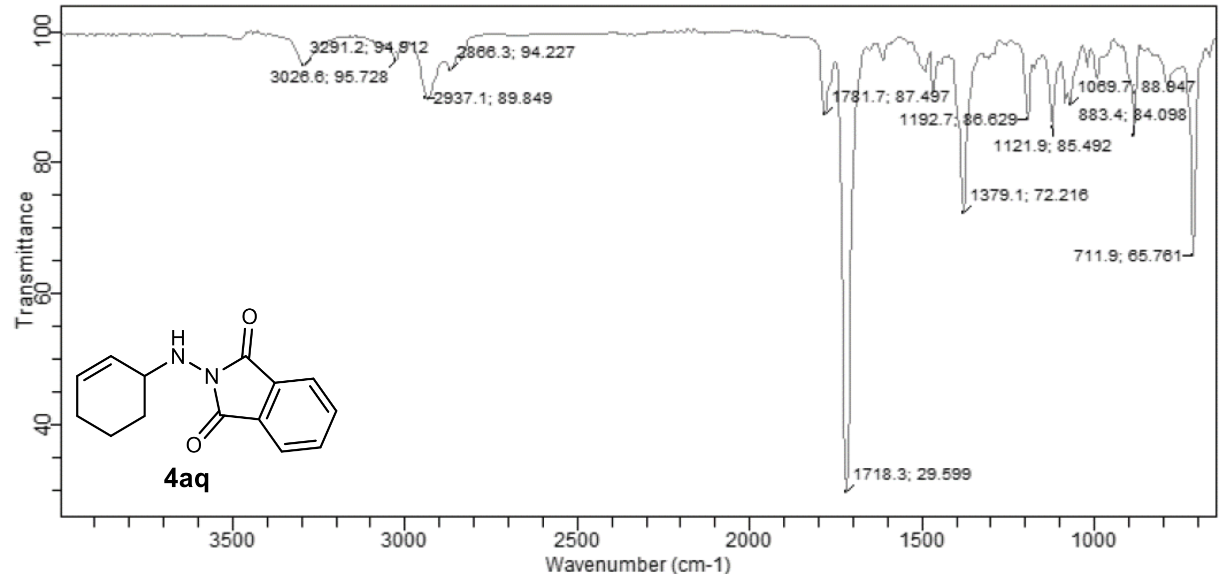

^{1}H NMR (400 MHz, CDCl3) of 4ar

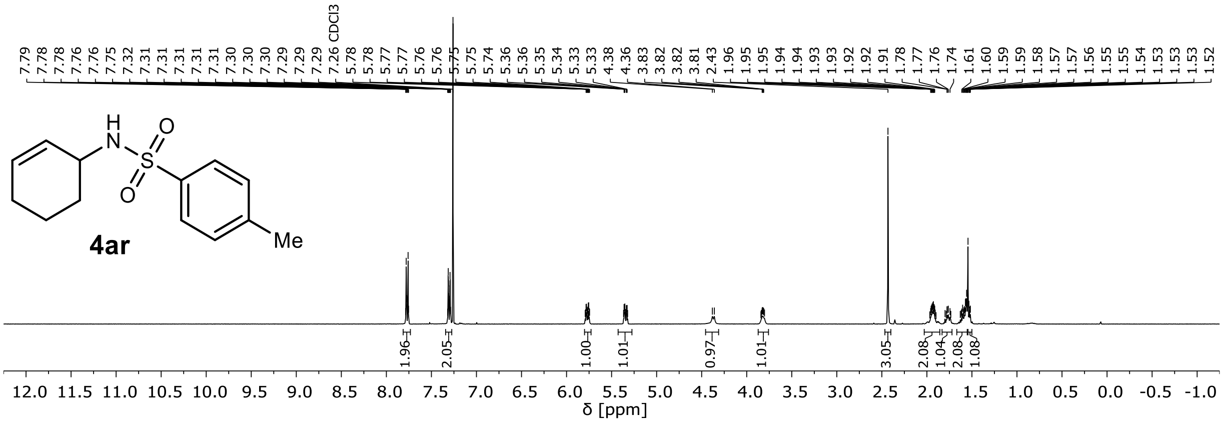

^{13}C NMR (101 MHz, CDCl3) of 4ar

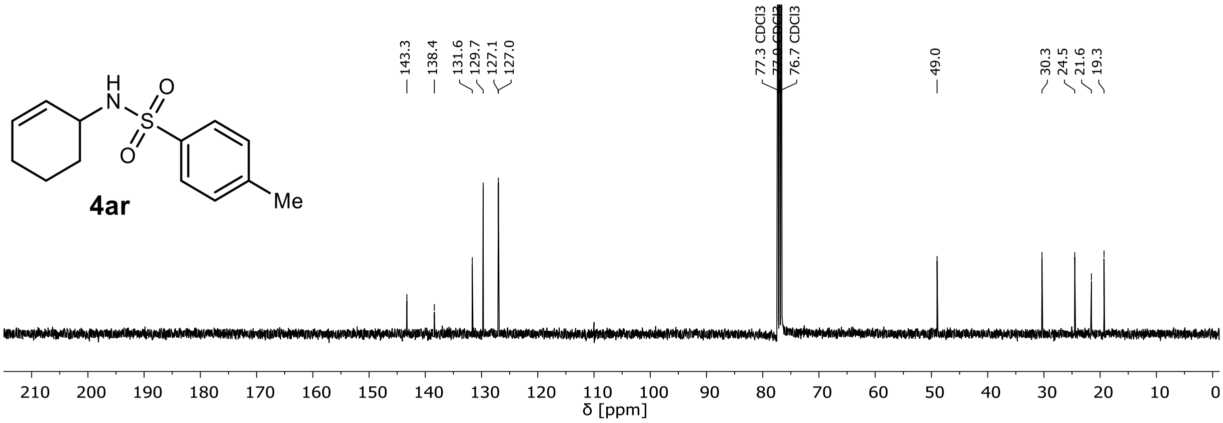

IR (ATR, neat) of 4ar

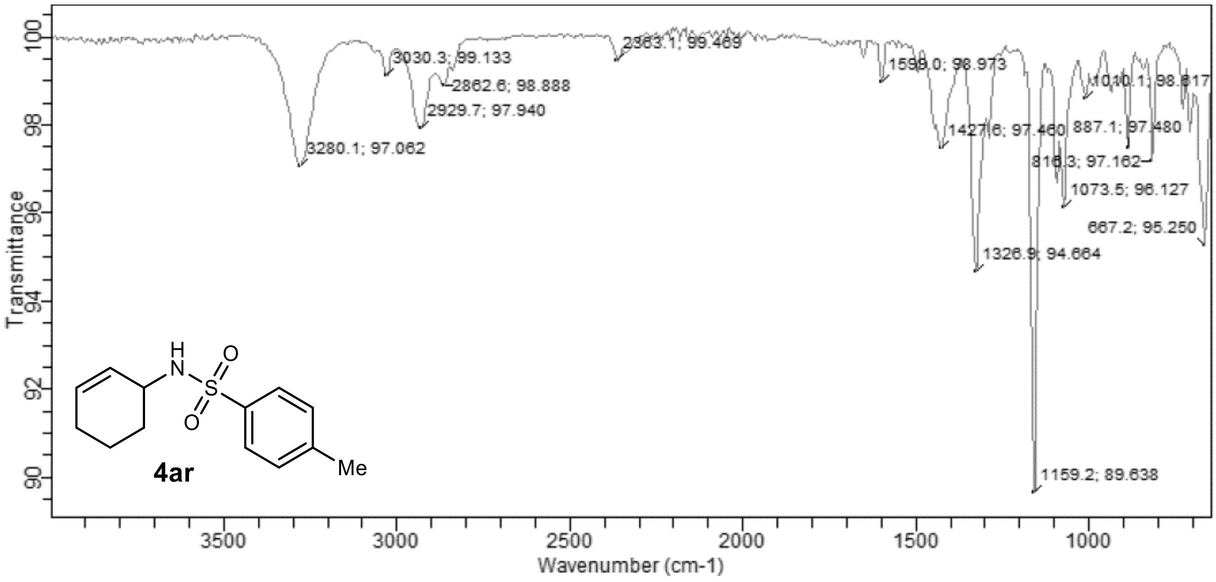

¹H NMR (400 MHz, CDCl3) of **4as**

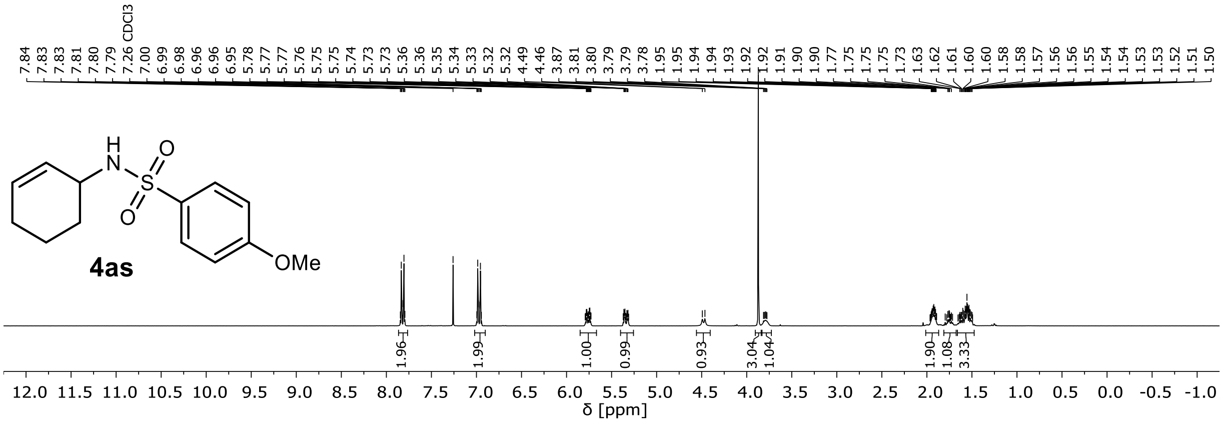

¹³C NMR (101 MHz, CDCl3) of **4as**

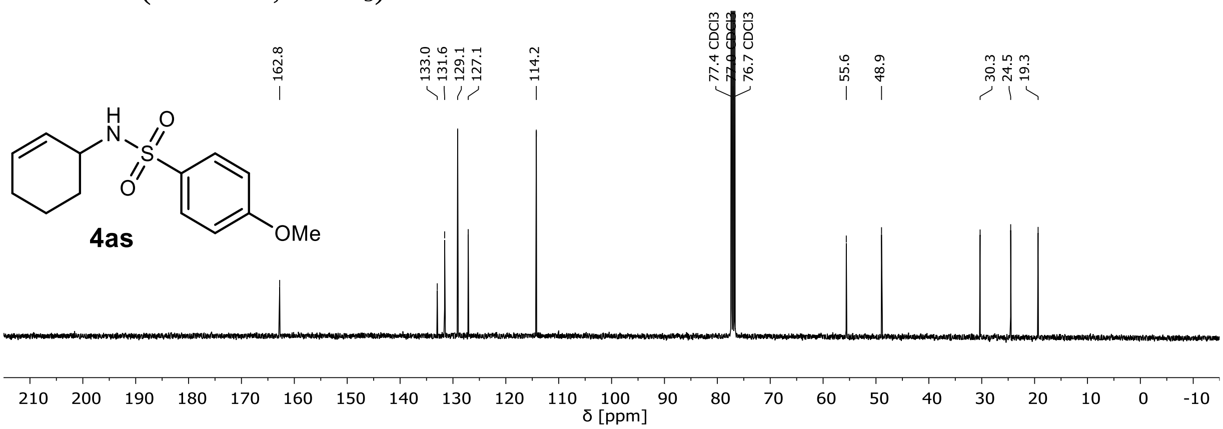

IR (ATR, neat) of **4as**

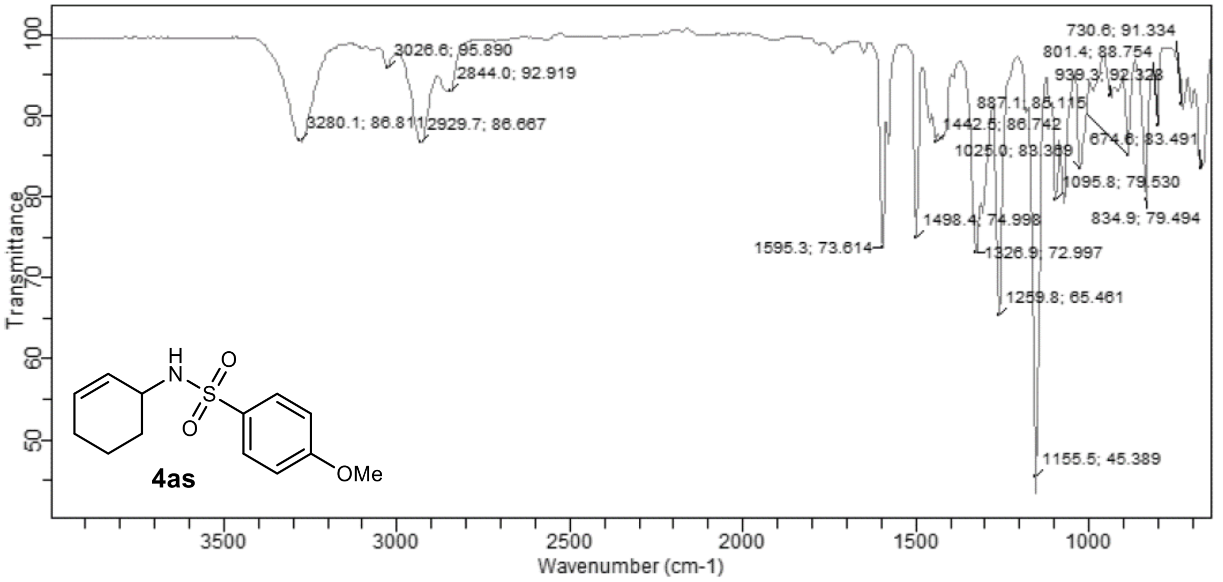

^{1}H NMR (300 MHz, $CDCl_3$) of **4at**

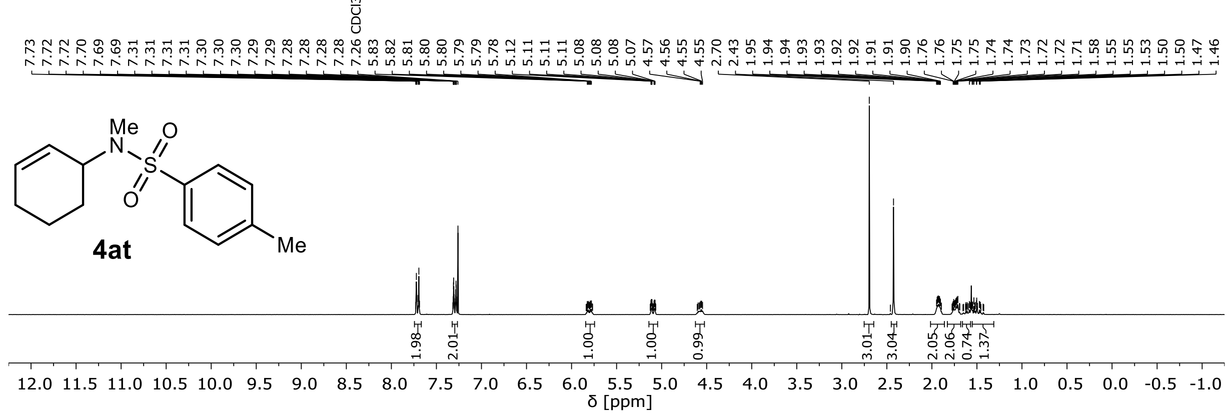

^{13}C NMR (101 MHz, $CDCl_3$) of **4at**

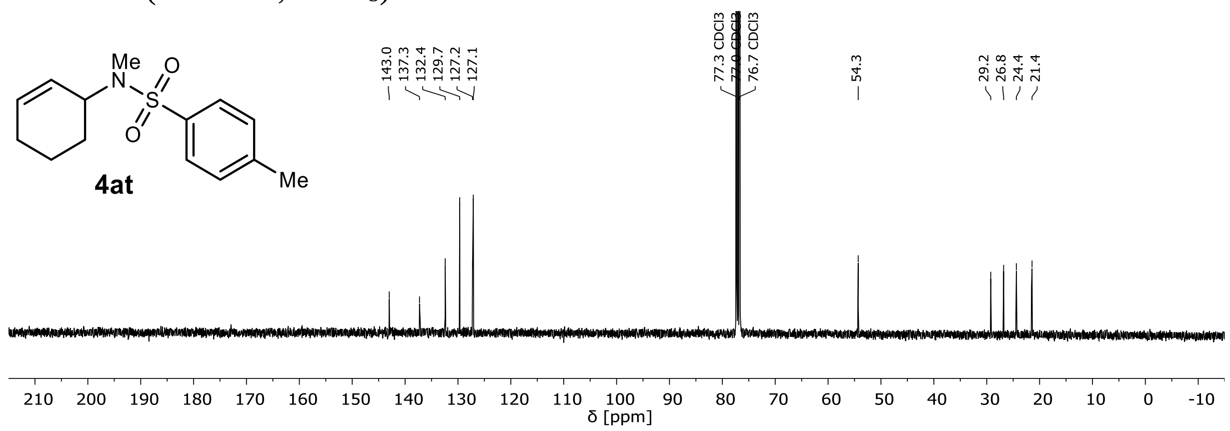

IR (ATR, neat) of **4at**

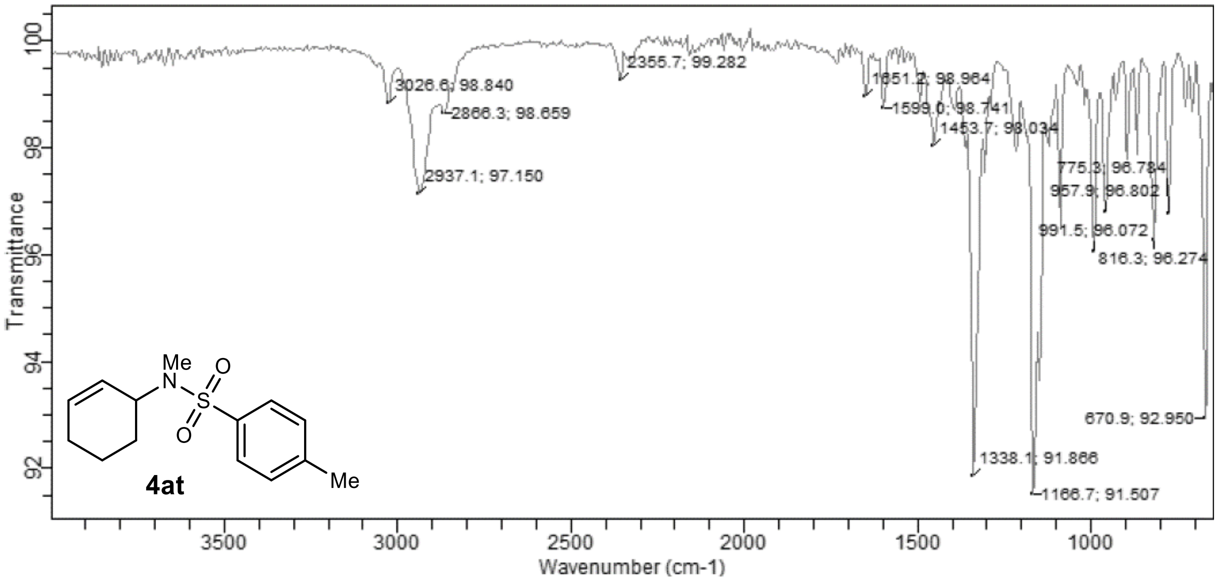

¹H NMR (400 MHz, CDCl₃) of **4au** (N¹ + N²)

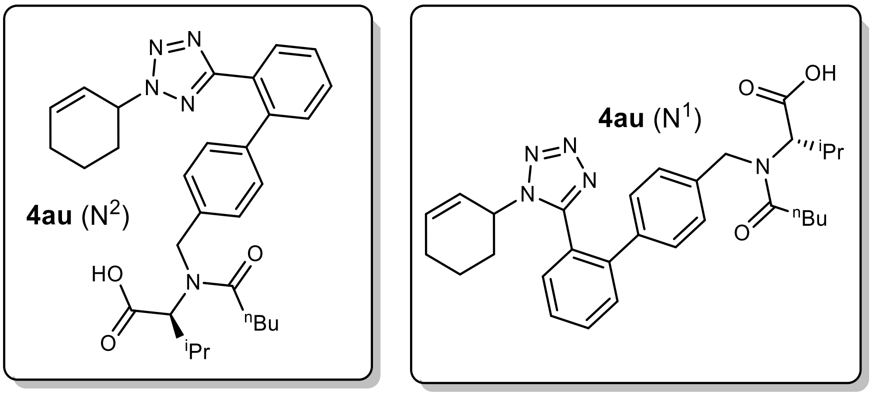

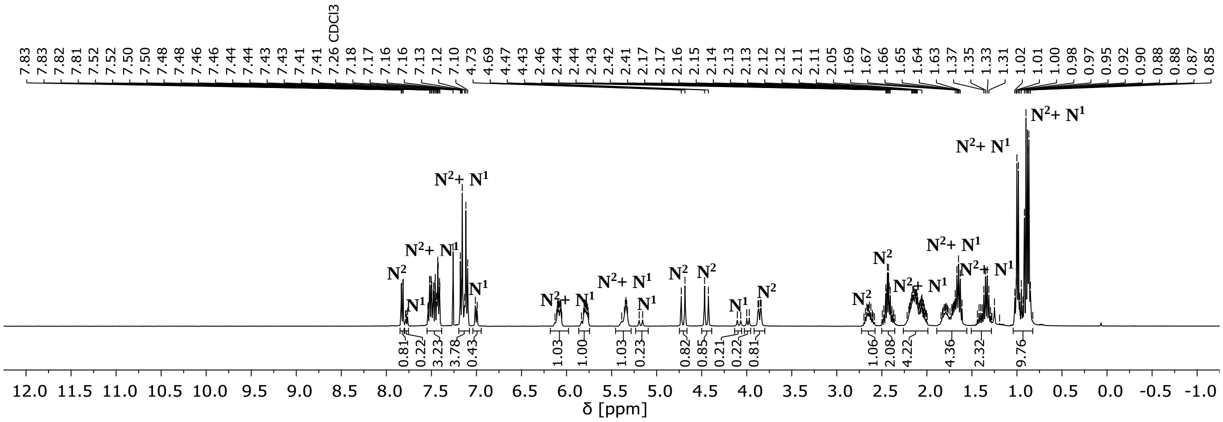

IR (ATR, neat) of **4au** (N¹ + N²)

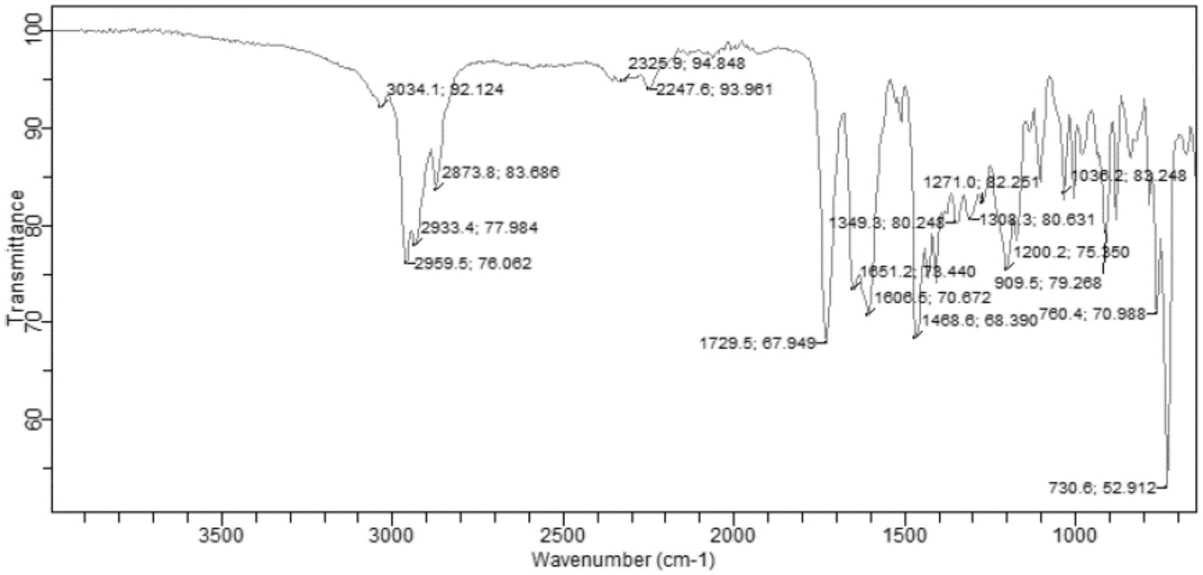

^{13}C NMR (101 MHz, $CDCl_3$) of **4au** (N^1 + N^2)

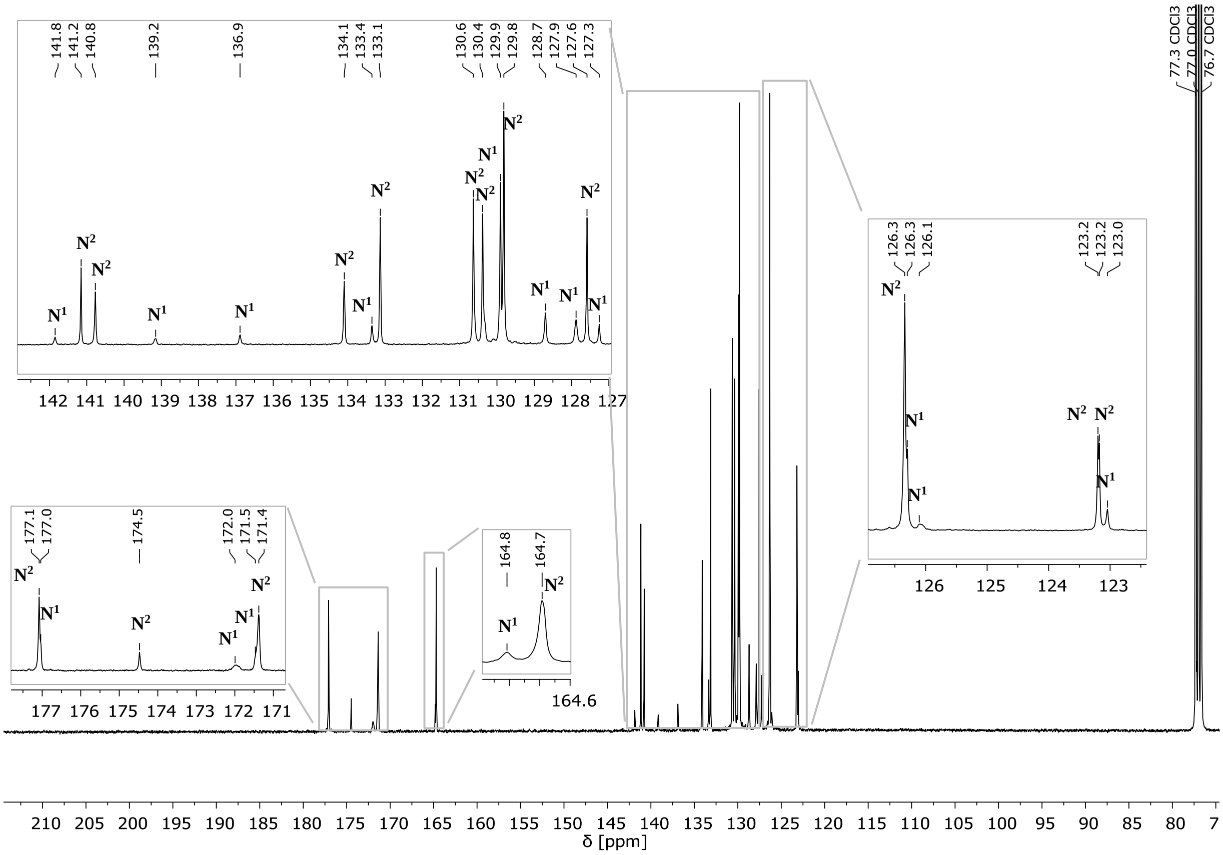

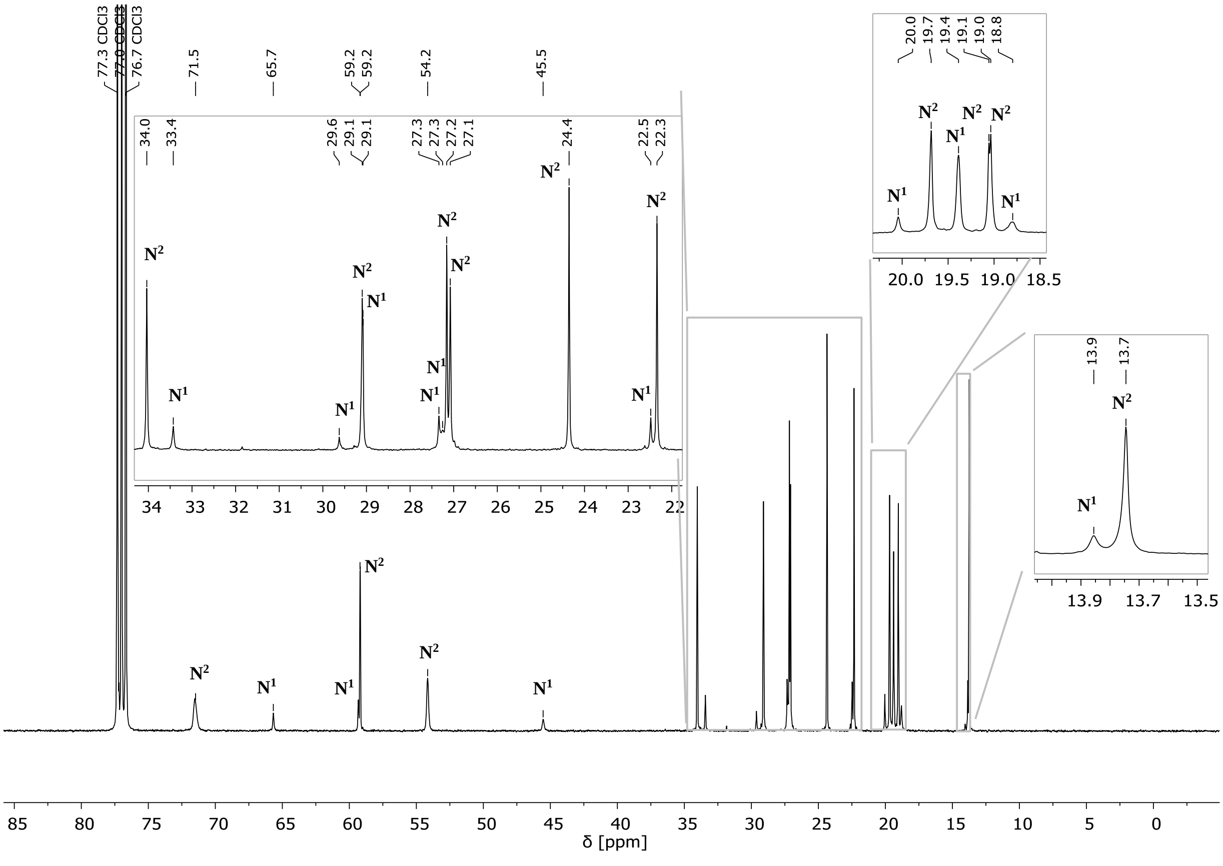

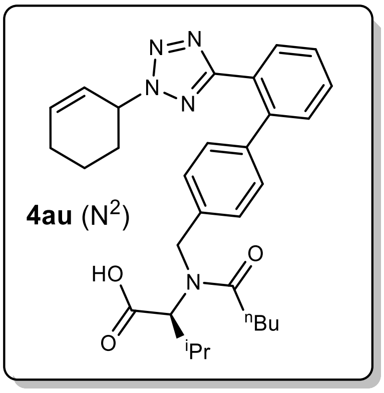
4au (N2)
HO
O
N
O
nBu
iPr

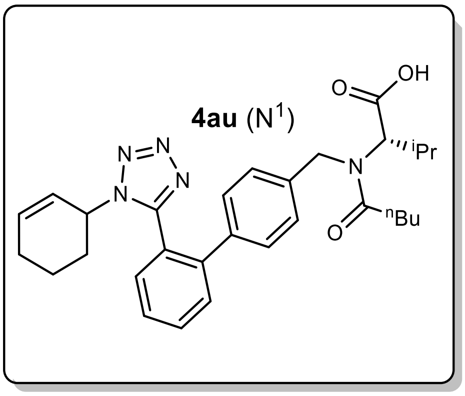
4au (N1)
OH
O
N
iPr
O
nBu

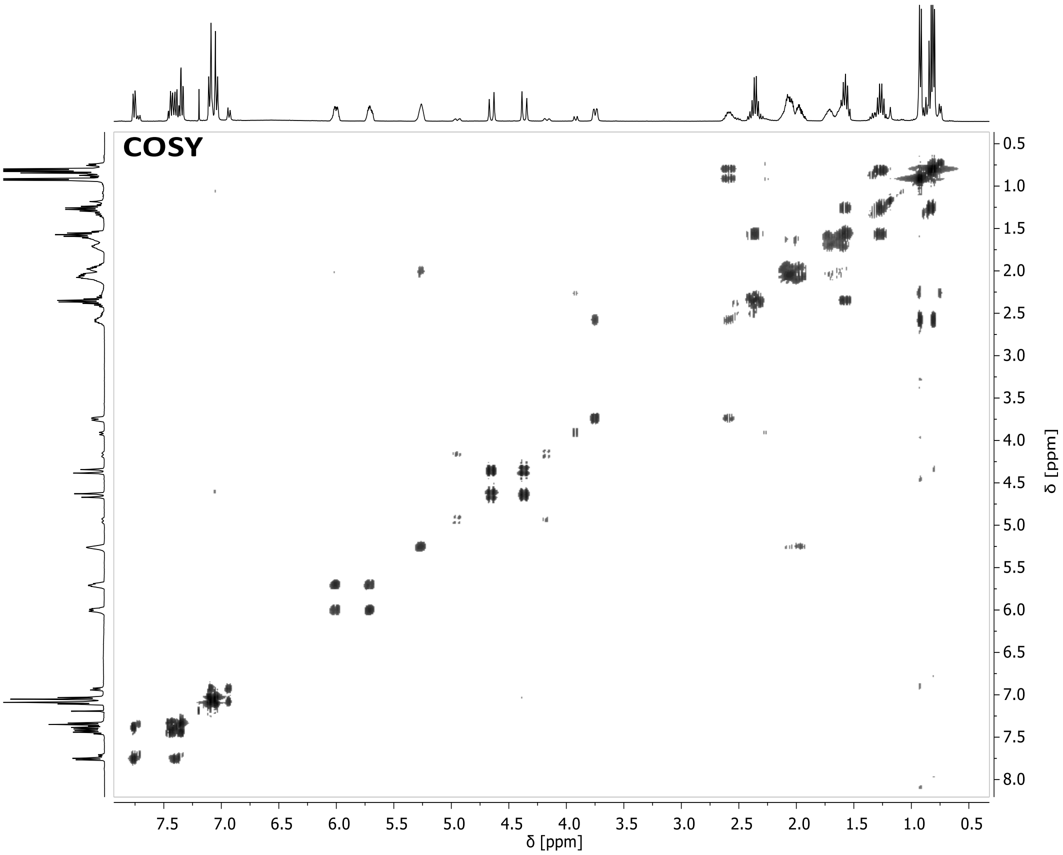
COSY
δ [ppm]
δ [ppm]

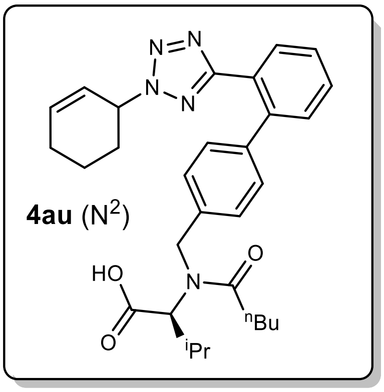
4au (N2)
HO
O
N
O
nBu
iPr

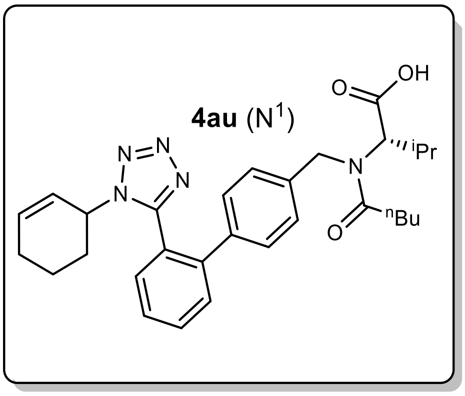
4au (N1)
O
OH
N
iPr
O
nBu

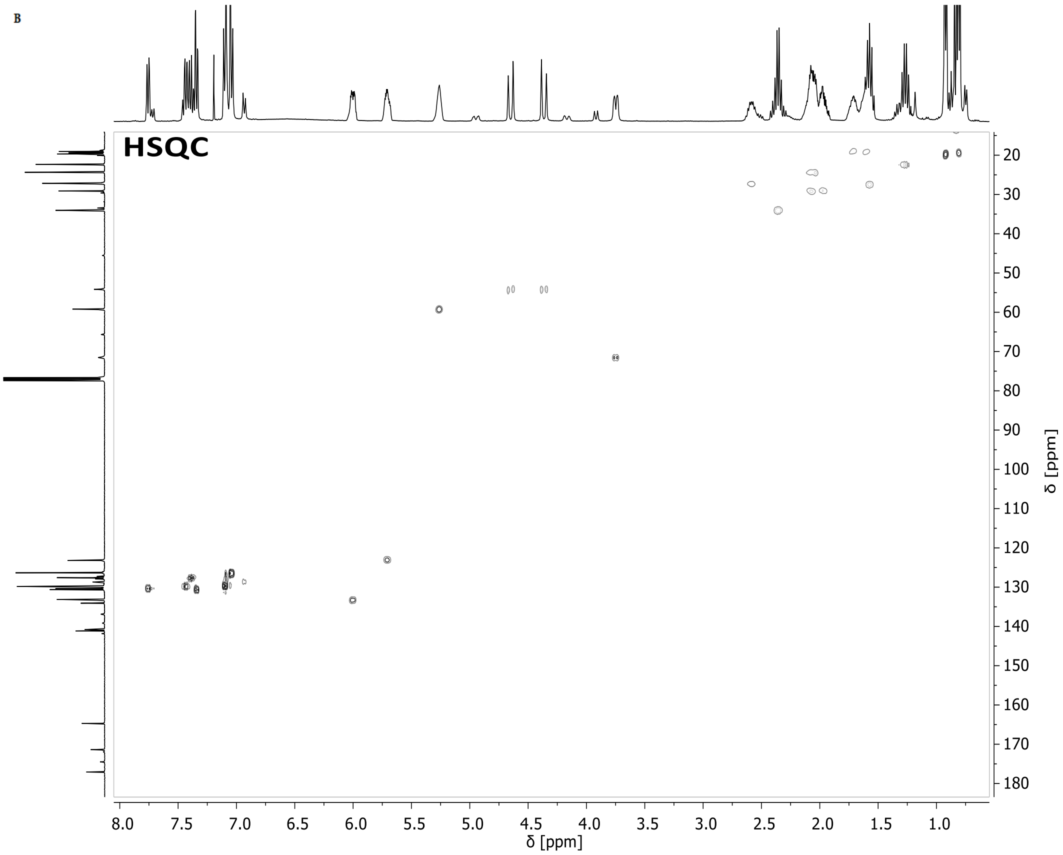
HSQC
δ [ppm]
δ [ppm]

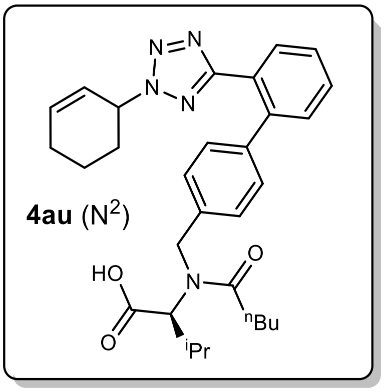
4au (N2)
HO
O
N
O
nBu
iPr
N=N
N
N

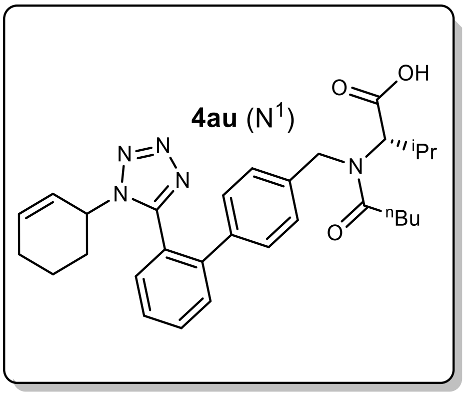
4au (N1)
O
OH
iPr
N
O
nBu
N=N
N
N

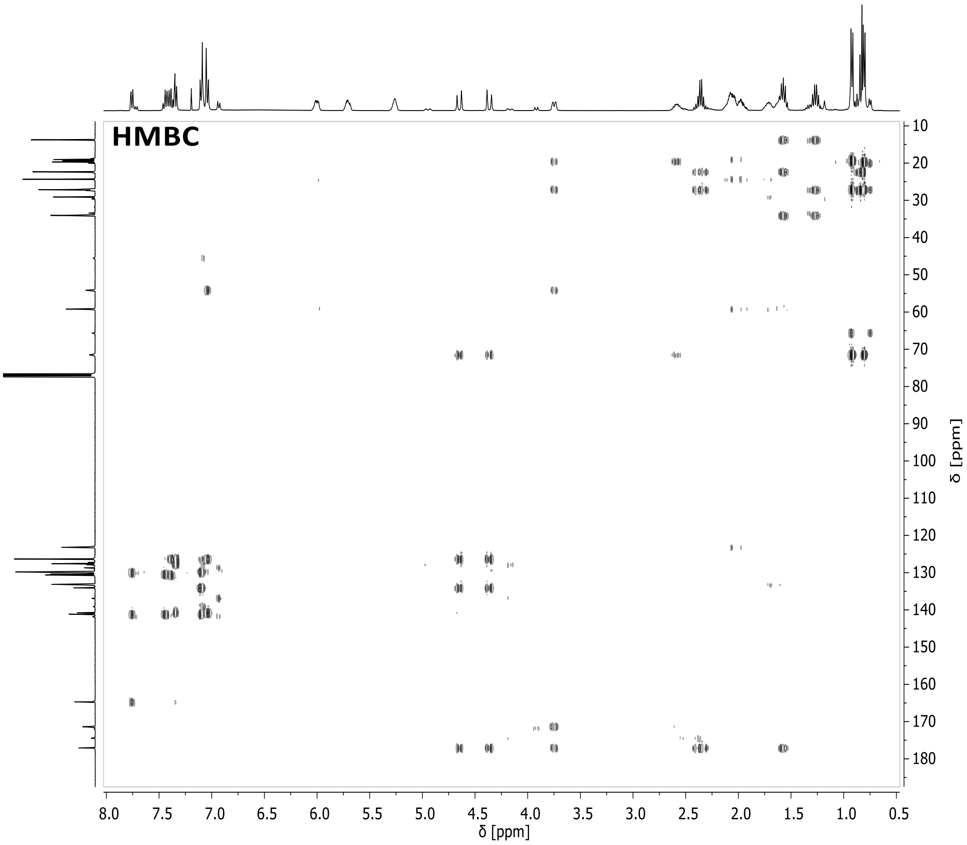
HMBC
δ [ppm]
δ [ppm]

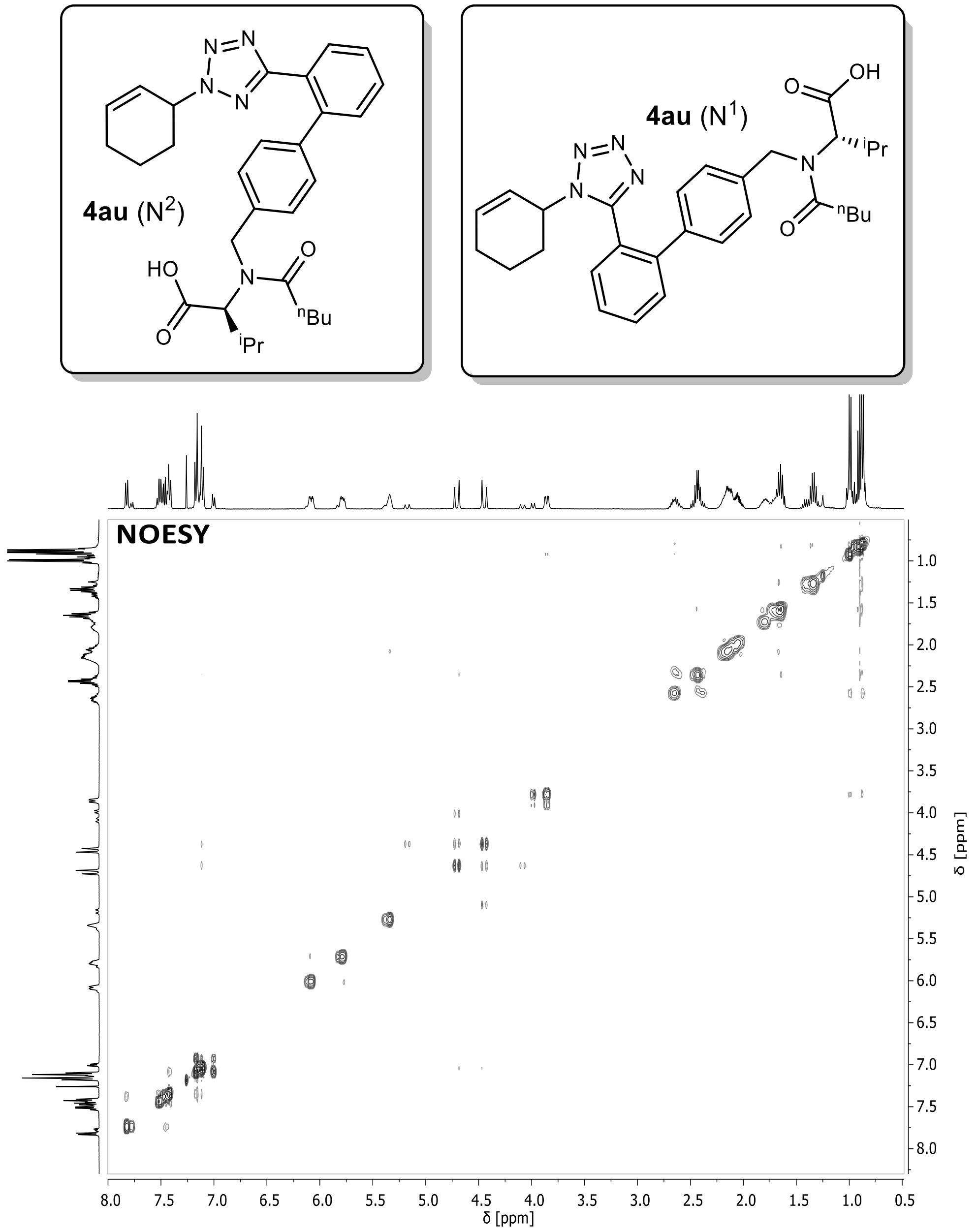

4au (N2)
4au (N1)
NOESY
δ [ppm]
δ [ppm]

^{1}H NMR (400 MHz, $CDCl_3$) of **4av**

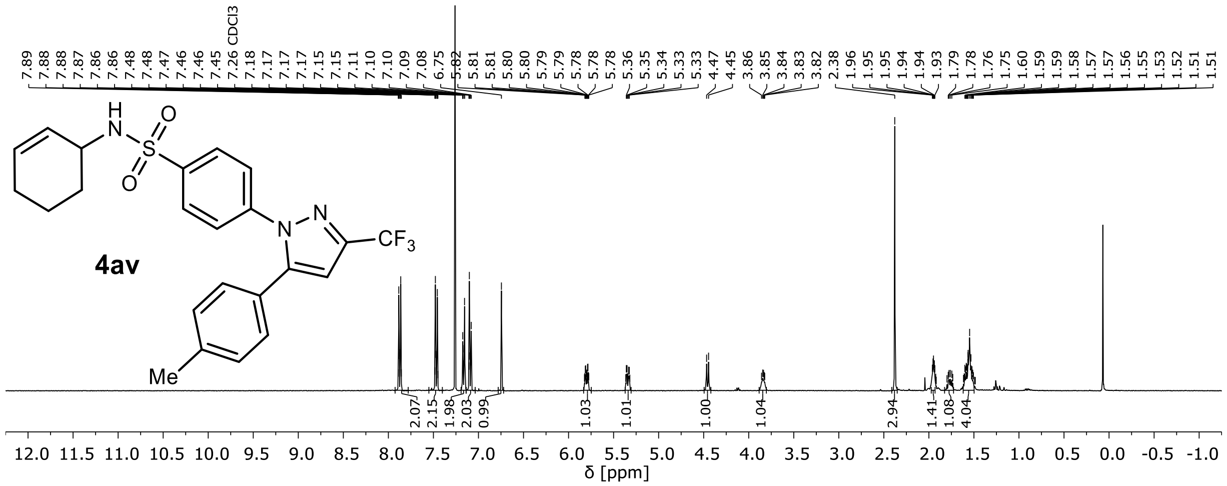

^{13}C NMR (101 MHz, $CDCl_3$) of **4av**

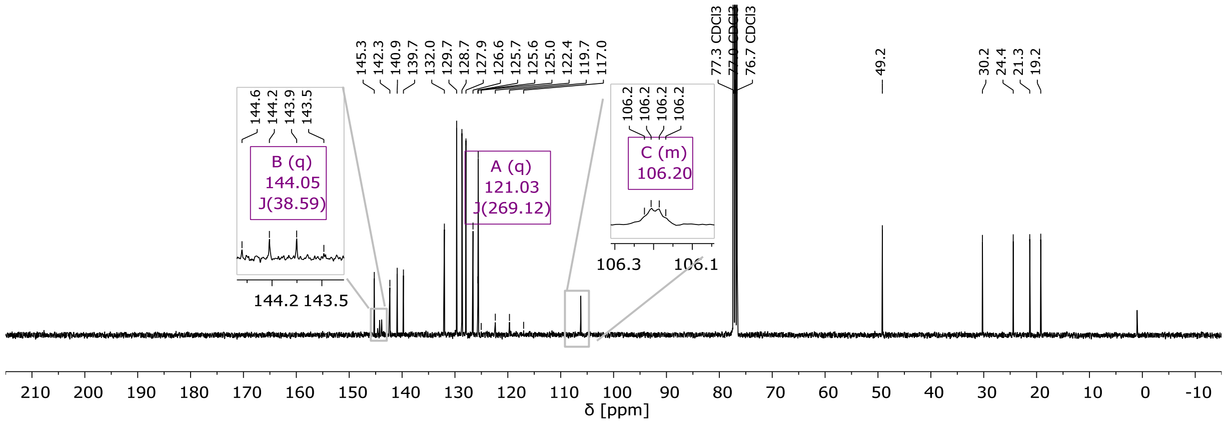

^{19}F NMR (377 MHz, $CDCl_3$) of **4av**

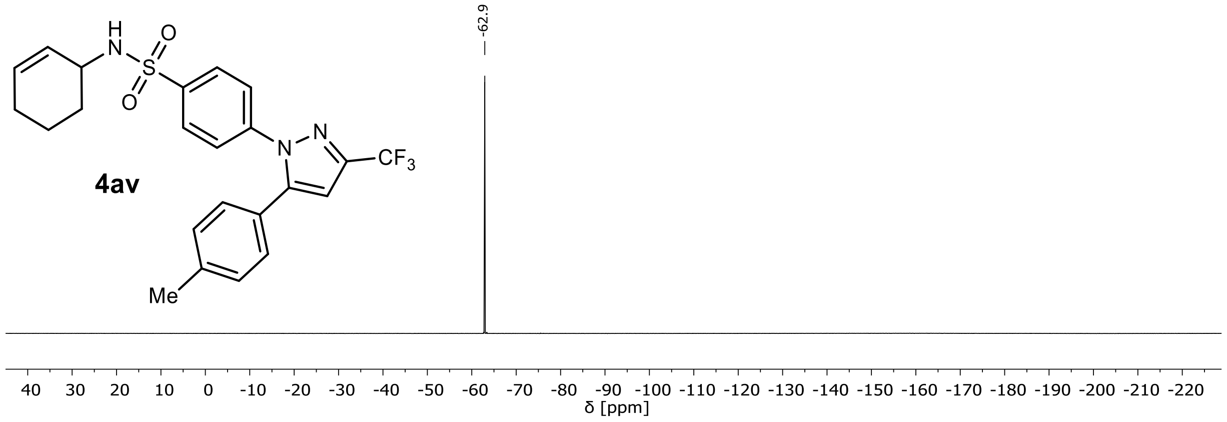

IR (ATR, neat) of **4av**

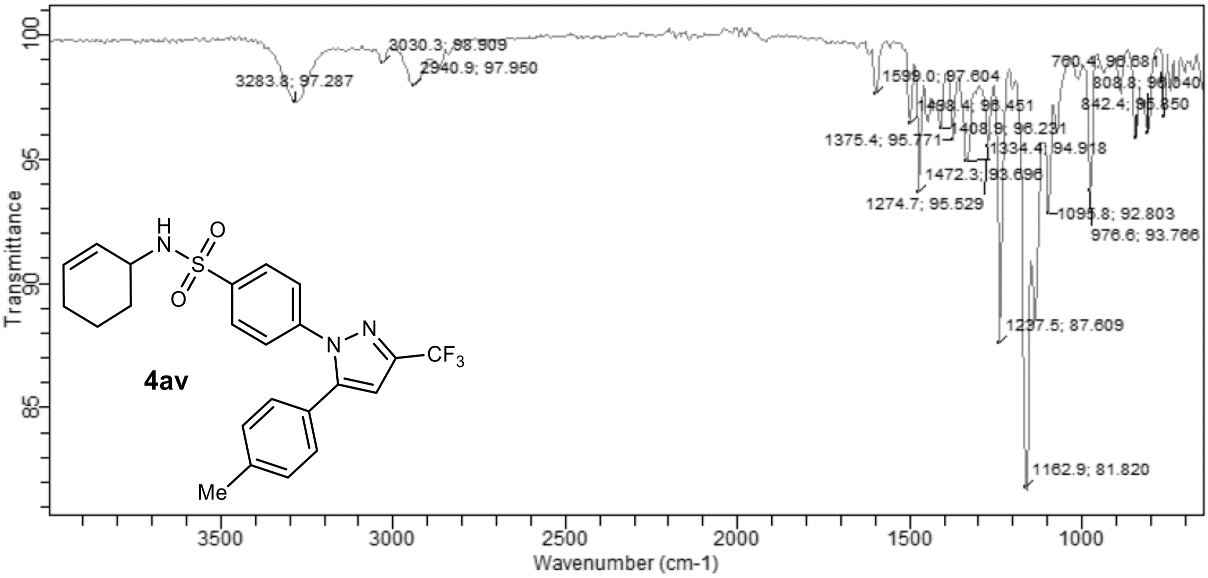

^{1}H NMR (400 MHz, $CDCl_3$) of **4br**

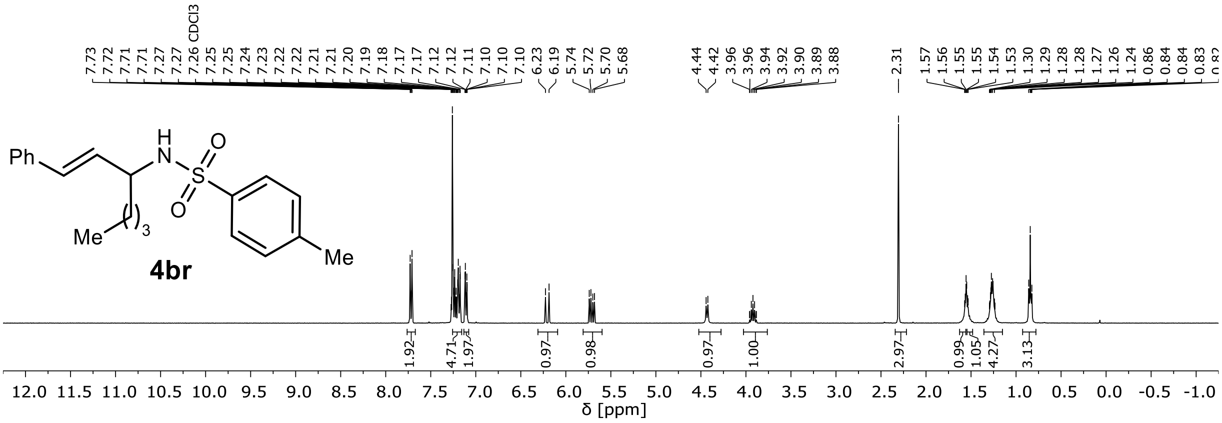

^{13}C NMR (101 MHz, $CDCl_3$) of **4br**

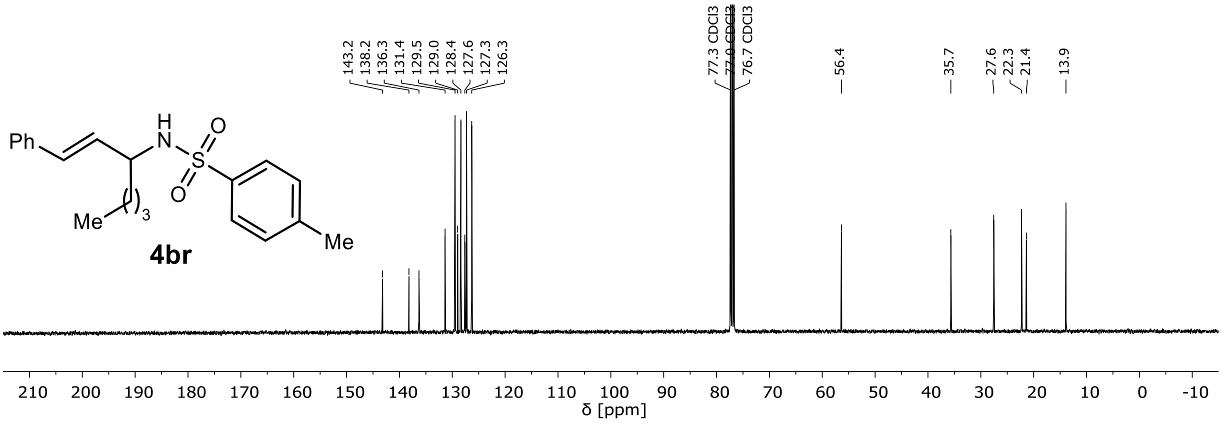

IR (ATR, neat) of **4br**

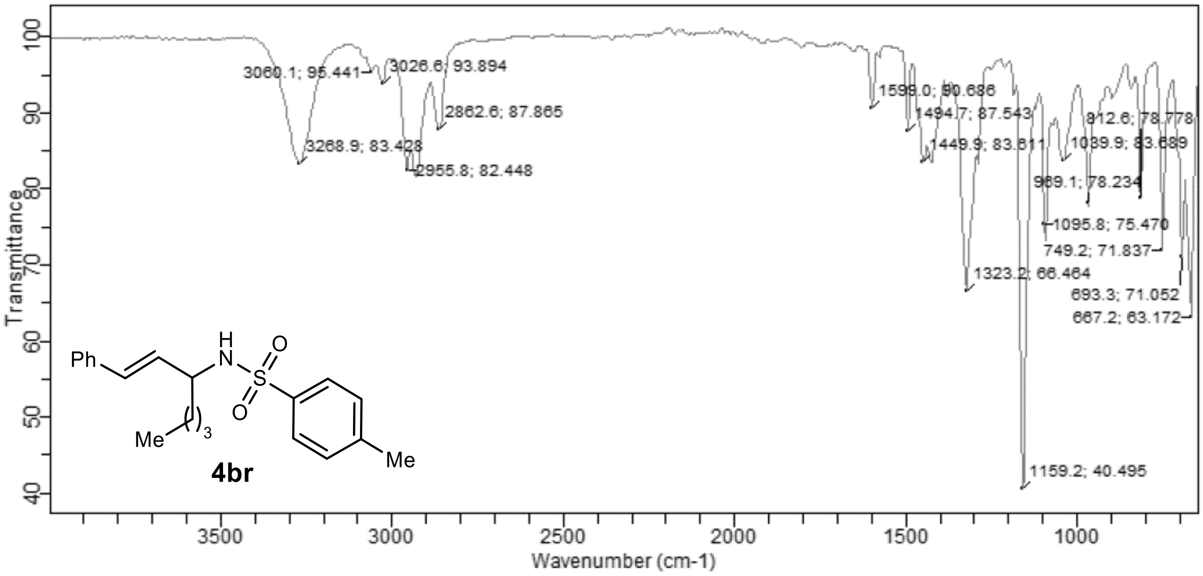

^{1}H NMR (300 MHz, $CDCl_3$) of **4cr**

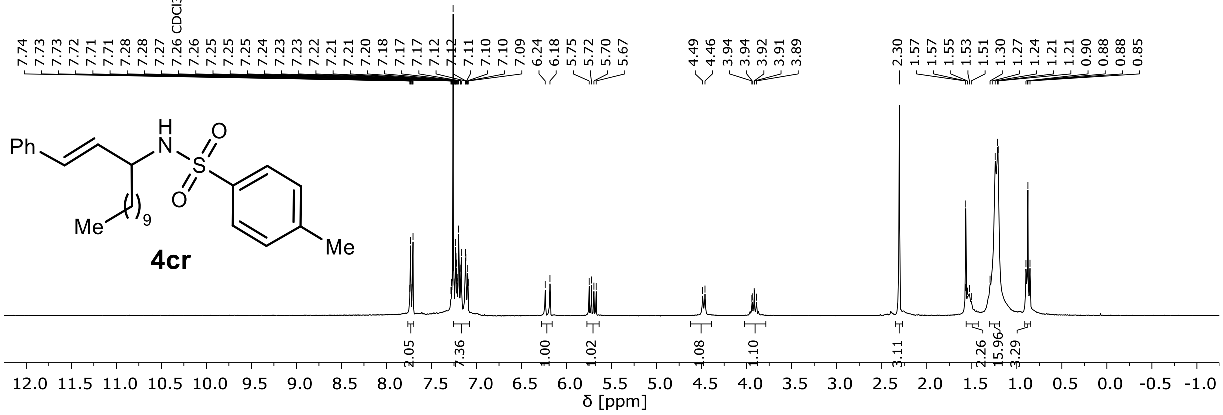

^{13}C NMR (101 MHz, $CDCl_3$) of **4cr**

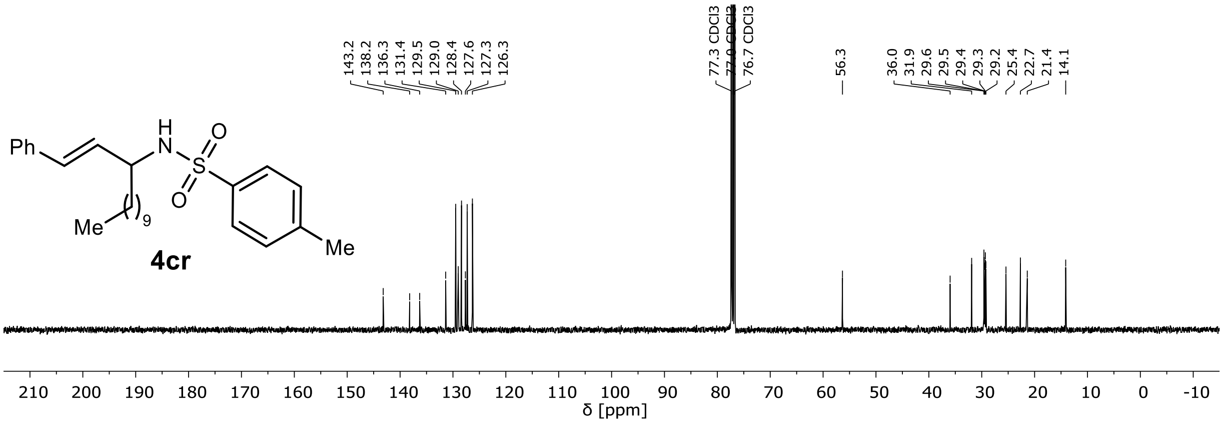

IR (ATR, neat) of **4cr**

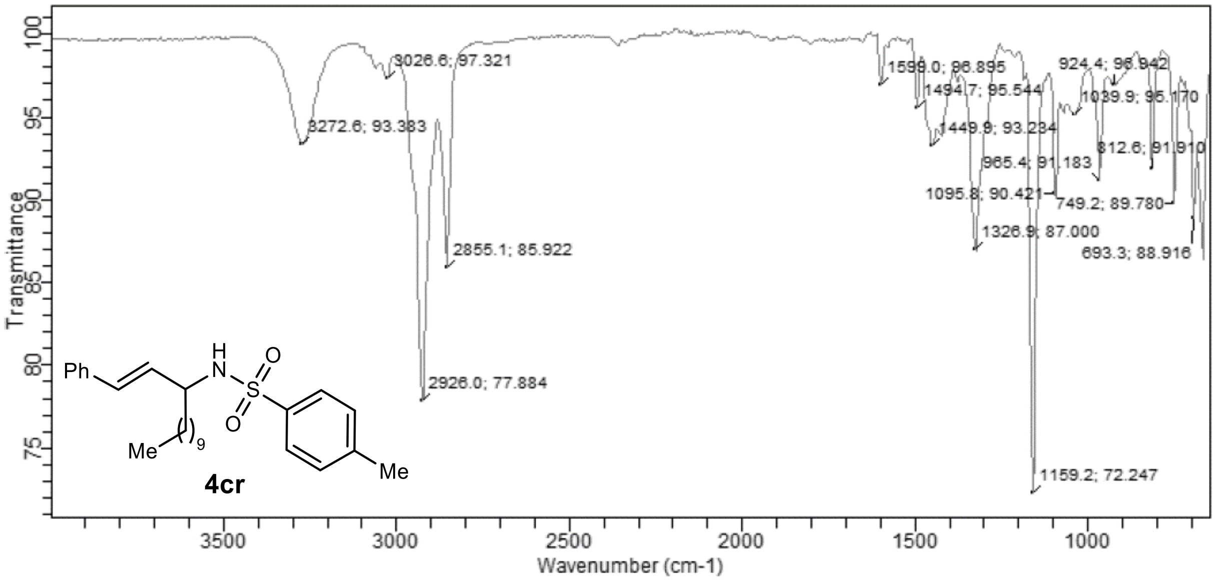

^{1}H NMR (400 MHz, $CDCl_3$) of **4dr**

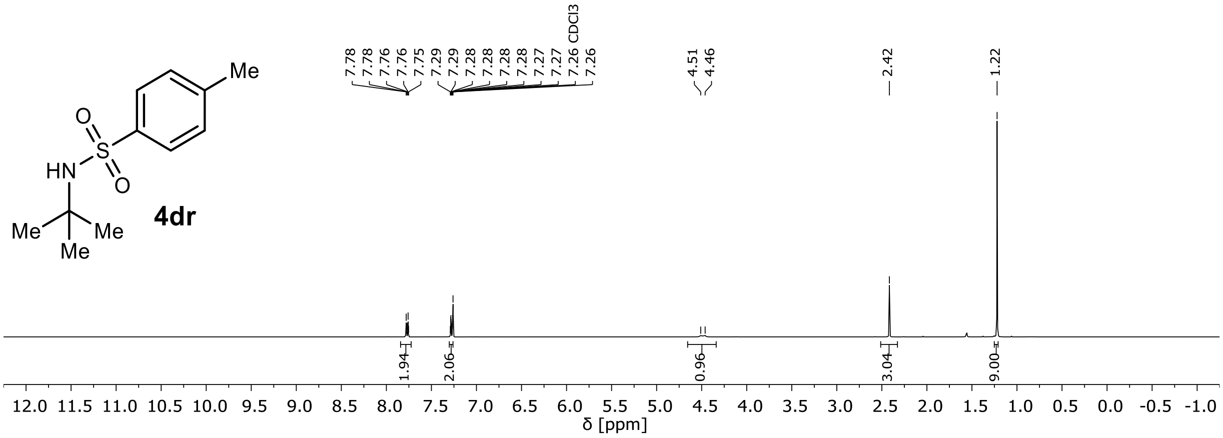

^{13}C NMR (101 MHz, $CDCl_3$) of **4dr**

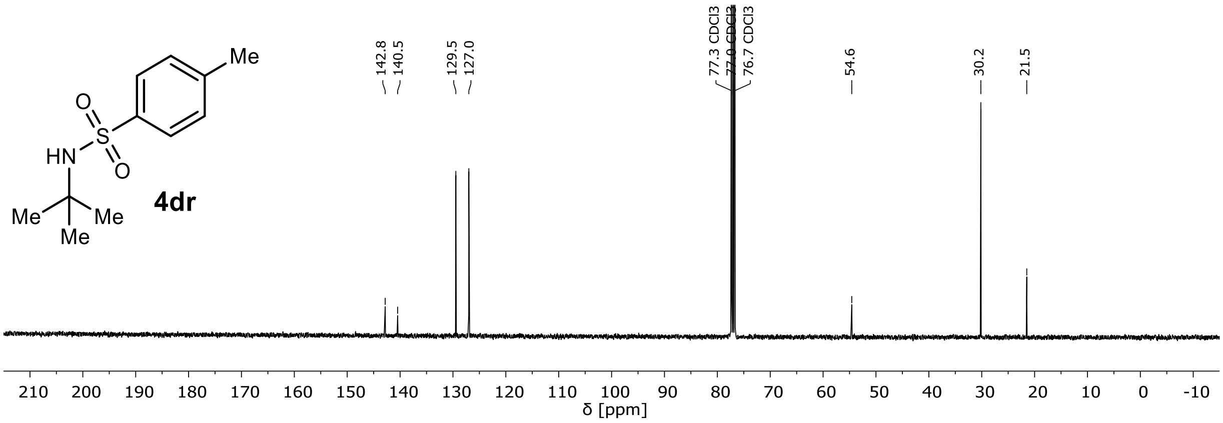

IR (ATR, neat) of **4dr**

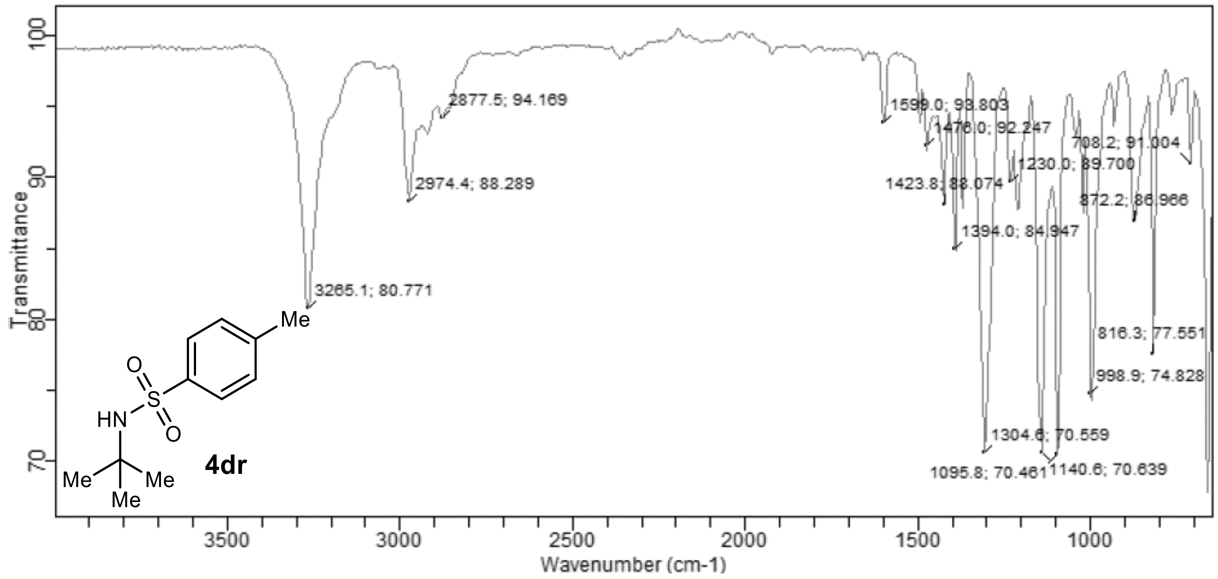

^{1}H NMR (400 MHz, $CDCl_3$) of **4er**

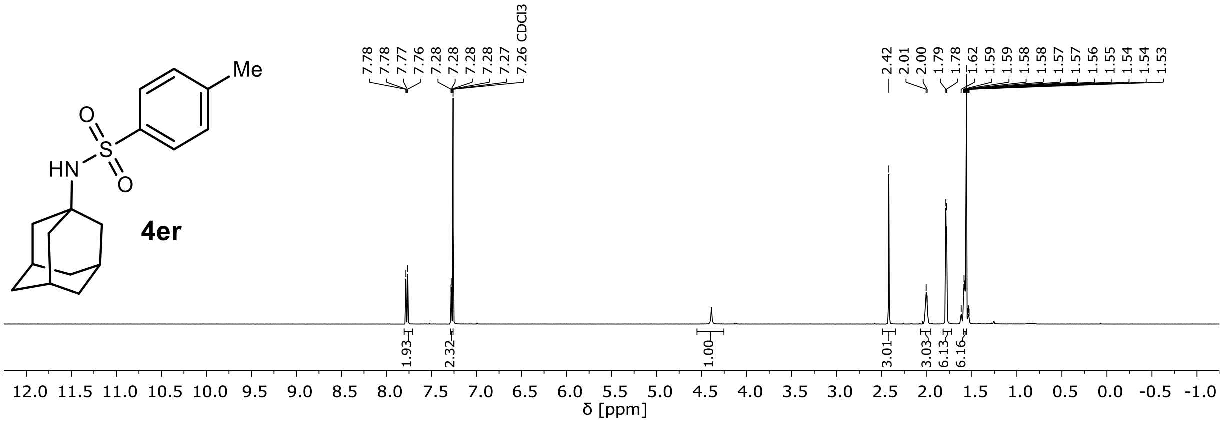

^{13}C NMR (101 MHz, $CDCl_3$) of **4er**

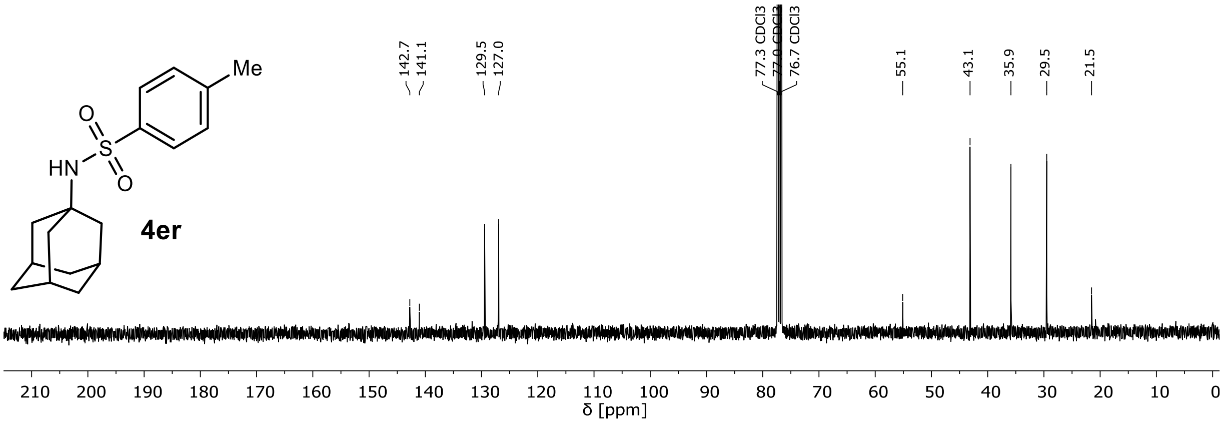

IR (ATR, neat) of **4er**

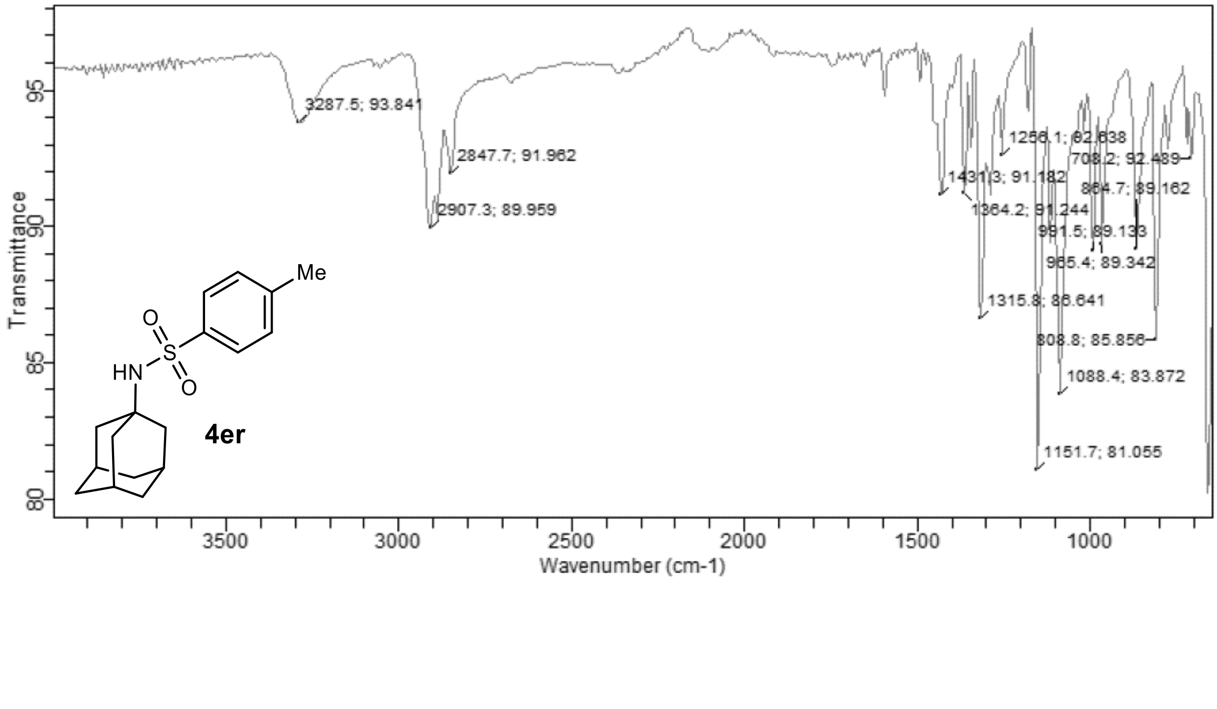

^{1}**H NMR** (400 MHz, CDCl$_3$) of **4fr**

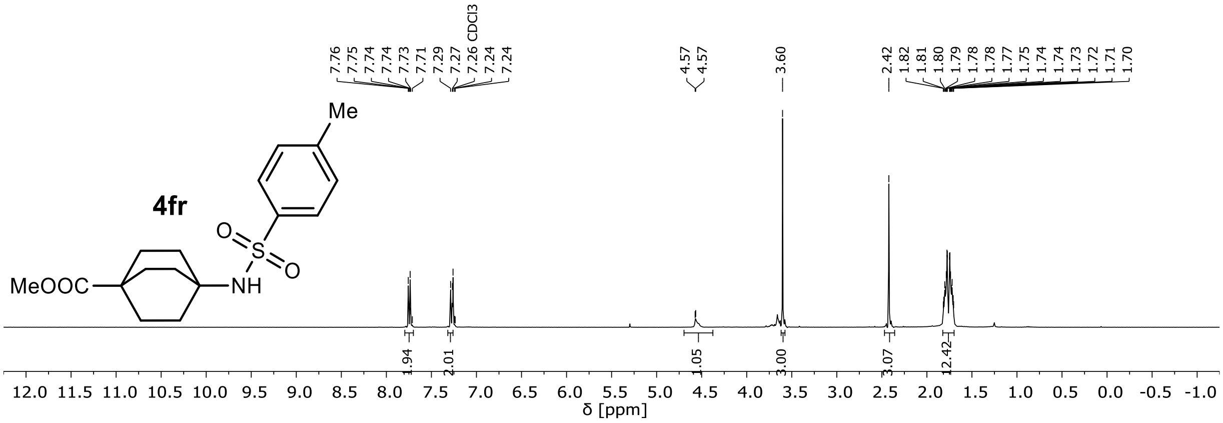

^{13}**C NMR** (101 MHz, CDCl$_3$) of **4fr**

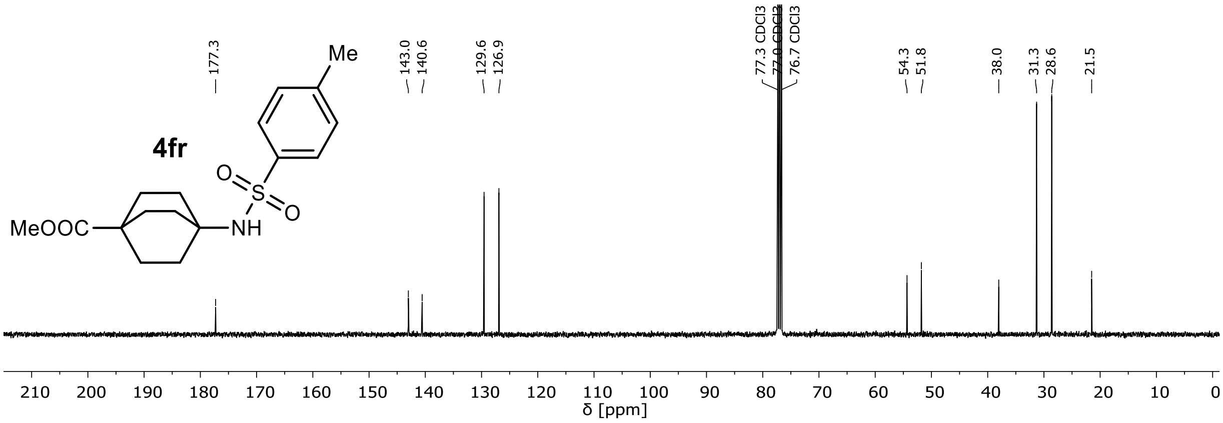

IR (ATR, neat) of **4fr**

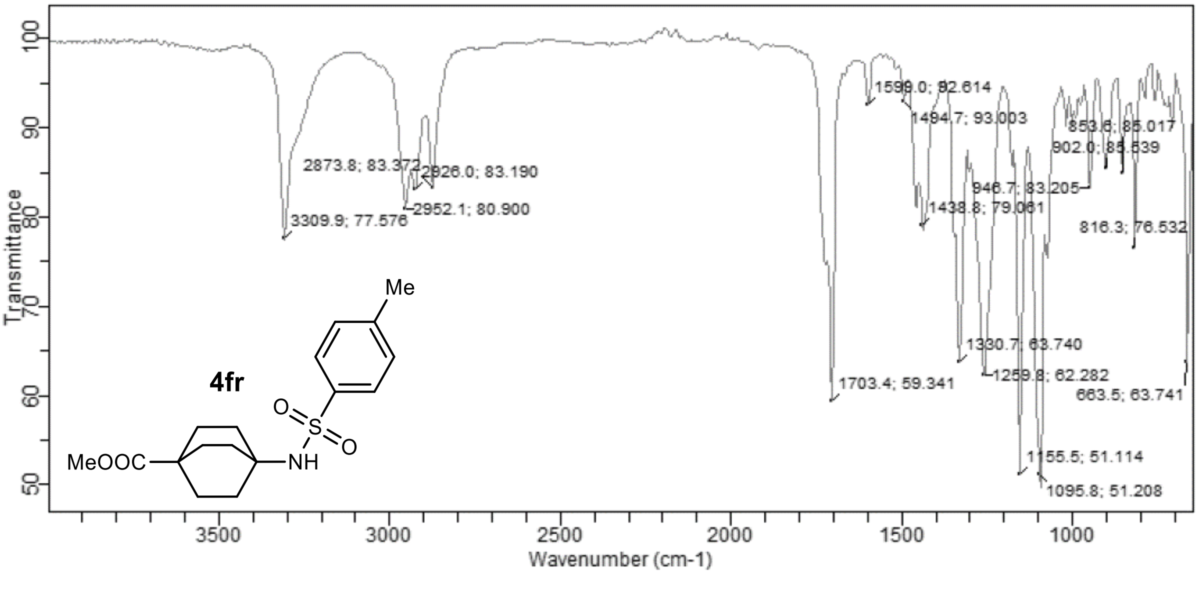

^{1}H NMR (400 MHz, $CDCl_3$) of **4fr'**

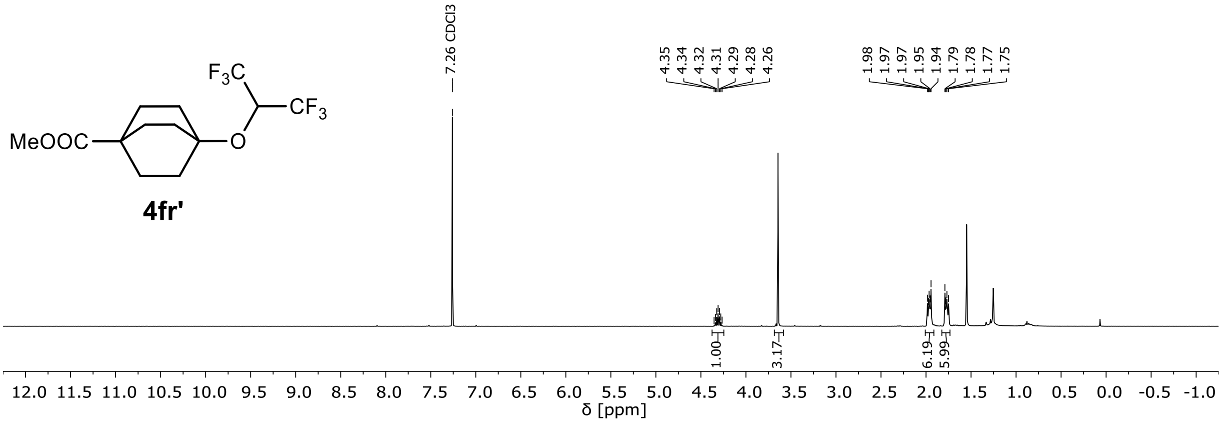

^{13}C NMR (151 MHz, $CDCl_3$) of **4fr'**

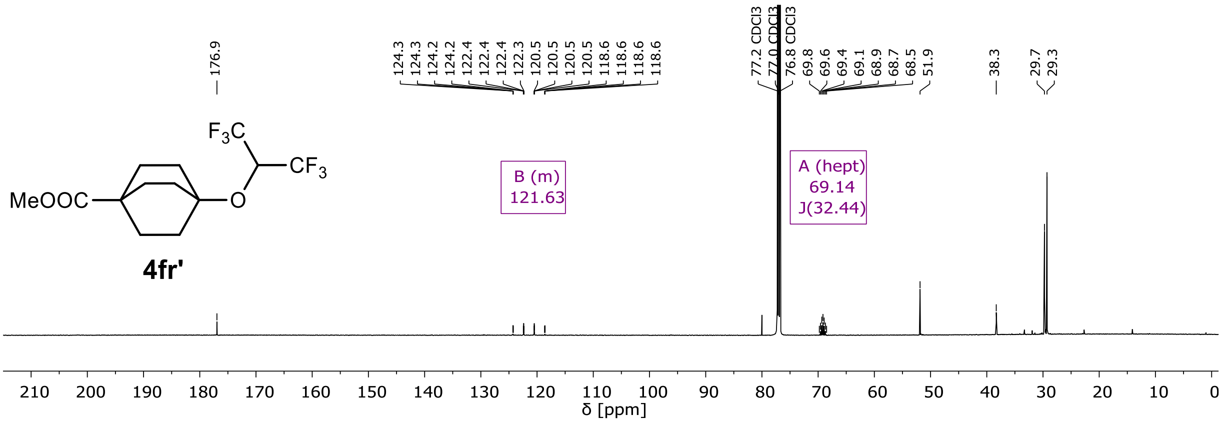

^{19}F NMR (377 MHz, $CDCl_3$) of **4fr'**

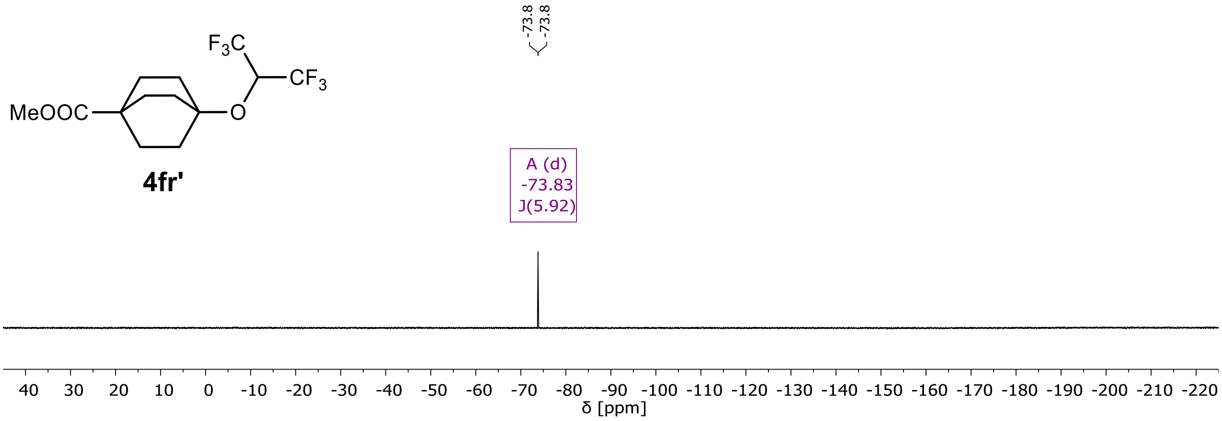

IR (ATR, neat) of **4fr'**

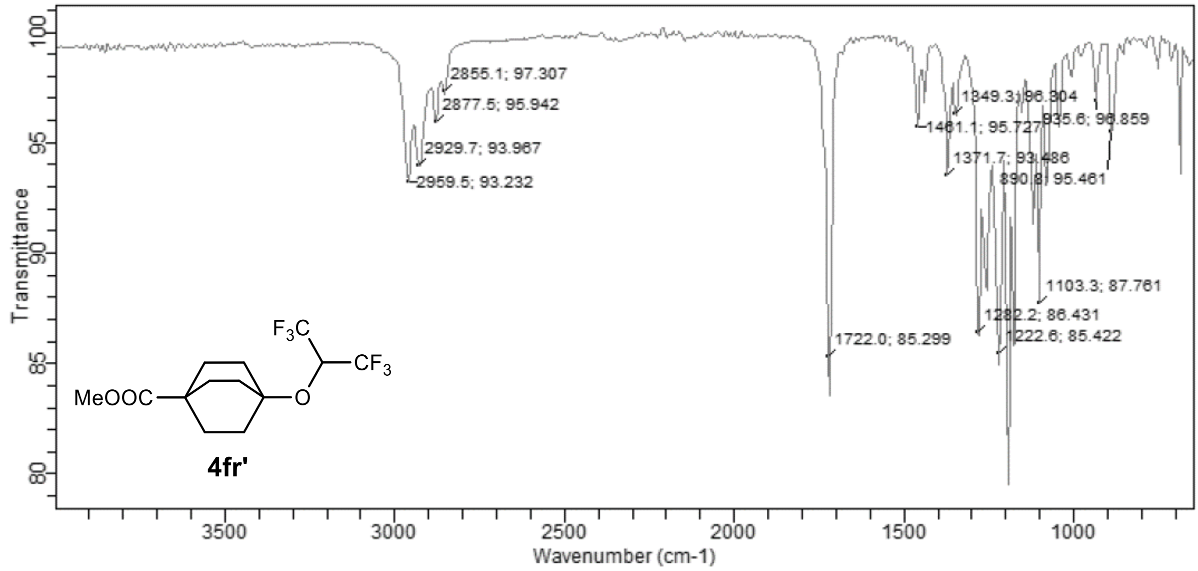

^{1}**H NMR** (400 MHz, $CDCl_3$) of **4aw**

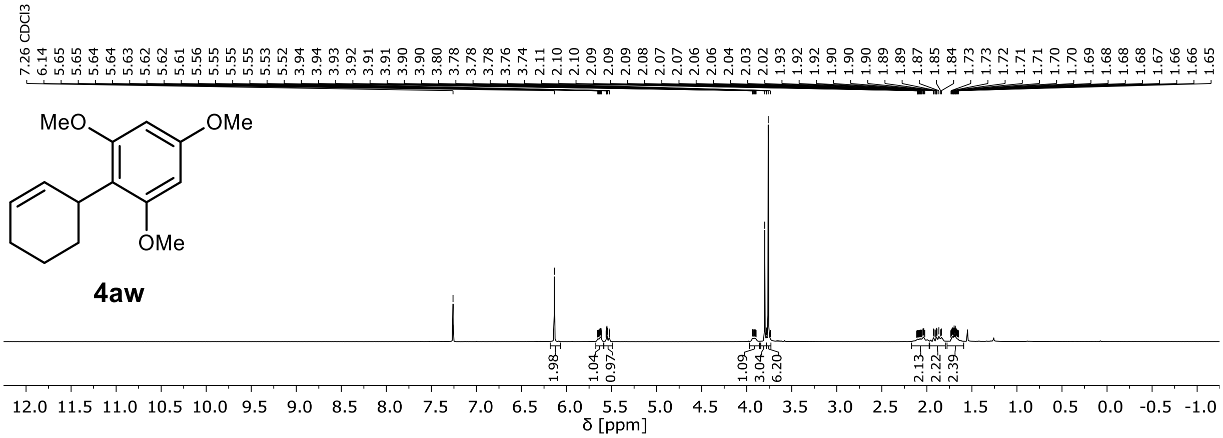

^{13}**C NMR** (101 MHz, $CDCl_3$) of **4aw**

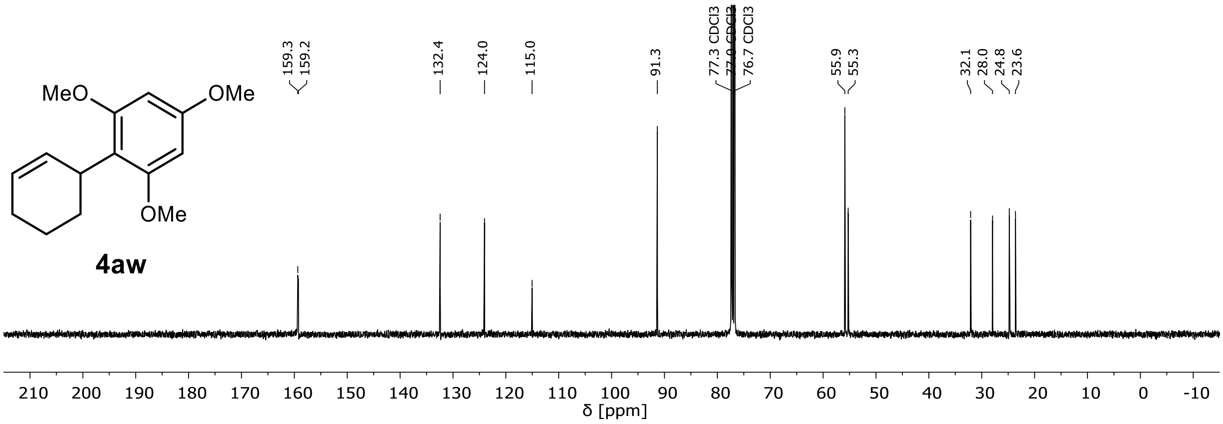

IR (ATR, neat) of **4aw**

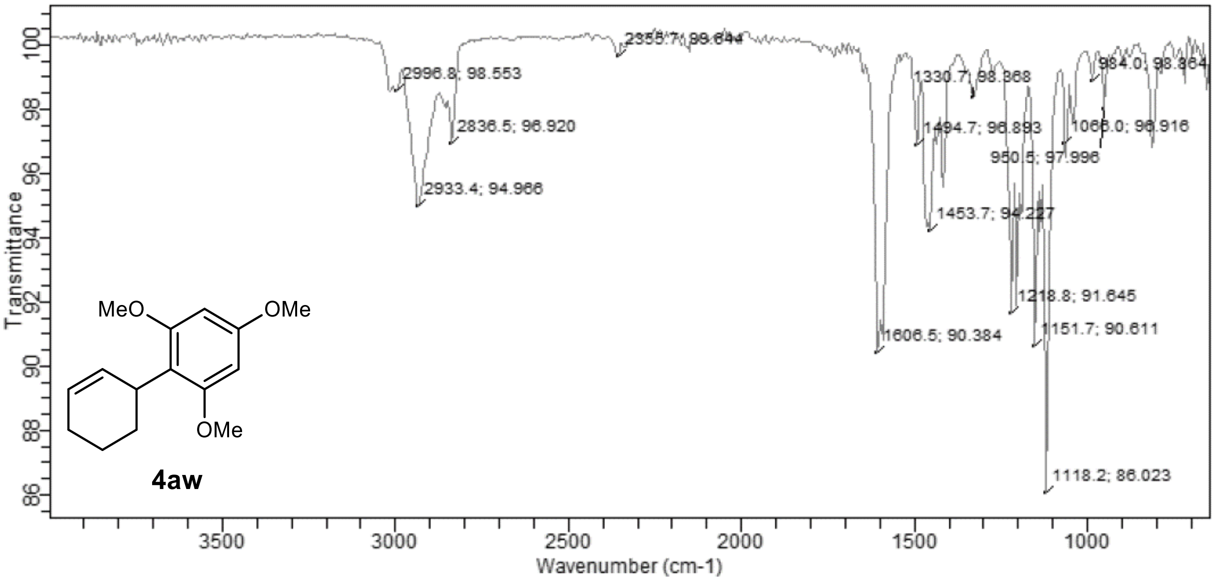

^{1}H NMR (400 MHz, $CDCl_3$) of **4dw**

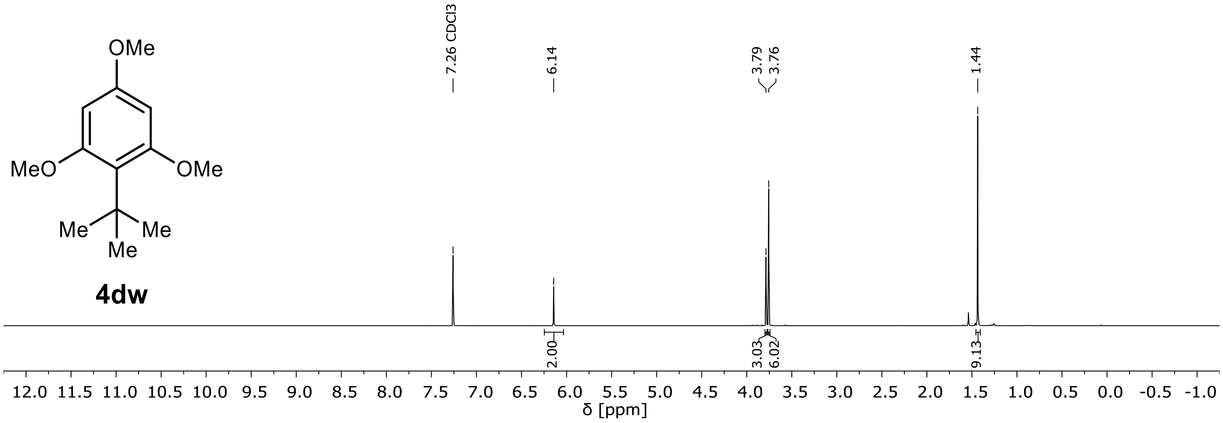

^{13}C NMR (101 MHz, $CDCl_3$) of **4dw**

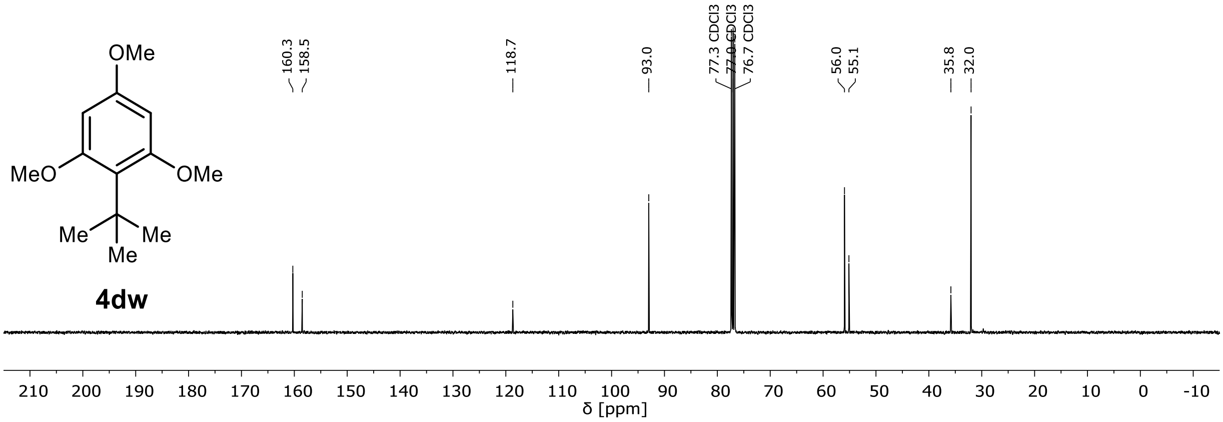

IR (ATR, neat) of **4dw**

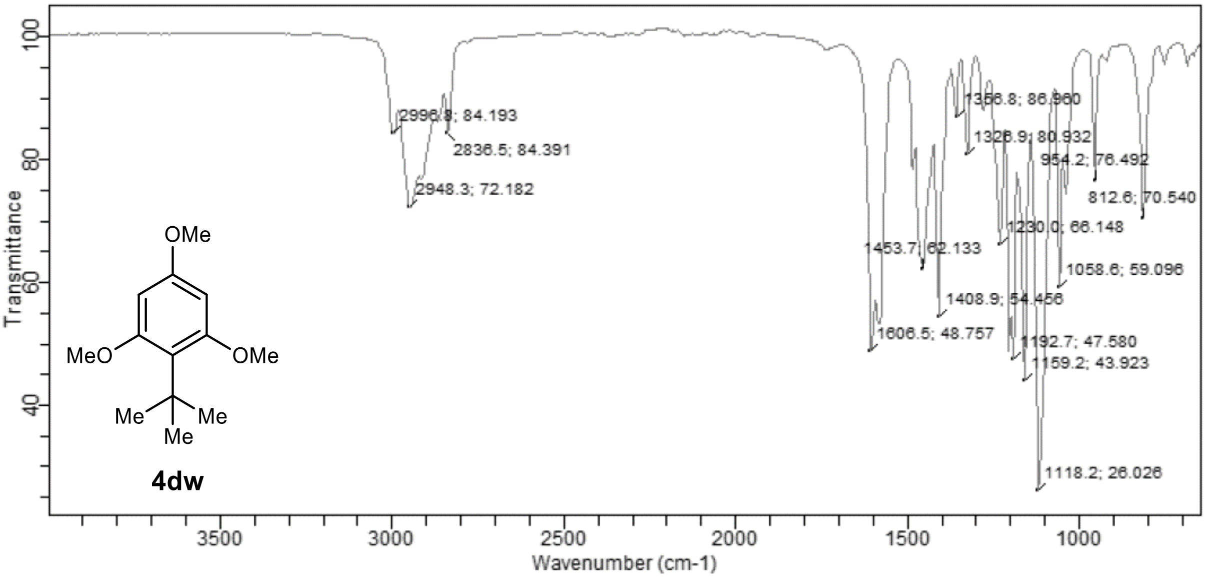

Supplement: Supplementary file 1 — Supplementary Information [file 41586_2024_7622_MOESM1_ESM.pdf]
